# Multi-omics analyses reveal that the gut microbiome and its metabolites promote milk fat synthesis in Zhongdian yak cows

**DOI:** 10.7717/peerj.14444

**Published:** 2022-12-02

**Authors:** Lily Liu, Peifu Wu, Fenfen Chen, Jielong Zhou, Aiwei Guo, Kerong Shi, Qin Zhang

**Affiliations:** 1 Southwest Forestry University, Kunming, Yunnan, China; 2 Shandong Agricultural University, Tai’an, China

**Keywords:** Zhongdian yak cows, Milk fat synthesis, Gut microbiome, Metabolite, Metagenomics, Metabolomics

## Abstract

**Background:**

Yak cows produce higher quality milk with higher concentrations of milk fat than dairy cows. Recently, studies have found the yak milk yield and milk fat percentage have decreased significantly over the past decade, highlighting the urgency for yak milk improvement. Therefore, we aimed to analyze how the gut microbiome impacts milk fat synthesis in Zhongdian yak cows.

**Methods:**

We collected milk samples from Zhongdian yak cows and analyzed the milk fat percentage, selecting five Zhongdian yak cows with a very high milk fat percentage (>7%, 8.70 ± 1.89%, H group) and five Zhongdian yak cows with a very low milk fat percentage (<5%, 4.12 ± 0.43%, L group), and then obtained gut samples of these ten Zhongdian yak cows through rectal palpation. Gut metagenomics, metabolomics, and conjoint metagenomics and metabolomics analyses were performed on these samples, identifying taxonomic changes, functional changes, and changes in gut microbes-metabolite interactions within the milk fat synthesis-associated Zhongdian yak cows gut microbiome, to identify potential regulatory mechanisms of milk fat at the gut microbiome level in Zhongdian yak cows.

**Results:**

The metagenomics analysis revealed *Firmicutes* and *Proteobacteria* were significantly more abundant in the gut of the high-milk fat Zhongdian yak cows. These bacteria are involved in the biosynthesis of unsaturated fatty acids and amino acids, leading to greater efficiency in converting energy to milk fat. The metabolomics analysis showed that the elevated gut metabolites in high milk fat percentage Zhongdian yak cows were mainly enriched in lipid and amino acid metabolism. Using a combined metagenomic and metabolomics analysis, positive correlations between *Firmicutes* (*Desulfocucumis*, *Anaerotignum*, *Dolosiccus*) and myristic acid, and *Proteobacteria* (*Catenovulum*, *Comamonas*, *Rubrivivax*, *Marivita*, *Succinimouas*) and choline were found in the gut of Zhongdian yak cows. These interactions may be the main contributors to methanogen inhibition, producing less methane leading to higher-efficient milk fat production.

**Conclusions:**

A study of the gut microbe, gut metabolites, and milk fat percentage of Zhongdian yak cows revealed that the variations in milk fat percentage between yak cows may be caused by the gut microbes and their metabolites, especially *Firmicutes-*myristic acid and *Proteobacteria-*choline interactions, which are important to milk fat synthesis. Our study provides new insights into the functional roles of the gut microbiome in producing small molecule metabolites and contributing to milk performance traits in yak cows.

## Introduction

The gastrointestinal tract harbors a dynamic and diverse symbiotic microbial community, including more than 99% bacteria and 1% other microorganisms (including archaea, bacteria, viruses and eukaryota), in which bacteria are present at levels of 10^11^–10^14^ cells/ml in colon (Thaiss et al., 2016; Cullender et al., 2013; Mokkala et al., 2021). The gut microbial community plays a major role in regulating metabolic processes, protecting the host from pathogenic microbes, modulating the immune system, and is regarded as an extra endocrine organ (Hagan et al., 2019; Kuipers, De Boer & Staels, 2020). Metagenomics is a powerful tool that can help us to gain a more comprehensive understanding of gut microbes, and research on the role of the human gut microbiome using metagenomics has seen a recent increase (Sipe et al., 2020; Strazar et al., 2021). These metagenomics studies have found a series of functional bacteria that could modulate many diseases and may even be associated with brain development and health through the brain-gut axis.

Milk fat is a high-value food that contains high levels of polyunsaturated fatty acids, which can lower the risk of obesity. Yak cows produce milk with milk fat concentrations 1.89-fold higher than Holstein cow milk (Luo et al., 2018; Ji et al., 2017). Research shows that yak milk production has decreased significantly in the past decade, so there is an urgent need to improve the production and quality of yak milk. Milk fat synthesis and fatty acid composition could be influenced by genetics, diet, and many other factors (Mohan et al., 2021), so current methods of improving milk production in animal agriculture include selective breeding, scientific management, and improvement of feed composition. In recent years, studies have found that rumen microbes are both symbiotic and heritable within an individual animal and could help regulate the milk fat yield and quality by creating nutrients in cows that would otherwise only be found in indigestible food sources (Gillah, Kifaro & Madsen, 2014; Xue et al., 2020). Recent research has also found that feed fermentation by gut microbes is the main contributor to bovine growth and health during early life stages (Fan et al., 2021) and also influences milk composition (Quinn, Joshi & Hickey, 2020). However, there are few studies about the influence of gut microbes on milk fat composition. In this study, using metagenomic and metabolomic analyses, we have made a significant contribution to understanding the gut microbiome and the metabolites regulating the milk fat synthesis in Zhongdian yak cows. Our study explores a new approach that may improve the milk fat yield and quality of Zhongdian yak milk, and our results may also provide opportunities for milk-optimizing breeding programs based on the microbiome.

## Material and Methods

### Housing and feeding systems

Zhongdian yak cows from Shangri-La in the Yunnan province (26−34°N, 94−102°E; 3600 m) were selected and gut samples were collected using rectal palpation as the main research objects. To minimize environmental variation, before sampling commenced, all yak cows foraged freely in the same pasture for at least 14 days.

### Collection of milk and gut samples

All samples from Zhongdian yak cows were collected according to the Animal Welfare Committee of Shandong Agricultural University (permit number SDAUA-2018-022). A total of 36, 8-year-old Zhongdian yak cows from Shangri-La, Yunnan province were selected and milked once daily during October 2021 (mean monthly temperature: 15 °C; altitude: 3,600 m). Milk samples were collected from each cow at milking, and analyzed for fat using mid-infrared instruments (FOSS, Denmark). The five Zhongdian yak cows with the highest milk fat percentage (>7%, 8.70 ± 1.89%, H group) and the five with the lowest milk fat percentage (<5%, 4.12 ± 0.43%, L group) were selected for gut analysis. Gut samples were obtained by rectal palpation and stored in liquid nitrogen until analyzed.

### DNA extraction, metagenomic sequencing, and metagenomics data processing

DNA was extracted using the PowerSoil^®^ DNA Isolation kit (MO BIO Laboratories, Inc., Carlsbad, CA). The quality and quantity of DNA were measured using Qubit V.2.0 fluorometer quantitation (Thermo Fisher Scientific, Inc., Waltham, MA, USA). The genomic DNA was randomly sheared into fragments and the library was constructed using TrueLib DNA Library Rapid Prep Kit for Illumina (ExCell Bio, Shanghai, China). Metagenomic library sequencing was performed on an Illumina Novaseq PE150 (150 bp paired-end sequencing, 500 pb inserts) at Biomarker Technologies Co. Ltd. (Beijing, China).

The trimmomatic (version 0.33) was used to control the quality of each dataset by trimming the 3′-end and the 5′-end of the reads. After cutting low-quality bases (quality scores <20) and removing short reads (<50 bp), the reads were aligned to the bovine genome (bosTau8 3.7, DOI: https://doi.org/10.18129/B9.bioc.BSgenome.Btaurus.UCSC.bosTau8) using bowtie2 (version 2.2.4). MEGAHIT (version 2.2.4) (Li et al., 2015) and QUAST (Gurevich et al., 2013) were used to *de novo* assemble and assess the filtered reads for each sample. MetaGeneMark (version 3.26) (Zhu, Lomsadze & Borodovsky, 2010) was used to predict the coding region from the assembled contigs with length >500 bp. MMseqs (version 4.6.6) (Steinegger & Soding, 2017) was used to pool the assembled contigs and construct non-redundancies based on identical contigs (>95% identity). DIAMOND (version 0.9.24) was used to estimate the abundances after mapping the original sequences to the predicted genes.

### Taxonomic and functional annotation from gut metagenomes

The taxonomic assessment of gut microbiota was performed using DIAMOND (version 0.9.24) against the non-redundant protein sequence database (Nr database) (Yu & Zhang, 2013). Taxonomic profiles were conducted at the phylum, class, family, genus, and species levels, with relative abundances calculated at each level. Microbial taxa with a relative abundance >0.1% in at least 50% of the Zhongdian yak cows in each group were used for downstream analysis. Contigs were annotated using DIAMOND against the EggNOG (Evolutionary Genealogy of Genes: Non-supervised Orthologous Groups, v4.0) (Powell et al., 2014) and KEGG databases (Kyoto Encyclopedia of Genes and Genomes, 2017-03) (Kanehisa et al., 2021) with an *E*-value <1e−5. The CAZymes annotation was performed using hmmer (version 3.0) against the CAZy database (carbohydrate-active enzymes database, version 6.0) (Helbert et al., 2019). In a downstream analysis, the abundances of EggNOG categories, KEGG Orthology (KO) pathways, and CAZymes were normalized into counts per million reads. The EggNOG categories, KEGG pathways, and CAZymes with cpm >5 in at least 50% of the animals in each group were used for the downstream analysis. The microbial network was constructed using the top 80 species, and associations with *R* > 0.5 and *P* < 0.05 were used for the downstream analysis.

### Analysis of metabolites in gut samples

All Zhongdian yak cow gut samples were prepared according to the manufacturer’s instructions of the Waters Acquity I-Class PLUS ultra-high performance liquid tandem Waters Xevo G2-XS QT high resolution mass spectrometer (Macioszek et al., 2021). After freezing in liquid nitrogen, the frozen gut samples were extracted with one mL of extracting solution, and then the samples were sonicated with KQ-500DE ultrasonic cleaner at 0 °C for 15 min, and centrifuged at 4 °C and 12,000 g for 15 min. The supernatants were collected into new tubes, and the extracts were dried in a vacuum concentrator, and then filtered through 0.22 µm nylon membrane filters.

The liquid chromatography was equipped with a Waters Acquity UPLC HSS T3 column (1.8 um 2.1*100 mm). Solvent A was composed of water and 0.1% formic acid, solvent B was composed of acetonitrile and 0.1% formic acid, and the mobile phase was composed of solvent A and solvent B. The gradient profile is outlined as follows: 0–2 min, 98% A; 2–11 min, 2–98% B; 11–13 min, 98% B; 13–15 min, 2–98% A. The flow rate was 200 µL/min, and the injection volume was 10 µL. The sample temperature was maintained at 10 °C, and the column temperature was maintained at 35 °C.

The Waters Xevo G2-XS QTOF high resolution mass spectrometer was used to collect the primary and secondary mass spectrometry data in MSe in the accompanying software (MassLynx V4.2, Waters) and to test metabolites eluted from the column in both positive and negative ion modes. In each data acquisition cycle, dual-channel data acquisition was performed on low collision energy (2V) and high collision energy (10−40 V), with a 0.2 s scan time. The capillary voltages were as follows: 2,000 V in positive ion mode and −1,500 V in negative ion mode. The sampling cone voltage was 30 V. The ion source temperature was set at 150 °C, the desolvent gas temperature was 500 °C, the backflush gas flow rate was 50 L/h, and the desolventizing gas flow rate was 800 L/h.

MassLynx V4.2 was used to collect the raw data. The peak extraction, peak alignment, and other data processing operations were then processed by the Progenesis QI software based on the METLIN database and Biomark’s self-built library for identification. The theoretical fragment identification and mass deviation were all within 100 ppm.

### Statistical significance testing

The milk fat percentage of Zhongdian yak cows was statistically analyzed using the R-package (R v3.02; *P* < 0.05). Gut microbial domains, phyla, genera, and species were compared using a Wilcoxon rank-sum test, with an FDR adjusted *P* value <0.05 considered significantly different (Lin et al., 2021). The abundances of microbial EggNOG categories, KEGG Orthology pathways, and CAZymes were compared between the two groups using a linear discriminate analysis effect size (LEfSe), with an LDA score >2 and *P* value <0.05 indicating significant differences. A T test was performed between the two groups, with an FDR adjusted *P* value <0.05 and VIP (Variable Importance in the Projection) >1 considered significantly different metabolites.

## Results

### Milk fat percentage and the gut metagenome of the selected yak cows

**Figure 1:**
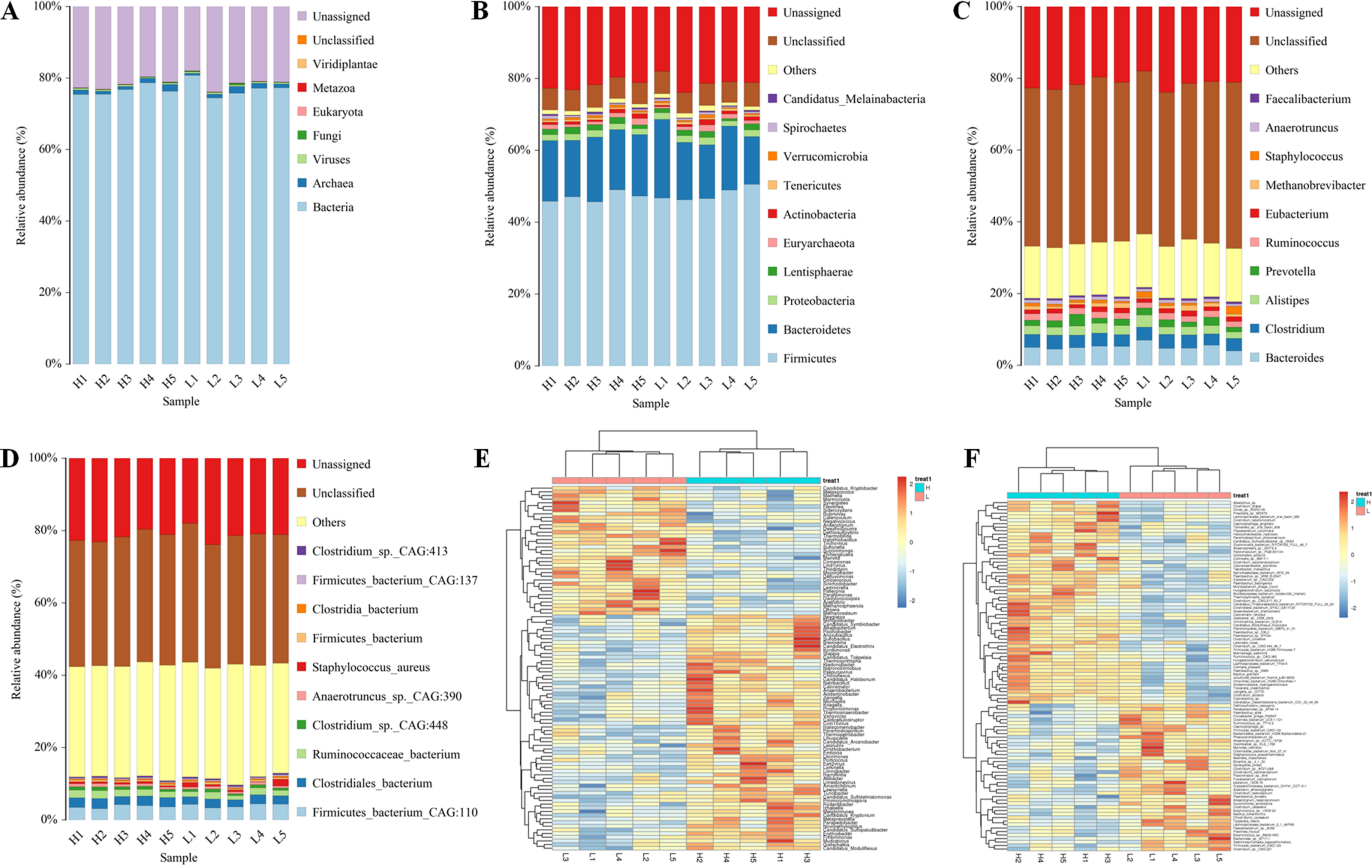
Profiles of gut microbial composition of yak cows and comparison of bacterial genura and species between H and L group using PERMANOVA. Profiles of the gut microbial composition of yak cows and a comparison of the bacterial genera and species between the high milk fat (H) and low milk fat (L) groups using PERMANOVA (permutational multivariate analysis of variance). (A) The gut microbial composition based on kingdom-level taxonomy. (B–D) Bacterial composition based on phylum-, genus- and species-level taxonomy. (E–F) PERMANOVA of genus- and species-level between H and L Zhongdian yak cows.

Five Zhongdian yak cows with high milk fat percentages (8.7 ± 1.89%) and five Zhongdian Yak cows with low milk fat percentages (4.12 ± 0.43%) were selected for gut metagenome analyses (Table 1). After quality control and the removal of host genes, a total of 828,374,098 reads were generated using metagenome sequencing, with 82,837,409.80 ± 6,903,448.80 per sample (10.6084/m9.figshare.19467455; DOI: 10.6084/m9.figshare.19434545). A total of 3,1451,262 contigs were generated (the N50 length of 622 ± 46 bp) after *de novo* assembly, with 3,145,126 ± 223,620 per sample. The gut metagenome of Zhongdian yak cows consisted of 76.81% bacteria, 1.20% archaea, 0.43% viruses, and 0.15% fungi, with 21.36% of the gut metagenome unassigned and 0.51% unclassified (Fig. 1A). The downstream comparison of the gut microbial taxa between the high milk fat (H) and low milk fat (L) Zhongdian yak cows focused on bacteria.

### The gut bacterial composition and differences between the H and L Zhongdian yak cows

Moving forward, we analyzed the composition and differences in the gut bacteria of H and L Zhongdian yak cows (Figs. 1B–1F). In the analysis of the abundance of bacteria, at the phyla level, the dominant bacteria were *Firmicutes* (47.40 ± 1.59%), *Bacteroidetas* (16.82 ± 2.27%), and *Proteobacteria* (1.79 ± 0.19%); at genus level, the dominant bacteria were *Bacteroides* (5.12 ± 0.75%), *Clostridium* (3.56 ± 0.22%), and *Alistipes* (2.45 ± 0.40%); at species level, the dominant bacteria were *Firmicutes_bacterium_CAG:110* (3.85 ± 0.47%), *Clostridiales_bacterium* (2.40 ± 0.27%), and *Ruminococcaceae_bacterium* (1.78 ± 0.32%). We then compared the differences in the gut microbial domains between H and L Zhongdian yak cows using a Wilcoxon rank-sum test, and found significant differences at the genus and species levels (*P* < 0.05). At the genus level, 91 genera, including *Halolamina*, *Aphanocapsa*, and *Limnobacter,* were significantly more abundant in the gut of H Zhongdian yak cows, while 53 genera, including *Epicoccum*, *Haloarcobacter,* and *Palleronia,* showed significant enrichment in the gut of L Zhongdian yak cows. At the species level, 42 species were significantly more abundant in the gut of H Zhongdian yak cows, with the three most significantly elevated being *Ruminococcus_sp._CAG:724*, *Ruminococcus_sp._CAG:382*, and *Acetobacter_sp._CAG:267*. There were 47 species that were significantly less abundant in the gut microbiome of the H group compared to the L Zhongdian yak cows, including *Clostridia_bacterium_UC5.1-1D1*, *Anaerorhabdus_furcosa,* and *Ruminococcus_sp._TF12-2*.

**Table 1:** Summary of sequence data generated from gut samples of ten yak cows.

**Sample ID**	**Milk fat percentage (%)**	**Number of reads**	**Contig number**	**N50 (bp)**
**H1**	11.96 ± 0.02^b^	76,220,843	2927668	628
**H2**	8.52 ± 0.01^b^	84,008,880	3114421	703
**H3**	8.11 ± 0.05^b^	89,352,938	3272755	652
**H4**	7.77 ± 0.01^b^	82,430,625	3225894	634
**H5**	7.14 ± 0.01^b^	77,734,294	3081254	613
**L1**	4.53 ± 0.01^a^	71,605,224	2737081	539
**L2**	4.56 ± 0.04^a^	89,617,998	3319900	630
**L3**	3.70 ± 0.01^a^	91,094,375	3456127	608
**L4**	3.67 ± 0.01^a^	77,047,461	2957049	651
**L5**	4.13 ± 0.01^a^	89,261,460	3359113	562
**Average**		82,837,409.8	3145126.2	622
**Sum**		828,374,098	31451262	6220

### Functional analysis of the gut microbiome and differential functions between H and L Zhongdian yak cows

**Figure 2:**
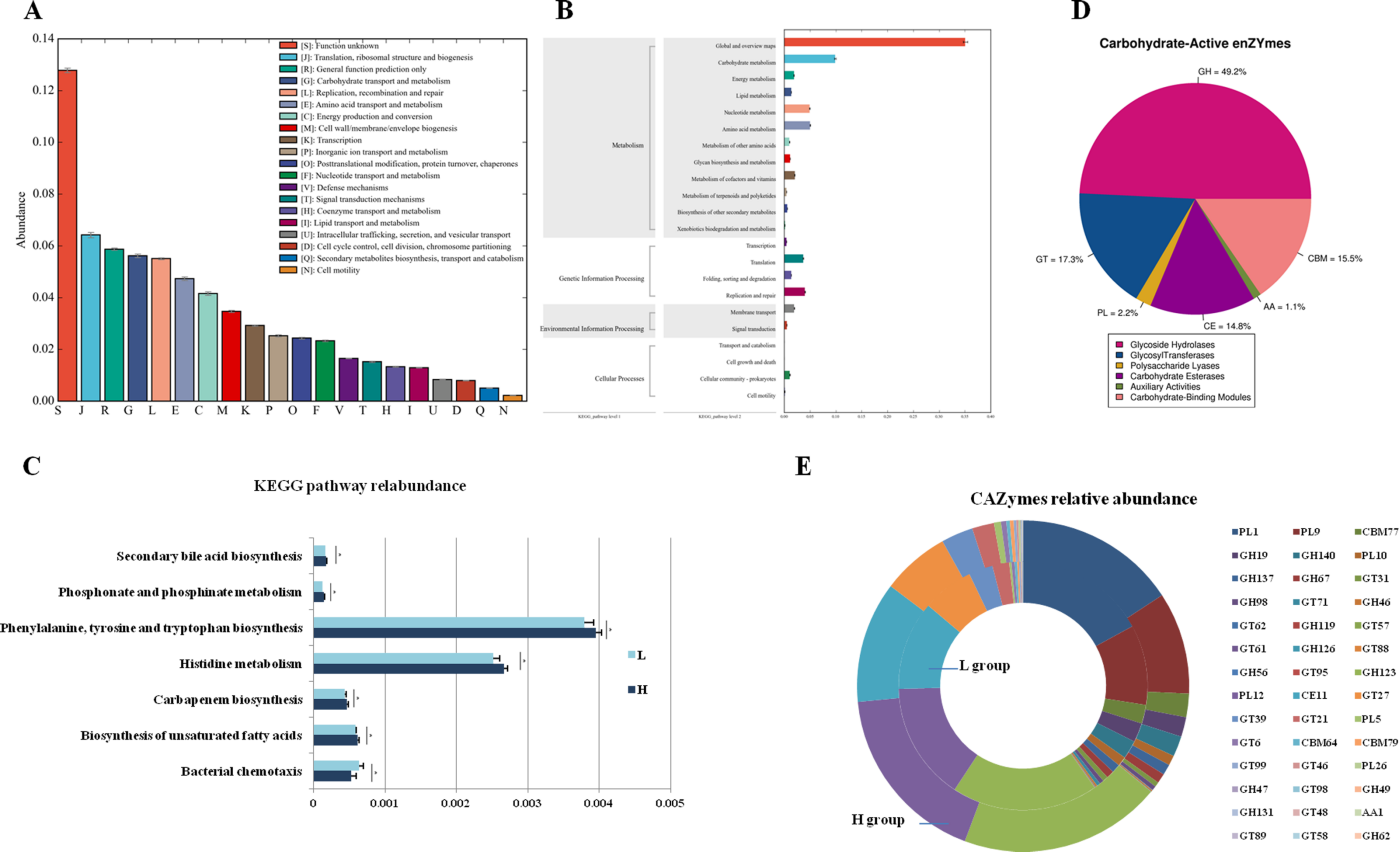
Functions annoation and different functions between H and L group. Functional annotation of different functions between the H and L Zhongdian yak cows. (A–C) EggNOG, KEGG, and CAZymes annotation of gut microbiota. (D–E) Comparison of gut microbial KEGG pathways and CAZymes between H and L Zhongdian yak cows.

To analyze the different functions of the gut microbes between the H and L Zhongdian yak cows, we identified the functions of the gut microbes using EggNOG, KEGG profiles, and CAZy. The EggNOG results identified three main categories including “Metabolism” (22.51 ± 1.83%), “Information storage and processing” (14.87 ± 3.00%), and “Cellular processes and signaling” (10.96 ± 1.12%). A total of 25 EggNOG classes were obtained (Fig. 2A), including “Translation, ribosomal structure and biogenesis” (6.42 ± 0.32%), “Carbohydrate transport and metabolism” (5.62 ± 0.22%), “Energy production and conversion” (4.16 ± 0.21%), and “Lipid transport and metabolism” (1.29 ± 0.05%). There were no differences in the EggNOG classes identified between the H and L Zhongdian yak cows.

The KEGG analysis identified 175 endogenous third-level pathways belonging to four first-level categories (Fig. 2B) including “Metabolism” (77.14%), “Genetic information processing” (12%), “Cellular processes” (6.86%), and “Environment information processing” (4%). At the second level, 22 categories were obtained, with “Xenobiotics biodegradation and metabolism” (9.71%), “Lipid metabolism” (8.57%), “Carbohydrate metabolism” (8.57%), “Amino acid metabolism” (8.00%), and “Metabolism of terpenoids and polyketides” (8.00%) being the most abundant. We then compared the identified KEGG pathways (Fig. 2C), and found five pathways that were significantly enriched in the H gut microbiome, including “Phosphonate and phosphinate metabolism” (*P* = 0.006, *df* = 8), “Carbapenem biosynthesis” (*P* = 0.023, *df* = 8), “Histidine metabolism” (*P* = 0.014, *df* = 8), “Biosynthesis of unsaturated fatty acids” (*P* = 0.029, *df* = 8), and “Biosynthesis of ansamycins” (*P* = 0.008, *df* = 8); while only one pathway, “Bacterial chemotaxis” (*P* = 0.028, *df* = 8), was significantly enriched in the gut of L Zhongdian yak cows.

The CAZyme analysis identified 125 glycoside hydrolases (GHs), 85 glycosyltransferases (GTs), 79 carbohydrate-binding modules (CBMs), 16 carbohydrate esterases (CEs), 23 polysaccharide lyases (PLs), and 11 auxiliary activities (Poon et al., 2021) (Fig. 2D). The five most dominant CAZymes were GT2 (0.21 ± 0.01%), GH13 (0.17 ± 0.01%), GH109 (0.17 ± 0.01%), GT4 (0.12 ±0.00%), and CE1 (0.11 ± 0.00%). Among all the CAZymes identified, 22 were significantly enriched in the gut of H Zhongdian yak cows (*P* < 0.05), including GH123 (*P* = 0.007, *df* = 8), PL12 (*P* = 0.031, *df* = 8), and CE11 (*P* = 0.012, *df* = 8), and 20 CAZymes were significantly enriched in the gut of L Zhongdian yak cows (*P* < 0.05), including PL1 (*P* = 0.028, *df* = 8), PL9 (*P* = 0.016, *df* = 8), and CBM77 (*P* = 0.019, *df* = 8). There were significantly more CAZymes identified in H Zhongdian yak cows than in L yak cows (Fig. 2E).

### Differences analysis of association networks of the gut microbiome in H and L Zhongdian yak cows

**Figure 3:**
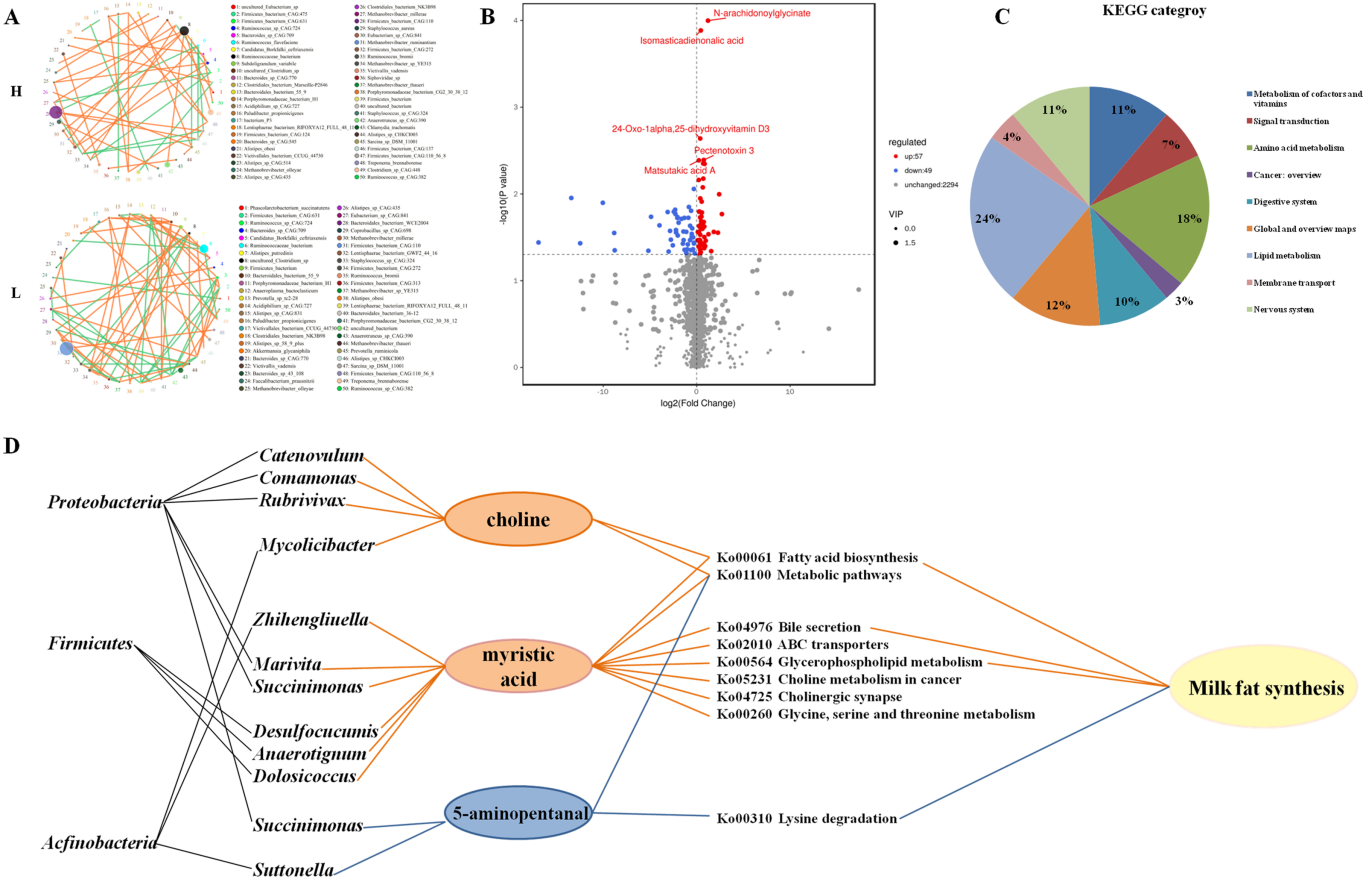
Microbial networks and association of different metabolites with microbial in the gut of H and L yak cows. Microbial networks and association of different metabolites with microbes in the gut of H and L Zhongdian yak cows. (A) The microbial networks of H and L Zhongdian yak cows (light orange: positive; light green: negative). (B) Volcano plot of different metabolites in H and L Zhongdian yak cows (red: up-regulated; blue: down-regulated). (C) KEGG categories of different metabolites in H and L Zhongdian yak cows. (D) Correlation networks showing associations between significantly different metabolites and bacterial species and phyla (orange: significantly enriched in (H) blue: significantly enriched in L).

Microbial network for both H and L yak cows were constructed at the species level based on the abundance and correlation of gut microbes (Fig. 3A). Both positive and negative associations were identified in both networks. Notably, in both H and L Zhongdian yak cows, the gut microbial network contained a greater number of positive associations than negative associations. Interestingly, the number of negative associations in H Zhongdian yak cows was significantly less than the number of positive associations in L Zhongdian Yak cows, and the majority of associations found within both networks were unique to that network. We hypothesized that this dramatic difference may reflect changes in the ecosystem associated with milk fat synthesis. We then analyzed the taxonomic relationships between the species within both networks. The microbial networks in the gut of both H and L Zhongdian yak cows exhibited a large abundance of taxonomically distant networks, and many of the bacterial associations identified were unique to that network. Notably, the negative associations found in H Zhongdian yak cows occurred between species that were taxonomically similar.

Our analysis of microbial networks found that *Ruminococcaceae_bacterium* and *Clostridiales_bacterium* were positively associated with other species in H Zhongdian yak cows, and negatively correlated with other species in L Zhongdian yak cows; *Firmicutes_bacterium* exhibited a negative association with other species in H Zhongdian yak cows and a positive association with other species in L Zhongdian yak cows. In H Zhongdian yak cows, there were unique positive correlations between *Anaerotruncus_sp_CAG:390* and *Clostridium_sp_CAG:413,* and between *Sarcina_sp_DSM_11001* and other species, and a unique negative correlation between *Firmicutes_bacterium_CAG:110_56_8* and *Firmicutes_bacterium_CAG:631*. All species that exhibited unique correlations with others in H Zhongdian yak cows belonged to the phylum *Firmicutes*. This may indicate that these are influential bacterial species within the networks and that they may play important roles in milk fat synthesis in Zhongdian yak cows.

### Gut metabolome analysis

A total of 2,400 compounds were identified in the gut metabolome. Using the *T*-test and Variable Importance in the Projection (VIP) to filter the relative concentrations of gut metabolites, 57 metabolites were significantly higher and 49 metabolites were significantly lower in the gut of H Zhongdian yak cows (*P* < 0.05, VIP >1, Fig. 3B). The KEGG annotation based on these 106 significantly different gut metabolites revealed the enrichment of nine first-level categories in H Zhongdian yak cows (Fig. 3C), including “Lipid metabolism” (23.61%), “Amino acid metabolism” (18.06%), “Global and overview maps” (12.5%), “Nervous system” (11.11%), “Metabolism of cofactors and vitamins” (11.11%), “Digestive system” (9.72%), “Signal transduction” (6.94%), “Membrane transport” (4.17%), and “Cancer: overview” (2.78%). Of these nine pathways, only “Metabolism of cofactors and vitamins” and “Signal transduction” were also enriched in L Zhongdian yak cows. To further identify the potential gut microbiome-metabolome interactions, a Spearman’s rank correlation and constrained correspondence analysis between the gut metabolome and microbiome were performed, with the results revealing 27 significant correlations (—CC—>0.80, CCP <0.05). Among these 27 correlations, 12 positive correlations were identified between: myristic acid and six genera, choline and four genera, and 5-aminopentanal and two genera (Fig. 3D). *Firmicutes* and *Proteobacteria* were the dominant bacterial phyla in the gut microbiome of H Zhongdian yak cows. These results indicate that the regulation of *Firmicutes* (*Desulfocucumis*, *Anaerotignum*, *Dolosiccus*)-myristic acid and *Proteobacteria* (*Catenovulum*, *Comamonas*, *Rubrivivax*, *Marivita*, *Succinimouas*)-choline interactions may be the main contributors to milk fat synthesis in Zhongdian yak cows.

## Discussion

In this study, controlling for other factors affecting milk fat synthesis in Zhongdian yak cows including feed, management, age, and lactation stage, we found that variations in milk fat could be attributed to variations in the microorganisms and metabolites in the gut. By analyzing the gut metagenome and metabolome, we investigated the microbial and metabolomic mechanisms in the gut that contribute to milk fat synthesis in Zhongdian yak cows and estimated the impact of metabolomic and microbial composition and functions on variations in milk fat. Similar to previous studies (Zeng et al., 2019; Li et al., 2020) that have assessed gut microbes using metagenomics, we identified the gut microbes at multiple levels, including the eukaryote and virus levels. Bacteria was the most abundant gut microbe in both H and L Zhongdian yak cows, which suggests that bacteria play a more significant role in milk fat synthesis in Zhongdian yak cows than other microbial kingdoms. Although we did not focus on eukaryotes or viruses in this study, their interactions and associations with bacteria could also be important factors affecting milk traits, and future studies should focus on this possibility.

Our study showed that the bacterial features in the gut of H and L Zhongdian yak cows differed at various taxonomic levels. At the species level, most of elevated species in H Zhongdian yak cows belonged to the genus *Clostridium* and genus *Ruminococcus*. The *Clostridium* and *Ruminococcus* genera both belong to the phylum *Firmicutes*, and are ubiquitous catalase-negative and oxidase-negative bacterium in the human gastrointestinal tract and in the rumen microbiome. They play an important role in the fermentation of cellulose rich feedstuffs and resistant starch, and can produce acetic acid and butyric acid to increase volatile fatty acid production, acting as butyrate and propionate producers (Gaffney et al., 2021; Maifeld et al., 2021). In our study, there was a higher abundance of *Clostridium* and *Ruminococcus* genera found in the gut of H Zhongdian yak cows, indicating that the production of more volatile fatty acid may contribute to milk fat synthesis *via* the gut-blood axis. These genera may be the main contributors to the higher milk fat percentage found in H Zhongdian yak cows.

After performing the taxonomic composition analysis, we sought to identify the functions of the gut microbes to help explain the differences between H and L Zhongdian yak cows. Our EggNOG analysis found no difference between H and L Zhongdian yak cows. The KEGG analysis, however, found that functions tied to fatty acids and amino acid biosynthesis were significantly elevated within the gut microbiome of H Zhongdian yak cows. Fatty acids and amino acids are important for short chain fatty acid production, which can increase insulin sensitivity and gut immunity through energy metabolism and by regulating hormone production and the absorption of a variety of nutrients in the intestinal tract (Castro et al., 2015; Hu et al., 2018; O’Hara et al., 2020). Our results suggest that the gut microbiome of H Zhongdian yak cows may promote the conversion of food into both energy and the required nutrients for milk fat. The CAZymes involved in deconstructing carbohydrates were more enriched in the gut of H Zhongdian yak cows than in L Zhongdian yak cows, providing further evidence that H Zhongdian yak cows were more capable of degrading complex substrates. Overall, the gut microbes of H Zhongdian yak cows might be more capable of and efficient in converting energy into milk fat than the gut microbes of L Zhongdian yak cows.

Most of the bacterial association networks identified were unique in H and L Zhongdian yak cows, suggesting that there was a large difference in the bacterial community structure of H- and L-associated gut microbes. More negative associations were identified between species that were taxonomically similar in the gut microbiome of H Zhongdian yak cows compared to the L gut microbiome, suggesting that there was more competitive exclusion between dissimilar species within the gut microbe of L Zhongdian yak cows. Additionally, we found that “bacterial chemotaxis” was enriched in the gut microbiome of L Zhongdian yak cows. Chemotaxis functions allow more microbes to move toward better nutritional conditions by enhancing the ability to sense chemical gradients (Mohan et al., 2021; Wallace et al., 2019). The fact that more correlations were found between dissimilar bacteria species in L Zhongdian yak cows than in H yak cows provides more evidence that bacterial diversity may play an important role in milk fat synthesis. Guan et al. (2008) found there was a greater similarity in the microbial profiles of the cattle that were better able to convert feed to useable products. There is increasing evidence that gut microbes are actively involved in immune functions, directly contributing to animal gut health in addition to their roles in feed digestion and energy production (Malmuthuge & Guan, 2017; Mulder et al., 2011; Malmuthuge et al., 2013). Taken together, our observations suggest that the similar gut microbes we observed in H Zhongdian yak cows may play important roles in milk fat synthesis by impacting gut health. Interestingly, correlations between *Clostridium_sp_CAG:413*, *Sarcina_sp_DSM_11001*, *Firmicutes_bacterium_CAG:110_56_8,* and *Firmicutes_bacterium_CAG:631* with other species were only observed in the network of H Zhongdian yak cows. *Ruminococcaceae_bacterium* and *Clostridiales_bacterium* were positively associated with other species in H Zhongdian yak cows, and negatively correlated with other species in L yak cows. These species all belong to the phylum *Firmicutes*. *Firmicutes* is one of the major microbes in the rumen and gut. Its microbial cohort contains both lipolytic and cellulolytic species. *Firmicutes* is capable of effectively degrading host-indigestible plant fiber and is vital to the provision of microbial proteins for muscle and milk synthesis (Firkins & Yu, 2015; Stojanov, Berlec & Strukelj, 2020). The results of our study support the possibility that *Firmicutes* may also contain functional capabilities better suited for milk fat synthesis in Zhongdian yak cows.

By identifying the metabolites that differed between the two groups, we found that the elevated metabolites in H Zhongdian yak cows were mainly involved in fatty acid biosynthesis, especially myristic acid and choline, which were over 2-fold higher in the gut of H Zhongdian yak cows and were correlated with *Firmicutes* and *Proteobacteria,* respectively. Additionally, 5-aminopentanal was higher in the gut of L Zhongdian yak cows and was correlated with *Proteobacteria*. Choline and myristic acid are involved in bile secretion, glycerophospholipid metabolism, and fatty acid biosynthesis, and 5-aminopentanal plays a significant role in lysine degradation. Previous studies have demonstrated that milk fat synthesis is sensitive to choline and lysine supplementation (Lashkari et al., 2020; Elek et al., 2008; Fehlberg et al., 2020), and myristic acid could inhibit methanogen activity, leading to higher efficiency in milk fat production (Newsome et al., 2021). The higher choline and myristic acid and lower 5-aminopentanal observed in the H Zhongdian yak cows in our study support our hypothesis that the gut microbiome of H Zhongdian yak cows is able to provide more metabolic energy to mammary glands for milk fat synthesis. Overall, our study suggests that both the gut microbiome and metabolome influenced milk fat synthesis in Zhongdian yak cows, and the interactions between the gut microbes and metabolites suggest that the interactions between *Firmicutes* (*Desulfocucumis*, *Anaerotignum*, *Dolosiccus*) and myristic acid and between *Proteobacteria* (*Catenovulum*, *Comamonas*, *Rubrivivax*, *Marivita*, *Succinimouas*) and choline may be crucial contributors to milk fat synthesis. The relationships between the gut microbes, metabolites, and their functions provide new insights into the functional roles of the gut microbes in producing small molecule metabolites and in contributing to milk performance traits in Zhongdian yak cows.

This study also has limitations. Even through the microbiome is known to play an important role in milk fat synthesis in dairy cows, there are no studies on the regulation of the gut microbiome on milk fat synthesis in other animals. Because of this, we are unable to identify the specific mechanisms of the alterations observed in the gut microbes and their metabolites, particularly the interactions of *Firmicutes*-myristic acid and *Proteobacteria*-choline in regulating milk fat synthesis in Zhongdian yak cows without further research. However, there are many studies that show that myristic acid and choline are associated with milk fat synthesis in dairy cows. In future studies, we hope to verify these results with a larger sample size. We also hope to study possible changes in blood metabolites and in gene expression levels in mammary tissues to help identify the mechanisms of the *Firmicutes* (*Desulfocucumis*, *Anaerotignum*, *Dolosiccus*) -myristic acid and *Proteobacteria* (*Catenovulum*, *Comamonas*, *Rubrivivax*, *Marivita*, *Succinimouas*) –choline interactions in regulating milk fat synthesis in Zhongdian yak cows.

## Conclusion

Our study identified the gut microbial taxonomic features, functions, and metabolites as well as the interactions between these that contribute to milk fat synthesis in Zhongdian yak cows. Yak cows with higher milk fat percentages (H) had higher abundances of *Firmicutes* and *Proteobacteria* contributing to differences in the metabolites absorbed and transported, leading to higher efficiency in energy utilization for milk fat biosynthesis. The microorganisms identified in the gut of H Zhongdian yak cows were stronger choline and myristic acid producers, which are associated with higher milk fat percentages. Altogether, our multi-omics analysis revealed that the gut microbial metabolites had a larger impact on milk fat synthesis than gut microbial composition or microbial functions. The microbial and metabolomic mechanisms contributing to milk fat synthesis identified in this study provide insights into strategies involving the alteration of gut microbes for higher milk fat quality and production through either feeding management or genetic selection.

##  Supplemental Information

10.7717/peerj.14444/supp-1 Supplemental Information 1 The raw data of bacterial composition based on the phylum

10.7717/peerj.14444/supp-2 Supplemental Information 2 The raw data of bacterial composition based on the genus

10.7717/peerj.14444/supp-3 Supplemental Information 3 The raw data of gut microbial composition based on the kingdom-level taxonomy

10.7717/peerj.14444/supp-4 Supplemental Information 4 The raw data of bacterial composition based on the genus

10.7717/peerj.14444/supp-5 Supplemental Information 5 The raw data of PERMANOVA analysis of genus level between H and L group

10.7717/peerj.14444/supp-6 Supplemental Information 6 The raw data of PERMANOVA analysis of species level between H and L group PERMANOVA analysis of species level between H and L group

10.7717/peerj.14444/supp-7 Supplemental Information 7 The raw data of eggNOG annoation of gut microbial

10.7717/peerj.14444/supp-8 Supplemental Information 8 The raw data of KEGG annoation of gut microbial

10.7717/peerj.14444/supp-9 Supplemental Information 9 The raw data of CAZymes annoation of gut microbial

10.7717/peerj.14444/supp-10 Supplemental Information 10 The raw data of network in H group

10.7717/peerj.14444/supp-11 Supplemental Information 11 The raw data of network in L group

10.7717/peerj.14444/supp-12 Supplemental Information 12 The raw data of metabolom

10.7717/peerj.14444/supp-13 Supplemental Information 13 The raw data of conjoint analysis between gut metagenomics and metabolome

10.7717/peerj.14444/supp-14 Supplemental Information 14 The milk fat percentage of the selected ten yak cows in Day 1 to Day 14

10.7717/peerj.14444/supp-15 Supplemental Information 15 Author checklist

## Additional Information and Declarations

### DNA Deposition

The following information was supplied regarding the deposition of DNA sequences: The bovine gut metagenome raw sequence reads are available at NCBI: PRJNA822929.

### Data Availability

The following information was supplied regarding data availability: The bovine gut metagenome raw sequence reads are available at NCBI: PRJNA822929. The analyzed data is available at Figshare: Liu, Lily; Wu, Peifu; Chen, Fenfen; Zhou, Jielong; Gao, Aiwei; Shi, Kerong; et al. (2022): Multi-omics reveals that the gut microbiome and its metabolite promotes milk fat synthesis in Zhongdian yak cows. figshare. Dataset. https://doi.org/10.6084/m9.figshare.19434545.v1. The sequencing data and asssembly result of metagenome are available at figshare: Liu, Lily; Wu, Peifu; Chen, Fenfen; Zhou, Jielong; Guo, Aiwei; Shi, Kerong; et al. (2022): Multi-omics reveals that the gut microbiome and its metabolite promotes milk fat synthesis in Zhongdian yak cows. figshare. Dataset. https://doi.org/10.6084/m9.figshare.19467455.v1.
