# Supplementary figures and images for: Multi-omics analyses reveal that the gut microbiome and its metabolites promote milk fat synthesis in Zhongdian yak cows (part 1 of 2)
Source: PeerJ. 2022 Dec 2;10:e14444. doi: 10.7717/peerj.14444 (PMC9744170; doi:10.7717/peerj.14444)

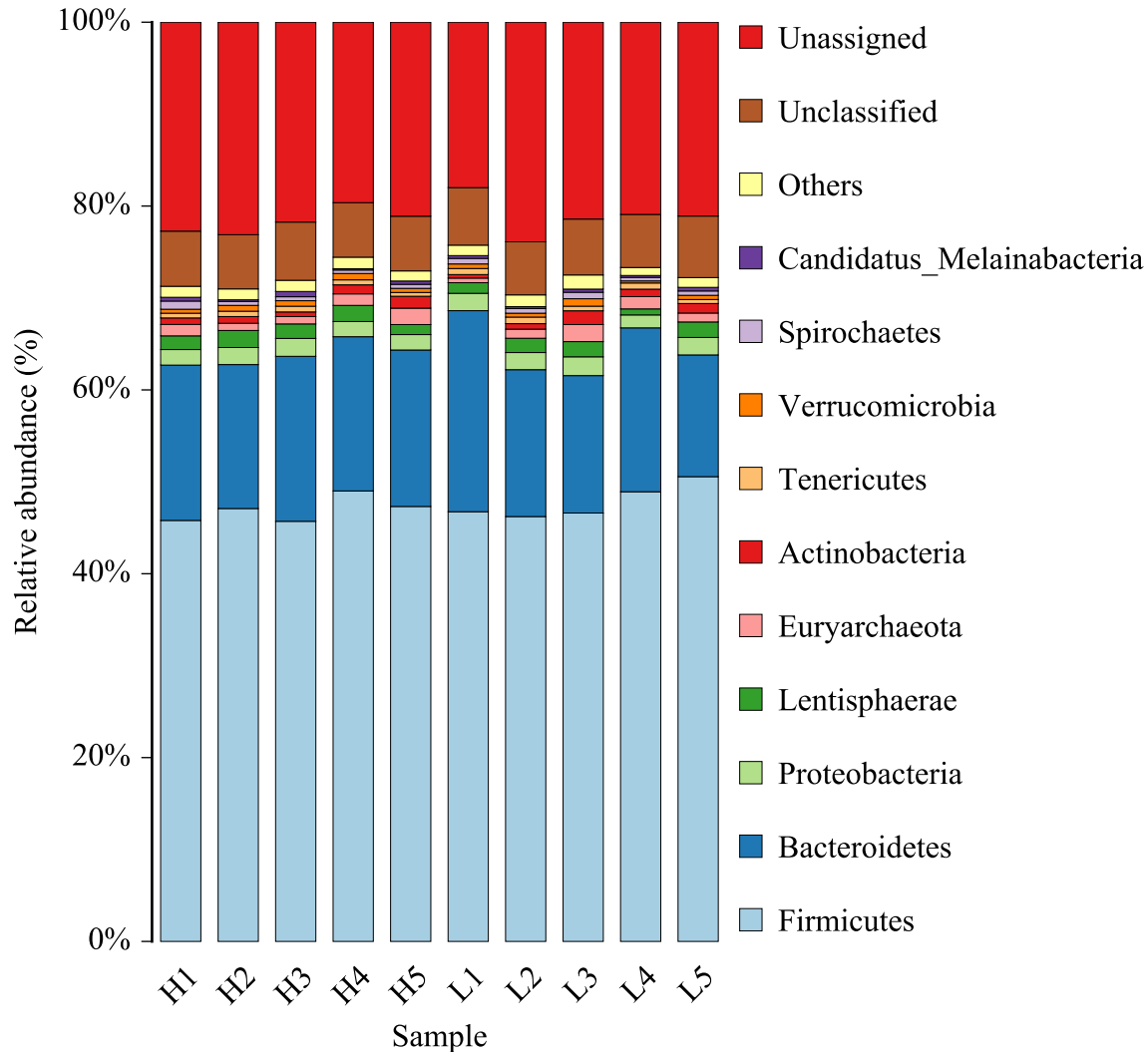

Supplement: Supplemental Information 1 [file peerj-10-14444-s001.pdf]

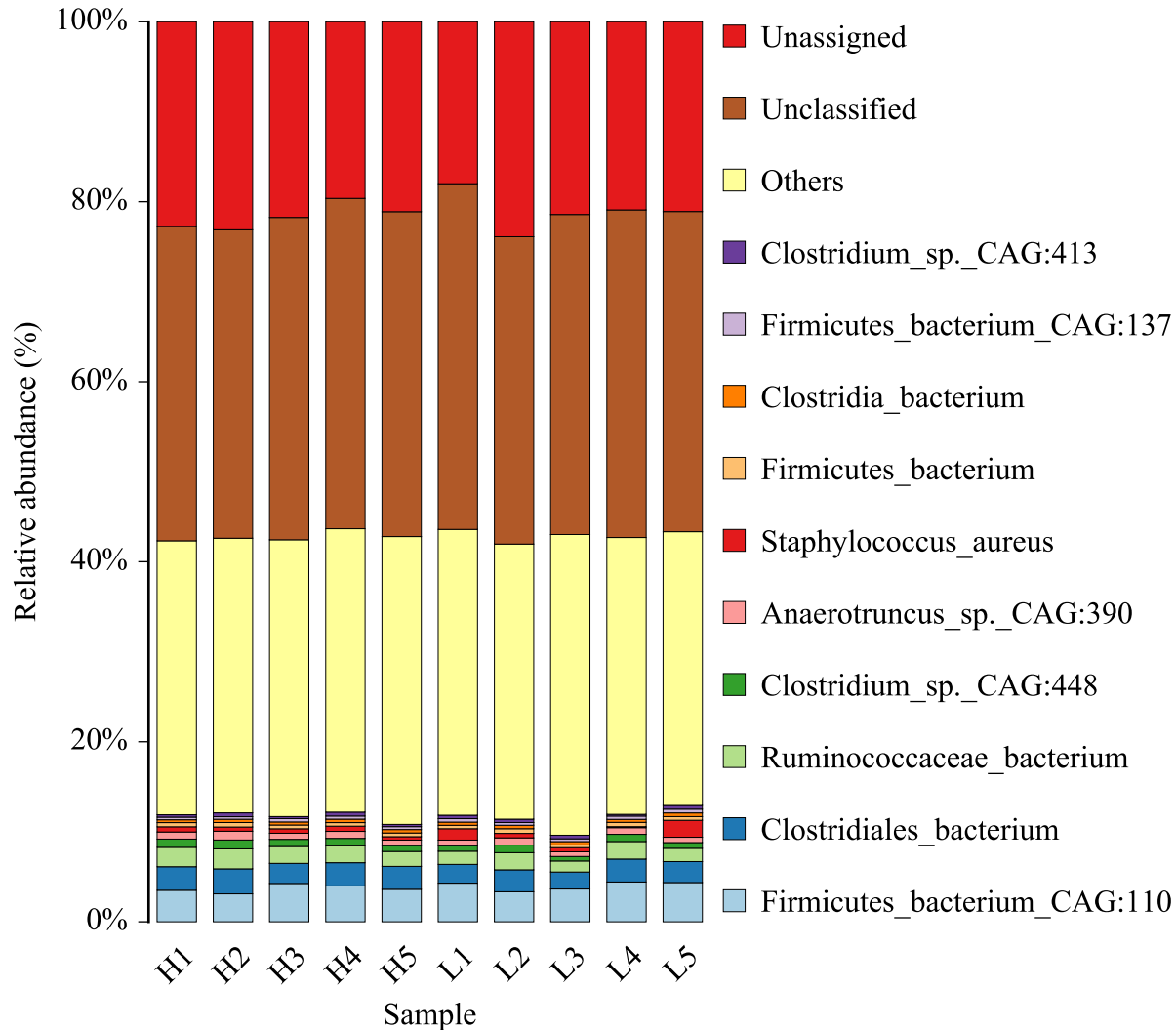

Supplement: Supplemental Information 2 [file peerj-10-14444-s002.pdf]

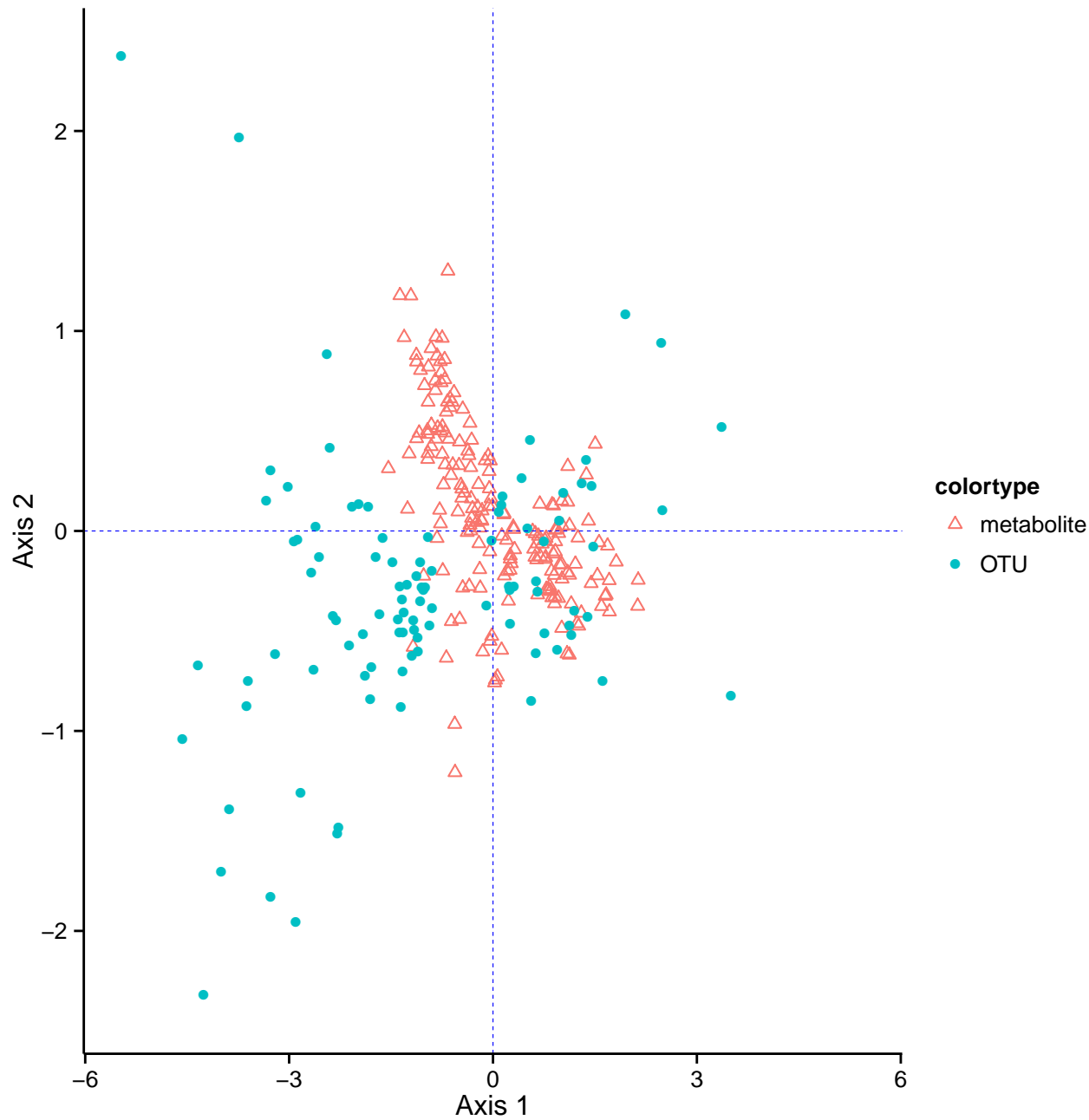

Supplement: Supplemental Information 12 [file peerj-10-14444-s012.zip › Web_Report/coinertia_analysis/taxonomy/treat1_H_L.vs.H_L/treat1_H_L.vs.H_L_coinertia_analysis.pdf]

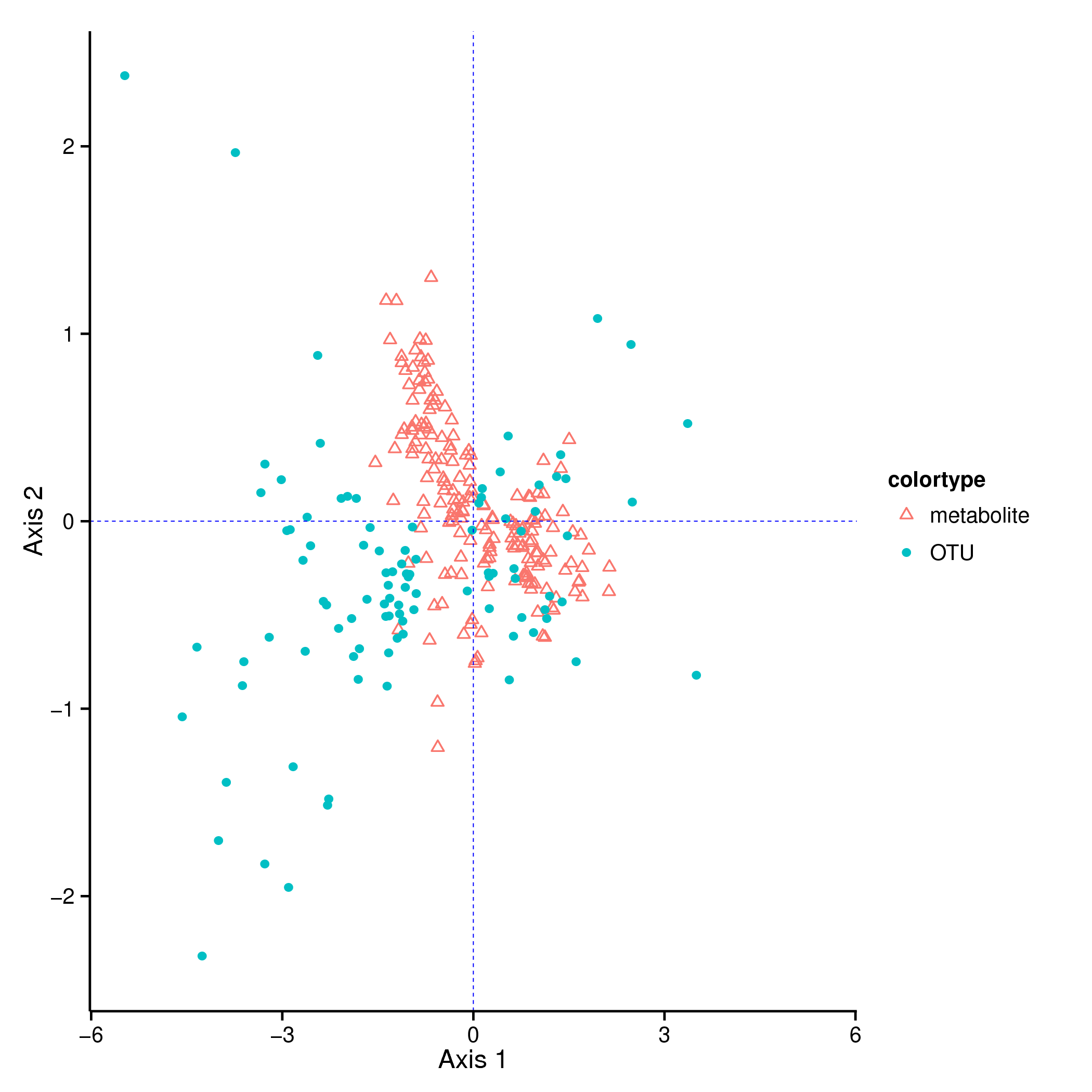

Supplement: Supplemental Information 12 [file peerj-10-14444-s012.zip › Web_Report/coinertia_analysis/taxonomy/treat1_H_L.vs.H_L/treat1_H_L.vs.H_L_coinertia_analysis.png]

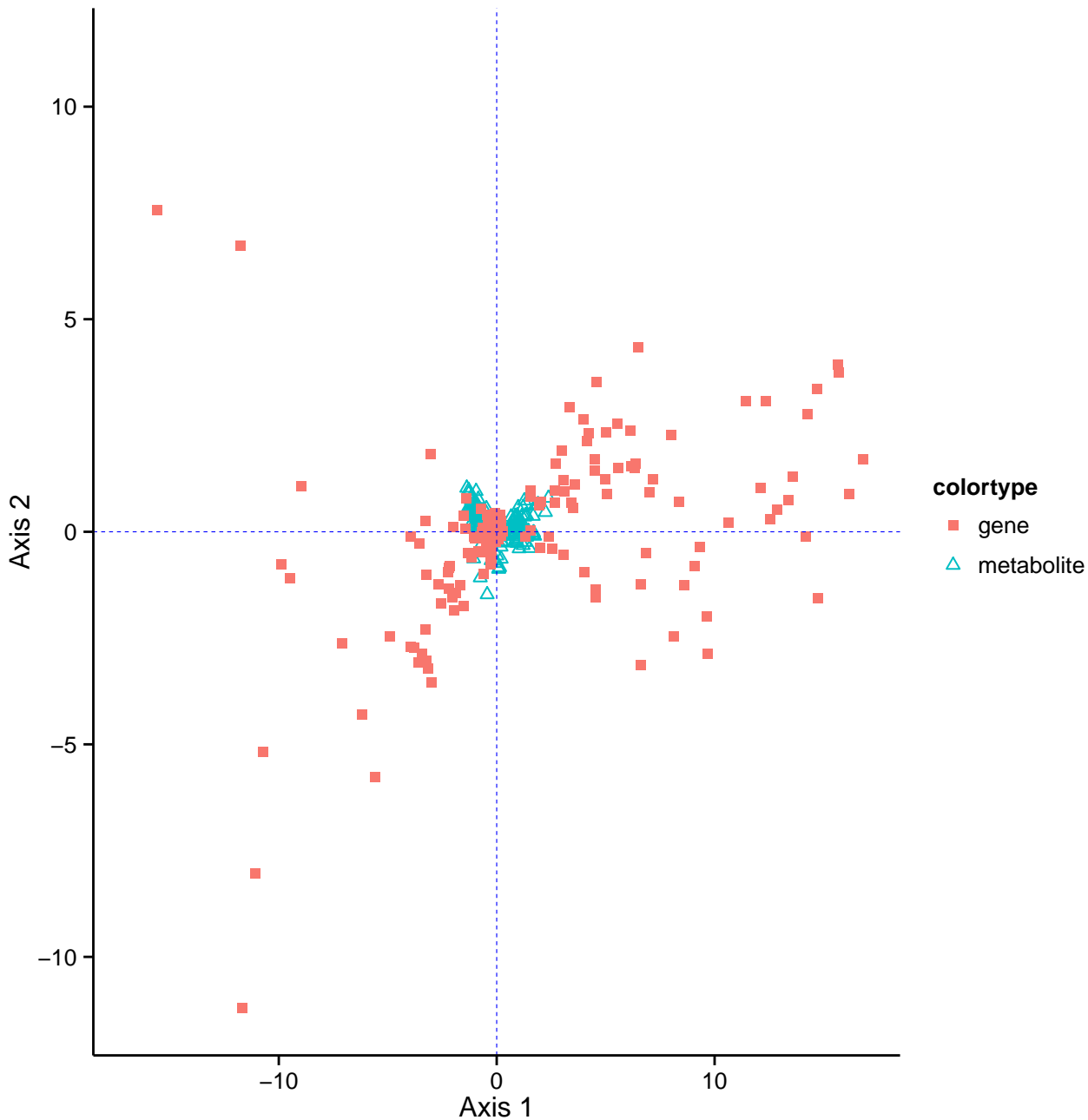

Supplement: Supplemental Information 12 [file peerj-10-14444-s012.zip › Web_Report/coinertia_analysis/function/treat1_H_L.vs.H_L/treat1_H_L.vs.H_L_coinertia_analysis.pdf]

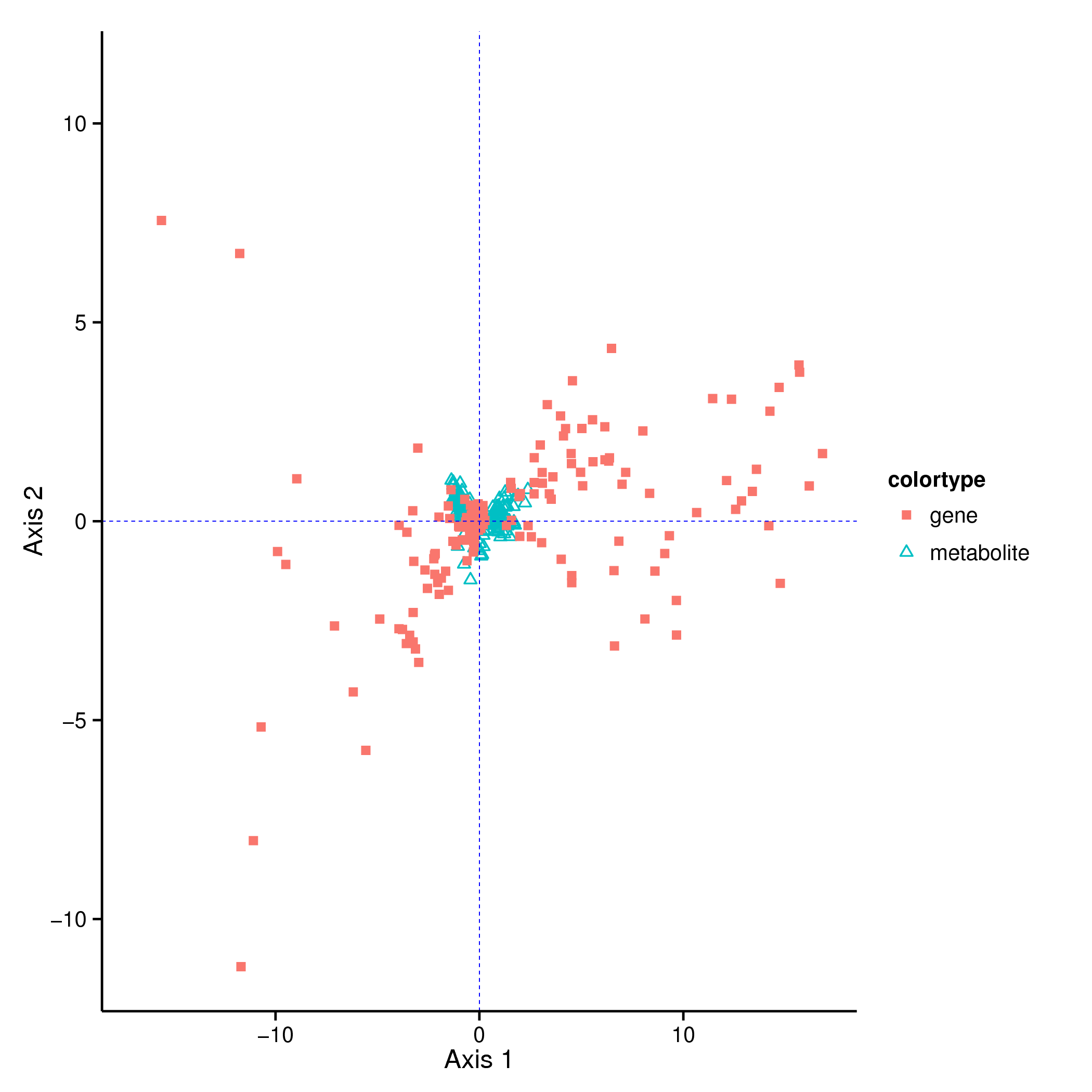

Supplement: Supplemental Information 12 [file peerj-10-14444-s012.zip › Web_Report/coinertia_analysis/function/treat1_H_L.vs.H_L/treat1_H_L.vs.H_L_coinertia_analysis.png]

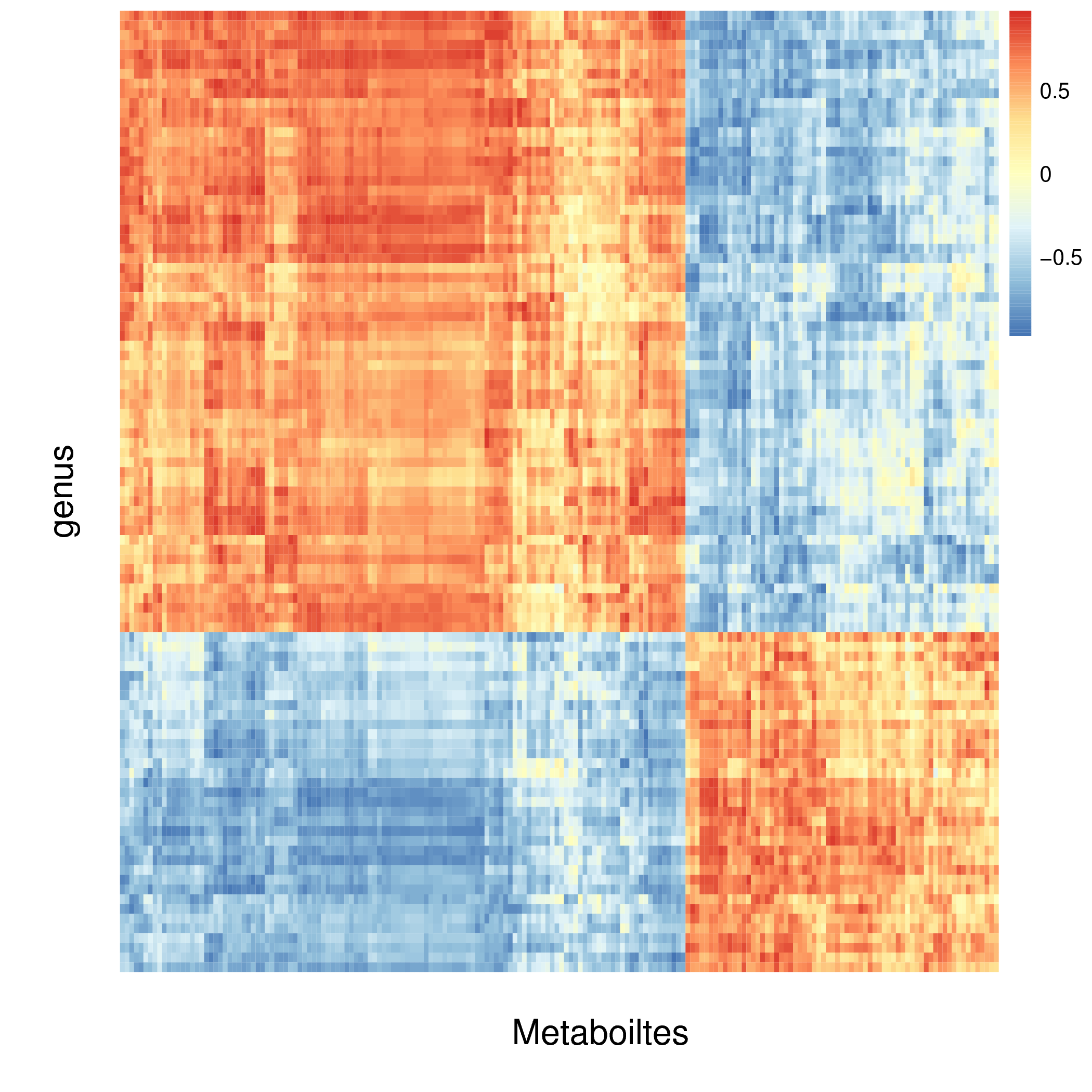

Supplement: Supplemental Information 12 [file peerj-10-14444-s012.zip › Web_Report/correlation_analysis/taxonomy/treat1_H_L.vs.H_L/treat1_H_L.vs.H_L_taxonomy_cor.png]

genus

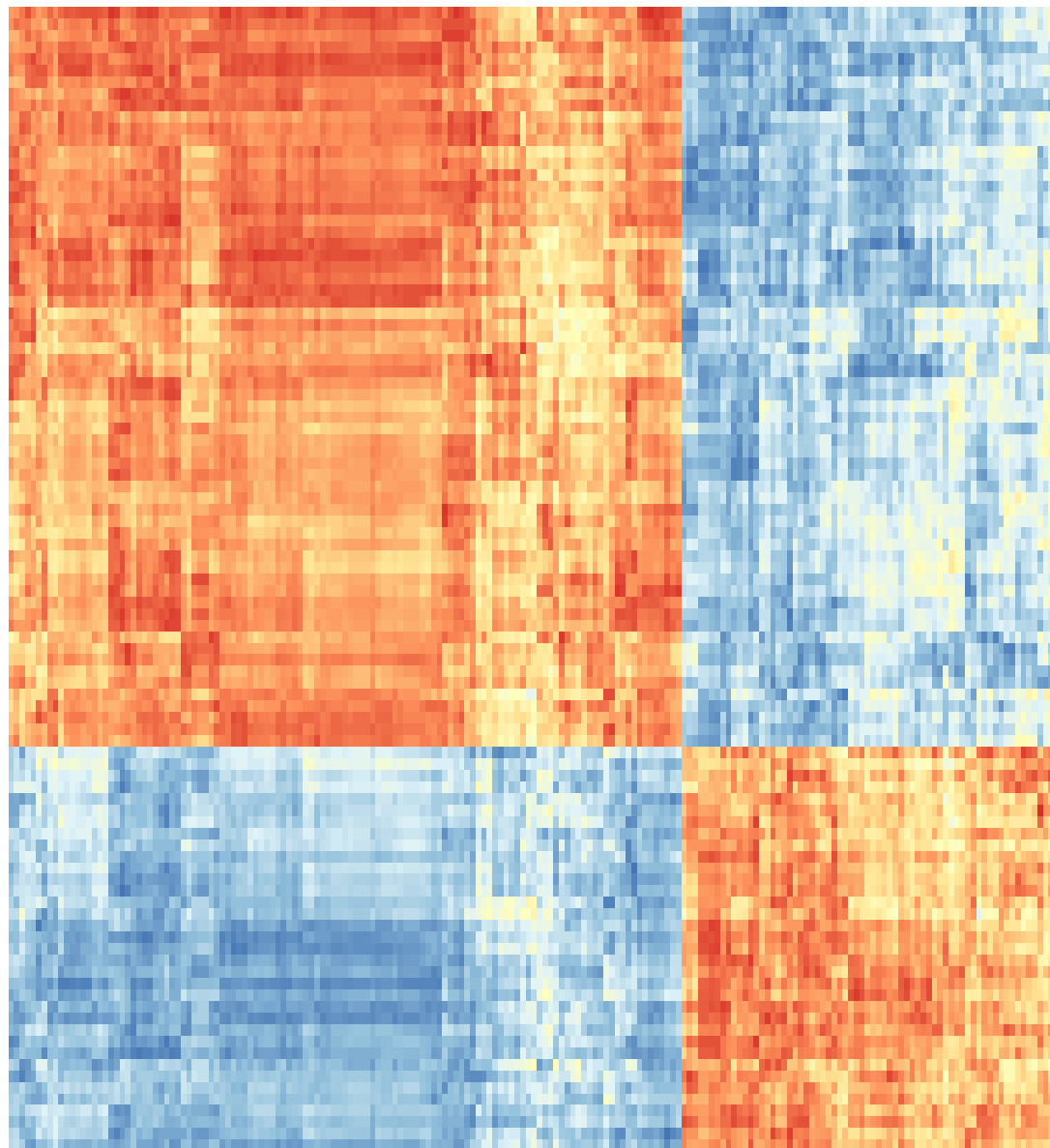

0.5

0

-0.5

Metaboiltes

Supplement: Supplemental Information 12 [file peerj-10-14444-s012.zip › Web_Report/correlation_analysis/taxonomy/treat1_H_L.vs.H_L/treat1_H_L.vs.H_L_taxonomy_cor.pdf]

Genes

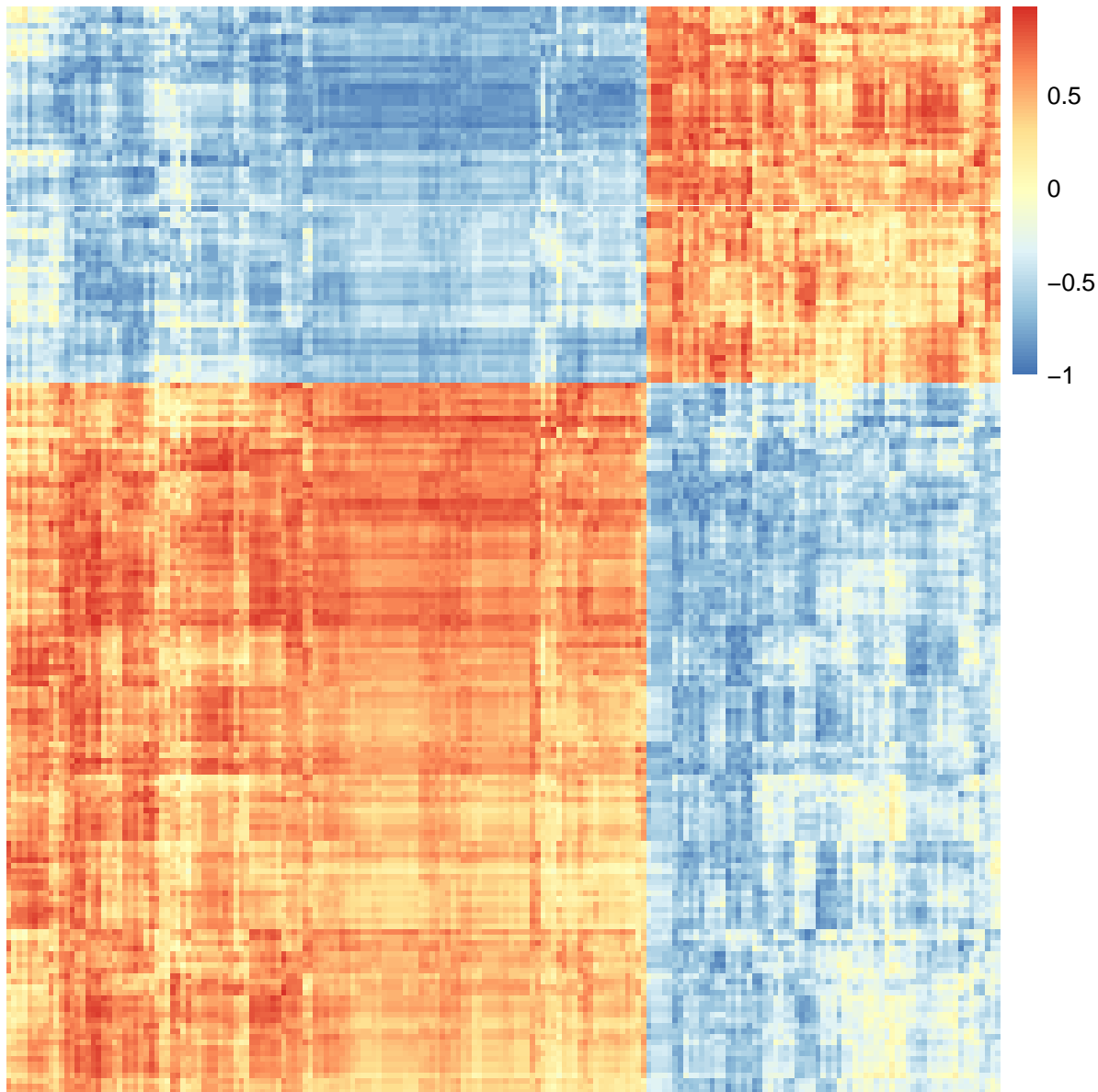

Metaboiltes

Supplement: Supplemental Information 12 [file peerj-10-14444-s012.zip › Web_Report/correlation_analysis/function/treat1_H_L.vs.H_L/treat1_H_L.vs.H_L_function_cor.pdf]

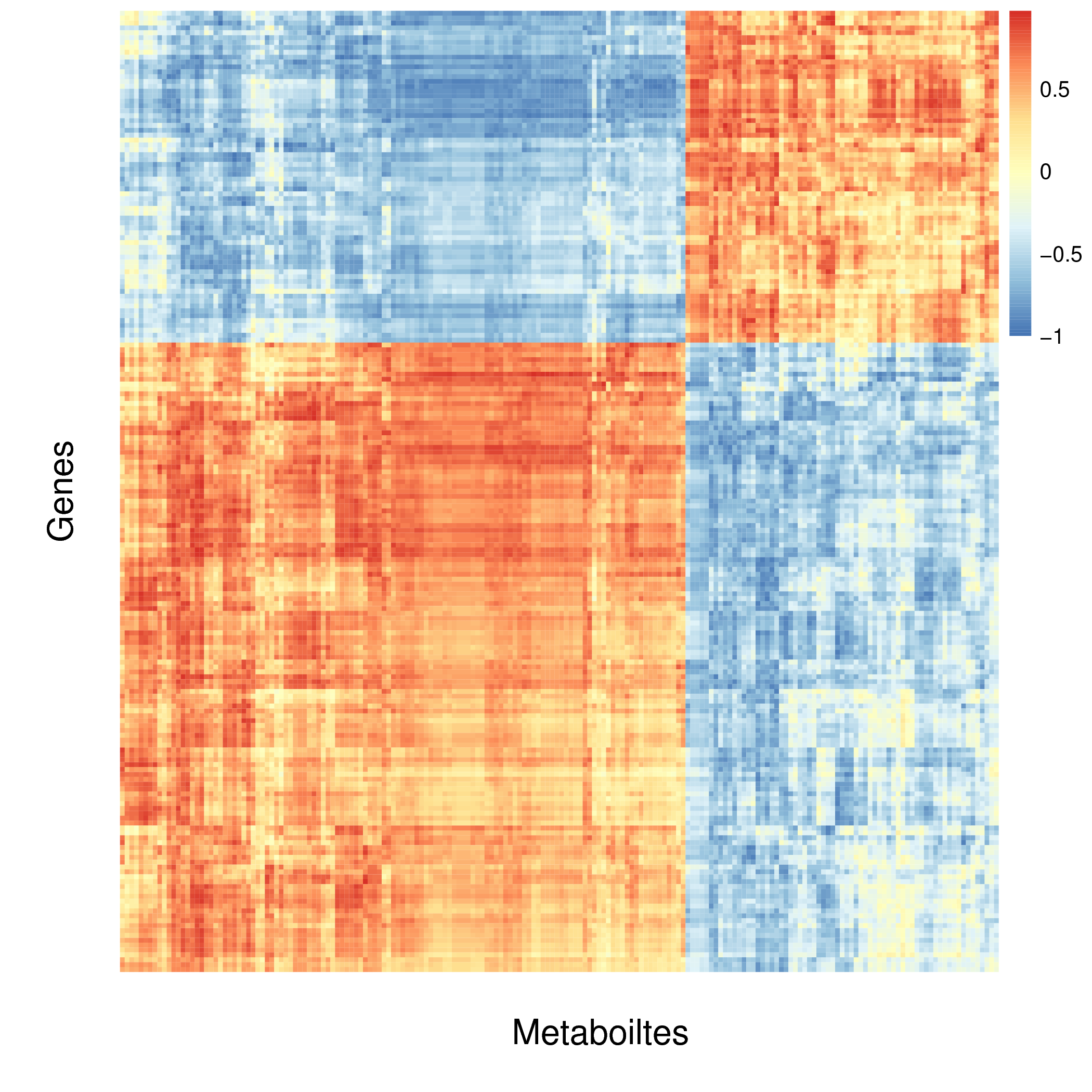

Supplement: Supplemental Information 12 [file peerj-10-14444-s012.zip › Web_Report/correlation_analysis/function/treat1_H_L.vs.H_L/treat1_H_L.vs.H_L_function_cor.png]

Supplement: Supplemental Information 12 [file peerj-10-14444-s012.zip › Web_Report/src/js/fancyBox/blank.gif]

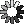

Supplement: Supplemental Information 12 [file peerj-10-14444-s012.zip › Web_Report/src/js/fancyBox/fancybox_loading.gif]

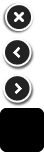

Supplement: Supplemental Information 12 [file peerj-10-14444-s012.zip › Web_Report/src/js/fancyBox/fancybox_sprite.png]

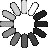

Supplement: Supplemental Information 12 [file peerj-10-14444-s012.zip › Web_Report/src/js/fancyBox/fancybox_loading@2x.gif]

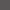

Supplement: Supplemental Information 12 [file peerj-10-14444-s012.zip › Web_Report/src/js/fancyBox/fancybox_overlay.png]

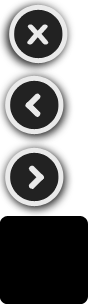

Supplement: Supplemental Information 12 [file peerj-10-14444-s012.zip › Web_Report/src/js/fancyBox/fancybox_sprite@2x.png]

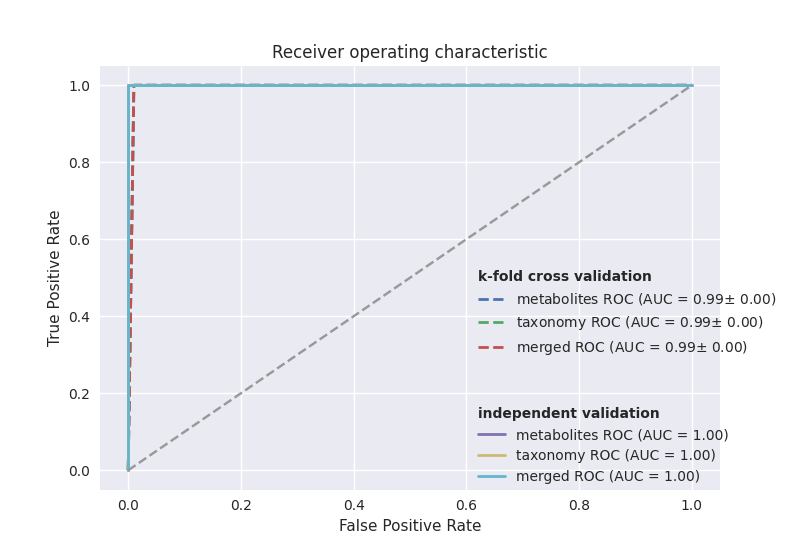

Supplement: Supplemental Information 12 [file peerj-10-14444-s012.zip › Web_Report/src/images/910a120b-00c3-4890-8f0c-16321ee16f4b.png]

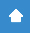

Supplement: Supplemental Information 12 [file peerj-10-14444-s012.zip › Web_Report/src/images/goTop.jpg]

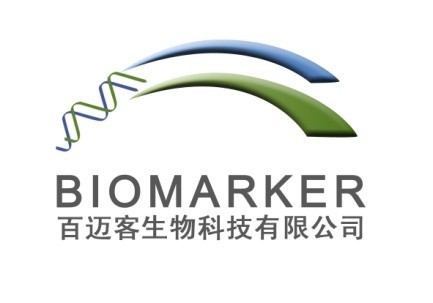

Supplement: Supplemental Information 12 [file peerj-10-14444-s012.zip › Web_Report/src/images/logo.jpg]

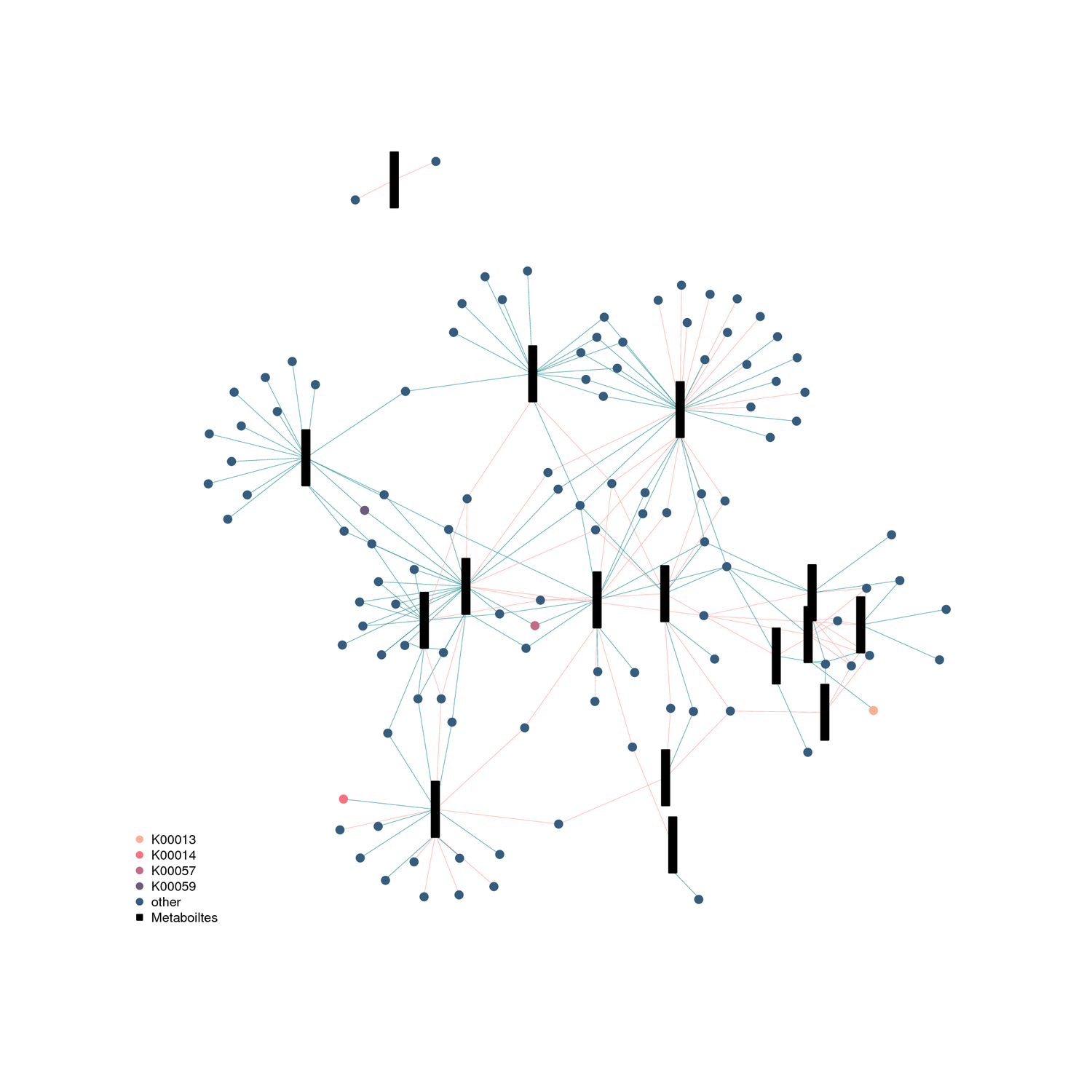

Supplement: Supplemental Information 12 [file peerj-10-14444-s012.zip › Web_Report/src/images/993b20a3-6b4d-4d4a-a714-01bec16c18a2.png]

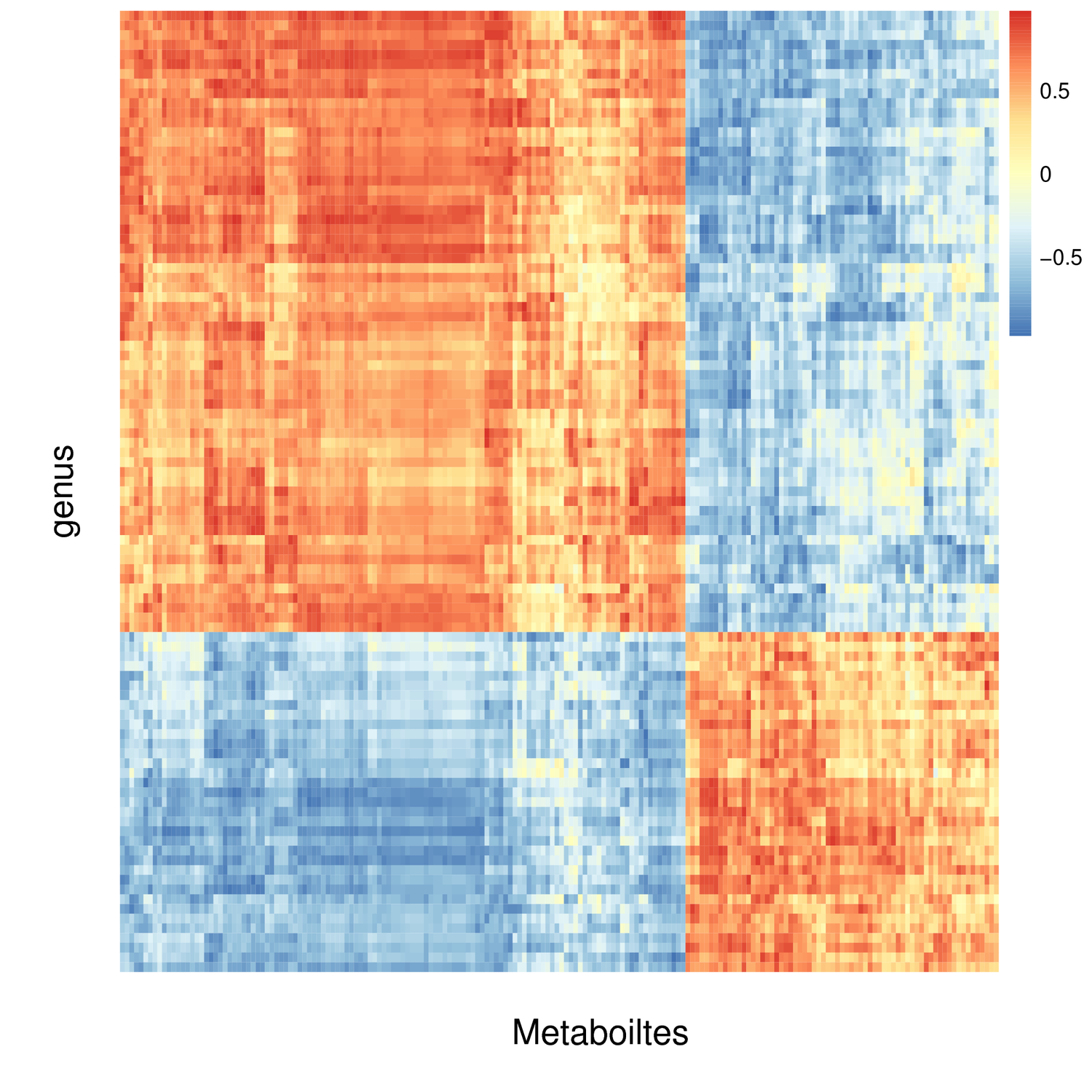

Supplement: Supplemental Information 12 [file peerj-10-14444-s012.zip › Web_Report/src/images/treat1_H_L.vs.H_L_taxonomy_cor.png]

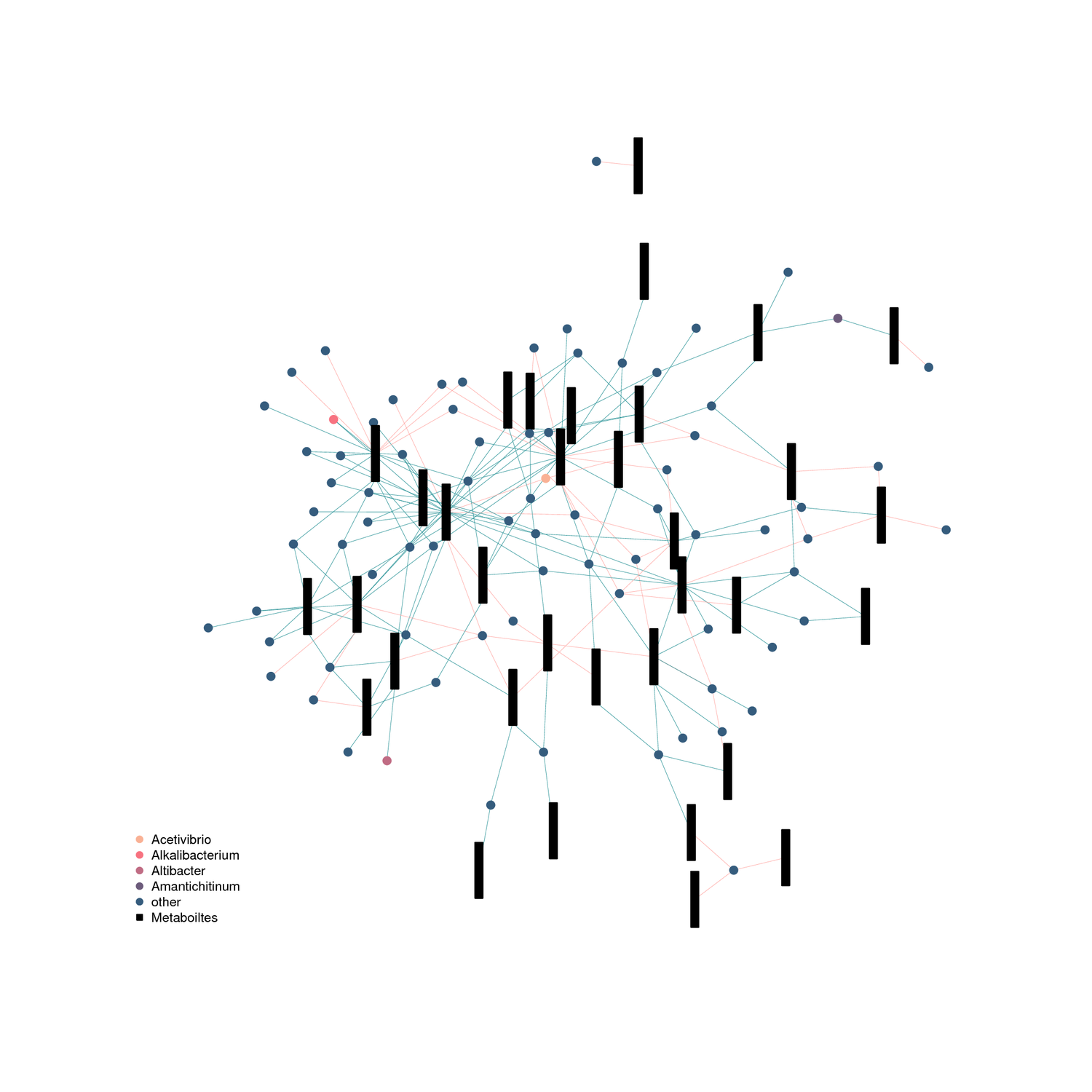

Supplement: Supplemental Information 12 [file peerj-10-14444-s012.zip › Web_Report/src/images/treat1_H_L.vs.H_L_module_2_network.png]

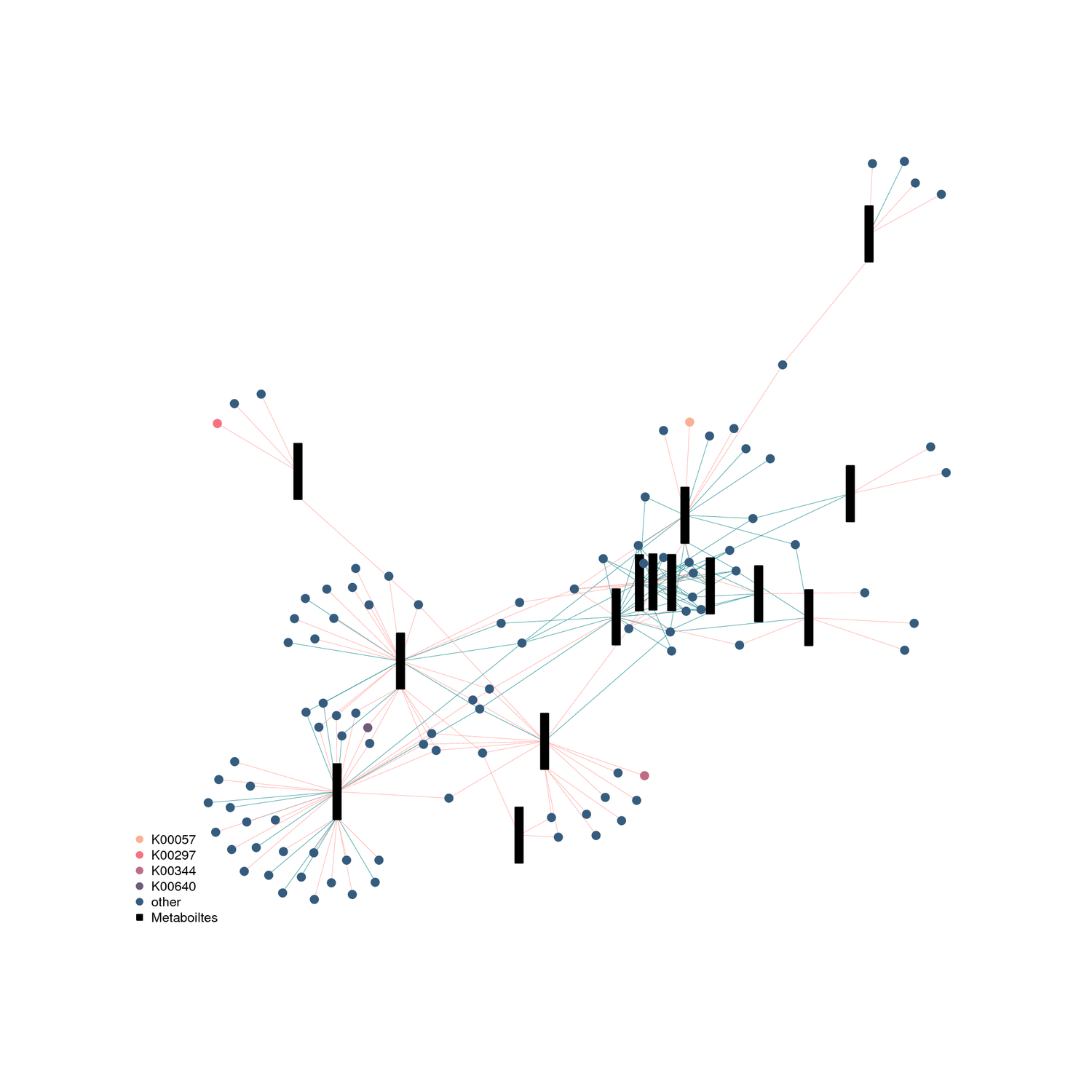

Supplement: Supplemental Information 12 [file peerj-10-14444-s012.zip › Web_Report/src/images/0a7946c5-e233-40e9-8611-7ab606ceacf2.png]

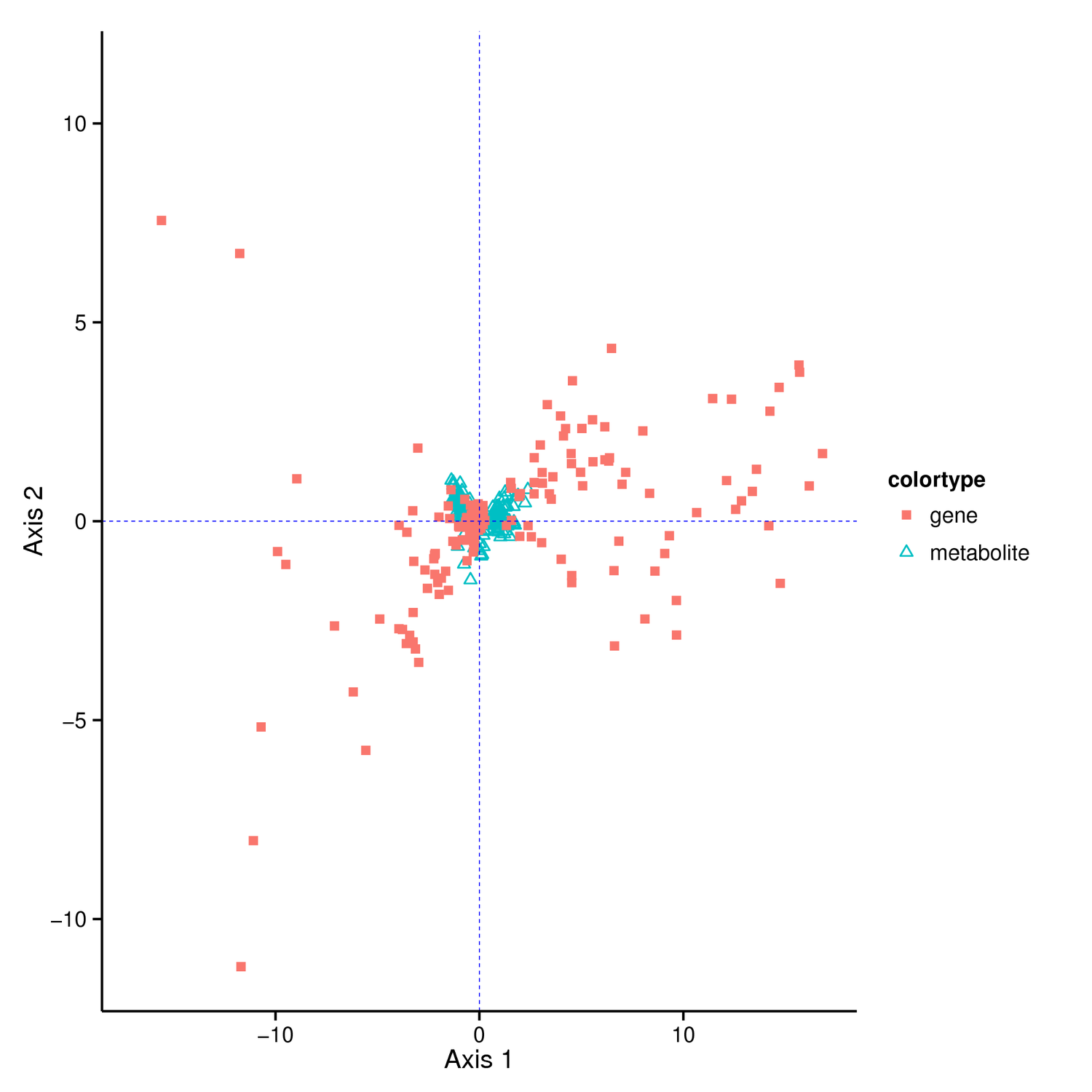

Supplement: Supplemental Information 12 [file peerj-10-14444-s012.zip › Web_Report/src/images/ec1d2901-623b-4f58-8a7c-2f36ebde8a97.png]

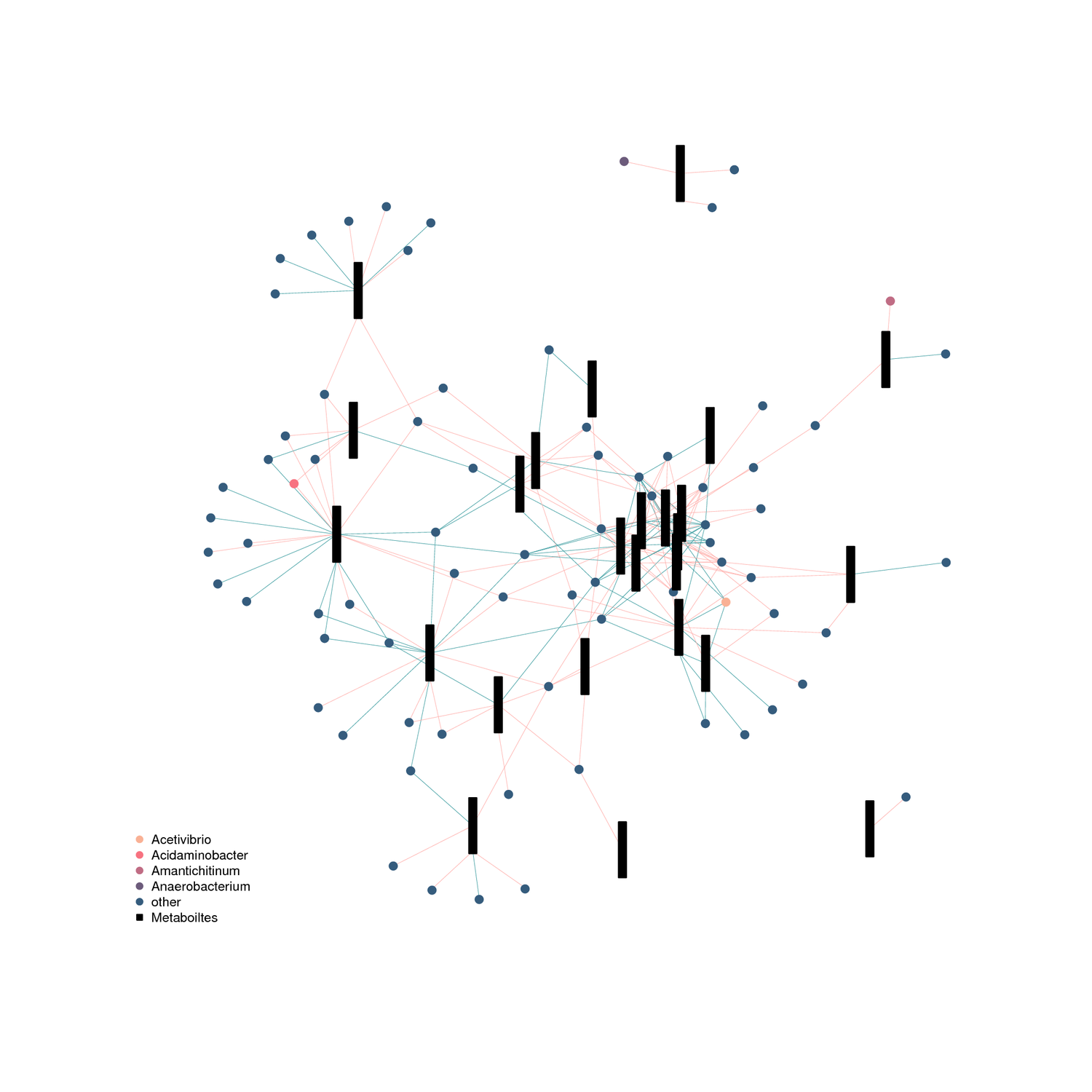

Supplement: Supplemental Information 12 [file peerj-10-14444-s012.zip › Web_Report/src/images/treat1_H_L.vs.H_L_module_1_network.png]

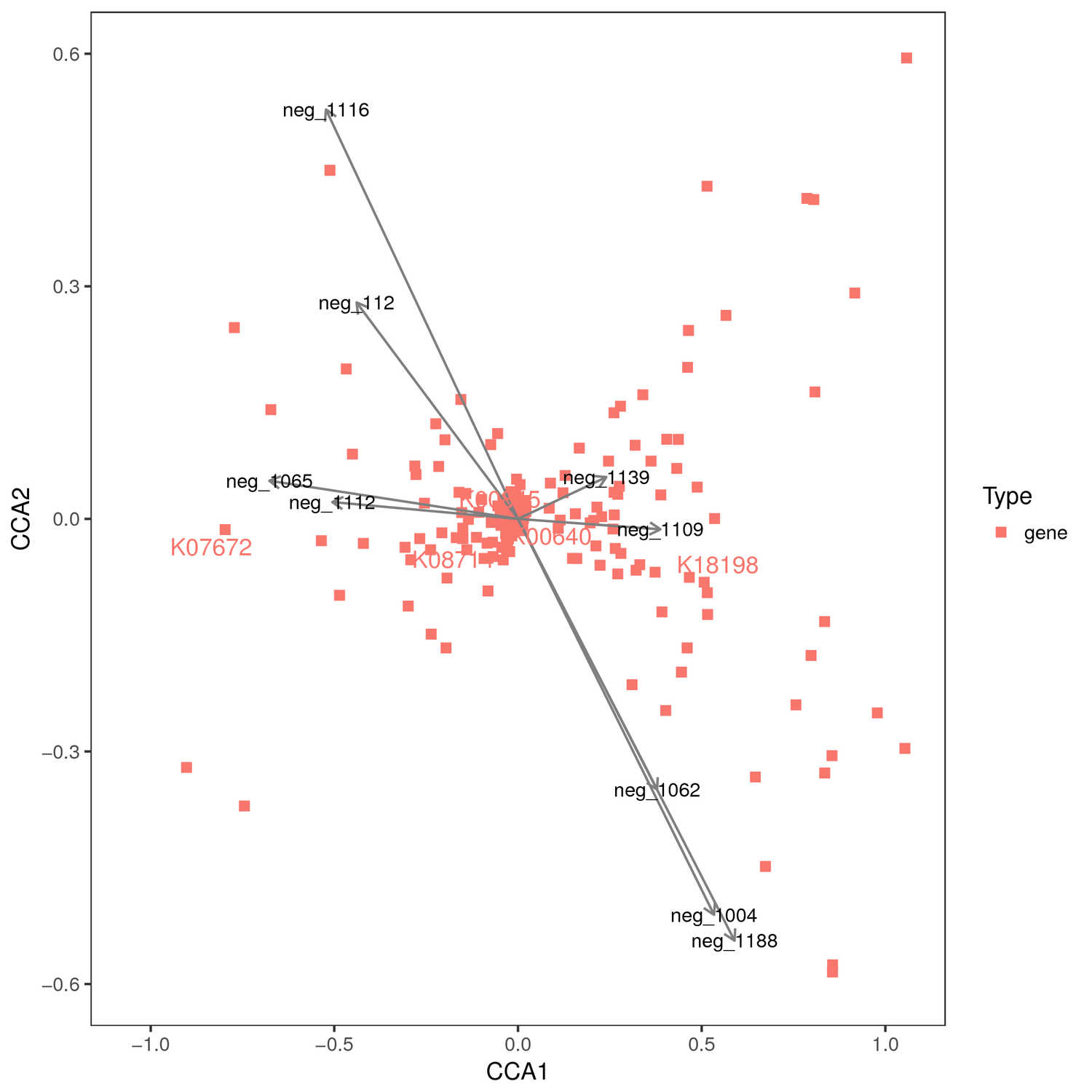

Supplement: Supplemental Information 12 [file peerj-10-14444-s012.zip › Web_Report/src/images/811eb401-d835-4f44-bb7b-70010271f803.png]

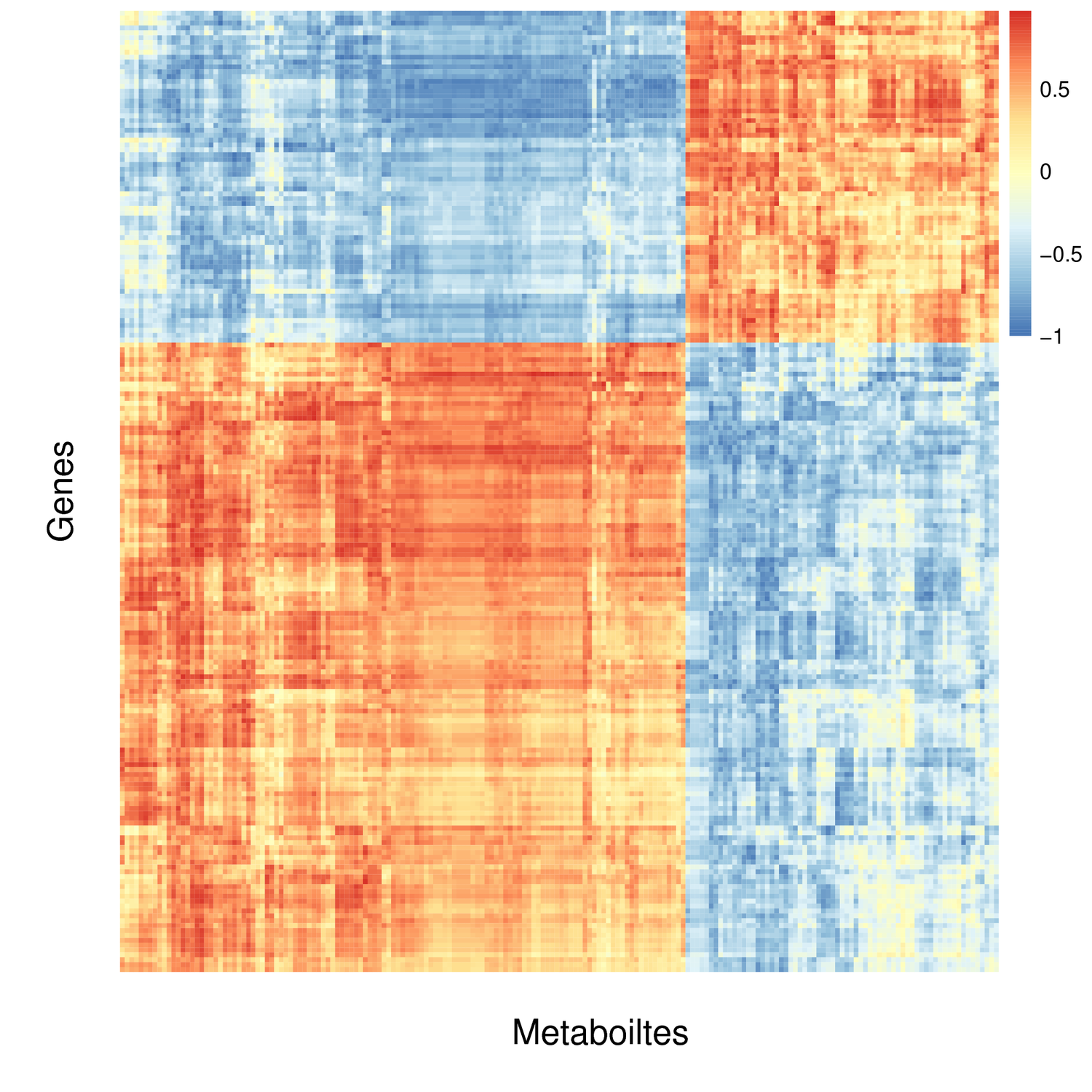

Supplement: Supplemental Information 12 [file peerj-10-14444-s012.zip › Web_Report/src/images/treat1_H_L.vs.H_L_function_cor.png]

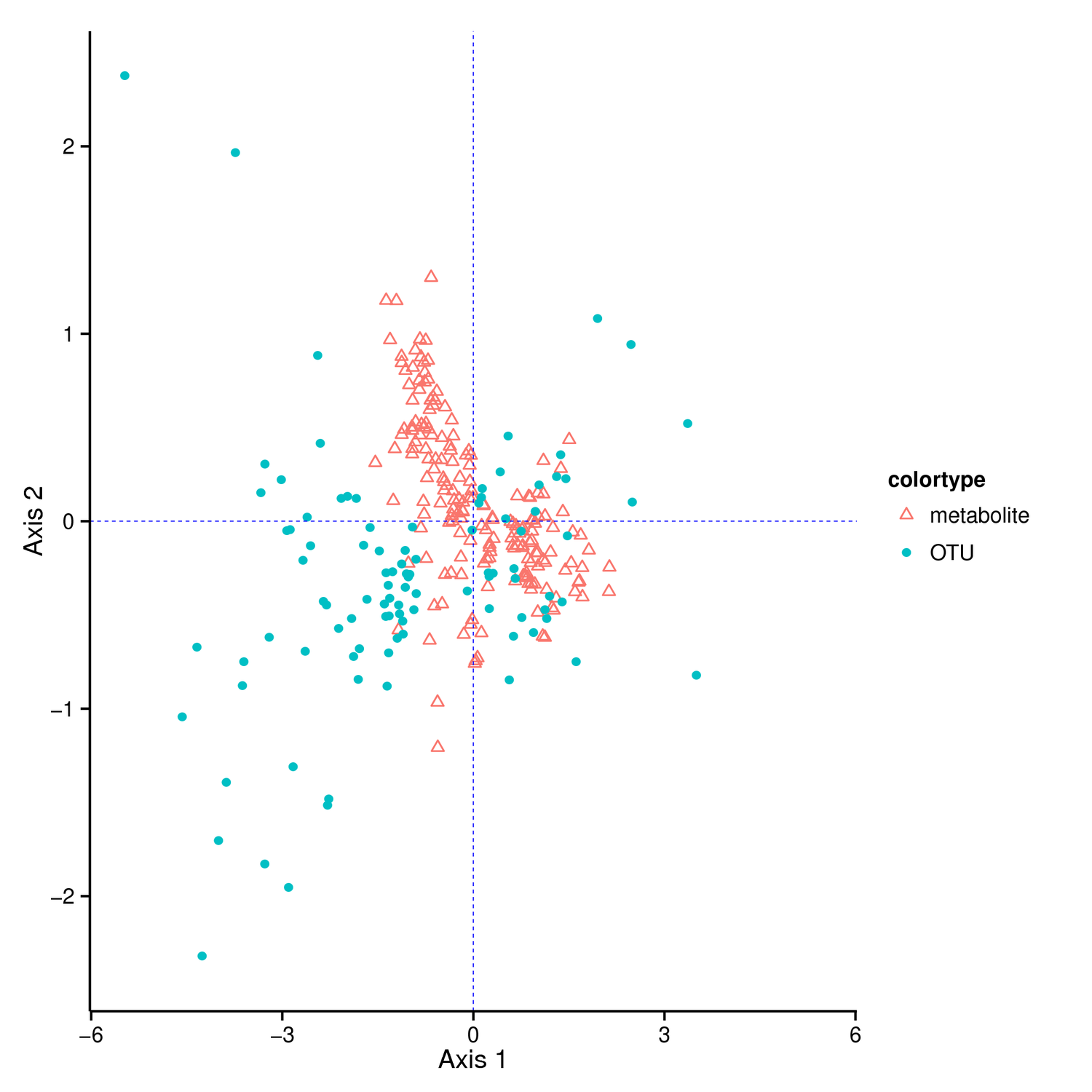

Supplement: Supplemental Information 12 [file peerj-10-14444-s012.zip › Web_Report/src/images/treat1_H_L.vs.H_L_coinertia_analysis.png]

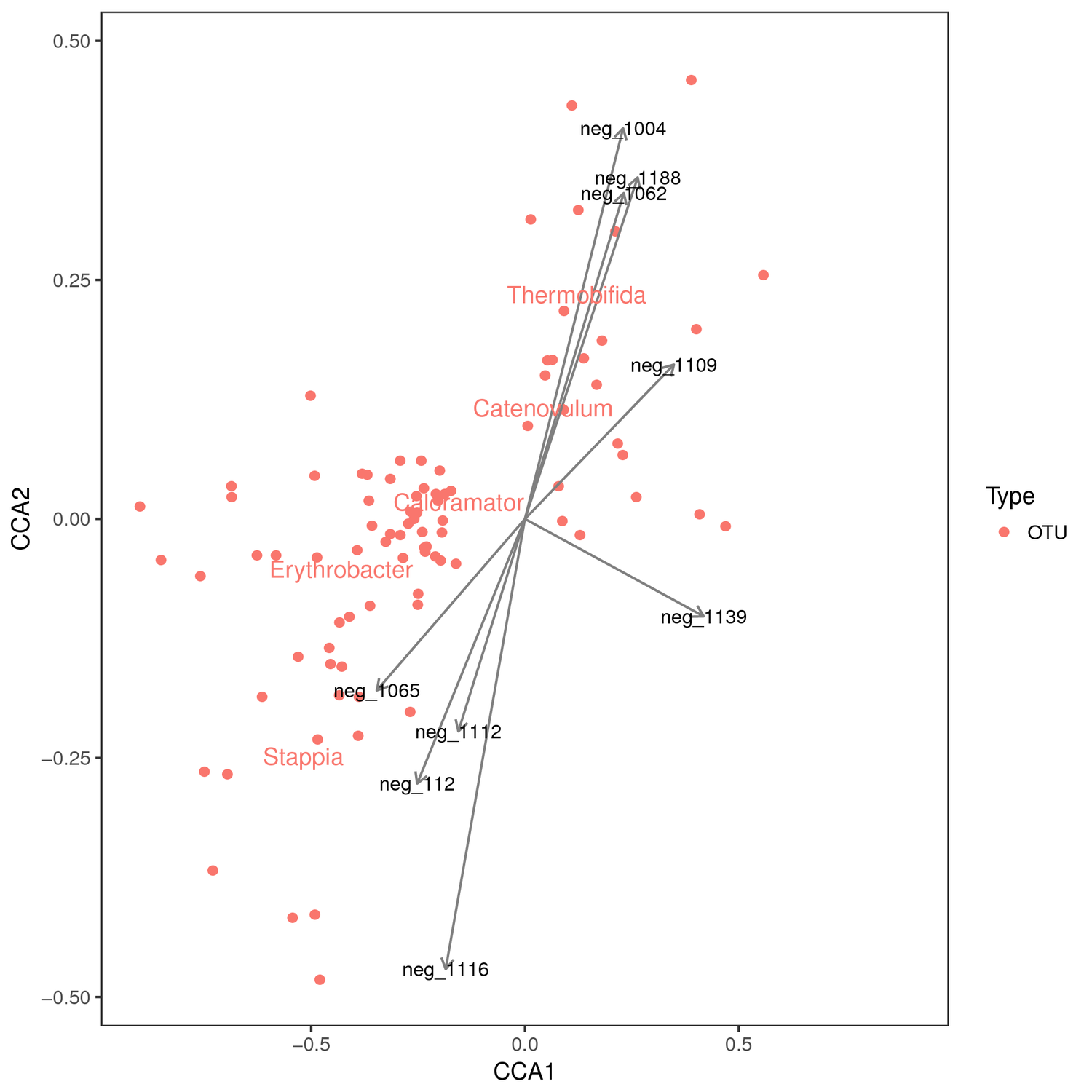

Supplement: Supplemental Information 12 [file peerj-10-14444-s012.zip › Web_Report/src/images/CCA.png]

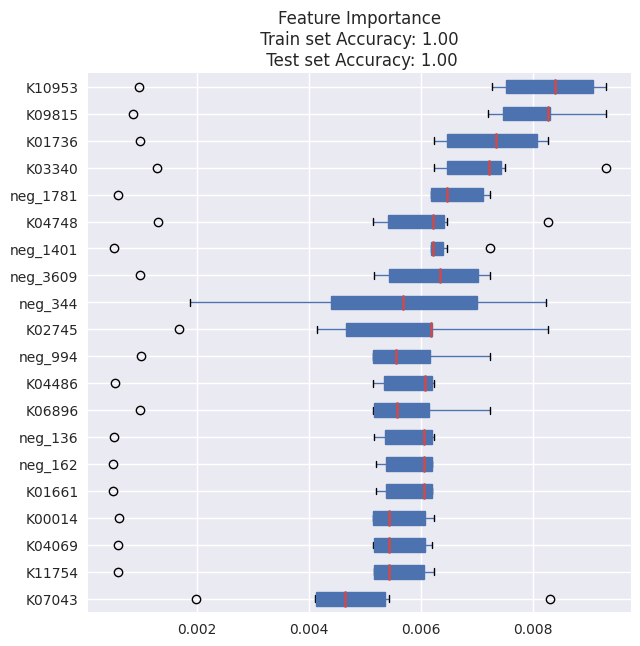

Supplement: Supplemental Information 12 [file peerj-10-14444-s012.zip › Web_Report/src/images/089d3e97-77fc-4a0c-813f-4bad0e141ab8.png]

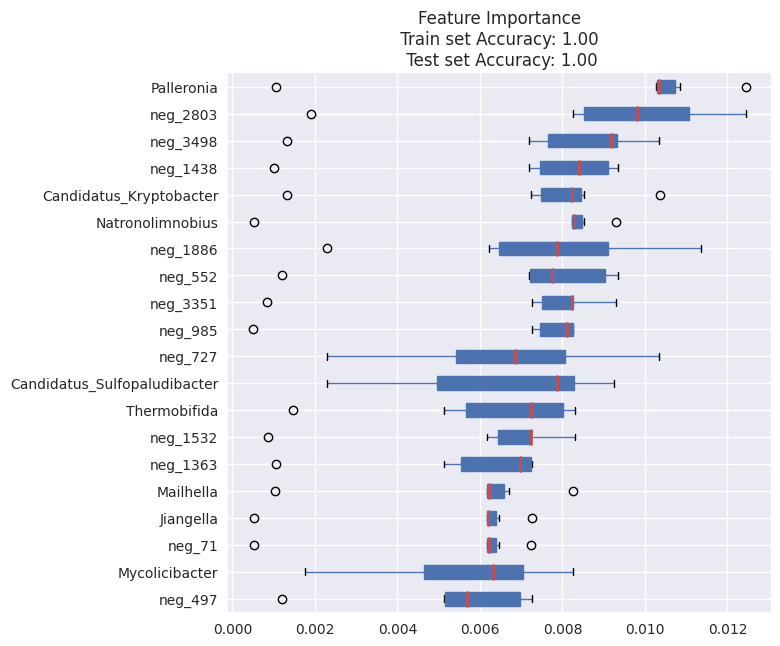

Supplement: Supplemental Information 12 [file peerj-10-14444-s012.zip › Web_Report/src/images/treat1_H_L.vs.H_L_feature_importance.png]

Receiver operating characteristic

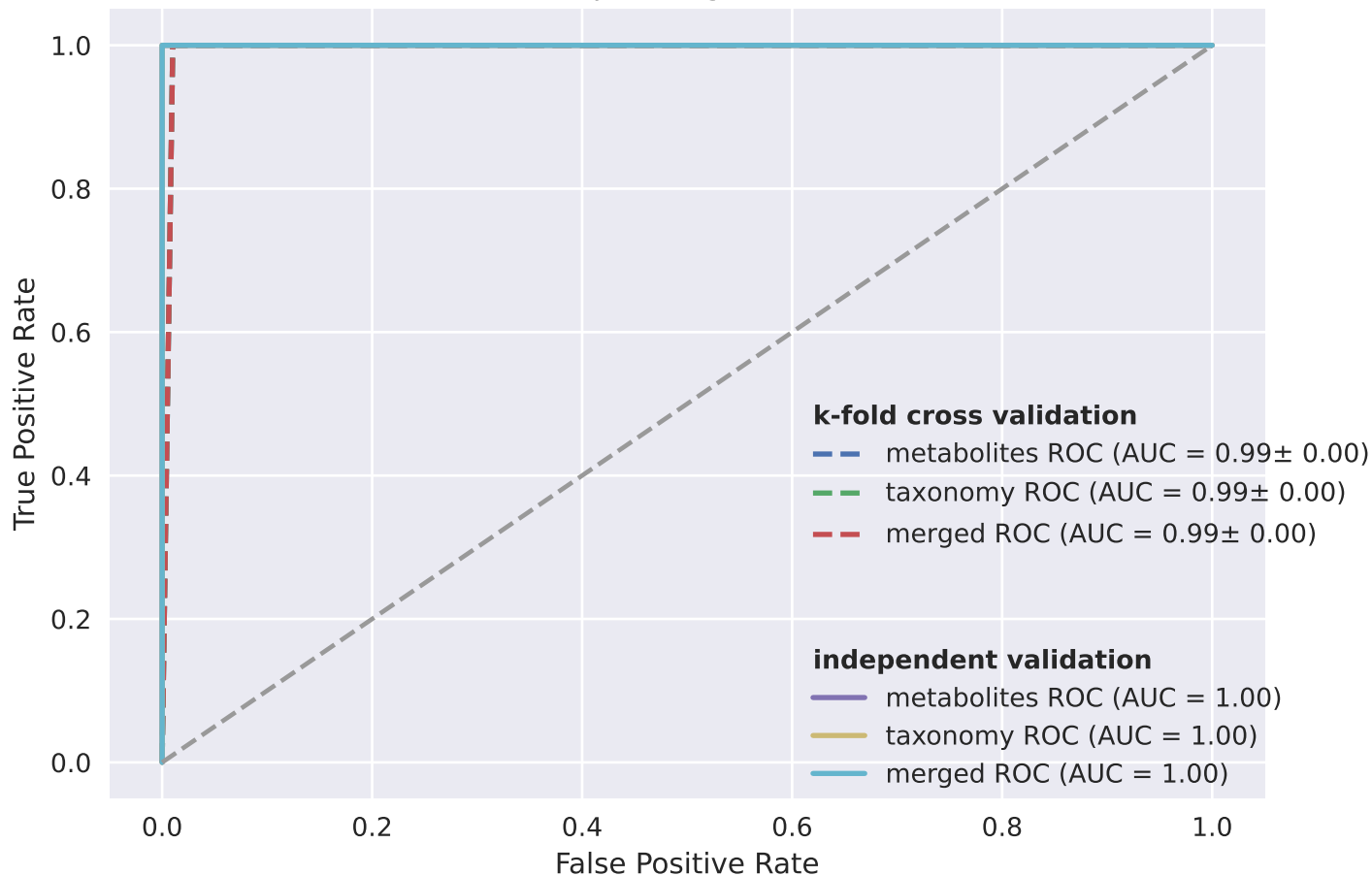

Supplement: Supplemental Information 12 [file peerj-10-14444-s012.zip › Web_Report/randomForest/taxonomy/treat1_H_L.vs.H_L/treat1_H_L.vs.H_L_ROC.pdf]

Feature Importance  
Train set Accuracy: 1.00  
Test set Accuracy: 1.00

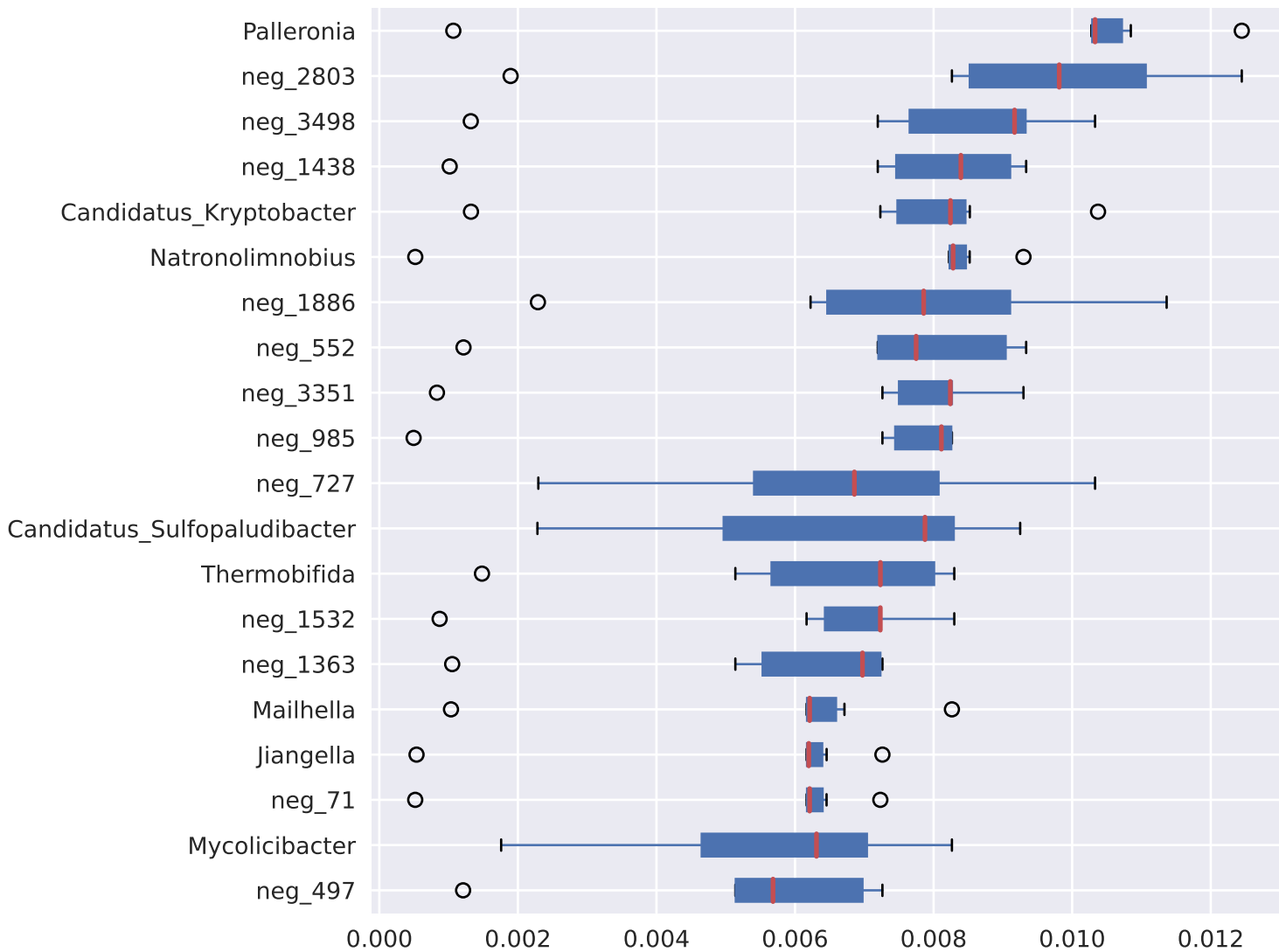

Supplement: Supplemental Information 12 [file peerj-10-14444-s012.zip › Web_Report/randomForest/taxonomy/treat1_H_L.vs.H_L/treat1_H_L.vs.H_L_feature_importance.pdf]

Receiver operating characteristic

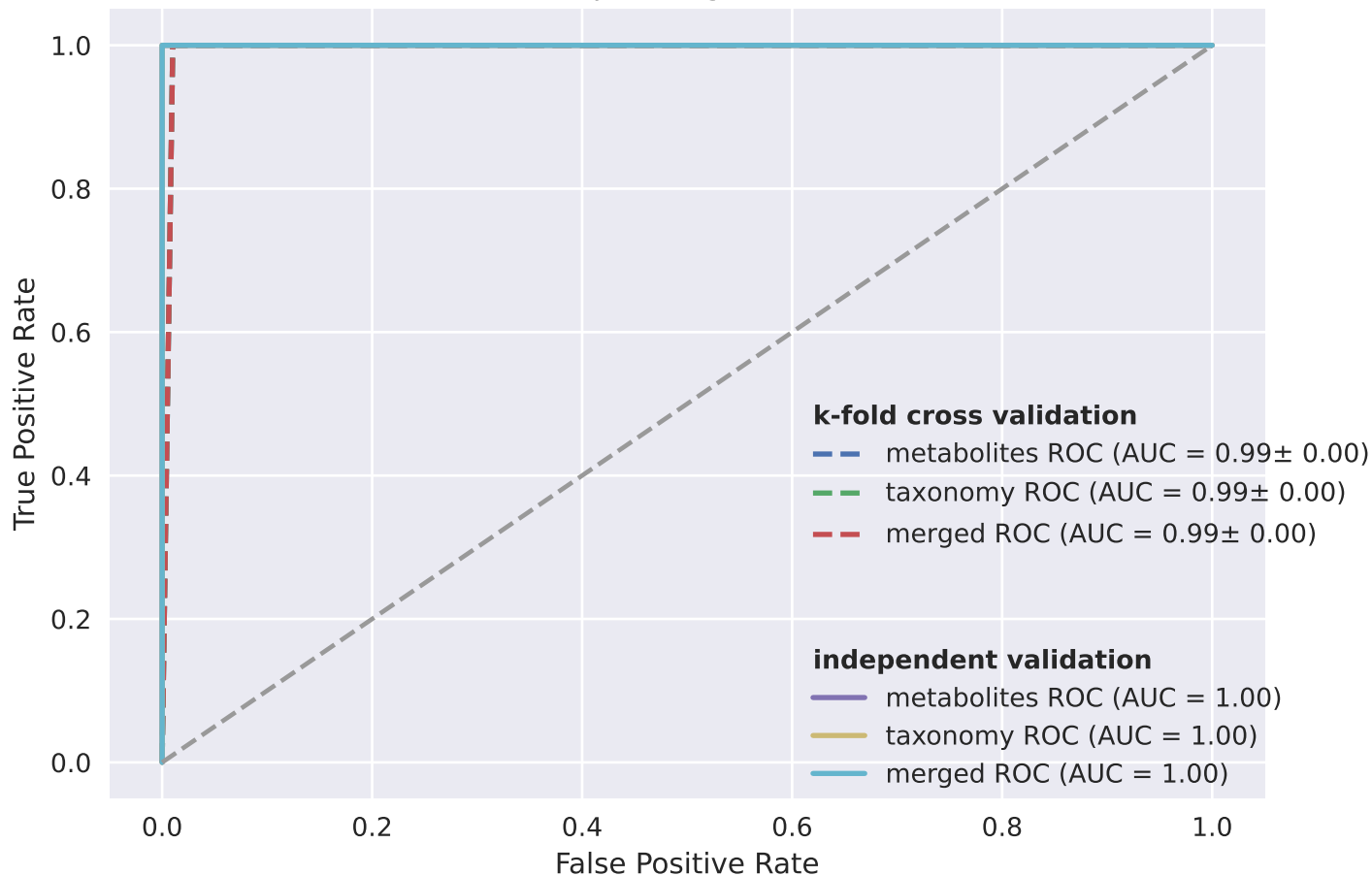

Supplement: Supplemental Information 12 [file peerj-10-14444-s012.zip › Web_Report/randomForest/function/treat1_H_L.vs.H_L/treat1_H_L.vs.H_L_ROC.pdf]

Feature Importance  
Train set Accuracy: 1.00  
Test set Accuracy: 1.00

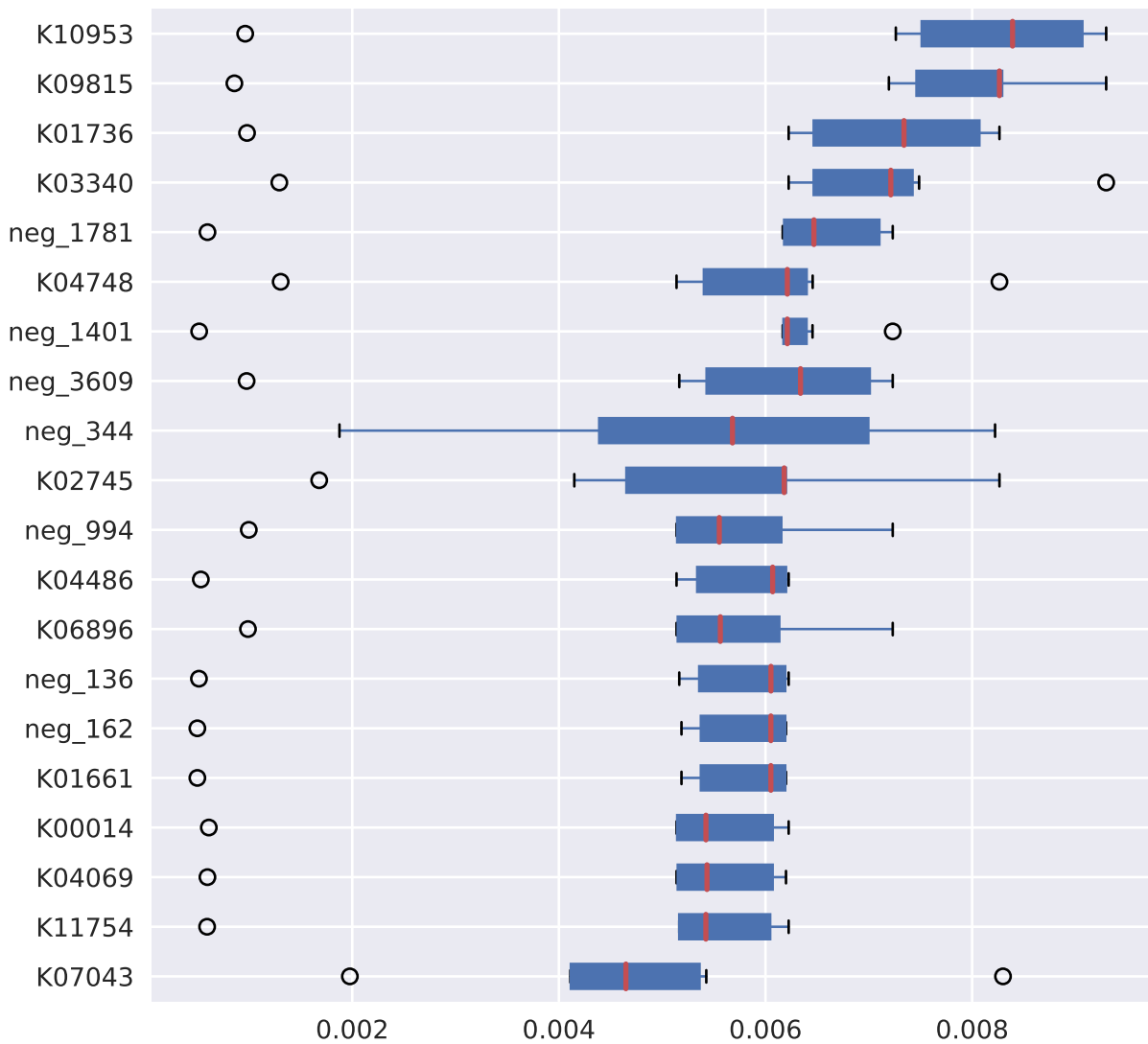

Supplement: Supplemental Information 12 [file peerj-10-14444-s012.zip › Web_Report/randomForest/function/treat1_H_L.vs.H_L/treat1_H_L.vs.H_L_feature_importance.pdf]

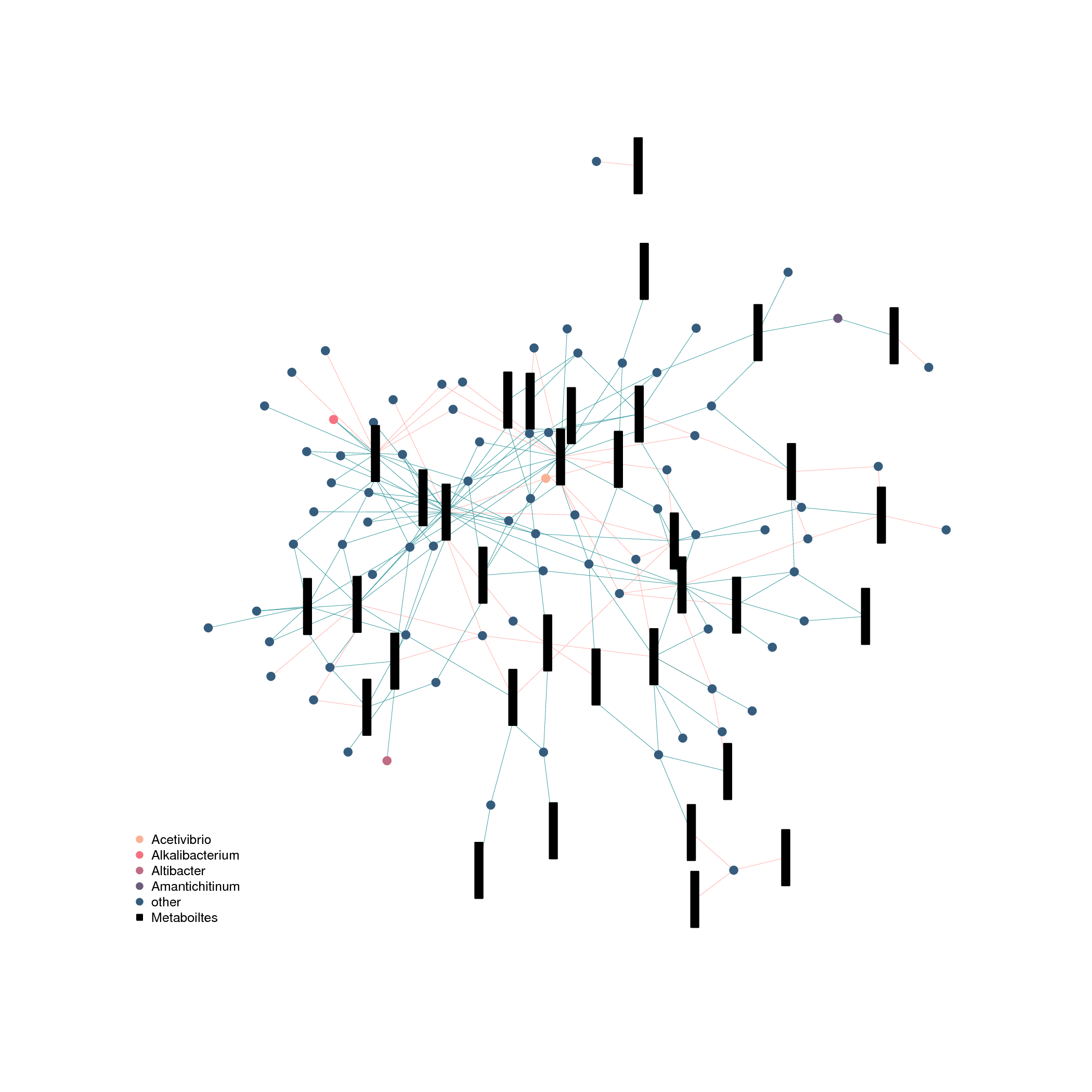

Supplement: Supplemental Information 12 [file peerj-10-14444-s012.zip › Web_Report/network_analysis/taxonomy/treat1_H_L.vs.H_L/treat1_H_L.vs.H_L_module_2_network.png]

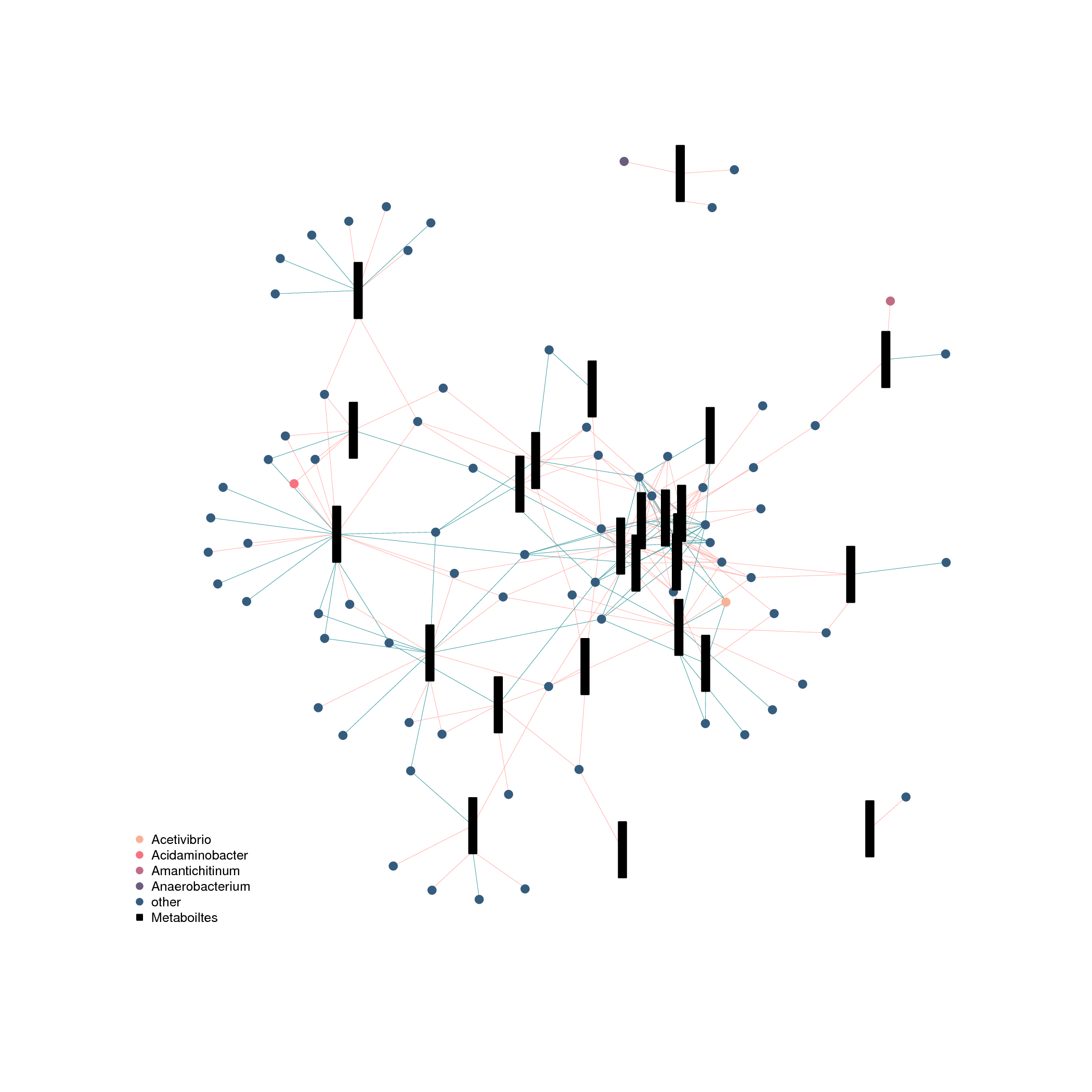

Supplement: Supplemental Information 12 [file peerj-10-14444-s012.zip › Web_Report/network_analysis/taxonomy/treat1_H_L.vs.H_L/treat1_H_L.vs.H_L_module_1_network.png]

- Acetivibrio
- Acidaminobacter
- Amantichitinum
- Anaerobacterium
- other
- Metabolites

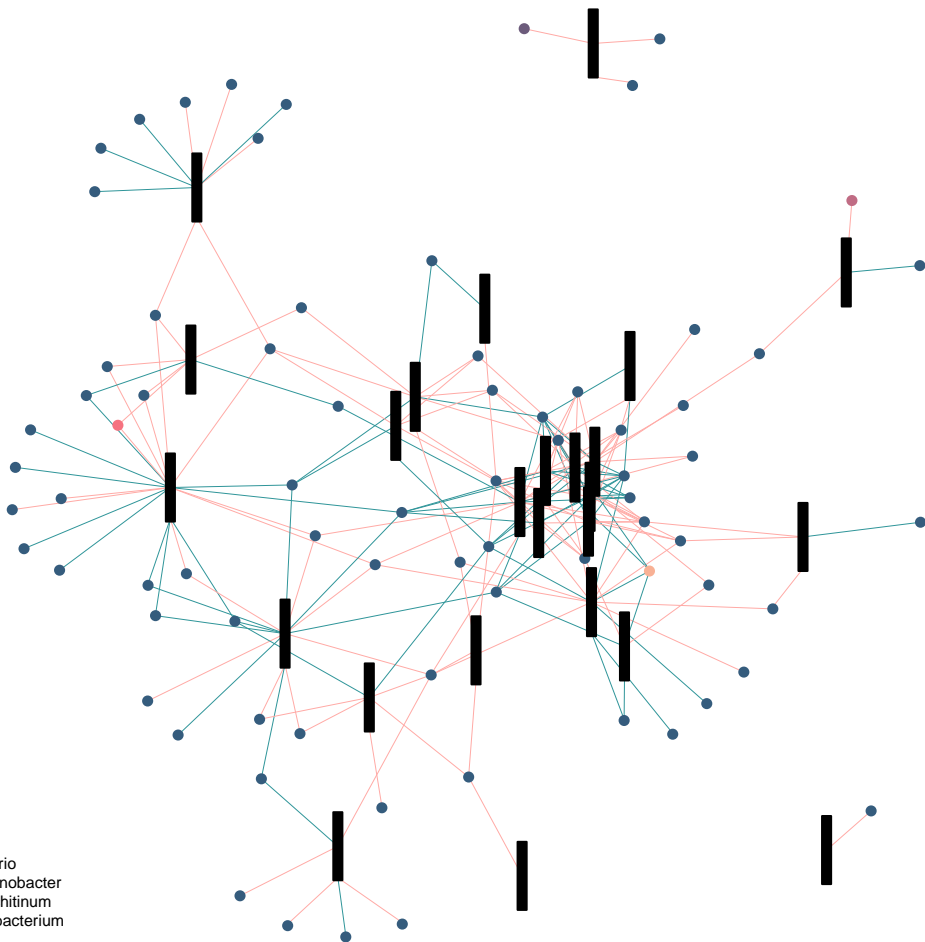

Supplement: Supplemental Information 12 [file peerj-10-14444-s012.zip › Web_Report/network_analysis/taxonomy/treat1_H_L.vs.H_L/treat1_H_L.vs.H_L_module_1_network.pdf]

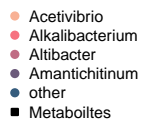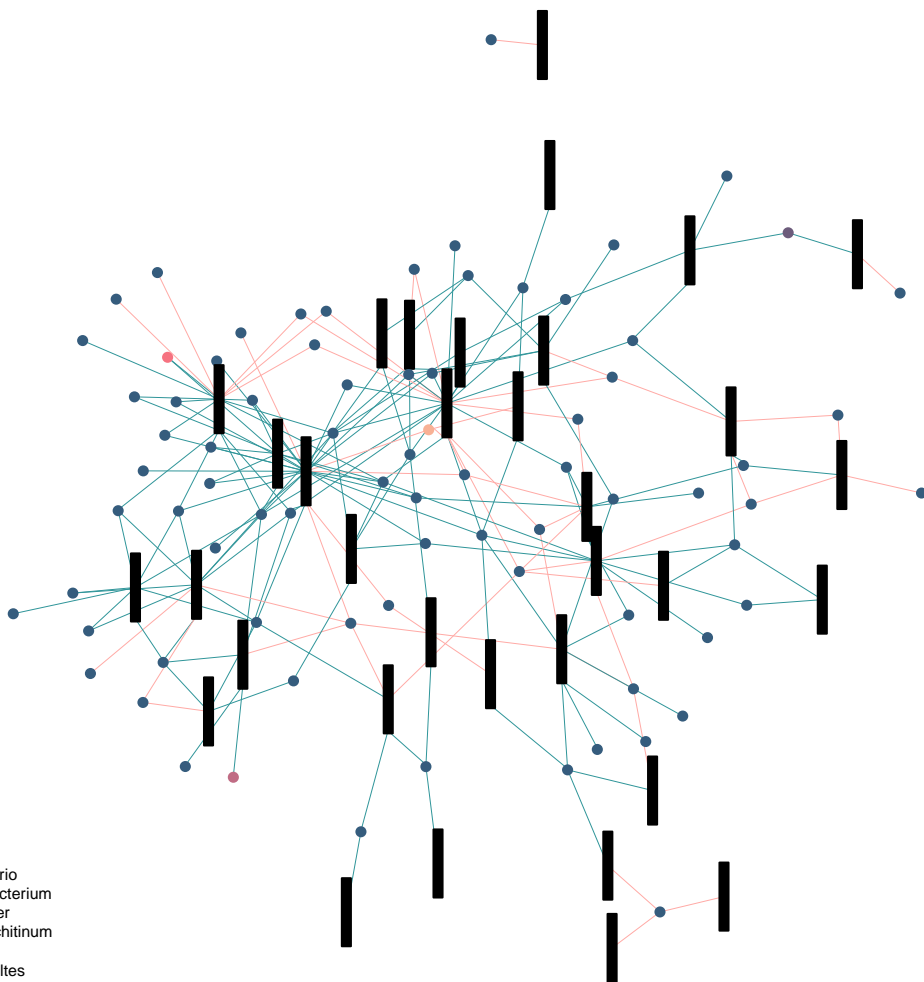

Supplement: Supplemental Information 12 [file peerj-10-14444-s012.zip › Web_Report/network_analysis/taxonomy/treat1_H_L.vs.H_L/treat1_H_L.vs.H_L_module_2_network.pdf]

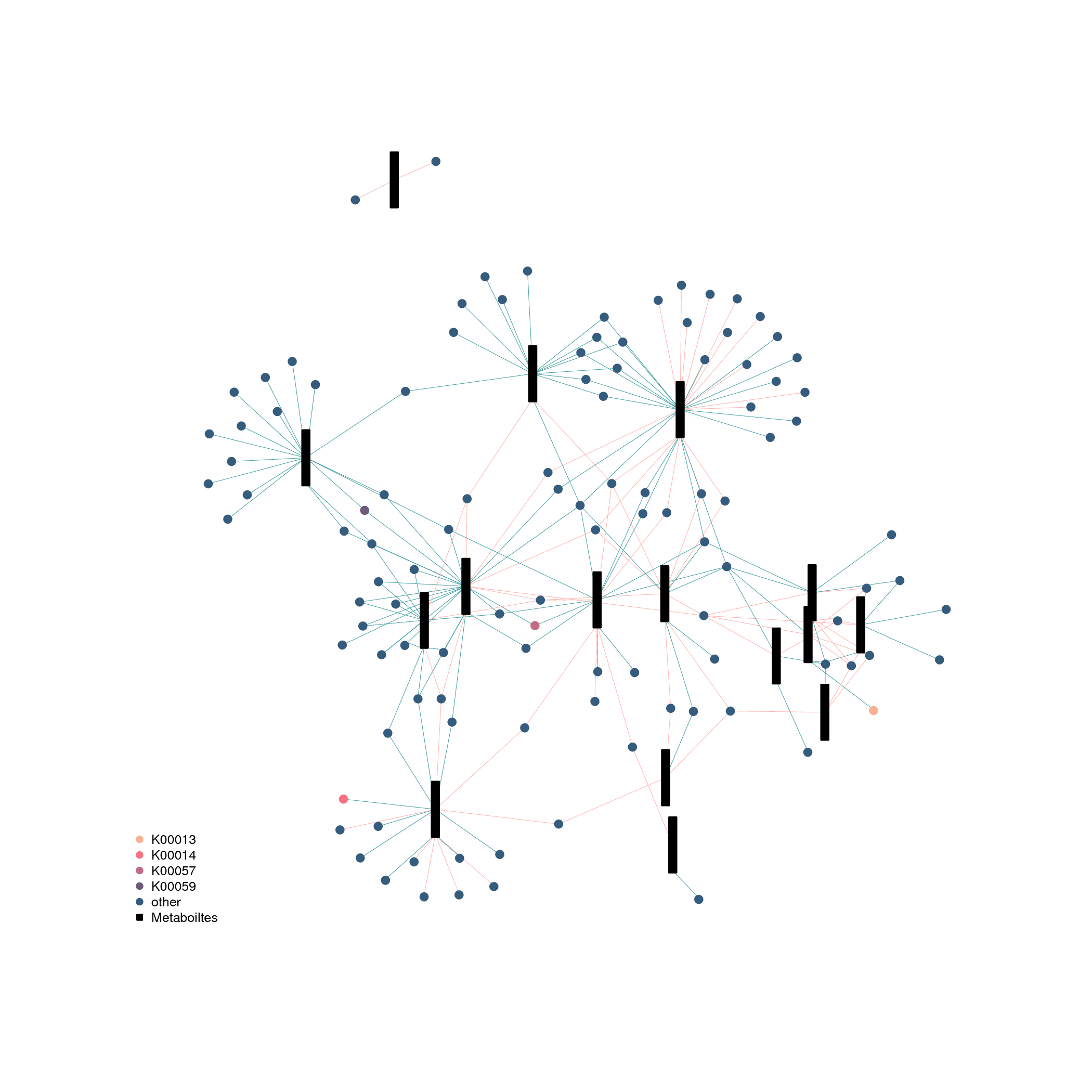

Supplement: Supplemental Information 12 [file peerj-10-14444-s012.zip › Web_Report/network_analysis/function/treat1_H_L.vs.H_L/treat1_H_L.vs.H_L_module_2_network.png]

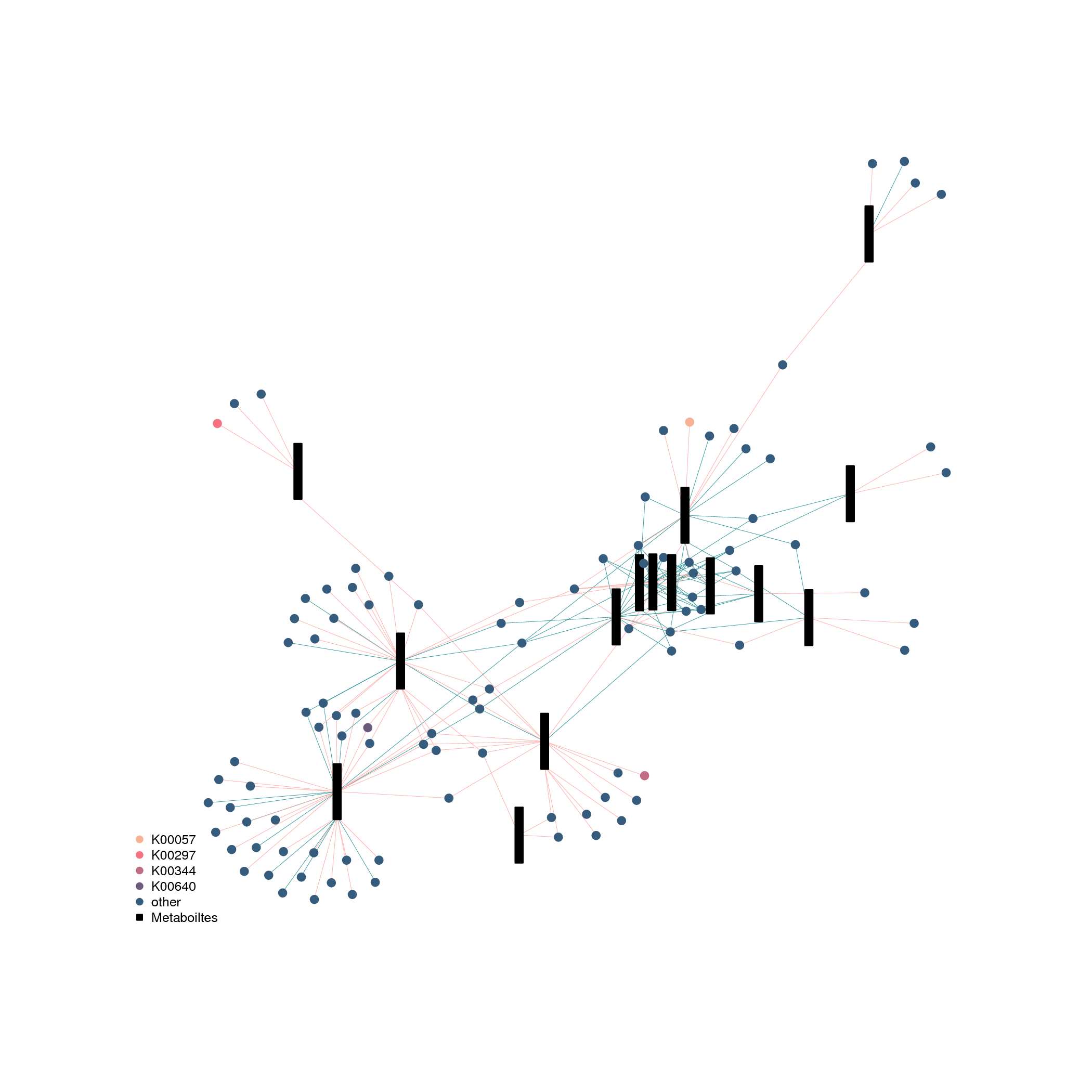

Supplement: Supplemental Information 12 [file peerj-10-14444-s012.zip › Web_Report/network_analysis/function/treat1_H_L.vs.H_L/treat1_H_L.vs.H_L_module_1_network.png]

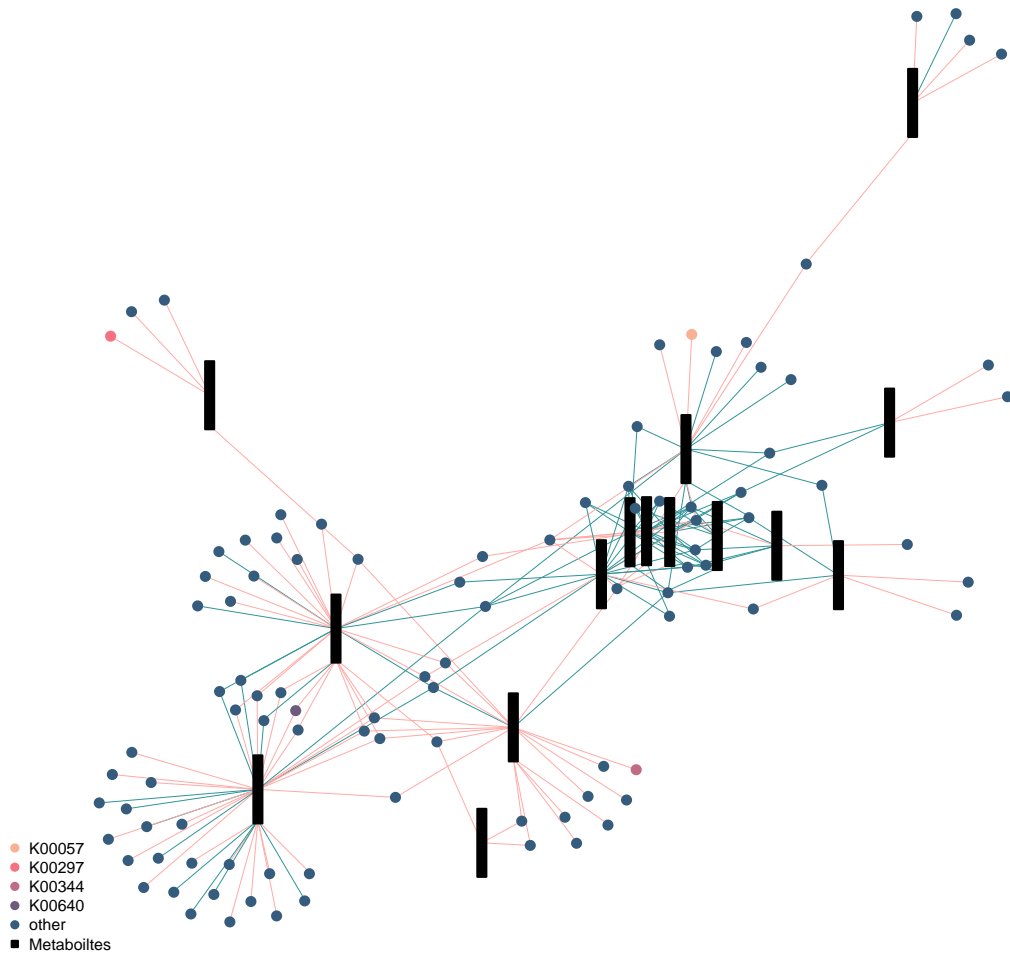

Supplement: Supplemental Information 12 [file peerj-10-14444-s012.zip › Web_Report/network_analysis/function/treat1_H_L.vs.H_L/treat1_H_L.vs.H_L_module_1_network.pdf]

● K00013  
● K00014  
● K00057  
● K00059  
● other  
■ Métabolites

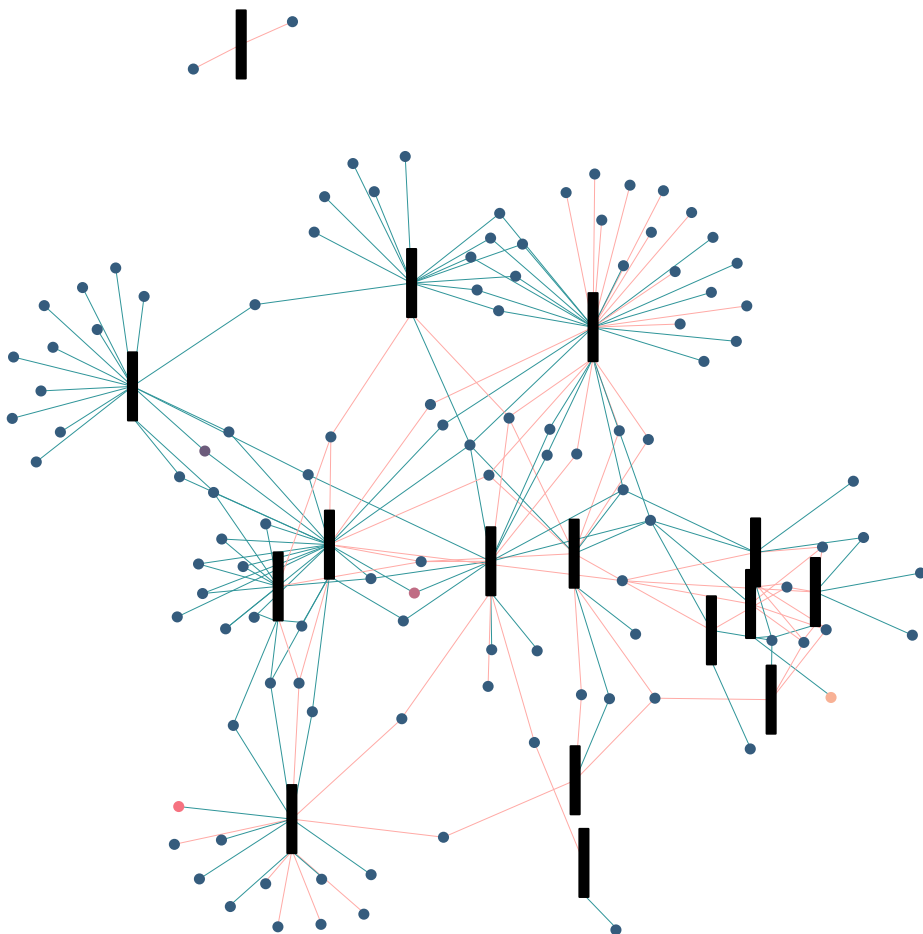

Supplement: Supplemental Information 12 [file peerj-10-14444-s012.zip › Web_Report/network_analysis/function/treat1_H_L.vs.H_L/treat1_H_L.vs.H_L_module_2_network.pdf]

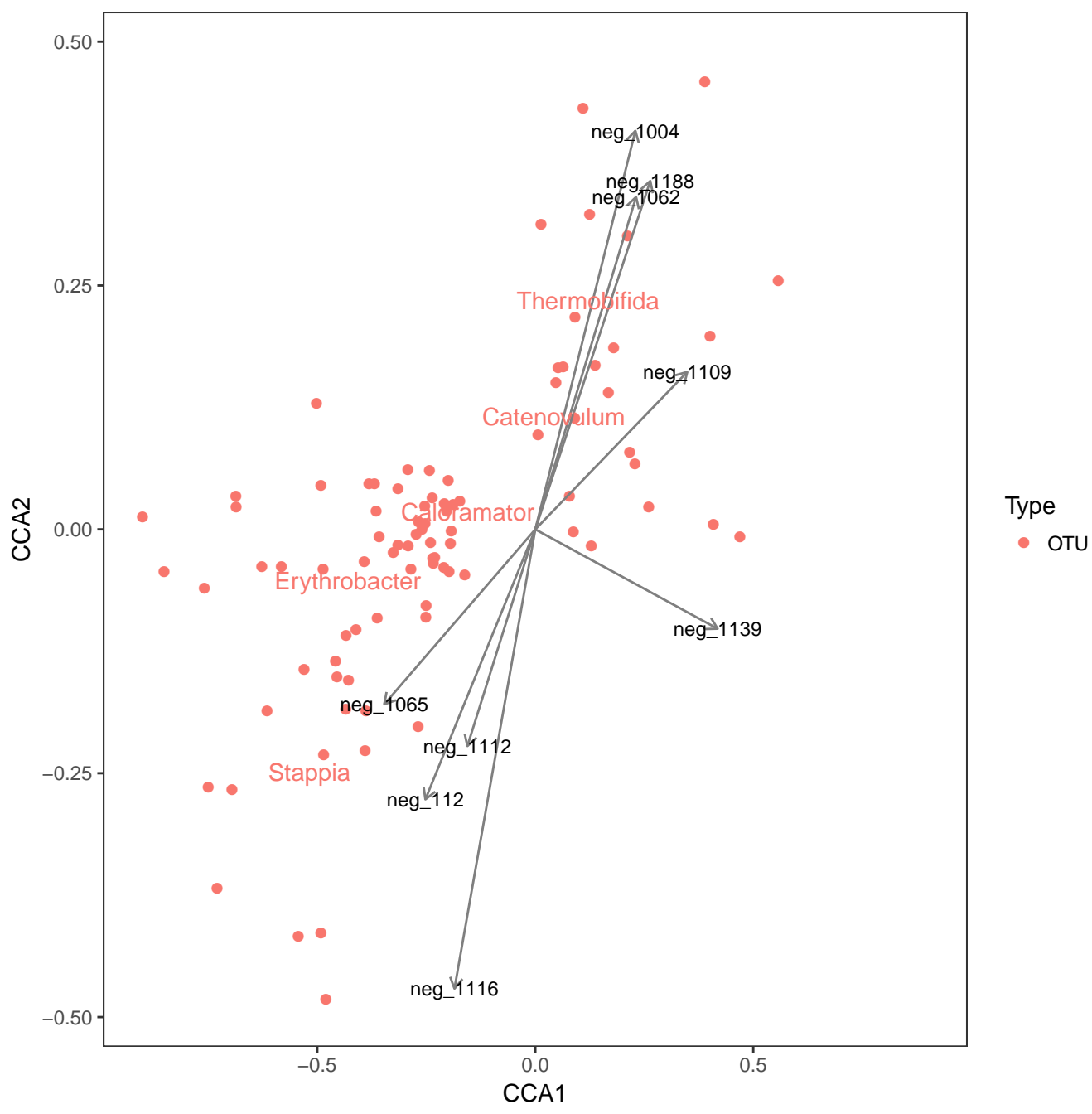

Supplement: Supplemental Information 12 [file peerj-10-14444-s012.zip › Web_Report/CCA/taxonomy/treat1_H_L.vs.H_L/CCA.pdf]

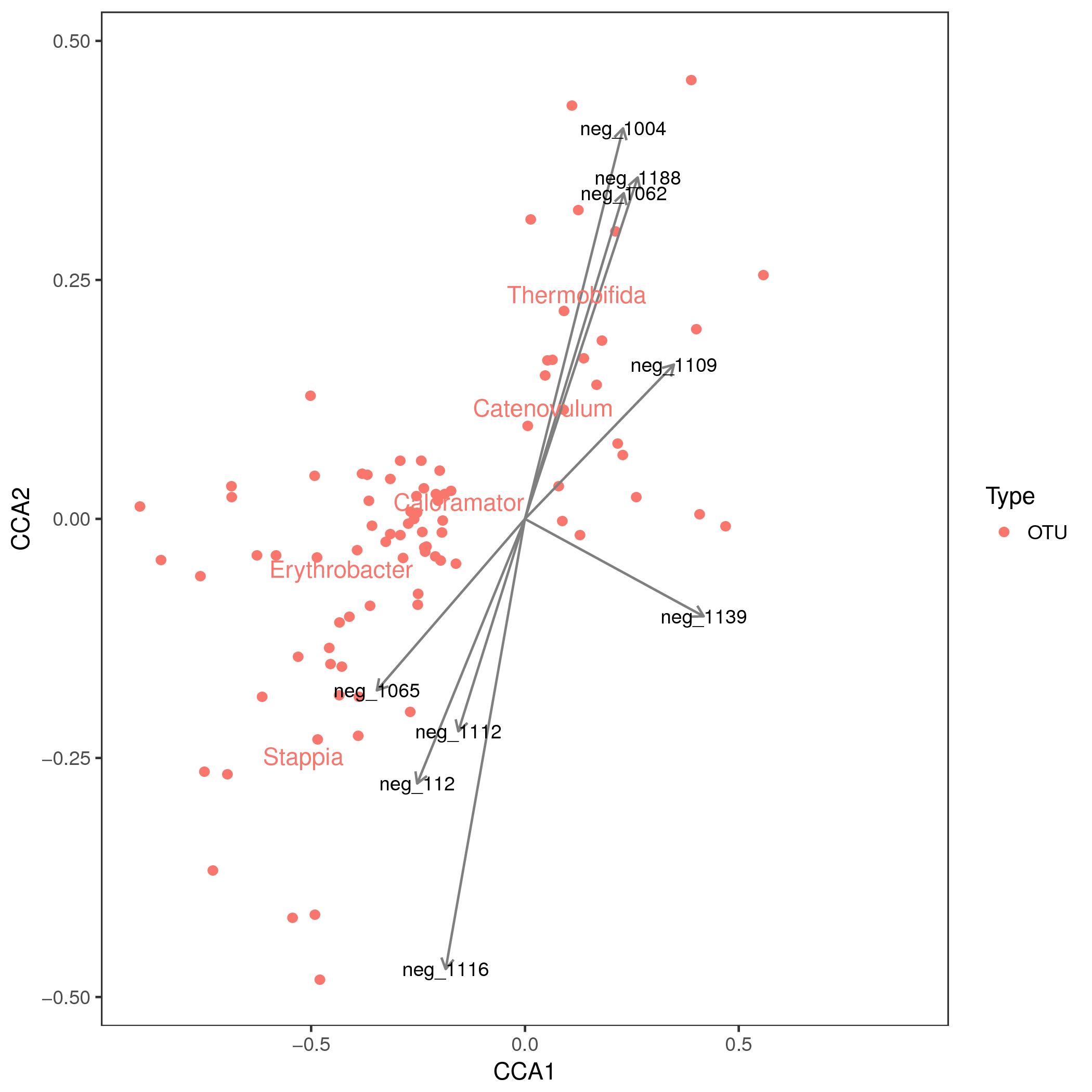

Supplement: Supplemental Information 12 [file peerj-10-14444-s012.zip › Web_Report/CCA/taxonomy/treat1_H_L.vs.H_L/CCA.png]

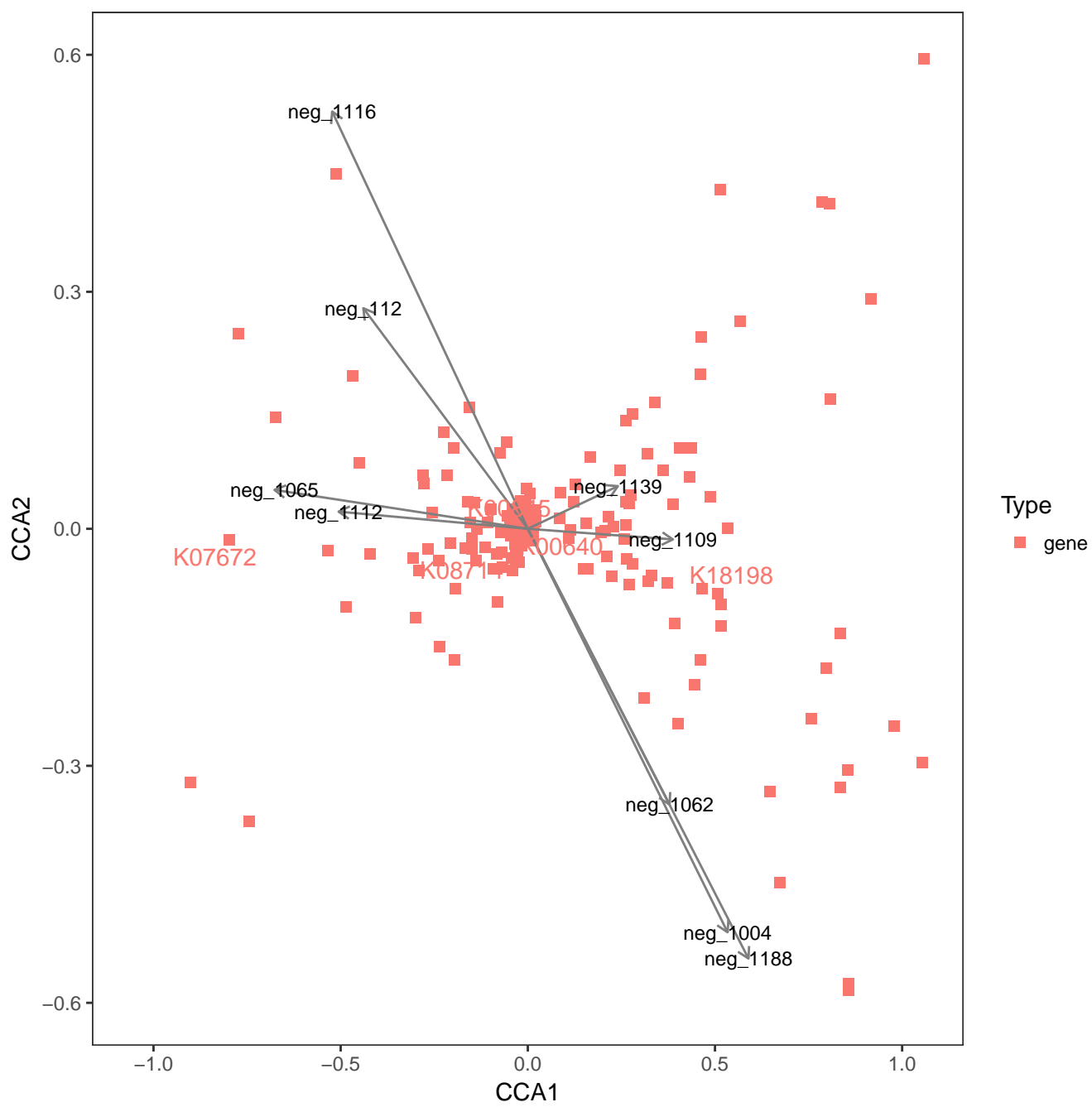

Supplement: Supplemental Information 12 [file peerj-10-14444-s012.zip › Web_Report/CCA/function/treat1_H_L.vs.H_L/CCA.pdf]

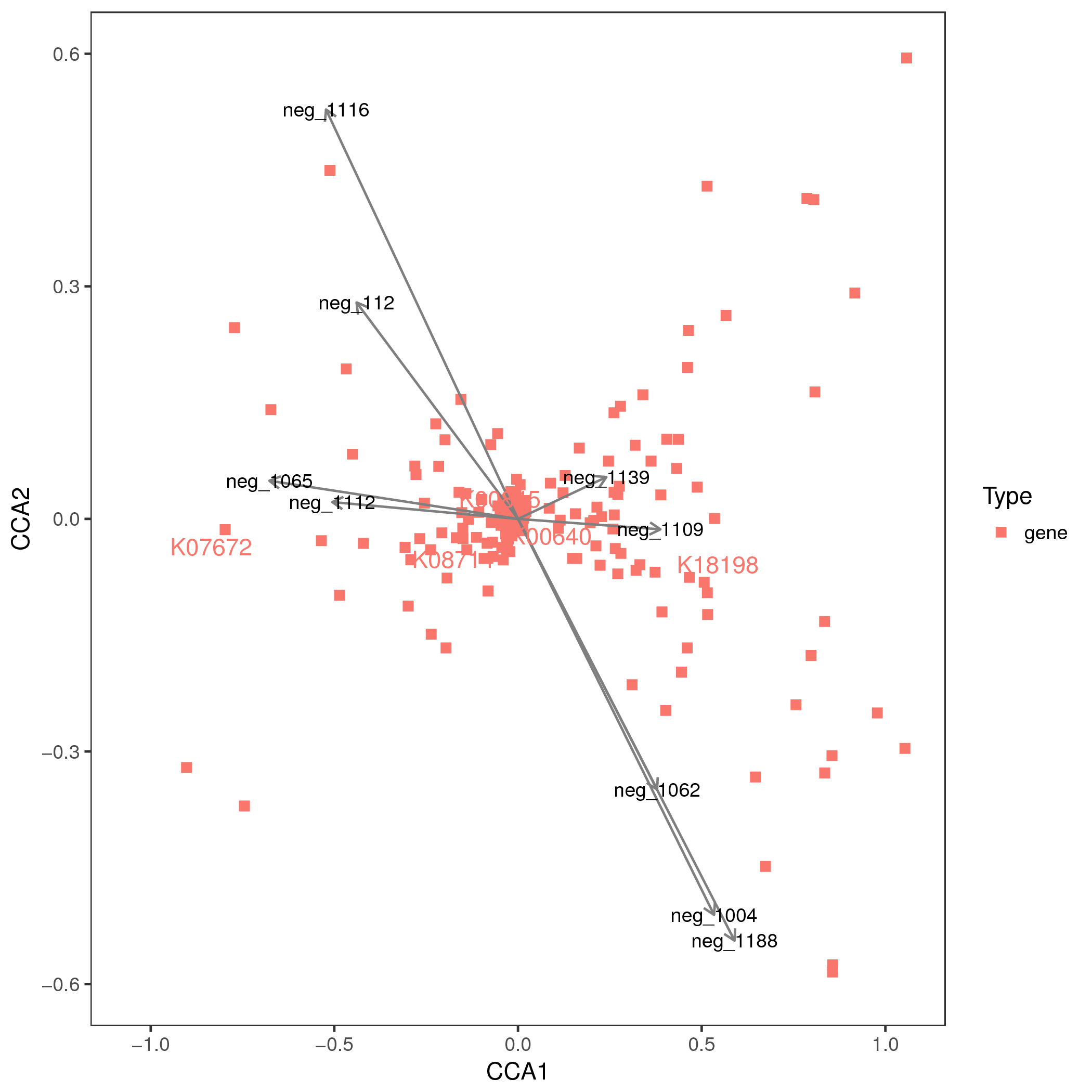

Supplement: Supplemental Information 12 [file peerj-10-14444-s012.zip › Web_Report/CCA/function/treat1_H_L.vs.H_L/CCA.png]

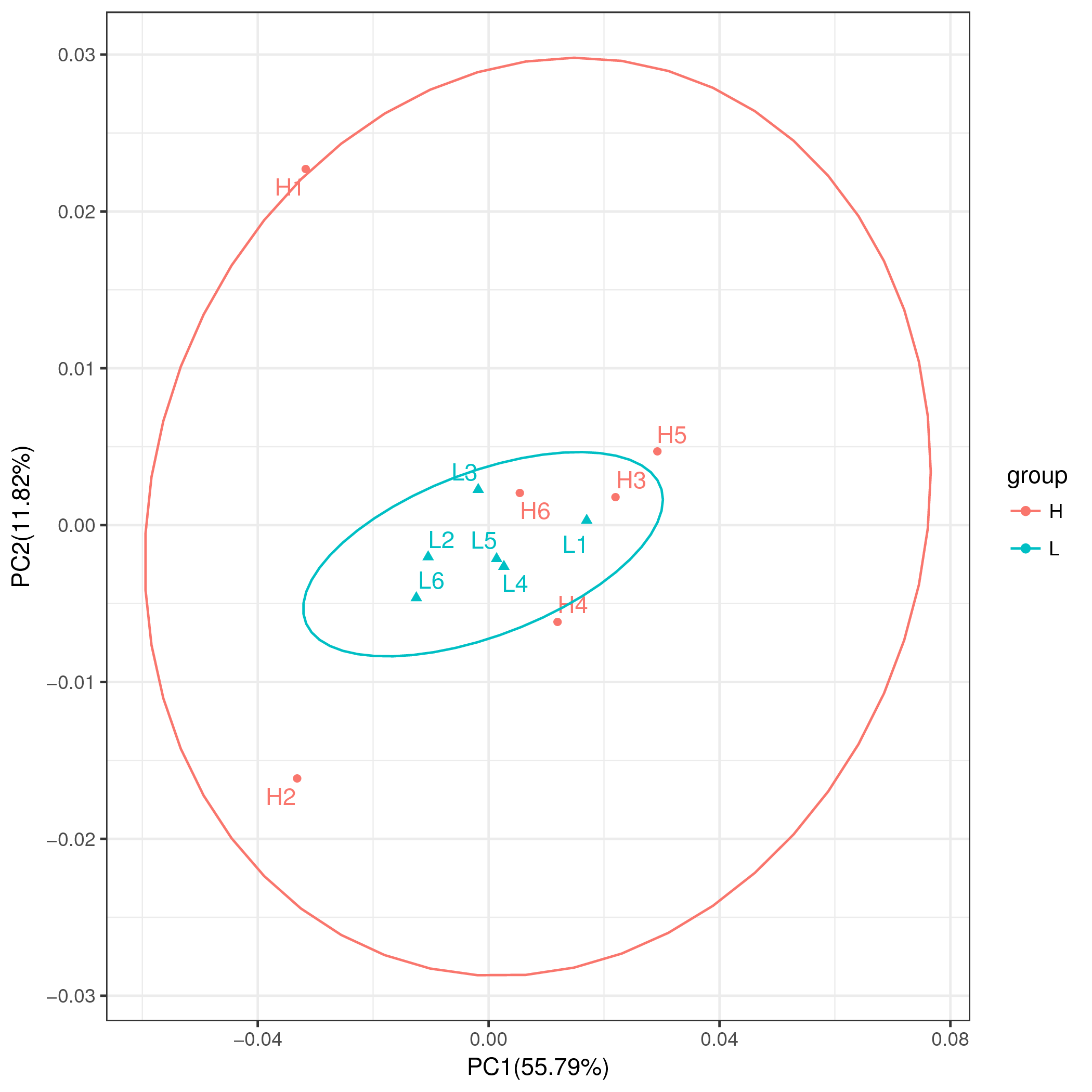

Supplement: Supplemental Information 13 [file peerj-10-14444-s013.zip › Web_Report/Data_assess/PCA/All_pca.png]

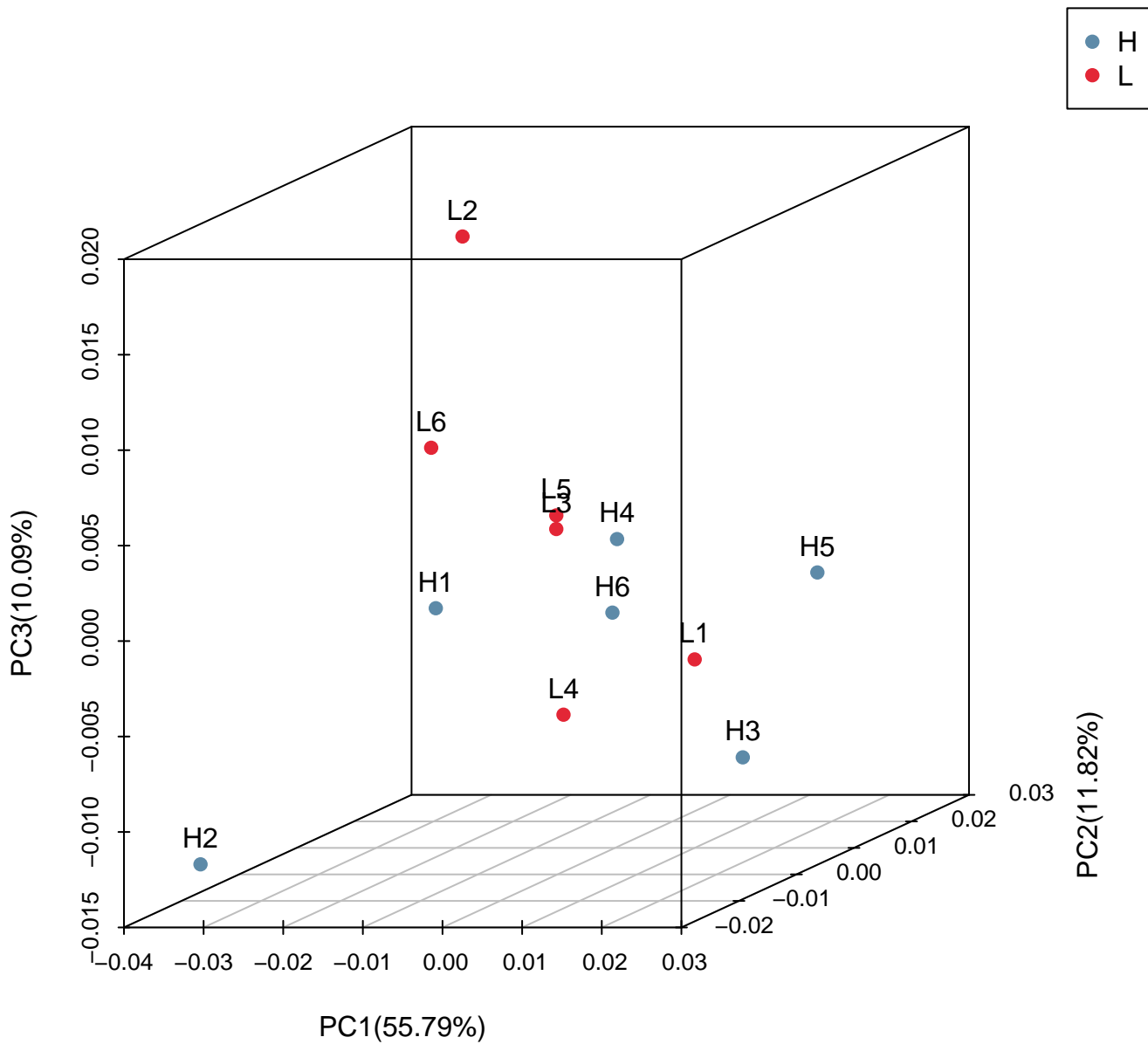

Supplement: Supplemental Information 13 [file peerj-10-14444-s013.zip › Web_Report/Data_assess/PCA/All_pca3D.pdf]

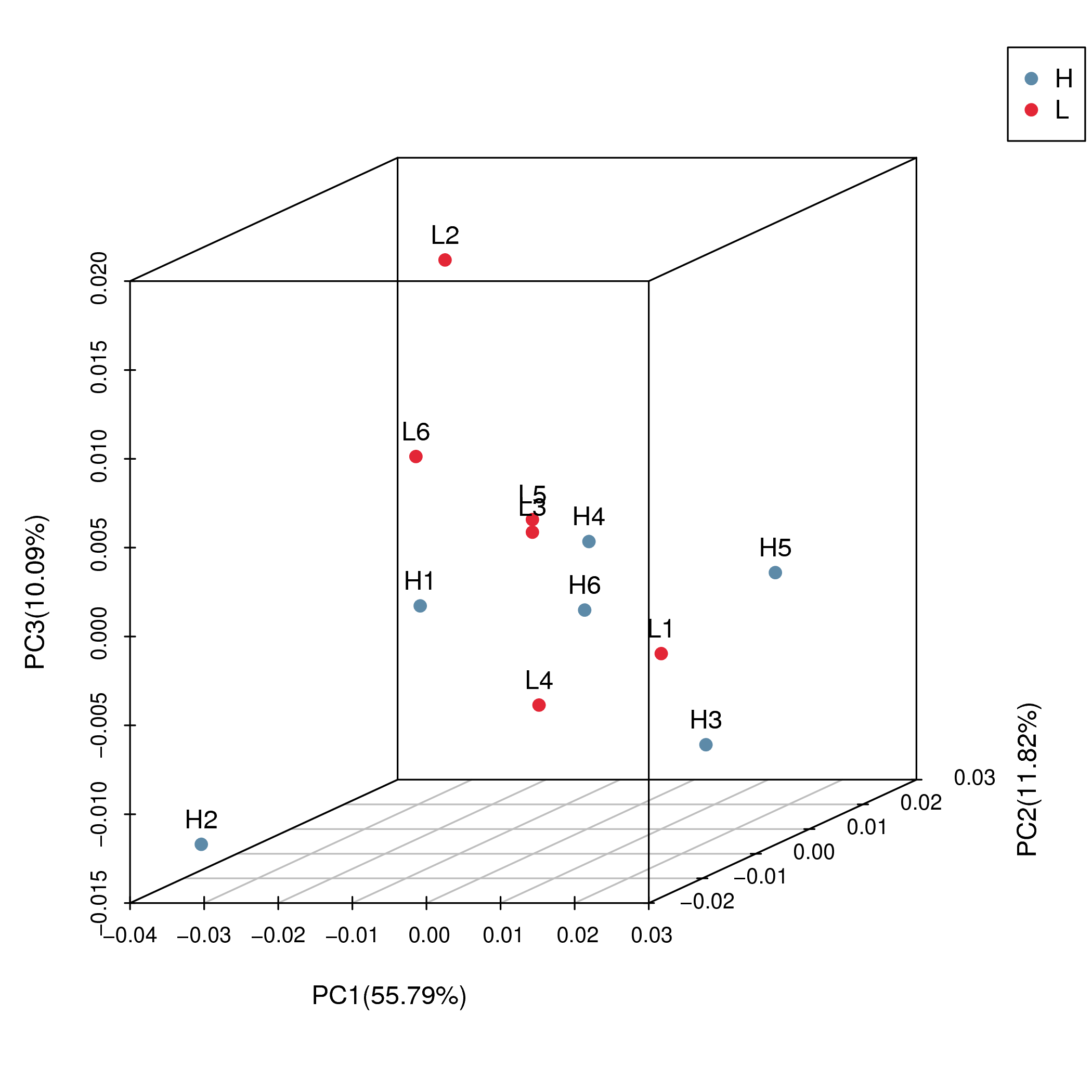

Supplement: Supplemental Information 13 [file peerj-10-14444-s013.zip › Web_Report/Data_assess/PCA/All_pca3D.png]

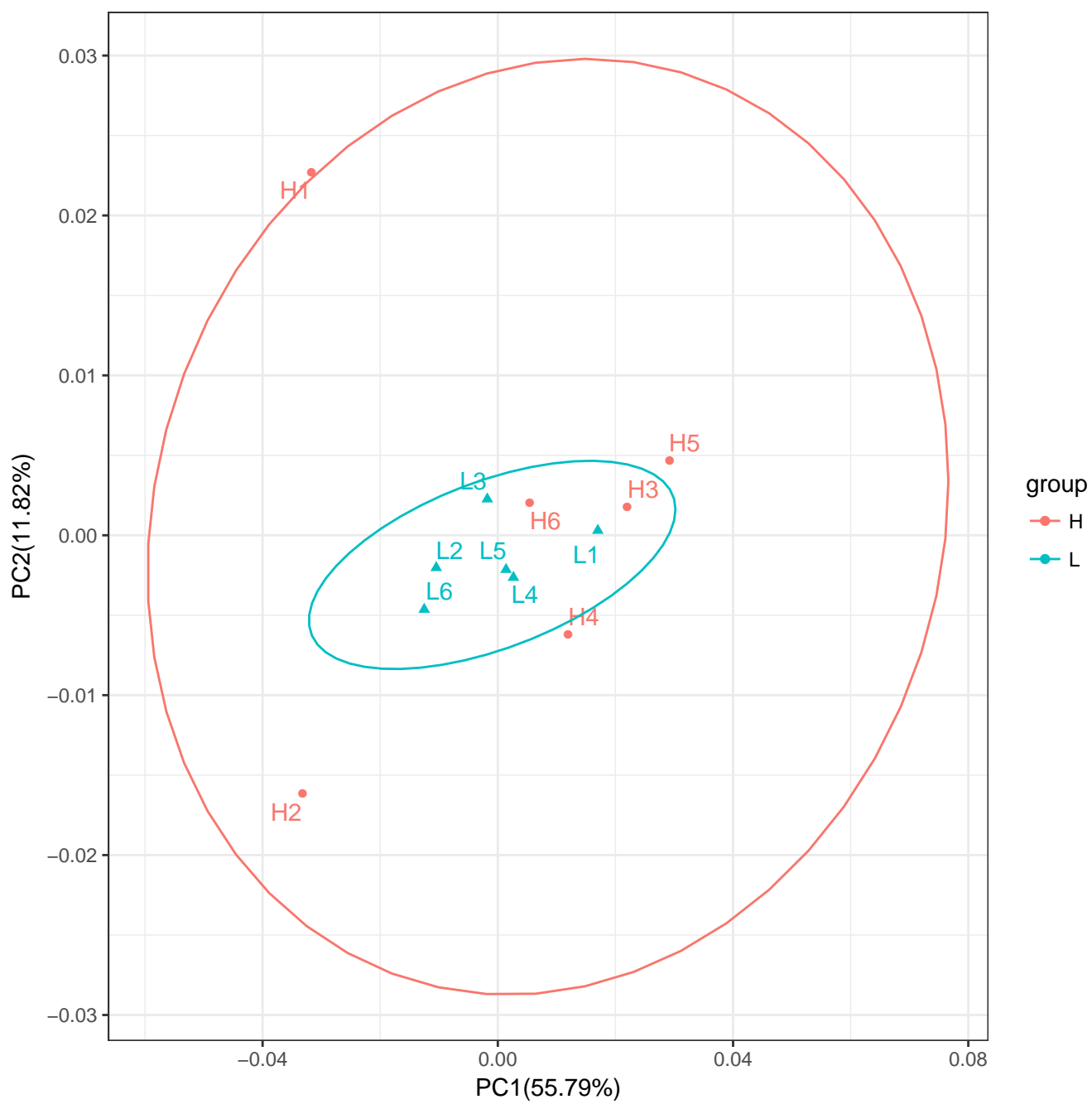

Supplement: Supplemental Information 13 [file peerj-10-14444-s013.zip › Web_Report/Data_assess/PCA/All_pca.pdf]

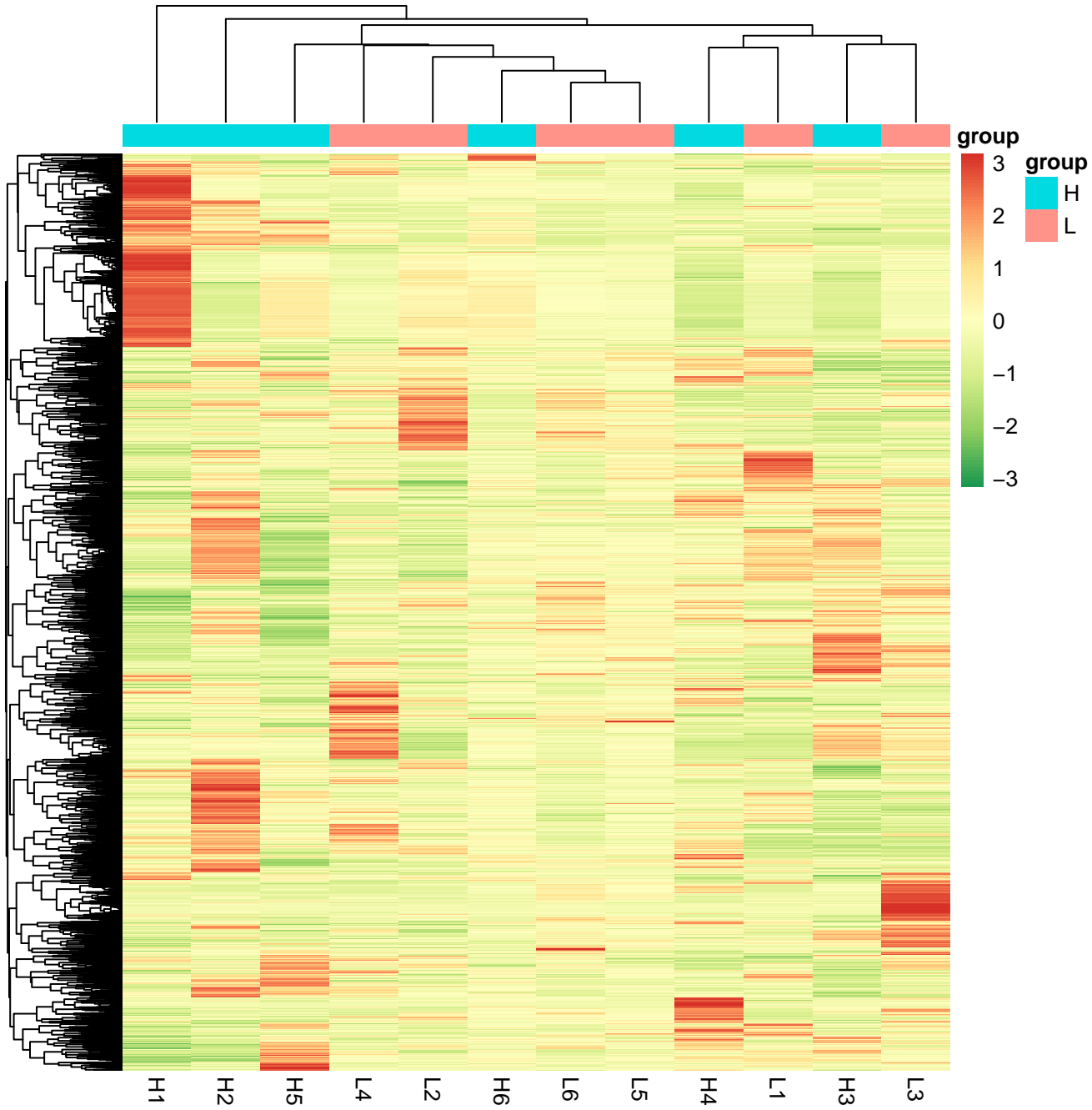

Supplement: Supplemental Information 13 [file peerj-10-14444-s013.zip › Web_Report/Data_assess/heatmap/All_heatmap.pdf]

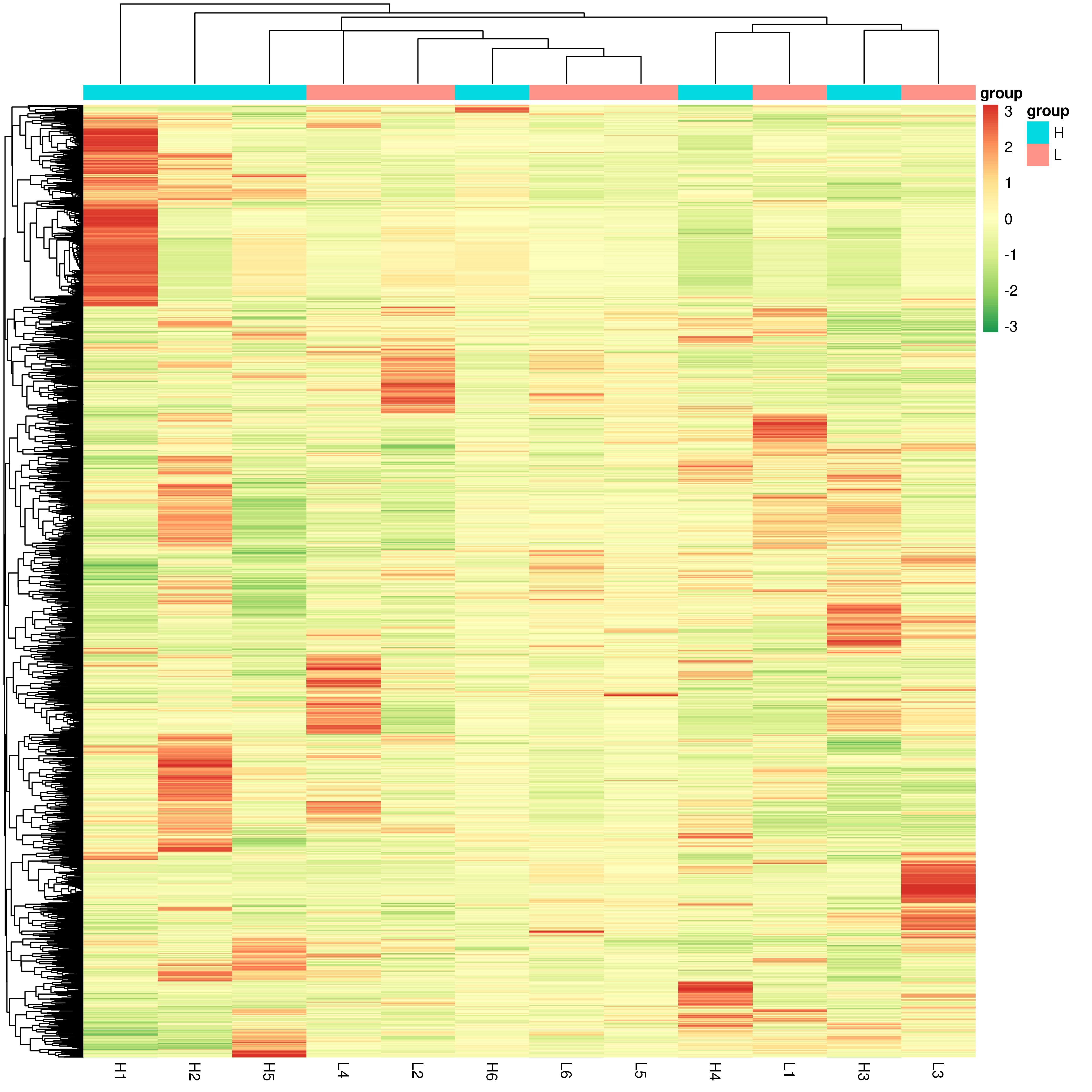

Supplement: Supplemental Information 13 [file peerj-10-14444-s013.zip › Web_Report/Data_assess/heatmap/All_heatmap.png]

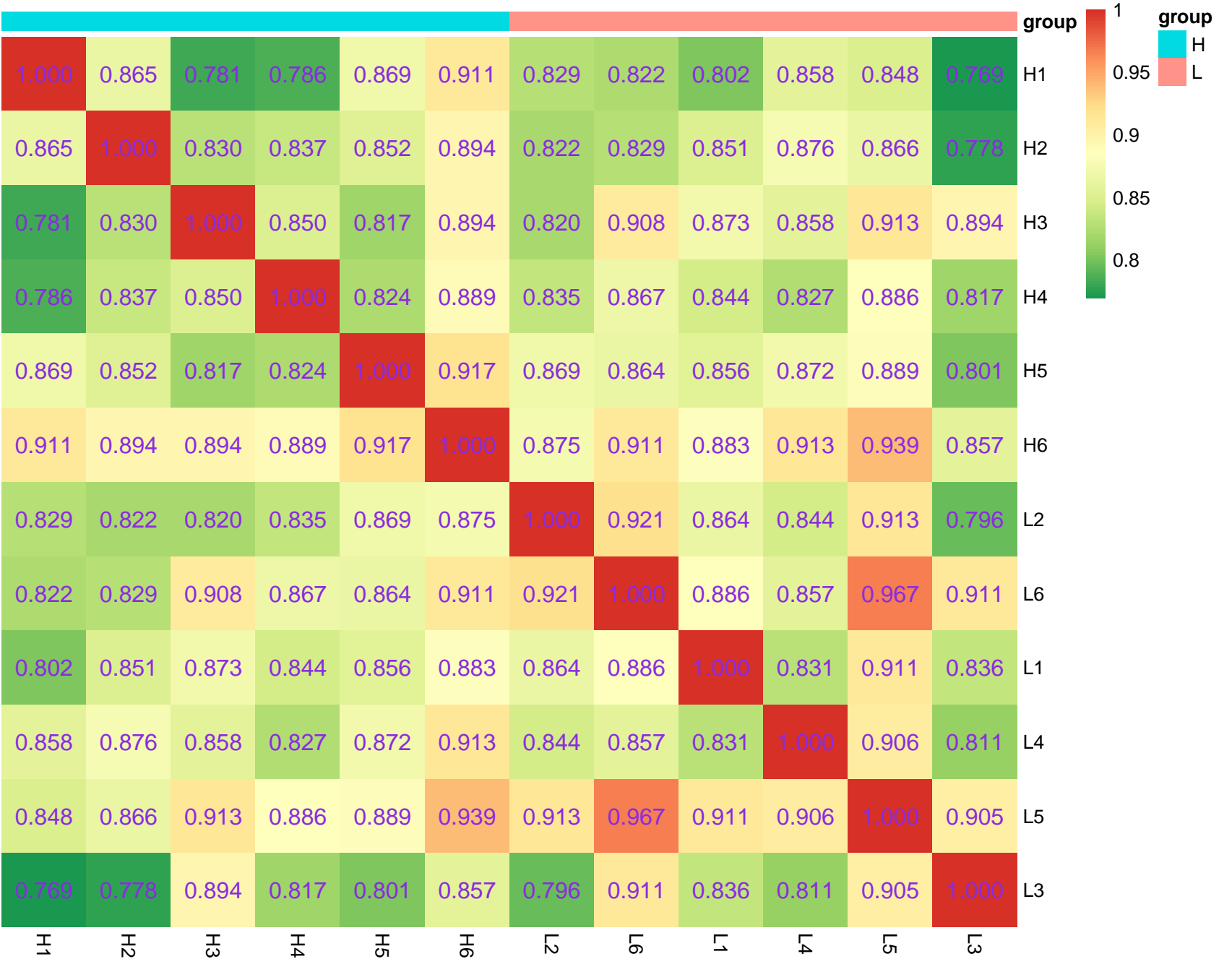

Supplement: Supplemental Information 13 [file peerj-10-14444-s013.zip › Web_Report/Data_assess/correlation_analysis/All_cor.pdf]

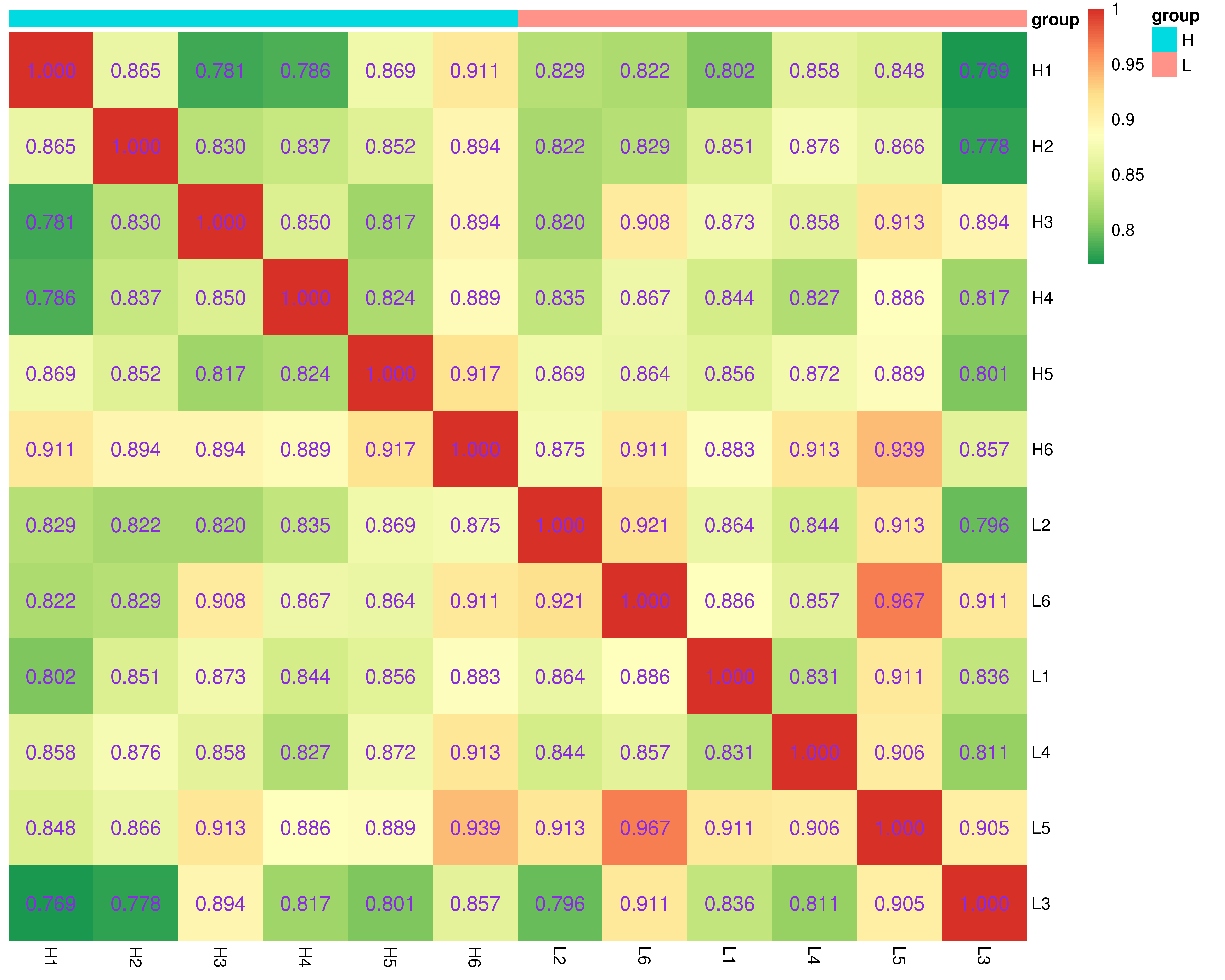

Supplement: Supplemental Information 13 [file peerj-10-14444-s013.zip › Web_Report/Data_assess/correlation_analysis/All_cor.png]

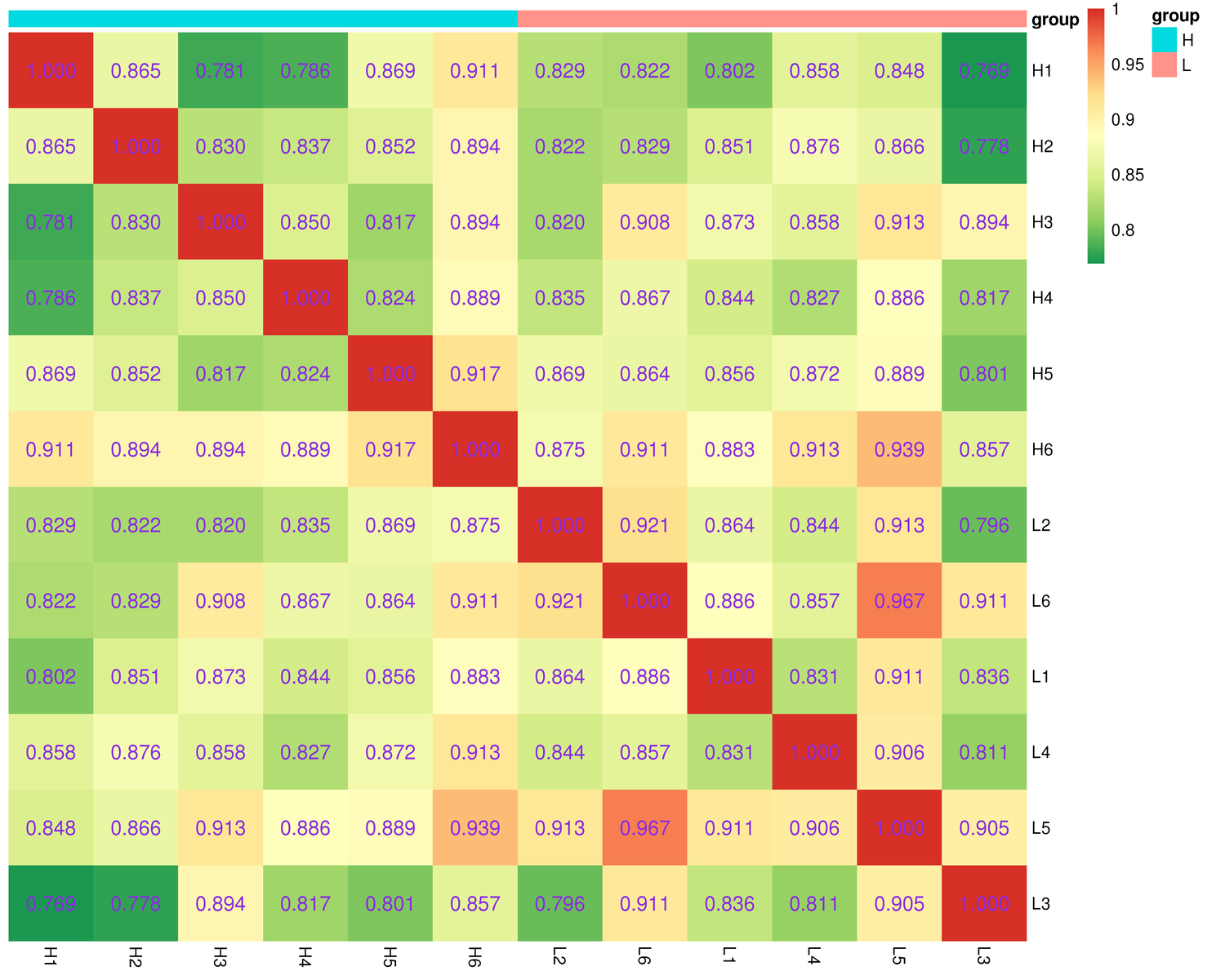

Supplement: Supplemental Information 13 [file peerj-10-14444-s013.zip › Web_Report/Quality_control/src_qc/images/All_cor.png]

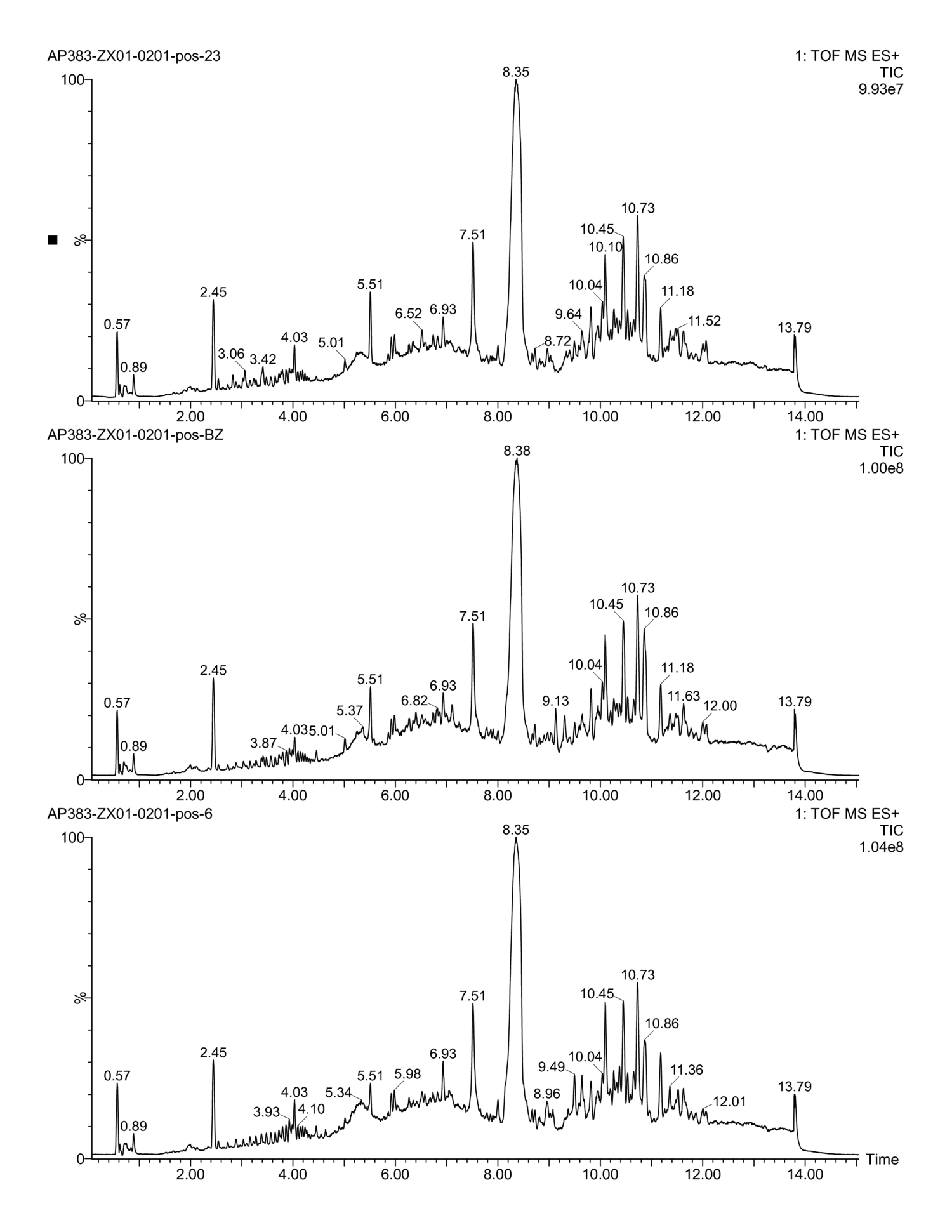

Supplement: Supplemental Information 13 [file peerj-10-14444-s013.zip › Web_Report/Quality_control/src_qc/images/tic-total-2.png]

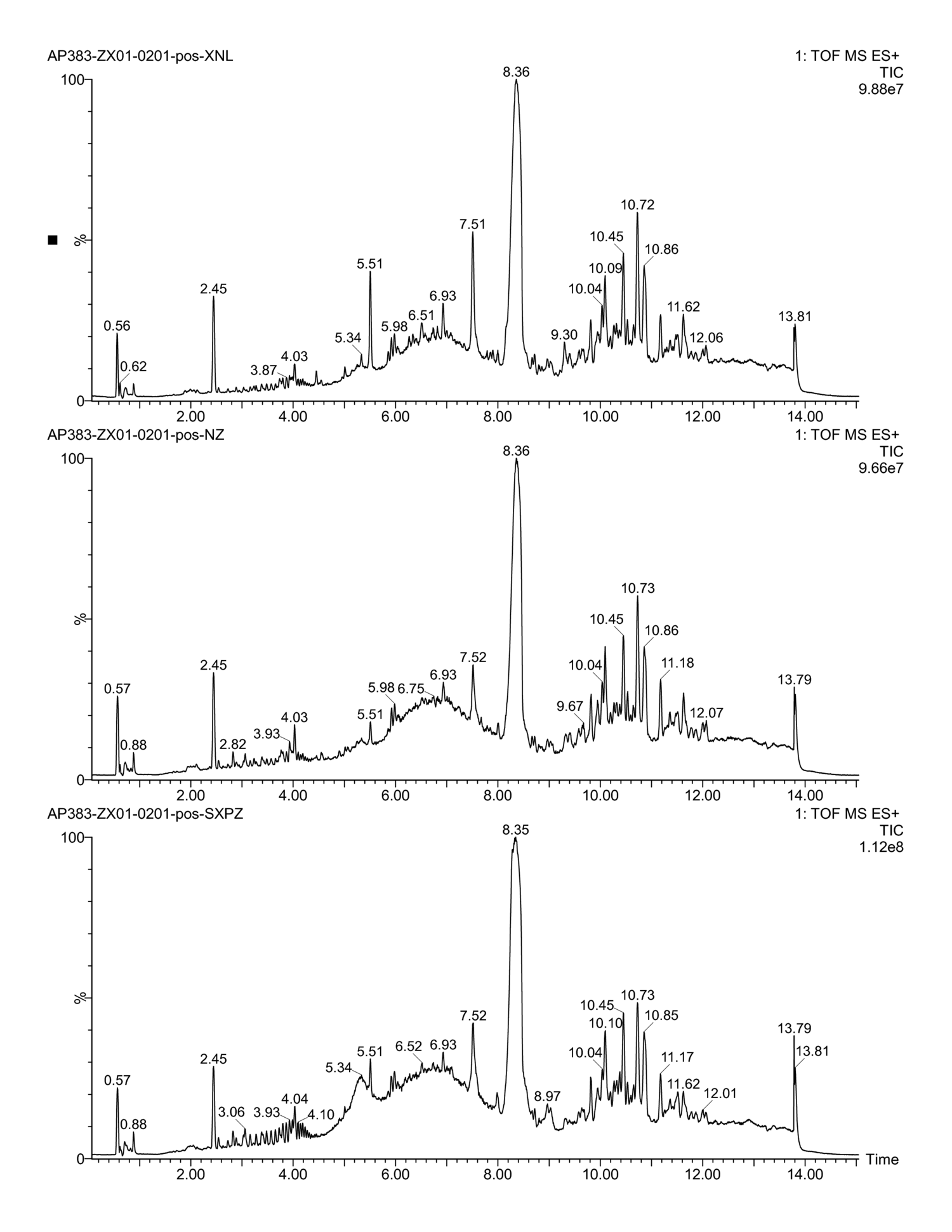

Supplement: Supplemental Information 13 [file peerj-10-14444-s013.zip › Web_Report/Quality_control/src_qc/images/tic-total-0.png]

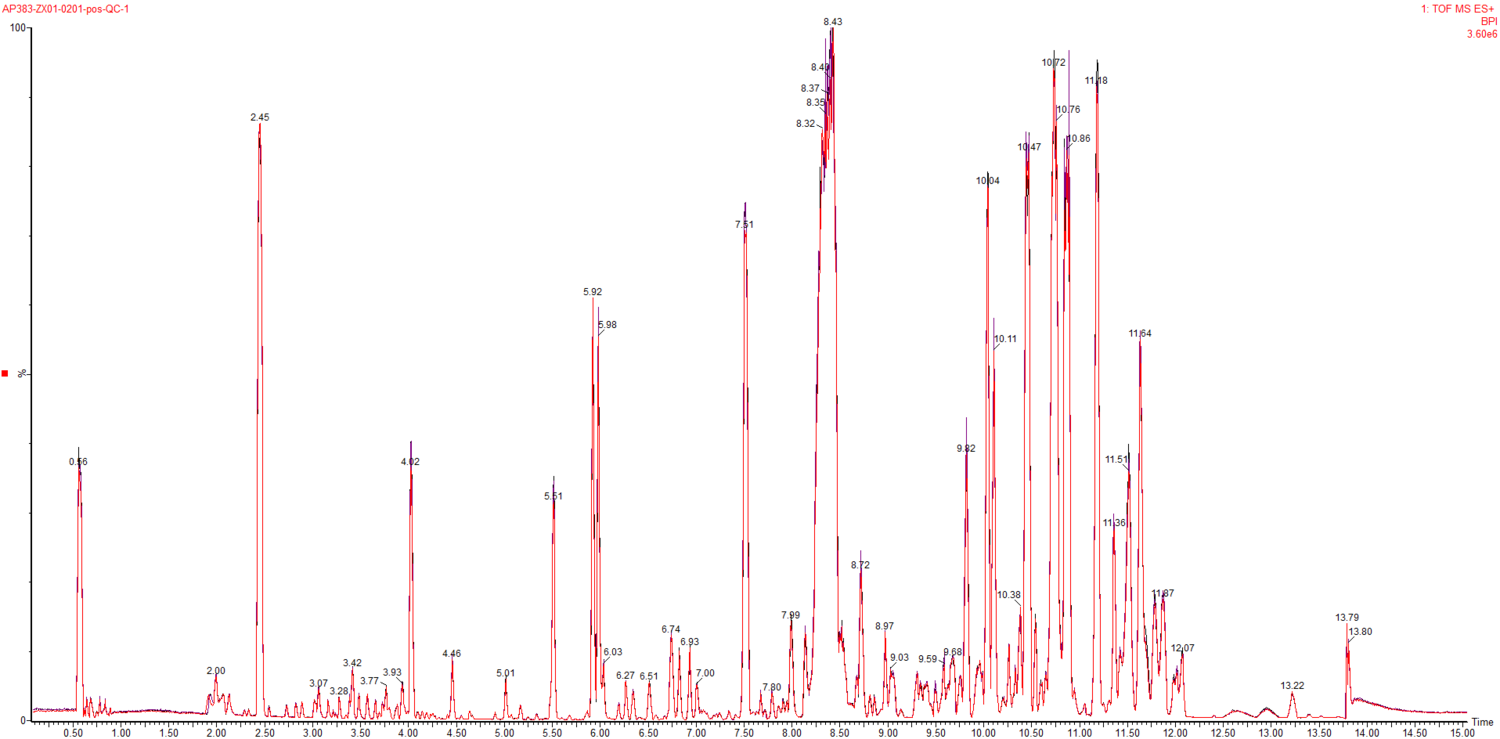

Supplement: Supplemental Information 13 [file peerj-10-14444-s013.zip › Web_Report/Quality_control/src_qc/images/bpi.png]

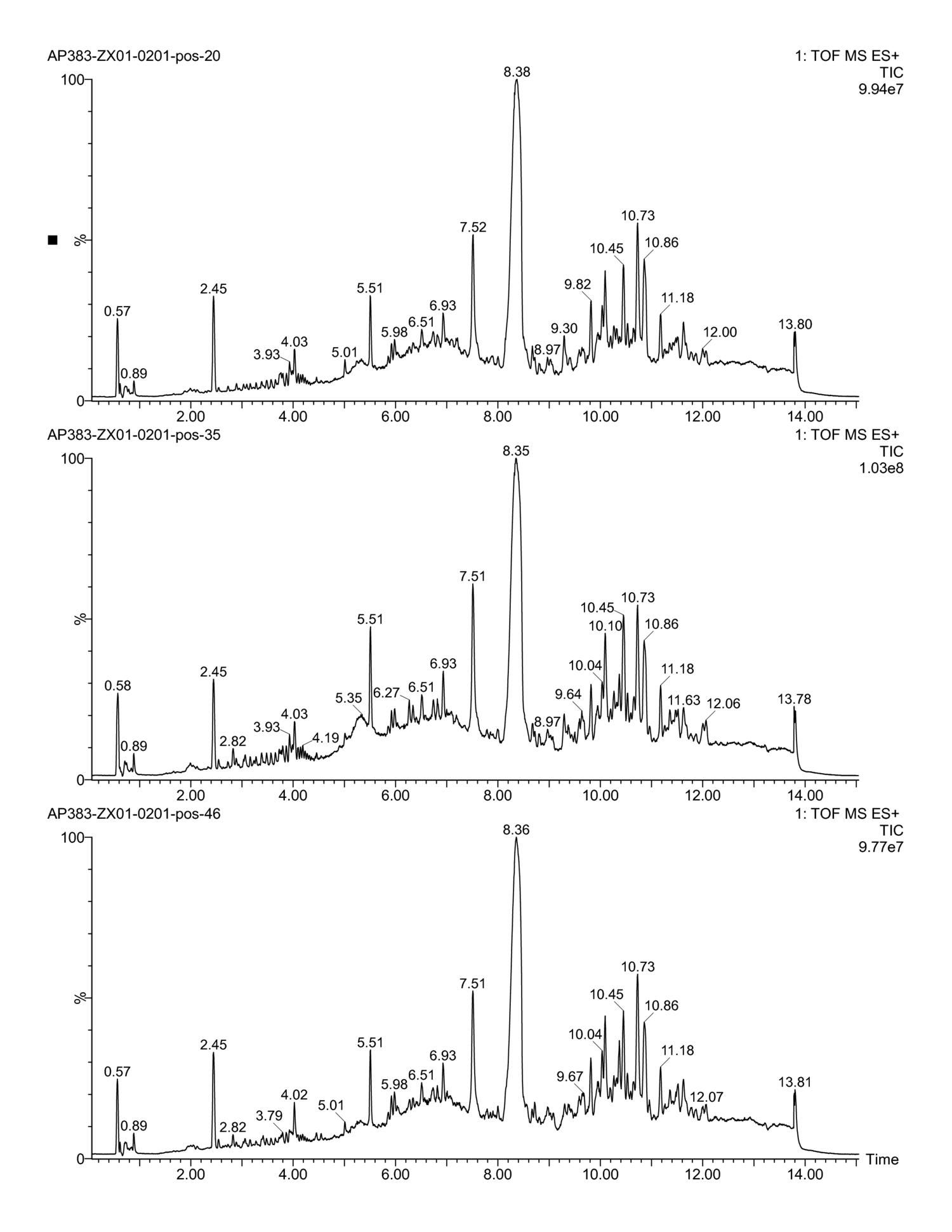

Supplement: Supplemental Information 13 [file peerj-10-14444-s013.zip › Web_Report/Quality_control/src_qc/images/tic-total-1.png]

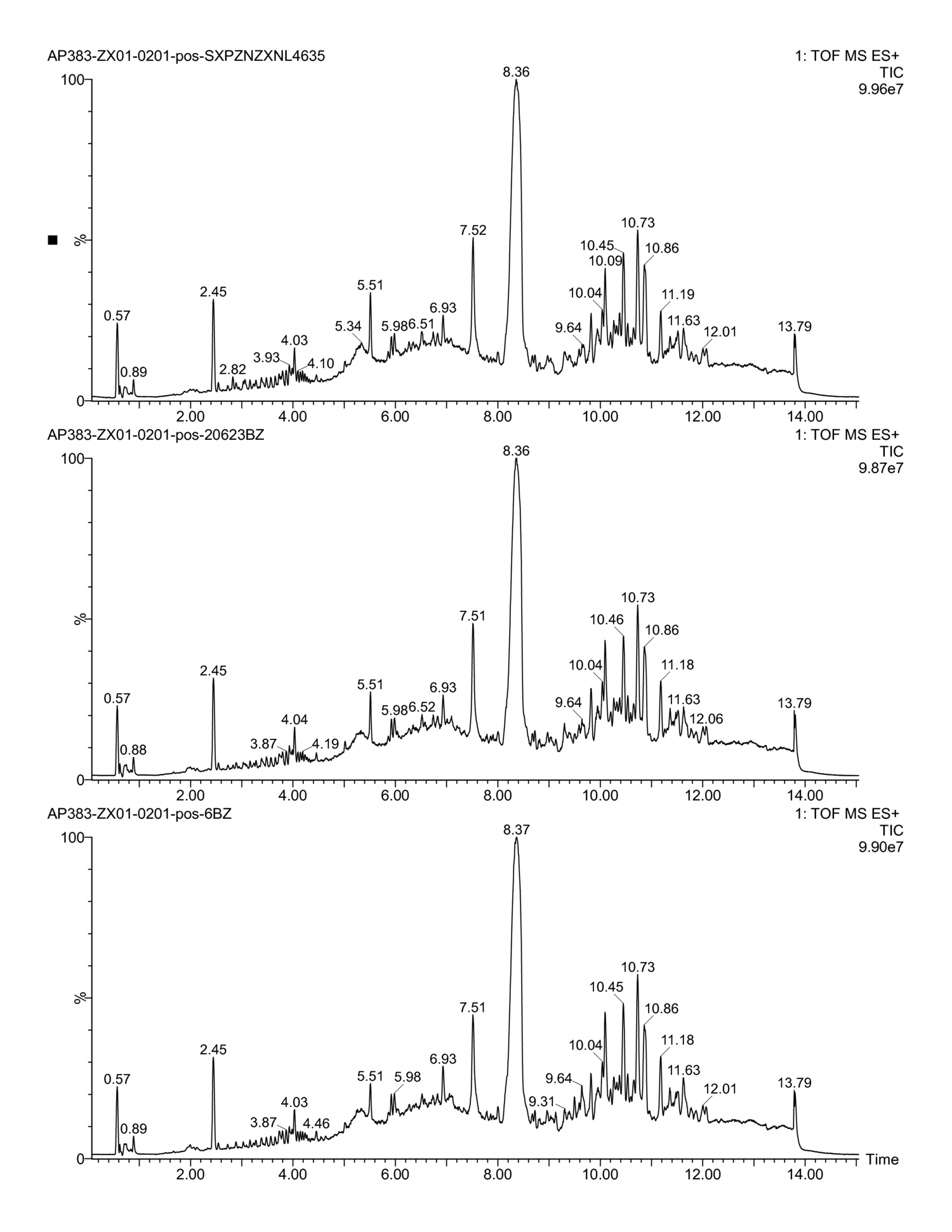

Supplement: Supplemental Information 13 [file peerj-10-14444-s013.zip › Web_Report/Quality_control/src_qc/images/tic-total-3.png]

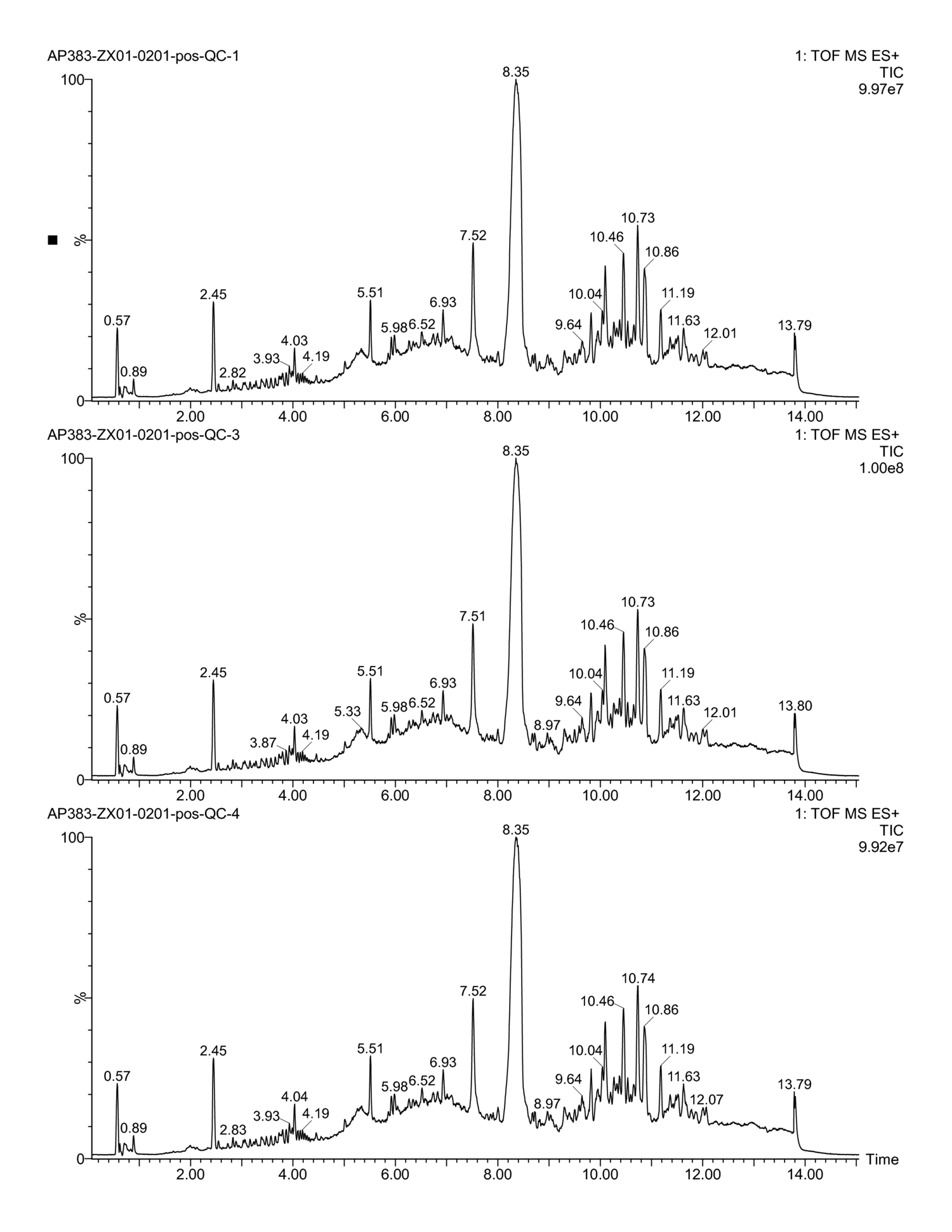

Supplement: Supplemental Information 13 [file peerj-10-14444-s013.zip › Web_Report/Quality_control/src_qc/images/tic-total-4.png]

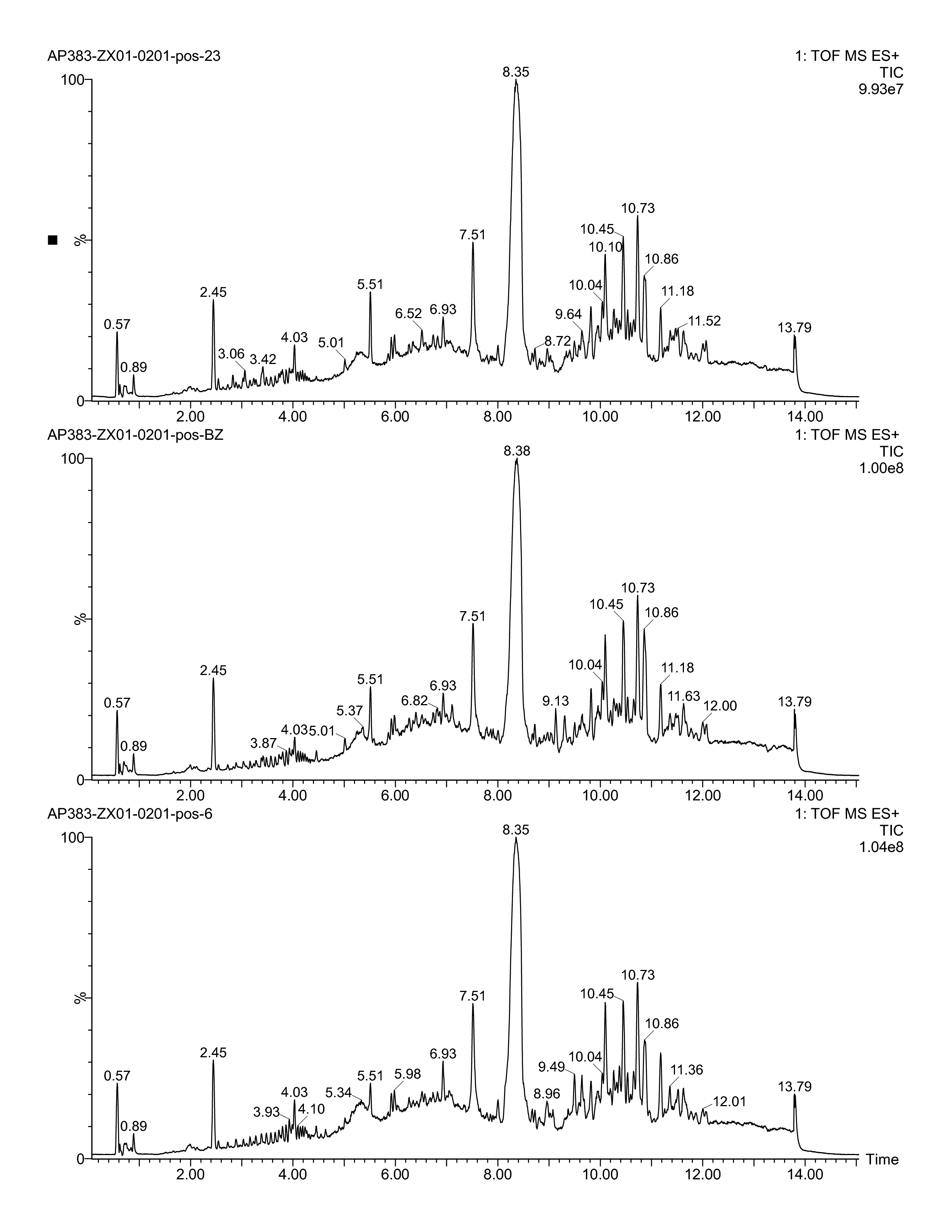

Supplement: Supplemental Information 13 [file peerj-10-14444-s013.zip › Web_Report/Quality_control/qc_data/tic-total-2.png]

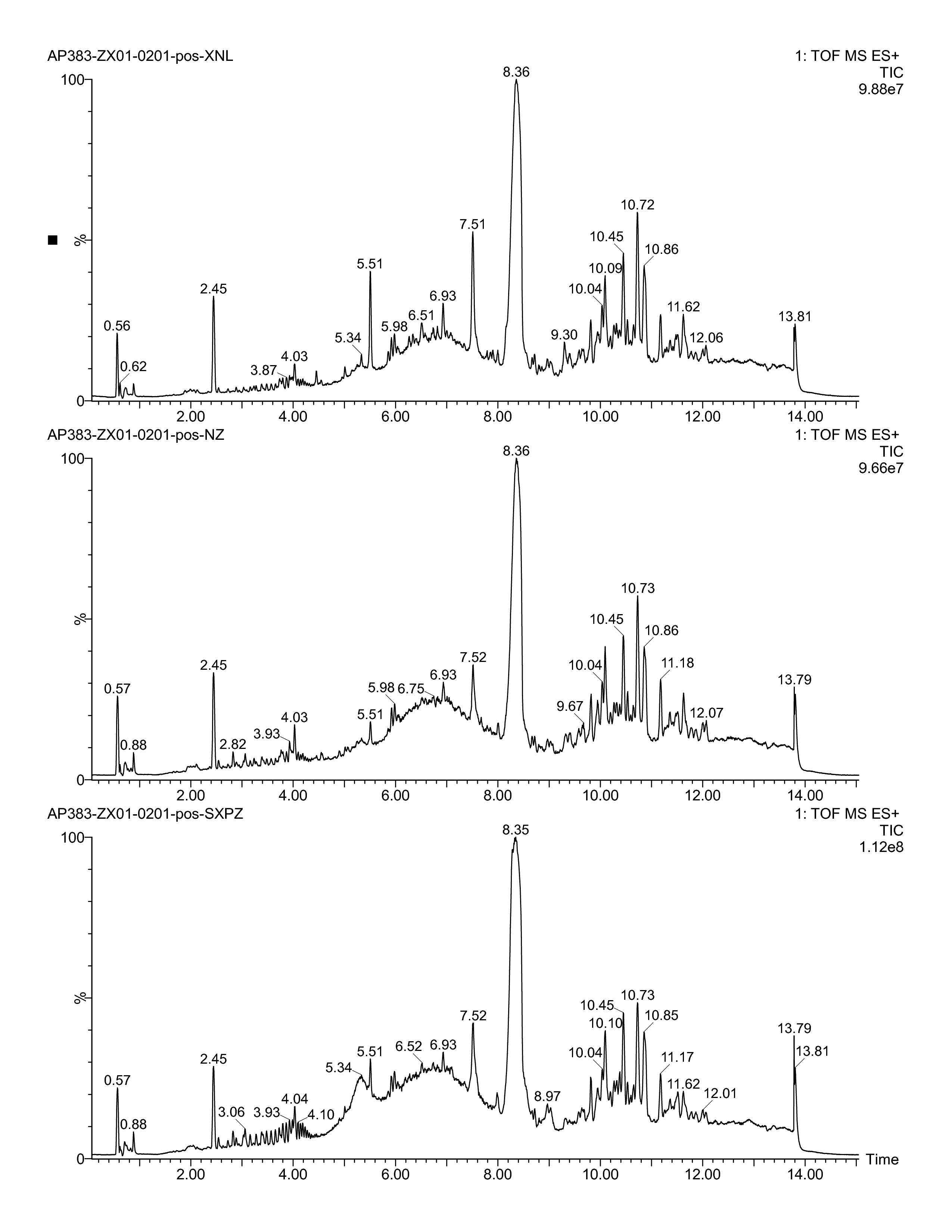

Supplement: Supplemental Information 13 [file peerj-10-14444-s013.zip › Web_Report/Quality_control/qc_data/tic-total-0.png]

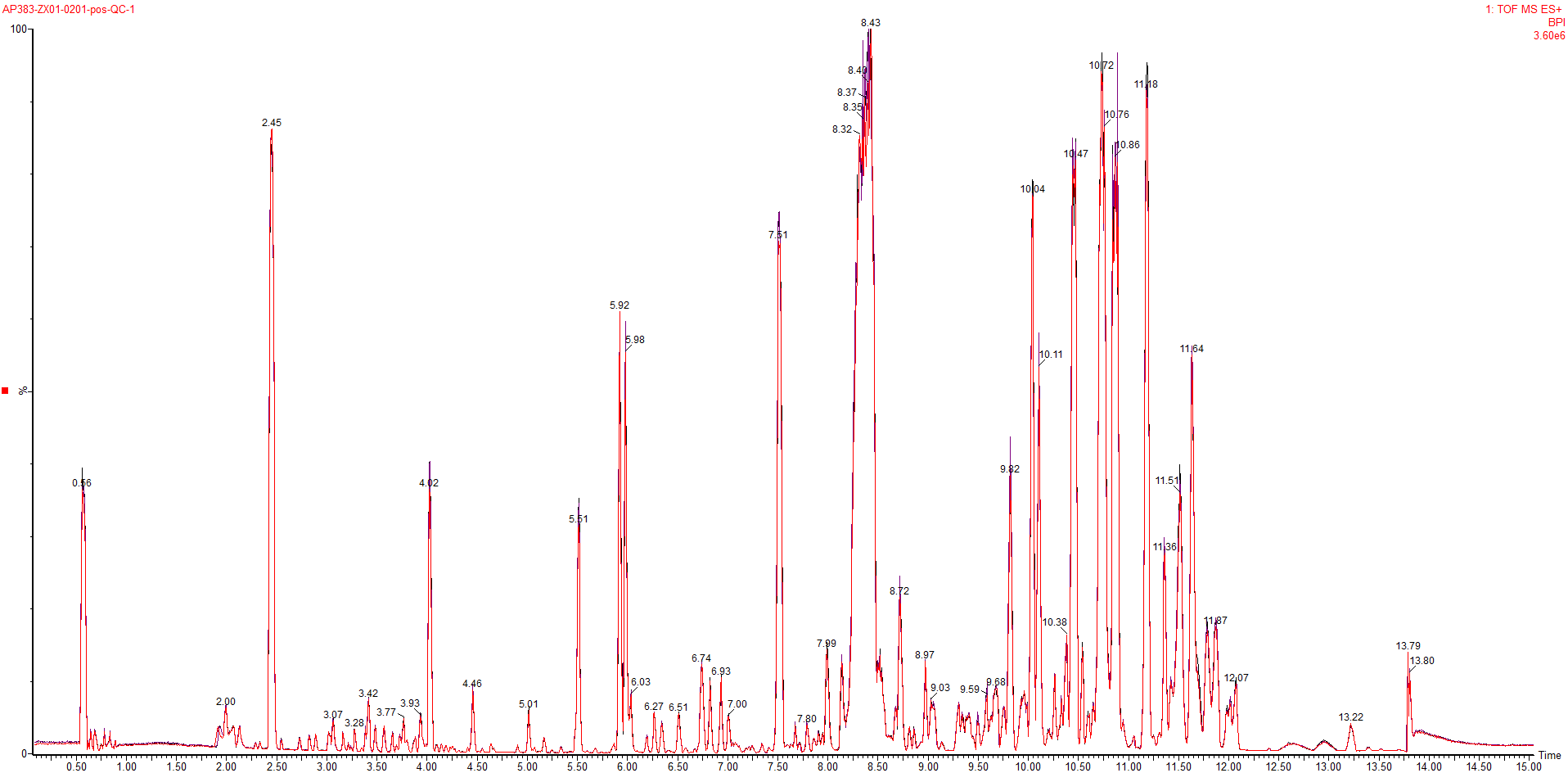

Supplement: Supplemental Information 13 [file peerj-10-14444-s013.zip › Web_Report/Quality_control/qc_data/bpi.png]

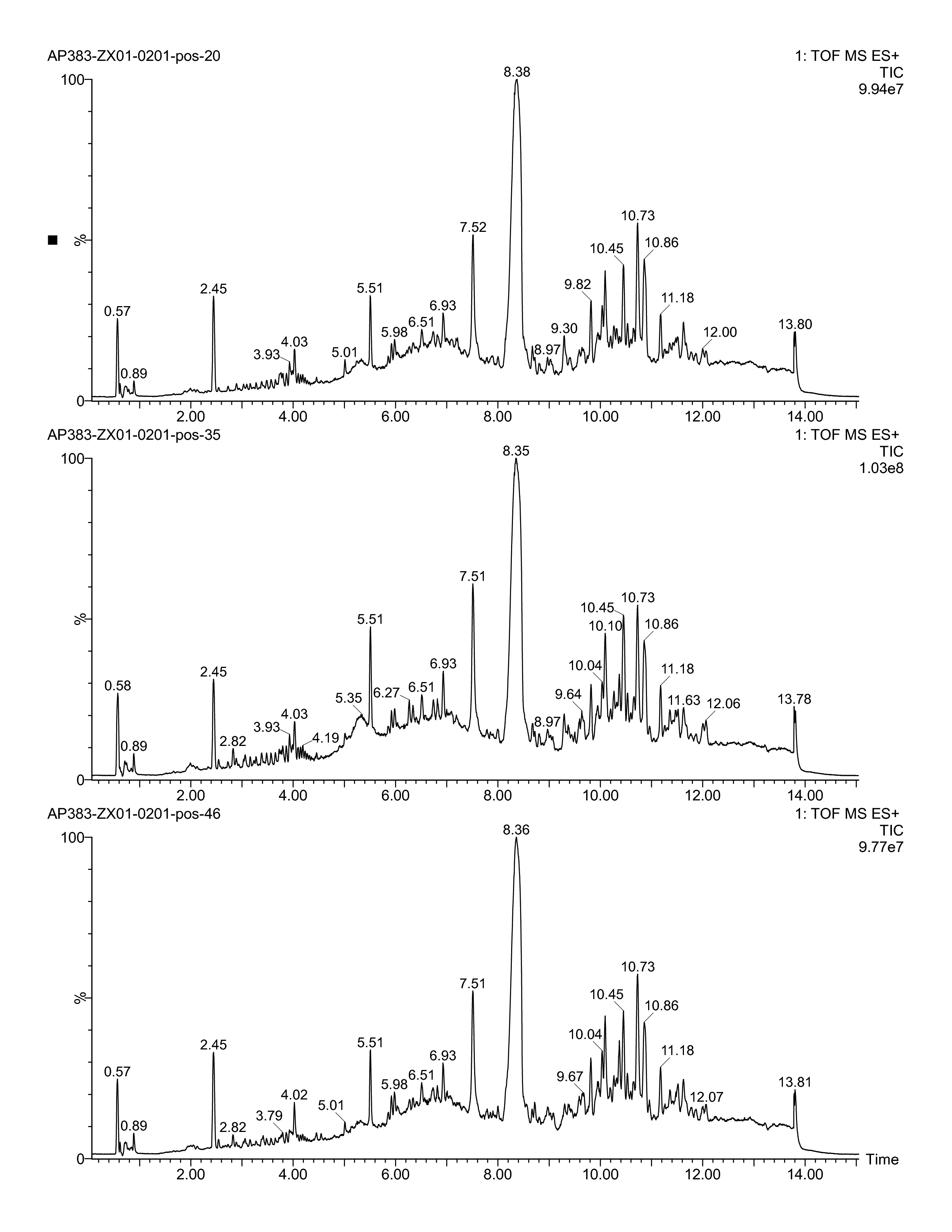

Supplement: Supplemental Information 13 [file peerj-10-14444-s013.zip › Web_Report/Quality_control/qc_data/tic-total-1.png]

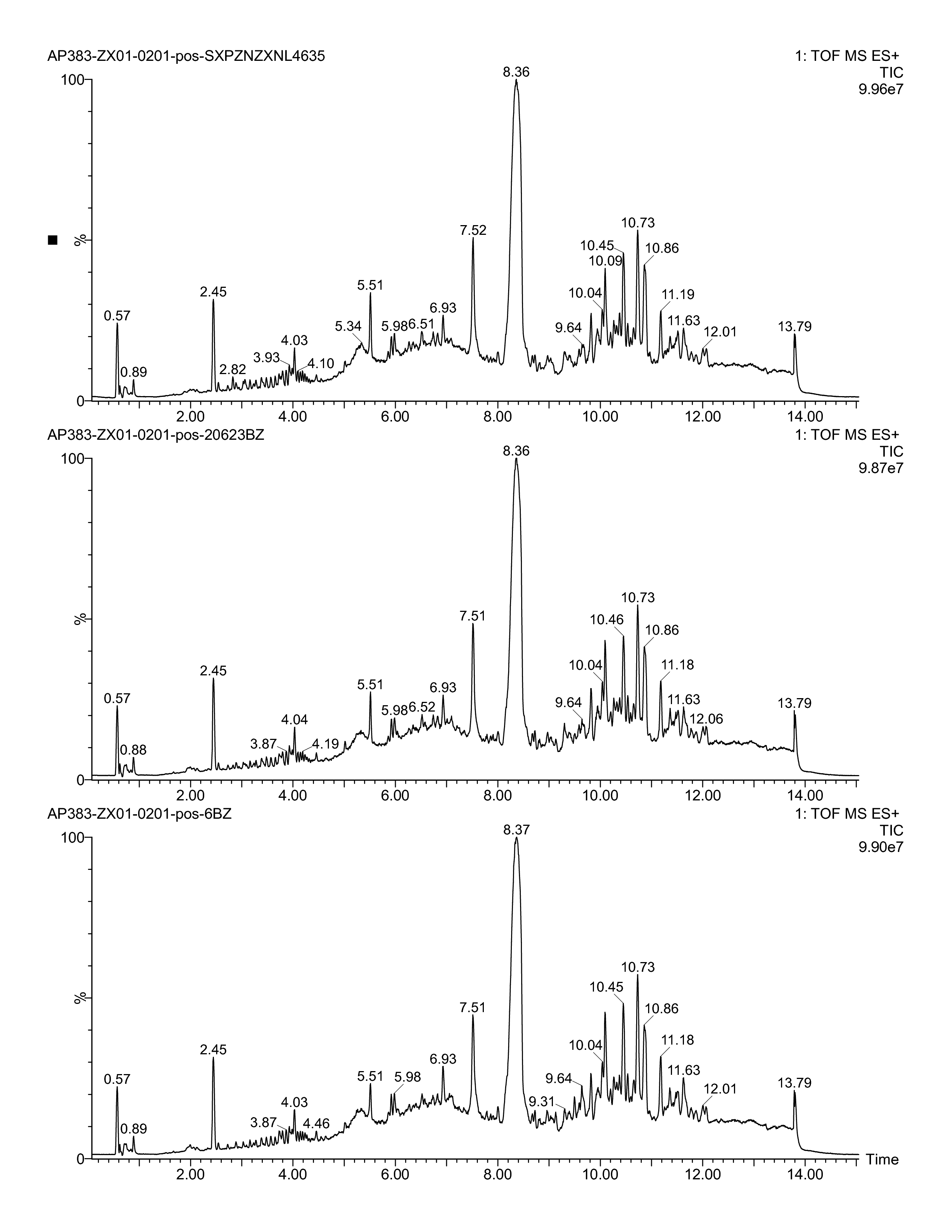

Supplement: Supplemental Information 13 [file peerj-10-14444-s013.zip › Web_Report/Quality_control/qc_data/tic-total-3.png]

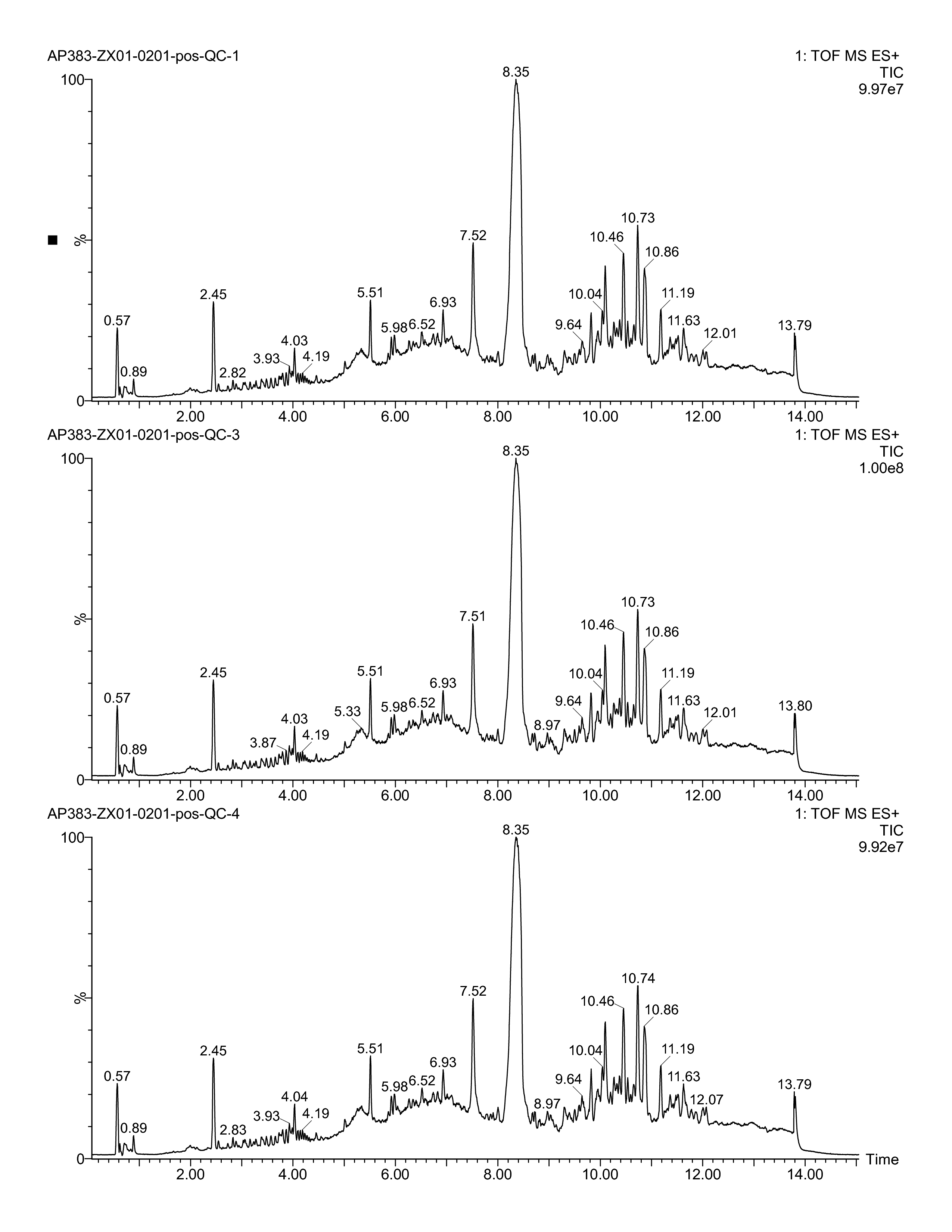

Supplement: Supplemental Information 13 [file peerj-10-14444-s013.zip › Web_Report/Quality_control/qc_data/tic-total-4.png]

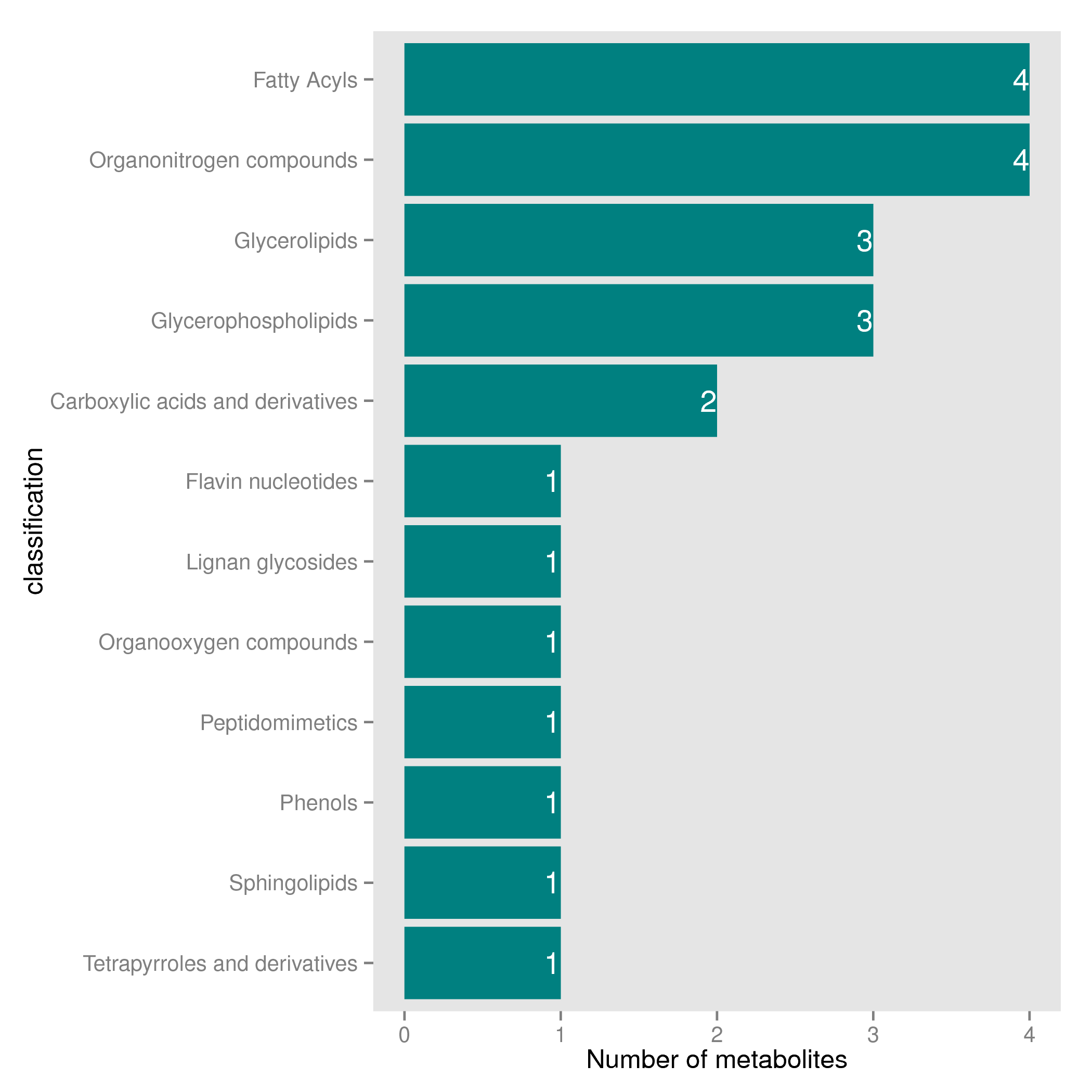

Supplement: Supplemental Information 13 [file peerj-10-14444-s013.zip › Web_Report/Diff_analysis/H_vs_L/H_vs_L_metabolites_classification_top20.png]

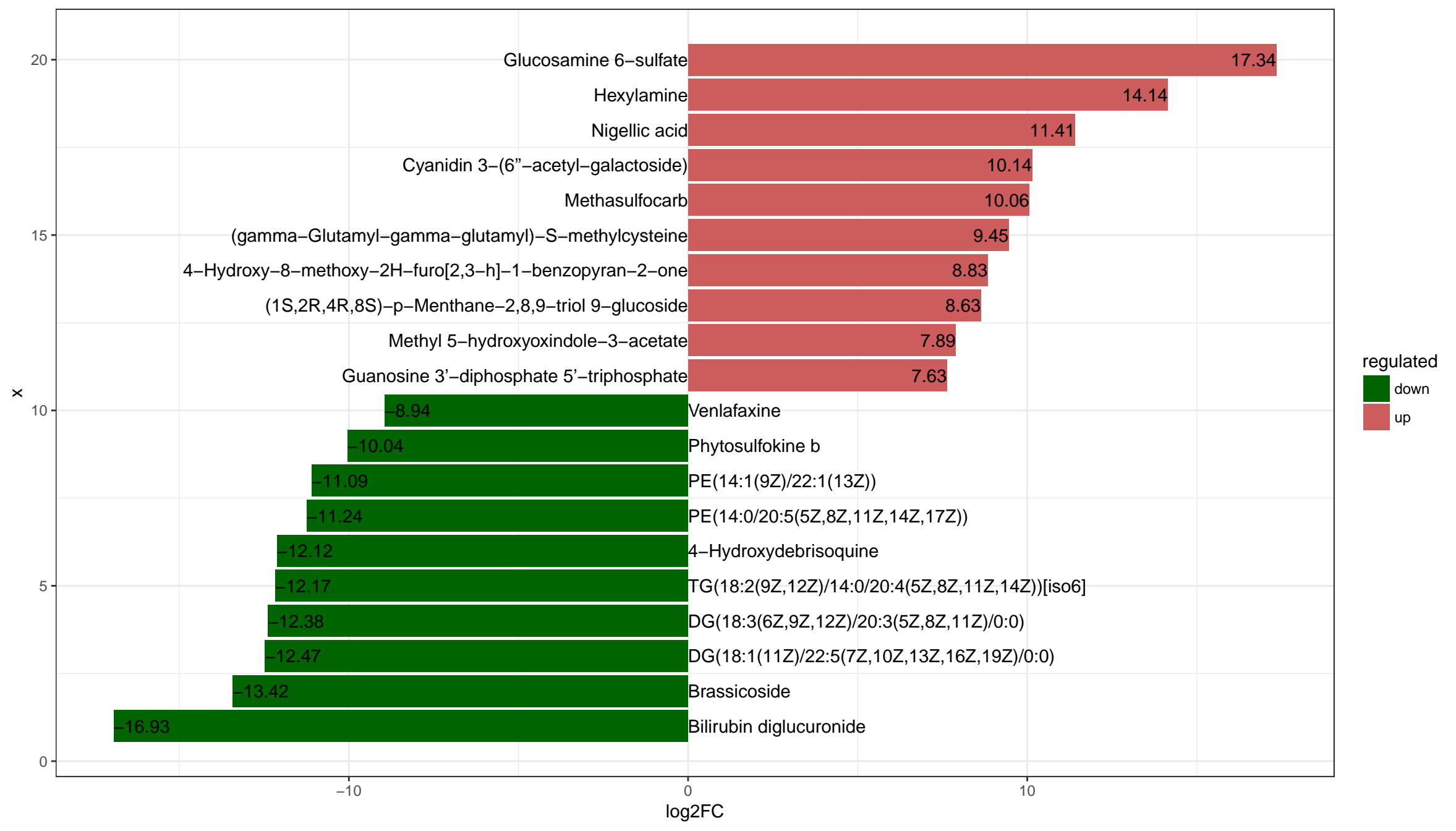

Supplement: Supplemental Information 13 [file peerj-10-14444-s013.zip › Web_Report/Diff_analysis/H_vs_L/H_vs_L_Top_20_FC_change.pdf]

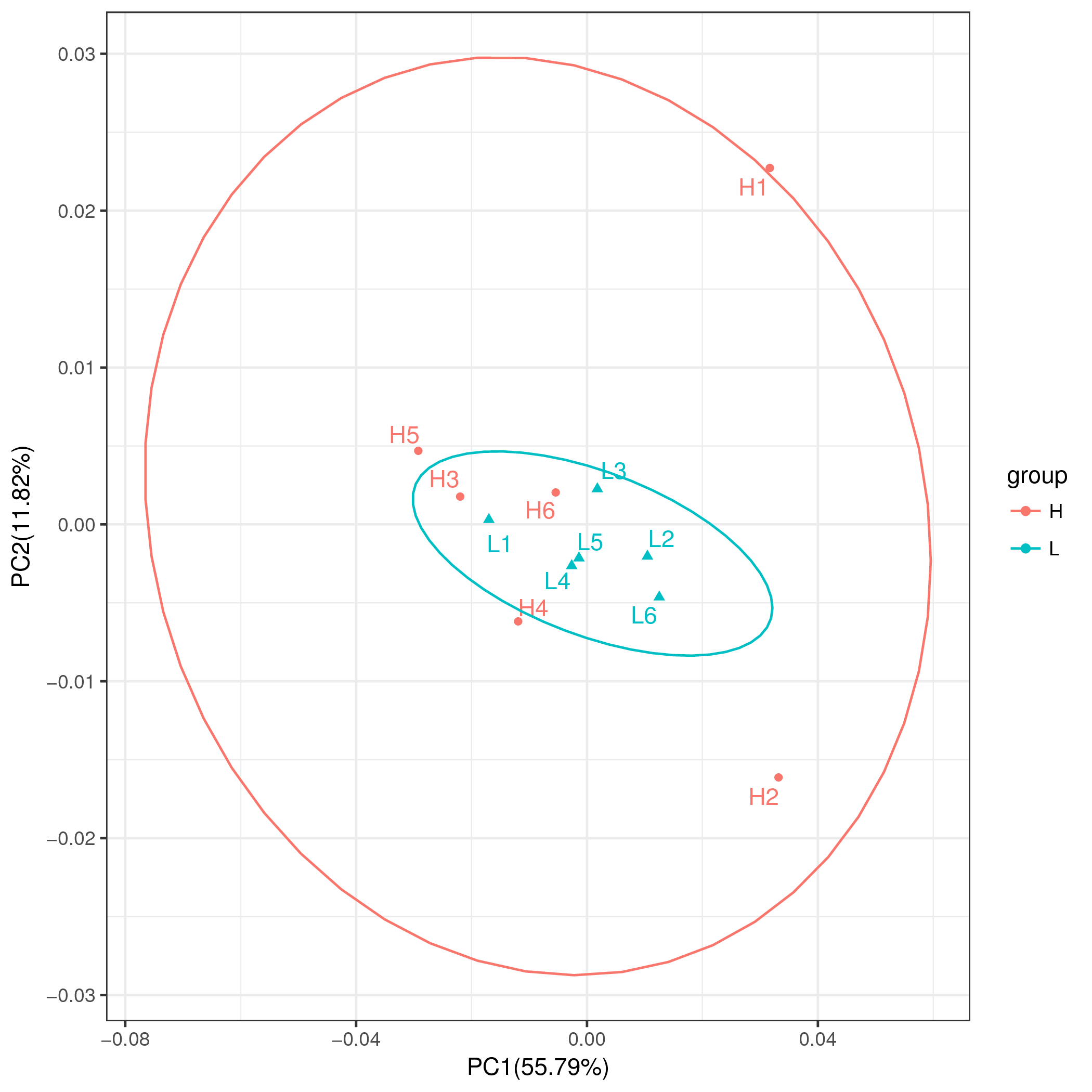

Supplement: Supplemental Information 13 [file peerj-10-14444-s013.zip › Web_Report/Diff_analysis/H_vs_L/H_vs_L_pca.png]

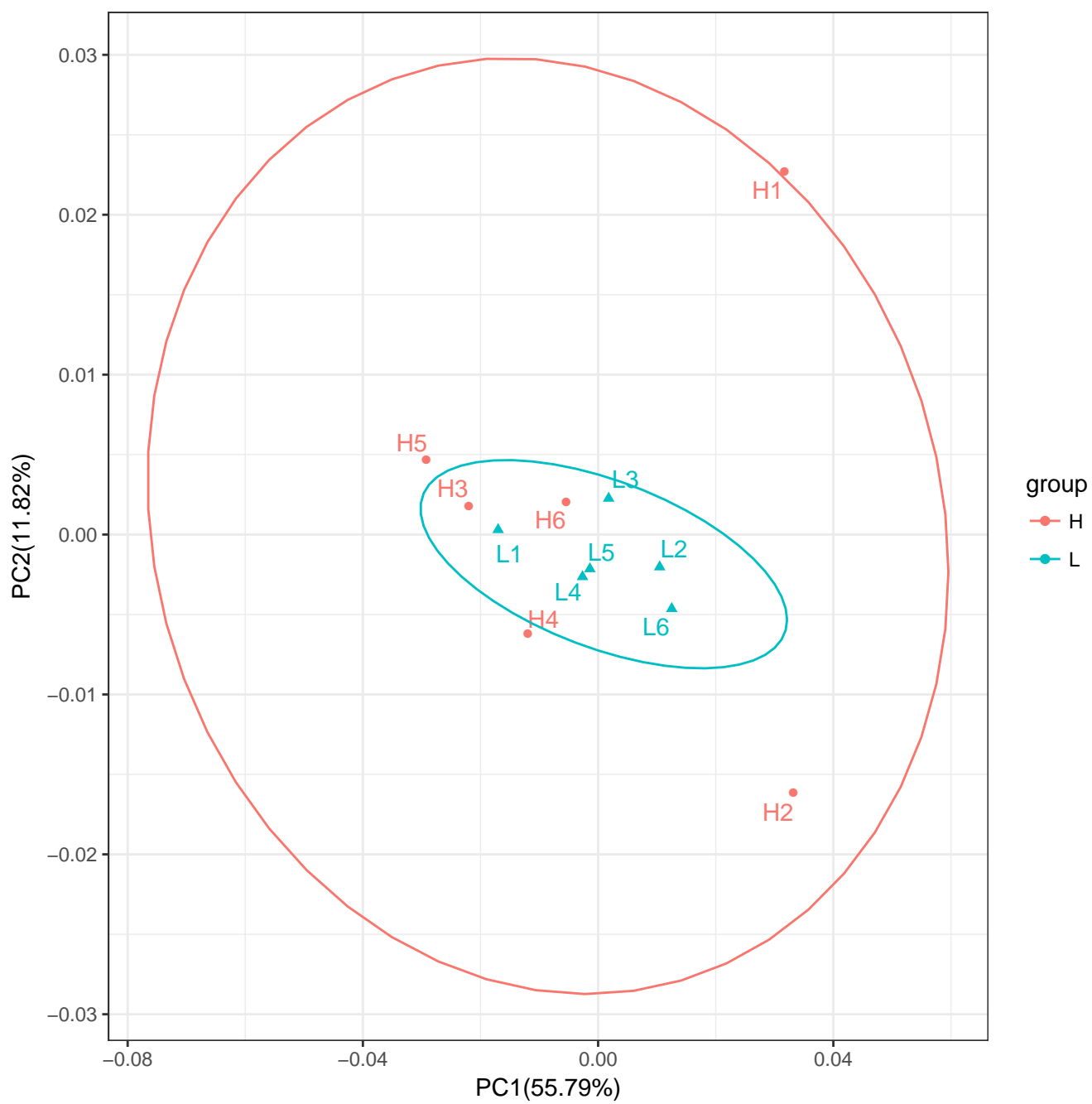

Supplement: Supplemental Information 13 [file peerj-10-14444-s013.zip › Web_Report/Diff_analysis/H_vs_L/H_vs_L_pca.pdf]

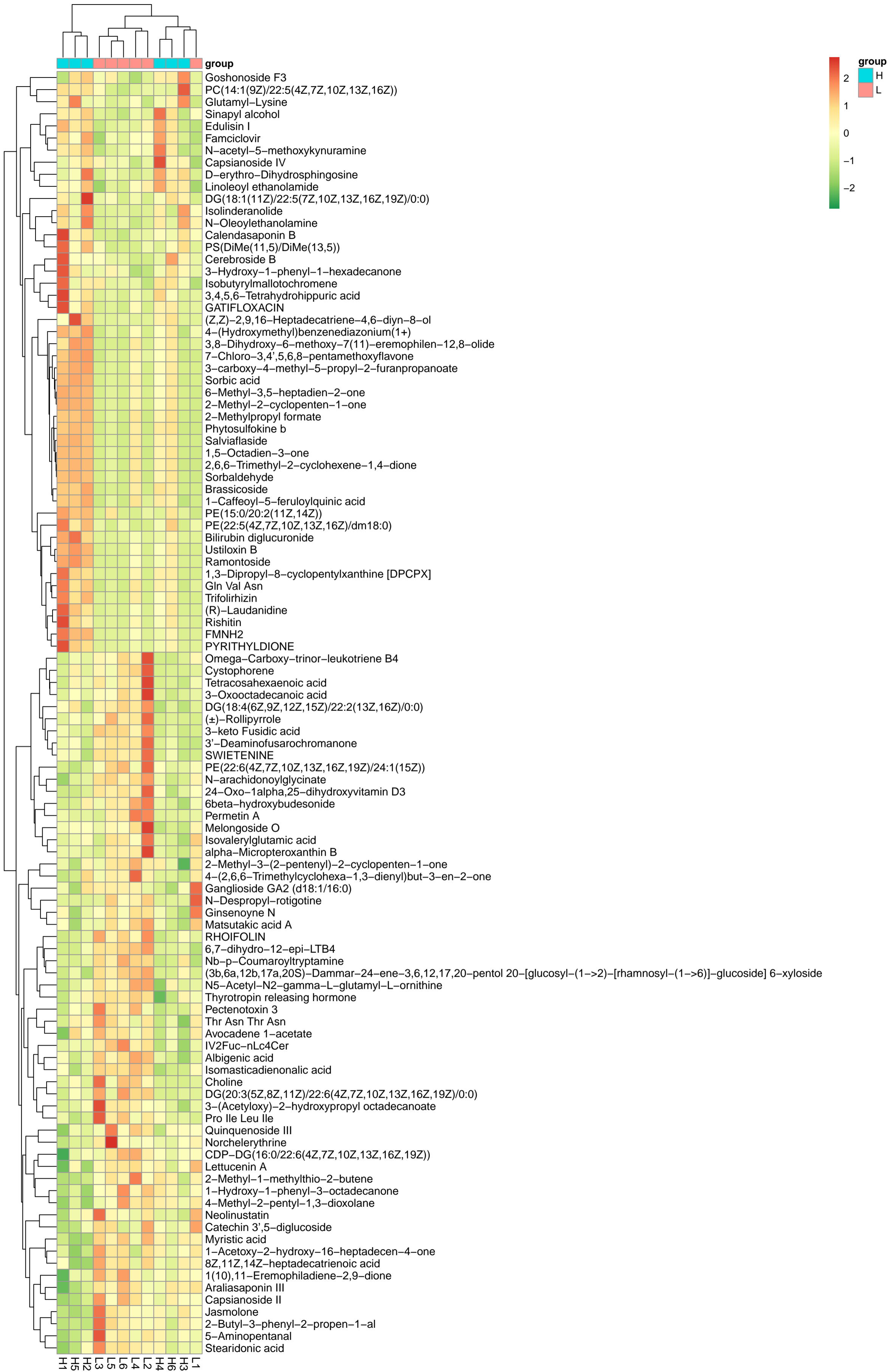

Supplement: Supplemental Information 13 [file peerj-10-14444-s013.zip › Web_Report/Diff_analysis/H_vs_L/H_vs_L_diff_heatmap_name.pdf]

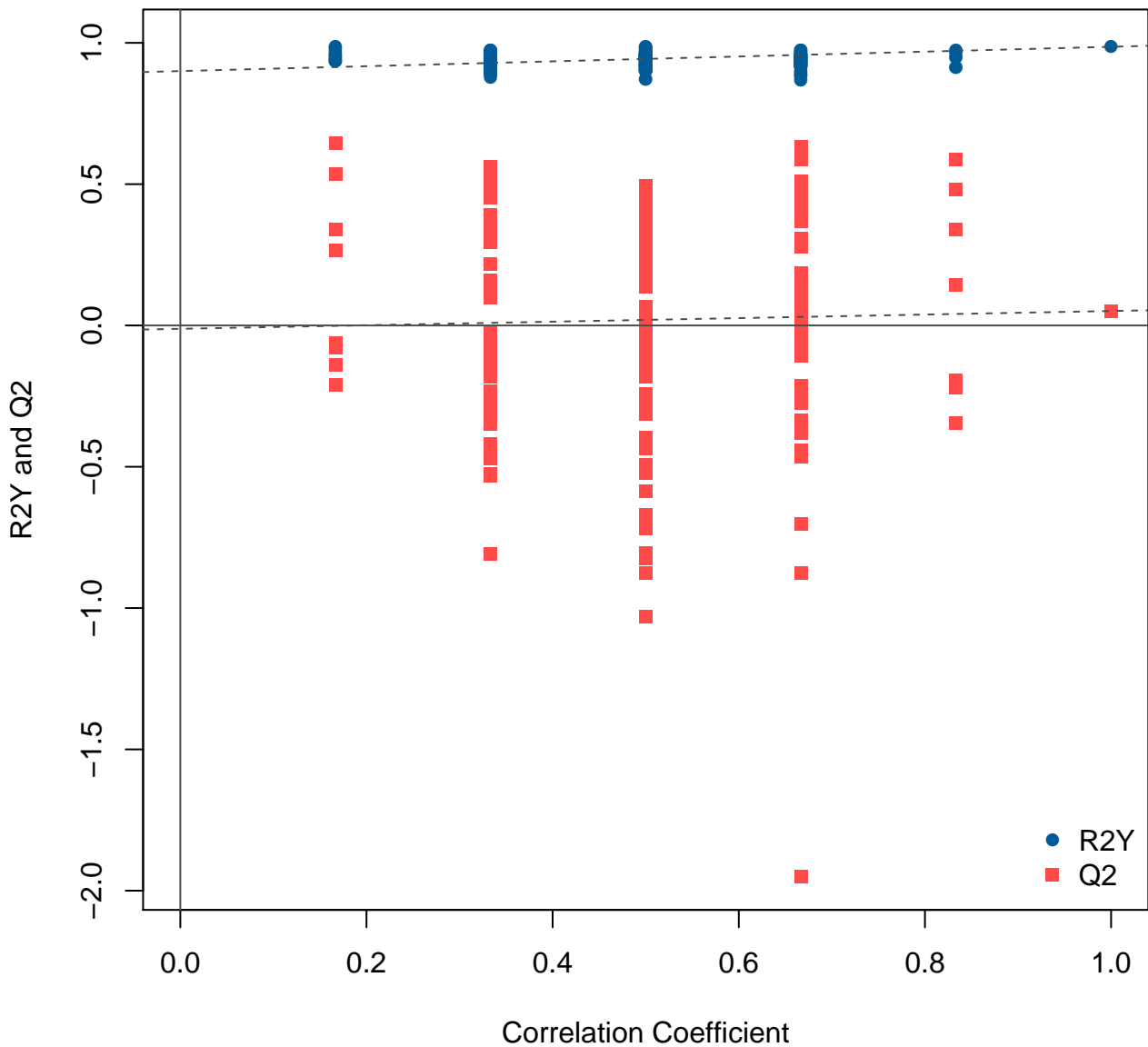

Supplement: Supplemental Information 13 [file peerj-10-14444-s013.zip › Web_Report/Diff_analysis/H_vs_L/H_vs_L_OPLS_DA_permutation.pdf]

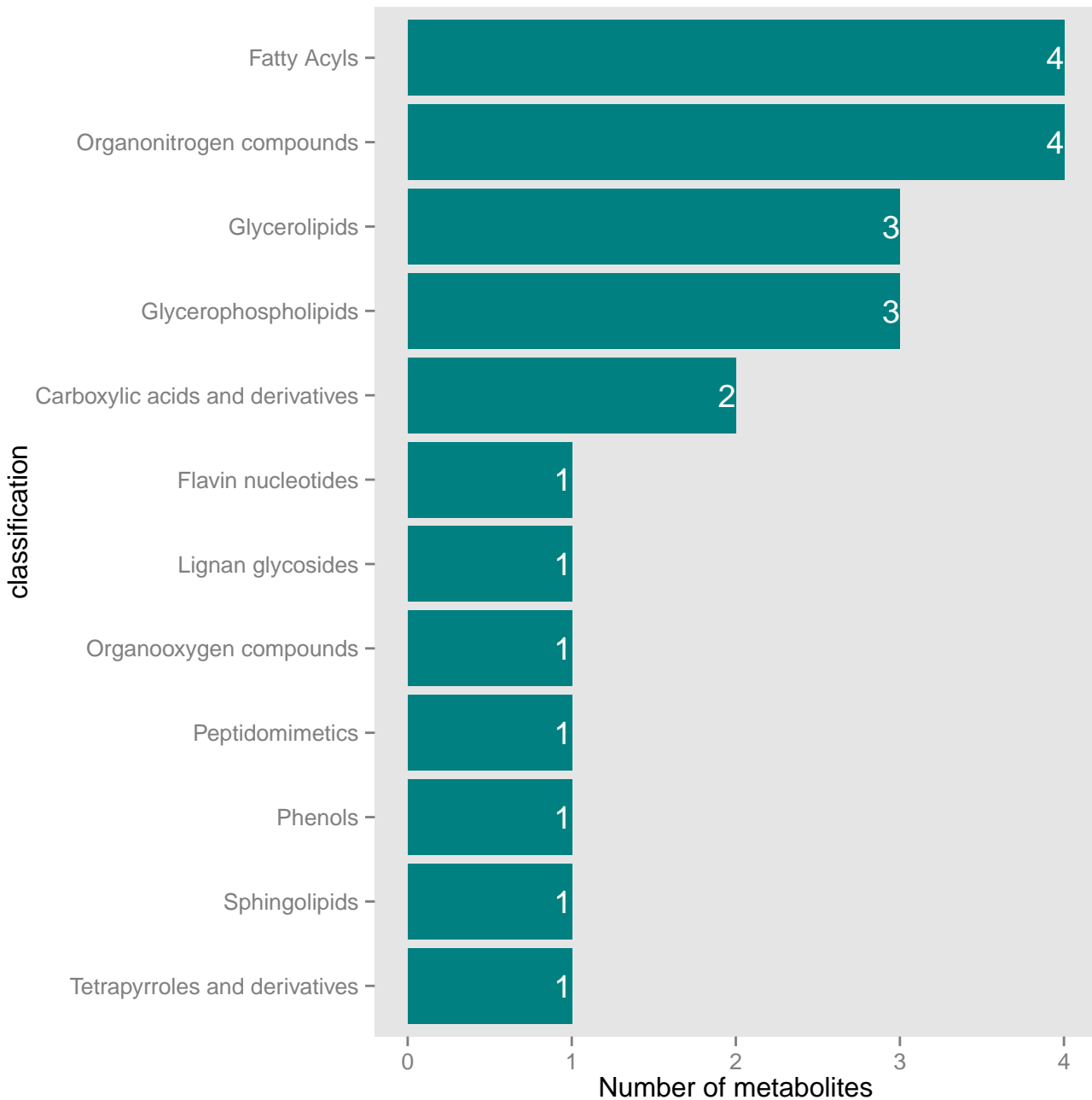

Supplement: Supplemental Information 13 [file peerj-10-14444-s013.zip › Web_Report/Diff_analysis/H_vs_L/H_vs_L_metabolites_classification_top20.pdf]

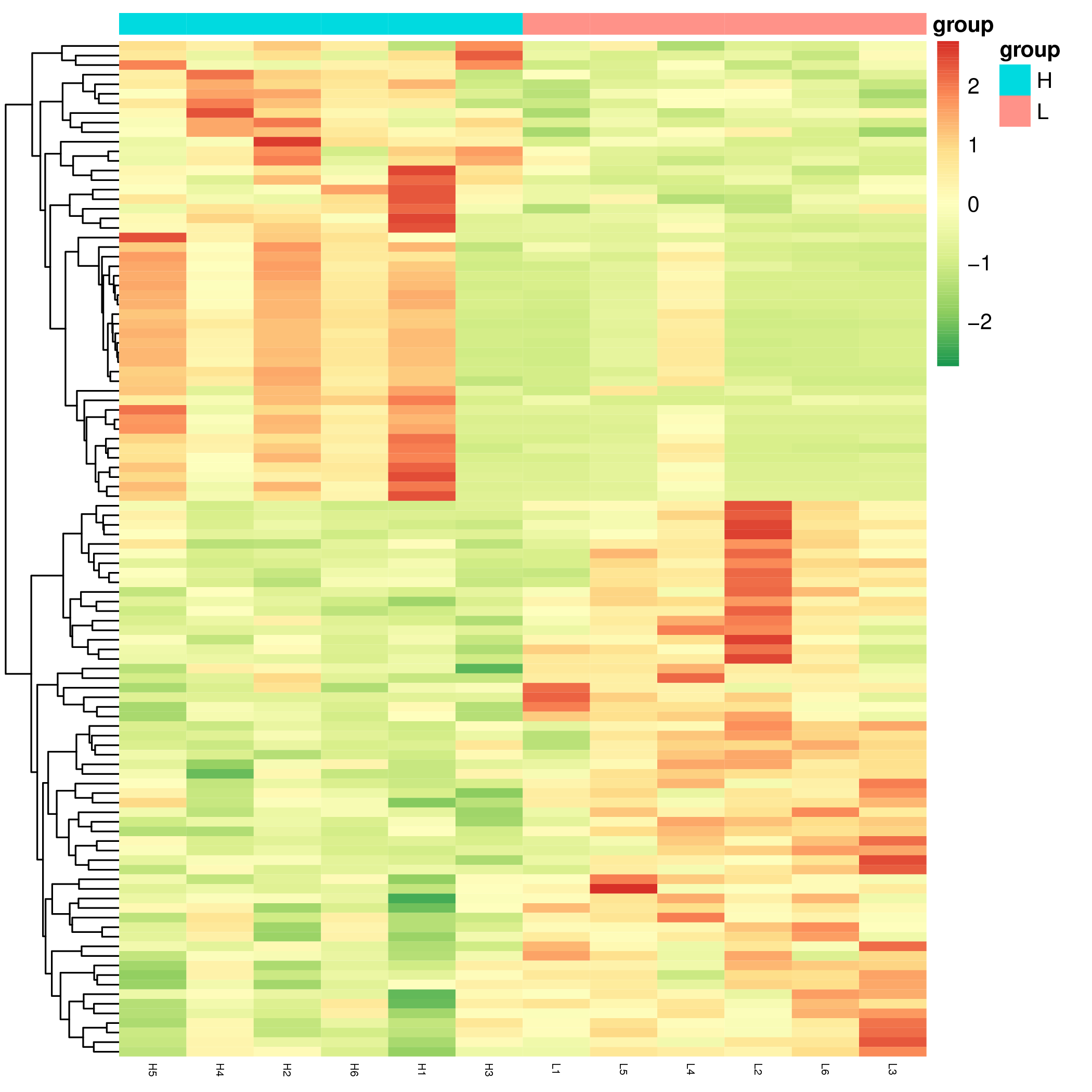

Supplement: Supplemental Information 13 [file peerj-10-14444-s013.zip › Web_Report/Diff_analysis/H_vs_L/H_vs_L_diff_heatmap_nonclustered_samples.png]

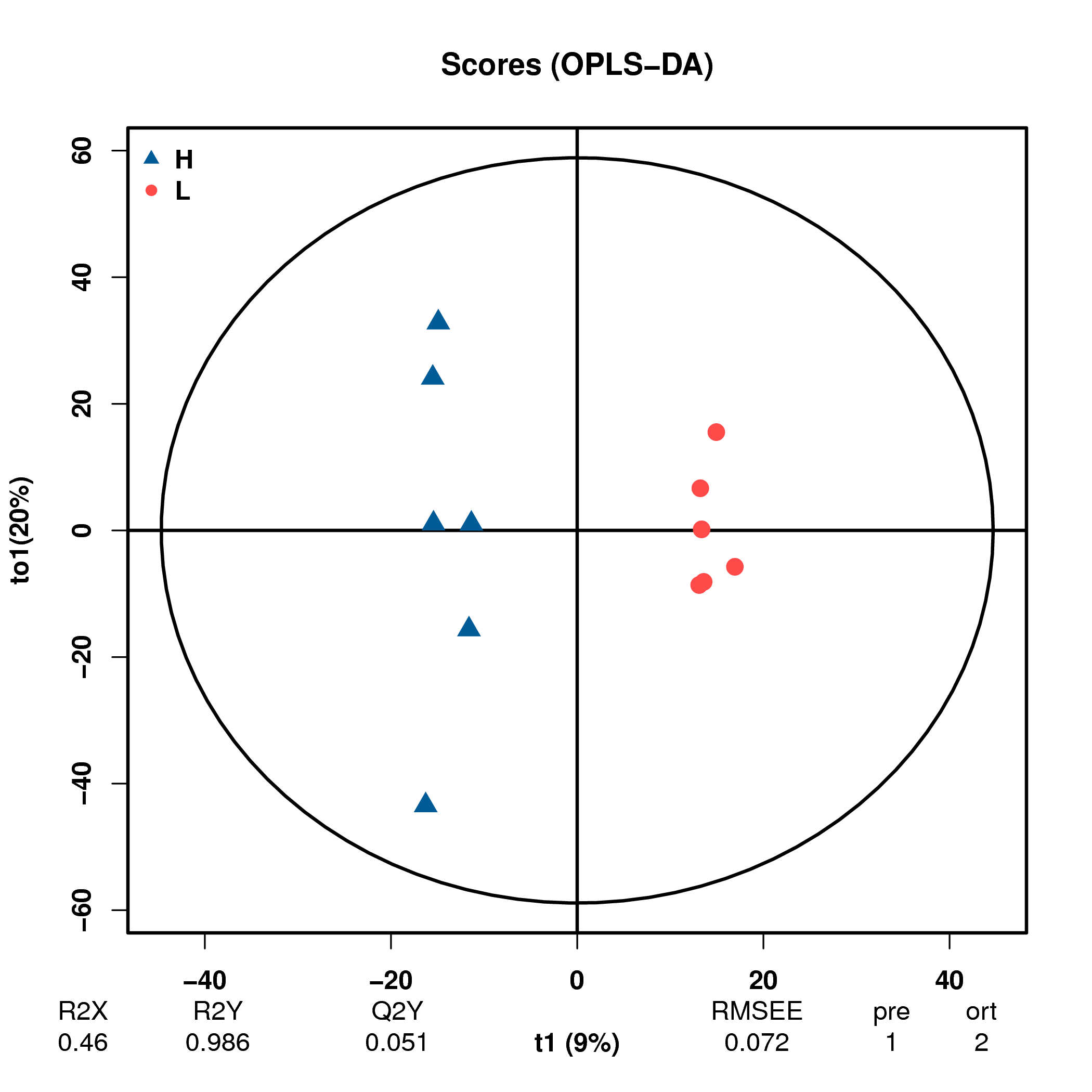

Supplement: Supplemental Information 13 [file peerj-10-14444-s013.zip › Web_Report/Diff_analysis/H_vs_L/H_vs_L_OPLS_DA_nolabel.png]

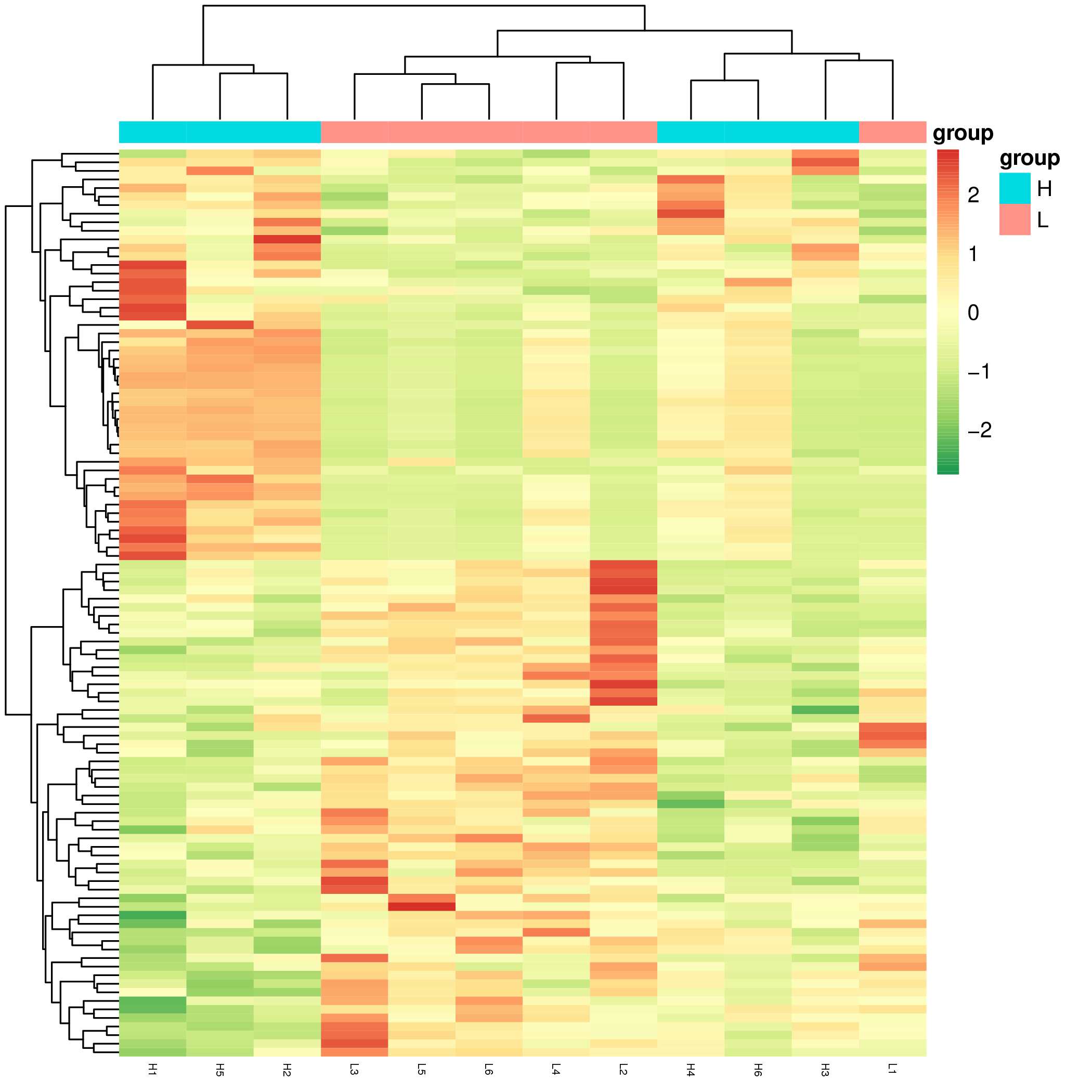

Supplement: Supplemental Information 13 [file peerj-10-14444-s013.zip › Web_Report/Diff_analysis/H_vs_L/H_vs_L_diff_heatmap.png]

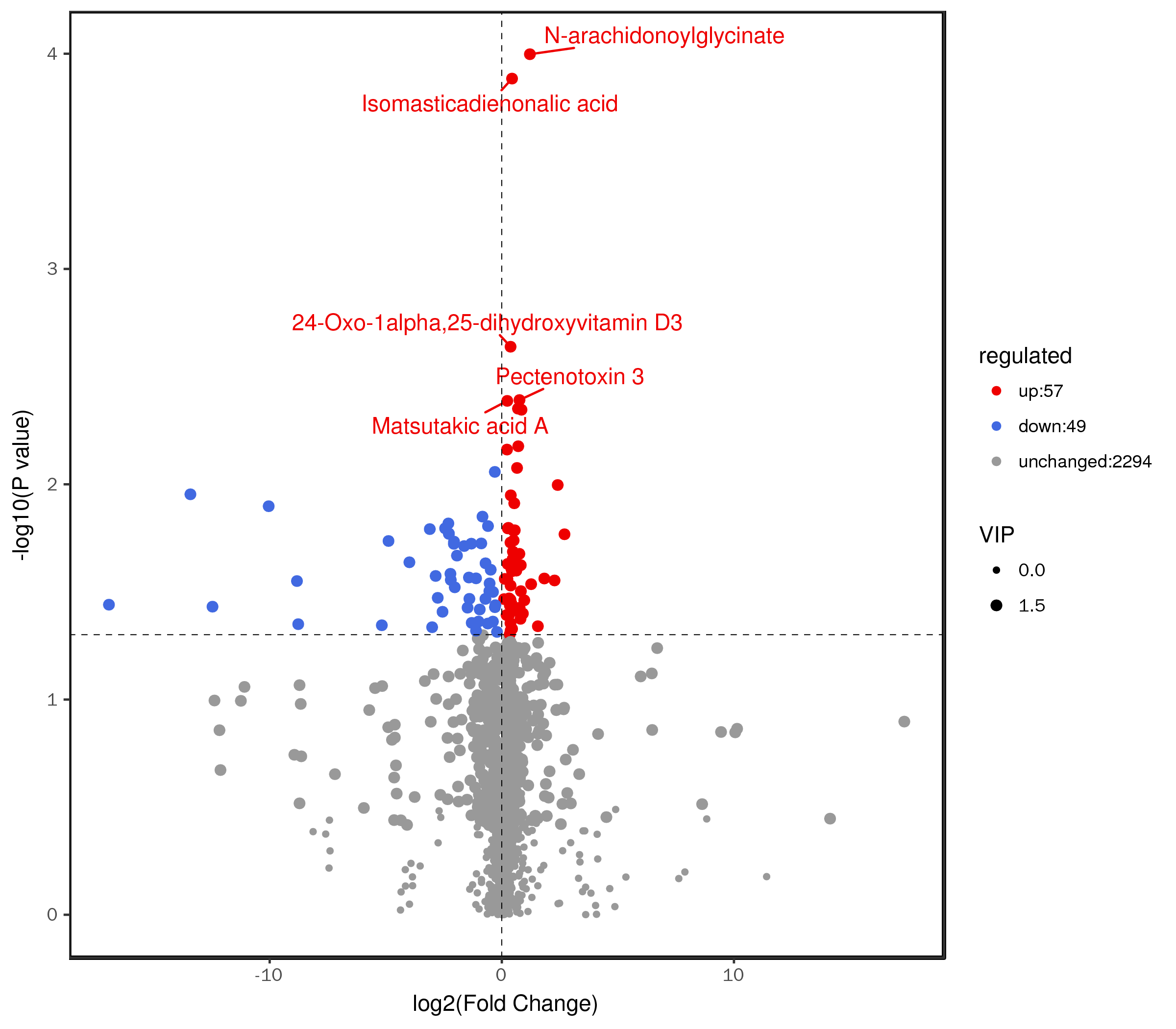

Supplement: Supplemental Information 13 [file peerj-10-14444-s013.zip › Web_Report/Diff_analysis/H_vs_L/H_vs_L.volcano.png]

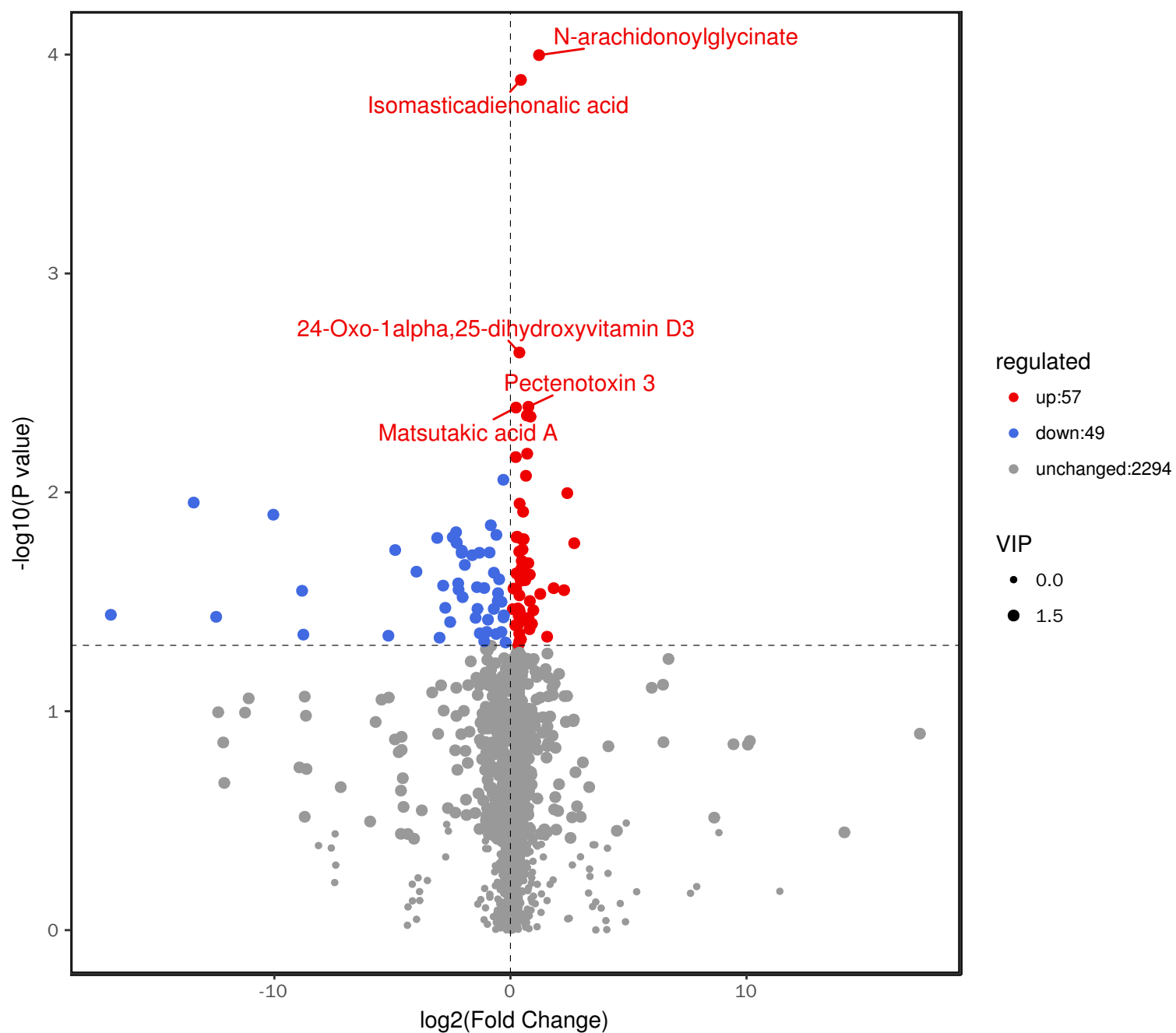

Supplement: Supplemental Information 13 [file peerj-10-14444-s013.zip › Web_Report/Diff_analysis/H_vs_L/H_vs_L.volcano.pdf]

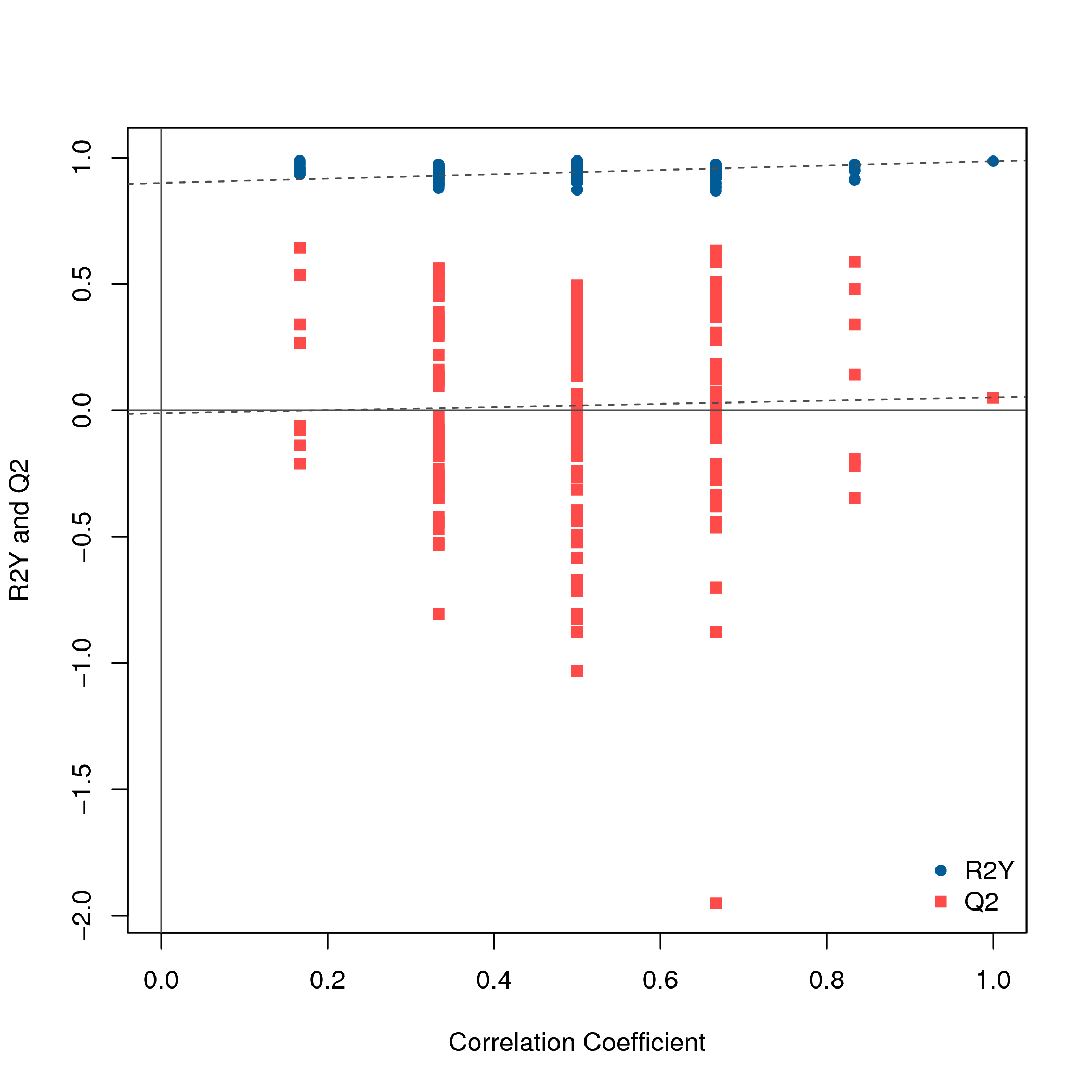

Supplement: Supplemental Information 13 [file peerj-10-14444-s013.zip › Web_Report/Diff_analysis/H_vs_L/H_vs_L_OPLS_DA_permutation.png]

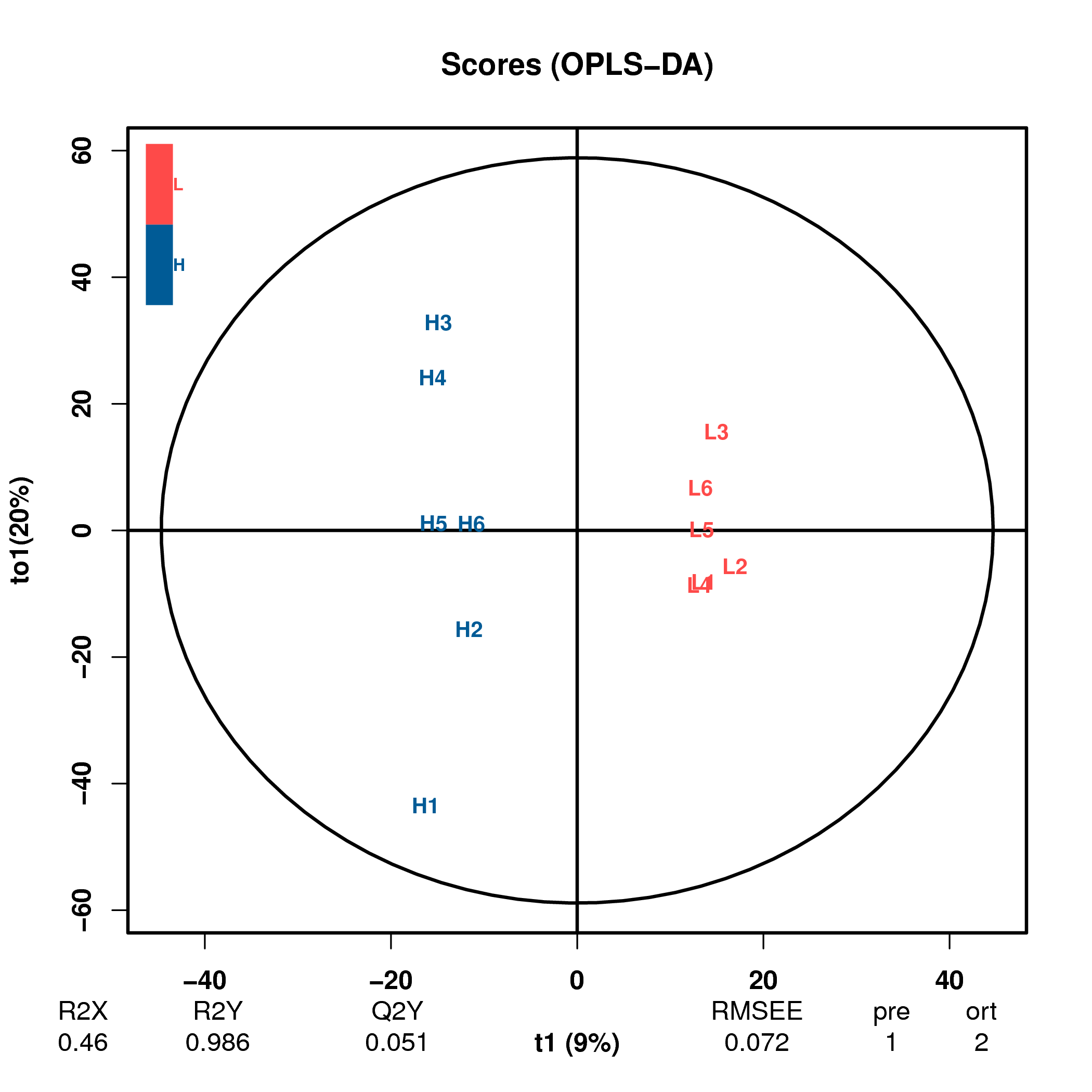

Supplement: Supplemental Information 13 [file peerj-10-14444-s013.zip › Web_Report/Diff_analysis/H_vs_L/H_vs_L_OPLS_DA.png]

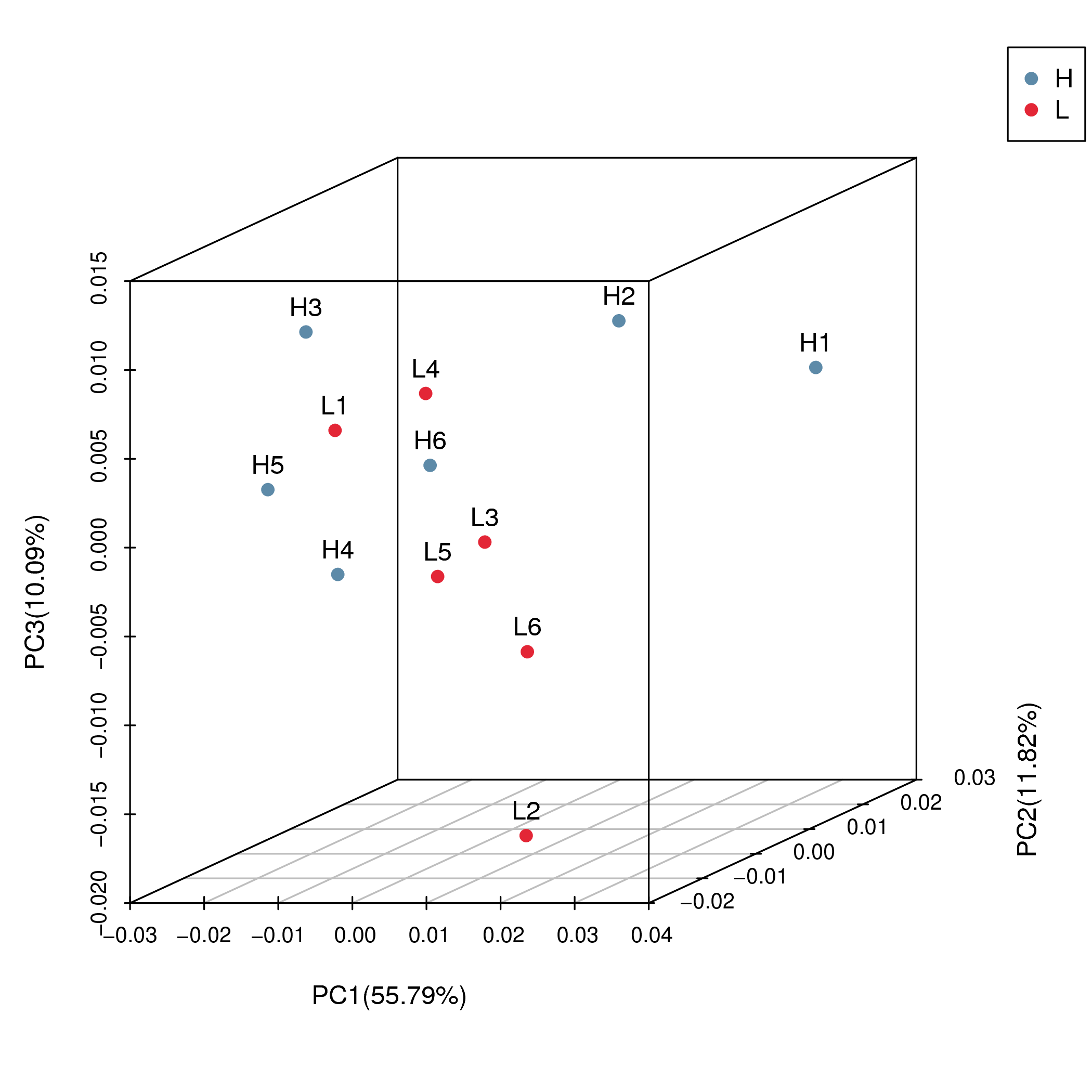

Supplement: Supplemental Information 13 [file peerj-10-14444-s013.zip › Web_Report/Diff_analysis/H_vs_L/H_vs_L_pca3D.png]

# Scores (OPLS-DA)

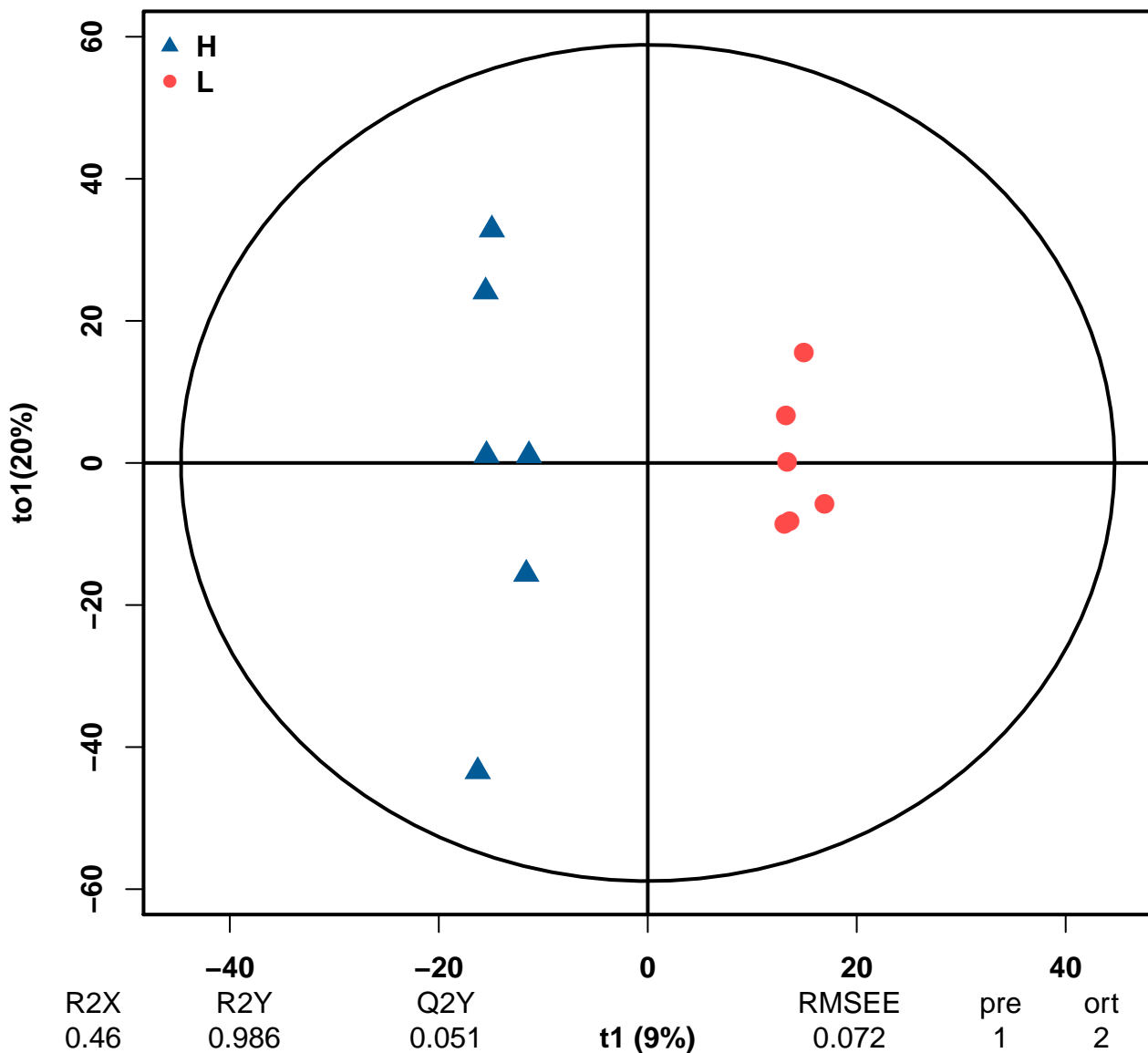

Supplement: Supplemental Information 13 [file peerj-10-14444-s013.zip › Web_Report/Diff_analysis/H_vs_L/H_vs_L_OPLS_DA_nolabel.pdf]

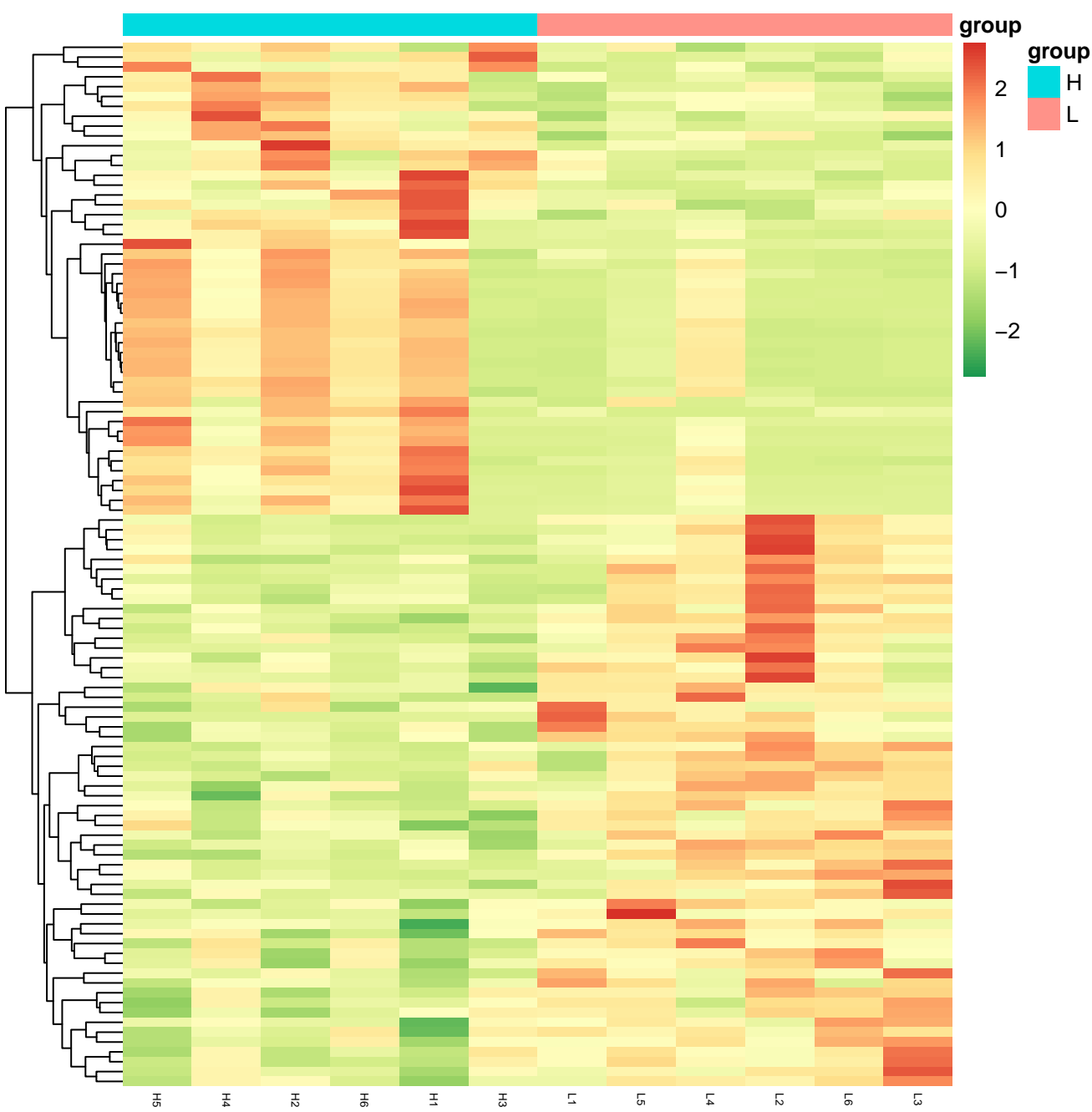

Supplement: Supplemental Information 13 [file peerj-10-14444-s013.zip › Web_Report/Diff_analysis/H_vs_L/H_vs_L_diff_heatmap_nonclustered_samples.pdf]

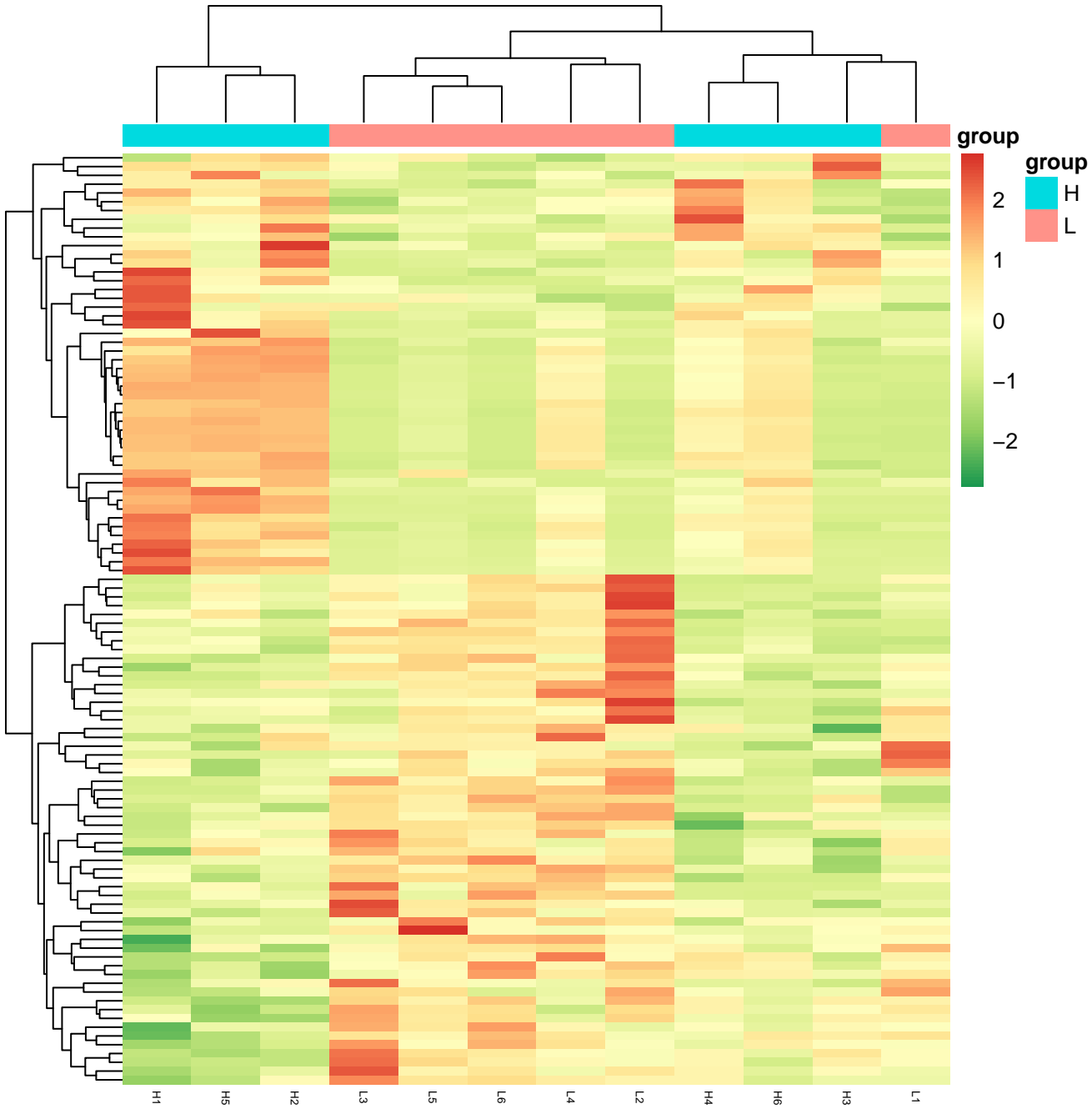

Supplement: Supplemental Information 13 [file peerj-10-14444-s013.zip › Web_Report/Diff_analysis/H_vs_L/H_vs_L_diff_heatmap.pdf]

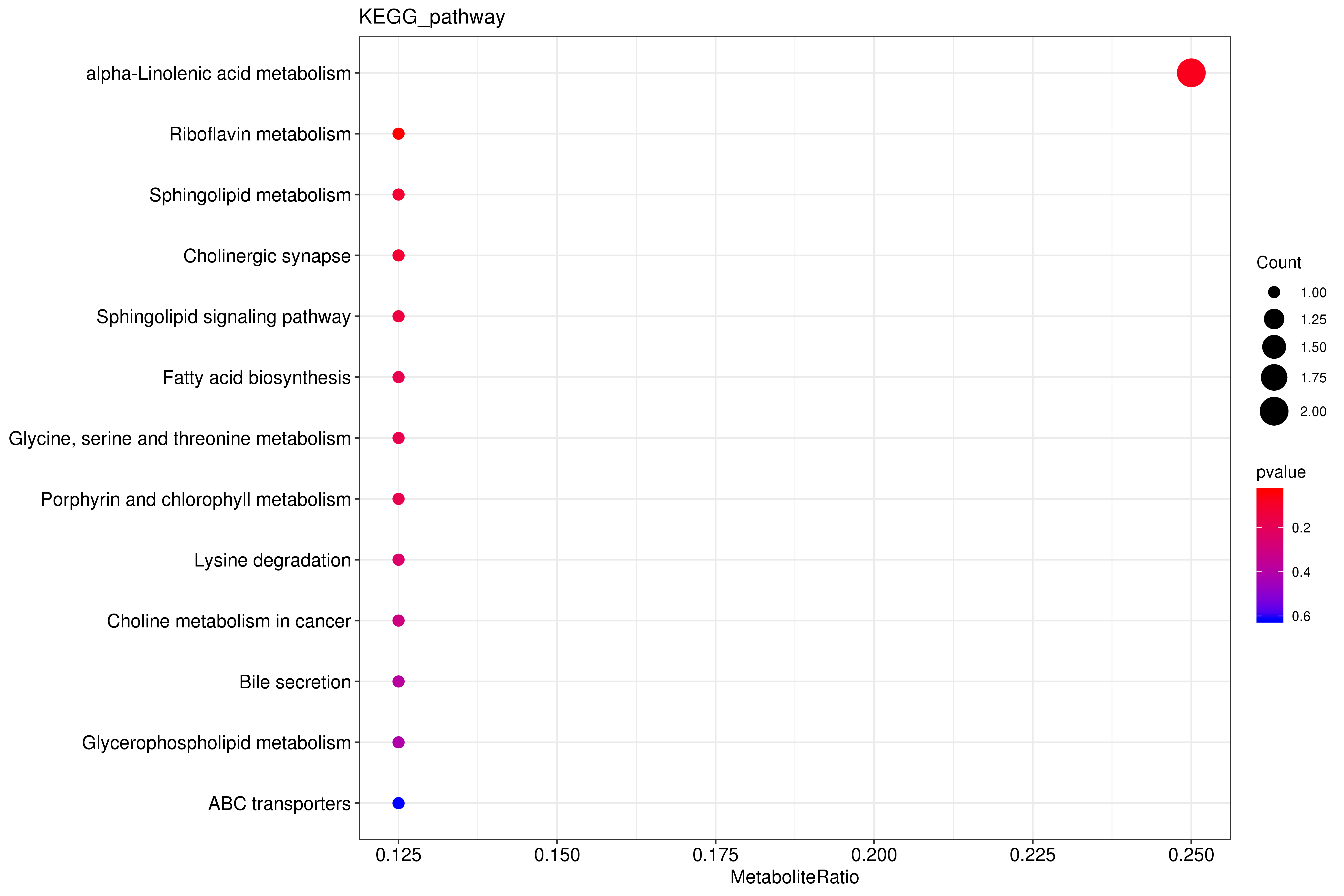

Supplement: Supplemental Information 13 [file peerj-10-14444-s013.zip › Web_Report/Diff_analysis/H_vs_L/KEGG/Graph/H_vs_L_KEGG_pathway_enrich_dotplot.png]

## KEGG\_pathway

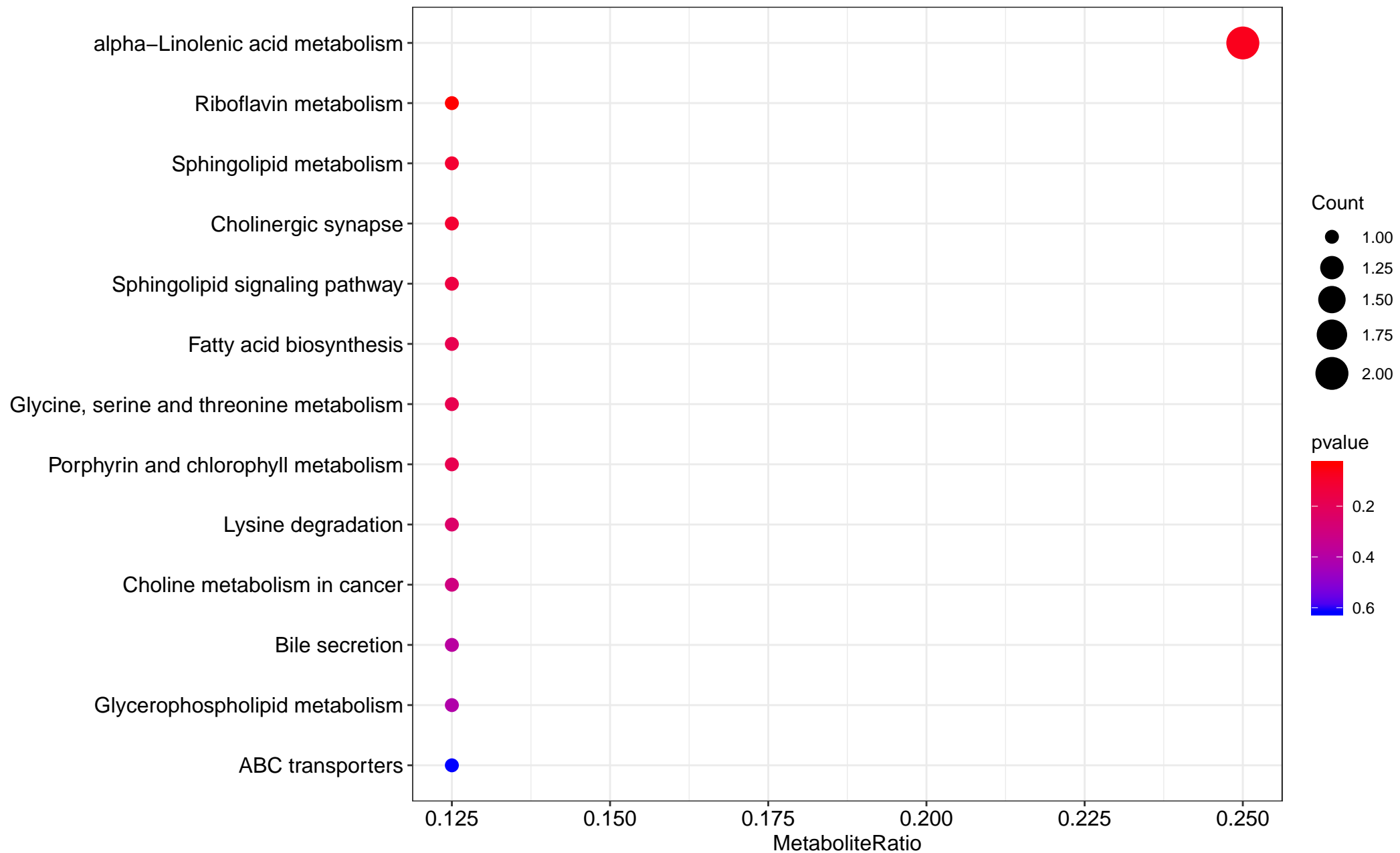

Supplement: Supplemental Information 13 [file peerj-10-14444-s013.zip › Web_Report/Diff_analysis/H_vs_L/KEGG/Graph/H_vs_L_KEGG_pathway_enrich_dotplot.pdf]

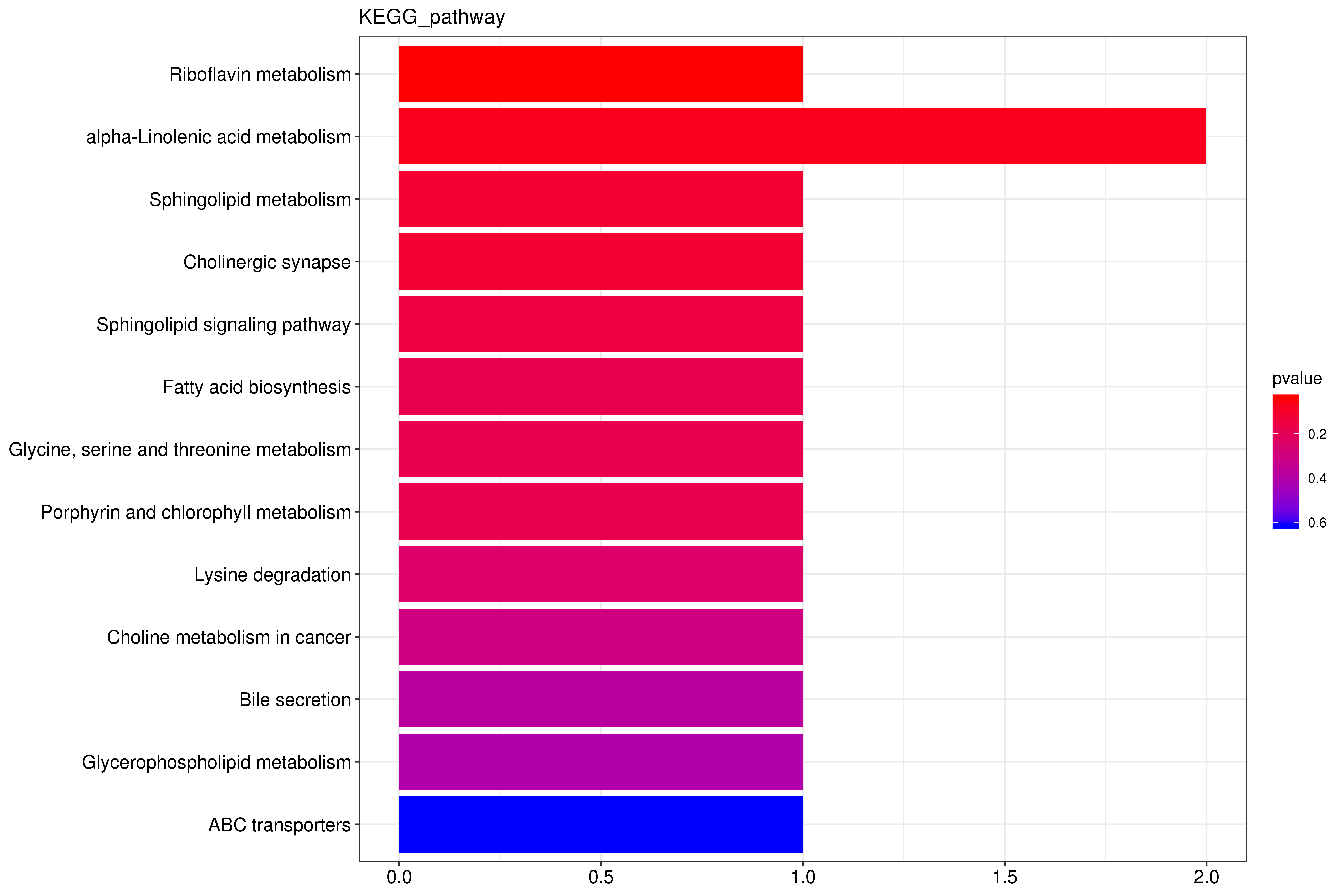

Supplement: Supplemental Information 13 [file peerj-10-14444-s013.zip › Web_Report/Diff_analysis/H_vs_L/KEGG/Graph/H_vs_L_KEGG_pathway_enrich_barplot.png]

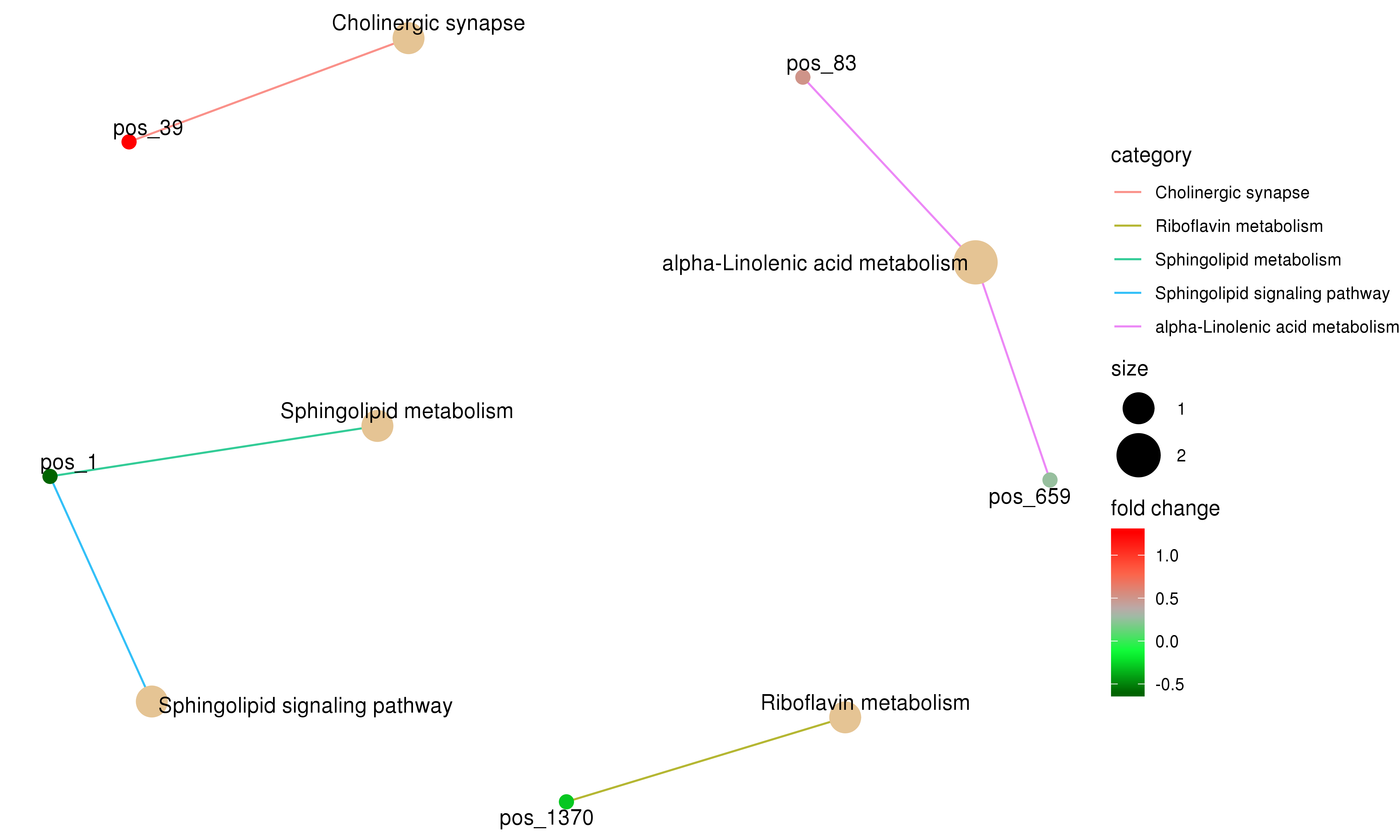

Supplement: Supplemental Information 13 [file peerj-10-14444-s013.zip › Web_Report/Diff_analysis/H_vs_L/KEGG/Graph/H_vs_L_KEGG_pathway_enrich_cnetplot.png]

## KEGG\_pathway

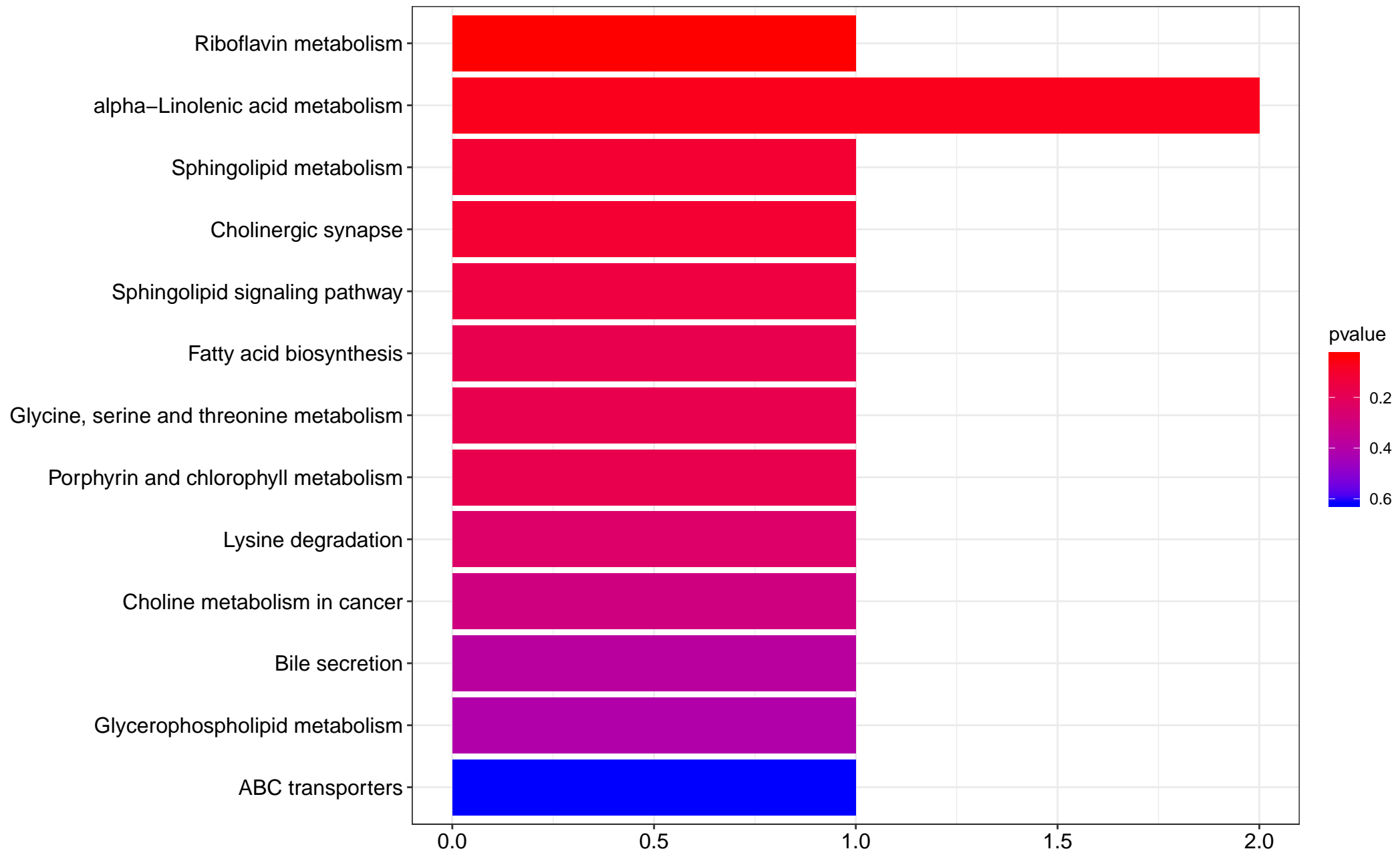

Supplement: Supplemental Information 13 [file peerj-10-14444-s013.zip › Web_Report/Diff_analysis/H_vs_L/KEGG/Graph/H_vs_L_KEGG_pathway_enrich_barplot.pdf]

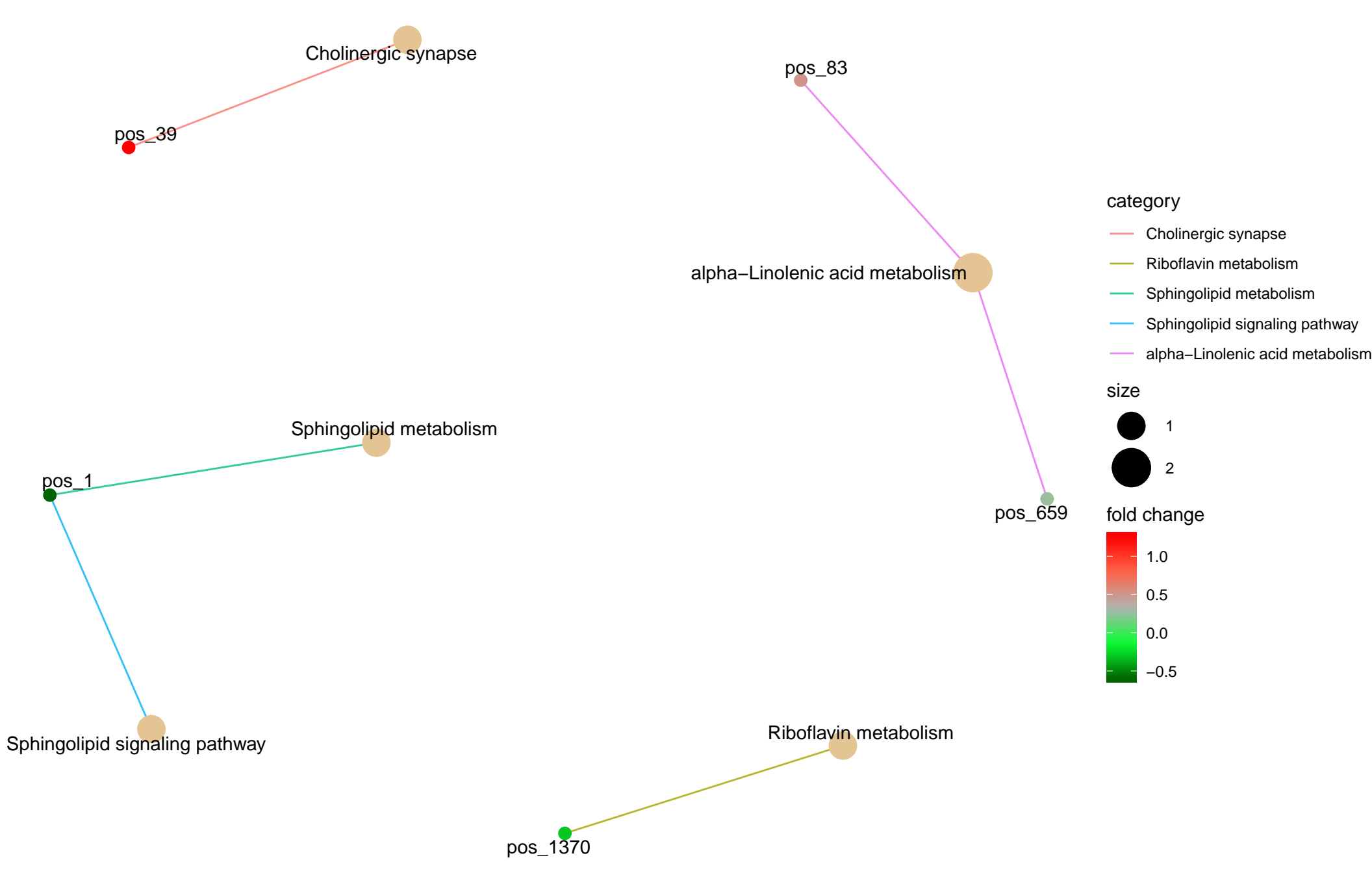

Supplement: Supplemental Information 13 [file peerj-10-14444-s013.zip › Web_Report/Diff_analysis/H_vs_L/KEGG/Graph/H_vs_L_KEGG_pathway_enrich_cnetplot.pdf]

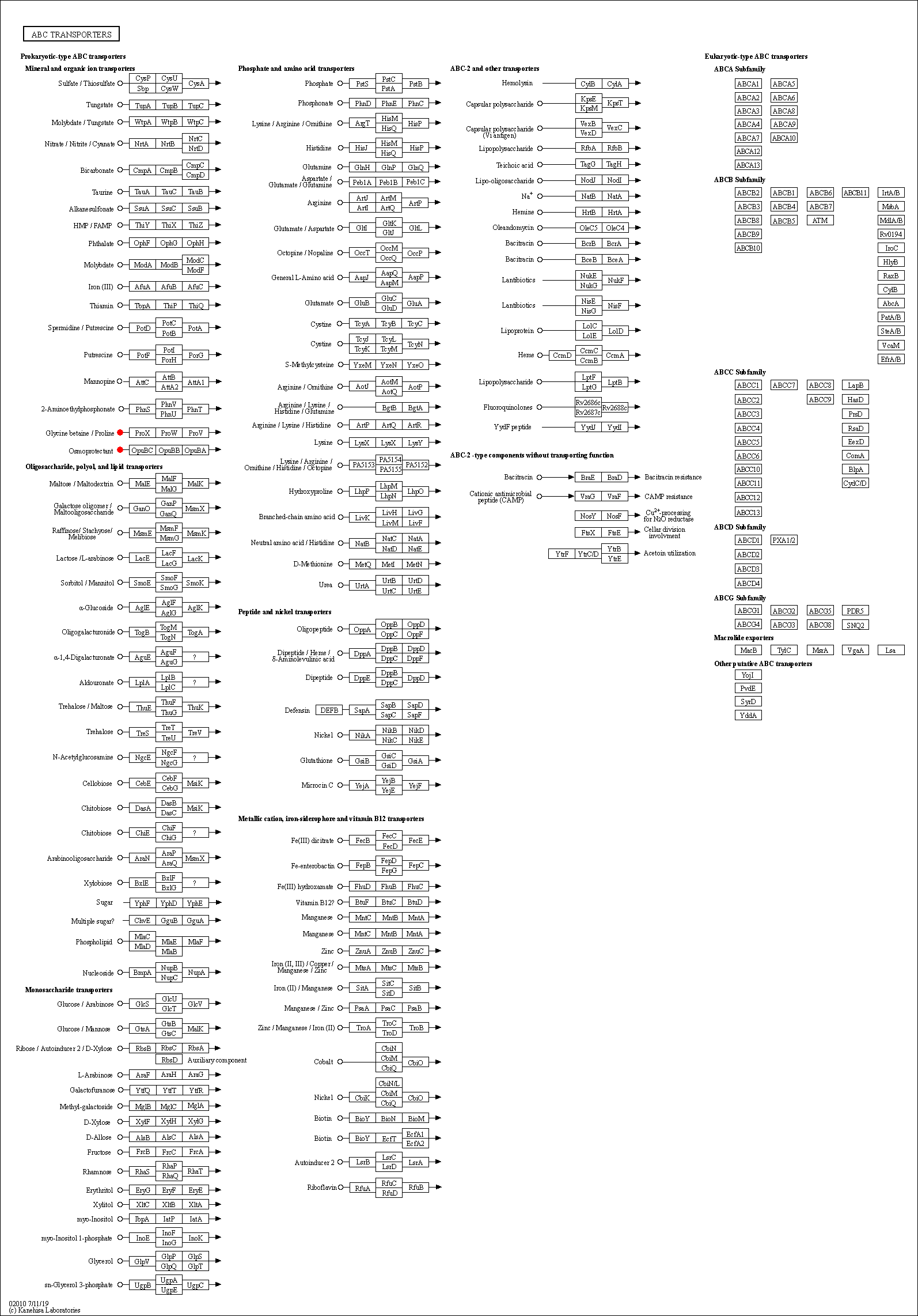

Supplement: Supplemental Information 13 [file peerj-10-14444-s013.zip › Web_Report/Diff_analysis/H_vs_L/KEGG/kegg_map/ko02010.png]

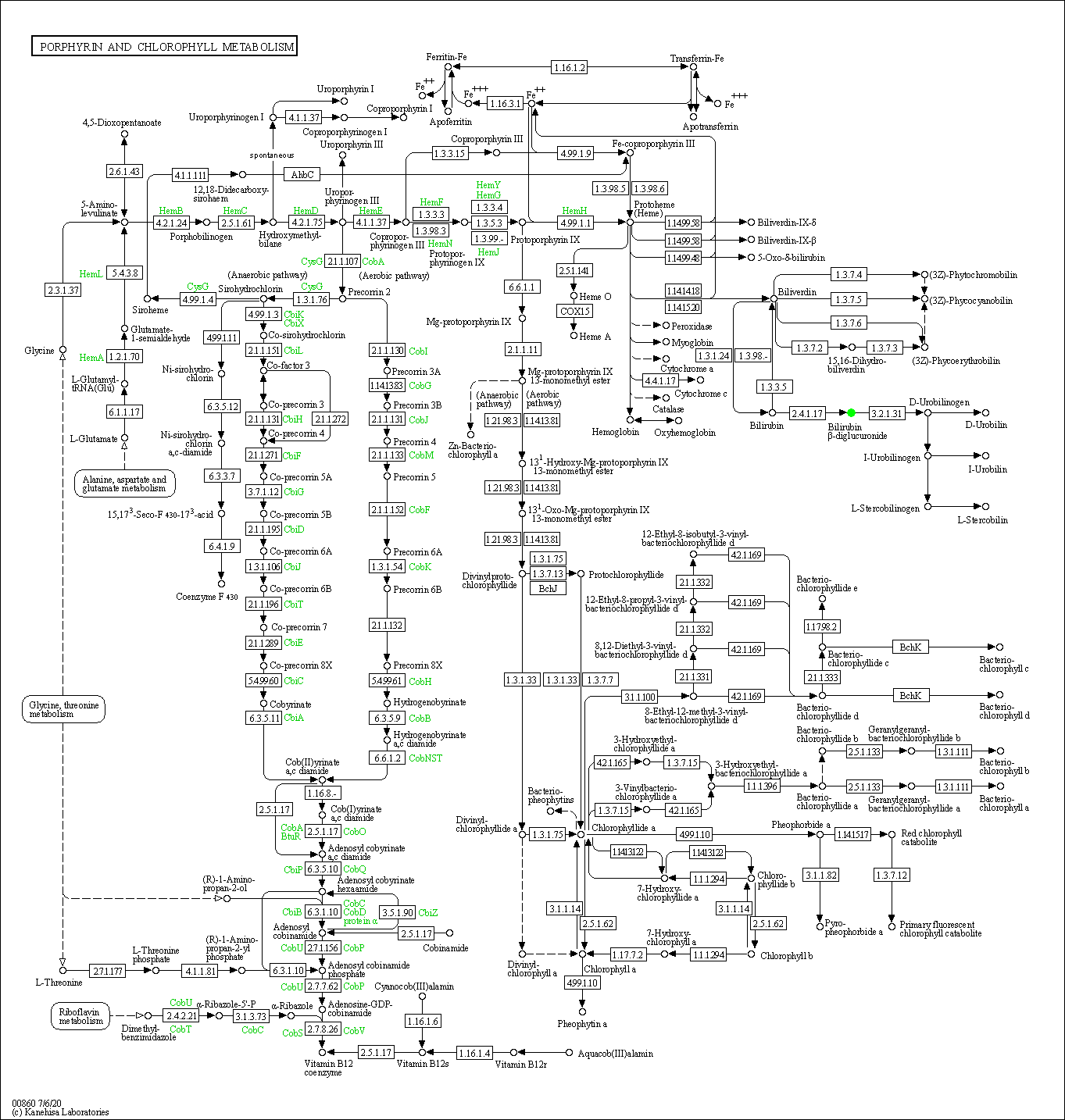

Supplement: Supplemental Information 13 [file peerj-10-14444-s013.zip › Web_Report/Diff_analysis/H_vs_L/KEGG/kegg_map/ko00860.png]

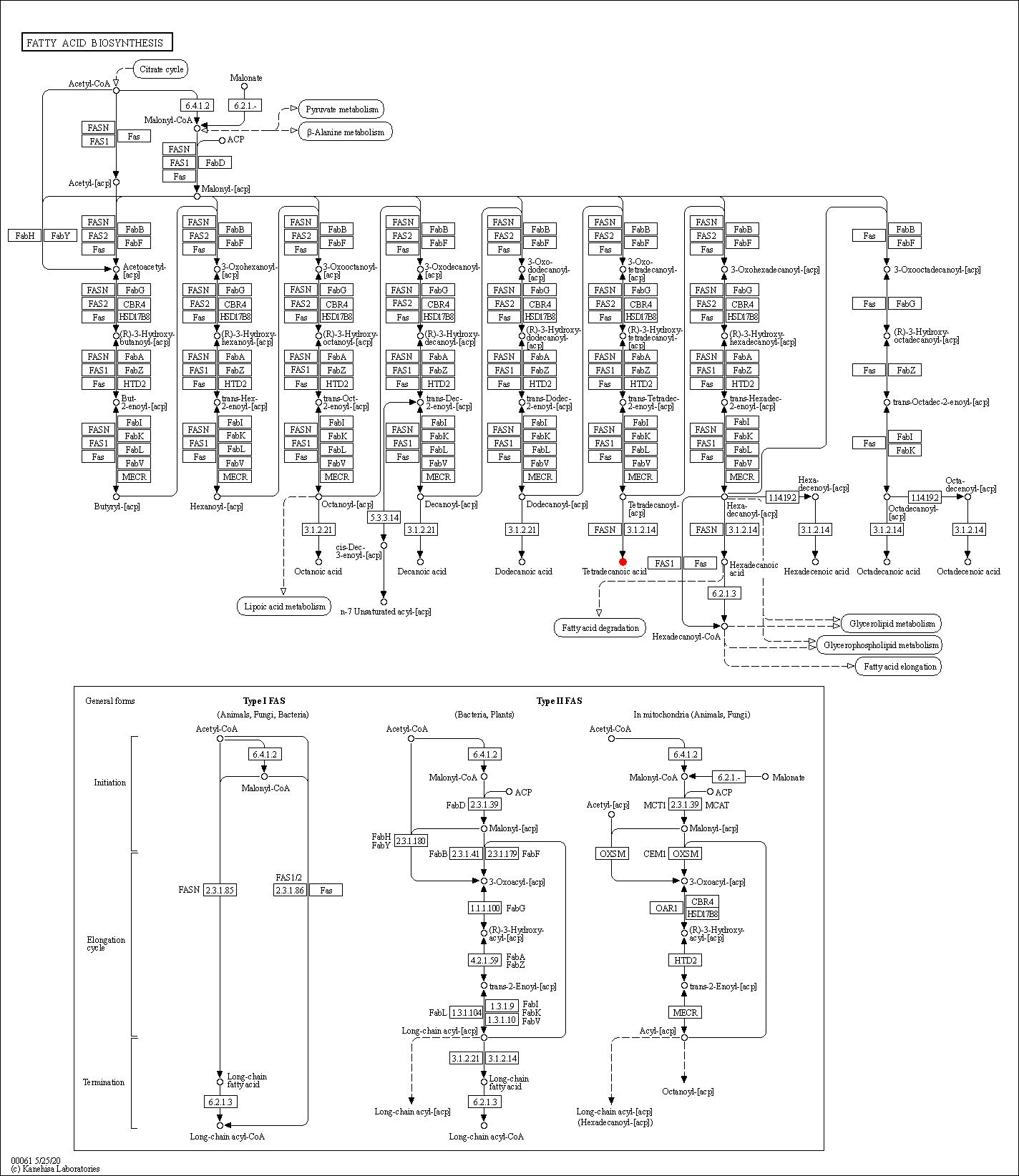

Supplement: Supplemental Information 13 [file peerj-10-14444-s013.zip › Web_Report/Diff_analysis/H_vs_L/KEGG/kegg_map/ko00061.png]

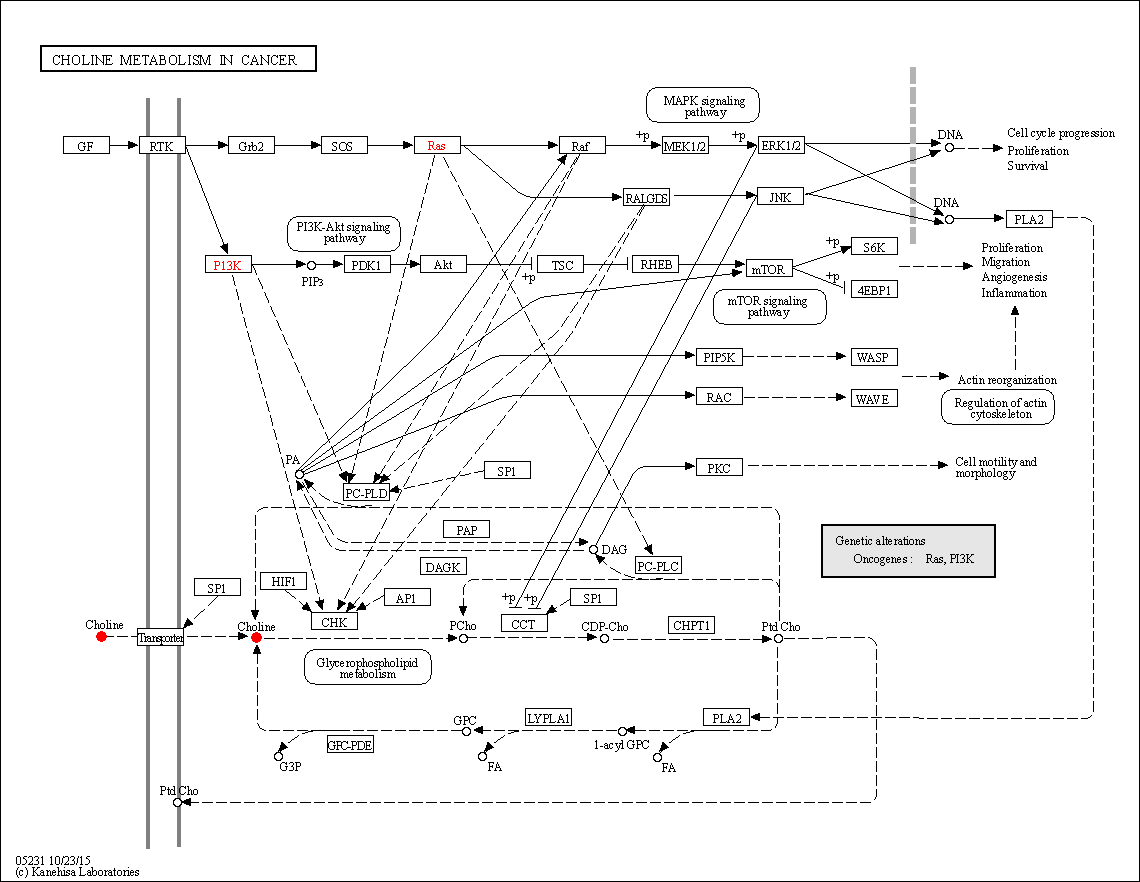

Supplement: Supplemental Information 13 [file peerj-10-14444-s013.zip › Web_Report/Diff_analysis/H_vs_L/KEGG/kegg_map/ko05231.png]

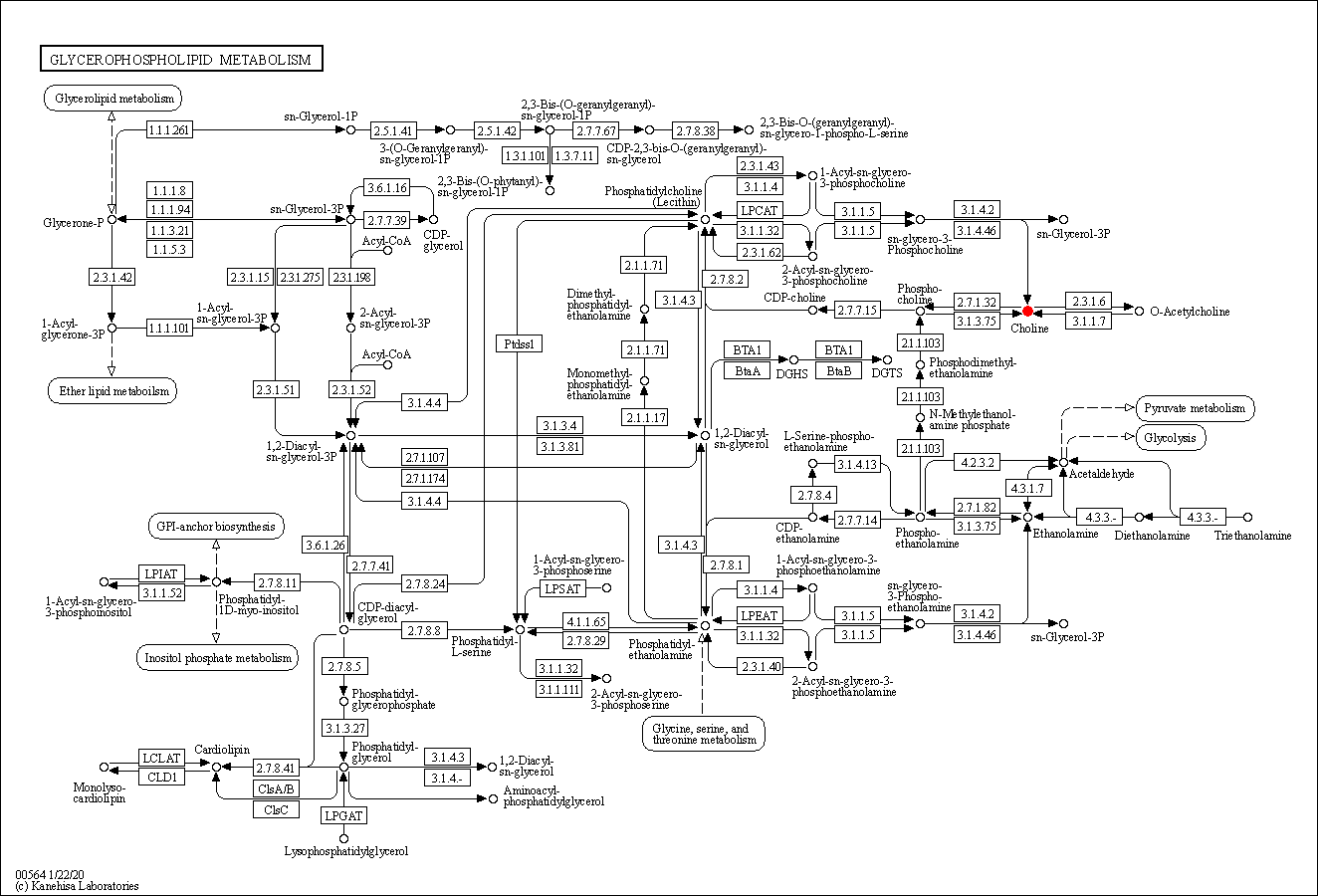

Supplement: Supplemental Information 13 [file peerj-10-14444-s013.zip › Web_Report/Diff_analysis/H_vs_L/KEGG/kegg_map/ko00564.png]

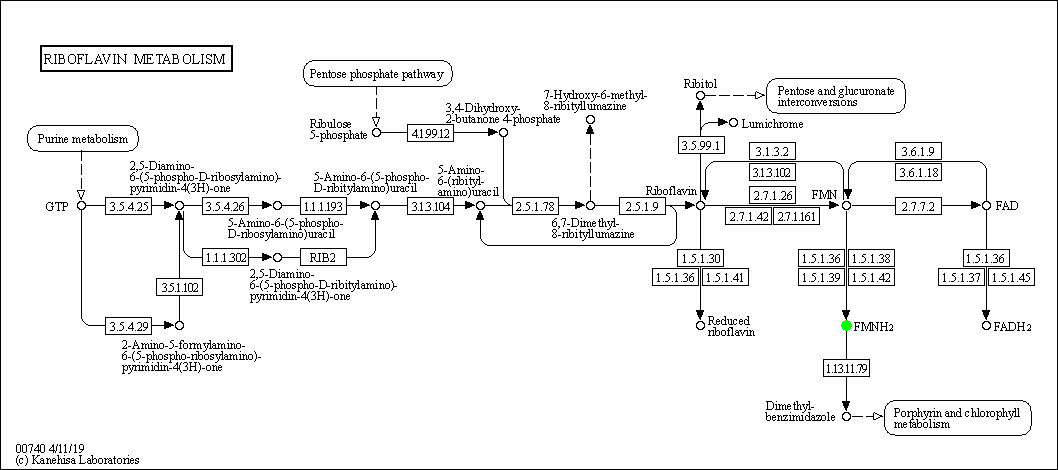

Supplement: Supplemental Information 13 [file peerj-10-14444-s013.zip › Web_Report/Diff_analysis/H_vs_L/KEGG/kegg_map/ko00740.png]

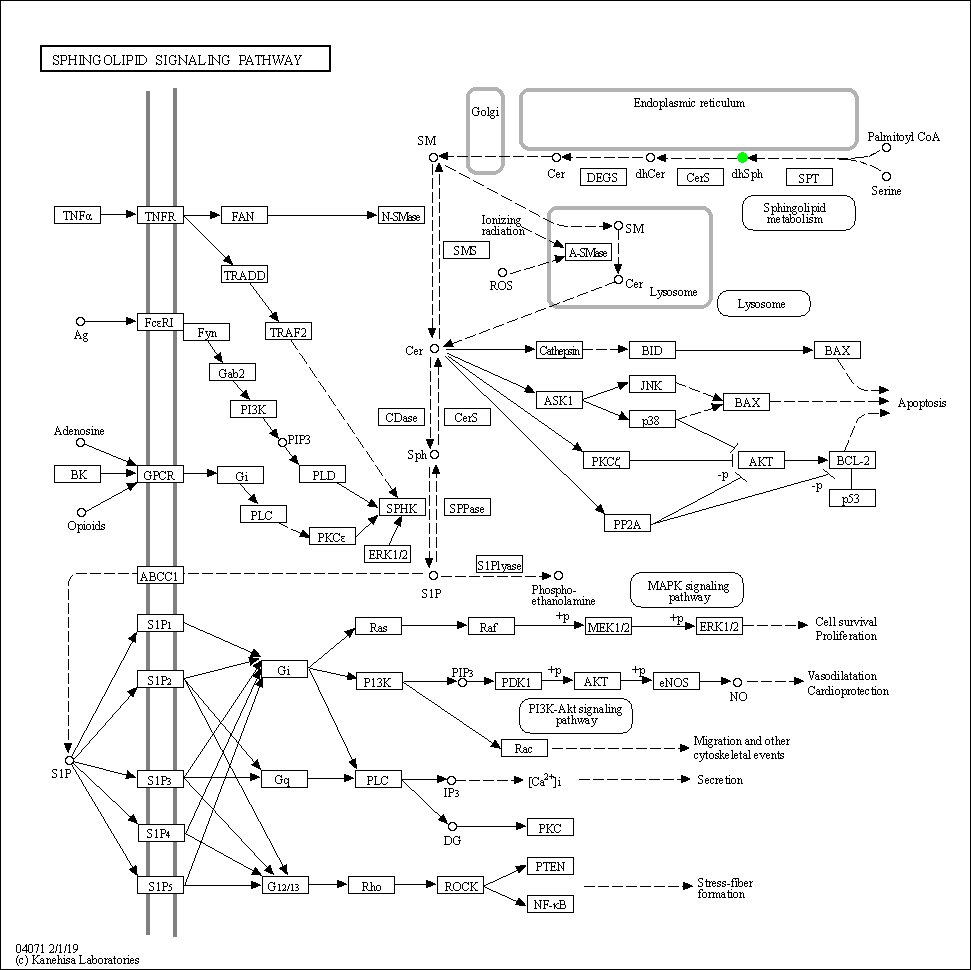

Supplement: Supplemental Information 13 [file peerj-10-14444-s013.zip › Web_Report/Diff_analysis/H_vs_L/KEGG/kegg_map/ko04071.png]

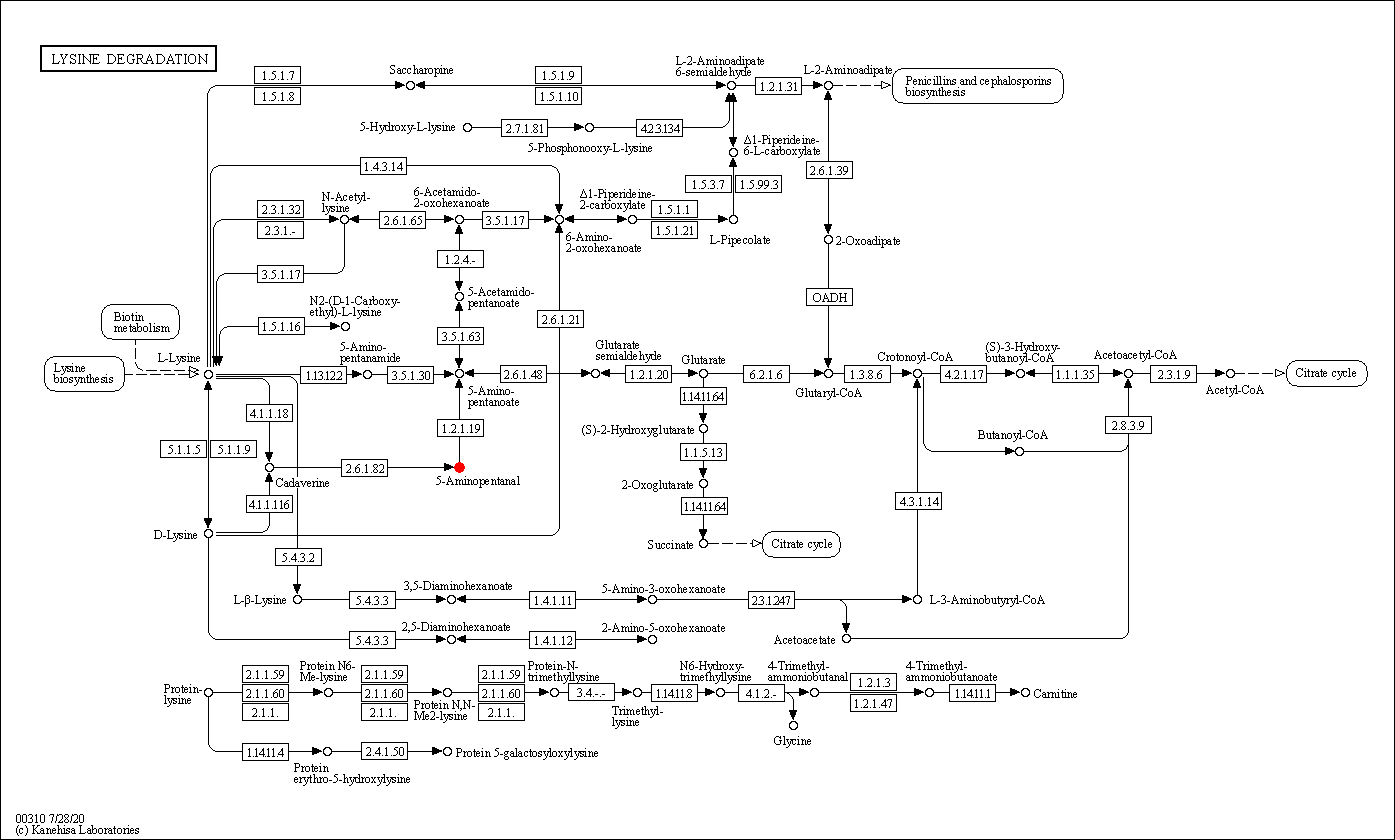

Supplement: Supplemental Information 13 [file peerj-10-14444-s013.zip › Web_Report/Diff_analysis/H_vs_L/KEGG/kegg_map/ko00310.png]
