# Supplementary material for: Multi-omics analyses reveal that the gut microbiome and its metabolites promote milk fat synthesis in Zhongdian yak cows
Source: PeerJ. 2022 Dec 2;10:e14444. doi: 10.7717/peerj.14444 (PMC9744170; doi:10.7717/peerj.14444)
Supplement: Supplemental Information 12 [file peerj-10-14444-s012.zip › Web_Report/index.html]

Neg-BMK211021-AP383-ZX01-0101-西南林业大学10个宏基因组12个代谢组联合分析

Neg-BMK211021-AP383-ZX01-0101-西南林业大学10个宏基因组12个代谢组联合分析

## Neg-BMK211021-AP383-ZX01-0101-西南林业大学10个宏基因组12个代谢组联合分析

### 摘要

基于宏基因组分析可得到微生物物种丰度和功能基因的相对含量，结合代谢组可揭示代谢物与这些物种丰度/功能基因之间的关系，并进一步分析微生物的种群结构、基因功能与代谢产物之间的关系。

### 1 代谢组与宏基因组物种丰度联合分析

#### 1.1 差异代谢物与物种丰度相关性分析

分析前，会对所有的物种丰度进行标准化，即每个物种的丰度除以样本所有物种的总表达量。

在计算相关性时，先使用了最大最小值标准化的方式对代谢和物种丰度进行了处理，然后采用了spearman方法对差异代谢物和差异物种的丰度进行相关性分析并绘制热图，结果如下：

- treat1\_H\_L.vs.H\_L\_taxonomy\_cor

图1.1 相关性热图

注：涉及物种和代谢物可能较多，不显示具体的名称。

为了进一步的分析，对相关性分析结果进行筛选，筛选条件为相关性系数和P值，其标准是：|CC|>0.80且CCP<0.05，部分结果如下表：

表1.1 差异代谢物及物种丰度相关性分析结果部分展示

| genusName | metaName | CC | CCP |
| --- | --- | --- | --- |
| Chloroflexus | neg\_1004 | -0.86 | 0.0015 |
| Pararhodospirillum | neg\_1004 | -0.83 | 0.0033 |
| Thermosyntropha | neg\_1004 | -0.8 | 0.0054 |
| Parafilimonas | neg\_1004 | 0.89 | 0.00048 |
| Emticicia | neg\_1062 | -0.83 | 0.0029 |
| Brevinema | neg\_1065 | 0.81 | 0.0049 |
| Candidatus\_Sulfopaludibacter | neg\_1065 | 0.84 | 0.0022 |
| Comamonas | neg\_1065 | -0.83 | 0.0029 |
| Candidatus\_Electrothrix | neg\_1065 | 0.82 | 0.0038 |

注：genus Name：物种的ID；metaName：差异代谢物ID；CC：相关性系数；CCP：相关性的p值，低于0.05为显著。

差异代谢物及物种多样性相关性分析结果

1. treat1\_H\_L.vs.H\_L\_taxonomy\_cor\_filter.xls

#### 1.2 相关性网络分析

对代谢物先进行加权共表达网络分析（WGCNA），把代谢物分成不同的模块，然后对于各个模块中的代谢物结合上述相关性分析结果可得到各个模块中代谢物与差异物种丰度之间的相关性网络图。其结果如下：

- treat1\_H\_L.vs.H\_L\_module\_1\_network
- treat1\_H\_L.vs.H\_L\_module\_2\_network

图2.2 相关性网路图

注：图中的圆圈表示物种，不同的颜色表示不同分类，矩形表示代谢物，蓝色的线表示负相关，红色的线表示正相关。目前网路图中最多可呈现200对代谢物与物种的关系，对于超过的可下载对应的文件利用CytoScape进行绘制。

#### 1.3 协惯量分析

协惯量分析（coinertia analysis）可用于两组变量的分析，常见于生态学中研究植被与环境的关系，随后被运用到多组学联合分析中。这里将差异物种按照所选分类，结合差异代谢物可展现出差异代谢物和微生物之间的关系，也能体现微生物不同类群间的分布情况，不同的差异分组中的近源物种分布可能不同。借助于R中的omicade4包进行分析的结果如下：

- treat1\_H\_L.vs.H\_L\_coinertia\_analysis

图3.3 协惯量分析结果图

注：图中一个圆代表一个物种，不同的颜色表示不同的分类，三角形是代谢物，物种、代谢物与原点连线形成的夹角反映了代谢物和物种之间的相关性，锐角为正相关，钝角为负相关，直角为不相关。

#### 1.4 对应分析

对应分析(Correspondence Analysis)也称关联分析，是一种多元统计分析技术，通过分析由定性变量构成的交互汇总表来揭示变量间的联系。可以揭示同一变量的各个类别之间的差异，以及不同变量各个类别之间的对应关系。将差异代谢物和菌群进行限制性对应分析（constrained correspondence analysis）能揭示菌群的分布特点的同时也能挖掘与之相关联的代谢物。这里使用了R中vegan包进行了分析，其结果如下：

- CCA

图4.4 限制性对应分析结果图

注：图中的点表示物种，箭头为代谢物。物种和原点连线与箭头形成的夹角反映了代谢物和物种之间的相关性，锐角为正相关，钝角为负相关，直角为不相关。

#### 1.5 随机森林分类分析

在机器学习中，随机森林（Random Forest）是一个包含多个决策树的分类器，并且其输出的类别是由个别树输出的类别的众数而定。在随机森林中， 集成模型中的每棵树构建时的样本都是由训练集经过有放回抽样得来的。随机森林构建过程的随机性能够产生具有不同预测错误的决策树。通过取这些决策树的平均，能够消除部分错误。随机森林建模可以在分类时评估特征的重要性。随机森林建模被广泛应用于少样本、高特征维度的数据集建模中。在多组学联合分析中，可以通过对模型特征的排序筛选出对模型重要的特征，从而起到筛选biomarker的目的。还可以通过不同组学的单独建模与合并数据建模的ROC曲线（受试者回归曲线，Receiver Operating Characteristic curve）对比，评估哪种组学能更好地分离对照组和实验组。

- treat1\_H\_L.vs.H\_L\_feature\_importance

图5.5 随机森林分类器特征重要性排序箱线图，将两组数据合并建模并按模型特征重要性从大到小排序，图片显示top 20的特征，依据yes重交叉验证结果作箱线图。

注：

- treat1\_H\_L.vs.H\_L\_ROC

图6.6 随机森林分类器ROC曲线图，虚线显示训练集yes重交叉验证的平均ROC曲线，实线显示独立验证的ROC曲线。不同颜色虚线和实线分别表示代谢组、宏基因组物种丰度分别建模以及合并建模的ROC曲线。

### 2 代谢组与宏基因组功能基因联合分析

#### 2.1 差异代谢物与差异功能基因相关性分析

分析前，会对所有的功能基因丰度进行标准化，即每个功能基因的丰度除以样本所有物种的总表达量。

在计算相关性时，先使用了最大最小值标准化的方式对代谢和功能基因丰度进行了处理，然后采用了spearman方法对差异代谢物和差异功能基因的丰度进行相关性分析并绘制热图，结果如下：

- treat1\_H\_L.vs.H\_L\_function\_cor

图7.1 相关性热图

注：涉及功能基因和代谢物可能较多，不显示具体的名称。

为了进一步的分析，对相关性分析结果进行筛选，筛选条件为相关性系数和P值，其标准是：|CC|>0.80且CCP<0.05，部分结果如下表：

表2.1 差异代谢物及功能基因丰度相关性分析结果部分展示

| GenesName | metaName | CC | CCP |
| --- | --- | --- | --- |
| K01814 | neg\_1004 | -0.81 | 0.0042 |
| K12072 | neg\_1004 | 0.85 | 0.0016 |
| K09815 | neg\_1004 | -0.8 | 0.0054 |
| K02665 | neg\_1004 | 0.9 | 0.00036 |
| K21217 | neg\_1004 | 0.87 | 0.00096 |
| K06871 | neg\_1004 | -0.88 | 0.00074 |
| K16014 | neg\_1004 | -0.81 | 0.0048 |
| K03414 | neg\_1004 | 0.82 | 0.0039 |
| K10942 | neg\_1004 | 0.82 | 0.0039 |

注：geneName：基因的ID；metaName：差异代谢物ID；CC：相关性系数；CCP：相关性的p值，低于0.05为显著。

差异代谢物及功能基因丰度相关性分析结果

1. treat1\_H\_L.vs.H\_L\_function\_cor\_filter.xls

#### 2.2 相关性网络分析

对代谢物先进行加权共表达网络分析（WGCNA），把代谢物分成不同的模块，然后对于各个模块中的代谢物结合上述相关性分析结果可得到各个模块中代谢物与差异功能基因丰度之间的相关性网络图。其结果如下：

- treat1\_H\_L.vs.H\_L\_module\_1\_network
- treat1\_H\_L.vs.H\_L\_module\_2\_network

图8.2 相关性网路图

注：图中的圆圈表示功能基因，不同的颜色表示不同分类，矩形表示代谢物，蓝色的线表示负相关，红色的线表示正相关。目前网路图中最多可呈现200对代谢物与功能基因的关系，对于超过的可下载对应的文件利用CytoScape进行绘制。

#### 2.3 协惯量分析

协惯量分析（coinertia analysis）可用于两组变量的分析，常见于生态学中研究植被与环境的关系，随后被运用到多组学联合分析中。这里将差异功能基因按照所选分类，结合差异代谢物可展现出差异代谢物和微生物功能基因之间的关系，也能体现微生物不同功能基因间的潜在互作趋势，不同的差异分组中的功能基因分布可能不同。借助于R中的omicade4包进行分析的结果如下：

- treat1\_H\_L.vs.H\_L\_coinertia\_analysis

图9.3 协惯量分析结果图

注：图中一个圆代表一个功能基因，不同的颜色表示不同的分类，三角形是代谢物，物种、代谢物与原点连线形成的夹角反映了代谢物和功能基因之间的相关性，锐角为正相关，钝角为负相关，直角为不相关。

#### 2.4 对应分析

对应分析(Correspondence Analysis)也称关联分析，是一种多元统计分析技术，通过分析由定性变量构成的交互汇总表来揭示变量间的联系。可以揭示同一变量的各个类别之间的差异，以及不同变量各个类别之间的对应关系。将差异代谢物和功能基因进行限制性对应分析（constrained correspondence analysis）能揭示菌群组间差异功能基因的分布特点的同时也能挖掘与之相关联的代谢物。这里使用了R中vegan包进行了分析，其结果如下：

- CCA

图10.4 限制性对应分析结果图

注：图中的点表示基因，箭头为代谢物。基因和原点连线与箭头形成的夹角反映了代谢物和基因之间的相关性，锐角为正相关，钝角为负相关，直角为不相关。

#### 2.5 随机森林分类分析

在机器学习中，随机森林（Random Forest）是一个包含多个决策树的分类器，并且其输出的类别是由个别树输出的类别的众数而定。在随机森林中， 集成模型中的每棵树构建时的样本都是由训练集经过有放回抽样得来的。随机森林构建过程的随机性能够产生具有不同预测错误的决策树。通过取这些决策树的平均，能够消除部分错误。随机森林建模可以在分类时评估特征的重要性。随机森林建模被广泛应用于少样本、高特征维度的数据集建模中。在多组学联合分析中，可以通过对模型特征的排序筛选出对模型重要的特征，从而起到筛选biomarker的目的。还可以通过不同组学的单独建模与合并数据建模的ROC曲线（受试者回归曲线，Receiver Operating Characteristic curve）对比，评估哪种组学能更好地分离对照组和实验组。

- treat1\_H\_L.vs.H\_L\_feature\_importance

图11.5 随机森林分类器特征重要性排序箱线图

注：将两组数据合并建模并按模型特征重要性从大到小排序，图片显示top 20的特征，依据yes重交叉验证结果作箱线图。

- treat1\_H\_L.vs.H\_L\_ROC

图12.6 随机森林分类器ROC曲线图

注：虚线显示训练集yes重交叉验证的平均ROC曲线，实线显示独立验证的ROC曲线。不同颜色虚线和实线分别表示代谢组、宏基因组功能基因分别建模以及合并建模的ROC曲线。

### 3 参考文献

1. McHardy I H, Goudarzi M, Tong M, et al. Integrative analysis of the microbiome and metabolome of the human intestinal mucosal surface reveals exquisite inter-relationships[J]. Microbiome, 2013, 1(1): 17.
2. Meng C, Kuster B, Culhane A C, et al. A multivariate approach to the integration of multi-omics datasets[J]. BMC bioinformatics, 2014, 15(1): 162.
3. Yachida S, Mizutani S, Shiroma H, et al. Metagenomic and metabolomic analyses reveal distinct stage-specific phenotypes of the gut microbiota in colorectal cancer[J]. Nature Medicine, 2019, 25(6): 968–976.

- 摘要
- 1 代谢组与宏基因组物种丰度联合分析
  - 1.1 差异代谢物与物种丰度相关性分析
  - 1.2 相关性网络分析
  - 1.3 协惯量分析
  - 1.4 对应分析
  - 1.5 随机森林分类分析
- 2 代谢组与宏基因组功能基因联合分析
  - 2.1 差异代谢物与差异功能基因相关性分析
  - 2.2 相关性网络分析
  - 2.3 协惯量分析
  - 2.4 对应分析
  - 2.5 随机森林分类分析
- 3 参考文献

Copyright © 2009-2021 北京百迈客生物科技有限公司版权所有 京ICP备10042835号

公司地址：北京市顺义区南法信府前街12号顺捷大厦5层

- Tel:400-600-3186
- Fax:010-57045001
- E-mail:tech@biomarker.com.cn
- 微信:biomarker\_tech
- 百迈客生物云平台
- 关于我们
