# Supplementary material for: Multi-omics analyses reveal that the gut microbiome and its metabolites promote milk fat synthesis in Zhongdian yak cows
Source: PeerJ. 2022 Dec 2;10:e14444. doi: 10.7717/peerj.14444 (PMC9744170; doi:10.7717/peerj.14444)
Supplement: Supplemental Information 12 [file peerj-10-14444-s012.zip › Web_Report/HTML/treat1_H_L.vs.H_L_function_cor_filter.xls.html]

treat1\_H\_L.vs.H\_L\_function\_cor\_filter.xls


## treat1\_H\_L.vs.H\_L\_function\_cor\_filter.xls

| GenesName | metaName | CC | CCP |
| --- | --- | --- | --- |
| K01814 | neg\_1004 | -0.812897026538849 | 0.00424919679863534 |
| K12072 | neg\_1004 | 0.854838728904724 | 0.00162424574299602 |
| K09815 | neg\_1004 | -0.800390899181366 | 0.0054162086926719 |
| K02665 | neg\_1004 | 0.90172153711319 | 0.000361951655085679 |
| K21217 | neg\_1004 | 0.873542726039886 | 0.000957809429852796 |
| K06871 | neg\_1004 | -0.881680607795715 | 0.000741594922435862 |
| K16014 | neg\_1004 | -0.806451618671417 | 0.00482540374486629 |
| K03414 | neg\_1004 | 0.817185163497925 | 0.00389426659690773 |
| K10942 | neg\_1004 | 0.817185163497925 | 0.00389426659690773 |
| K18198 | neg\_1065 | -0.842424213886261 | 0.00222003275351312 |
| K07672 | neg\_1065 | 0.806643962860107 | 0.00480744414521128 |
| K03340 | neg\_1065 | 0.818181812763214 | 0.00381492051076338 |
| K11754 | neg\_1065 | 0.818181812763214 | 0.00381492051076338 |
| K02493 | neg\_1065 | 0.939393937587738 | 5.48405364009241e-05 |
| K02784 | neg\_1065 | 0.903030276298523 | 0.000343612565232743 |
| K03769 | neg\_1065 | 0.854545474052429 | 0.00163680247839526 |
| K17331 | neg\_1065 | 0.887625336647034 | 0.000607926148504134 |
| K09761 | neg\_1065 | 0.818181812763214 | 0.00381492051076338 |
| K19334 | neg\_1065 | 0.854545474052429 | 0.00163680247839526 |
| K21217 | neg\_1065 | -0.826174378395081 | 0.00321991677364641 |
| K03573 | neg\_1065 | 0.854545474052429 | 0.00163680247839526 |
| K03501 | neg\_1065 | 0.842424213886261 | 0.00222003275351312 |
| K00344 | neg\_1065 | 0.830303013324738 | 0.00294022813612127 |
| K00969 | neg\_1065 | 0.830303013324738 | 0.00294022813612127 |
| K06145 | neg\_1065 | 0.818181812763214 | 0.00381492051076338 |
| K05970 | neg\_1065 | 0.866666674613953 | 0.0011735379121256 |
| K01409 | neg\_1065 | 0.830303013324738 | 0.00294022813612127 |
| K00919 | neg\_1065 | 0.842424213886261 | 0.00222003275351312 |
| K20525 | neg\_1065 | 0.927272737026215 | 0.000112034447641074 |
| K02458 | neg\_1109 | 0.816012322902679 | 0.0039891464803441 |
| K05802 | neg\_1109 | 0.821065366268158 | 0.00359188206024896 |
| K09512 | neg\_1109 | -0.846925735473633 | 0.0019883679465349 |
| K06311 | neg\_1109 | 0.863225877285004 | 0.00129381457010247 |
| K04565 | neg\_1112 | 0.830303013324738 | 0.00294022813612127 |
| K07139 | neg\_1112 | 0.830303013324738 | 0.00294022813612127 |
| K05787 | neg\_1112 | 0.854545474052429 | 0.00163680247839526 |
| K20452 | neg\_1112 | -0.901908397674561 | 0.000359290276283897 |
| K14575 | neg\_1112 | 0.802435338497162 | 0.00521145970824932 |
| K01814 | neg\_1116 | 0.830303013324738 | 0.00294022813612127 |
| K12072 | neg\_1116 | -0.850415349006653 | 0.00182121334938934 |
| K02665 | neg\_1116 | -0.887625336647034 | 0.000607926148504134 |
| K21217 | neg\_1116 | -0.833002269268036 | 0.00276710271221536 |
| K06871 | neg\_1116 | 0.878787875175476 | 0.000813862205061078 |
| K16014 | neg\_1116 | 0.81915009021759 | 0.00373894966455346 |
| K11933 | neg\_112 | -0.835593700408936 | 0.00260791837015528 |
| K17329 | neg\_112 | -0.860478162765503 | 0.00139610202996154 |
| K10237 | neg\_112 | -0.813785552978516 | 0.00417382592013071 |
| K02461 | neg\_112 | -0.935483872890472 | 7.00850078045878e-05 |
| K03651 | neg\_112 | 0.800390899181366 | 0.0054162086926719 |
| K02665 | neg\_112 | -0.845363914966583 | 0.00206666064770777 |
| K21217 | neg\_112 | -0.845363914966583 | 0.00206666064770777 |
| K19157 | neg\_112 | 0.912945866584778 | 0.000225958764512368 |
| K03414 | neg\_112 | -0.845363914966583 | 0.00206666064770777 |
| K05802 | neg\_112 | -0.900500416755676 | 0.000379701199800131 |
| K05851 | neg\_112 | -0.800000011920929 | 0.00545599878335024 |
| K10942 | neg\_112 | -0.845363914966583 | 0.00206666064770777 |
| K06311 | neg\_112 | -0.865548312664032 | 0.00121169851367053 |
| K02919 | neg\_112 | -0.81915009021759 | 0.00373894966455346 |
| K12543 | neg\_1139 | 0.866666674613953 | 0.0011735379121256 |
| K06139 | neg\_1139 | -0.830303013324738 | 0.00294022813612127 |
| K00013 | neg\_1188 | -0.830303013324738 | 0.00294022813612127 |
| K02665 | neg\_1188 | 0.846658051013947 | 0.00200163182665913 |
| K21217 | neg\_1188 | 0.819346487522125 | 0.00372367376291516 |
| K16014 | neg\_1188 | -0.931705057621002 | 8.75967112814457e-05 |
| K06145 | neg\_1188 | -0.866666674613953 | 0.0011735379121256 |
| K03414 | neg\_1188 | 0.805690705776215 | 0.00489692183645341 |
| K10942 | neg\_1188 | 0.805690705776215 | 0.00489692183645341 |
| K17329 | neg\_1307 | -0.859855890274048 | 0.00142005806532719 |
| K02461 | neg\_1307 | -0.856668412685394 | 0.00154747182074688 |
| K03651 | neg\_1307 | 0.842424213886261 | 0.00222003275351312 |
| K02665 | neg\_1307 | -0.833002269268036 | 0.00276710271221536 |
| K19157 | neg\_1307 | 0.878787875175476 | 0.000813862205061078 |
| K04748 | neg\_1307 | -0.818181812763214 | 0.00381492051076338 |
| K06145 | neg\_1307 | 0.806060612201691 | 0.0048620605246823 |
| K05802 | neg\_1307 | -0.898646354675293 | 0.000407860267078952 |
| K06311 | neg\_1307 | -0.826751530170441 | 0.00317971472634282 |
| K02919 | neg\_1307 | -0.806060612201691 | 0.0048620605246823 |
| K02461 | neg\_1330 | -0.848254203796387 | 0.00192348015922827 |
| K02665 | neg\_1330 | -0.8156378865242 | 0.00401978302331507 |
| K21217 | neg\_1330 | -0.8156378865242 | 0.00401978302331507 |
| K19157 | neg\_1330 | 0.889637529850006 | 0.000566964581027118 |
| K03414 | neg\_1330 | -0.843286633491516 | 0.00217421422639408 |
| K05802 | neg\_1330 | -0.922830998897552 | 0.000141238827938484 |
| K10942 | neg\_1330 | -0.843286633491516 | 0.00217421422639408 |
| K06311 | neg\_1330 | -0.916962146759033 | 0.000187990654342052 |
| K02919 | neg\_1330 | -0.816012322902679 | 0.0039891464803441 |
| K16014 | neg\_1354 | -0.919198930263519 | 0.00016900592052238 |
| K03414 | neg\_1354 | 0.819346487522125 | 0.00372367376291516 |
| K05802 | neg\_1354 | 0.846925735473633 | 0.0019883679465349 |
| K10942 | neg\_1354 | 0.819346487522125 | 0.00372367376291516 |
| K18198 | neg\_136 | 0.854545474052429 | 0.00163680247839526 |
| K17329 | neg\_136 | 0.924506723880768 | 0.000129632545059266 |
| K00100 | neg\_136 | 0.818181812763214 | 0.00381492051076338 |
| K02461 | neg\_136 | 0.856668412685394 | 0.00154747182074688 |
| K07126 | neg\_136 | -0.818181812763214 | 0.00381492051076338 |
| K03651 | neg\_136 | -0.878787875175476 | 0.000813862205061078 |
| K09789 | neg\_136 | -0.830303013324738 | 0.00294022813612127 |
| K02665 | neg\_136 | 0.846658051013947 | 0.00200163182665913 |
| K04748 | neg\_136 | 0.890909075737 | 0.000542144516154419 |
| K06145 | neg\_136 | -0.878787875175476 | 0.000813862205061078 |
| K05802 | neg\_136 | 0.833995580673218 | 0.00270528265085312 |
| K09512 | neg\_136 | -0.846925735473633 | 0.0019883679465349 |
| K06311 | neg\_136 | 0.83890962600708 | 0.00241398974898654 |
| K17329 | neg\_1361 | -0.911576509475708 | 0.000240107073119944 |
| K10237 | neg\_1361 | -0.833995580673218 | 0.00270528265085312 |
| K02461 | neg\_1361 | -0.90043979883194 | 0.000380598613888061 |
| K01729 | neg\_1361 | 0.866666674613953 | 0.0011735379121256 |
| K03651 | neg\_1361 | 0.818181812763214 | 0.00381492051076338 |
| K06285 | neg\_1361 | -0.854545474052429 | 0.00163680247839526 |
| K12072 | neg\_1361 | -0.812897026538849 | 0.00424919679863534 |
| K04565 | neg\_1361 | 0.818181812763214 | 0.00381492051076338 |
| K02665 | neg\_1361 | -0.873969614505768 | 0.000945449010965937 |
| K21217 | neg\_1361 | -0.846658051013947 | 0.00200163182665913 |
| K03643 | neg\_1361 | 0.830303013324738 | 0.00294022813612127 |
| K19157 | neg\_1361 | 0.854545474052429 | 0.00163680247839526 |
| K18692 | neg\_1361 | -0.830303013324738 | 0.00294022813612127 |
| K06145 | neg\_1361 | 0.830303013324738 | 0.00294022813612127 |
| K17680 | neg\_1361 | -0.806060612201691 | 0.0048620605246823 |
| K05851 | neg\_1361 | -0.806643962860107 | 0.00480744414521128 |
| K18298 | neg\_1361 | -0.866666674613953 | 0.0011735379121256 |
| K00057 | neg\_1361 | 0.806060612201691 | 0.0048620605246823 |
| K11754 | neg\_1363 | 0.830303013324738 | 0.00294022813612127 |
| K00297 | neg\_1363 | 0.903030276298523 | 0.000343612565232743 |
| K00848 | neg\_1363 | 0.854545474052429 | 0.00163680247839526 |
| K01151 | neg\_1363 | 0.842424213886261 | 0.00222003275351312 |
| K11933 | neg\_1382 | -0.865095794200897 | 0.00122739260821847 |
| K17329 | neg\_1382 | -0.872786045074463 | 0.000980007503875058 |
| K02461 | neg\_1382 | -0.831656157970428 | 0.00285249465359505 |
| K02745 | neg\_1382 | -0.806060612201691 | 0.0048620605246823 |
| K03651 | neg\_1382 | 0.866666674613953 | 0.0011735379121256 |
| K06926 | neg\_1382 | 0.806060612201691 | 0.0048620605246823 |
| K19157 | neg\_1382 | 0.806060612201691 | 0.0048620605246823 |
| K04748 | neg\_1382 | -0.866666674613953 | 0.0011735379121256 |
| K18692 | neg\_1382 | -0.806060612201691 | 0.0048620605246823 |
| K06145 | neg\_1382 | 0.830303013324738 | 0.00294022813612127 |
| K03414 | neg\_1382 | -0.819346487522125 | 0.00372367376291516 |
| K05802 | neg\_1382 | -0.885716199874878 | 0.000648751005291714 |
| K10942 | neg\_1382 | -0.819346487522125 | 0.00372367376291516 |
| K06311 | neg\_1382 | -0.826751530170441 | 0.00317971472634282 |
| K02919 | neg\_1382 | -0.830303013324738 | 0.00294022813612127 |
| K18198 | neg\_1401 | 0.866666674613953 | 0.0011735379121256 |
| K08714 | neg\_1401 | -0.818181812763214 | 0.00381492051076338 |
| K07672 | neg\_1401 | -0.850415349006653 | 0.00182121334938934 |
| K03340 | neg\_1401 | -0.842424213886261 | 0.00222003275351312 |
| K03470 | neg\_1401 | -0.818181812763214 | 0.00381492051076338 |
| K10237 | neg\_1401 | 0.821065366268158 | 0.00359188206024896 |
| K11754 | neg\_1401 | -0.878787875175476 | 0.000813862205061078 |
| K02461 | neg\_1401 | 0.831656157970428 | 0.00285249465359505 |
| K07126 | neg\_1401 | -0.866666674613953 | 0.0011735379121256 |
| K03651 | neg\_1401 | -0.866666674613953 | 0.0011735379121256 |
| K12543 | neg\_1401 | 0.854545474052429 | 0.00163680247839526 |
| K05830 | neg\_1401 | 0.963636338710785 | 7.32099466027591e-06 |
| K07454 | neg\_1401 | -0.806060612201691 | 0.0048620605246823 |
| K06926 | neg\_1401 | -0.830303013324738 | 0.00294022813612127 |
| K01909 | neg\_1401 | 0.818181812763214 | 0.00381492051076338 |
| K06145 | neg\_1401 | -0.830303013324738 | 0.00294022813612127 |
| K00057 | neg\_1401 | -0.842424213886261 | 0.00222003275351312 |
| K08714 | neg\_1413 | -0.842424213886261 | 0.00222003275351312 |
| K17329 | neg\_1413 | 0.898646354675293 | 0.000407860267078952 |
| K01159 | neg\_1413 | -0.903030276298523 | 0.000343612565232743 |
| K03470 | neg\_1413 | -0.866666674613953 | 0.0011735379121256 |
| K04061 | neg\_1413 | 0.818181812763214 | 0.00381492051076338 |
| K04096 | neg\_1413 | -0.806060612201691 | 0.0048620605246823 |
| K02461 | neg\_1413 | 0.850415349006653 | 0.00182121334938934 |
| K07126 | neg\_1413 | -0.878787875175476 | 0.000813862205061078 |
| K06285 | neg\_1413 | 0.915151536464691 | 0.000204472206099204 |
| K02852 | neg\_1413 | -0.842424213886261 | 0.00222003275351312 |
| K04565 | neg\_1413 | -0.806060612201691 | 0.0048620605246823 |
| K02665 | neg\_1413 | 0.839830160140991 | 0.00236205253090405 |
| K07139 | neg\_1413 | -0.806060612201691 | 0.0048620605246823 |
| K05787 | neg\_1413 | -0.842424213886261 | 0.00222003275351312 |
| K00059 | neg\_1413 | -0.878787875175476 | 0.000813862205061078 |
| K03643 | neg\_1413 | -0.915151536464691 | 0.000204472206099204 |
| K06179 | neg\_1413 | -0.842424213886261 | 0.00222003275351312 |
| K06926 | neg\_1413 | -0.903030276298523 | 0.000343612565232743 |
| K00783 | neg\_1413 | -0.818181812763214 | 0.00381492051076338 |
| K00849 | neg\_1413 | -0.806060612201691 | 0.0048620605246823 |
| K11068 | neg\_1413 | -0.866666674613953 | 0.0011735379121256 |
| K05851 | neg\_1413 | 0.831656157970428 | 0.00285249465359505 |
| K14575 | neg\_1413 | -0.875383973121643 | 0.00090532610126548 |
| K14051 | neg\_1413 | -0.846658051013947 | 0.00200163182665913 |
| K01409 | neg\_1413 | -0.806060612201691 | 0.0048620605246823 |
| K09951 | neg\_1413 | -0.806060612201691 | 0.0048620605246823 |
| K00057 | neg\_1413 | -0.842424213886261 | 0.00222003275351312 |
| K08714 | neg\_1432 | -0.806060612201691 | 0.0048620605246823 |
| K03524 | neg\_1432 | -0.806060612201691 | 0.0048620605246823 |
| K02461 | neg\_1432 | 0.800390899181366 | 0.0054162086926719 |
| K06285 | neg\_1432 | 0.806060612201691 | 0.0048620605246823 |
| K02852 | neg\_1432 | -0.806060612201691 | 0.0048620605246823 |
| K04565 | neg\_1432 | -0.818181812763214 | 0.00381492051076338 |
| K07139 | neg\_1432 | -0.878787875175476 | 0.000813862205061078 |
| K05787 | neg\_1432 | -0.878787875175476 | 0.000813862205061078 |
| K03643 | neg\_1432 | -0.866666674613953 | 0.0011735379121256 |
| K06179 | neg\_1432 | -0.818181812763214 | 0.00381492051076338 |
| K11068 | neg\_1432 | -0.842424213886261 | 0.00222003275351312 |
| K05851 | neg\_1432 | 0.881680607795715 | 0.000741594922435862 |
| K14575 | neg\_1432 | -0.936174511909485 | 6.71888372667517e-05 |
| K14051 | neg\_1432 | -0.887625336647034 | 0.000607926148504134 |
| K09951 | neg\_1432 | -0.866666674613953 | 0.0011735379121256 |
| K00845 | neg\_1438 | 0.818181812763214 | 0.00381492051076338 |
| K18198 | neg\_1438 | -0.842424213886261 | 0.00222003275351312 |
| K00640 | neg\_1438 | 0.818181812763214 | 0.00381492051076338 |
| K07672 | neg\_1438 | 0.894186735153198 | 0.000481835628326488 |
| K05946 | neg\_1438 | 0.951515138149261 | 2.27985739738035e-05 |
| K07099 | neg\_1438 | 0.842424213886261 | 0.00222003275351312 |
| K03340 | neg\_1438 | 0.890909075737 | 0.000542144516154419 |
| K03827 | neg\_1438 | -0.818181812763214 | 0.00381492051076338 |
| K03470 | neg\_1438 | 0.830303013324738 | 0.00294022813612127 |
| K10237 | neg\_1438 | -0.859855890274048 | 0.00142005806532719 |
| K01261 | neg\_1438 | 0.818181812763214 | 0.00381492051076338 |
| K07166 | neg\_1438 | 0.830303013324738 | 0.00294022813612127 |
| K17331 | neg\_1438 | 0.860313832759857 | 0.00140239962667921 |
| K09761 | neg\_1438 | 0.878787875175476 | 0.000813862205061078 |
| K00949 | neg\_1438 | 0.830303013324738 | 0.00294022813612127 |
| K14571 | neg\_1438 | -0.844988703727722 | 0.00208579660703911 |
| K15599 | neg\_1438 | -0.890909075737 | 0.000542144516154419 |
| K07396 | neg\_1438 | -0.890909075737 | 0.000542144516154419 |
| K03607 | neg\_1438 | -0.833002269268036 | 0.00276710271221536 |
| K05985 | neg\_1438 | 0.830303013324738 | 0.00294022813612127 |
| K12231 | neg\_1438 | -0.846658051013947 | 0.00200163182665913 |
| K05989 | neg\_1438 | 0.866666674613953 | 0.0011735379121256 |
| K09777 | neg\_1438 | 0.842424213886261 | 0.00222003275351312 |
| K15792 | neg\_1438 | 0.818181812763214 | 0.00381492051076338 |
| K00859 | neg\_1438 | 0.818181812763214 | 0.00381492051076338 |
| K16199 | neg\_1438 | 0.830303013324738 | 0.00294022813612127 |
| K01990 | neg\_1438 | 0.806060612201691 | 0.0048620605246823 |
| K00849 | neg\_1438 | 0.806060612201691 | 0.0048620605246823 |
| K00969 | neg\_1438 | 0.866666674613953 | 0.0011735379121256 |
| K03218 | neg\_1438 | 0.818181812763214 | 0.00381492051076338 |
| K10578 | neg\_1438 | -0.846658051013947 | 0.00200163182665913 |
| K01736 | neg\_1438 | 0.806060612201691 | 0.0048620605246823 |
| K07053 | neg\_1438 | 0.842424213886261 | 0.00222003275351312 |
| K01299 | neg\_1438 | -0.854545474052429 | 0.00163680247839526 |
| K15598 | neg\_1438 | -0.806060612201691 | 0.0048620605246823 |
| K05964 | neg\_1438 | -0.842424213886261 | 0.00222003275351312 |
| K07707 | neg\_1438 | -0.830303013324738 | 0.00294022813612127 |
| K02461 | neg\_1450 | -0.900500416755676 | 0.000379701199800131 |
| K19157 | neg\_1450 | 0.911576509475708 | 0.000240107073119944 |
| K05802 | neg\_1450 | -0.862068951129913 | 0.00133619469409485 |
| K09512 | neg\_1450 | 0.834482729434967 | 0.00267533077056248 |
| K06311 | neg\_1450 | -0.830049574375153 | 0.00295687357761532 |
| K02919 | neg\_1450 | -0.859855890274048 | 0.00142005806532719 |
| K18198 | neg\_1496 | -0.890909075737 | 0.000542144516154419 |
| K07672 | neg\_1496 | 0.812897026538849 | 0.00424919679863534 |
| K11933 | neg\_1496 | -0.816012322902679 | 0.0039891464803441 |
| K17329 | neg\_1496 | -0.821065366268158 | 0.00359188206024896 |
| K10237 | neg\_1496 | -0.872786045074463 | 0.000980007503875058 |
| K02461 | neg\_1496 | -0.869174480438232 | 0.00109115185763287 |
| K02745 | neg\_1496 | -0.806060612201691 | 0.0048620605246823 |
| K03651 | neg\_1496 | 0.915151536464691 | 0.000204472206099204 |
| K05830 | neg\_1496 | -0.878787875175476 | 0.000813862205061078 |
| K21217 | neg\_1496 | -0.833002269268036 | 0.00276710271221536 |
| K19157 | neg\_1496 | 0.866666674613953 | 0.0011735379121256 |
| K04748 | neg\_1496 | -0.854545474052429 | 0.00163680247839526 |
| K16014 | neg\_1496 | 0.806643962860107 | 0.00480744414521128 |
| K06145 | neg\_1496 | 0.890909075737 | 0.000542144516154419 |
| K03414 | neg\_1496 | -0.873969614505768 | 0.000945449010965937 |
| K05802 | neg\_1496 | -0.885716199874878 | 0.000648751005291714 |
| K10942 | neg\_1496 | -0.873969614505768 | 0.000945449010965937 |
| K06311 | neg\_1496 | -0.869304955005646 | 0.00108698415655106 |
| K02919 | neg\_1496 | -0.854545474052429 | 0.00163680247839526 |
| K18198 | neg\_1532 | 0.915151536464691 | 0.000204472206099204 |
| K07672 | neg\_1532 | -0.919198930263519 | 0.00016900592052238 |
| K01060 | neg\_1532 | -0.818181812763214 | 0.00381492051076338 |
| K05946 | neg\_1532 | -0.806060612201691 | 0.0048620605246823 |
| K17329 | neg\_1532 | 0.859855890274048 | 0.00142005806532719 |
| K03340 | neg\_1532 | -0.830303013324738 | 0.00294022813612127 |
| K01159 | neg\_1532 | -0.806060612201691 | 0.0048620605246823 |
| K01261 | neg\_1532 | -0.903030276298523 | 0.000343612565232743 |
| K00100 | neg\_1532 | 0.830303013324738 | 0.00294022813612127 |
| K04061 | neg\_1532 | 0.878787875175476 | 0.000813862205061078 |
| K03406 | neg\_1532 | 0.951515138149261 | 2.27985739738035e-05 |
| K07126 | neg\_1532 | -0.818181812763214 | 0.00381492051076338 |
| K02745 | neg\_1532 | 0.915151536464691 | 0.000204472206099204 |
| K00852 | neg\_1532 | -0.818181812763214 | 0.00381492051076338 |
| K19411 | neg\_1532 | -0.939393937587738 | 5.48405364009241e-05 |
| K03412 | neg\_1532 | 0.806060612201691 | 0.0048620605246823 |
| K03651 | neg\_1532 | -0.927272737026215 | 0.000112034447641074 |
| K17331 | neg\_1532 | -0.81251859664917 | 0.00428159166664499 |
| K19334 | neg\_1532 | -0.878787875175476 | 0.000813862205061078 |
| K01684 | neg\_1532 | -0.818181812763214 | 0.00381492051076338 |
| K03573 | neg\_1532 | -0.854545474052429 | 0.00163680247839526 |
| K02747 | neg\_1532 | 0.806060612201691 | 0.0048620605246823 |
| K15520 | neg\_1532 | -0.927272737026215 | 0.000112034447641074 |
| K13014 | neg\_1532 | -0.854545474052429 | 0.00163680247839526 |
| K04748 | neg\_1532 | 0.927272737026215 | 0.000112034447641074 |
| K02744 | neg\_1532 | 0.806060612201691 | 0.0048620605246823 |
| K01909 | neg\_1532 | 0.830303013324738 | 0.00294022813612127 |
| K04085 | neg\_1532 | 0.806060612201691 | 0.0048620605246823 |
| K06145 | neg\_1532 | -0.951515138149261 | 2.27985739738035e-05 |
| K00230 | neg\_1532 | -0.830303013324738 | 0.00294022813612127 |
| K08169 | neg\_1532 | 0.842424213886261 | 0.00222003275351312 |
| K01299 | neg\_1532 | 0.842424213886261 | 0.00222003275351312 |
| K07341 | neg\_1532 | -0.806060612201691 | 0.0048620605246823 |
| K05964 | neg\_1532 | 0.806060612201691 | 0.0048620605246823 |
| K03415 | neg\_1532 | 0.854545474052429 | 0.00163680247839526 |
| K02784 | neg\_1554 | 0.830303013324738 | 0.00294022813612127 |
| K05970 | neg\_1554 | 0.806060612201691 | 0.0048620605246823 |
| K20525 | neg\_1554 | 0.866666674613953 | 0.0011735379121256 |
| K18198 | neg\_1563 | 0.830303013324738 | 0.00294022813612127 |
| K07126 | neg\_1563 | -0.818181812763214 | 0.00381492051076338 |
| K19411 | neg\_1563 | -0.951515138149261 | 2.27985739738035e-05 |
| K02493 | neg\_1563 | -0.854545474052429 | 0.00163680247839526 |
| K06285 | neg\_1563 | 0.806060612201691 | 0.0048620605246823 |
| K03769 | neg\_1563 | -0.878787875175476 | 0.000813862205061078 |
| K17331 | neg\_1563 | -0.833002269268036 | 0.00276710271221536 |
| K19334 | neg\_1563 | -0.939393937587738 | 5.48405364009241e-05 |
| K04070 | neg\_1563 | -0.818181812763214 | 0.00381492051076338 |
| K03573 | neg\_1563 | -0.915151536464691 | 0.000204472206099204 |
| K15520 | neg\_1563 | -0.806060612201691 | 0.0048620605246823 |
| K00344 | neg\_1563 | -0.842424213886261 | 0.00222003275351312 |
| K13014 | neg\_1563 | -0.890909075737 | 0.000542144516154419 |
| K05970 | neg\_1563 | -0.818181812763214 | 0.00381492051076338 |
| K00230 | neg\_1563 | -0.806060612201691 | 0.0048620605246823 |
| K20525 | neg\_1563 | -0.806060612201691 | 0.0048620605246823 |
| K18198 | neg\_162 | -0.927272737026215 | 0.000112034447641074 |
| K00640 | neg\_162 | 0.830303013324738 | 0.00294022813612127 |
| K07672 | neg\_162 | 0.887933671474457 | 0.000601513437458179 |
| K05946 | neg\_162 | 0.854545474052429 | 0.00163680247839526 |
| K03340 | neg\_162 | 0.915151536464691 | 0.000204472206099204 |
| K01159 | neg\_162 | 0.830303013324738 | 0.00294022813612127 |
| K03470 | neg\_162 | 0.878787875175476 | 0.000813862205061078 |
| K07391 | neg\_162 | 0.830303013324738 | 0.00294022813612127 |
| K10237 | neg\_162 | -0.833995580673218 | 0.00270528265085312 |
| K11754 | neg\_162 | 0.806060612201691 | 0.0048620605246823 |
| K04061 | neg\_162 | -0.842424213886261 | 0.00222003275351312 |
| K07126 | neg\_162 | 0.915151536464691 | 0.000204472206099204 |
| K03651 | neg\_162 | 0.806060612201691 | 0.0048620605246823 |
| K09761 | neg\_162 | 0.866666674613953 | 0.0011735379121256 |
| K12543 | neg\_162 | -0.842424213886261 | 0.00222003275351312 |
| K14571 | neg\_162 | -0.83890962600708 | 0.00241398974898654 |
| K05830 | neg\_162 | -0.878787875175476 | 0.000813862205061078 |
| K15599 | neg\_162 | -0.818181812763214 | 0.00381492051076338 |
| K02802 | neg\_162 | 0.833002269268036 | 0.00276710271221536 |
| K06926 | neg\_162 | 0.842424213886261 | 0.00222003275351312 |
| K03501 | neg\_162 | 0.806060612201691 | 0.0048620605246823 |
| K01990 | neg\_162 | 0.866666674613953 | 0.0011735379121256 |
| K03101 | neg\_162 | 0.830303013324738 | 0.00294022813612127 |
| K00849 | neg\_162 | 0.818181812763214 | 0.00381492051076338 |
| K06287 | neg\_162 | 0.818181812763214 | 0.00381492051076338 |
| K00969 | neg\_162 | 0.854545474052429 | 0.00163680247839526 |
| K20885 | neg\_162 | 0.818181812763214 | 0.00381492051076338 |
| K00945 | neg\_162 | 0.818181812763214 | 0.00381492051076338 |
| K05970 | neg\_162 | 0.842424213886261 | 0.00222003275351312 |
| K05964 | neg\_162 | -0.818181812763214 | 0.00381492051076338 |
| K09789 | neg\_1649 | 0.806060612201691 | 0.0048620605246823 |
| K13014 | neg\_1649 | 0.818181812763214 | 0.00381492051076338 |
| K04748 | neg\_1649 | -0.806060612201691 | 0.0048620605246823 |
| K18692 | neg\_1649 | -0.830303013324738 | 0.00294022813612127 |
| K17680 | neg\_1649 | -0.806060612201691 | 0.0048620605246823 |
| K03415 | neg\_1649 | -0.866666674613953 | 0.0011735379121256 |
| K03651 | neg\_1717 | -0.830303013324738 | 0.00294022813612127 |
| K12072 | neg\_1717 | 0.862921476364136 | 0.00130486922968087 |
| K02665 | neg\_1717 | 0.833002269268036 | 0.00276710271221536 |
| K21217 | neg\_1717 | 0.887625336647034 | 0.000607926148504134 |
| K19157 | neg\_1717 | -0.818181812763214 | 0.00381492051076338 |
| K06871 | neg\_1717 | -0.842424213886261 | 0.00222003275351312 |
| K16014 | neg\_1717 | -0.906692802906036 | 0.000295911879524491 |
| K06145 | neg\_1717 | -0.878787875175476 | 0.000813862205061078 |
| K03414 | neg\_1717 | 0.846658051013947 | 0.00200163182665913 |
| K10942 | neg\_1717 | 0.846658051013947 | 0.00200163182665913 |
| K03470 | neg\_1718 | -0.856668412685394 | 0.00154747182074688 |
| K09761 | neg\_1718 | -0.925451993942261 | 0.000123404671922511 |
| K00059 | neg\_1718 | -0.806643962860107 | 0.00480744414521128 |
| K00783 | neg\_1718 | -0.875427544116974 | 0.000904110124716251 |
| K03501 | neg\_1718 | -0.881680607795715 | 0.000741594922435862 |
| K01990 | neg\_1718 | -0.83790922164917 | 0.00247136195299369 |
| K03101 | neg\_1718 | -0.956717252731323 | 1.45713471533249e-05 |
| K06287 | neg\_1718 | -0.912945866584778 | 0.000225958764512368 |
| K11068 | neg\_1718 | -0.894186735153198 | 0.000481835628326488 |
| K00969 | neg\_1718 | -0.925451993942261 | 0.000123404671922511 |
| K00945 | neg\_1718 | -0.90043979883194 | 0.000380598613888061 |
| K03218 | neg\_1718 | -0.856668412685394 | 0.00154747182074688 |
| K05970 | neg\_1718 | -0.850415349006653 | 0.00182121334938934 |
| K00981 | neg\_1718 | -0.931705057621002 | 8.75967112814457e-05 |
| K03584 | neg\_1718 | -0.906692802906036 | 0.000295911879524491 |
| K01409 | neg\_1718 | -0.806643962860107 | 0.00480744414521128 |
| K00919 | neg\_1718 | -0.894186735153198 | 0.000481835628326488 |
| K02784 | neg\_1727 | -0.818181812763214 | 0.00381492051076338 |
| K12072 | neg\_1727 | 0.81915009021759 | 0.00373894966455346 |
| K02665 | neg\_1727 | 0.826174378395081 | 0.00321991677364641 |
| K16014 | neg\_1727 | -0.850415349006653 | 0.00182121334938934 |
| K06145 | neg\_1727 | -0.830303013324738 | 0.00294022813612127 |
| K20452 | neg\_1737 | 0.865095794200897 | 0.00122739260821847 |
| K18298 | neg\_1737 | 0.939393937587738 | 5.48405364009241e-05 |
| K11933 | neg\_176 | 0.938721001148224 | 5.72697606402439e-05 |
| K10237 | neg\_176 | 0.808135211467743 | 0.00466981917809761 |
| K03524 | neg\_176 | -0.806060612201691 | 0.0048620605246823 |
| K04096 | neg\_176 | -0.830303013324738 | 0.00294022813612127 |
| K04069 | neg\_176 | -0.806060612201691 | 0.0048620605246823 |
| K00817 | neg\_176 | -0.830303013324738 | 0.00294022813612127 |
| K02458 | neg\_176 | 0.938721001148224 | 5.72697606402439e-05 |
| K03607 | neg\_176 | 0.805690705776215 | 0.00489692183645341 |
| K01483 | neg\_176 | 0.818181812763214 | 0.00381492051076338 |
| K02521 | neg\_176 | 0.927272737026215 | 0.000112034447641074 |
| K00014 | neg\_176 | -0.939393937587738 | 5.48405364009241e-05 |
| K18537 | neg\_176 | 0.866666674613953 | 0.0011735379121256 |
| K05851 | neg\_176 | 0.812897026538849 | 0.00424919679863534 |
| K04486 | neg\_176 | -0.806060612201691 | 0.0048620605246823 |
| K14051 | neg\_176 | -0.826174378395081 | 0.00321991677364641 |
| K03724 | neg\_176 | -0.830303013324738 | 0.00294022813612127 |
| K06896 | neg\_176 | -0.903030276298523 | 0.000343612565232743 |
| K01060 | neg\_1781 | 0.830303013324738 | 0.00294022813612127 |
| K11933 | neg\_1781 | -0.975533545017242 | 1.52213781534627e-06 |
| K07099 | neg\_1781 | 0.806060612201691 | 0.0048620605246823 |
| K04075 | neg\_1781 | 0.806060612201691 | 0.0048620605246823 |
| K07391 | neg\_1781 | 0.806060612201691 | 0.0048620605246823 |
| K10237 | neg\_1781 | -0.885716199874878 | 0.000648751005291714 |
| K03524 | neg\_1781 | 0.866666674613953 | 0.0011735379121256 |
| K04096 | neg\_1781 | 0.927272737026215 | 0.000112034447641074 |
| K02461 | neg\_1781 | -0.800390899181366 | 0.0054162086926719 |
| K04069 | neg\_1781 | 0.842424213886261 | 0.00222003275351312 |
| K09779 | neg\_1781 | 0.806060612201691 | 0.0048620605246823 |
| K00817 | neg\_1781 | 0.854545474052429 | 0.00163680247839526 |
| K06285 | neg\_1781 | -0.854545474052429 | 0.00163680247839526 |
| K02458 | neg\_1781 | -0.889637529850006 | 0.000566964581027118 |
| K04565 | neg\_1781 | 0.915151536464691 | 0.000204472206099204 |
| K07031 | neg\_1781 | -0.818181812763214 | 0.00381492051076338 |
| K03607 | neg\_1781 | -0.805690705776215 | 0.00489692183645341 |
| K03643 | neg\_1781 | 0.806060612201691 | 0.0048620605246823 |
| K00849 | neg\_1781 | 0.854545474052429 | 0.00163680247839526 |
| K00014 | neg\_1781 | 0.818181812763214 | 0.00381492051076338 |
| K18537 | neg\_1781 | -0.878787875175476 | 0.000813862205061078 |
| K07720 | neg\_1781 | 0.830303013324738 | 0.00294022813612127 |
| K05851 | neg\_1781 | -0.881680607795715 | 0.000741594922435862 |
| K14051 | neg\_1781 | 0.873969614505768 | 0.000945449010965937 |
| K03724 | neg\_1781 | 0.830303013324738 | 0.00294022813612127 |
| K07139 | neg\_1791 | 0.830303013324738 | 0.00294022813612127 |
| K05787 | neg\_1791 | 0.866666674613953 | 0.0011735379121256 |
| K00059 | neg\_1791 | 0.830303013324738 | 0.00294022813612127 |
| K08688 | neg\_1791 | 0.806060612201691 | 0.0048620605246823 |
| K00588 | neg\_1791 | -0.852824926376343 | 0.00171189540742711 |
| K06926 | neg\_1791 | 0.818181812763214 | 0.00381492051076338 |
| K09951 | neg\_1791 | 0.830303013324738 | 0.00294022813612127 |
| K07672 | neg\_1796 | 0.812897026538849 | 0.00424919679863534 |
| K01060 | neg\_1796 | 0.806060612201691 | 0.0048620605246823 |
| K03827 | neg\_1796 | -0.854545474052429 | 0.00163680247839526 |
| K01261 | neg\_1796 | 0.854545474052429 | 0.00163680247839526 |
| K03406 | neg\_1796 | -0.830303013324738 | 0.00294022813612127 |
| K03225 | neg\_1796 | -0.915151536464691 | 0.000204472206099204 |
| K09789 | neg\_1796 | 0.866666674613953 | 0.0011735379121256 |
| K07039 | neg\_1796 | 0.818181812763214 | 0.00381492051076338 |
| K16199 | neg\_1796 | 0.806060612201691 | 0.0048620605246823 |
| K15652 | neg\_1796 | 0.927272737026215 | 0.000112034447641074 |
| K20885 | neg\_1796 | 0.854545474052429 | 0.00163680247839526 |
| K04085 | neg\_1796 | -1 | 0 |
| K00230 | neg\_1796 | 0.842424213886261 | 0.00222003275351312 |
| K07341 | neg\_1796 | 0.903030276298523 | 0.000343612565232743 |
| K03415 | neg\_1796 | -0.806060612201691 | 0.0048620605246823 |
| K01060 | neg\_1798 | 0.878787875175476 | 0.000813862205061078 |
| K11933 | neg\_1798 | -0.914179265499115 | 0.000213749081869885 |
| K17329 | neg\_1798 | -0.821065366268158 | 0.00359188206024896 |
| K04096 | neg\_1798 | 0.842424213886261 | 0.00222003275351312 |
| K00817 | neg\_1798 | 0.806060612201691 | 0.0048620605246823 |
| K03412 | neg\_1798 | -0.818181812763214 | 0.00381492051076338 |
| K06285 | neg\_1798 | -0.866666674613953 | 0.0011735379121256 |
| K02458 | neg\_1798 | -0.816012322902679 | 0.0039891464803441 |
| K04565 | neg\_1798 | 0.830303013324738 | 0.00294022813612127 |
| K07031 | neg\_1798 | -0.903030276298523 | 0.000343612565232743 |
| K21029 | neg\_1798 | -0.866666674613953 | 0.0011735379121256 |
| K18537 | neg\_1798 | -0.854545474052429 | 0.00163680247839526 |
| K00887 | neg\_1798 | -0.866666674613953 | 0.0011735379121256 |
| K07720 | neg\_1798 | 0.806060612201691 | 0.0048620605246823 |
| K14051 | neg\_1798 | 0.873969614505768 | 0.000945449010965937 |
| K07574 | neg\_1798 | 0.806060612201691 | 0.0048620605246823 |
| K11933 | neg\_1804 | -0.861575186252594 | 0.00135458546448364 |
| K10237 | neg\_1804 | -0.810595333576202 | 0.00444896679095952 |
| K02461 | neg\_1804 | -0.871820390224457 | 0.00100887727685151 |
| K03651 | neg\_1804 | 0.814593434333801 | 0.00410613103846336 |
| K21217 | neg\_1804 | -0.814992010593414 | 0.00407302440095103 |
| K19157 | neg\_1804 | 0.881463050842285 | 0.000746860533753502 |
| K03414 | neg\_1804 | -0.86978143453598 | 0.00107186247146052 |
| K05802 | neg\_1804 | -0.933805823326111 | 7.75059745339313e-05 |
| K10942 | neg\_1804 | -0.86978143453598 | 0.00107186247146052 |
| K06311 | neg\_1804 | -0.884146332740784 | 0.00068379278040176 |
| K02919 | neg\_1804 | -0.899700224399567 | 0.000391673296721073 |
| K08714 | neg\_1852 | 0.906692802906036 | 0.000295911879524491 |
| K01159 | neg\_1852 | 0.856668412685394 | 0.00154747182074688 |
| K03470 | neg\_1852 | 0.844162285327911 | 0.00212839548320387 |
| K07391 | neg\_1852 | 0.806643962860107 | 0.00480744414521128 |
| K04061 | neg\_1852 | -0.83790922164917 | 0.00247136195299369 |
| K03524 | neg\_1852 | 0.844162285327911 | 0.00212839548320387 |
| K07126 | neg\_1852 | 0.800390899181366 | 0.0054162086926719 |
| K06167 | neg\_1852 | 0.844162285327911 | 0.00212839548320387 |
| K06285 | neg\_1852 | -0.81915009021759 | 0.00373894966455346 |
| K02852 | neg\_1852 | 0.856668412685394 | 0.00154747182074688 |
| K04565 | neg\_1852 | 0.831656157970428 | 0.00285249465359505 |
| K07139 | neg\_1852 | 0.937958121299744 | 6.01189982394335e-05 |
| K05787 | neg\_1852 | 0.956717252731323 | 1.45713471533249e-05 |
| K00059 | neg\_1852 | 0.844162285327911 | 0.00212839548320387 |
| K08688 | neg\_1852 | 0.806643962860107 | 0.00480744414521128 |
| K03643 | neg\_1852 | 0.831656157970428 | 0.00285249465359505 |
| K06179 | neg\_1852 | 0.856668412685394 | 0.00154747182074688 |
| K06926 | neg\_1852 | 0.90043979883194 | 0.000380598613888061 |
| K00783 | neg\_1852 | 0.869174480438232 | 0.00109115185763287 |
| K03101 | neg\_1852 | 0.81915009021759 | 0.00373894966455346 |
| K11068 | neg\_1852 | 0.894186735153198 | 0.000481835628326488 |
| K05851 | neg\_1852 | -0.800000011920929 | 0.00545599878335024 |
| K14575 | neg\_1852 | 0.903180837631226 | 0.000341547437214818 |
| K03584 | neg\_1852 | 0.800390899181366 | 0.0054162086926719 |
| K14051 | neg\_1852 | 0.90172153711319 | 0.000361951655085679 |
| K09951 | neg\_1852 | 0.831656157970428 | 0.00285249465359505 |
| K08714 | neg\_1863 | 0.890909075737 | 0.000542144516154419 |
| K01159 | neg\_1863 | 0.878787875175476 | 0.000813862205061078 |
| K04061 | neg\_1863 | -0.806060612201691 | 0.0048620605246823 |
| K07126 | neg\_1863 | 0.842424213886261 | 0.00222003275351312 |
| K12543 | neg\_1863 | -0.842424213886261 | 0.00222003275351312 |
| K07031 | neg\_1863 | -0.903030276298523 | 0.000343612565232743 |
| K07139 | neg\_1863 | 0.818181812763214 | 0.00381492051076338 |
| K05787 | neg\_1863 | 0.854545474052429 | 0.00163680247839526 |
| K00059 | neg\_1863 | 0.830303013324738 | 0.00294022813612127 |
| K00588 | neg\_1863 | -0.889637529850006 | 0.000566964581027118 |
| K06926 | neg\_1863 | 0.866666674613953 | 0.0011735379121256 |
| K09512 | neg\_1863 | 0.821065366268158 | 0.00359188206024896 |
| K18298 | neg\_1863 | -0.806060612201691 | 0.0048620605246823 |
| K14051 | neg\_1863 | 0.826174378395081 | 0.00321991677364641 |
| K00057 | neg\_1863 | 0.818181812763214 | 0.00381492051076338 |
| K11754 | neg\_1874 | -0.830303013324738 | 0.00294022813612127 |
| K02784 | neg\_1874 | -0.806060612201691 | 0.0048620605246823 |
| K00059 | neg\_1874 | -0.830303013324738 | 0.00294022813612127 |
| K03501 | neg\_1874 | -0.830303013324738 | 0.00294022813612127 |
| K01990 | neg\_1874 | -0.818181812763214 | 0.00381492051076338 |
| K03101 | neg\_1874 | -0.830303013324738 | 0.00294022813612127 |
| K03218 | neg\_1874 | -0.806060612201691 | 0.0048620605246823 |
| K01060 | neg\_1886 | 0.854545474052429 | 0.00163680247839526 |
| K04069 | neg\_1886 | 0.830303013324738 | 0.00294022813612127 |
| K09779 | neg\_1886 | 0.806060612201691 | 0.0048620605246823 |
| K07166 | neg\_1886 | 0.806060612201691 | 0.0048620605246823 |
| K13798 | neg\_1886 | -0.806060612201691 | 0.0048620605246823 |
| K12231 | neg\_1886 | -0.819346487522125 | 0.00372367376291516 |
| K13611 | neg\_1886 | -0.833995580673218 | 0.00270528265085312 |
| K07720 | neg\_1886 | 0.842424213886261 | 0.00222003275351312 |
| K10578 | neg\_1886 | -0.873969614505768 | 0.000945449010965937 |
| K04486 | neg\_1886 | 0.878787875175476 | 0.000813862205061078 |
| K03724 | neg\_1886 | 0.854545474052429 | 0.00163680247839526 |
| K08688 | neg\_1901 | 0.878787875175476 | 0.000813862205061078 |
| K07043 | neg\_1901 | 0.830303013324738 | 0.00294022813612127 |
| K08302 | neg\_1901 | 0.866666674613953 | 0.0011735379121256 |
| K07031 | neg\_193 | -0.866666674613953 | 0.0011735379121256 |
| K21029 | neg\_193 | -0.866666674613953 | 0.0011735379121256 |
| K09512 | neg\_193 | 0.833995580673218 | 0.00270528265085312 |
| K11933 | neg\_1993 | -0.840554058551788 | 0.00232177976116144 |
| K00817 | neg\_1993 | 0.854545474052429 | 0.00163680247839526 |
| K02745 | neg\_1993 | -0.806060612201691 | 0.0048620605246823 |
| K04565 | neg\_1993 | 0.842424213886261 | 0.00222003275351312 |
| K21217 | neg\_1993 | -0.805690705776215 | 0.00489692183645341 |
| K18692 | neg\_1993 | -0.842424213886261 | 0.00222003275351312 |
| K01524 | neg\_1993 | 0.927272737026215 | 0.000112034447641074 |
| K03414 | neg\_1993 | -0.873969614505768 | 0.000945449010965937 |
| K05851 | neg\_1993 | -0.90043979883194 | 0.000380598613888061 |
| K10942 | neg\_1993 | -0.873969614505768 | 0.000945449010965937 |
| K03724 | neg\_1993 | 0.818181812763214 | 0.00381492051076338 |
| K11933 | neg\_1996 | -0.889637529850006 | 0.000566964581027118 |
| K10237 | neg\_1996 | -0.833995580673218 | 0.00270528265085312 |
| K02461 | neg\_1996 | -0.881680607795715 | 0.000741594922435862 |
| K02458 | neg\_1996 | -0.840554058551788 | 0.00232177976116144 |
| K21217 | neg\_1996 | -0.805690705776215 | 0.00489692183645341 |
| K19157 | neg\_1996 | 0.842424213886261 | 0.00222003275351312 |
| K18537 | neg\_1996 | -0.854545474052429 | 0.00163680247839526 |
| K03414 | neg\_1996 | -0.873969614505768 | 0.000945449010965937 |
| K05802 | neg\_1996 | -0.924506723880768 | 0.000129632545059266 |
| K05851 | neg\_1996 | -0.800390899181366 | 0.0054162086926719 |
| K10942 | neg\_1996 | -0.873969614505768 | 0.000945449010965937 |
| K06311 | neg\_1996 | -0.899700224399567 | 0.000391673296721073 |
| K02919 | neg\_1996 | -0.878787875175476 | 0.000813862205061078 |
| K00852 | neg\_1998 | -0.854545474052429 | 0.00163680247839526 |
| K10907 | neg\_1998 | -0.842424213886261 | 0.00222003275351312 |
| K00297 | neg\_1998 | -0.818181812763214 | 0.00381492051076338 |
| K01814 | neg\_1998 | -0.818181812763214 | 0.00381492051076338 |
| K02747 | neg\_1998 | 0.878787875175476 | 0.000813862205061078 |
| K07454 | neg\_1998 | -0.842424213886261 | 0.00222003275351312 |
| K18348 | neg\_1998 | -0.830303013324738 | 0.00294022813612127 |
| K03660 | neg\_1998 | -0.854545474052429 | 0.00163680247839526 |
| K03407 | neg\_1998 | 0.890909075737 | 0.000542144516154419 |
| K01191 | neg\_1998 | -0.818181812763214 | 0.00381492051076338 |
| K01151 | neg\_1998 | -0.830303013324738 | 0.00294022813612127 |
| K07039 | neg\_2032 | -0.830303013324738 | 0.00294022813612127 |
| K07396 | neg\_2032 | 0.806060612201691 | 0.0048620605246823 |
| K00765 | neg\_2032 | -0.854545474052429 | 0.00163680247839526 |
| K10953 | neg\_2032 | -0.937436878681183 | 6.21251750798635e-05 |
| K19784 | neg\_2032 | 0.914179265499115 | 0.000213749081869885 |
| K01661 | neg\_2032 | 0.866666674613953 | 0.0011735379121256 |
| K17329 | neg\_2038 | 0.833995580673218 | 0.00270528265085312 |
| K00100 | neg\_2038 | 0.818181812763214 | 0.00381492051076338 |
| K00817 | neg\_2038 | -0.818181812763214 | 0.00381492051076338 |
| K10907 | neg\_2038 | -0.878787875175476 | 0.000813862205061078 |
| K00931 | neg\_2038 | -0.927272737026215 | 0.000112034447641074 |
| K03769 | neg\_2038 | -0.830303013324738 | 0.00294022813612127 |
| K01814 | neg\_2038 | -0.951515138149261 | 2.27985739738035e-05 |
| K12072 | neg\_2038 | 0.919198930263519 | 0.00016900592052238 |
| K09815 | neg\_2038 | -0.903030276298523 | 0.000343612565232743 |
| K00013 | neg\_2038 | -0.842424213886261 | 0.00222003275351312 |
| K07121 | neg\_2038 | 0.854545474052429 | 0.00163680247839526 |
| K13665 | neg\_2038 | 0.806060612201691 | 0.0048620605246823 |
| K07454 | neg\_2038 | -0.806060612201691 | 0.0048620605246823 |
| K06871 | neg\_2038 | -0.830303013324738 | 0.00294022813612127 |
| K02778 | neg\_2038 | 0.854545474052429 | 0.00163680247839526 |
| K03407 | neg\_2038 | 0.915151536464691 | 0.000204472206099204 |
| K05836 | neg\_2038 | -0.806060612201691 | 0.0048620605246823 |
| K00009 | neg\_2038 | -0.878787875175476 | 0.000813862205061078 |
| K01649 | neg\_2038 | -0.830303013324738 | 0.00294022813612127 |
| K11933 | neg\_2056 | -0.888198733329773 | 0.000596040365381301 |
| K10237 | neg\_2056 | -0.922830998897552 | 0.000141238827938484 |
| K04096 | neg\_2056 | 0.80374151468277 | 0.00508357521208702 |
| K02461 | neg\_2056 | -0.879905462265015 | 0.000785360184749173 |
| K02458 | neg\_2056 | -0.838509321212769 | 0.00243683023127983 |
| K04565 | neg\_2056 | 0.828283190727234 | 0.00307477558489877 |
| K03607 | neg\_2056 | -0.843286633491516 | 0.00217421422639408 |
| K02521 | neg\_2056 | -0.816012322902679 | 0.0039891464803441 |
| K18537 | neg\_2056 | -0.914179265499115 | 0.000213749081869885 |
| K03414 | neg\_2056 | -0.822550058364868 | 0.00348076923888341 |
| K10942 | neg\_2056 | -0.822550058364868 | 0.00348076923888341 |
| K06311 | neg\_2056 | -0.80003410577774 | 0.00545251995569918 |
| K03724 | neg\_2056 | 0.852824926376343 | 0.00171189540742711 |
| K02919 | neg\_2056 | -0.828283190727234 | 0.00307477558489877 |
| K11933 | neg\_2068 | -0.840554058551788 | 0.00232177976116144 |
| K17329 | neg\_2068 | -0.833995580673218 | 0.00270528265085312 |
| K04061 | neg\_2068 | -0.830303013324738 | 0.00294022813612127 |
| K04096 | neg\_2068 | 0.842424213886261 | 0.00222003275351312 |
| K00817 | neg\_2068 | 0.842424213886261 | 0.00222003275351312 |
| K19411 | neg\_2068 | 0.890909075737 | 0.000542144516154419 |
| K03412 | neg\_2068 | -0.842424213886261 | 0.00222003275351312 |
| K06285 | neg\_2068 | -0.890909075737 | 0.000542144516154419 |
| K04565 | neg\_2068 | 0.842424213886261 | 0.00222003275351312 |
| K03643 | neg\_2068 | 0.878787875175476 | 0.000813862205061078 |
| K06926 | neg\_2068 | 0.806060612201691 | 0.0048620605246823 |
| K13014 | neg\_2068 | 0.878787875175476 | 0.000813862205061078 |
| K01524 | neg\_2068 | 0.878787875175476 | 0.000813862205061078 |
| K03414 | neg\_2068 | -0.819346487522125 | 0.00372367376291516 |
| K05851 | neg\_2068 | -0.869174480438232 | 0.00109115185763287 |
| K10942 | neg\_2068 | -0.819346487522125 | 0.00372367376291516 |
| K14051 | neg\_2068 | 0.819346487522125 | 0.00372367376291516 |
| K11933 | neg\_207 | -0.80374151468277 | 0.00508357521208702 |
| K01524 | neg\_207 | 0.927272737026215 | 0.000112034447641074 |
| K03414 | neg\_207 | -0.860313832759857 | 0.00140239962667921 |
| K05802 | neg\_207 | -0.859855890274048 | 0.00142005806532719 |
| K05851 | neg\_207 | -0.844162285327911 | 0.00212839548320387 |
| K10942 | neg\_207 | -0.860313832759857 | 0.00140239962667921 |
| K00845 | neg\_2109 | 0.854545474052429 | 0.00163680247839526 |
| K05946 | neg\_2109 | 0.830303013324738 | 0.00294022813612127 |
| K07099 | neg\_2109 | 0.818181812763214 | 0.00381492051076338 |
| K03827 | neg\_2109 | -0.866666674613953 | 0.0011735379121256 |
| K03225 | neg\_2109 | -0.830303013324738 | 0.00294022813612127 |
| K17331 | neg\_2109 | 0.873969614505768 | 0.000945449010965937 |
| K07039 | neg\_2109 | 0.890909075737 | 0.000542144516154419 |
| K00949 | neg\_2109 | 0.866666674613953 | 0.0011735379121256 |
| K15599 | neg\_2109 | -0.806060612201691 | 0.0048620605246823 |
| K14170 | neg\_2109 | 0.806060612201691 | 0.0048620605246823 |
| K07396 | neg\_2109 | -0.903030276298523 | 0.000343612565232743 |
| K05985 | neg\_2109 | 0.842424213886261 | 0.00222003275351312 |
| K12231 | neg\_2109 | -0.839830160140991 | 0.00236205253090405 |
| K05989 | neg\_2109 | 0.854545474052429 | 0.00163680247839526 |
| K09777 | neg\_2109 | 0.830303013324738 | 0.00294022813612127 |
| K16199 | neg\_2109 | 0.830303013324738 | 0.00294022813612127 |
| K15652 | neg\_2109 | 0.878787875175476 | 0.000813862205061078 |
| K04085 | neg\_2109 | -0.830303013324738 | 0.00294022813612127 |
| K07707 | neg\_2109 | -0.842424213886261 | 0.00222003275351312 |
| K08714 | neg\_2116 | 0.842424213886261 | 0.00222003275351312 |
| K01060 | neg\_2116 | 0.927272737026215 | 0.000112034447641074 |
| K05946 | neg\_2116 | 0.854545474052429 | 0.00163680247839526 |
| K11933 | neg\_2116 | -0.865095794200897 | 0.00122739260821847 |
| K07099 | neg\_2116 | 0.854545474052429 | 0.00163680247839526 |
| K07391 | neg\_2116 | 0.830303013324738 | 0.00294022813612127 |
| K10237 | neg\_2116 | -0.821065366268158 | 0.00359188206024896 |
| K04061 | neg\_2116 | -0.806060612201691 | 0.0048620605246823 |
| K01714 | neg\_2116 | 0.878787875175476 | 0.000813862205061078 |
| K04096 | neg\_2116 | 0.806060612201691 | 0.0048620605246823 |
| K04069 | neg\_2116 | 0.903030276298523 | 0.000343612565232743 |
| K09779 | neg\_2116 | 0.842424213886261 | 0.00222003275351312 |
| K02745 | neg\_2116 | -0.818181812763214 | 0.00381492051076338 |
| K07166 | neg\_2116 | 0.927272737026215 | 0.000112034447641074 |
| K00852 | neg\_2116 | 0.866666674613953 | 0.0011735379121256 |
| K03412 | neg\_2116 | -0.806060612201691 | 0.0048620605246823 |
| K04565 | neg\_2116 | 0.842424213886261 | 0.00222003275351312 |
| K13798 | neg\_2116 | -0.854545474052429 | 0.00163680247839526 |
| K05787 | neg\_2116 | 0.818181812763214 | 0.00381492051076338 |
| K12231 | neg\_2116 | -0.846658051013947 | 0.00200163182665913 |
| K01295 | neg\_2116 | 0.842424213886261 | 0.00222003275351312 |
| K18348 | neg\_2116 | 0.806060612201691 | 0.0048620605246823 |
| K13611 | neg\_2116 | -0.808135211467743 | 0.00466981917809761 |
| K00887 | neg\_2116 | -0.818181812763214 | 0.00381492051076338 |
| K07720 | neg\_2116 | 0.890909075737 | 0.000542144516154419 |
| K10578 | neg\_2116 | -0.887625336647034 | 0.000607926148504134 |
| K07493 | neg\_2116 | -0.825403153896332 | 0.00327420560319291 |
| K04486 | neg\_2116 | 0.878787875175476 | 0.000813862205061078 |
| K14051 | neg\_2116 | 0.846658051013947 | 0.00200163182665913 |
| K03724 | neg\_2116 | 0.927272737026215 | 0.000112034447641074 |
| K07574 | neg\_2116 | 0.806060612201691 | 0.0048620605246823 |
| K17329 | neg\_2134 | -0.859855890274048 | 0.00142005806532719 |
| K02461 | neg\_2134 | -0.856668412685394 | 0.00154747182074688 |
| K03651 | neg\_2134 | 0.806060612201691 | 0.0048620605246823 |
| K01814 | neg\_2134 | 0.818181812763214 | 0.00381492051076338 |
| K12072 | neg\_2134 | -0.831656157970428 | 0.00285249465359505 |
| K00013 | neg\_2134 | 0.830303013324738 | 0.00294022813612127 |
| K02665 | neg\_2134 | -0.805690705776215 | 0.00489692183645341 |
| K19157 | neg\_2134 | 0.806060612201691 | 0.0048620605246823 |
| K04748 | neg\_2134 | -0.818181812763214 | 0.00381492051076338 |
| K16014 | neg\_2134 | 0.844162285327911 | 0.00212839548320387 |
| K05802 | neg\_2134 | -0.898646354675293 | 0.000407860267078952 |
| K06311 | neg\_2134 | -0.808514356613159 | 0.00463528246178302 |
| K01649 | neg\_2134 | 0.830303013324738 | 0.00294022813612127 |
| K02493 | neg\_2135 | 0.854545474052429 | 0.00163680247839526 |
| K02784 | neg\_2135 | 0.878787875175476 | 0.000813862205061078 |
| K03769 | neg\_2135 | 0.818181812763214 | 0.00381492051076338 |
| K20525 | neg\_2135 | 0.806060612201691 | 0.0048620605246823 |
| K02461 | neg\_2157 | -0.812897026538849 | 0.00424919679863534 |
| K19157 | neg\_2157 | 0.854545474052429 | 0.00163680247839526 |
| K03414 | neg\_2157 | -0.819346487522125 | 0.00372367376291516 |
| K05802 | neg\_2157 | -0.924506723880768 | 0.000129632545059266 |
| K10942 | neg\_2157 | -0.819346487522125 | 0.00372367376291516 |
| K06311 | neg\_2157 | -0.887542068958282 | 0.000609666476501403 |
| K02919 | neg\_2157 | -0.806060612201691 | 0.0048620605246823 |
| K08714 | neg\_2164 | 0.806060612201691 | 0.0048620605246823 |
| K01060 | neg\_2164 | 0.878787875175476 | 0.000813862205061078 |
| K11933 | neg\_2164 | -0.950991809368134 | 2.37837330223645e-05 |
| K10237 | neg\_2164 | -0.846925735473633 | 0.0019883679465349 |
| K03524 | neg\_2164 | 0.806060612201691 | 0.0048620605246823 |
| K01714 | neg\_2164 | 0.830303013324738 | 0.00294022813612127 |
| K04096 | neg\_2164 | 0.854545474052429 | 0.00163680247839526 |
| K04069 | neg\_2164 | 0.842424213886261 | 0.00222003275351312 |
| K00817 | neg\_2164 | 0.830303013324738 | 0.00294022813612127 |
| K06285 | neg\_2164 | -0.806060612201691 | 0.0048620605246823 |
| K02458 | neg\_2164 | -0.865095794200897 | 0.00122739260821847 |
| K04565 | neg\_2164 | 0.854545474052429 | 0.00163680247839526 |
| K07031 | neg\_2164 | -0.854545474052429 | 0.00163680247839526 |
| K21029 | neg\_2164 | -0.830303013324738 | 0.00294022813612127 |
| K12231 | neg\_2164 | -0.833002269268036 | 0.00276710271221536 |
| K02521 | neg\_2164 | -0.818181812763214 | 0.00381492051076338 |
| K00014 | neg\_2164 | 0.806060612201691 | 0.0048620605246823 |
| K18537 | neg\_2164 | -0.903030276298523 | 0.000343612565232743 |
| K00887 | neg\_2164 | -0.854545474052429 | 0.00163680247839526 |
| K07720 | neg\_2164 | 0.830303013324738 | 0.00294022813612127 |
| K10578 | neg\_2164 | -0.819346487522125 | 0.00372367376291516 |
| K05851 | neg\_2164 | -0.81915009021759 | 0.00373894966455346 |
| K14051 | neg\_2164 | 0.887625336647034 | 0.000607926148504134 |
| K03724 | neg\_2164 | 0.866666674613953 | 0.0011735379121256 |
| K07574 | neg\_2164 | 0.806060612201691 | 0.0048620605246823 |
| K11933 | neg\_2197 | -0.900621116161346 | 0.000377918936301569 |
| K17329 | neg\_2197 | -0.870471775531769 | 0.00105022593325454 |
| K10237 | neg\_2197 | -0.831202387809753 | 0.00288170263251697 |
| K04061 | neg\_2197 | -0.828283190727234 | 0.00307477558489877 |
| K04096 | neg\_2197 | 0.828283190727234 | 0.00307477558489877 |
| K02461 | neg\_2197 | -0.867244958877563 | 0.00115415224037485 |
| K07126 | neg\_2197 | 0.816012322902679 | 0.0039891464803441 |
| K03651 | neg\_2197 | 0.840554058551788 | 0.00232177976116144 |
| K04565 | neg\_2197 | 0.80374151468277 | 0.00508357521208702 |
| K02665 | neg\_2197 | -0.8156378865242 | 0.00401978302331507 |
| K05830 | neg\_2197 | -0.80374151468277 | 0.00508357521208702 |
| K21217 | neg\_2197 | -0.8156378865242 | 0.00401978302331507 |
| K06926 | neg\_2197 | 0.889637529850006 | 0.000566964581027118 |
| K19157 | neg\_2197 | 0.828283190727234 | 0.00307477558489877 |
| K04748 | neg\_2197 | -0.80374151468277 | 0.00508357521208702 |
| K06145 | neg\_2197 | 0.80374151468277 | 0.00508357521208702 |
| K03414 | neg\_2197 | -0.843286633491516 | 0.00217421422639408 |
| K05802 | neg\_2197 | -0.883561611175537 | 0.000697191219888094 |
| K05851 | neg\_2197 | -0.848254203796387 | 0.00192348015922827 |
| K10942 | neg\_2197 | -0.843286633491516 | 0.00217421422639408 |
| K14051 | neg\_2197 | 0.850198805332184 | 0.00183127870149313 |
| K03724 | neg\_2197 | 0.80374151468277 | 0.00508357521208702 |
| K02919 | neg\_2197 | -0.840554058551788 | 0.00232177976116144 |
| K06285 | neg\_2251 | -0.866666674613953 | 0.0011735379121256 |
| K03643 | neg\_2251 | 0.927272737026215 | 0.000112034447641074 |
| K14575 | neg\_2251 | 0.857146799564362 | 0.0015278405327328 |
| K01409 | neg\_2251 | 0.818181812763214 | 0.00381492051076338 |
| K11933 | neg\_2257 | -0.80618816614151 | 0.00485008058916403 |
| K17329 | neg\_2257 | -0.849503874778748 | 0.00186385232781783 |
| K02461 | neg\_2257 | -0.853004157543182 | 0.00170395875980178 |
| K03651 | neg\_2257 | 0.808514356613159 | 0.00463528246178302 |
| K02665 | neg\_2257 | -0.814992010593414 | 0.00407302440095103 |
| K19157 | neg\_2257 | 0.83890962600708 | 0.00241398974898654 |
| K03414 | neg\_2257 | -0.808143377304077 | 0.00466907341223743 |
| K05802 | neg\_2257 | -0.914351522922516 | 0.000212083364557358 |
| K10942 | neg\_2257 | -0.808143377304077 | 0.00466907341223743 |
| K09512 | neg\_2257 | 0.810595333576202 | 0.00444896679095952 |
| K06311 | neg\_2257 | -0.878048777580261 | 0.000833121234763867 |
| K02919 | neg\_2257 | -0.814593434333801 | 0.00410613103846336 |
| K11933 | neg\_2264 | 0.865095794200897 | 0.00122739260821847 |
| K10237 | neg\_2264 | 0.821065366268158 | 0.00359188206024896 |
| K04096 | neg\_2264 | -0.806060612201691 | 0.0048620605246823 |
| K02461 | neg\_2264 | 0.912945866584778 | 0.000225958764512368 |
| K02458 | neg\_2264 | 0.865095794200897 | 0.00122739260821847 |
| K04565 | neg\_2264 | -0.806060612201691 | 0.0048620605246823 |
| K21217 | neg\_2264 | 0.873969614505768 | 0.000945449010965937 |
| K19157 | neg\_2264 | -0.854545474052429 | 0.00163680247839526 |
| K03414 | neg\_2264 | 0.873969614505768 | 0.000945449010965937 |
| K05802 | neg\_2264 | 0.872786045074463 | 0.000980007503875058 |
| K05851 | neg\_2264 | 0.894186735153198 | 0.000481835628326488 |
| K10942 | neg\_2264 | 0.873969614505768 | 0.000945449010965937 |
| K06311 | neg\_2264 | 0.802435338497162 | 0.00521145970824932 |
| K01060 | neg\_2267 | 0.890909075737 | 0.000542144516154419 |
| K05946 | neg\_2267 | 0.818181812763214 | 0.00381492051076338 |
| K09779 | neg\_2267 | 0.818181812763214 | 0.00381492051076338 |
| K07166 | neg\_2267 | 0.818181812763214 | 0.00381492051076338 |
| K04565 | neg\_2267 | 0.806060612201691 | 0.0048620605246823 |
| K13798 | neg\_2267 | -0.830303013324738 | 0.00294022813612127 |
| K03607 | neg\_2267 | -0.819346487522125 | 0.00372367376291516 |
| K12231 | neg\_2267 | -0.887625336647034 | 0.000607926148504134 |
| K20452 | neg\_2267 | -0.865095794200897 | 0.00122739260821847 |
| K13611 | neg\_2267 | -0.808135211467743 | 0.00466981917809761 |
| K07720 | neg\_2267 | 0.806060612201691 | 0.0048620605246823 |
| K10578 | neg\_2267 | -0.846658051013947 | 0.00200163182665913 |
| K04486 | neg\_2267 | 0.806060612201691 | 0.0048620605246823 |
| K11933 | neg\_2280 | -0.849266946315765 | 0.00187505312445646 |
| K10237 | neg\_2280 | -0.849503874778748 | 0.00186385232781783 |
| K02461 | neg\_2280 | -0.896908760070801 | 0.000435612478490999 |
| K03651 | neg\_2280 | 0.802435338497162 | 0.00521145970824932 |
| K02458 | neg\_2280 | -0.80003410577774 | 0.00545251995569918 |
| K21217 | neg\_2280 | -0.828689396381378 | 0.00304736829502406 |
| K19157 | neg\_2280 | 0.881463050842285 | 0.000746860533753502 |
| K18537 | neg\_2280 | -0.826751530170441 | 0.00317971472634282 |
| K16014 | neg\_2280 | 0.821643710136414 | 0.00354830133511608 |
| K03414 | neg\_2280 | -0.883478760719299 | 0.000699105060886662 |
| K05802 | neg\_2280 | -0.920836269855499 | 0.000156033361973673 |
| K10942 | neg\_2280 | -0.883478760719299 | 0.000699105060886662 |
| K06311 | neg\_2280 | -0.896341443061829 | 0.000444965811158937 |
| K02919 | neg\_2280 | -0.875383973121643 | 0.00090532610126548 |
| K19157 | neg\_2282 | 0.842424213886261 | 0.00222003275351312 |
| K01524 | neg\_2282 | 0.806060612201691 | 0.0048620605246823 |
| K03414 | neg\_2282 | -0.860313832759857 | 0.00140239962667921 |
| K05802 | neg\_2282 | -0.937436878681183 | 6.21251750798635e-05 |
| K10942 | neg\_2282 | -0.860313832759857 | 0.00140239962667921 |
| K06311 | neg\_2282 | -0.893621146678925 | 0.000491873064334847 |
| K02919 | neg\_2282 | -0.806060612201691 | 0.0048620605246823 |
| K07043 | neg\_2298 | 0.842424213886261 | 0.00222003275351312 |
| K02493 | neg\_2310 | -0.927272737026215 | 0.000112034447641074 |
| K02784 | neg\_2310 | -0.830303013324738 | 0.00294022813612127 |
| K03769 | neg\_2310 | -0.878787875175476 | 0.000813862205061078 |
| K19334 | neg\_2310 | -0.806060612201691 | 0.0048620605246823 |
| K02665 | neg\_2310 | 0.826174378395081 | 0.00321991677364641 |
| K01409 | neg\_2310 | -0.842424213886261 | 0.00222003275351312 |
| K00919 | neg\_2310 | -0.806060612201691 | 0.0048620605246823 |
| K20525 | neg\_2310 | -0.842424213886261 | 0.00222003275351312 |
| K11933 | neg\_2373 | -0.80374151468277 | 0.00508357521208702 |
| K02461 | neg\_2373 | -0.806643962860107 | 0.00480744414521128 |
| K21217 | neg\_2373 | -0.805690705776215 | 0.00489692183645341 |
| K19157 | neg\_2373 | 0.806060612201691 | 0.0048620605246823 |
| K18692 | neg\_2373 | -0.806060612201691 | 0.0048620605246823 |
| K03414 | neg\_2373 | -0.873969614505768 | 0.000945449010965937 |
| K05802 | neg\_2373 | -0.885716199874878 | 0.000648751005291714 |
| K10942 | neg\_2373 | -0.873969614505768 | 0.000945449010965937 |
| K06311 | neg\_2373 | -0.942253589630127 | 4.53587077142714e-05 |
| K01261 | neg\_2383 | -0.866666674613953 | 0.0011735379121256 |
| K09789 | neg\_2383 | -0.818181812763214 | 0.00381492051076338 |
| K20452 | neg\_2383 | 0.816012322902679 | 0.0039891464803441 |
| K18298 | neg\_2383 | 0.878787875175476 | 0.000813862205061078 |
| K02491 | neg\_2383 | 0.889637529850006 | 0.000566964581027118 |
| K05946 | neg\_2400 | 0.866666674613953 | 0.0011735379121256 |
| K01159 | neg\_2400 | 0.830303013324738 | 0.00294022813612127 |
| K04061 | neg\_2400 | -0.890909075737 | 0.000542144516154419 |
| K01729 | neg\_2400 | 0.818181812763214 | 0.00381492051076338 |
| K09761 | neg\_2400 | 0.866666674613953 | 0.0011735379121256 |
| K04565 | neg\_2400 | 0.842424213886261 | 0.00222003275351312 |
| K07139 | neg\_2400 | 0.842424213886261 | 0.00222003275351312 |
| K05787 | neg\_2400 | 0.927272737026215 | 0.000112034447641074 |
| K20452 | neg\_2400 | -0.901908397674561 | 0.000359290276283897 |
| K00969 | neg\_2400 | 0.806060612201691 | 0.0048620605246823 |
| K14575 | neg\_2400 | 0.802435338497162 | 0.00521145970824932 |
| K05964 | neg\_2400 | -0.890909075737 | 0.000542144516154419 |
| K11933 | neg\_2415 | -0.886191606521606 | 0.000638403173477631 |
| K10237 | neg\_2415 | -0.849503874778748 | 0.00186385232781783 |
| K04096 | neg\_2415 | 0.802435338497162 | 0.00521145970824932 |
| K02461 | neg\_2415 | -0.896908760070801 | 0.000435612478490999 |
| K02458 | neg\_2415 | -0.836958706378937 | 0.00252677787751843 |
| K21217 | neg\_2415 | -0.828689396381378 | 0.00304736829502406 |
| K19157 | neg\_2415 | 0.869304955005646 | 0.00108698415655106 |
| K18537 | neg\_2415 | -0.83890962600708 | 0.00241398974898654 |
| K03414 | neg\_2415 | -0.883478760719299 | 0.000699105060886662 |
| K05802 | neg\_2415 | -0.920836269855499 | 0.000156033361973673 |
| K05851 | neg\_2415 | -0.815371572971344 | 0.00404167522068155 |
| K10942 | neg\_2415 | -0.883478760719299 | 0.000699105060886662 |
| K06311 | neg\_2415 | -0.896341443061829 | 0.000444965811158937 |
| K02919 | neg\_2415 | -0.887542068958282 | 0.000609666476501403 |
| K11933 | neg\_2446 | -0.80374151468277 | 0.00508357521208702 |
| K17329 | neg\_2446 | -0.808135211467743 | 0.00466981917809761 |
| K02461 | neg\_2446 | -0.894186735153198 | 0.000481835628326488 |
| K03651 | neg\_2446 | 0.842424213886261 | 0.00222003275351312 |
| K02665 | neg\_2446 | -0.819346487522125 | 0.00372367376291516 |
| K05830 | neg\_2446 | -0.818181812763214 | 0.00381492051076338 |
| K21217 | neg\_2446 | -0.846658051013947 | 0.00200163182665913 |
| K06926 | neg\_2446 | 0.830303013324738 | 0.00294022813612127 |
| K19157 | neg\_2446 | 0.939393937587738 | 5.48405364009241e-05 |
| K06145 | neg\_2446 | 0.806060612201691 | 0.0048620605246823 |
| K03414 | neg\_2446 | -0.846658051013947 | 0.00200163182665913 |
| K05802 | neg\_2446 | -0.898646354675293 | 0.000407860267078952 |
| K10942 | neg\_2446 | -0.846658051013947 | 0.00200163182665913 |
| K06311 | neg\_2446 | -0.83890962600708 | 0.00241398974898654 |
| K02919 | neg\_2446 | -0.866666674613953 | 0.0011735379121256 |
| K07031 | neg\_2456 | -0.806643962860107 | 0.00480744414521128 |
| K09512 | neg\_2456 | 0.953863382339478 | 1.87461202005856e-05 |
| K06139 | neg\_2456 | 0.825403153896332 | 0.00327420560319291 |
| K02461 | neg\_2461 | -0.850415349006653 | 0.00182121334938934 |
| K21217 | neg\_2461 | -0.833002269268036 | 0.00276710271221536 |
| K19157 | neg\_2461 | 0.890909075737 | 0.000542144516154419 |
| K03414 | neg\_2461 | -0.873969614505768 | 0.000945449010965937 |
| K05802 | neg\_2461 | -0.924506723880768 | 0.000129632545059266 |
| K10942 | neg\_2461 | -0.873969614505768 | 0.000945449010965937 |
| K06311 | neg\_2461 | -0.930095493793488 | 9.59607762942571e-05 |
| K02919 | neg\_2461 | -0.854545474052429 | 0.00163680247839526 |
| K11933 | neg\_2462 | -0.889637529850006 | 0.000566964581027118 |
| K17329 | neg\_2462 | -0.821065366268158 | 0.00359188206024896 |
| K10237 | neg\_2462 | -0.885716199874878 | 0.000648751005291714 |
| K02461 | neg\_2462 | -0.869174480438232 | 0.00109115185763287 |
| K03651 | neg\_2462 | 0.842424213886261 | 0.00222003275351312 |
| K21217 | neg\_2462 | -0.805690705776215 | 0.00489692183645341 |
| K19157 | neg\_2462 | 0.818181812763214 | 0.00381492051076338 |
| K04748 | neg\_2462 | -0.818181812763214 | 0.00381492051076338 |
| K18537 | neg\_2462 | -0.866666674613953 | 0.0011735379121256 |
| K18692 | neg\_2462 | -0.806060612201691 | 0.0048620605246823 |
| K06145 | neg\_2462 | 0.818181812763214 | 0.00381492051076338 |
| K03414 | neg\_2462 | -0.873969614505768 | 0.000945449010965937 |
| K05802 | neg\_2462 | -0.885716199874878 | 0.000648751005291714 |
| K10942 | neg\_2462 | -0.873969614505768 | 0.000945449010965937 |
| K06311 | neg\_2462 | -0.863225877285004 | 0.00129381457010247 |
| K03724 | neg\_2462 | 0.854545474052429 | 0.00163680247839526 |
| K02919 | neg\_2462 | -0.854545474052429 | 0.00163680247839526 |
| K00845 | neg\_2485 | 0.854545474052429 | 0.00163680247839526 |
| K01060 | neg\_2485 | 0.890909075737 | 0.000542144516154419 |
| K11933 | neg\_2485 | -0.889637529850006 | 0.000566964581027118 |
| K07099 | neg\_2485 | 0.818181812763214 | 0.00381492051076338 |
| K04075 | neg\_2485 | 0.806060612201691 | 0.0048620605246823 |
| K10237 | neg\_2485 | -0.808135211467743 | 0.00466981917809761 |
| K01714 | neg\_2485 | 0.878787875175476 | 0.000813862205061078 |
| K04069 | neg\_2485 | 0.903030276298523 | 0.000343612565232743 |
| K00817 | neg\_2485 | 0.866666674613953 | 0.0011735379121256 |
| K07166 | neg\_2485 | 0.854545474052429 | 0.00163680247839526 |
| K00852 | neg\_2485 | 0.830303013324738 | 0.00294022813612127 |
| K04565 | neg\_2485 | 0.818181812763214 | 0.00381492051076338 |
| K13798 | neg\_2485 | -0.842424213886261 | 0.00222003275351312 |
| K12231 | neg\_2485 | -0.833002269268036 | 0.00276710271221536 |
| K01295 | neg\_2485 | 0.842424213886261 | 0.00222003275351312 |
| K02521 | neg\_2485 | -0.806060612201691 | 0.0048620605246823 |
| K00014 | neg\_2485 | 0.818181812763214 | 0.00381492051076338 |
| K13611 | neg\_2485 | -0.821065366268158 | 0.00359188206024896 |
| K00887 | neg\_2485 | -0.842424213886261 | 0.00222003275351312 |
| K07720 | neg\_2485 | 0.890909075737 | 0.000542144516154419 |
| K10578 | neg\_2485 | -0.819346487522125 | 0.00372367376291516 |
| K05851 | neg\_2485 | -0.806643962860107 | 0.00480744414521128 |
| K04486 | neg\_2485 | 0.915151536464691 | 0.000204472206099204 |
| K14051 | neg\_2485 | 0.833002269268036 | 0.00276710271221536 |
| K03724 | neg\_2485 | 0.915151536464691 | 0.000204472206099204 |
| K07574 | neg\_2485 | 0.854545474052429 | 0.00163680247839526 |
| K06896 | neg\_2485 | 0.842424213886261 | 0.00222003275351312 |
| K01060 | neg\_2505 | 0.818181812763214 | 0.00381492051076338 |
| K11933 | neg\_2505 | -0.926450133323669 | 0.000117071096429999 |
| K17329 | neg\_2505 | -0.833995580673218 | 0.00270528265085312 |
| K07391 | neg\_2505 | 0.818181812763214 | 0.00381492051076338 |
| K10237 | neg\_2505 | -0.846925735473633 | 0.0019883679465349 |
| K04061 | neg\_2505 | -0.878787875175476 | 0.000813862205061078 |
| K04096 | neg\_2505 | 0.903030276298523 | 0.000343612565232743 |
| K04069 | neg\_2505 | 0.842424213886261 | 0.00222003275351312 |
| K00817 | neg\_2505 | 0.830303013324738 | 0.00294022813612127 |
| K02745 | neg\_2505 | -0.842424213886261 | 0.00222003275351312 |
| K00852 | neg\_2505 | 0.806060612201691 | 0.0048620605246823 |
| K19411 | neg\_2505 | 0.854545474052429 | 0.00163680247839526 |
| K06285 | neg\_2505 | -0.854545474052429 | 0.00163680247839526 |
| K04565 | neg\_2505 | 0.903030276298523 | 0.000343612565232743 |
| K03643 | neg\_2505 | 0.830303013324738 | 0.00294022813612127 |
| K06926 | neg\_2505 | 0.866666674613953 | 0.0011735379121256 |
| K01524 | neg\_2505 | 0.866666674613953 | 0.0011735379121256 |
| K03414 | neg\_2505 | -0.860313832759857 | 0.00140239962667921 |
| K05802 | neg\_2505 | -0.821065366268158 | 0.00359188206024896 |
| K05851 | neg\_2505 | -0.894186735153198 | 0.000481835628326488 |
| K10942 | neg\_2505 | -0.860313832759857 | 0.00140239962667921 |
| K14051 | neg\_2505 | 0.860313832759857 | 0.00140239962667921 |
| K03724 | neg\_2505 | 0.830303013324738 | 0.00294022813612127 |
| K01060 | neg\_2564 | 0.890909075737 | 0.000542144516154419 |
| K11933 | neg\_2564 | -0.840554058551788 | 0.00232177976116144 |
| K04069 | neg\_2564 | 0.830303013324738 | 0.00294022813612127 |
| K04565 | neg\_2564 | 0.830303013324738 | 0.00294022813612127 |
| K12231 | neg\_2564 | -0.833002269268036 | 0.00276710271221536 |
| K18537 | neg\_2564 | -0.842424213886261 | 0.00222003275351312 |
| K07720 | neg\_2564 | 0.842424213886261 | 0.00222003275351312 |
| K10578 | neg\_2564 | -0.819346487522125 | 0.00372367376291516 |
| K04486 | neg\_2564 | 0.854545474052429 | 0.00163680247839526 |
| K03724 | neg\_2564 | 0.854545474052429 | 0.00163680247839526 |
| K17329 | neg\_2570 | -0.821065366268158 | 0.00359188206024896 |
| K02461 | neg\_2570 | -0.825403153896332 | 0.00327420560319291 |
| K03651 | neg\_2570 | 0.854545474052429 | 0.00163680247839526 |
| K02665 | neg\_2570 | -0.833002269268036 | 0.00276710271221536 |
| K19157 | neg\_2570 | 0.903030276298523 | 0.000343612565232743 |
| K04748 | neg\_2570 | -0.818181812763214 | 0.00381492051076338 |
| K16014 | neg\_2570 | 0.844162285327911 | 0.00212839548320387 |
| K06145 | neg\_2570 | 0.830303013324738 | 0.00294022813612127 |
| K05802 | neg\_2570 | -0.898646354675293 | 0.000407860267078952 |
| K06311 | neg\_2570 | -0.851067781448364 | 0.00179112909994994 |
| K06167 | neg\_26 | -0.806060612201691 | 0.0048620605246823 |
| K02852 | neg\_26 | -0.830303013324738 | 0.00294022813612127 |
| K15045 | neg\_26 | -0.842424213886261 | 0.00222003275351312 |
| K03573 | neg\_26 | -0.830303013324738 | 0.00294022813612127 |
| K07043 | neg\_26 | -0.806060612201691 | 0.0048620605246823 |
| K05970 | neg\_26 | -0.842424213886261 | 0.00222003275351312 |
| K02566 | neg\_26 | -0.842424213886261 | 0.00222003275351312 |
| K01191 | neg\_26 | -0.818181812763214 | 0.00381492051076338 |
| K00845 | neg\_2627 | -0.818181812763214 | 0.00381492051076338 |
| K00640 | neg\_2627 | -0.806060612201691 | 0.0048620605246823 |
| K07672 | neg\_2627 | -0.862921476364136 | 0.00130486922968087 |
| K17329 | neg\_2627 | 0.833995580673218 | 0.00270528265085312 |
| K03827 | neg\_2627 | 0.903030276298523 | 0.000343612565232743 |
| K01261 | neg\_2627 | -0.842424213886261 | 0.00222003275351312 |
| K00100 | neg\_2627 | 0.890909075737 | 0.000542144516154419 |
| K19411 | neg\_2627 | -0.818181812763214 | 0.00381492051076338 |
| K03225 | neg\_2627 | 0.866666674613953 | 0.0011735379121256 |
| K10907 | neg\_2627 | -0.842424213886261 | 0.00222003275351312 |
| K03769 | neg\_2627 | -0.890909075737 | 0.000542144516154419 |
| K17331 | neg\_2627 | -0.873969614505768 | 0.000945449010965937 |
| K09815 | neg\_2627 | -0.842424213886261 | 0.00222003275351312 |
| K19334 | neg\_2627 | -0.878787875175476 | 0.000813862205061078 |
| K09789 | neg\_2627 | -0.866666674613953 | 0.0011735379121256 |
| K07121 | neg\_2627 | 0.903030276298523 | 0.000343612565232743 |
| K07039 | neg\_2627 | -0.842424213886261 | 0.00222003275351312 |
| K13665 | neg\_2627 | 0.903030276298523 | 0.000343612565232743 |
| K07396 | neg\_2627 | 0.818181812763214 | 0.00381492051076338 |
| K00344 | neg\_2627 | -0.806060612201691 | 0.0048620605246823 |
| K04748 | neg\_2627 | 0.806060612201691 | 0.0048620605246823 |
| K02778 | neg\_2627 | 0.842424213886261 | 0.00222003275351312 |
| K15652 | neg\_2627 | -0.842424213886261 | 0.00222003275351312 |
| K20885 | neg\_2627 | -0.806060612201691 | 0.0048620605246823 |
| K04085 | neg\_2627 | 0.927272737026215 | 0.000112034447641074 |
| K00230 | neg\_2627 | -0.866666674613953 | 0.0011735379121256 |
| K07341 | neg\_2627 | -0.830303013324738 | 0.00294022813612127 |
| K05836 | neg\_2627 | -0.927272737026215 | 0.000112034447641074 |
| K18198 | neg\_2643 | -0.866666674613953 | 0.0011735379121256 |
| K00640 | neg\_2643 | 0.818181812763214 | 0.00381492051076338 |
| K11933 | neg\_2643 | -0.852824926376343 | 0.00171189540742711 |
| K17329 | neg\_2643 | -0.821065366268158 | 0.00359188206024896 |
| K10237 | neg\_2643 | -0.885716199874878 | 0.000648751005291714 |
| K04096 | neg\_2643 | 0.818181812763214 | 0.00381492051076338 |
| K02461 | neg\_2643 | -0.869174480438232 | 0.00109115185763287 |
| K03651 | neg\_2643 | 0.830303013324738 | 0.00294022813612127 |
| K21217 | neg\_2643 | -0.805690705776215 | 0.00489692183645341 |
| K04748 | neg\_2643 | -0.806060612201691 | 0.0048620605246823 |
| K18537 | neg\_2643 | -0.830303013324738 | 0.00294022813612127 |
| K06145 | neg\_2643 | 0.830303013324738 | 0.00294022813612127 |
| K03414 | neg\_2643 | -0.873969614505768 | 0.000945449010965937 |
| K05802 | neg\_2643 | -0.885716199874878 | 0.000648751005291714 |
| K10942 | neg\_2643 | -0.873969614505768 | 0.000945449010965937 |
| K06311 | neg\_2643 | -0.924016416072845 | 0.000132952728702396 |
| K02919 | neg\_2643 | -0.818181812763214 | 0.00381492051076338 |
| K02745 | neg\_2684 | -0.83890962600708 | 0.00241398974898654 |
| K01814 | neg\_2684 | 0.857146799564362 | 0.0015278405327328 |
| K12072 | neg\_2684 | -0.834187865257263 | 0.00269343151576251 |
| K21217 | neg\_2684 | -0.828689396381378 | 0.00304736829502406 |
| K02747 | neg\_2684 | -0.820672512054443 | 0.00362170350001234 |
| K02501 | neg\_2684 | 0.924016416072845 | 0.000132952728702396 |
| K18692 | neg\_2684 | -0.857146799564362 | 0.0015278405327328 |
| K16014 | neg\_2684 | 0.834187865257263 | 0.00269343151576251 |
| K06145 | neg\_2684 | 0.826751530170441 | 0.00317971472634282 |
| K03407 | neg\_2684 | -0.863225877285004 | 0.00129381457010247 |
| K03414 | neg\_2684 | -0.883478760719299 | 0.000699105060886662 |
| K10942 | neg\_2684 | -0.883478760719299 | 0.000699105060886662 |
| K02746 | neg\_2684 | -0.875383973121643 | 0.00090532610126548 |
| K03724 | neg\_2684 | 0.851067781448364 | 0.00179112909994994 |
| K06305 | neg\_2684 | -0.802435338497162 | 0.00521145970824932 |
| K02493 | neg\_2705 | 0.866666674613953 | 0.0011735379121256 |
| K03769 | neg\_2705 | 0.806060612201691 | 0.0048620605246823 |
| K17331 | neg\_2705 | 0.887625336647034 | 0.000607926148504134 |
| K19334 | neg\_2705 | 0.830303013324738 | 0.00294022813612127 |
| K01990 | neg\_2705 | 0.806060612201691 | 0.0048620605246823 |
| K00344 | neg\_2705 | 0.854545474052429 | 0.00163680247839526 |
| K05970 | neg\_2705 | 0.818181812763214 | 0.00381492051076338 |
| K00919 | neg\_2705 | 0.818181812763214 | 0.00381492051076338 |
| K20525 | neg\_2705 | 0.854545474052429 | 0.00163680247839526 |
| K11933 | neg\_2738 | 0.840554058551788 | 0.00232177976116144 |
| K17329 | neg\_2738 | 0.859855890274048 | 0.00142005806532719 |
| K04061 | neg\_2738 | 0.830303013324738 | 0.00294022813612127 |
| K07126 | neg\_2738 | -0.842424213886261 | 0.00222003275351312 |
| K19411 | neg\_2738 | -0.806060612201691 | 0.0048620605246823 |
| K03651 | neg\_2738 | -0.854545474052429 | 0.00163680247839526 |
| K05830 | neg\_2738 | 0.818181812763214 | 0.00381492051076338 |
| K00588 | neg\_2738 | 0.80374151468277 | 0.00508357521208702 |
| K07454 | neg\_2738 | -0.854545474052429 | 0.00163680247839526 |
| K06926 | neg\_2738 | -0.890909075737 | 0.000542144516154419 |
| K04748 | neg\_2738 | 0.854545474052429 | 0.00163680247839526 |
| K00887 | neg\_2738 | 0.842424213886261 | 0.00222003275351312 |
| K05802 | neg\_2738 | 0.808135211467743 | 0.00466981917809761 |
| K14051 | neg\_2738 | -0.839830160140991 | 0.00236205253090405 |
| K02919 | neg\_2738 | 0.806060612201691 | 0.0048620605246823 |
| K01261 | neg\_2754 | 0.806060612201691 | 0.0048620605246823 |
| K20452 | neg\_2754 | -0.828283190727234 | 0.00307477558489877 |
| K18298 | neg\_2754 | -0.842424213886261 | 0.00222003275351312 |
| K02491 | neg\_2754 | -0.828283190727234 | 0.00307477558489877 |
| K08302 | neg\_2754 | 0.806060612201691 | 0.0048620605246823 |
| K02784 | neg\_2788 | -0.806060612201691 | 0.0048620605246823 |
| K09761 | neg\_2788 | -0.854545474052429 | 0.00163680247839526 |
| K01990 | neg\_2788 | -0.806060612201691 | 0.0048620605246823 |
| K00969 | neg\_2788 | -0.842424213886261 | 0.00222003275351312 |
| K01809 | neg\_2788 | -0.903030276298523 | 0.000343612565232743 |
| K00945 | neg\_2788 | -0.818181812763214 | 0.00381492051076338 |
| K05970 | neg\_2788 | -0.878787875175476 | 0.000813862205061078 |
| K05964 | neg\_2788 | 0.854545474052429 | 0.00163680247839526 |
| K02458 | neg\_2801 | 0.840554058551788 | 0.00232177976116144 |
| K07031 | neg\_2801 | 0.830303013324738 | 0.00294022813612127 |
| K21029 | neg\_2801 | 0.806060612201691 | 0.0048620605246823 |
| K18537 | neg\_2801 | 0.866666674613953 | 0.0011735379121256 |
| K09512 | neg\_2801 | -0.859855890274048 | 0.00142005806532719 |
| K02919 | neg\_2801 | 0.830303013324738 | 0.00294022813612127 |
| K05946 | neg\_2803 | 0.806060612201691 | 0.0048620605246823 |
| K01159 | neg\_2803 | 0.818181812763214 | 0.00381492051076338 |
| K04061 | neg\_2803 | -0.915151536464691 | 0.000204472206099204 |
| K01729 | neg\_2803 | 0.842424213886261 | 0.00222003275351312 |
| K07139 | neg\_2803 | 0.842424213886261 | 0.00222003275351312 |
| K05787 | neg\_2803 | 0.927272737026215 | 0.000112034447641074 |
| K20452 | neg\_2803 | -0.852824926376343 | 0.00171189540742711 |
| K06926 | neg\_2803 | 0.818181812763214 | 0.00381492051076338 |
| K18298 | neg\_2803 | -0.806060612201691 | 0.0048620605246823 |
| K05964 | neg\_2803 | -0.878787875175476 | 0.000813862205061078 |
| K01060 | neg\_2833 | 0.830303013324738 | 0.00294022813612127 |
| K11933 | neg\_2833 | -0.938721001148224 | 5.72697606402439e-05 |
| K10237 | neg\_2833 | -0.808135211467743 | 0.00466981917809761 |
| K03524 | neg\_2833 | 0.854545474052429 | 0.00163680247839526 |
| K04096 | neg\_2833 | 0.878787875175476 | 0.000813862205061078 |
| K04069 | neg\_2833 | 0.818181812763214 | 0.00381492051076338 |
| K09779 | neg\_2833 | 0.854545474052429 | 0.00163680247839526 |
| K00817 | neg\_2833 | 0.806060612201691 | 0.0048620605246823 |
| K02458 | neg\_2833 | -0.877366662025452 | 0.000851188393096614 |
| K04565 | neg\_2833 | 0.866666674613953 | 0.0011735379121256 |
| K07031 | neg\_2833 | -0.842424213886261 | 0.00222003275351312 |
| K12231 | neg\_2833 | -0.833002269268036 | 0.00276710271221536 |
| K01295 | neg\_2833 | 0.830303013324738 | 0.00294022813612127 |
| K01483 | neg\_2833 | -0.818181812763214 | 0.00381492051076338 |
| K00014 | neg\_2833 | 0.818181812763214 | 0.00381492051076338 |
| K18537 | neg\_2833 | -0.878787875175476 | 0.000813862205061078 |
| K07720 | neg\_2833 | 0.854545474052429 | 0.00163680247839526 |
| K10578 | neg\_2833 | -0.819346487522125 | 0.00372367376291516 |
| K05851 | neg\_2833 | -0.806643962860107 | 0.00480744414521128 |
| K14051 | neg\_2833 | 0.873969614505768 | 0.000945449010965937 |
| K07574 | neg\_2833 | 0.842424213886261 | 0.00222003275351312 |
| K00845 | neg\_2842 | 0.806060612201691 | 0.0048620605246823 |
| K00640 | neg\_2842 | 0.806060612201691 | 0.0048620605246823 |
| K01060 | neg\_2842 | 0.830303013324738 | 0.00294022813612127 |
| K05946 | neg\_2842 | 0.866666674613953 | 0.0011735379121256 |
| K11933 | neg\_2842 | -0.938721001148224 | 5.72697606402439e-05 |
| K07099 | neg\_2842 | 0.915151536464691 | 0.000204472206099204 |
| K04075 | neg\_2842 | 0.866666674613953 | 0.0011735379121256 |
| K03470 | neg\_2842 | 0.830303013324738 | 0.00294022813612127 |
| K07391 | neg\_2842 | 0.878787875175476 | 0.000813862205061078 |
| K10237 | neg\_2842 | -0.898646354675293 | 0.000407860267078952 |
| K04061 | neg\_2842 | -0.818181812763214 | 0.00381492051076338 |
| K03524 | neg\_2842 | 0.903030276298523 | 0.000343612565232743 |
| K04096 | neg\_2842 | 0.975757598876953 | 1.46754063035104e-06 |
| K04069 | neg\_2842 | 0.915151536464691 | 0.000204472206099204 |
| K09779 | neg\_2842 | 0.890909075737 | 0.000542144516154419 |
| K00817 | neg\_2842 | 0.866666674613953 | 0.0011735379121256 |
| K07166 | neg\_2842 | 0.842424213886261 | 0.00222003275351312 |
| K00852 | neg\_2842 | 0.842424213886261 | 0.00222003275351312 |
| K19411 | neg\_2842 | 0.842424213886261 | 0.00222003275351312 |
| K00800 | neg\_2842 | 0.866666674613953 | 0.0011735379121256 |
| K06285 | neg\_2842 | -0.866666674613953 | 0.0011735379121256 |
| K04565 | neg\_2842 | 0.951515138149261 | 2.27985739738035e-05 |
| K15599 | neg\_2842 | -0.806060612201691 | 0.0048620605246823 |
| K03643 | neg\_2842 | 0.830303013324738 | 0.00294022813612127 |
| K05985 | neg\_2842 | 0.806060612201691 | 0.0048620605246823 |
| K06926 | neg\_2842 | 0.818181812763214 | 0.00381492051076338 |
| K09777 | neg\_2842 | 0.830303013324738 | 0.00294022813612127 |
| K00859 | neg\_2842 | 0.854545474052429 | 0.00163680247839526 |
| K01295 | neg\_2842 | 0.866666674613953 | 0.0011735379121256 |
| K00849 | neg\_2842 | 0.915151536464691 | 0.000204472206099204 |
| K04517 | neg\_2842 | 0.903030276298523 | 0.000343612565232743 |
| K07720 | neg\_2842 | 0.915151536464691 | 0.000204472206099204 |
| K10578 | neg\_2842 | -0.805690705776215 | 0.00489692183645341 |
| K03414 | neg\_2842 | -0.826174378395081 | 0.00321991677364641 |
| K05851 | neg\_2842 | -0.894186735153198 | 0.000481835628326488 |
| K04486 | neg\_2842 | 0.818181812763214 | 0.00381492051076338 |
| K10942 | neg\_2842 | -0.826174378395081 | 0.00321991677364641 |
| K01736 | neg\_2842 | 0.818181812763214 | 0.00381492051076338 |
| K07053 | neg\_2842 | 0.818181812763214 | 0.00381492051076338 |
| K09775 | neg\_2842 | 0.866666674613953 | 0.0011735379121256 |
| K03724 | neg\_2842 | 0.830303013324738 | 0.00294022813612127 |
| K16898 | neg\_2842 | 0.866666674613953 | 0.0011735379121256 |
| K08714 | neg\_2851 | -0.818181812763214 | 0.00381492051076338 |
| K11754 | neg\_2851 | -0.842424213886261 | 0.00222003275351312 |
| K00100 | neg\_2851 | 0.842424213886261 | 0.00222003275351312 |
| K00297 | neg\_2851 | -0.818181812763214 | 0.00381492051076338 |
| K00765 | neg\_2851 | -0.830303013324738 | 0.00294022813612127 |
| K06179 | neg\_2851 | -0.818181812763214 | 0.00381492051076338 |
| K07454 | neg\_2851 | -0.890909075737 | 0.000542144516154419 |
| K06871 | neg\_2851 | -0.878787875175476 | 0.000813862205061078 |
| K00848 | neg\_2851 | -0.830303013324738 | 0.00294022813612127 |
| K00057 | neg\_2851 | -0.939393937587738 | 5.48405364009241e-05 |
| K01649 | neg\_2851 | -0.818181812763214 | 0.00381492051076338 |
| K17329 | neg\_286 | -0.860478162765503 | 0.00139610202996154 |
| K10237 | neg\_286 | -0.813785552978516 | 0.00417382592013071 |
| K02461 | neg\_286 | -0.935483872890472 | 7.00850078045878e-05 |
| K03651 | neg\_286 | 0.83790922164917 | 0.00247136195299369 |
| K12072 | neg\_286 | -0.800000011920929 | 0.00545599878335024 |
| K02665 | neg\_286 | -0.845363914966583 | 0.00206666064770777 |
| K21217 | neg\_286 | -0.845363914966583 | 0.00206666064770777 |
| K19157 | neg\_286 | 0.925451993942261 | 0.000123404671922511 |
| K16014 | neg\_286 | 0.845161318778992 | 0.00207697732308088 |
| K06145 | neg\_286 | 0.81915009021759 | 0.00373894966455346 |
| K03414 | neg\_286 | -0.845363914966583 | 0.00206666064770777 |
| K05802 | neg\_286 | -0.900500416755676 | 0.000379701199800131 |
| K10942 | neg\_286 | -0.845363914966583 | 0.00206666064770777 |
| K06311 | neg\_286 | -0.865548312664032 | 0.00121169851367053 |
| K02919 | neg\_286 | -0.806643962860107 | 0.00480744414521128 |
| K11933 | neg\_2886 | -0.80374151468277 | 0.00508357521208702 |
| K17329 | neg\_2886 | -0.808135211467743 | 0.00466981917809761 |
| K02461 | neg\_2886 | -0.894186735153198 | 0.000481835628326488 |
| K03651 | neg\_2886 | 0.842424213886261 | 0.00222003275351312 |
| K02665 | neg\_2886 | -0.819346487522125 | 0.00372367376291516 |
| K05830 | neg\_2886 | -0.818181812763214 | 0.00381492051076338 |
| K21217 | neg\_2886 | -0.846658051013947 | 0.00200163182665913 |
| K06926 | neg\_2886 | 0.830303013324738 | 0.00294022813612127 |
| K19157 | neg\_2886 | 0.939393937587738 | 5.48405364009241e-05 |
| K06145 | neg\_2886 | 0.806060612201691 | 0.0048620605246823 |
| K03414 | neg\_2886 | -0.846658051013947 | 0.00200163182665913 |
| K05802 | neg\_2886 | -0.898646354675293 | 0.000407860267078952 |
| K10942 | neg\_2886 | -0.846658051013947 | 0.00200163182665913 |
| K06311 | neg\_2886 | -0.83890962600708 | 0.00241398974898654 |
| K02919 | neg\_2886 | -0.866666674613953 | 0.0011735379121256 |
| K02461 | neg\_2892 | 0.806643962860107 | 0.00480744414521128 |
| K00588 | neg\_2892 | 0.828283190727234 | 0.00307477558489877 |
| K09512 | neg\_2892 | -0.846925735473633 | 0.0019883679465349 |
| K11933 | neg\_2932 | -0.816012322902679 | 0.0039891464803441 |
| K10237 | neg\_2932 | -0.808135211467743 | 0.00466981917809761 |
| K02461 | neg\_2932 | -0.875427544116974 | 0.000904110124716251 |
| K21217 | neg\_2932 | -0.846658051013947 | 0.00200163182665913 |
| K19157 | neg\_2932 | 0.903030276298523 | 0.000343612565232743 |
| K03414 | neg\_2932 | -0.887625336647034 | 0.000607926148504134 |
| K05802 | neg\_2932 | -0.911576509475708 | 0.000240107073119944 |
| K10942 | neg\_2932 | -0.887625336647034 | 0.000607926148504134 |
| K06311 | neg\_2932 | -0.911858320236206 | 0.000237143587637378 |
| K02919 | neg\_2932 | -0.854545474052429 | 0.00163680247839526 |
| K04565 | neg\_2965 | 0.808514356613159 | 0.00463528246178302 |
| K07139 | neg\_2965 | 0.802435338497162 | 0.00521145970824932 |
| K05787 | neg\_2965 | 0.875383973121643 | 0.00090532610126548 |
| K20452 | neg\_2965 | -0.972349107265472 | 2.47360780414319e-06 |
| K18298 | neg\_2965 | -0.857146799564362 | 0.0015278405327328 |
| K03225 | neg\_2972 | -0.842424213886261 | 0.00222003275351312 |
| K18692 | neg\_2972 | -0.818181812763214 | 0.00381492051076338 |
| K06871 | neg\_2973 | -0.866666674613953 | 0.0011735379121256 |
| K10907 | neg\_302 | -0.939393937587738 | 5.48405364009241e-05 |
| K00931 | neg\_302 | -0.903030276298523 | 0.000343612565232743 |
| K01814 | neg\_302 | -0.939393937587738 | 5.48405364009241e-05 |
| K12072 | neg\_302 | 0.831656157970428 | 0.00285249465359505 |
| K09815 | neg\_302 | -0.818181812763214 | 0.00381492051076338 |
| K00013 | neg\_302 | -0.830303013324738 | 0.00294022813612127 |
| K07121 | neg\_302 | 0.818181812763214 | 0.00381492051076338 |
| K18348 | neg\_302 | -0.806060612201691 | 0.0048620605246823 |
| K02778 | neg\_302 | 0.854545474052429 | 0.00163680247839526 |
| K02501 | neg\_302 | -0.806060612201691 | 0.0048620605246823 |
| K00887 | neg\_302 | 0.806060612201691 | 0.0048620605246823 |
| K03407 | neg\_302 | 0.927272737026215 | 0.000112034447641074 |
| K00009 | neg\_302 | -0.842424213886261 | 0.00222003275351312 |
| K01649 | neg\_302 | -0.806060612201691 | 0.0048620605246823 |
| K01661 | neg\_302 | 0.854545474052429 | 0.00163680247839526 |
| K17329 | neg\_3064 | -0.859855890274048 | 0.00142005806532719 |
| K02461 | neg\_3064 | -0.919198930263519 | 0.00016900592052238 |
| K03651 | neg\_3064 | 0.866666674613953 | 0.0011735379121256 |
| K02665 | neg\_3064 | -0.873969614505768 | 0.000945449010965937 |
| K05830 | neg\_3064 | -0.818181812763214 | 0.00381492051076338 |
| K21217 | neg\_3064 | -0.846658051013947 | 0.00200163182665913 |
| K19157 | neg\_3064 | 0.963636338710785 | 7.32099466027591e-06 |
| K06871 | neg\_3064 | 0.830303013324738 | 0.00294022813612127 |
| K16014 | neg\_3064 | 0.81915009021759 | 0.00373894966455346 |
| K06145 | neg\_3064 | 0.842424213886261 | 0.00222003275351312 |
| K05802 | neg\_3064 | -0.846925735473633 | 0.0019883679465349 |
| K17329 | neg\_3088 | -0.831202387809753 | 0.00288170263251697 |
| K02461 | neg\_3088 | -0.879905462265015 | 0.000785360184749173 |
| K03651 | neg\_3088 | 0.877366662025452 | 0.000851188393096614 |
| K02665 | neg\_3088 | -0.8156378865242 | 0.00401978302331507 |
| K05830 | neg\_3088 | -0.828283190727234 | 0.00307477558489877 |
| K21217 | neg\_3088 | -0.8156378865242 | 0.00401978302331507 |
| K19157 | neg\_3088 | 0.914179265499115 | 0.000213749081869885 |
| K04748 | neg\_3088 | -0.828283190727234 | 0.00307477558489877 |
| K16014 | neg\_3088 | 0.82926344871521 | 0.00300893631849064 |
| K06145 | neg\_3088 | 0.840554058551788 | 0.00232177976116144 |
| K03414 | neg\_3088 | -0.843286633491516 | 0.00217421422639408 |
| K05802 | neg\_3088 | -0.922830998897552 | 0.000141238827938484 |
| K10942 | neg\_3088 | -0.843286633491516 | 0.00217421422639408 |
| K06311 | neg\_3088 | -0.861575186252594 | 0.00135458546448364 |
| K02919 | neg\_3088 | -0.852824926376343 | 0.00171189540742711 |
| K02493 | neg\_3092 | 0.818181812763214 | 0.00381492051076338 |
| K02665 | neg\_3092 | -0.846658051013947 | 0.00200163182665913 |
| K21217 | neg\_3092 | -0.819346487522125 | 0.00372367376291516 |
| K03414 | neg\_3092 | -0.805690705776215 | 0.00489692183645341 |
| K05802 | neg\_3092 | -0.808135211467743 | 0.00466981917809761 |
| K10942 | neg\_3092 | -0.805690705776215 | 0.00489692183645341 |
| K04061 | neg\_3101 | 0.820672512054443 | 0.00362170350001234 |
| K01729 | neg\_3101 | -0.820672512054443 | 0.00362170350001234 |
| K05787 | neg\_3101 | -0.802435338497162 | 0.00521145970824932 |
| K00588 | neg\_3101 | 0.818496406078339 | 0.00379011736651003 |
| K18298 | neg\_3101 | 0.899700224399567 | 0.000391673296721073 |
| K07139 | neg\_3158 | 0.806060612201691 | 0.0048620605246823 |
| K05787 | neg\_3158 | 0.854545474052429 | 0.00163680247839526 |
| K20452 | neg\_3158 | -0.865095794200897 | 0.00122739260821847 |
| K18298 | neg\_3158 | -0.903030276298523 | 0.000343612565232743 |
| K02461 | neg\_3198 | -0.806643962860107 | 0.00480744414521128 |
| K01729 | neg\_3198 | 0.842424213886261 | 0.00222003275351312 |
| K04565 | neg\_3198 | 0.818181812763214 | 0.00381492051076338 |
| K02665 | neg\_3198 | -0.833002269268036 | 0.00276710271221536 |
| K21217 | neg\_3198 | -0.887625336647034 | 0.000607926148504134 |
| K03643 | neg\_3198 | 0.830303013324738 | 0.00294022813612127 |
| K19157 | neg\_3198 | 0.830303013324738 | 0.00294022813612127 |
| K03414 | neg\_3198 | -0.846658051013947 | 0.00200163182665913 |
| K05851 | neg\_3198 | -0.850415349006653 | 0.00182121334938934 |
| K10942 | neg\_3198 | -0.846658051013947 | 0.00200163182665913 |
| K14575 | neg\_3198 | 0.83890962600708 | 0.00241398974898654 |
| K09951 | neg\_3198 | 0.854545474052429 | 0.00163680247839526 |
| K11754 | neg\_3252 | -0.818181812763214 | 0.00381492051076338 |
| K06167 | neg\_3252 | -0.903030276298523 | 0.000343612565232743 |
| K01729 | neg\_3252 | -0.854545474052429 | 0.00163680247839526 |
| K02852 | neg\_3252 | -0.890909075737 | 0.000542144516154419 |
| K05787 | neg\_3252 | -0.806060612201691 | 0.0048620605246823 |
| K00783 | neg\_3252 | -0.818181812763214 | 0.00381492051076338 |
| K14575 | neg\_3252 | -0.832830607891083 | 0.00277788845123905 |
| K09951 | neg\_3252 | -0.806060612201691 | 0.0048620605246823 |
| K12543 | neg\_3294 | 0.830303013324738 | 0.00294022813612127 |
| K01060 | neg\_3351 | 0.903030276298523 | 0.000343612565232743 |
| K11933 | neg\_3351 | -0.877366662025452 | 0.000851188393096614 |
| K10237 | neg\_3351 | -0.846925735473633 | 0.0019883679465349 |
| K04565 | neg\_3351 | 0.830303013324738 | 0.00294022813612127 |
| K07031 | neg\_3351 | -0.806060612201691 | 0.0048620605246823 |
| K12231 | neg\_3351 | -0.833002269268036 | 0.00276710271221536 |
| K18537 | neg\_3351 | -0.951515138149261 | 2.27985739738035e-05 |
| K18692 | neg\_3351 | -0.842424213886261 | 0.00222003275351312 |
| K10578 | neg\_3351 | -0.819346487522125 | 0.00372367376291516 |
| K02491 | neg\_3351 | -0.852824926376343 | 0.00171189540742711 |
| K03724 | neg\_3351 | 0.842424213886261 | 0.00222003275351312 |
| K14571 | neg\_3355 | -0.814593434333801 | 0.00410613103846336 |
| K01483 | neg\_3355 | -0.806060612201691 | 0.0048620605246823 |
| K02521 | neg\_3355 | -0.830303013324738 | 0.00294022813612127 |
| K18537 | neg\_3355 | -0.818181812763214 | 0.00381492051076338 |
| K10578 | neg\_3355 | -0.839830160140991 | 0.00236205253090405 |
| K19784 | neg\_3355 | -0.816012322902679 | 0.0039891464803441 |
| K02919 | neg\_3355 | -0.854545474052429 | 0.00163680247839526 |
| K18198 | neg\_3360 | -0.850415349006653 | 0.00182121334938934 |
| K17329 | neg\_3360 | -0.900500416755676 | 0.000379701199800131 |
| K10237 | neg\_3360 | -0.867148578166962 | 0.00115736689574253 |
| K02461 | neg\_3360 | -0.922580659389496 | 0.000143036279588005 |
| K03651 | neg\_3360 | 0.90043979883194 | 0.000380598613888061 |
| K12072 | neg\_3360 | -0.83225804567337 | 0.00281408229788882 |
| K02665 | neg\_3360 | -0.845363914966583 | 0.00206666064770777 |
| K05830 | neg\_3360 | -0.825403153896332 | 0.00327420560319291 |
| K21217 | neg\_3360 | -0.845363914966583 | 0.00206666064770777 |
| K19157 | neg\_3360 | 0.875427544116974 | 0.000904110124716251 |
| K04748 | neg\_3360 | -0.869174480438232 | 0.00109115185763287 |
| K16014 | neg\_3360 | 0.83225804567337 | 0.00281408229788882 |
| K06145 | neg\_3360 | 0.894186735153198 | 0.000481835628326488 |
| K03414 | neg\_3360 | -0.845363914966583 | 0.00206666064770777 |
| K05802 | neg\_3360 | -0.860478162765503 | 0.00139610202996154 |
| K10942 | neg\_3360 | -0.845363914966583 | 0.00206666064770777 |
| K06311 | neg\_3360 | -0.859276235103607 | 0.00144264047431664 |
| K11933 | neg\_3377 | -0.852824926376343 | 0.00171189540742711 |
| K00817 | neg\_3377 | 0.818181812763214 | 0.00381492051076338 |
| K01524 | neg\_3377 | 0.842424213886261 | 0.00222003275351312 |
| K03414 | neg\_3377 | -0.819346487522125 | 0.00372367376291516 |
| K05802 | neg\_3377 | -0.885716199874878 | 0.000648751005291714 |
| K05851 | neg\_3377 | -0.83790922164917 | 0.00247136195299369 |
| K10942 | neg\_3377 | -0.819346487522125 | 0.00372367376291516 |
| K14051 | neg\_3377 | 0.839830160140991 | 0.00236205253090405 |
| K08714 | neg\_3414 | -0.963636338710785 | 7.32099466027591e-06 |
| K11933 | neg\_3414 | 0.865095794200897 | 0.00122739260821847 |
| K04075 | neg\_3414 | -0.854545474052429 | 0.00163680247839526 |
| K03470 | neg\_3414 | -0.818181812763214 | 0.00381492051076338 |
| K07391 | neg\_3414 | -0.830303013324738 | 0.00294022813612127 |
| K10237 | neg\_3414 | 0.846925735473633 | 0.0019883679465349 |
| K03524 | neg\_3414 | -0.854545474052429 | 0.00163680247839526 |
| K01714 | neg\_3414 | -0.878787875175476 | 0.000813862205061078 |
| K04096 | neg\_3414 | -0.806060612201691 | 0.0048620605246823 |
| K04069 | neg\_3414 | -0.806060612201691 | 0.0048620605246823 |
| K07166 | neg\_3414 | -0.878787875175476 | 0.000813862205061078 |
| K06167 | neg\_3414 | -0.806060612201691 | 0.0048620605246823 |
| K04565 | neg\_3414 | -0.818181812763214 | 0.00381492051076338 |
| K13798 | neg\_3414 | 0.854545474052429 | 0.00163680247839526 |
| K07031 | neg\_3414 | 0.854545474052429 | 0.00163680247839526 |
| K07139 | neg\_3414 | -0.878787875175476 | 0.000813862205061078 |
| K05787 | neg\_3414 | -0.866666674613953 | 0.0011735379121256 |
| K03607 | neg\_3414 | 0.846658051013947 | 0.00200163182665913 |
| K12231 | neg\_3414 | 0.873969614505768 | 0.000945449010965937 |
| K00765 | neg\_3414 | -0.806060612201691 | 0.0048620605246823 |
| K06179 | neg\_3414 | -0.866666674613953 | 0.0011735379121256 |
| K00783 | neg\_3414 | -0.842424213886261 | 0.00222003275351312 |
| K01483 | neg\_3414 | 0.842424213886261 | 0.00222003275351312 |
| K00014 | neg\_3414 | -0.806060612201691 | 0.0048620605246823 |
| K10578 | neg\_3414 | 0.833002269268036 | 0.00276710271221536 |
| K14051 | neg\_3414 | -0.873969614505768 | 0.000945449010965937 |
| K03724 | neg\_3414 | -0.830303013324738 | 0.00294022813612127 |
| K07574 | neg\_3414 | -0.854545474052429 | 0.00163680247839526 |
| K00057 | neg\_3414 | -0.842424213886261 | 0.00222003275351312 |
| K02461 | neg\_3422 | 0.83790922164917 | 0.00247136195299369 |
| K02665 | neg\_3422 | 0.846658051013947 | 0.00200163182665913 |
| K21217 | neg\_3422 | 0.873969614505768 | 0.000945449010965937 |
| K19157 | neg\_3422 | -0.927272737026215 | 0.000112034447641074 |
| K16014 | neg\_3422 | -0.931705057621002 | 8.75967112814457e-05 |
| K03414 | neg\_3422 | 0.833002269268036 | 0.00276710271221536 |
| K05802 | neg\_3422 | 0.859855890274048 | 0.00142005806532719 |
| K10942 | neg\_3422 | 0.833002269268036 | 0.00276710271221536 |
| K06311 | neg\_3422 | 0.863225877285004 | 0.00129381457010247 |
| K18198 | neg\_344 | -0.842424213886261 | 0.00222003275351312 |
| K17329 | neg\_344 | -0.872786045074463 | 0.000980007503875058 |
| K03470 | neg\_344 | 0.842424213886261 | 0.00222003275351312 |
| K10237 | neg\_344 | -0.885716199874878 | 0.000648751005291714 |
| K02461 | neg\_344 | -0.956717252731323 | 1.45713471533249e-05 |
| K07126 | neg\_344 | 0.806060612201691 | 0.0048620605246823 |
| K06285 | neg\_344 | -0.806060612201691 | 0.0048620605246823 |
| K09815 | neg\_344 | 0.806060612201691 | 0.0048620605246823 |
| K12543 | neg\_344 | -0.878787875175476 | 0.000813862205061078 |
| K02665 | neg\_344 | -0.846658051013947 | 0.00200163182665913 |
| K21217 | neg\_344 | -0.873969614505768 | 0.000945449010965937 |
| K19157 | neg\_344 | 0.818181812763214 | 0.00381492051076338 |
| K00849 | neg\_344 | 0.842424213886261 | 0.00222003275351312 |
| K06145 | neg\_344 | 0.806060612201691 | 0.0048620605246823 |
| K03414 | neg\_344 | -0.819346487522125 | 0.00372367376291516 |
| K10942 | neg\_344 | -0.819346487522125 | 0.00372367376291516 |
| K06311 | neg\_344 | -0.826751530170441 | 0.00317971472634282 |
| K00057 | neg\_344 | 0.806060612201691 | 0.0048620605246823 |
| K20452 | neg\_3440 | -0.950991809368134 | 2.37837330223645e-05 |
| K18298 | neg\_3440 | -0.818181812763214 | 0.00381492051076338 |
| K02491 | neg\_3440 | -0.80374151468277 | 0.00508357521208702 |
| K00845 | neg\_3458 | 0.830303013324738 | 0.00294022813612127 |
| K03769 | neg\_3458 | 0.806060612201691 | 0.0048620605246823 |
| K12072 | neg\_3458 | -0.90043979883194 | 0.000380598613888061 |
| K09815 | neg\_3458 | 0.890909075737 | 0.000542144516154419 |
| K07121 | neg\_3458 | -0.842424213886261 | 0.00222003275351312 |
| K21217 | neg\_3458 | -0.819346487522125 | 0.00372367376291516 |
| K16014 | neg\_3458 | 0.869174480438232 | 0.00109115185763287 |
| K03414 | neg\_3458 | -0.833002269268036 | 0.00276710271221536 |
| K10942 | neg\_3458 | -0.833002269268036 | 0.00276710271221536 |
| K02461 | neg\_3487 | 0.844162285327911 | 0.00212839548320387 |
| K02665 | neg\_3487 | 0.826174378395081 | 0.00321991677364641 |
| K17329 | neg\_3496 | -0.859855890274048 | 0.00142005806532719 |
| K02461 | neg\_3496 | -0.856668412685394 | 0.00154747182074688 |
| K03651 | neg\_3496 | 0.842424213886261 | 0.00222003275351312 |
| K02665 | neg\_3496 | -0.833002269268036 | 0.00276710271221536 |
| K19157 | neg\_3496 | 0.878787875175476 | 0.000813862205061078 |
| K04748 | neg\_3496 | -0.818181812763214 | 0.00381492051076338 |
| K06145 | neg\_3496 | 0.806060612201691 | 0.0048620605246823 |
| K05802 | neg\_3496 | -0.898646354675293 | 0.000407860267078952 |
| K06311 | neg\_3496 | -0.826751530170441 | 0.00317971472634282 |
| K02919 | neg\_3496 | -0.806060612201691 | 0.0048620605246823 |
| K18198 | neg\_3498 | 0.830303013324738 | 0.00294022813612127 |
| K07672 | neg\_3498 | -0.850415349006653 | 0.00182121334938934 |
| K17329 | neg\_3498 | 0.821065366268158 | 0.00359188206024896 |
| K01261 | neg\_3498 | -0.903030276298523 | 0.000343612565232743 |
| K00100 | neg\_3498 | 0.878787875175476 | 0.000813862205061078 |
| K04061 | neg\_3498 | 0.830303013324738 | 0.00294022813612127 |
| K03406 | neg\_3498 | 0.818181812763214 | 0.00381492051076338 |
| K01729 | neg\_3498 | -0.806060612201691 | 0.0048620605246823 |
| K03651 | neg\_3498 | -0.939393937587738 | 5.48405364009241e-05 |
| K05830 | neg\_3498 | 0.818181812763214 | 0.00381492051076338 |
| K07454 | neg\_3498 | -0.806060612201691 | 0.0048620605246823 |
| K04748 | neg\_3498 | 0.927272737026215 | 0.000112034447641074 |
| K02744 | neg\_3498 | 0.830303013324738 | 0.00294022813612127 |
| K01909 | neg\_3498 | 0.903030276298523 | 0.000343612565232743 |
| K06145 | neg\_3498 | -0.903030276298523 | 0.000343612565232743 |
| K05964 | neg\_3498 | 0.854545474052429 | 0.00163680247839526 |
| K11933 | neg\_3504 | -0.826086938381195 | 0.00322603919015818 |
| K17329 | neg\_3504 | -0.831202387809753 | 0.00288170263251697 |
| K02461 | neg\_3504 | -0.879905462265015 | 0.000785360184749173 |
| K03651 | neg\_3504 | 0.840554058551788 | 0.00232177976116144 |
| K02665 | neg\_3504 | -0.8156378865242 | 0.00401978302331507 |
| K21217 | neg\_3504 | -0.8156378865242 | 0.00401978302331507 |
| K06926 | neg\_3504 | 0.80374151468277 | 0.00508357521208702 |
| K19157 | neg\_3504 | 0.901908397674561 | 0.000359290276283897 |
| K06145 | neg\_3504 | 0.80374151468277 | 0.00508357521208702 |
| K03414 | neg\_3504 | -0.843286633491516 | 0.00217421422639408 |
| K05802 | neg\_3504 | -0.922830998897552 | 0.000141238827938484 |
| K10942 | neg\_3504 | -0.843286633491516 | 0.00217421422639408 |
| K06311 | neg\_3504 | -0.861575186252594 | 0.00135458546448364 |
| K02919 | neg\_3504 | -0.865095794200897 | 0.00122739260821847 |
| K18198 | neg\_3529 | 0.830303013324738 | 0.00294022813612127 |
| K02461 | neg\_3529 | 0.812897026538849 | 0.00424919679863534 |
| K02665 | neg\_3529 | 0.873969614505768 | 0.000945449010965937 |
| K21217 | neg\_3529 | 0.846658051013947 | 0.00200163182665913 |
| K16014 | neg\_3529 | -0.806643962860107 | 0.00480744414521128 |
| K05802 | neg\_3529 | 0.808135211467743 | 0.00466981917809761 |
| K06311 | neg\_3529 | 0.851067781448364 | 0.00179112909994994 |
| K20525 | neg\_3529 | -0.818181812763214 | 0.00381492051076338 |
| K02784 | neg\_3564 | -0.806060612201691 | 0.0048620605246823 |
| K03501 | neg\_3564 | -0.818181812763214 | 0.00381492051076338 |
| K01809 | neg\_3564 | -0.878787875175476 | 0.000813862205061078 |
| K03218 | neg\_3564 | -0.842424213886261 | 0.00222003275351312 |
| K07493 | neg\_3566 | 0.862921476364136 | 0.00130486922968087 |
| K20452 | neg\_3586 | -0.877366662025452 | 0.000851188393096614 |
| K20452 | neg\_3587 | 0.80374151468277 | 0.00508357521208702 |
| K11933 | neg\_3592 | -0.816012322902679 | 0.0039891464803441 |
| K10237 | neg\_3592 | -0.833995580673218 | 0.00270528265085312 |
| K02461 | neg\_3592 | -0.881680607795715 | 0.000741594922435862 |
| K03651 | neg\_3592 | 0.806060612201691 | 0.0048620605246823 |
| K21217 | neg\_3592 | -0.805690705776215 | 0.00489692183645341 |
| K19157 | neg\_3592 | 0.842424213886261 | 0.00222003275351312 |
| K18537 | neg\_3592 | -0.818181812763214 | 0.00381492051076338 |
| K16014 | neg\_3592 | 0.844162285327911 | 0.00212839548320387 |
| K03414 | neg\_3592 | -0.873969614505768 | 0.000945449010965937 |
| K05802 | neg\_3592 | -0.924506723880768 | 0.000129632545059266 |
| K10942 | neg\_3592 | -0.873969614505768 | 0.000945449010965937 |
| K06311 | neg\_3592 | -0.930095493793488 | 9.59607762942571e-05 |
| K02919 | neg\_3592 | -0.842424213886261 | 0.00222003275351312 |
| K17329 | neg\_3608 | -0.831202387809753 | 0.00288170263251697 |
| K02461 | neg\_3608 | -0.879905462265015 | 0.000785360184749173 |
| K03651 | neg\_3608 | 0.877366662025452 | 0.000851188393096614 |
| K02665 | neg\_3608 | -0.8156378865242 | 0.00401978302331507 |
| K05830 | neg\_3608 | -0.828283190727234 | 0.00307477558489877 |
| K21217 | neg\_3608 | -0.8156378865242 | 0.00401978302331507 |
| K19157 | neg\_3608 | 0.914179265499115 | 0.000213749081869885 |
| K04748 | neg\_3608 | -0.828283190727234 | 0.00307477558489877 |
| K16014 | neg\_3608 | 0.82926344871521 | 0.00300893631849064 |
| K06145 | neg\_3608 | 0.840554058551788 | 0.00232177976116144 |
| K03414 | neg\_3608 | -0.843286633491516 | 0.00217421422639408 |
| K05802 | neg\_3608 | -0.922830998897552 | 0.000141238827938484 |
| K10942 | neg\_3608 | -0.843286633491516 | 0.00217421422639408 |
| K06311 | neg\_3608 | -0.861575186252594 | 0.00135458546448364 |
| K02919 | neg\_3608 | -0.852824926376343 | 0.00171189540742711 |
| K18198 | neg\_3609 | -0.842424213886261 | 0.00222003275351312 |
| K11933 | neg\_3609 | -0.901908397674561 | 0.000359290276283897 |
| K17329 | neg\_3609 | -0.833995580673218 | 0.00270528265085312 |
| K10237 | neg\_3609 | -0.846925735473633 | 0.0019883679465349 |
| K04061 | neg\_3609 | -0.830303013324738 | 0.00294022813612127 |
| K04096 | neg\_3609 | 0.854545474052429 | 0.00163680247839526 |
| K02461 | neg\_3609 | -0.844162285327911 | 0.00212839548320387 |
| K07126 | neg\_3609 | 0.806060612201691 | 0.0048620605246823 |
| K03651 | neg\_3609 | 0.830303013324738 | 0.00294022813612127 |
| K04565 | neg\_3609 | 0.830303013324738 | 0.00294022813612127 |
| K06926 | neg\_3609 | 0.842424213886261 | 0.00222003275351312 |
| K04748 | neg\_3609 | -0.806060612201691 | 0.0048620605246823 |
| K18537 | neg\_3609 | -0.806060612201691 | 0.0048620605246823 |
| K06145 | neg\_3609 | 0.806060612201691 | 0.0048620605246823 |
| K03414 | neg\_3609 | -0.860313832759857 | 0.00140239962667921 |
| K05802 | neg\_3609 | -0.898646354675293 | 0.000407860267078952 |
| K05851 | neg\_3609 | -0.806643962860107 | 0.00480744414521128 |
| K10942 | neg\_3609 | -0.860313832759857 | 0.00140239962667921 |
| K06311 | neg\_3609 | -0.881463050842285 | 0.000746860533753502 |
| K02919 | neg\_3609 | -0.866666674613953 | 0.0011735379121256 |
| K11933 | neg\_3637 | -0.80374151468277 | 0.00508357521208702 |
| K17329 | neg\_3637 | -0.808135211467743 | 0.00466981917809761 |
| K02461 | neg\_3637 | -0.894186735153198 | 0.000481835628326488 |
| K03651 | neg\_3637 | 0.842424213886261 | 0.00222003275351312 |
| K02665 | neg\_3637 | -0.819346487522125 | 0.00372367376291516 |
| K05830 | neg\_3637 | -0.818181812763214 | 0.00381492051076338 |
| K21217 | neg\_3637 | -0.846658051013947 | 0.00200163182665913 |
| K06926 | neg\_3637 | 0.830303013324738 | 0.00294022813612127 |
| K19157 | neg\_3637 | 0.939393937587738 | 5.48405364009241e-05 |
| K06145 | neg\_3637 | 0.806060612201691 | 0.0048620605246823 |
| K03414 | neg\_3637 | -0.846658051013947 | 0.00200163182665913 |
| K05802 | neg\_3637 | -0.898646354675293 | 0.000407860267078952 |
| K10942 | neg\_3637 | -0.846658051013947 | 0.00200163182665913 |
| K06311 | neg\_3637 | -0.83890962600708 | 0.00241398974898654 |
| K02919 | neg\_3637 | -0.866666674613953 | 0.0011735379121256 |
| K11933 | neg\_3653 | -0.865095794200897 | 0.00122739260821847 |
| K02461 | neg\_3653 | -0.856668412685394 | 0.00154747182074688 |
| K03651 | neg\_3653 | 0.806060612201691 | 0.0048620605246823 |
| K19157 | neg\_3653 | 0.854545474052429 | 0.00163680247839526 |
| K03414 | neg\_3653 | -0.860313832759857 | 0.00140239962667921 |
| K05802 | neg\_3653 | -0.937436878681183 | 6.21251750798635e-05 |
| K10942 | neg\_3653 | -0.860313832759857 | 0.00140239962667921 |
| K06311 | neg\_3653 | -0.887542068958282 | 0.000609666476501403 |
| K02919 | neg\_3653 | -0.890909075737 | 0.000542144516154419 |
| K00640 | neg\_3656 | -0.818181812763214 | 0.00381492051076338 |
| K03827 | neg\_3656 | 0.854545474052429 | 0.00163680247839526 |
| K03225 | neg\_3656 | 0.806060612201691 | 0.0048620605246823 |
| K07031 | neg\_3656 | 0.866666674613953 | 0.0011735379121256 |
| K07039 | neg\_3656 | -0.806060612201691 | 0.0048620605246823 |
| K21029 | neg\_3656 | 0.806060612201691 | 0.0048620605246823 |
| K07396 | neg\_3656 | 0.806060612201691 | 0.0048620605246823 |
| K06907 | neg\_3656 | 0.842424213886261 | 0.00222003275351312 |
| K20885 | neg\_3656 | -0.818181812763214 | 0.00381492051076338 |
| K04085 | neg\_3656 | 0.854545474052429 | 0.00163680247839526 |
| K09512 | neg\_3656 | -0.885716199874878 | 0.000648751005291714 |
| K07707 | neg\_3656 | 0.854545474052429 | 0.00163680247839526 |
| K11933 | neg\_3666 | -0.852824926376343 | 0.00171189540742711 |
| K07099 | neg\_3666 | 0.842424213886261 | 0.00222003275351312 |
| K07391 | neg\_3666 | 0.818181812763214 | 0.00381492051076338 |
| K04096 | neg\_3666 | 0.878787875175476 | 0.000813862205061078 |
| K04069 | neg\_3666 | 0.903030276298523 | 0.000343612565232743 |
| K00817 | neg\_3666 | 0.939393937587738 | 5.48405364009241e-05 |
| K00852 | neg\_3666 | 0.878787875175476 | 0.000813862205061078 |
| K19411 | neg\_3666 | 0.890909075737 | 0.000542144516154419 |
| K00800 | neg\_3666 | 0.806060612201691 | 0.0048620605246823 |
| K06285 | neg\_3666 | -0.842424213886261 | 0.00222003275351312 |
| K04565 | neg\_3666 | 0.830303013324738 | 0.00294022813612127 |
| K19334 | neg\_3666 | 0.818181812763214 | 0.00381492051076338 |
| K03573 | neg\_3666 | 0.866666674613953 | 0.0011735379121256 |
| K03643 | neg\_3666 | 0.842424213886261 | 0.00222003275351312 |
| K01295 | neg\_3666 | 0.818181812763214 | 0.00381492051076338 |
| K00849 | neg\_3666 | 0.830303013324738 | 0.00294022813612127 |
| K13014 | neg\_3666 | 0.806060612201691 | 0.0048620605246823 |
| K04517 | neg\_3666 | 0.830303013324738 | 0.00294022813612127 |
| K01524 | neg\_3666 | 0.806060612201691 | 0.0048620605246823 |
| K07720 | neg\_3666 | 0.842424213886261 | 0.00222003275351312 |
| K03414 | neg\_3666 | -0.860313832759857 | 0.00140239962667921 |
| K07043 | neg\_3666 | 0.842424213886261 | 0.00222003275351312 |
| K05851 | neg\_3666 | -0.906692802906036 | 0.000295911879524491 |
| K10942 | neg\_3666 | -0.860313832759857 | 0.00140239962667921 |
| K03724 | neg\_3666 | 0.806060612201691 | 0.0048620605246823 |
| K16898 | neg\_3666 | 0.806060612201691 | 0.0048620605246823 |
| K11933 | neg\_3689 | -0.914179265499115 | 0.000213749081869885 |
| K17329 | neg\_3689 | -0.846925735473633 | 0.0019883679465349 |
| K10237 | neg\_3689 | -0.872786045074463 | 0.000980007503875058 |
| K04096 | neg\_3689 | 0.854545474052429 | 0.00163680247839526 |
| K02461 | neg\_3689 | -0.881680607795715 | 0.000741594922435862 |
| K06285 | neg\_3689 | -0.830303013324738 | 0.00294022813612127 |
| K02458 | neg\_3689 | -0.852824926376343 | 0.00171189540742711 |
| K04565 | neg\_3689 | 0.854545474052429 | 0.00163680247839526 |
| K18537 | neg\_3689 | -0.890909075737 | 0.000542144516154419 |
| K03414 | neg\_3689 | -0.846658051013947 | 0.00200163182665913 |
| K05802 | neg\_3689 | -0.859855890274048 | 0.00142005806532719 |
| K05851 | neg\_3689 | -0.81915009021759 | 0.00373894966455346 |
| K10942 | neg\_3689 | -0.846658051013947 | 0.00200163182665913 |
| K09512 | neg\_3689 | 0.808135211467743 | 0.00466981917809761 |
| K06311 | neg\_3689 | -0.881463050842285 | 0.000746860533753502 |
| K02461 | neg\_3705 | 0.83790922164917 | 0.00247136195299369 |
| K02665 | neg\_3705 | 0.846658051013947 | 0.00200163182665913 |
| K21217 | neg\_3705 | 0.873969614505768 | 0.000945449010965937 |
| K19157 | neg\_3705 | -0.890909075737 | 0.000542144516154419 |
| K06871 | neg\_3705 | -0.842424213886261 | 0.00222003275351312 |
| K16014 | neg\_3705 | -0.869174480438232 | 0.00109115185763287 |
| K03414 | neg\_3705 | 0.833002269268036 | 0.00276710271221536 |
| K05802 | neg\_3705 | 0.859855890274048 | 0.00142005806532719 |
| K10942 | neg\_3705 | 0.833002269268036 | 0.00276710271221536 |
| K19157 | neg\_3723 | -0.818181812763214 | 0.00381492051076338 |
| K18298 | neg\_3723 | 0.830303013324738 | 0.00294022813612127 |
| K00845 | neg\_3739 | 0.939393937587738 | 5.48405364009241e-05 |
| K00640 | neg\_3739 | 0.878787875175476 | 0.000813862205061078 |
| K07672 | neg\_3739 | 0.850415349006653 | 0.00182121334938934 |
| K07099 | neg\_3739 | 0.854545474052429 | 0.00163680247839526 |
| K03827 | neg\_3739 | -0.975757598876953 | 1.46754063035104e-06 |
| K10237 | neg\_3739 | -0.846925735473633 | 0.0019883679465349 |
| K00100 | neg\_3739 | -0.830303013324738 | 0.00294022813612127 |
| K01714 | neg\_3739 | 0.890909075737 | 0.000542144516154419 |
| K04069 | neg\_3739 | 0.818181812763214 | 0.00381492051076338 |
| K00852 | neg\_3739 | 0.830303013324738 | 0.00294022813612127 |
| K10907 | neg\_3739 | 0.806060612201691 | 0.0048620605246823 |
| K12072 | neg\_3739 | -0.81915009021759 | 0.00373894966455346 |
| K09815 | neg\_3739 | 0.854545474052429 | 0.00163680247839526 |
| K07121 | neg\_3739 | -0.866666674613953 | 0.0011735379121256 |
| K07039 | neg\_3739 | 0.878787875175476 | 0.000813862205061078 |
| K00949 | neg\_3739 | 0.806060612201691 | 0.0048620605246823 |
| K13665 | neg\_3739 | -0.818181812763214 | 0.00381492051076338 |
| K07396 | neg\_3739 | -0.963636338710785 | 7.32099466027591e-06 |
| K12231 | neg\_3739 | -0.833002269268036 | 0.00276710271221536 |
| K04517 | neg\_3739 | 0.830303013324738 | 0.00294022813612127 |
| K13611 | neg\_3739 | -0.859855890274048 | 0.00142005806532719 |
| K10953 | neg\_3739 | 0.885716199874878 | 0.000648751005291714 |
| K10578 | neg\_3739 | -0.819346487522125 | 0.00372367376291516 |
| K07707 | neg\_3739 | -0.806060612201691 | 0.0048620605246823 |
| K03724 | neg\_3739 | 0.830303013324738 | 0.00294022813612127 |
| K19784 | neg\_3739 | -0.840554058551788 | 0.00232177976116144 |
| K01661 | neg\_3739 | -0.854545474052429 | 0.00163680247839526 |
| K02493 | neg\_3759 | 0.852824926376343 | 0.00171189540742711 |
| K02784 | neg\_3759 | 0.877366662025452 | 0.000851188393096614 |
| K03769 | neg\_3759 | 0.926450133323669 | 0.000117071096429999 |
| K17331 | neg\_3759 | 0.898584127426147 | 0.000408831129309783 |
| K19334 | neg\_3759 | 0.816012322902679 | 0.0039891464803441 |
| K13665 | neg\_3759 | -0.828283190727234 | 0.00307477558489877 |
| K05836 | neg\_3759 | 0.816012322902679 | 0.0039891464803441 |
| K01409 | neg\_3759 | 0.840554058551788 | 0.00232177976116144 |
| K20525 | neg\_3759 | 0.852824926376343 | 0.00171189540742711 |
| K01159 | neg\_3762 | 0.806060612201691 | 0.0048620605246823 |
| K01729 | neg\_3762 | 0.806060612201691 | 0.0048620605246823 |
| K07139 | neg\_3762 | 0.806060612201691 | 0.0048620605246823 |
| K05787 | neg\_3762 | 0.878787875175476 | 0.000813862205061078 |
| K20452 | neg\_3762 | -0.926450133323669 | 0.000117071096429999 |
| K11068 | neg\_3762 | 0.806060612201691 | 0.0048620605246823 |
| K14575 | neg\_3762 | 0.83890962600708 | 0.00241398974898654 |
| K18298 | neg\_3762 | -0.915151536464691 | 0.000204472206099204 |
| K09951 | neg\_3762 | 0.806060612201691 | 0.0048620605246823 |
| K08714 | neg\_3785 | 0.927272737026215 | 0.000112034447641074 |
| K12543 | neg\_3785 | -0.818181812763214 | 0.00381492051076338 |
| K07031 | neg\_3785 | -0.806060612201691 | 0.0048620605246823 |
| K07139 | neg\_3785 | 0.890909075737 | 0.000542144516154419 |
| K05787 | neg\_3785 | 0.890909075737 | 0.000542144516154419 |
| K00588 | neg\_3785 | -0.852824926376343 | 0.00171189540742711 |
| K06926 | neg\_3785 | 0.830303013324738 | 0.00294022813612127 |
| K11933 | neg\_3807 | -0.877366662025452 | 0.000851188393096614 |
| K10237 | neg\_3807 | -0.808135211467743 | 0.00466981917809761 |
| K02458 | neg\_3807 | -0.889637529850006 | 0.000566964581027118 |
| K07031 | neg\_3807 | -0.842424213886261 | 0.00222003275351312 |
| K12231 | neg\_3807 | -0.833002269268036 | 0.00276710271221536 |
| K02521 | neg\_3807 | -0.842424213886261 | 0.00222003275351312 |
| K18537 | neg\_3807 | -0.987878799438477 | 9.30742460880651e-08 |
| K10578 | neg\_3807 | -0.819346487522125 | 0.00372367376291516 |
| K09512 | neg\_3807 | 0.821065366268158 | 0.00359188206024896 |
| K02491 | neg\_3807 | -0.816012322902679 | 0.0039891464803441 |
| K07707 | neg\_3807 | -0.818181812763214 | 0.00381492051076338 |
| K02919 | neg\_3807 | -0.806060612201691 | 0.0048620605246823 |
| K18198 | neg\_3820 | -0.806060612201691 | 0.0048620605246823 |
| K11933 | neg\_3820 | -0.828283190727234 | 0.00307477558489877 |
| K17329 | neg\_3820 | -0.872786045074463 | 0.000980007503875058 |
| K02461 | neg\_3820 | -0.831656157970428 | 0.00285249465359505 |
| K02745 | neg\_3820 | -0.818181812763214 | 0.00381492051076338 |
| K03651 | neg\_3820 | 0.903030276298523 | 0.000343612565232743 |
| K05830 | neg\_3820 | -0.806060612201691 | 0.0048620605246823 |
| K19157 | neg\_3820 | 0.818181812763214 | 0.00381492051076338 |
| K04748 | neg\_3820 | -0.903030276298523 | 0.000343612565232743 |
| K18692 | neg\_3820 | -0.818181812763214 | 0.00381492051076338 |
| K06145 | neg\_3820 | 0.866666674613953 | 0.0011735379121256 |
| K03414 | neg\_3820 | -0.819346487522125 | 0.00372367376291516 |
| K05802 | neg\_3820 | -0.885716199874878 | 0.000648751005291714 |
| K10942 | neg\_3820 | -0.819346487522125 | 0.00372367376291516 |
| K06311 | neg\_3820 | -0.826751530170441 | 0.00317971472634282 |
| K03724 | neg\_3820 | 0.806060612201691 | 0.0048620605246823 |
| K02919 | neg\_3820 | -0.818181812763214 | 0.00381492051076338 |
| K08714 | neg\_3852 | 0.854545474052429 | 0.00163680247839526 |
| K00640 | neg\_3852 | 0.806060612201691 | 0.0048620605246823 |
| K05946 | neg\_3852 | 0.939393937587738 | 5.48405364009241e-05 |
| K11933 | neg\_3852 | -0.877366662025452 | 0.000851188393096614 |
| K07099 | neg\_3852 | 0.903030276298523 | 0.000343612565232743 |
| K04075 | neg\_3852 | 0.903030276298523 | 0.000343612565232743 |
| K01159 | neg\_3852 | 0.806060612201691 | 0.0048620605246823 |
| K03470 | neg\_3852 | 0.890909075737 | 0.000542144516154419 |
| K07391 | neg\_3852 | 0.951515138149261 | 2.27985739738035e-05 |
| K10237 | neg\_3852 | -0.885716199874878 | 0.000648751005291714 |
| K04061 | neg\_3852 | -0.890909075737 | 0.000542144516154419 |
| K03524 | neg\_3852 | 0.939393937587738 | 5.48405364009241e-05 |
| K04096 | neg\_3852 | 0.951515138149261 | 2.27985739738035e-05 |
| K04069 | neg\_3852 | 0.890909075737 | 0.000542144516154419 |
| K07126 | neg\_3852 | 0.818181812763214 | 0.00381492051076338 |
| K09779 | neg\_3852 | 0.927272737026215 | 0.000112034447641074 |
| K07166 | neg\_3852 | 0.915151536464691 | 0.000204472206099204 |
| K00852 | neg\_3852 | 0.830303013324738 | 0.00294022813612127 |
| K19411 | neg\_3852 | 0.818181812763214 | 0.00381492051076338 |
| K06167 | neg\_3852 | 0.842424213886261 | 0.00222003275351312 |
| K00800 | neg\_3852 | 0.854545474052429 | 0.00163680247839526 |
| K09761 | neg\_3852 | 0.890909075737 | 0.000542144516154419 |
| K04565 | neg\_3852 | 0.939393937587738 | 5.48405364009241e-05 |
| K13798 | neg\_3852 | -0.830303013324738 | 0.00294022813612127 |
| K07139 | neg\_3852 | 0.866666674613953 | 0.0011735379121256 |
| K15599 | neg\_3852 | -0.890909075737 | 0.000542144516154419 |
| K05787 | neg\_3852 | 0.890909075737 | 0.000542144516154419 |
| K03573 | neg\_3852 | 0.830303013324738 | 0.00294022813612127 |
| K03607 | neg\_3852 | -0.805690705776215 | 0.00489692183645341 |
| K05985 | neg\_3852 | 0.854545474052429 | 0.00163680247839526 |
| K06926 | neg\_3852 | 0.890909075737 | 0.000542144516154419 |
| K00783 | neg\_3852 | 0.878787875175476 | 0.000813862205061078 |
| K09777 | neg\_3852 | 0.878787875175476 | 0.000813862205061078 |
| K00859 | neg\_3852 | 0.915151536464691 | 0.000204472206099204 |
| K01295 | neg\_3852 | 0.866666674613953 | 0.0011735379121256 |
| K03101 | neg\_3852 | 0.830303013324738 | 0.00294022813612127 |
| K01483 | neg\_3852 | -0.842424213886261 | 0.00222003275351312 |
| K00849 | neg\_3852 | 0.903030276298523 | 0.000343612565232743 |
| K04517 | neg\_3852 | 0.878787875175476 | 0.000813862205061078 |
| K11068 | neg\_3852 | 0.806060612201691 | 0.0048620605246823 |
| K00969 | neg\_3852 | 0.866666674613953 | 0.0011735379121256 |
| K07720 | neg\_3852 | 0.903030276298523 | 0.000343612565232743 |
| K10578 | neg\_3852 | -0.846658051013947 | 0.00200163182665913 |
| K07043 | neg\_3852 | 0.818181812763214 | 0.00381492051076338 |
| K05970 | neg\_3852 | 0.806060612201691 | 0.0048620605246823 |
| K05851 | neg\_3852 | -0.831656157970428 | 0.00285249465359505 |
| K04486 | neg\_3852 | 0.806060612201691 | 0.0048620605246823 |
| K01736 | neg\_3852 | 0.890909075737 | 0.000542144516154419 |
| K07053 | neg\_3852 | 0.890909075737 | 0.000542144516154419 |
| K09775 | neg\_3852 | 0.866666674613953 | 0.0011735379121256 |
| K03724 | neg\_3852 | 0.806060612201691 | 0.0048620605246823 |
| K00883 | neg\_3852 | 0.842424213886261 | 0.00222003275351312 |
| K08714 | neg\_3889 | -0.830303013324738 | 0.00294022813612127 |
| K06167 | neg\_3889 | -0.818181812763214 | 0.00381492051076338 |
| K02852 | neg\_3889 | -0.878787875175476 | 0.000813862205061078 |
| K07139 | neg\_3889 | -0.818181812763214 | 0.00381492051076338 |
| K05787 | neg\_3889 | -0.818181812763214 | 0.00381492051076338 |
| K00588 | neg\_3889 | 0.840554058551788 | 0.00232177976116144 |
| K06926 | neg\_3889 | -0.854545474052429 | 0.00163680247839526 |
| K14051 | neg\_3889 | -0.826174378395081 | 0.00321991677364641 |
| K11933 | neg\_392 | -0.865095794200897 | 0.00122739260821847 |
| K02461 | neg\_392 | -0.856668412685394 | 0.00154747182074688 |
| K03651 | neg\_392 | 0.806060612201691 | 0.0048620605246823 |
| K19157 | neg\_392 | 0.854545474052429 | 0.00163680247839526 |
| K03414 | neg\_392 | -0.860313832759857 | 0.00140239962667921 |
| K05802 | neg\_392 | -0.937436878681183 | 6.21251750798635e-05 |
| K10942 | neg\_392 | -0.860313832759857 | 0.00140239962667921 |
| K06311 | neg\_392 | -0.887542068958282 | 0.000609666476501403 |
| K02919 | neg\_392 | -0.890909075737 | 0.000542144516154419 |
| K11933 | neg\_3940 | -0.914179265499115 | 0.000213749081869885 |
| K17329 | neg\_3940 | -0.808135211467743 | 0.00466981917809761 |
| K10237 | neg\_3940 | -0.911576509475708 | 0.000240107073119944 |
| K04061 | neg\_3940 | -0.806060612201691 | 0.0048620605246823 |
| K04096 | neg\_3940 | 0.854545474052429 | 0.00163680247839526 |
| K02461 | neg\_3940 | -0.894186735153198 | 0.000481835628326488 |
| K03651 | neg\_3940 | 0.818181812763214 | 0.00381492051076338 |
| K02458 | neg\_3940 | -0.816012322902679 | 0.0039891464803441 |
| K04565 | neg\_3940 | 0.866666674613953 | 0.0011735379121256 |
| K21217 | neg\_3940 | -0.846658051013947 | 0.00200163182665913 |
| K06926 | neg\_3940 | 0.818181812763214 | 0.00381492051076338 |
| K19157 | neg\_3940 | 0.854545474052429 | 0.00163680247839526 |
| K18537 | neg\_3940 | -0.842424213886261 | 0.00222003275351312 |
| K03414 | neg\_3940 | -0.887625336647034 | 0.000607926148504134 |
| K05802 | neg\_3940 | -0.872786045074463 | 0.000980007503875058 |
| K05851 | neg\_3940 | -0.856668412685394 | 0.00154747182074688 |
| K10942 | neg\_3940 | -0.887625336647034 | 0.000607926148504134 |
| K06311 | neg\_3940 | -0.851067781448364 | 0.00179112909994994 |
| K03724 | neg\_3940 | 0.830303013324738 | 0.00294022813612127 |
| K02919 | neg\_3940 | -0.878787875175476 | 0.000813862205061078 |
| K02784 | neg\_396 | 0.842424213886261 | 0.00222003275351312 |
| K01060 | neg\_410 | 0.878787875175476 | 0.000813862205061078 |
| K17329 | neg\_410 | -0.821065366268158 | 0.00359188206024896 |
| K03412 | neg\_410 | -0.987878799438477 | 9.30742460880651e-08 |
| K00931 | neg\_410 | 0.842424213886261 | 0.00222003275351312 |
| K08688 | neg\_410 | 0.878787875175476 | 0.000813862205061078 |
| K07454 | neg\_410 | 0.842424213886261 | 0.00222003275351312 |
| K00887 | neg\_410 | -0.951515138149261 | 2.27985739738035e-05 |
| K14051 | neg\_410 | 0.846658051013947 | 0.00200163182665913 |
| K08302 | neg\_410 | 0.963636338710785 | 7.32099466027591e-06 |
| K00575 | neg\_410 | -0.806060612201691 | 0.0048620605246823 |
| K00009 | neg\_410 | 0.915151536464691 | 0.000204472206099204 |
| K08714 | neg\_412 | 0.866666674613953 | 0.0011735379121256 |
| K02461 | neg\_412 | -0.881680607795715 | 0.000741594922435862 |
| K02852 | neg\_412 | 0.830303013324738 | 0.00294022813612127 |
| K05830 | neg\_412 | -0.830303013324738 | 0.00294022813612127 |
| K07139 | neg\_412 | 0.806060612201691 | 0.0048620605246823 |
| K05787 | neg\_412 | 0.818181812763214 | 0.00381492051076338 |
| K00588 | neg\_412 | -0.852824926376343 | 0.00171189540742711 |
| K06926 | neg\_412 | 0.866666674613953 | 0.0011735379121256 |
| K19157 | neg\_412 | 0.854545474052429 | 0.00163680247839526 |
| K06871 | neg\_412 | 0.806060612201691 | 0.0048620605246823 |
| K00057 | neg\_412 | 0.842424213886261 | 0.00222003275351312 |
| K14051 | neg\_416 | 0.873969614505768 | 0.000945449010965937 |
| K11933 | neg\_420 | -0.80374151468277 | 0.00508357521208702 |
| K02461 | neg\_420 | -0.825403153896332 | 0.00327420560319291 |
| K19157 | neg\_420 | 0.842424213886261 | 0.00222003275351312 |
| K03414 | neg\_420 | -0.860313832759857 | 0.00140239962667921 |
| K05802 | neg\_420 | -0.937436878681183 | 6.21251750798635e-05 |
| K10942 | neg\_420 | -0.860313832759857 | 0.00140239962667921 |
| K06311 | neg\_420 | -0.942253589630127 | 4.53587077142714e-05 |
| K02919 | neg\_420 | -0.842424213886261 | 0.00222003275351312 |
| K11933 | neg\_430 | -0.863354027271271 | 0.00128918106835152 |
| K17329 | neg\_430 | -0.831202387809753 | 0.00288170263251697 |
| K02461 | neg\_430 | -0.879905462265015 | 0.000785360184749173 |
| K02665 | neg\_430 | -0.8156378865242 | 0.00401978302331507 |
| K21217 | neg\_430 | -0.8156378865242 | 0.00401978302331507 |
| K06926 | neg\_430 | 0.840554058551788 | 0.00232177976116144 |
| K19157 | neg\_430 | 0.865095794200897 | 0.00122739260821847 |
| K03414 | neg\_430 | -0.843286633491516 | 0.00217421422639408 |
| K05802 | neg\_430 | -0.922830998897552 | 0.000141238827938484 |
| K05851 | neg\_430 | -0.816602885723114 | 0.00394116687238188 |
| K10942 | neg\_430 | -0.843286633491516 | 0.00217421422639408 |
| K06311 | neg\_430 | -0.818496406078339 | 0.00379011736651003 |
| K14051 | neg\_430 | 0.801813542842865 | 0.00527313728075196 |
| K02919 | neg\_430 | -0.852824926376343 | 0.00171189540742711 |
| K02493 | neg\_483 | 0.854545474052429 | 0.00163680247839526 |
| K02784 | neg\_483 | 0.890909075737 | 0.000542144516154419 |
| K17331 | neg\_483 | 0.839830160140991 | 0.00236205253090405 |
| K09761 | neg\_483 | 0.830303013324738 | 0.00294022813612127 |
| K03501 | neg\_483 | 0.915151536464691 | 0.000204472206099204 |
| K01990 | neg\_483 | 0.830303013324738 | 0.00294022813612127 |
| K00969 | neg\_483 | 0.854545474052429 | 0.00163680247839526 |
| K00945 | neg\_483 | 0.806060612201691 | 0.0048620605246823 |
| K03218 | neg\_483 | 0.818181812763214 | 0.00381492051076338 |
| K05970 | neg\_483 | 0.806060612201691 | 0.0048620605246823 |
| K00981 | neg\_483 | 0.806060612201691 | 0.0048620605246823 |
| K01409 | neg\_483 | 0.830303013324738 | 0.00294022813612127 |
| K00919 | neg\_483 | 0.890909075737 | 0.000542144516154419 |
| K20525 | neg\_483 | 0.854545474052429 | 0.00163680247839526 |
| K10237 | neg\_497 | 0.859855890274048 | 0.00142005806532719 |
| K02461 | neg\_497 | 0.800390899181366 | 0.0054162086926719 |
| K14571 | neg\_497 | 0.820672512054443 | 0.00362170350001234 |
| K03607 | neg\_497 | 0.860313832759857 | 0.00140239962667921 |
| K18537 | neg\_497 | 0.878787875175476 | 0.000813862205061078 |
| K07707 | neg\_497 | 0.806060612201691 | 0.0048620605246823 |
| K02919 | neg\_497 | 0.806060612201691 | 0.0048620605246823 |
| K18198 | neg\_552 | -0.854545474052429 | 0.00163680247839526 |
| K07672 | neg\_552 | 0.812897026538849 | 0.00424919679863534 |
| K17329 | neg\_552 | -0.872786045074463 | 0.000980007503875058 |
| K03406 | neg\_552 | -0.806060612201691 | 0.0048620605246823 |
| K02461 | neg\_552 | -0.831656157970428 | 0.00285249465359505 |
| K02745 | neg\_552 | -0.806060612201691 | 0.0048620605246823 |
| K03651 | neg\_552 | 0.915151536464691 | 0.000204472206099204 |
| K00013 | neg\_552 | 0.806060612201691 | 0.0048620605246823 |
| K05830 | neg\_552 | -0.830303013324738 | 0.00294022813612127 |
| K19157 | neg\_552 | 0.806060612201691 | 0.0048620605246823 |
| K04748 | neg\_552 | -0.915151536464691 | 0.000204472206099204 |
| K16014 | neg\_552 | 0.806643962860107 | 0.00480744414521128 |
| K06145 | neg\_552 | 0.890909075737 | 0.000542144516154419 |
| K03414 | neg\_552 | -0.819346487522125 | 0.00372367376291516 |
| K05802 | neg\_552 | -0.885716199874878 | 0.000648751005291714 |
| K10942 | neg\_552 | -0.819346487522125 | 0.00372367376291516 |
| K06311 | neg\_552 | -0.857146799564362 | 0.0015278405327328 |
| K18198 | neg\_560 | 0.890909075737 | 0.000542144516154419 |
| K07672 | neg\_560 | -0.887933671474457 | 0.000601513437458179 |
| K03340 | neg\_560 | -0.927272737026215 | 0.000112034447641074 |
| K01159 | neg\_560 | -0.830303013324738 | 0.00294022813612127 |
| K01261 | neg\_560 | -0.818181812763214 | 0.00381492051076338 |
| K04061 | neg\_560 | 0.818181812763214 | 0.00381492051076338 |
| K07126 | neg\_560 | -0.854545474052429 | 0.00163680247839526 |
| K19411 | neg\_560 | -0.866666674613953 | 0.0011735379121256 |
| K02784 | neg\_560 | -0.830303013324738 | 0.00294022813612127 |
| K17331 | neg\_560 | -0.873969614505768 | 0.000945449010965937 |
| K09761 | neg\_560 | -0.830303013324738 | 0.00294022813612127 |
| K19334 | neg\_560 | -0.818181812763214 | 0.00381492051076338 |
| K01684 | neg\_560 | -0.915151536464691 | 0.000204472206099204 |
| K03573 | neg\_560 | -0.903030276298523 | 0.000343612565232743 |
| K15520 | neg\_560 | -0.866666674613953 | 0.0011735379121256 |
| K05989 | neg\_560 | -0.806060612201691 | 0.0048620605246823 |
| K08314 | neg\_560 | -0.806060612201691 | 0.0048620605246823 |
| K01990 | neg\_560 | -0.830303013324738 | 0.00294022813612127 |
| K00969 | neg\_560 | -0.818181812763214 | 0.00381492051076338 |
| K20885 | neg\_560 | -0.818181812763214 | 0.00381492051076338 |
| K01809 | neg\_560 | -0.866666674613953 | 0.0011735379121256 |
| K00945 | neg\_560 | -0.830303013324738 | 0.00294022813612127 |
| K06145 | neg\_560 | -0.806060612201691 | 0.0048620605246823 |
| K05970 | neg\_560 | -0.939393937587738 | 5.48405364009241e-05 |
| K00230 | neg\_560 | -0.806060612201691 | 0.0048620605246823 |
| K08169 | neg\_560 | 0.806060612201691 | 0.0048620605246823 |
| K01299 | neg\_560 | 0.842424213886261 | 0.00222003275351312 |
| K02445 | neg\_560 | -0.842424213886261 | 0.00222003275351312 |
| K05964 | neg\_560 | 0.842424213886261 | 0.00222003275351312 |
| K02566 | neg\_560 | -0.842424213886261 | 0.00222003275351312 |
| K18198 | neg\_580 | 0.842424213886261 | 0.00222003275351312 |
| K02493 | neg\_580 | -0.806060612201691 | 0.0048620605246823 |
| K20525 | neg\_580 | -0.890909075737 | 0.000542144516154419 |
| K18198 | neg\_59 | 0.842424213886261 | 0.00222003275351312 |
| K17329 | neg\_59 | 0.898646354675293 | 0.000407860267078952 |
| K03827 | neg\_59 | 0.806060612201691 | 0.0048620605246823 |
| K02461 | neg\_59 | 0.844162285327911 | 0.00212839548320387 |
| K03651 | neg\_59 | -0.854545474052429 | 0.00163680247839526 |
| K09789 | neg\_59 | -0.806060612201691 | 0.0048620605246823 |
| K02665 | neg\_59 | 0.805690705776215 | 0.00489692183645341 |
| K04748 | neg\_59 | 0.878787875175476 | 0.000813862205061078 |
| K06145 | neg\_59 | -0.854545474052429 | 0.00163680247839526 |
| K05802 | neg\_59 | 0.859855890274048 | 0.00142005806532719 |
| K09512 | neg\_59 | -0.846925735473633 | 0.0019883679465349 |
| K06311 | neg\_59 | 0.875383973121643 | 0.00090532610126548 |
| K02461 | neg\_637 | 0.844162285327911 | 0.00212839548320387 |
| K02665 | neg\_637 | 0.826174378395081 | 0.00321991677364641 |
| K19157 | neg\_637 | -0.866666674613953 | 0.0011735379121256 |
| K06311 | neg\_637 | 0.826751530170441 | 0.00317971472634282 |
| K00845 | neg\_64 | 0.830303013324738 | 0.00294022813612127 |
| K18198 | neg\_64 | -0.866666674613953 | 0.0011735379121256 |
| K00640 | neg\_64 | 0.806060612201691 | 0.0048620605246823 |
| K07672 | neg\_64 | 0.869174480438232 | 0.00109115185763287 |
| K01060 | neg\_64 | 0.830303013324738 | 0.00294022813612127 |
| K05946 | neg\_64 | 0.866666674613953 | 0.0011735379121256 |
| K07099 | neg\_64 | 0.842424213886261 | 0.00222003275351312 |
| K03827 | neg\_64 | -0.830303013324738 | 0.00294022813612127 |
| K10237 | neg\_64 | -0.898646354675293 | 0.000407860267078952 |
| K03406 | neg\_64 | -0.830303013324738 | 0.00294022813612127 |
| K04096 | neg\_64 | 0.806060612201691 | 0.0048620605246823 |
| K04069 | neg\_64 | 0.818181812763214 | 0.00381492051076338 |
| K02745 | neg\_64 | -0.854545474052429 | 0.00163680247839526 |
| K00852 | neg\_64 | 0.818181812763214 | 0.00381492051076338 |
| K03651 | neg\_64 | 0.818181812763214 | 0.00381492051076338 |
| K04565 | neg\_64 | 0.830303013324738 | 0.00294022813612127 |
| K07396 | neg\_64 | -0.830303013324738 | 0.00294022813612127 |
| K15792 | neg\_64 | 0.842424213886261 | 0.00222003275351312 |
| K16199 | neg\_64 | 0.818181812763214 | 0.00381492051076338 |
| K00344 | neg\_64 | 0.818181812763214 | 0.00381492051076338 |
| K04517 | neg\_64 | 0.806060612201691 | 0.0048620605246823 |
| K04748 | neg\_64 | -0.806060612201691 | 0.0048620605246823 |
| K10578 | neg\_64 | -0.805690705776215 | 0.00489692183645341 |
| K06145 | neg\_64 | 0.854545474052429 | 0.00163680247839526 |
| K03414 | neg\_64 | -0.826174378395081 | 0.00321991677364641 |
| K10942 | neg\_64 | -0.826174378395081 | 0.00321991677364641 |
| K03724 | neg\_64 | 0.830303013324738 | 0.00294022813612127 |
| K11933 | neg\_675 | 0.852824926376343 | 0.00171189540742711 |
| K09779 | neg\_675 | -0.842424213886261 | 0.00222003275351312 |
| K02458 | neg\_675 | 0.828283190727234 | 0.00307477558489877 |
| K07031 | neg\_675 | 0.830303013324738 | 0.00294022813612127 |
| K01295 | neg\_675 | -0.818181812763214 | 0.00381492051076338 |
| K01483 | neg\_675 | 0.854545474052429 | 0.00163680247839526 |
| K18537 | neg\_675 | 0.842424213886261 | 0.00222003275351312 |
| K07720 | neg\_675 | -0.806060612201691 | 0.0048620605246823 |
| K14051 | neg\_675 | -0.826174378395081 | 0.00321991677364641 |
| K07574 | neg\_675 | -0.830303013324738 | 0.00294022813612127 |
| K17329 | neg\_677 | -0.821065366268158 | 0.00359188206024896 |
| K02461 | neg\_677 | -0.844162285327911 | 0.00212839548320387 |
| K01814 | neg\_677 | 0.818181812763214 | 0.00381492051076338 |
| K12072 | neg\_677 | -0.925451993942261 | 0.000123404671922511 |
| K09815 | neg\_677 | 0.890909075737 | 0.000542144516154419 |
| K00013 | neg\_677 | 0.806060612201691 | 0.0048620605246823 |
| K07121 | neg\_677 | -0.818181812763214 | 0.00381492051076338 |
| K02665 | neg\_677 | -0.819346487522125 | 0.00372367376291516 |
| K06871 | neg\_677 | 0.854545474052429 | 0.00163680247839526 |
| K16014 | neg\_677 | 0.881680607795715 | 0.000741594922435862 |
| K05802 | neg\_677 | -0.808135211467743 | 0.00466981917809761 |
| K00057 | neg\_677 | 0.818181812763214 | 0.00381492051076338 |
| K01649 | neg\_677 | 0.842424213886261 | 0.00222003275351312 |
| K19157 | neg\_681 | -0.830303013324738 | 0.00294022813612127 |
| K09951 | neg\_681 | -0.866666674613953 | 0.0011735379121256 |
| K05946 | neg\_685 | 0.866666674613953 | 0.0011735379121256 |
| K01159 | neg\_685 | 0.830303013324738 | 0.00294022813612127 |
| K04061 | neg\_685 | -0.890909075737 | 0.000542144516154419 |
| K01729 | neg\_685 | 0.818181812763214 | 0.00381492051076338 |
| K09761 | neg\_685 | 0.866666674613953 | 0.0011735379121256 |
| K04565 | neg\_685 | 0.842424213886261 | 0.00222003275351312 |
| K07139 | neg\_685 | 0.842424213886261 | 0.00222003275351312 |
| K05787 | neg\_685 | 0.927272737026215 | 0.000112034447641074 |
| K20452 | neg\_685 | -0.901908397674561 | 0.000359290276283897 |
| K00969 | neg\_685 | 0.806060612201691 | 0.0048620605246823 |
| K14575 | neg\_685 | 0.802435338497162 | 0.00521145970824932 |
| K05964 | neg\_685 | -0.890909075737 | 0.000542144516154419 |
| K02665 | neg\_708 | 0.826174378395081 | 0.00321991677364641 |
| K16014 | neg\_708 | -0.90043979883194 | 0.000380598613888061 |
| K06145 | neg\_708 | -0.806060612201691 | 0.0048620605246823 |
| K01060 | neg\_71 | 0.830303013324738 | 0.00294022813612127 |
| K11933 | neg\_71 | -0.865095794200897 | 0.00122739260821847 |
| K10237 | neg\_71 | -0.885716199874878 | 0.000648751005291714 |
| K04096 | neg\_71 | 0.842424213886261 | 0.00222003275351312 |
| K02461 | neg\_71 | -0.800390899181366 | 0.0054162086926719 |
| K04565 | neg\_71 | 0.866666674613953 | 0.0011735379121256 |
| K03607 | neg\_71 | -0.805690705776215 | 0.00489692183645341 |
| K18537 | neg\_71 | -0.903030276298523 | 0.000343612565232743 |
| K06311 | neg\_71 | -0.851067781448364 | 0.00179112909994994 |
| K07707 | neg\_71 | -0.806060612201691 | 0.0048620605246823 |
| K11933 | neg\_726 | -0.865095794200897 | 0.00122739260821847 |
| K02461 | neg\_726 | -0.856668412685394 | 0.00154747182074688 |
| K03651 | neg\_726 | 0.806060612201691 | 0.0048620605246823 |
| K19157 | neg\_726 | 0.854545474052429 | 0.00163680247839526 |
| K03414 | neg\_726 | -0.860313832759857 | 0.00140239962667921 |
| K05802 | neg\_726 | -0.937436878681183 | 6.21251750798635e-05 |
| K10942 | neg\_726 | -0.860313832759857 | 0.00140239962667921 |
| K06311 | neg\_726 | -0.887542068958282 | 0.000609666476501403 |
| K02919 | neg\_726 | -0.890909075737 | 0.000542144516154419 |
| K11933 | neg\_727 | -0.813664615154266 | 0.00418402797726447 |
| K17329 | neg\_727 | -0.896651387214661 | 0.000439837733652304 |
| K10237 | neg\_727 | -0.857381999492645 | 0.00151825527936911 |
| K04061 | neg\_727 | -0.816012322902679 | 0.0039891464803441 |
| K02461 | neg\_727 | -0.943208038806915 | 4.2483102273394e-05 |
| K01729 | neg\_727 | 0.840554058551788 | 0.00232177976116144 |
| K03651 | neg\_727 | 0.840554058551788 | 0.00232177976116144 |
| K06285 | neg\_727 | -0.816012322902679 | 0.0039891464803441 |
| K04565 | neg\_727 | 0.816012322902679 | 0.0039891464803441 |
| K02665 | neg\_727 | -0.870935380458832 | 0.00103587528006388 |
| K21217 | neg\_727 | -0.870935380458832 | 0.00103587528006388 |
| K03643 | neg\_727 | 0.80374151468277 | 0.00508357521208702 |
| K06926 | neg\_727 | 0.828283190727234 | 0.00307477558489877 |
| K19157 | neg\_727 | 0.914179265499115 | 0.000213749081869885 |
| K06145 | neg\_727 | 0.828283190727234 | 0.00307477558489877 |
| K03414 | neg\_727 | -0.8156378865242 | 0.00401978302331507 |
| K05802 | neg\_727 | -0.805022776126862 | 0.00496032148676617 |
| K05851 | neg\_727 | -0.82926344871521 | 0.00300893631849064 |
| K10942 | neg\_727 | -0.8156378865242 | 0.00401978302331507 |
| K18298 | neg\_727 | -0.80374151468277 | 0.00508357521208702 |
| K00057 | neg\_727 | 0.816012322902679 | 0.0039891464803441 |
| K17329 | neg\_757 | -0.846925735473633 | 0.0019883679465349 |
| K02461 | neg\_757 | -0.881680607795715 | 0.000741594922435862 |
| K03651 | neg\_757 | 0.854545474052429 | 0.00163680247839526 |
| K02665 | neg\_757 | -0.846658051013947 | 0.00200163182665913 |
| K05830 | neg\_757 | -0.806060612201691 | 0.0048620605246823 |
| K21217 | neg\_757 | -0.819346487522125 | 0.00372367376291516 |
| K06926 | neg\_757 | 0.818181812763214 | 0.00381492051076338 |
| K19157 | neg\_757 | 0.927272737026215 | 0.000112034447641074 |
| K06145 | neg\_757 | 0.818181812763214 | 0.00381492051076338 |
| K03414 | neg\_757 | -0.805690705776215 | 0.00489692183645341 |
| K05802 | neg\_757 | -0.885716199874878 | 0.000648751005291714 |
| K10942 | neg\_757 | -0.805690705776215 | 0.00489692183645341 |
| K06311 | neg\_757 | -0.814593434333801 | 0.00410613103846336 |
| K02919 | neg\_757 | -0.818181812763214 | 0.00381492051076338 |
| K00845 | neg\_758 | 0.878787875175476 | 0.000813862205061078 |
| K07672 | neg\_758 | 0.825403153896332 | 0.00327420560319291 |
| K01060 | neg\_758 | 0.866666674613953 | 0.0011735379121256 |
| K05946 | neg\_758 | 0.806060612201691 | 0.0048620605246823 |
| K07099 | neg\_758 | 0.806060612201691 | 0.0048620605246823 |
| K03827 | neg\_758 | -0.866666674613953 | 0.0011735379121256 |
| K10237 | neg\_758 | -0.885716199874878 | 0.000648751005291714 |
| K03406 | neg\_758 | -0.818181812763214 | 0.00381492051076338 |
| K04069 | neg\_758 | 0.806060612201691 | 0.0048620605246823 |
| K02745 | neg\_758 | -0.830303013324738 | 0.00294022813612127 |
| K00852 | neg\_758 | 0.806060612201691 | 0.0048620605246823 |
| K03225 | neg\_758 | -0.842424213886261 | 0.00222003275351312 |
| K03651 | neg\_758 | 0.806060612201691 | 0.0048620605246823 |
| K12072 | neg\_758 | -0.800390899181366 | 0.0054162086926719 |
| K07121 | neg\_758 | -0.806060612201691 | 0.0048620605246823 |
| K07396 | neg\_758 | -0.854545474052429 | 0.00163680247839526 |
| K03607 | neg\_758 | -0.805690705776215 | 0.00489692183645341 |
| K04748 | neg\_758 | -0.818181812763214 | 0.00381492051076338 |
| K18692 | neg\_758 | -0.854545474052429 | 0.00163680247839526 |
| K06145 | neg\_758 | 0.842424213886261 | 0.00222003275351312 |
| K17680 | neg\_758 | -0.818181812763214 | 0.00381492051076338 |
| K07341 | neg\_758 | 0.806060612201691 | 0.0048620605246823 |
| K03724 | neg\_758 | 0.854545474052429 | 0.00163680247839526 |
| K06305 | neg\_758 | -0.806060612201691 | 0.0048620605246823 |
| K01159 | neg\_763 | -0.842424213886261 | 0.00222003275351312 |
| K03470 | neg\_763 | -0.818181812763214 | 0.00381492051076338 |
| K07391 | neg\_763 | -0.854545474052429 | 0.00163680247839526 |
| K04061 | neg\_763 | 0.890909075737 | 0.000542144516154419 |
| K03524 | neg\_763 | -0.806060612201691 | 0.0048620605246823 |
| K07126 | neg\_763 | -0.830303013324738 | 0.00294022813612127 |
| K06167 | neg\_763 | -0.903030276298523 | 0.000343612565232743 |
| K09761 | neg\_763 | -0.854545474052429 | 0.00163680247839526 |
| K02852 | neg\_763 | -0.903030276298523 | 0.000343612565232743 |
| K15045 | neg\_763 | -0.878787875175476 | 0.000813862205061078 |
| K07139 | neg\_763 | -0.866666674613953 | 0.0011735379121256 |
| K05787 | neg\_763 | -0.903030276298523 | 0.000343612565232743 |
| K00059 | neg\_763 | -0.842424213886261 | 0.00222003275351312 |
| K03573 | neg\_763 | -0.818181812763214 | 0.00381492051076338 |
| K08688 | neg\_763 | -0.818181812763214 | 0.00381492051076338 |
| K06926 | neg\_763 | -0.915151536464691 | 0.000204472206099204 |
| K00783 | neg\_763 | -0.890909075737 | 0.000542144516154419 |
| K03101 | neg\_763 | -0.854545474052429 | 0.00163680247839526 |
| K11068 | neg\_763 | -0.842424213886261 | 0.00222003275351312 |
| K00969 | neg\_763 | -0.830303013324738 | 0.00294022813612127 |
| K07043 | neg\_763 | -0.818181812763214 | 0.00381492051076338 |
| K05970 | neg\_763 | -0.878787875175476 | 0.000813862205061078 |
| K14575 | neg\_763 | -0.881463050842285 | 0.000746860533753502 |
| K03584 | neg\_763 | -0.830303013324738 | 0.00294022813612127 |
| K09951 | neg\_763 | -0.818181812763214 | 0.00381492051076338 |
| K00845 | neg\_769 | -0.830303013324738 | 0.00294022813612127 |
| K07672 | neg\_769 | -0.844162285327911 | 0.00212839548320387 |
| K01060 | neg\_769 | -0.842424213886261 | 0.00222003275351312 |
| K05946 | neg\_769 | -0.927272737026215 | 0.000112034447641074 |
| K07099 | neg\_769 | -0.818181812763214 | 0.00381492051076338 |
| K03340 | neg\_769 | -0.818181812763214 | 0.00381492051076338 |
| K10237 | neg\_769 | 0.859855890274048 | 0.00142005806532719 |
| K01261 | neg\_769 | -0.830303013324738 | 0.00294022813612127 |
| K04061 | neg\_769 | 0.806060612201691 | 0.0048620605246823 |
| K01714 | neg\_769 | -0.818181812763214 | 0.00381492051076338 |
| K04069 | neg\_769 | -0.806060612201691 | 0.0048620605246823 |
| K07166 | neg\_769 | -0.878787875175476 | 0.000813862205061078 |
| K00852 | neg\_769 | -0.806060612201691 | 0.0048620605246823 |
| K09761 | neg\_769 | -0.830303013324738 | 0.00294022813612127 |
| K04565 | neg\_769 | -0.806060612201691 | 0.0048620605246823 |
| K13798 | neg\_769 | 0.818181812763214 | 0.00381492051076338 |
| K07396 | neg\_769 | 0.854545474052429 | 0.00163680247839526 |
| K03607 | neg\_769 | 0.833002269268036 | 0.00276710271221536 |
| K12231 | neg\_769 | 0.846658051013947 | 0.00200163182665913 |
| K20452 | neg\_769 | 0.80374151468277 | 0.00508357521208702 |
| K13611 | neg\_769 | 0.808135211467743 | 0.00466981917809761 |
| K00969 | neg\_769 | -0.818181812763214 | 0.00381492051076338 |
| K10578 | neg\_769 | 0.846658051013947 | 0.00200163182665913 |
| K01299 | neg\_769 | 0.854545474052429 | 0.00163680247839526 |
| K05964 | neg\_769 | 0.878787875175476 | 0.000813862205061078 |
| K03724 | neg\_769 | -0.830303013324738 | 0.00294022813612127 |
| K18198 | neg\_781 | 0.806060612201691 | 0.0048620605246823 |
| K17329 | neg\_781 | 0.859855890274048 | 0.00142005806532719 |
| K02461 | neg\_781 | 0.856668412685394 | 0.00154747182074688 |
| K02665 | neg\_781 | 0.805690705776215 | 0.00489692183645341 |
| K05802 | neg\_781 | 0.898646354675293 | 0.000407860267078952 |
| K09512 | neg\_781 | -0.833995580673218 | 0.00270528265085312 |
| K06311 | neg\_781 | 0.899700224399567 | 0.000391673296721073 |
| K18198 | neg\_783 | 0.842424213886261 | 0.00222003275351312 |
| K01159 | neg\_783 | -0.830303013324738 | 0.00294022813612127 |
| K07126 | neg\_783 | -0.806060612201691 | 0.0048620605246823 |
| K19411 | neg\_783 | -0.854545474052429 | 0.00163680247839526 |
| K02493 | neg\_783 | -0.915151536464691 | 0.000204472206099204 |
| K02784 | neg\_783 | -0.866666674613953 | 0.0011735379121256 |
| K06285 | neg\_783 | 0.818181812763214 | 0.00381492051076338 |
| K03769 | neg\_783 | -0.866666674613953 | 0.0011735379121256 |
| K17331 | neg\_783 | -0.846658051013947 | 0.00200163182665913 |
| K09761 | neg\_783 | -0.818181812763214 | 0.00381492051076338 |
| K19334 | neg\_783 | -0.830303013324738 | 0.00294022813612127 |
| K02665 | neg\_783 | 0.826174378395081 | 0.00321991677364641 |
| K03573 | neg\_783 | -0.842424213886261 | 0.00222003275351312 |
| K03643 | neg\_783 | -0.830303013324738 | 0.00294022813612127 |
| K13014 | neg\_783 | -0.854545474052429 | 0.00163680247839526 |
| K06145 | neg\_783 | -0.806060612201691 | 0.0048620605246823 |
| K05970 | neg\_783 | -0.890909075737 | 0.000542144516154419 |
| K01409 | neg\_783 | -0.842424213886261 | 0.00222003275351312 |
| K00919 | neg\_783 | -0.806060612201691 | 0.0048620605246823 |
| K20525 | neg\_783 | -0.951515138149261 | 2.27985739738035e-05 |
| K17329 | neg\_794 | -0.885716199874878 | 0.000648751005291714 |
| K02461 | neg\_794 | -0.931705057621002 | 8.75967112814457e-05 |
| K02665 | neg\_794 | -0.887625336647034 | 0.000607926148504134 |
| K21217 | neg\_794 | -0.833002269268036 | 0.00276710271221536 |
| K19157 | neg\_794 | 0.927272737026215 | 0.000112034447641074 |
| K05802 | neg\_794 | -0.821065366268158 | 0.00359188206024896 |
| K15652 | neg\_826 | 0.866666674613953 | 0.0011735379121256 |
| K11933 | neg\_84 | 0.938721001148224 | 5.72697606402439e-05 |
| K10237 | neg\_84 | 0.846925735473633 | 0.0019883679465349 |
| K03524 | neg\_84 | -0.806060612201691 | 0.0048620605246823 |
| K04096 | neg\_84 | -0.866666674613953 | 0.0011735379121256 |
| K02461 | neg\_84 | 0.81915009021759 | 0.00373894966455346 |
| K02458 | neg\_84 | 0.950991809368134 | 2.37837330223645e-05 |
| K04565 | neg\_84 | -0.830303013324738 | 0.00294022813612127 |
| K07031 | neg\_84 | 0.830303013324738 | 0.00294022813612127 |
| K03607 | neg\_84 | 0.805690705776215 | 0.00489692183645341 |
| K00849 | neg\_84 | -0.806060612201691 | 0.0048620605246823 |
| K02521 | neg\_84 | 0.854545474052429 | 0.00163680247839526 |
| K00014 | neg\_84 | -0.830303013324738 | 0.00294022813612127 |
| K18537 | neg\_84 | 0.939393937587738 | 5.48405364009241e-05 |
| K02919 | neg\_84 | 0.818181812763214 | 0.00381492051076338 |
| K04075 | neg\_842 | 0.818181812763214 | 0.00381492051076338 |
| K00817 | neg\_842 | 0.903030276298523 | 0.000343612565232743 |
| K02493 | neg\_842 | 0.830303013324738 | 0.00294022813612127 |
| K12072 | neg\_842 | -0.81915009021759 | 0.00373894966455346 |
| K09815 | neg\_842 | 0.842424213886261 | 0.00222003275351312 |
| K02852 | neg\_842 | 0.842424213886261 | 0.00222003275351312 |
| K02665 | neg\_842 | -0.833002269268036 | 0.00276710271221536 |
| K21217 | neg\_842 | -0.887625336647034 | 0.000607926148504134 |
| K03643 | neg\_842 | 0.830303013324738 | 0.00294022813612127 |
| K06871 | neg\_842 | 0.806060612201691 | 0.0048620605246823 |
| K00849 | neg\_842 | 0.806060612201691 | 0.0048620605246823 |
| K03414 | neg\_842 | -0.846658051013947 | 0.00200163182665913 |
| K05851 | neg\_842 | -0.925451993942261 | 0.000123404671922511 |
| K10942 | neg\_842 | -0.846658051013947 | 0.00200163182665913 |
| K18198 | neg\_846 | 0.821065366268158 | 0.00359188206024896 |
| K02493 | neg\_846 | -0.924506723880768 | 0.000129632545059266 |
| K03769 | neg\_846 | -0.808135211467743 | 0.00466981917809761 |
| K19334 | neg\_846 | -0.821065366268158 | 0.00359188206024896 |
| K02665 | neg\_846 | 0.903162717819214 | 0.0003417954885645 |
| K21217 | neg\_846 | 0.932296991348267 | 8.46607002542576e-05 |
| K03643 | neg\_846 | -0.808135211467743 | 0.00466981917809761 |
| K13014 | neg\_846 | -0.833995580673218 | 0.00270528265085312 |
| K03414 | neg\_846 | 0.888595581054688 | 0.000587914326861227 |
| K10942 | neg\_846 | 0.888595581054688 | 0.000587914326861227 |
| K20525 | neg\_846 | -0.911576509475708 | 0.000240107073119944 |
| K11933 | neg\_861 | -0.816012322902679 | 0.0039891464803441 |
| K10237 | neg\_861 | -0.821065366268158 | 0.00359188206024896 |
| K02461 | neg\_861 | -0.881680607795715 | 0.000741594922435862 |
| K03651 | neg\_861 | 0.842424213886261 | 0.00222003275351312 |
| K05830 | neg\_861 | -0.854545474052429 | 0.00163680247839526 |
| K21217 | neg\_861 | -0.833002269268036 | 0.00276710271221536 |
| K19157 | neg\_861 | 0.890909075737 | 0.000542144516154419 |
| K16014 | neg\_861 | 0.844162285327911 | 0.00212839548320387 |
| K06145 | neg\_861 | 0.806060612201691 | 0.0048620605246823 |
| K03414 | neg\_861 | -0.873969614505768 | 0.000945449010965937 |
| K05802 | neg\_861 | -0.924506723880768 | 0.000129632545059266 |
| K10942 | neg\_861 | -0.873969614505768 | 0.000945449010965937 |
| K06311 | neg\_861 | -0.832830607891083 | 0.00277788845123905 |
| K02919 | neg\_861 | -0.866666674613953 | 0.0011735379121256 |
| K18198 | neg\_911 | -0.854545474052429 | 0.00163680247839526 |
| K00640 | neg\_911 | 0.806060612201691 | 0.0048620605246823 |
| K11933 | neg\_911 | -0.889637529850006 | 0.000566964581027118 |
| K17329 | neg\_911 | -0.821065366268158 | 0.00359188206024896 |
| K10237 | neg\_911 | -0.885716199874878 | 0.000648751005291714 |
| K04096 | neg\_911 | 0.866666674613953 | 0.0011735379121256 |
| K02461 | neg\_911 | -0.869174480438232 | 0.00109115185763287 |
| K04565 | neg\_911 | 0.842424213886261 | 0.00222003275351312 |
| K21217 | neg\_911 | -0.805690705776215 | 0.00489692183645341 |
| K00849 | neg\_911 | 0.818181812763214 | 0.00381492051076338 |
| K18537 | neg\_911 | -0.842424213886261 | 0.00222003275351312 |
| K03414 | neg\_911 | -0.873969614505768 | 0.000945449010965937 |
| K05802 | neg\_911 | -0.885716199874878 | 0.000648751005291714 |
| K10942 | neg\_911 | -0.873969614505768 | 0.000945449010965937 |
| K06311 | neg\_911 | -0.924016416072845 | 0.000132952728702396 |
| K02919 | neg\_911 | -0.830303013324738 | 0.00294022813612127 |
| K00845 | neg\_919 | 0.866666674613953 | 0.0011735379121256 |
| K01060 | neg\_919 | 0.866666674613953 | 0.0011735379121256 |
| K03827 | neg\_919 | -0.866666674613953 | 0.0011735379121256 |
| K10237 | neg\_919 | -0.859855890274048 | 0.00142005806532719 |
| K00100 | neg\_919 | -0.806060612201691 | 0.0048620605246823 |
| K03225 | neg\_919 | -0.866666674613953 | 0.0011735379121256 |
| K12072 | neg\_919 | -0.800390899181366 | 0.0054162086926719 |
| K07121 | neg\_919 | -0.830303013324738 | 0.00294022813612127 |
| K07396 | neg\_919 | -0.866666674613953 | 0.0011735379121256 |
| K03607 | neg\_919 | -0.833002269268036 | 0.00276710271221536 |
| K12231 | neg\_919 | -0.846658051013947 | 0.00200163182665913 |
| K04748 | neg\_919 | -0.806060612201691 | 0.0048620605246823 |
| K13611 | neg\_919 | -0.808135211467743 | 0.00466981917809761 |
| K18537 | neg\_919 | -0.854545474052429 | 0.00163680247839526 |
| K18692 | neg\_919 | -0.842424213886261 | 0.00222003275351312 |
| K17680 | neg\_919 | -0.830303013324738 | 0.00294022813612127 |
| K07341 | neg\_919 | 0.806060612201691 | 0.0048620605246823 |
| K02491 | neg\_919 | -0.828283190727234 | 0.00307477558489877 |
| K03724 | neg\_919 | 0.818181812763214 | 0.00381492051076338 |
| K18198 | neg\_94 | 0.806060612201691 | 0.0048620605246823 |
| K17329 | neg\_94 | 0.859855890274048 | 0.00142005806532719 |
| K02461 | neg\_94 | 0.856668412685394 | 0.00154747182074688 |
| K02665 | neg\_94 | 0.805690705776215 | 0.00489692183645341 |
| K05802 | neg\_94 | 0.898646354675293 | 0.000407860267078952 |
| K09512 | neg\_94 | -0.833995580673218 | 0.00270528265085312 |
| K06311 | neg\_94 | 0.899700224399567 | 0.000391673296721073 |
| K18198 | neg\_985 | -0.818181812763214 | 0.00381492051076338 |
| K01060 | neg\_985 | 0.842424213886261 | 0.00222003275351312 |
| K11933 | neg\_985 | -0.852824926376343 | 0.00171189540742711 |
| K17329 | neg\_985 | -0.872786045074463 | 0.000980007503875058 |
| K04061 | neg\_985 | -0.878787875175476 | 0.000813862205061078 |
| K03406 | neg\_985 | -0.830303013324738 | 0.00294022813612127 |
| K04096 | neg\_985 | 0.818181812763214 | 0.00381492051076338 |
| K02745 | neg\_985 | -0.866666674613953 | 0.0011735379121256 |
| K19411 | neg\_985 | 0.866666674613953 | 0.0011735379121256 |
| K03412 | neg\_985 | -0.830303013324738 | 0.00294022813612127 |
| K03651 | neg\_985 | 0.842424213886261 | 0.00222003275351312 |
| K06285 | neg\_985 | -0.854545474052429 | 0.00163680247839526 |
| K04565 | neg\_985 | 0.842424213886261 | 0.00222003275351312 |
| K03643 | neg\_985 | 0.806060612201691 | 0.0048620605246823 |
| K06926 | neg\_985 | 0.818181812763214 | 0.00381492051076338 |
| K13014 | neg\_985 | 0.842424213886261 | 0.00222003275351312 |
| K04748 | neg\_985 | -0.842424213886261 | 0.00222003275351312 |
| K18692 | neg\_985 | -0.842424213886261 | 0.00222003275351312 |
| K01524 | neg\_985 | 0.842424213886261 | 0.00222003275351312 |
| K06145 | neg\_985 | 0.842424213886261 | 0.00222003275351312 |
| K03414 | neg\_985 | -0.819346487522125 | 0.00372367376291516 |
| K05802 | neg\_985 | -0.808135211467743 | 0.00466981917809761 |
| K05851 | neg\_985 | -0.812897026538849 | 0.00424919679863534 |
| K10942 | neg\_985 | -0.819346487522125 | 0.00372367376291516 |
| K17329 | neg\_994 | -0.924506723880768 | 0.000129632545059266 |
| K01159 | neg\_994 | 0.878787875175476 | 0.000813862205061078 |
| K03470 | neg\_994 | 0.878787875175476 | 0.000813862205061078 |
| K11754 | neg\_994 | 0.830303013324738 | 0.00294022813612127 |
| K00100 | neg\_994 | -0.842424213886261 | 0.00222003275351312 |
| K07126 | neg\_994 | 0.854545474052429 | 0.00163680247839526 |
| K00817 | neg\_994 | 0.830303013324738 | 0.00294022813612127 |
| K19411 | neg\_994 | 0.878787875175476 | 0.000813862205061078 |
| K02493 | neg\_994 | 0.927272737026215 | 0.000112034447641074 |
| K02784 | neg\_994 | 0.854545474052429 | 0.00163680247839526 |
| K06285 | neg\_994 | -0.915151536464691 | 0.000204472206099204 |
| K03769 | neg\_994 | 0.915151536464691 | 0.000204472206099204 |
| K12072 | neg\_994 | -0.844162285327911 | 0.00212839548320387 |
| K17331 | neg\_994 | 0.819346487522125 | 0.00372367376291516 |
| K09815 | neg\_994 | 0.939393937587738 | 5.48405364009241e-05 |
| K02852 | neg\_994 | 0.842424213886261 | 0.00222003275351312 |
| K02665 | neg\_994 | -0.887625336647034 | 0.000607926148504134 |
| K21217 | neg\_994 | -0.833002269268036 | 0.00276710271221536 |
| K13665 | neg\_994 | -0.903030276298523 | 0.000343612565232743 |
| K00059 | neg\_994 | 0.830303013324738 | 0.00294022813612127 |
| K03573 | neg\_994 | 0.842424213886261 | 0.00222003275351312 |
| K03643 | neg\_994 | 0.903030276298523 | 0.000343612565232743 |
| K06179 | neg\_994 | 0.830303013324738 | 0.00294022813612127 |
| K02802 | neg\_994 | 0.846658051013947 | 0.00200163182665913 |
| K06926 | neg\_994 | 0.806060612201691 | 0.0048620605246823 |
| K00783 | neg\_994 | 0.806060612201691 | 0.0048620605246823 |
| K03501 | neg\_994 | 0.806060612201691 | 0.0048620605246823 |
| K00849 | neg\_994 | 0.854545474052429 | 0.00163680247839526 |
| K00969 | neg\_994 | 0.818181812763214 | 0.00381492051076338 |
| K05970 | neg\_994 | 0.806060612201691 | 0.0048620605246823 |
| K05851 | neg\_994 | -0.825403153896332 | 0.00327420560319291 |
| K01409 | neg\_994 | 0.903030276298523 | 0.000343612565232743 |
| K00919 | neg\_994 | 0.806060612201691 | 0.0048620605246823 |
| K00057 | neg\_994 | 0.842424213886261 | 0.00222003275351312 |
