# Supplementary material for: Multi-omics analyses reveal that the gut microbiome and its metabolites promote milk fat synthesis in Zhongdian yak cows
Source: PeerJ. 2022 Dec 2;10:e14444. doi: 10.7717/peerj.14444 (PMC9744170; doi:10.7717/peerj.14444)
Supplement: Supplemental Information 12 [file peerj-10-14444-s012.zip › Web_Report/HTML/treat1_H_L.vs.H_L_taxonomy_cor_filter.xls.html]

treat1\_H\_L.vs.H\_L\_taxonomy\_cor\_filter.xls


## treat1\_H\_L.vs.H\_L\_taxonomy\_cor\_filter.xls

| genusName | metaName | CC | CCP |
| --- | --- | --- | --- |
| Chloroflexus | neg\_1004 | -0.856668412685394 | 0.00154747182074688 |
| Pararhodospirillum | neg\_1004 | -0.825403153896332 | 0.00327420560319291 |
| Thermosyntropha | neg\_1004 | -0.800390899181366 | 0.0054162086926719 |
| Parafilimonas | neg\_1004 | 0.894186735153198 | 0.000481835628326488 |
| Emticicia | neg\_1062 | -0.830303013324738 | 0.00294022813612127 |
| Brevinema | neg\_1065 | 0.806060612201691 | 0.0048620605246823 |
| Candidatus\_Sulfopaludibacter | neg\_1065 | 0.842424213886261 | 0.00222003275351312 |
| Comamonas | neg\_1065 | -0.830303013324738 | 0.00294022813612127 |
| Candidatus\_Electrothrix | neg\_1065 | 0.818181812763214 | 0.00381492051076338 |
| Thermopetrobacter | neg\_1065 | 0.830303013324738 | 0.00294022813612127 |
| Rubrivivax | neg\_1065 | -0.842424213886261 | 0.00222003275351312 |
| Lihuaxuella | neg\_1109 | -0.854545474052429 | 0.00163680247839526 |
| Izhakiella | neg\_1109 | -0.924016416072845 | 0.000132952728702396 |
| Amantichitinum | neg\_1109 | -0.854545474052429 | 0.00163680247839526 |
| Candidatus\_Arcanobacter | neg\_1109 | -0.890909075737 | 0.000542144516154419 |
| Litorimonas | neg\_1116 | 0.806060612201691 | 0.0048620605246823 |
| Chloroflexus | neg\_1116 | 0.842424213886261 | 0.00222003275351312 |
| Porticoccus | neg\_1116 | 0.818181812763214 | 0.00381492051076338 |
| Parafilimonas | neg\_1116 | -0.866666674613953 | 0.0011735379121256 |
| Thermobifida | neg\_112 | -0.83790922164917 | 0.00247136195299369 |
| Litorimonas | neg\_112 | 0.856668412685394 | 0.00154747182074688 |
| Haliscomenobacter | neg\_112 | 0.881680607795715 | 0.000741594922435862 |
| Zhihengliuella | neg\_112 | -0.894186735153198 | 0.000481835628326488 |
| Lawsonella | neg\_112 | 0.853004157543182 | 0.00170395875980178 |
| Rodentibacter | neg\_112 | 0.806643962860107 | 0.00480744414521128 |
| Izhakiella | neg\_112 | 0.853004157543182 | 0.00170395875980178 |
| Desulfocucumis | neg\_112 | -0.800390899181366 | 0.0054162086926719 |
| Porticoccus | neg\_112 | 0.806643962860107 | 0.00480744414521128 |
| Succinimonas | neg\_112 | -0.881680607795715 | 0.000741594922435862 |
| Mongoliibacter | neg\_1139 | -0.878787875175476 | 0.000813862205061078 |
| Suttonella | neg\_1139 | 0.830303013324738 | 0.00294022813612127 |
| Litorimonas | neg\_1188 | -0.830303013324738 | 0.00294022813612127 |
| Rodentibacter | neg\_1188 | -0.806060612201691 | 0.0048620605246823 |
| Candidatus\_Sulfopaludibacter | neg\_1188 | -0.806060612201691 | 0.0048620605246823 |
| Dolosicoccus | neg\_1188 | 0.806060612201691 | 0.0048620605246823 |
| Thermopetrobacter | neg\_1188 | -0.806060612201691 | 0.0048620605246823 |
| Porticoccus | neg\_1188 | -0.927272737026215 | 0.000112034447641074 |
| Thermobifida | neg\_1307 | -0.842424213886261 | 0.00222003275351312 |
| Stappia | neg\_1307 | 0.806060612201691 | 0.0048620605246823 |
| Litorimonas | neg\_1307 | 0.830303013324738 | 0.00294022813612127 |
| Haliscomenobacter | neg\_1307 | 0.806060612201691 | 0.0048620605246823 |
| Zhihengliuella | neg\_1307 | -0.830303013324738 | 0.00294022813612127 |
| Acetivibrio | neg\_1307 | -0.818181812763214 | 0.00381492051076338 |
| Harryflintia | neg\_1307 | 0.806060612201691 | 0.0048620605246823 |
| Desulfocucumis | neg\_1307 | -0.866666674613953 | 0.0011735379121256 |
| Succinimonas | neg\_1307 | -0.806060612201691 | 0.0048620605246823 |
| Litorimonas | neg\_1330 | 0.828283190727234 | 0.00307477558489877 |
| Zhihengliuella | neg\_1330 | -0.914179265499115 | 0.000213749081869885 |
| Lawsonella | neg\_1330 | 0.80618816614151 | 0.00485008058916403 |
| Rodentibacter | neg\_1330 | 0.865095794200897 | 0.00122739260821847 |
| Marivita | neg\_1330 | -0.816012322902679 | 0.0039891464803441 |
| Izhakiella | neg\_1330 | 0.843112826347351 | 0.00218339276204738 |
| Porticoccus | neg\_1330 | 0.80374151468277 | 0.00508357521208702 |
| Succinimonas | neg\_1330 | -0.816012322902679 | 0.0039891464803441 |
| Lihuaxuella | neg\_1354 | -0.806060612201691 | 0.0048620605246823 |
| Candidatus\_Sulfopaludibacter | neg\_1354 | -0.830303013324738 | 0.00294022813612127 |
| Thermopetrobacter | neg\_1354 | -0.842424213886261 | 0.00222003275351312 |
| Thermobifida | neg\_136 | 0.854545474052429 | 0.00163680247839526 |
| Stappia | neg\_136 | -0.818181812763214 | 0.00381492051076338 |
| Anaerotignum | neg\_136 | 0.854545474052429 | 0.00163680247839526 |
| Erythrobacter | neg\_136 | -0.842424213886261 | 0.00222003275351312 |
| Litorimonas | neg\_136 | -0.806060612201691 | 0.0048620605246823 |
| Brevinema | neg\_136 | -0.866666674613953 | 0.0011735379121256 |
| Pakpunavirus | neg\_136 | -0.802435338497162 | 0.00521145970824932 |
| Ottowia | neg\_136 | 0.842424213886261 | 0.00222003275351312 |
| Zhihengliuella | neg\_136 | 0.830303013324738 | 0.00294022813612127 |
| Lihuaxuella | neg\_136 | -0.842424213886261 | 0.00222003275351312 |
| Rodentibacter | neg\_136 | -0.854545474052429 | 0.00163680247839526 |
| Candidatus\_Sulfopaludibacter | neg\_136 | -0.866666674613953 | 0.0011735379121256 |
| Palleronia | neg\_136 | 0.814593434333801 | 0.00410613103846336 |
| Marivita | neg\_136 | 0.842424213886261 | 0.00222003275351312 |
| Acetivibrio | neg\_136 | 0.903030276298523 | 0.000343612565232743 |
| Turicibacter | neg\_136 | -0.818181812763214 | 0.00381492051076338 |
| Dolosicoccus | neg\_136 | 0.830303013324738 | 0.00294022813612127 |
| Izhakiella | neg\_136 | -0.820672512054443 | 0.00362170350001234 |
| Candidatus\_Electrothrix | neg\_136 | -0.806060612201691 | 0.0048620605246823 |
| Thiodictyon | neg\_136 | 0.842424213886261 | 0.00222003275351312 |
| Desulfocucumis | neg\_136 | 0.890909075737 | 0.000542144516154419 |
| Promicromonospora | neg\_136 | -0.806060612201691 | 0.0048620605246823 |
| Mudcatvirus | neg\_136 | -0.830303013324738 | 0.00294022813612127 |
| Thermobifida | neg\_1361 | -0.830303013324738 | 0.00294022813612127 |
| Litorimonas | neg\_1361 | 0.903030276298523 | 0.000343612565232743 |
| Brevinema | neg\_1361 | 0.878787875175476 | 0.000813862205061078 |
| Chloroflexus | neg\_1361 | 0.830303013324738 | 0.00294022813612127 |
| Candidatus\_Tokpelaia | neg\_1361 | 0.830303013324738 | 0.00294022813612127 |
| Haliscomenobacter | neg\_1361 | 0.830303013324738 | 0.00294022813612127 |
| Zhihengliuella | neg\_1361 | -0.890909075737 | 0.000542144516154419 |
| Lawsonella | neg\_1361 | 0.802435338497162 | 0.00521145970824932 |
| Acetivibrio | neg\_1361 | -0.830303013324738 | 0.00294022813612127 |
| Limnobacter | neg\_1361 | 0.869304955005646 | 0.00108698415655106 |
| Lmd1virus | neg\_1361 | -0.881680607795715 | 0.000741594922435862 |
| Dolosicoccus | neg\_1361 | -0.818181812763214 | 0.00381492051076338 |
| Dactylococcopsis | neg\_1361 | -0.842424213886261 | 0.00222003275351312 |
| Porticoccus | neg\_1361 | 0.806060612201691 | 0.0048620605246823 |
| Eah2virus | neg\_1361 | 0.833002269268036 | 0.00276710271221536 |
| Lacinutrix | neg\_1363 | 0.939393937587738 | 5.48405364009241e-05 |
| Thermobifida | neg\_1382 | -0.842424213886261 | 0.00222003275351312 |
| Stappia | neg\_1382 | 0.854545474052429 | 0.00163680247839526 |
| Caloramator | neg\_1382 | 0.818181812763214 | 0.00381492051076338 |
| Anaerotignum | neg\_1382 | -0.854545474052429 | 0.00163680247839526 |
| Erythrobacter | neg\_1382 | 0.830303013324738 | 0.00294022813612127 |
| Litorimonas | neg\_1382 | 0.830303013324738 | 0.00294022813612127 |
| Zhihengliuella | neg\_1382 | -0.806060612201691 | 0.0048620605246823 |
| Lawsonella | neg\_1382 | 0.869304955005646 | 0.00108698415655106 |
| Rodentibacter | neg\_1382 | 0.830303013324738 | 0.00294022813612127 |
| Marivita | neg\_1382 | -0.818181812763214 | 0.00381492051076338 |
| Acetivibrio | neg\_1382 | -0.830303013324738 | 0.00294022813612127 |
| Harryflintia | neg\_1382 | 0.806060612201691 | 0.0048620605246823 |
| Izhakiella | neg\_1382 | 0.808514356613159 | 0.00463528246178302 |
| Candidatus\_Sulfotelmatomonas | neg\_1382 | 0.818181812763214 | 0.00381492051076338 |
| Desulfocucumis | neg\_1382 | -0.903030276298523 | 0.000343612565232743 |
| Promicromonospora | neg\_1382 | 0.806060612201691 | 0.0048620605246823 |
| Mudcatvirus | neg\_1382 | 0.830303013324738 | 0.00294022813612127 |
| Succinimonas | neg\_1382 | -0.806060612201691 | 0.0048620605246823 |
| Anaerotignum | neg\_1401 | 0.830303013324738 | 0.00294022813612127 |
| Brevinema | neg\_1401 | -0.830303013324738 | 0.00294022813612127 |
| Parapedobacter | neg\_1401 | -0.878787875175476 | 0.000813862205061078 |
| Mongoliibacter | neg\_1401 | -0.842424213886261 | 0.00222003275351312 |
| Suttonella | neg\_1401 | 0.878787875175476 | 0.000813862205061078 |
| Limnobacter | neg\_1401 | -0.851067781448364 | 0.00179112909994994 |
| Harryflintia | neg\_1401 | -0.854545474052429 | 0.00163680247839526 |
| Comamonas | neg\_1401 | 0.842424213886261 | 0.00222003275351312 |
| Lacinutrix | neg\_1401 | -0.842424213886261 | 0.00222003275351312 |
| Candidatus\_Symbiobacter | neg\_1401 | -0.878787875175476 | 0.000813862205061078 |
| Thermobifida | neg\_1413 | 0.903030276298523 | 0.000343612565232743 |
| Stappia | neg\_1413 | -0.854545474052429 | 0.00163680247839526 |
| Brevinema | neg\_1413 | -0.866666674613953 | 0.0011735379121256 |
| Candidatus\_Tokpelaia | neg\_1413 | -0.915151536464691 | 0.000204472206099204 |
| Haliscomenobacter | neg\_1413 | -0.890909075737 | 0.000542144516154419 |
| Suttonella | neg\_1413 | 0.806060612201691 | 0.0048620605246823 |
| Kordiimonas | neg\_1413 | -0.818181812763214 | 0.00381492051076338 |
| Candidatus\_Electrothrix | neg\_1413 | -0.854545474052429 | 0.00163680247839526 |
| Beggiatoa | neg\_1413 | -0.842424213886261 | 0.00222003275351312 |
| Succinimonas | neg\_1413 | 0.830303013324738 | 0.00294022813612127 |
| Candidatus\_Tokpelaia | neg\_1432 | -0.866666674613953 | 0.0011735379121256 |
| Haliscomenobacter | neg\_1432 | -0.842424213886261 | 0.00222003275351312 |
| Catenovulum | neg\_1438 | -0.818181812763214 | 0.00381492051076338 |
| Anaerotignum | neg\_1438 | -0.818181812763214 | 0.00381492051076338 |
| Kriegella | neg\_1438 | 0.939393937587738 | 5.48405364009241e-05 |
| Candidatus\_Kryptonium | neg\_1438 | 0.842424213886261 | 0.00222003275351312 |
| Mycolicibacter | neg\_1438 | -0.806060612201691 | 0.0048620605246823 |
| Parapedobacter | neg\_1438 | 0.927272737026215 | 0.000112034447641074 |
| Propionicimonas | neg\_1438 | 0.899700224399567 | 0.000391673296721073 |
| Palleronia | neg\_1438 | -0.869304955005646 | 0.00108698415655106 |
| Sinirhodobacter | neg\_1438 | -0.890909075737 | 0.000542144516154419 |
| Microscilla | neg\_1438 | 0.866666674613953 | 0.0011735379121256 |
| Limnobacter | neg\_1438 | 0.875383973121643 | 0.00090532610126548 |
| Thermoanaerobacter | neg\_1438 | 0.818181812763214 | 0.00381492051076338 |
| Jiangella | neg\_1438 | 0.854545474052429 | 0.00163680247839526 |
| Negativicoccus | neg\_1438 | -0.806060612201691 | 0.0048620605246823 |
| Acidaminobacter | neg\_1438 | 0.842424213886261 | 0.00222003275351312 |
| Sideroxydans | neg\_1438 | -0.975757598876953 | 1.46754063035104e-06 |
| Mailhella | neg\_1438 | -0.915151536464691 | 0.000204472206099204 |
| Synergistes | neg\_1438 | -0.854545474052429 | 0.00163680247839526 |
| Anoxybacillus | neg\_1438 | 0.842424213886261 | 0.00222003275351312 |
| Flexilinea | neg\_1438 | -0.927272737026215 | 0.000112034447641074 |
| Haliscomenobacter | neg\_1450 | 0.846925735473633 | 0.0019883679465349 |
| Zhihengliuella | neg\_1450 | -0.833995580673218 | 0.00270528265085312 |
| Izhakiella | neg\_1450 | 0.849503874778748 | 0.00186385232781783 |
| Succinimonas | neg\_1450 | -0.846925735473633 | 0.0019883679465349 |
| Anaerotignum | neg\_1496 | -0.915151536464691 | 0.000204472206099204 |
| Erythrobacter | neg\_1496 | 0.866666674613953 | 0.0011735379121256 |
| Litorimonas | neg\_1496 | 0.890909075737 | 0.000542144516154419 |
| Brevinema | neg\_1496 | 0.818181812763214 | 0.00381492051076338 |
| Parapedobacter | neg\_1496 | 0.830303013324738 | 0.00294022813612127 |
| Zhihengliuella | neg\_1496 | -0.866666674613953 | 0.0011735379121256 |
| Lawsonella | neg\_1496 | 0.826751530170441 | 0.00317971472634282 |
| Rodentibacter | neg\_1496 | 0.915151536464691 | 0.000204472206099204 |
| Marivita | neg\_1496 | -0.830303013324738 | 0.00294022813612127 |
| Larkinella | neg\_1496 | 0.806060612201691 | 0.0048620605246823 |
| Limnobacter | neg\_1496 | 0.83890962600708 | 0.00241398974898654 |
| Harryflintia | neg\_1496 | 0.842424213886261 | 0.00222003275351312 |
| Trichormus | neg\_1496 | -0.830303013324738 | 0.00294022813612127 |
| Izhakiella | neg\_1496 | 0.899700224399567 | 0.000391673296721073 |
| Desulfocucumis | neg\_1496 | -0.878787875175476 | 0.000813862205061078 |
| Porticoccus | neg\_1496 | 0.854545474052429 | 0.00163680247839526 |
| Succinimonas | neg\_1496 | -0.830303013324738 | 0.00294022813612127 |
| Caloramator | neg\_1532 | -0.903030276298523 | 0.000343612565232743 |
| Erythrobacter | neg\_1532 | -0.939393937587738 | 5.48405364009241e-05 |
| Kriegella | neg\_1532 | -0.842424213886261 | 0.00222003275351312 |
| Litorimonas | neg\_1532 | -0.806060612201691 | 0.0048620605246823 |
| Brevinema | neg\_1532 | -0.866666674613953 | 0.0011735379121256 |
| Chloroflexus | neg\_1532 | -0.830303013324738 | 0.00294022813612127 |
| Candidatus\_Moduliflexus | neg\_1532 | -0.951515138149261 | 2.27985739738035e-05 |
| Pakpunavirus | neg\_1532 | -0.826751530170441 | 0.00317971472634282 |
| Propionicimonas | neg\_1532 | -0.808514356613159 | 0.00463528246178302 |
| Rodentibacter | neg\_1532 | -0.878787875175476 | 0.000813862205061078 |
| Candidatus\_Sulfopaludibacter | neg\_1532 | -0.830303013324738 | 0.00294022813612127 |
| Marivita | neg\_1532 | 0.927272737026215 | 0.000112034447641074 |
| Acetivibrio | neg\_1532 | 0.806060612201691 | 0.0048620605246823 |
| Microscilla | neg\_1532 | -0.830303013324738 | 0.00294022813612127 |
| Gottschalkia | neg\_1532 | -0.830303013324738 | 0.00294022813612127 |
| Lmd1virus | neg\_1532 | 0.806643962860107 | 0.00480744414521128 |
| Trichormus | neg\_1532 | 0.830303013324738 | 0.00294022813612127 |
| Comamonas | neg\_1532 | 0.878787875175476 | 0.000813862205061078 |
| Candidatus\_Electrothrix | neg\_1532 | -0.806060612201691 | 0.0048620605246823 |
| Cvm10virus | neg\_1532 | -0.881680607795715 | 0.000741594922435862 |
| Mudcatvirus | neg\_1532 | -0.842424213886261 | 0.00222003275351312 |
| Pacificibacter | neg\_1532 | -0.818181812763214 | 0.00381492051076338 |
| Eah2virus | neg\_1532 | -0.805690705776215 | 0.00489692183645341 |
| Candidatus\_Halobonum | neg\_1532 | -0.826751530170441 | 0.00317971472634282 |
| Caloramator | neg\_1563 | -0.818181812763214 | 0.00381492051076338 |
| Candidatus\_Moduliflexus | neg\_1563 | -0.890909075737 | 0.000542144516154419 |
| Pakpunavirus | neg\_1563 | -0.814593434333801 | 0.00410613103846336 |
| Candidatus\_Sulfopaludibacter | neg\_1563 | -0.854545474052429 | 0.00163680247839526 |
| Alkalibacterium | neg\_1563 | -0.866666674613953 | 0.0011735379121256 |
| Kordiimonas | neg\_1563 | -0.830303013324738 | 0.00294022813612127 |
| Candidatus\_Electrothrix | neg\_1563 | -0.915151536464691 | 0.000204472206099204 |
| Cvm10virus | neg\_1563 | -0.894186735153198 | 0.000481835628326488 |
| Pacificibacter | neg\_1563 | -0.878787875175476 | 0.000813862205061078 |
| Metaprevotella | neg\_1563 | -0.806060612201691 | 0.0048620605246823 |
| Anaerotignum | neg\_162 | -0.866666674613953 | 0.0011735379121256 |
| Brevinema | neg\_162 | 0.890909075737 | 0.000542144516154419 |
| Mycolicibacter | neg\_162 | -0.842424213886261 | 0.00222003275351312 |
| Parapedobacter | neg\_162 | 0.939393937587738 | 5.48405364009241e-05 |
| Palleronia | neg\_162 | -0.802435338497162 | 0.00521145970824932 |
| Mongoliibacter | neg\_162 | 0.927272737026215 | 0.000112034447641074 |
| Marivita | neg\_162 | -0.818181812763214 | 0.00381492051076338 |
| Suttonella | neg\_162 | -0.854545474052429 | 0.00163680247839526 |
| Limnobacter | neg\_162 | 0.83890962600708 | 0.00241398974898654 |
| Sideroxydans | neg\_162 | -0.903030276298523 | 0.000343612565232743 |
| Comamonas | neg\_162 | -0.806060612201691 | 0.0048620605246823 |
| Anoxybacillus | neg\_162 | 0.866666674613953 | 0.0011735379121256 |
| Flexilinea | neg\_162 | -0.830303013324738 | 0.00294022813612127 |
| Candidatus\_Symbiobacter | neg\_162 | 0.915151536464691 | 0.000204472206099204 |
| Cvm10virus | neg\_162 | 0.800390899181366 | 0.0054162086926719 |
| Rodentibacter | neg\_1649 | 0.830303013324738 | 0.00294022813612127 |
| Marivita | neg\_1649 | -0.854545474052429 | 0.00163680247839526 |
| Acetivibrio | neg\_1649 | -0.939393937587738 | 5.48405364009241e-05 |
| Dolosicoccus | neg\_1649 | -0.806060612201691 | 0.0048620605246823 |
| Mudcatvirus | neg\_1649 | 0.866666674613953 | 0.0011735379121256 |
| Litorimonas | neg\_1717 | -0.939393937587738 | 5.48405364009241e-05 |
| Zhihengliuella | neg\_1717 | 0.806060612201691 | 0.0048620605246823 |
| Rodentibacter | neg\_1717 | -0.818181812763214 | 0.00381492051076338 |
| Larkinella | neg\_1717 | -0.939393937587738 | 5.48405364009241e-05 |
| Limnobacter | neg\_1717 | -0.863225877285004 | 0.00129381457010247 |
| Porticoccus | neg\_1717 | -0.963636338710785 | 7.32099466027591e-06 |
| Rubrivivax | neg\_1718 | 0.906692802906036 | 0.000295911879524491 |
| Candidatus\_Sulfopaludibacter | neg\_1727 | -0.806060612201691 | 0.0048620605246823 |
| Thermopetrobacter | neg\_1727 | -0.818181812763214 | 0.00381492051076338 |
| Porticoccus | neg\_1727 | -0.854545474052429 | 0.00163680247839526 |
| Turicibacter | neg\_1737 | -0.806060612201691 | 0.0048620605246823 |
| Lawsonella | neg\_176 | -0.905779242515564 | 0.000307323103084567 |
| Halothiobacillus | neg\_176 | 0.854545474052429 | 0.00163680247839526 |
| Candidatus\_Kryptobacter | neg\_176 | 0.842424213886261 | 0.00222003275351312 |
| Succinimonas | neg\_176 | 0.806060612201691 | 0.0048620605246823 |
| Stappia | neg\_1781 | 0.878787875175476 | 0.000813862205061078 |
| Caloramator | neg\_1781 | 0.806060612201691 | 0.0048620605246823 |
| Candidatus\_Kryptonium | neg\_1781 | 0.806060612201691 | 0.0048620605246823 |
| Candidatus\_Tokpelaia | neg\_1781 | 0.806060612201691 | 0.0048620605246823 |
| Haliscomenobacter | neg\_1781 | 0.818181812763214 | 0.00381492051076338 |
| Lawsonella | neg\_1781 | 0.97872793674469 | 8.73146423829851e-07 |
| Palleronia | neg\_1781 | -0.844988703727722 | 0.00208579660703911 |
| Halothiobacillus | neg\_1781 | -0.818181812763214 | 0.00381492051076338 |
| Variovorax | neg\_1781 | 0.830303013324738 | 0.00294022813612127 |
| Succinimonas | neg\_1781 | -0.854545474052429 | 0.00163680247839526 |
| Beggiatoa | neg\_1791 | 0.927272737026215 | 0.000112034447641074 |
| Kriegella | neg\_1796 | 0.830303013324738 | 0.00294022813612127 |
| Candidatus\_Kryptonium | neg\_1796 | 0.806060612201691 | 0.0048620605246823 |
| Candidatus\_Moduliflexus | neg\_1796 | 0.866666674613953 | 0.0011735379121256 |
| Ottowia | neg\_1796 | -0.818181812763214 | 0.00381492051076338 |
| Gottschalkia | neg\_1796 | 0.915151536464691 | 0.000204472206099204 |
| Melissococcus | neg\_1796 | -0.866666674613953 | 0.0011735379121256 |
| Methanosalsum | neg\_1796 | -0.878787875175476 | 0.000813862205061078 |
| Thiodictyon | neg\_1796 | -0.890909075737 | 0.000542144516154419 |
| Metaprevotella | neg\_1796 | 0.818181812763214 | 0.00381492051076338 |
| Thermobifida | neg\_1798 | -0.854545474052429 | 0.00163680247839526 |
| Stappia | neg\_1798 | 0.927272737026215 | 0.000112034447641074 |
| Caloramator | neg\_1798 | 0.842424213886261 | 0.00222003275351312 |
| Candidatus\_Kryptonium | neg\_1798 | 0.806060612201691 | 0.0048620605246823 |
| Lawsonella | neg\_1798 | 0.905779242515564 | 0.000307323103084567 |
| Palleronia | neg\_1798 | -0.808514356613159 | 0.00463528246178302 |
| Anaerotignum | neg\_1804 | -0.83890962600708 | 0.00241398974898654 |
| Litorimonas | neg\_1804 | 0.83890962600708 | 0.00241398974898654 |
| Haliscomenobacter | neg\_1804 | 0.820672512054443 | 0.00362170350001234 |
| Zhihengliuella | neg\_1804 | -0.863225877285004 | 0.00129381457010247 |
| Lawsonella | neg\_1804 | 0.878048777580261 | 0.000833121234763867 |
| Rodentibacter | neg\_1804 | 0.83890962600708 | 0.00241398974898654 |
| Harryflintia | neg\_1804 | 0.820672512054443 | 0.00362170350001234 |
| Izhakiella | neg\_1804 | 0.878048777580261 | 0.000833121234763867 |
| Desulfocucumis | neg\_1804 | -0.83890962600708 | 0.00241398974898654 |
| Succinimonas | neg\_1804 | -0.869304955005646 | 0.00108698415655106 |
| Brevinema | neg\_1852 | 0.806643962860107 | 0.00480744414521128 |
| Candidatus\_Tokpelaia | neg\_1852 | 0.831656157970428 | 0.00285249465359505 |
| Lawsonella | neg\_1852 | 0.805963456630707 | 0.00487119959616233 |
| Beggiatoa | neg\_1852 | 0.906692802906036 | 0.000295911879524491 |
| Thermobifida | neg\_1863 | -0.854545474052429 | 0.00163680247839526 |
| Brevinema | neg\_1863 | 0.818181812763214 | 0.00381492051076338 |
| Mycolicibacter | neg\_1863 | -0.806060612201691 | 0.0048620605246823 |
| Mongoliibacter | neg\_1863 | 0.854545474052429 | 0.00163680247839526 |
| Turicibacter | neg\_1863 | 0.890909075737 | 0.000542144516154419 |
| Candidatus\_Symbiobacter | neg\_1863 | 0.830303013324738 | 0.00294022813612127 |
| Beggiatoa | neg\_1863 | 0.854545474052429 | 0.00163680247839526 |
| Mongoliibacter | neg\_1874 | -0.830303013324738 | 0.00294022813612127 |
| Rubrivivax | neg\_1874 | 0.806060612201691 | 0.0048620605246823 |
| Ornithobacterium | neg\_1884 | -0.806060612201691 | 0.0048620605246823 |
| Catenovulum | neg\_1886 | -0.854545474052429 | 0.00163680247839526 |
| Kriegella | neg\_1886 | 0.842424213886261 | 0.00222003275351312 |
| Propionicimonas | neg\_1886 | 0.802435338497162 | 0.00521145970824932 |
| Thermoanaerobacter | neg\_1886 | 0.890909075737 | 0.000542144516154419 |
| Acidaminobacter | neg\_1886 | 0.842424213886261 | 0.00222003275351312 |
| Trichormus | neg\_1886 | -0.806060612201691 | 0.0048620605246823 |
| Variovorax | neg\_1886 | 0.951515138149261 | 2.27985739738035e-05 |
| Anaerobacterium | neg\_1901 | 0.842424213886261 | 0.00222003275351312 |
| Natribacillus | neg\_1901 | 0.854545474052429 | 0.00163680247839526 |
| Caldicellulosiruptor | neg\_1901 | 0.806060612201691 | 0.0048620605246823 |
| Dethiosulfovibrio | neg\_193 | -0.915151536464691 | 0.000204472206099204 |
| Candidatus\_Sulfotelmatomonas | neg\_193 | 0.830303013324738 | 0.00294022813612127 |
| Amantichitinum | neg\_193 | 0.878787875175476 | 0.000813862205061078 |
| Lawsonella | neg\_1993 | 0.851067781448364 | 0.00179112909994994 |
| Halothiobacillus | neg\_1993 | -0.818181812763214 | 0.00381492051076338 |
| Anaerotignum | neg\_1996 | -0.830303013324738 | 0.00294022813612127 |
| Litorimonas | neg\_1996 | 0.806060612201691 | 0.0048620605246823 |
| Haliscomenobacter | neg\_1996 | 0.830303013324738 | 0.00294022813612127 |
| Zhihengliuella | neg\_1996 | -0.854545474052429 | 0.00163680247839526 |
| Lawsonella | neg\_1996 | 0.899700224399567 | 0.000391673296721073 |
| Rodentibacter | neg\_1996 | 0.818181812763214 | 0.00381492051076338 |
| Izhakiella | neg\_1996 | 0.899700224399567 | 0.000391673296721073 |
| Desulfocucumis | neg\_1996 | -0.806060612201691 | 0.0048620605246823 |
| Succinimonas | neg\_1996 | -0.878787875175476 | 0.000813862205061078 |
| Erythrobacter | neg\_1998 | -0.806060612201691 | 0.0048620605246823 |
| Pakpunavirus | neg\_1998 | -0.826751530170441 | 0.00317971472634282 |
| Thermosyntropha | neg\_1998 | -0.890909075737 | 0.000542144516154419 |
| Comamonas | neg\_1998 | 0.866666674613953 | 0.0011735379121256 |
| Limestonevirus | neg\_1998 | -0.80374151468277 | 0.00508357521208702 |
| Altibacter | neg\_1998 | -0.830303013324738 | 0.00294022813612127 |
| Marmoricola | neg\_2032 | 0.963636338710785 | 7.32099466027591e-06 |
| Erythrobacter | neg\_2038 | -0.806060612201691 | 0.0048620605246823 |
| Chloroflexus | neg\_2038 | -0.939393937587738 | 5.48405364009241e-05 |
| Pakpunavirus | neg\_2038 | -0.936174511909485 | 6.71888372667517e-05 |
| Candidatus\_Sulfopaludibacter | neg\_2038 | -0.806060612201691 | 0.0048620605246823 |
| Thermosyntropha | neg\_2038 | -0.903030276298523 | 0.000343612565232743 |
| Methanosphaerula | neg\_2038 | 0.806060612201691 | 0.0048620605246823 |
| Fimbriimonas | neg\_2038 | -0.866666674613953 | 0.0011735379121256 |
| Comamonas | neg\_2038 | 0.806060612201691 | 0.0048620605246823 |
| Parafilimonas | neg\_2038 | 0.903030276298523 | 0.000343612565232743 |
| Natronolimnobius | neg\_2038 | -0.806060612201691 | 0.0048620605246823 |
| Candidatus\_Halobonum | neg\_2038 | -0.924016416072845 | 0.000132952728702396 |
| Ktedonobacter | neg\_2038 | -0.806060612201691 | 0.0048620605246823 |
| Anaerotignum | neg\_2056 | -0.852824926376343 | 0.00171189540742711 |
| Litorimonas | neg\_2056 | 0.828283190727234 | 0.00307477558489877 |
| Parapedobacter | neg\_2056 | 0.80374151468277 | 0.00508357521208702 |
| Haliscomenobacter | neg\_2056 | 0.80374151468277 | 0.00508357521208702 |
| Zhihengliuella | neg\_2056 | -0.816012322902679 | 0.0039891464803441 |
| Lawsonella | neg\_2056 | 0.892345726490021 | 0.000515068773110805 |
| Rodentibacter | neg\_2056 | 0.80374151468277 | 0.00508357521208702 |
| Palleronia | neg\_2056 | -0.849266946315765 | 0.00187505312445646 |
| Larkinella | neg\_2056 | 0.840554058551788 | 0.00232177976116144 |
| Limnobacter | neg\_2056 | 0.880037486553192 | 0.000782041934040523 |
| Izhakiella | neg\_2056 | 0.867729306221008 | 0.00113809565686207 |
| Succinimonas | neg\_2056 | -0.840554058551788 | 0.00232177976116144 |
| Stappia | neg\_2068 | 0.854545474052429 | 0.00163680247839526 |
| Caloramator | neg\_2068 | 0.915151536464691 | 0.000204472206099204 |
| Candidatus\_Tokpelaia | neg\_2068 | 0.878787875175476 | 0.000813862205061078 |
| Lawsonella | neg\_2068 | 0.857146799564362 | 0.0015278405327328 |
| Kordiimonas | neg\_2068 | 0.866666674613953 | 0.0011735379121256 |
| Anaerobacterium | neg\_2068 | 0.806060612201691 | 0.0048620605246823 |
| Candidatus\_Electrothrix | neg\_2068 | 0.854545474052429 | 0.00163680247839526 |
| Pacificibacter | neg\_2068 | 0.818181812763214 | 0.00381492051076338 |
| Lawsonella | neg\_207 | 0.826751530170441 | 0.00317971472634282 |
| Halothiobacillus | neg\_207 | -0.806060612201691 | 0.0048620605246823 |
| Kriegella | neg\_2109 | 0.842424213886261 | 0.00222003275351312 |
| Candidatus\_Kryptonium | neg\_2109 | 0.866666674613953 | 0.0011735379121256 |
| Parapedobacter | neg\_2109 | 0.806060612201691 | 0.0048620605246823 |
| Ottowia | neg\_2109 | -0.842424213886261 | 0.00222003275351312 |
| Propionicimonas | neg\_2109 | 0.857146799564362 | 0.0015278405327328 |
| Palleronia | neg\_2109 | -0.826751530170441 | 0.00317971472634282 |
| Sinirhodobacter | neg\_2109 | -0.903030276298523 | 0.000343612565232743 |
| Microscilla | neg\_2109 | 0.818181812763214 | 0.00381492051076338 |
| Melghirimyces | neg\_2109 | 0.806060612201691 | 0.0048620605246823 |
| Marmoricola | neg\_2109 | -0.806060612201691 | 0.0048620605246823 |
| Novimethylophilus | neg\_2109 | 0.854545474052429 | 0.00163680247839526 |
| Jiangella | neg\_2109 | 0.890909075737 | 0.000542144516154419 |
| Melissococcus | neg\_2109 | -0.854545474052429 | 0.00163680247839526 |
| Acidaminobacter | neg\_2109 | 0.854545474052429 | 0.00163680247839526 |
| Sideroxydans | neg\_2109 | -0.890909075737 | 0.000542144516154419 |
| Mailhella | neg\_2109 | -0.975757598876953 | 1.46754063035104e-06 |
| Flexilinea | neg\_2109 | -0.854545474052429 | 0.00163680247839526 |
| Caloramator | neg\_2116 | 0.866666674613953 | 0.0011735379121256 |
| Catenovulum | neg\_2116 | -0.963636338710785 | 7.32099466027591e-06 |
| Kriegella | neg\_2116 | 0.878787875175476 | 0.000813862205061078 |
| Propionicimonas | neg\_2116 | 0.851067781448364 | 0.00179112909994994 |
| Lawsonella | neg\_2116 | 0.863225877285004 | 0.00129381457010247 |
| Thermoanaerobacter | neg\_2116 | 0.878787875175476 | 0.000813862205061078 |
| Anaerobacterium | neg\_2116 | 0.806060612201691 | 0.0048620605246823 |
| Trichormus | neg\_2116 | -0.866666674613953 | 0.0011735379121256 |
| Variovorax | neg\_2116 | 0.915151536464691 | 0.000204472206099204 |
| Caldicellulosiruptor | neg\_2116 | 0.806060612201691 | 0.0048620605246823 |
| Eah2virus | neg\_2116 | 0.81251859664917 | 0.00428159166664499 |
| Thermobifida | neg\_2134 | -0.866666674613953 | 0.0011735379121256 |
| Stappia | neg\_2134 | 0.818181812763214 | 0.00381492051076338 |
| Erythrobacter | neg\_2134 | 0.806060612201691 | 0.0048620605246823 |
| Pakpunavirus | neg\_2134 | 0.802435338497162 | 0.00521145970824932 |
| Lihuaxuella | neg\_2134 | 0.854545474052429 | 0.00163680247839526 |
| Acetivibrio | neg\_2134 | -0.818181812763214 | 0.00381492051076338 |
| Fimbriimonas | neg\_2134 | 0.842424213886261 | 0.00222003275351312 |
| Izhakiella | neg\_2134 | 0.814593434333801 | 0.00410613103846336 |
| Desulfocucumis | neg\_2134 | -0.866666674613953 | 0.0011735379121256 |
| Promicromonospora | neg\_2134 | 0.878787875175476 | 0.000813862205061078 |
| Litorimonas | neg\_2157 | 0.818181812763214 | 0.00381492051076338 |
| Zhihengliuella | neg\_2157 | -0.842424213886261 | 0.00222003275351312 |
| Rodentibacter | neg\_2157 | 0.818181812763214 | 0.00381492051076338 |
| Acetivibrio | neg\_2157 | -0.818181812763214 | 0.00381492051076338 |
| Izhakiella | neg\_2157 | 0.808514356613159 | 0.00463528246178302 |
| Desulfocucumis | neg\_2157 | -0.830303013324738 | 0.00294022813612127 |
| Thermobifida | neg\_2164 | -0.818181812763214 | 0.00381492051076338 |
| Stappia | neg\_2164 | 0.878787875175476 | 0.000813862205061078 |
| Caloramator | neg\_2164 | 0.806060612201691 | 0.0048620605246823 |
| Catenovulum | neg\_2164 | -0.818181812763214 | 0.00381492051076338 |
| Lawsonella | neg\_2164 | 0.936174511909485 | 6.71888372667517e-05 |
| Dethiosulfovibrio | neg\_2164 | -0.806060612201691 | 0.0048620605246823 |
| Variovorax | neg\_2164 | 0.830303013324738 | 0.00294022813612127 |
| Thermobifida | neg\_2197 | -0.865095794200897 | 0.00122739260821847 |
| Stappia | neg\_2197 | 0.865095794200897 | 0.00122739260821847 |
| Caloramator | neg\_2197 | 0.80374151468277 | 0.00508357521208702 |
| Anaerotignum | neg\_2197 | -0.828283190727234 | 0.00307477558489877 |
| Litorimonas | neg\_2197 | 0.828283190727234 | 0.00307477558489877 |
| Haliscomenobacter | neg\_2197 | 0.852824926376343 | 0.00171189540742711 |
| Zhihengliuella | neg\_2197 | -0.80374151468277 | 0.00508357521208702 |
| Lawsonella | neg\_2197 | 0.904653906822205 | 0.000321819947074919 |
| Harryflintia | neg\_2197 | 0.852824926376343 | 0.00171189540742711 |
| Desulfocucumis | neg\_2197 | -0.840554058551788 | 0.00232177976116144 |
| Succinimonas | neg\_2197 | -0.877366662025452 | 0.000851188393096614 |
| Candidatus\_Tokpelaia | neg\_2251 | 0.927272737026215 | 0.000112034447641074 |
| Kordiimonas | neg\_2251 | 0.939393937587738 | 5.48405364009241e-05 |
| Pararhodospirillum | neg\_2251 | 0.818181812763214 | 0.00381492051076338 |
| Candidatus\_Electrothrix | neg\_2251 | 0.854545474052429 | 0.00163680247839526 |
| Parafilimonas | neg\_2251 | -0.854545474052429 | 0.00163680247839526 |
| Thermobifida | neg\_2257 | -0.820672512054443 | 0.00362170350001234 |
| Stappia | neg\_2257 | 0.826751530170441 | 0.00317971472634282 |
| Anaerotignum | neg\_2257 | -0.826751530170441 | 0.00317971472634282 |
| Haliscomenobacter | neg\_2257 | 0.814593434333801 | 0.00410613103846336 |
| Zhihengliuella | neg\_2257 | -0.851067781448364 | 0.00179112909994994 |
| Lihuaxuella | neg\_2257 | 0.808514356613159 | 0.00463528246178302 |
| Lawsonella | neg\_2257 | 0.826219499111176 | 0.00321676075800825 |
| Rodentibacter | neg\_2257 | 0.826751530170441 | 0.00317971472634282 |
| Marivita | neg\_2257 | -0.820672512054443 | 0.00362170350001234 |
| Acetivibrio | neg\_2257 | -0.802435338497162 | 0.00521145970824932 |
| Izhakiella | neg\_2257 | 0.83841460943222 | 0.00244225698888578 |
| Desulfocucumis | neg\_2257 | -0.863225877285004 | 0.00129381457010247 |
| Succinimonas | neg\_2257 | -0.826751530170441 | 0.00317971472634282 |
| Haliscomenobacter | neg\_2264 | -0.915151536464691 | 0.000204472206099204 |
| Zhihengliuella | neg\_2264 | 0.842424213886261 | 0.00222003275351312 |
| Lawsonella | neg\_2264 | -0.881463050842285 | 0.000746860533753502 |
| Halothiobacillus | neg\_2264 | 0.842424213886261 | 0.00222003275351312 |
| Izhakiella | neg\_2264 | -0.808514356613159 | 0.00463528246178302 |
| Succinimonas | neg\_2264 | 0.939393937587738 | 5.48405364009241e-05 |
| Catenovulum | neg\_2267 | -0.866666674613953 | 0.0011735379121256 |
| Kriegella | neg\_2267 | 0.854545474052429 | 0.00163680247839526 |
| Thermoanaerobacter | neg\_2267 | 0.890909075737 | 0.000542144516154419 |
| Variovorax | neg\_2267 | 0.915151536464691 | 0.000204472206099204 |
| Anaerotignum | neg\_2280 | -0.83890962600708 | 0.00241398974898654 |
| Litorimonas | neg\_2280 | 0.851067781448364 | 0.00179112909994994 |
| Haliscomenobacter | neg\_2280 | 0.820672512054443 | 0.00362170350001234 |
| Zhihengliuella | neg\_2280 | -0.863225877285004 | 0.00129381457010247 |
| Lawsonella | neg\_2280 | 0.853658556938171 | 0.00167520705151691 |
| Rodentibacter | neg\_2280 | 0.83890962600708 | 0.00241398974898654 |
| Larkinella | neg\_2280 | 0.820672512054443 | 0.00362170350001234 |
| Izhakiella | neg\_2280 | 0.914634168148041 | 0.00020937092948925 |
| Desulfocucumis | neg\_2280 | -0.814593434333801 | 0.00410613103846336 |
| Porticoccus | neg\_2280 | 0.826751530170441 | 0.00317971472634282 |
| Succinimonas | neg\_2280 | -0.869304955005646 | 0.00108698415655106 |
| Candidatus\_Arcanobacter | neg\_2280 | 0.814593434333801 | 0.00410613103846336 |
| Zhihengliuella | neg\_2282 | -0.842424213886261 | 0.00222003275351312 |
| Lawsonella | neg\_2282 | 0.802435338497162 | 0.00521145970824932 |
| Izhakiella | neg\_2282 | 0.802435338497162 | 0.00521145970824932 |
| Sulfobacillus | neg\_2298 | 0.806060612201691 | 0.0048620605246823 |
| Candidatus\_Sulfopaludibacter | neg\_2310 | -0.806060612201691 | 0.0048620605246823 |
| Kordiimonas | neg\_2310 | -0.806060612201691 | 0.0048620605246823 |
| Pararhodospirillum | neg\_2310 | -0.878787875175476 | 0.000813862205061078 |
| Candidatus\_Electrothrix | neg\_2310 | -0.806060612201691 | 0.0048620605246823 |
| Thermopetrobacter | neg\_2310 | -0.854545474052429 | 0.00163680247839526 |
| Litorimonas | neg\_2373 | 0.818181812763214 | 0.00381492051076338 |
| Zhihengliuella | neg\_2373 | -0.915151536464691 | 0.000204472206099204 |
| Lawsonella | neg\_2373 | 0.851067781448364 | 0.00179112909994994 |
| Rodentibacter | neg\_2373 | 0.890909075737 | 0.000542144516154419 |
| Izhakiella | neg\_2373 | 0.851067781448364 | 0.00179112909994994 |
| Brevinema | neg\_2383 | -0.806060612201691 | 0.0048620605246823 |
| Acetivibrio | neg\_2383 | 0.806060612201691 | 0.0048620605246823 |
| Turicibacter | neg\_2383 | -0.915151536464691 | 0.000204472206099204 |
| Catenovulum | neg\_2400 | -0.806060612201691 | 0.0048620605246823 |
| Kriegella | neg\_2400 | 0.818181812763214 | 0.00381492051076338 |
| Brevinema | neg\_2400 | 0.842424213886261 | 0.00222003275351312 |
| Limnobacter | neg\_2400 | 0.826751530170441 | 0.00317971472634282 |
| Thermoanaerobacter | neg\_2400 | 0.878787875175476 | 0.000813862205061078 |
| Lmd1virus | neg\_2400 | -0.806643962860107 | 0.00480744414521128 |
| Sideroxydans | neg\_2400 | -0.806060612201691 | 0.0048620605246823 |
| Anaerotignum | neg\_2415 | -0.826751530170441 | 0.00317971472634282 |
| Litorimonas | neg\_2415 | 0.826751530170441 | 0.00317971472634282 |
| Haliscomenobacter | neg\_2415 | 0.844988703727722 | 0.00208579660703911 |
| Zhihengliuella | neg\_2415 | -0.875383973121643 | 0.00090532610126548 |
| Lawsonella | neg\_2415 | 0.902438998222351 | 0.00035181140785312 |
| Rodentibacter | neg\_2415 | 0.826751530170441 | 0.00317971472634282 |
| Izhakiella | neg\_2415 | 0.902438998222351 | 0.00035181140785312 |
| Succinimonas | neg\_2415 | -0.893621146678925 | 0.000491873064334847 |
| Litorimonas | neg\_2446 | 0.878787875175476 | 0.000813862205061078 |
| Brevinema | neg\_2446 | 0.806060612201691 | 0.0048620605246823 |
| Haliscomenobacter | neg\_2446 | 0.842424213886261 | 0.00222003275351312 |
| Zhihengliuella | neg\_2446 | -0.890909075737 | 0.000542144516154419 |
| Lawsonella | neg\_2446 | 0.832830607891083 | 0.00277788845123905 |
| Rodentibacter | neg\_2446 | 0.830303013324738 | 0.00294022813612127 |
| Harryflintia | neg\_2446 | 0.842424213886261 | 0.00222003275351312 |
| Izhakiella | neg\_2446 | 0.832830607891083 | 0.00277788845123905 |
| Desulfocucumis | neg\_2446 | -0.806060612201691 | 0.0048620605246823 |
| Porticoccus | neg\_2446 | 0.830303013324738 | 0.00294022813612127 |
| Succinimonas | neg\_2446 | -0.866666674613953 | 0.0011735379121256 |
| Thermobifida | neg\_2456 | -0.806643962860107 | 0.00480744414521128 |
| Turicibacter | neg\_2456 | 0.862921476364136 | 0.00130486922968087 |
| Litorimonas | neg\_2461 | 0.830303013324738 | 0.00294022813612127 |
| Zhihengliuella | neg\_2461 | -0.927272737026215 | 0.000112034447641074 |
| Lawsonella | neg\_2461 | 0.83890962600708 | 0.00241398974898654 |
| Rodentibacter | neg\_2461 | 0.878787875175476 | 0.000813862205061078 |
| Izhakiella | neg\_2461 | 0.875383973121643 | 0.00090532610126548 |
| Porticoccus | neg\_2461 | 0.806060612201691 | 0.0048620605246823 |
| Succinimonas | neg\_2461 | -0.842424213886261 | 0.00222003275351312 |
| Anaerotignum | neg\_2462 | -0.878787875175476 | 0.000813862205061078 |
| Erythrobacter | neg\_2462 | 0.830303013324738 | 0.00294022813612127 |
| Litorimonas | neg\_2462 | 0.854545474052429 | 0.00163680247839526 |
| Zhihengliuella | neg\_2462 | -0.830303013324738 | 0.00294022813612127 |
| Lawsonella | neg\_2462 | 0.887542068958282 | 0.000609666476501403 |
| Rodentibacter | neg\_2462 | 0.854545474052429 | 0.00163680247839526 |
| Palleronia | neg\_2462 | -0.814593434333801 | 0.00410613103846336 |
| Larkinella | neg\_2462 | 0.830303013324738 | 0.00294022813612127 |
| Limnobacter | neg\_2462 | 0.814593434333801 | 0.00410613103846336 |
| Trichormus | neg\_2462 | -0.818181812763214 | 0.00381492051076338 |
| Izhakiella | neg\_2462 | 0.887542068958282 | 0.000609666476501403 |
| Desulfocucumis | neg\_2462 | -0.854545474052429 | 0.00163680247839526 |
| Succinimonas | neg\_2462 | -0.842424213886261 | 0.00222003275351312 |
| Catenovulum | neg\_2485 | -0.878787875175476 | 0.000813862205061078 |
| Lawsonella | neg\_2485 | 0.851067781448364 | 0.00179112909994994 |
| Anaerobacterium | neg\_2485 | 0.806060612201691 | 0.0048620605246823 |
| Variovorax | neg\_2485 | 0.878787875175476 | 0.000813862205061078 |
| Candidatus\_Kryptobacter | neg\_2485 | -0.890909075737 | 0.000542144516154419 |
| Stappia | neg\_2505 | 0.854545474052429 | 0.00163680247839526 |
| Caloramator | neg\_2505 | 0.903030276298523 | 0.000343612565232743 |
| Anaerotignum | neg\_2505 | -0.806060612201691 | 0.0048620605246823 |
| Candidatus\_Tokpelaia | neg\_2505 | 0.830303013324738 | 0.00294022813612127 |
| Lawsonella | neg\_2505 | 0.948332667350769 | 2.92858403718732e-05 |
| Rodentibacter | neg\_2505 | 0.830303013324738 | 0.00294022813612127 |
| Marivita | neg\_2505 | -0.818181812763214 | 0.00381492051076338 |
| Halothiobacillus | neg\_2505 | -0.818181812763214 | 0.00381492051076338 |
| Trichormus | neg\_2505 | -0.818181812763214 | 0.00381492051076338 |
| Succinimonas | neg\_2505 | -0.806060612201691 | 0.0048620605246823 |
| Catenovulum | neg\_2564 | -0.830303013324738 | 0.00294022813612127 |
| Lawsonella | neg\_2564 | 0.83890962600708 | 0.00241398974898654 |
| Variovorax | neg\_2564 | 0.927272737026215 | 0.000112034447641074 |
| Litorimonas | neg\_2570 | 0.866666674613953 | 0.0011735379121256 |
| Zhihengliuella | neg\_2570 | -0.842424213886261 | 0.00222003275351312 |
| Rodentibacter | neg\_2570 | 0.818181812763214 | 0.00381492051076338 |
| Acetivibrio | neg\_2570 | -0.854545474052429 | 0.00163680247839526 |
| Desulfocucumis | neg\_2570 | -0.842424213886261 | 0.00222003275351312 |
| Porticoccus | neg\_2570 | 0.866666674613953 | 0.0011735379121256 |
| Erythrobacter | neg\_2627 | -0.854545474052429 | 0.00163680247839526 |
| Candidatus\_Moduliflexus | neg\_2627 | -0.878787875175476 | 0.000813862205061078 |
| Pakpunavirus | neg\_2627 | -0.887542068958282 | 0.000609666476501403 |
| Ottowia | neg\_2627 | 0.939393937587738 | 5.48405364009241e-05 |
| Propionicimonas | neg\_2627 | -0.802435338497162 | 0.00521145970824932 |
| Candidatus\_Sulfopaludibacter | neg\_2627 | -0.927272737026215 | 0.000112034447641074 |
| Palleronia | neg\_2627 | 0.802435338497162 | 0.00521145970824932 |
| Alkalibacterium | neg\_2627 | -0.806060612201691 | 0.0048620605246823 |
| Microscilla | neg\_2627 | -0.830303013324738 | 0.00294022813612127 |
| Novimethylophilus | neg\_2627 | -0.830303013324738 | 0.00294022813612127 |
| Gottschalkia | neg\_2627 | -0.830303013324738 | 0.00294022813612127 |
| Melissococcus | neg\_2627 | 0.903030276298523 | 0.000343612565232743 |
| Fimbriimonas | neg\_2627 | -0.830303013324738 | 0.00294022813612127 |
| Candidatus\_Electrothrix | neg\_2627 | -0.806060612201691 | 0.0048620605246823 |
| Methanosalsum | neg\_2627 | 0.854545474052429 | 0.00163680247839526 |
| Thiodictyon | neg\_2627 | 0.890909075737 | 0.000542144516154419 |
| Candidatus\_Halobonum | neg\_2627 | -0.83890962600708 | 0.00241398974898654 |
| Metaprevotella | neg\_2627 | -0.878787875175476 | 0.000813862205061078 |
| Anaerotignum | neg\_2643 | -0.939393937587738 | 5.48405364009241e-05 |
| Erythrobacter | neg\_2643 | 0.830303013324738 | 0.00294022813612127 |
| Candidatus\_Kryptonium | neg\_2643 | 0.818181812763214 | 0.00381492051076338 |
| Litorimonas | neg\_2643 | 0.806060612201691 | 0.0048620605246823 |
| Parapedobacter | neg\_2643 | 0.842424213886261 | 0.00222003275351312 |
| Ottowia | neg\_2643 | -0.806060612201691 | 0.0048620605246823 |
| Zhihengliuella | neg\_2643 | -0.866666674613953 | 0.0011735379121256 |
| Lihuaxuella | neg\_2643 | 0.842424213886261 | 0.00222003275351312 |
| Lawsonella | neg\_2643 | 0.863225877285004 | 0.00129381457010247 |
| Rodentibacter | neg\_2643 | 0.915151536464691 | 0.000204472206099204 |
| Palleronia | neg\_2643 | -0.899700224399567 | 0.000391673296721073 |
| Microscilla | neg\_2643 | 0.830303013324738 | 0.00294022813612127 |
| Limnobacter | neg\_2643 | 0.802435338497162 | 0.00521145970824932 |
| Melghirimyces | neg\_2643 | 0.854545474052429 | 0.00163680247839526 |
| Izhakiella | neg\_2643 | 0.948332667350769 | 2.92858403718732e-05 |
| Desulfocucumis | neg\_2643 | -0.854545474052429 | 0.00163680247839526 |
| Succinimonas | neg\_2643 | -0.842424213886261 | 0.00222003275351312 |
| Candidatus\_Arcanobacter | neg\_2643 | 0.854545474052429 | 0.00163680247839526 |
| Erythrobacter | neg\_2684 | 0.820672512054443 | 0.00362170350001234 |
| Litorimonas | neg\_2684 | 0.887542068958282 | 0.000609666476501403 |
| Chloroflexus | neg\_2684 | 0.814593434333801 | 0.00410613103846336 |
| Larkinella | neg\_2684 | 0.942253589630127 | 4.53587077142714e-05 |
| Thermosyntropha | neg\_2684 | 0.814593434333801 | 0.00410613103846336 |
| Trichormus | neg\_2684 | -0.814593434333801 | 0.00410613103846336 |
| Porticoccus | neg\_2684 | 0.863225877285004 | 0.00129381457010247 |
| Natronolimnobius | neg\_2684 | 0.820672512054443 | 0.00362170350001234 |
| Ktedonobacter | neg\_2684 | 0.899700224399567 | 0.000391673296721073 |
| Thermobifida | neg\_2738 | 0.878787875175476 | 0.000813862205061078 |
| Stappia | neg\_2738 | -0.878787875175476 | 0.000813862205061078 |
| Caloramator | neg\_2738 | -0.842424213886261 | 0.00222003275351312 |
| Anaerotignum | neg\_2738 | 0.818181812763214 | 0.00381492051076338 |
| Lawsonella | neg\_2738 | -0.83890962600708 | 0.00241398974898654 |
| Marivita | neg\_2738 | 0.818181812763214 | 0.00381492051076338 |
| Harryflintia | neg\_2738 | -0.866666674613953 | 0.0011735379121256 |
| Candidatus\_Sulfotelmatomonas | neg\_2738 | -0.854545474052429 | 0.00163680247839526 |
| Desulfocucumis | neg\_2738 | 0.890909075737 | 0.000542144516154419 |
| Turicibacter | neg\_2754 | 0.878787875175476 | 0.000813862205061078 |
| Natribacillus | neg\_2754 | 0.854545474052429 | 0.00163680247839526 |
| Rubrivivax | neg\_2788 | 0.830303013324738 | 0.00294022813612127 |
| Dethiosulfovibrio | neg\_2801 | 0.818181812763214 | 0.00381492051076338 |
| Amantichitinum | neg\_2801 | -0.951515138149261 | 2.27985739738035e-05 |
| Brevinema | neg\_2803 | 0.854545474052429 | 0.00163680247839526 |
| Mongoliibacter | neg\_2803 | 0.806060612201691 | 0.0048620605246823 |
| Limnobacter | neg\_2803 | 0.808514356613159 | 0.00463528246178302 |
| Thermoanaerobacter | neg\_2803 | 0.842424213886261 | 0.00222003275351312 |
| Lmd1virus | neg\_2803 | -0.831656157970428 | 0.00285249465359505 |
| Eah2virus | neg\_2803 | 0.805690705776215 | 0.00489692183645341 |
| Stappia | neg\_2833 | 0.842424213886261 | 0.00222003275351312 |
| Lawsonella | neg\_2833 | 0.936174511909485 | 6.71888372667517e-05 |
| Variovorax | neg\_2833 | 0.866666674613953 | 0.0011735379121256 |
| Stappia | neg\_2842 | 0.830303013324738 | 0.00294022813612127 |
| Caloramator | neg\_2842 | 0.866666674613953 | 0.0011735379121256 |
| Catenovulum | neg\_2842 | -0.830303013324738 | 0.00294022813612127 |
| Anaerotignum | neg\_2842 | -0.806060612201691 | 0.0048620605246823 |
| Kriegella | neg\_2842 | 0.818181812763214 | 0.00381492051076338 |
| Candidatus\_Kryptonium | neg\_2842 | 0.842424213886261 | 0.00222003275351312 |
| Candidatus\_Tokpelaia | neg\_2842 | 0.830303013324738 | 0.00294022813612127 |
| Lawsonella | neg\_2842 | 0.948332667350769 | 2.92858403718732e-05 |
| Palleronia | neg\_2842 | -0.869304955005646 | 0.00108698415655106 |
| Sinirhodobacter | neg\_2842 | -0.806060612201691 | 0.0048620605246823 |
| Microscilla | neg\_2842 | 0.878787875175476 | 0.000813862205061078 |
| Halothiobacillus | neg\_2842 | -0.806060612201691 | 0.0048620605246823 |
| Variovorax | neg\_2842 | 0.890909075737 | 0.000542144516154419 |
| Cvm10virus | neg\_2842 | 0.831656157970428 | 0.00285249465359505 |
| Thermobifida | neg\_2851 | 0.830303013324738 | 0.00294022813612127 |
| Chloroflexus | neg\_2851 | -0.806060612201691 | 0.0048620605246823 |
| Emticicia | neg\_2851 | -0.818181812763214 | 0.00381492051076338 |
| Ornithobacterium | neg\_2851 | -0.890909075737 | 0.000542144516154419 |
| Comamonas | neg\_2851 | 0.806060612201691 | 0.0048620605246823 |
| Thermobifida | neg\_286 | -0.825403153896332 | 0.00327420560319291 |
| Litorimonas | neg\_286 | 0.881680607795715 | 0.000741594922435862 |
| Haliscomenobacter | neg\_286 | 0.856668412685394 | 0.00154747182074688 |
| Zhihengliuella | neg\_286 | -0.881680607795715 | 0.000741594922435862 |
| Lihuaxuella | neg\_286 | 0.806643962860107 | 0.00480744414521128 |
| Lawsonella | neg\_286 | 0.802827417850494 | 0.00517283391309364 |
| Rodentibacter | neg\_286 | 0.81915009021759 | 0.00373894966455346 |
| Acetivibrio | neg\_286 | -0.806643962860107 | 0.00480744414521128 |
| Izhakiella | neg\_286 | 0.865548312664032 | 0.00121169851367053 |
| Desulfocucumis | neg\_286 | -0.825403153896332 | 0.00327420560319291 |
| Porticoccus | neg\_286 | 0.856668412685394 | 0.00154747182074688 |
| Succinimonas | neg\_286 | -0.856668412685394 | 0.00154747182074688 |
| Candidatus\_Arcanobacter | neg\_286 | 0.800390899181366 | 0.0054162086926719 |
| Litorimonas | neg\_2886 | 0.878787875175476 | 0.000813862205061078 |
| Brevinema | neg\_2886 | 0.806060612201691 | 0.0048620605246823 |
| Haliscomenobacter | neg\_2886 | 0.842424213886261 | 0.00222003275351312 |
| Zhihengliuella | neg\_2886 | -0.890909075737 | 0.000542144516154419 |
| Lawsonella | neg\_2886 | 0.832830607891083 | 0.00277788845123905 |
| Rodentibacter | neg\_2886 | 0.830303013324738 | 0.00294022813612127 |
| Harryflintia | neg\_2886 | 0.842424213886261 | 0.00222003275351312 |
| Izhakiella | neg\_2886 | 0.832830607891083 | 0.00277788845123905 |
| Desulfocucumis | neg\_2886 | -0.806060612201691 | 0.0048620605246823 |
| Porticoccus | neg\_2886 | 0.830303013324738 | 0.00294022813612127 |
| Succinimonas | neg\_2886 | -0.866666674613953 | 0.0011735379121256 |
| Thermobifida | neg\_2892 | 0.830303013324738 | 0.00294022813612127 |
| Haliscomenobacter | neg\_2892 | -0.830303013324738 | 0.00294022813612127 |
| Litorimonas | neg\_2932 | 0.854545474052429 | 0.00163680247839526 |
| Haliscomenobacter | neg\_2932 | 0.806060612201691 | 0.0048620605246823 |
| Zhihengliuella | neg\_2932 | -0.915151536464691 | 0.000204472206099204 |
| Lawsonella | neg\_2932 | 0.851067781448364 | 0.00179112909994994 |
| Rodentibacter | neg\_2932 | 0.842424213886261 | 0.00222003275351312 |
| Izhakiella | neg\_2932 | 0.875383973121643 | 0.00090532610126548 |
| Porticoccus | neg\_2932 | 0.830303013324738 | 0.00294022813612127 |
| Succinimonas | neg\_2932 | -0.854545474052429 | 0.00163680247839526 |
| Limnobacter | neg\_2965 | 0.826219499111176 | 0.00321676075800825 |
| Thermoanaerobacter | neg\_2965 | 0.820672512054443 | 0.00362170350001234 |
| Eah2virus | neg\_2965 | 0.808143377304077 | 0.00466907341223743 |
| Rodentibacter | neg\_2972 | 0.830303013324738 | 0.00294022813612127 |
| Palleronia | neg\_2972 | -0.863225877285004 | 0.00129381457010247 |
| Leminorella | neg\_2972 | -0.842424213886261 | 0.00222003275351312 |
| Emticicia | neg\_2973 | -0.818181812763214 | 0.00381492051076338 |
| Thermopetrobacter | neg\_2973 | -0.818181812763214 | 0.00381492051076338 |
| Chloroflexus | neg\_302 | -0.854545474052429 | 0.00163680247839526 |
| Pakpunavirus | neg\_302 | -0.887542068958282 | 0.000609666476501403 |
| Thermosyntropha | neg\_302 | -0.915151536464691 | 0.000204472206099204 |
| Anaerobacterium | neg\_302 | -0.806060612201691 | 0.0048620605246823 |
| Fimbriimonas | neg\_302 | -0.878787875175476 | 0.000813862205061078 |
| Natronolimnobius | neg\_302 | -0.842424213886261 | 0.00222003275351312 |
| Candidatus\_Halobonum | neg\_302 | -0.924016416072845 | 0.000132952728702396 |
| Ktedonobacter | neg\_302 | -0.806060612201691 | 0.0048620605246823 |
| Thermobifida | neg\_3064 | -0.818181812763214 | 0.00381492051076338 |
| Litorimonas | neg\_3064 | 0.903030276298523 | 0.000343612565232743 |
| Brevinema | neg\_3064 | 0.830303013324738 | 0.00294022813612127 |
| Haliscomenobacter | neg\_3064 | 0.842424213886261 | 0.00222003275351312 |
| Zhihengliuella | neg\_3064 | -0.878787875175476 | 0.000813862205061078 |
| Lmd1virus | neg\_3064 | -0.81915009021759 | 0.00373894966455346 |
| Harryflintia | neg\_3064 | 0.806060612201691 | 0.0048620605246823 |
| Porticoccus | neg\_3064 | 0.878787875175476 | 0.000813862205061078 |
| Succinimonas | neg\_3064 | -0.818181812763214 | 0.00381492051076338 |
| Anaerotignum | neg\_3088 | -0.828283190727234 | 0.00307477558489877 |
| Erythrobacter | neg\_3088 | 0.80374151468277 | 0.00508357521208702 |
| Litorimonas | neg\_3088 | 0.877366662025452 | 0.000851188393096614 |
| Haliscomenobacter | neg\_3088 | 0.80374151468277 | 0.00508357521208702 |
| Zhihengliuella | neg\_3088 | -0.852824926376343 | 0.00171189540742711 |
| Rodentibacter | neg\_3088 | 0.840554058551788 | 0.00232177976116144 |
| Harryflintia | neg\_3088 | 0.852824926376343 | 0.00171189540742711 |
| Izhakiella | neg\_3088 | 0.85542106628418 | 0.00159951806374248 |
| Desulfocucumis | neg\_3088 | -0.877366662025452 | 0.000851188393096614 |
| Porticoccus | neg\_3088 | 0.852824926376343 | 0.00171189540742711 |
| Succinimonas | neg\_3088 | -0.828283190727234 | 0.00307477558489877 |
| Kordiimonas | neg\_3092 | 0.854545474052429 | 0.00163680247839526 |
| Pararhodospirillum | neg\_3092 | 0.951515138149261 | 2.27985739738035e-05 |
| Thermopetrobacter | neg\_3092 | 0.890909075737 | 0.000542144516154419 |
| Brevinema | neg\_3101 | -0.863225877285004 | 0.00129381457010247 |
| Mongoliibacter | neg\_3101 | -0.820672512054443 | 0.00362170350001234 |
| Lmd1virus | neg\_3101 | 0.865548312664032 | 0.00121169851367053 |
| Turicibacter | neg\_3101 | -0.826751530170441 | 0.00317971472634282 |
| Candidatus\_Symbiobacter | neg\_3101 | -0.814593434333801 | 0.00410613103846336 |
| Litorimonas | neg\_3198 | 0.842424213886261 | 0.00222003275351312 |
| Candidatus\_Tokpelaia | neg\_3198 | 0.830303013324738 | 0.00294022813612127 |
| Zhihengliuella | neg\_3198 | -0.927272737026215 | 0.000112034447641074 |
| Kordiimonas | neg\_3198 | 0.842424213886261 | 0.00222003275351312 |
| Lmd1virus | neg\_3198 | -0.806643962860107 | 0.00480744414521128 |
| Dolosicoccus | neg\_3198 | -0.806060612201691 | 0.0048620605246823 |
| Rubrivivax | neg\_3252 | 0.854545474052429 | 0.00163680247839526 |
| Mongoliibacter | neg\_3294 | -0.866666674613953 | 0.0011735379121256 |
| Suttonella | neg\_3294 | 0.830303013324738 | 0.00294022813612127 |
| Limnobacter | neg\_3294 | -0.893621146678925 | 0.000491873064334847 |
| Synergistes | neg\_3294 | 0.830303013324738 | 0.00294022813612127 |
| Candidatus\_Symbiobacter | neg\_3294 | -0.878787875175476 | 0.000813862205061078 |
| Anaerotignum | neg\_3351 | -0.806060612201691 | 0.0048620605246823 |
| Kriegella | neg\_3351 | 0.818181812763214 | 0.00381492051076338 |
| Lawsonella | neg\_3351 | 0.887542068958282 | 0.000609666476501403 |
| Palleronia | neg\_3351 | -0.851067781448364 | 0.00179112909994994 |
| Variovorax | neg\_3351 | 0.806060612201691 | 0.0048620605246823 |
| Leminorella | neg\_3351 | -0.830303013324738 | 0.00294022813612127 |
| Eah2virus | neg\_3351 | 0.833002269268036 | 0.00276710271221536 |
| Anaerotignum | neg\_3355 | -0.806060612201691 | 0.0048620605246823 |
| Dethiosulfovibrio | neg\_3355 | -0.903030276298523 | 0.000343612565232743 |
| Candidatus\_Sulfotelmatomonas | neg\_3355 | 0.915151536464691 | 0.000204472206099204 |
| Amantichitinum | neg\_3355 | 0.878787875175476 | 0.000813862205061078 |
| Thermobifida | neg\_3360 | -0.825403153896332 | 0.00327420560319291 |
| Anaerotignum | neg\_3360 | -0.869174480438232 | 0.00109115185763287 |
| Erythrobacter | neg\_3360 | 0.850415349006653 | 0.00182121334938934 |
| Litorimonas | neg\_3360 | 0.894186735153198 | 0.000481835628326488 |
| Brevinema | neg\_3360 | 0.850415349006653 | 0.00182121334938934 |
| Ottowia | neg\_3360 | -0.825403153896332 | 0.00327420560319291 |
| Haliscomenobacter | neg\_3360 | 0.831656157970428 | 0.00285249465359505 |
| Zhihengliuella | neg\_3360 | -0.881680607795715 | 0.000741594922435862 |
| Lihuaxuella | neg\_3360 | 0.81915009021759 | 0.00373894966455346 |
| Lawsonella | neg\_3360 | 0.802827417850494 | 0.00517283391309364 |
| Rodentibacter | neg\_3360 | 0.881680607795715 | 0.000741594922435862 |
| Candidatus\_Sulfopaludibacter | neg\_3360 | 0.806643962860107 | 0.00480744414521128 |
| Palleronia | neg\_3360 | -0.840459942817688 | 0.00232698748109961 |
| Larkinella | neg\_3360 | 0.800390899181366 | 0.0054162086926719 |
| Acetivibrio | neg\_3360 | -0.844162285327911 | 0.00212839548320387 |
| Limnobacter | neg\_3360 | 0.871820390224457 | 0.00100887727685151 |
| Dolosicoccus | neg\_3360 | -0.81915009021759 | 0.00373894966455346 |
| Izhakiella | neg\_3360 | 0.878092467784882 | 0.000831973628321769 |
| Desulfocucumis | neg\_3360 | -0.862921476364136 | 0.00130486922968087 |
| Porticoccus | neg\_3360 | 0.856668412685394 | 0.00154747182074688 |
| Succinimonas | neg\_3360 | -0.831656157970428 | 0.00285249465359505 |
| Candidatus\_Arcanobacter | neg\_3360 | 0.812897026538849 | 0.00424919679863534 |
| Synergistes | neg\_3362 | 0.878787875175476 | 0.000813862205061078 |
| Thermobifida | neg\_3377 | -0.806060612201691 | 0.0048620605246823 |
| Stappia | neg\_3377 | 0.854545474052429 | 0.00163680247839526 |
| Lawsonella | neg\_3377 | 0.844988703727722 | 0.00208579660703911 |
| Halothiobacillus | neg\_3377 | -0.842424213886261 | 0.00222003275351312 |
| Succinimonas | neg\_3377 | -0.818181812763214 | 0.00381492051076338 |
| Catenovulum | neg\_3414 | 0.890909075737 | 0.000542144516154419 |
| Lawsonella | neg\_3414 | -0.851067781448364 | 0.00179112909994994 |
| Dethiosulfovibrio | neg\_3414 | 0.866666674613953 | 0.0011735379121256 |
| Ornithobacterium | neg\_3414 | -0.878787875175476 | 0.000813862205061078 |
| Litorimonas | neg\_3422 | -0.854545474052429 | 0.00163680247839526 |
| Zhihengliuella | neg\_3422 | 0.890909075737 | 0.000542144516154419 |
| Rodentibacter | neg\_3422 | -0.818181812763214 | 0.00381492051076338 |
| Dolosicoccus | neg\_3422 | 0.866666674613953 | 0.0011735379121256 |
| Thermopetrobacter | neg\_3422 | -0.818181812763214 | 0.00381492051076338 |
| Porticoccus | neg\_3422 | -0.951515138149261 | 2.27985739738035e-05 |
| Anaerotignum | neg\_344 | -0.818181812763214 | 0.00381492051076338 |
| Brevinema | neg\_344 | 0.878787875175476 | 0.000813862205061078 |
| Parapedobacter | neg\_344 | 0.830303013324738 | 0.00294022813612127 |
| Ottowia | neg\_344 | -0.854545474052429 | 0.00163680247839526 |
| Haliscomenobacter | neg\_344 | 0.903030276298523 | 0.000343612565232743 |
| Zhihengliuella | neg\_344 | -0.890909075737 | 0.000542144516154419 |
| Lihuaxuella | neg\_344 | 0.842424213886261 | 0.00222003275351312 |
| Rodentibacter | neg\_344 | 0.830303013324738 | 0.00294022813612127 |
| Candidatus\_Sulfopaludibacter | neg\_344 | 0.818181812763214 | 0.00381492051076338 |
| Palleronia | neg\_344 | -0.899700224399567 | 0.000391673296721073 |
| Suttonella | neg\_344 | -0.903030276298523 | 0.000343612565232743 |
| Limnobacter | neg\_344 | 0.863225877285004 | 0.00129381457010247 |
| Melghirimyces | neg\_344 | 0.818181812763214 | 0.00381492051076338 |
| Dolosicoccus | neg\_344 | -0.866666674613953 | 0.0011735379121256 |
| Izhakiella | neg\_344 | 0.881463050842285 | 0.000746860533753502 |
| Thermopetrobacter | neg\_344 | 0.818181812763214 | 0.00381492051076338 |
| Succinimonas | neg\_344 | -0.878787875175476 | 0.000813862205061078 |
| Candidatus\_Arcanobacter | neg\_344 | 0.878787875175476 | 0.000813862205061078 |
| Thermoanaerobacter | neg\_3440 | 0.830303013324738 | 0.00294022813612127 |
| Eah2virus | neg\_3440 | 0.805690705776215 | 0.00489692183645341 |
| Pakpunavirus | neg\_3458 | 0.857146799564362 | 0.0015278405327328 |
| Ottowia | neg\_3458 | -0.890909075737 | 0.000542144516154419 |
| Lihuaxuella | neg\_3458 | 0.854545474052429 | 0.00163680247839526 |
| Candidatus\_Sulfopaludibacter | neg\_3458 | 0.878787875175476 | 0.000813862205061078 |
| Larkinella | neg\_3458 | 0.818181812763214 | 0.00381492051076338 |
| Microscilla | neg\_3458 | 0.842424213886261 | 0.00222003275351312 |
| Melghirimyces | neg\_3458 | 0.806060612201691 | 0.0048620605246823 |
| Novimethylophilus | neg\_3458 | 0.878787875175476 | 0.000813862205061078 |
| Thermopetrobacter | neg\_3458 | 0.818181812763214 | 0.00381492051076338 |
| Natronolimnobius | neg\_3458 | 0.830303013324738 | 0.00294022813612127 |
| Candidatus\_Arcanobacter | neg\_3458 | 0.842424213886261 | 0.00222003275351312 |
| Haliscomenobacter | neg\_3487 | -0.878787875175476 | 0.000813862205061078 |
| Pararhodospirillum | neg\_3487 | -0.878787875175476 | 0.000813862205061078 |
| Thermopetrobacter | neg\_3487 | -0.878787875175476 | 0.000813862205061078 |
| Succinimonas | neg\_3487 | 0.842424213886261 | 0.00222003275351312 |
| Thermobifida | neg\_3496 | -0.842424213886261 | 0.00222003275351312 |
| Stappia | neg\_3496 | 0.806060612201691 | 0.0048620605246823 |
| Litorimonas | neg\_3496 | 0.830303013324738 | 0.00294022813612127 |
| Haliscomenobacter | neg\_3496 | 0.806060612201691 | 0.0048620605246823 |
| Zhihengliuella | neg\_3496 | -0.830303013324738 | 0.00294022813612127 |
| Acetivibrio | neg\_3496 | -0.818181812763214 | 0.00381492051076338 |
| Harryflintia | neg\_3496 | 0.806060612201691 | 0.0048620605246823 |
| Desulfocucumis | neg\_3496 | -0.866666674613953 | 0.0011735379121256 |
| Succinimonas | neg\_3496 | -0.806060612201691 | 0.0048620605246823 |
| Erythrobacter | neg\_3498 | -0.842424213886261 | 0.00222003275351312 |
| Litorimonas | neg\_3498 | -0.842424213886261 | 0.00222003275351312 |
| Brevinema | neg\_3498 | -0.878787875175476 | 0.000813862205061078 |
| Marivita | neg\_3498 | 0.830303013324738 | 0.00294022813612127 |
| Limnobacter | neg\_3498 | -0.887542068958282 | 0.000609666476501403 |
| Lmd1virus | neg\_3498 | 0.869174480438232 | 0.00109115185763287 |
| Synergistes | neg\_3498 | 0.866666674613953 | 0.0011735379121256 |
| Comamonas | neg\_3498 | 0.842424213886261 | 0.00222003275351312 |
| Mudcatvirus | neg\_3498 | -0.806060612201691 | 0.0048620605246823 |
| Eah2virus | neg\_3498 | -0.846658051013947 | 0.00200163182665913 |
| Thermobifida | neg\_3504 | -0.80374151468277 | 0.00508357521208702 |
| Anaerotignum | neg\_3504 | -0.816012322902679 | 0.0039891464803441 |
| Litorimonas | neg\_3504 | 0.852824926376343 | 0.00171189540742711 |
| Haliscomenobacter | neg\_3504 | 0.828283190727234 | 0.00307477558489877 |
| Zhihengliuella | neg\_3504 | -0.865095794200897 | 0.00122739260821847 |
| Lawsonella | neg\_3504 | 0.843112826347351 | 0.00218339276204738 |
| Rodentibacter | neg\_3504 | 0.828283190727234 | 0.00307477558489877 |
| Harryflintia | neg\_3504 | 0.828283190727234 | 0.00307477558489877 |
| Izhakiella | neg\_3504 | 0.843112826347351 | 0.00218339276204738 |
| Desulfocucumis | neg\_3504 | -0.852824926376343 | 0.00171189540742711 |
| Porticoccus | neg\_3504 | 0.80374151468277 | 0.00508357521208702 |
| Succinimonas | neg\_3504 | -0.852824926376343 | 0.00171189540742711 |
| Brevinema | neg\_3529 | -0.830303013324738 | 0.00294022813612127 |
| Zhihengliuella | neg\_3529 | 0.866666674613953 | 0.0011735379121256 |
| Lihuaxuella | neg\_3529 | -0.806060612201691 | 0.0048620605246823 |
| Rodentibacter | neg\_3529 | -0.842424213886261 | 0.00222003275351312 |
| Candidatus\_Sulfopaludibacter | neg\_3529 | -0.854545474052429 | 0.00163680247839526 |
| Pararhodospirillum | neg\_3529 | -0.818181812763214 | 0.00381492051076338 |
| Dolosicoccus | neg\_3529 | 0.927272737026215 | 0.000112034447641074 |
| Candidatus\_Electrothrix | neg\_3529 | -0.818181812763214 | 0.00381492051076338 |
| Thermopetrobacter | neg\_3529 | -0.915151536464691 | 0.000204472206099204 |
| Porticoccus | neg\_3529 | -0.806060612201691 | 0.0048620605246823 |
| Synergistes | neg\_3564 | 0.854545474052429 | 0.00163680247839526 |
| Thermoanaerobacter | neg\_3566 | -0.878787875175476 | 0.000813862205061078 |
| Trichormus | neg\_3566 | 0.818181812763214 | 0.00381492051076338 |
| Variovorax | neg\_3566 | -0.903030276298523 | 0.000343612565232743 |
| Thermoanaerobacter | neg\_3586 | 0.806060612201691 | 0.0048620605246823 |
| Sinirhodobacter | neg\_3587 | 0.806060612201691 | 0.0048620605246823 |
| Flexilinea | neg\_3587 | 0.842424213886261 | 0.00222003275351312 |
| Anaerotignum | neg\_3592 | -0.878787875175476 | 0.000813862205061078 |
| Litorimonas | neg\_3592 | 0.818181812763214 | 0.00381492051076338 |
| Zhihengliuella | neg\_3592 | -0.854545474052429 | 0.00163680247839526 |
| Lihuaxuella | neg\_3592 | 0.842424213886261 | 0.00222003275351312 |
| Lawsonella | neg\_3592 | 0.814593434333801 | 0.00410613103846336 |
| Rodentibacter | neg\_3592 | 0.866666674613953 | 0.0011735379121256 |
| Palleronia | neg\_3592 | -0.808514356613159 | 0.00463528246178302 |
| Izhakiella | neg\_3592 | 0.948332667350769 | 2.92858403718732e-05 |
| Desulfocucumis | neg\_3592 | -0.842424213886261 | 0.00222003275351312 |
| Porticoccus | neg\_3592 | 0.818181812763214 | 0.00381492051076338 |
| Succinimonas | neg\_3592 | -0.842424213886261 | 0.00222003275351312 |
| Candidatus\_Arcanobacter | neg\_3592 | 0.866666674613953 | 0.0011735379121256 |
| Anaerotignum | neg\_3608 | -0.828283190727234 | 0.00307477558489877 |
| Erythrobacter | neg\_3608 | 0.80374151468277 | 0.00508357521208702 |
| Litorimonas | neg\_3608 | 0.877366662025452 | 0.000851188393096614 |
| Haliscomenobacter | neg\_3608 | 0.80374151468277 | 0.00508357521208702 |
| Zhihengliuella | neg\_3608 | -0.852824926376343 | 0.00171189540742711 |
| Rodentibacter | neg\_3608 | 0.840554058551788 | 0.00232177976116144 |
| Harryflintia | neg\_3608 | 0.852824926376343 | 0.00171189540742711 |
| Izhakiella | neg\_3608 | 0.85542106628418 | 0.00159951806374248 |
| Desulfocucumis | neg\_3608 | -0.877366662025452 | 0.000851188393096614 |
| Porticoccus | neg\_3608 | 0.852824926376343 | 0.00171189540742711 |
| Succinimonas | neg\_3608 | -0.828283190727234 | 0.00307477558489877 |
| Stappia | neg\_3609 | 0.830303013324738 | 0.00294022813612127 |
| Caloramator | neg\_3609 | 0.806060612201691 | 0.0048620605246823 |
| Anaerotignum | neg\_3609 | -0.903030276298523 | 0.000343612565232743 |
| Litorimonas | neg\_3609 | 0.806060612201691 | 0.0048620605246823 |
| Brevinema | neg\_3609 | 0.806060612201691 | 0.0048620605246823 |
| Haliscomenobacter | neg\_3609 | 0.806060612201691 | 0.0048620605246823 |
| Zhihengliuella | neg\_3609 | -0.854545474052429 | 0.00163680247839526 |
| Lawsonella | neg\_3609 | 0.924016416072845 | 0.000132952728702396 |
| Rodentibacter | neg\_3609 | 0.878787875175476 | 0.000813862205061078 |
| Palleronia | neg\_3609 | -0.808514356613159 | 0.00463528246178302 |
| Marivita | neg\_3609 | -0.842424213886261 | 0.00222003275351312 |
| Izhakiella | neg\_3609 | 0.875383973121643 | 0.00090532610126548 |
| Desulfocucumis | neg\_3609 | -0.866666674613953 | 0.0011735379121256 |
| Succinimonas | neg\_3609 | -0.854545474052429 | 0.00163680247839526 |
| Litorimonas | neg\_3637 | 0.878787875175476 | 0.000813862205061078 |
| Brevinema | neg\_3637 | 0.806060612201691 | 0.0048620605246823 |
| Haliscomenobacter | neg\_3637 | 0.842424213886261 | 0.00222003275351312 |
| Zhihengliuella | neg\_3637 | -0.890909075737 | 0.000542144516154419 |
| Lawsonella | neg\_3637 | 0.832830607891083 | 0.00277788845123905 |
| Rodentibacter | neg\_3637 | 0.830303013324738 | 0.00294022813612127 |
| Harryflintia | neg\_3637 | 0.842424213886261 | 0.00222003275351312 |
| Izhakiella | neg\_3637 | 0.832830607891083 | 0.00277788845123905 |
| Desulfocucumis | neg\_3637 | -0.806060612201691 | 0.0048620605246823 |
| Porticoccus | neg\_3637 | 0.830303013324738 | 0.00294022813612127 |
| Succinimonas | neg\_3637 | -0.866666674613953 | 0.0011735379121256 |
| Anaerotignum | neg\_3653 | -0.842424213886261 | 0.00222003275351312 |
| Litorimonas | neg\_3653 | 0.818181812763214 | 0.00381492051076338 |
| Haliscomenobacter | neg\_3653 | 0.806060612201691 | 0.0048620605246823 |
| Zhihengliuella | neg\_3653 | -0.842424213886261 | 0.00222003275351312 |
| Lawsonella | neg\_3653 | 0.875383973121643 | 0.00090532610126548 |
| Rodentibacter | neg\_3653 | 0.830303013324738 | 0.00294022813612127 |
| Harryflintia | neg\_3653 | 0.806060612201691 | 0.0048620605246823 |
| Izhakiella | neg\_3653 | 0.875383973121643 | 0.00090532610126548 |
| Desulfocucumis | neg\_3653 | -0.854545474052429 | 0.00163680247839526 |
| Succinimonas | neg\_3653 | -0.854545474052429 | 0.00163680247839526 |
| Anaerotignum | neg\_3656 | 0.806060612201691 | 0.0048620605246823 |
| Candidatus\_Kryptonium | neg\_3656 | -0.878787875175476 | 0.000813862205061078 |
| Ottowia | neg\_3656 | 0.830303013324738 | 0.00294022813612127 |
| Melissococcus | neg\_3656 | 0.842424213886261 | 0.00222003275351312 |
| Thiodictyon | neg\_3656 | 0.878787875175476 | 0.000813862205061078 |
| Promicromonospora | neg\_3656 | -0.806060612201691 | 0.0048620605246823 |
| Leminorella | neg\_3656 | 0.818181812763214 | 0.00381492051076338 |
| Stappia | neg\_3666 | 0.830303013324738 | 0.00294022813612127 |
| Caloramator | neg\_3666 | 0.866666674613953 | 0.0011735379121256 |
| Chloroflexus | neg\_3666 | 0.818181812763214 | 0.00381492051076338 |
| Candidatus\_Tokpelaia | neg\_3666 | 0.842424213886261 | 0.00222003275351312 |
| Pakpunavirus | neg\_3666 | 0.857146799564362 | 0.0015278405327328 |
| Lawsonella | neg\_3666 | 0.826751530170441 | 0.00317971472634282 |
| Kordiimonas | neg\_3666 | 0.806060612201691 | 0.0048620605246823 |
| Microscilla | neg\_3666 | 0.830303013324738 | 0.00294022813612127 |
| Halothiobacillus | neg\_3666 | -0.854545474052429 | 0.00163680247839526 |
| Candidatus\_Electrothrix | neg\_3666 | 0.818181812763214 | 0.00381492051076338 |
| Cvm10virus | neg\_3666 | 0.81915009021759 | 0.00373894966455346 |
| Parafilimonas | neg\_3666 | -0.854545474052429 | 0.00163680247839526 |
| Thermobifida | neg\_3689 | -0.806060612201691 | 0.0048620605246823 |
| Stappia | neg\_3689 | 0.842424213886261 | 0.00222003275351312 |
| Anaerotignum | neg\_3689 | -0.854545474052429 | 0.00163680247839526 |
| Haliscomenobacter | neg\_3689 | 0.842424213886261 | 0.00222003275351312 |
| Zhihengliuella | neg\_3689 | -0.854545474052429 | 0.00163680247839526 |
| Lihuaxuella | neg\_3689 | 0.818181812763214 | 0.00381492051076338 |
| Lawsonella | neg\_3689 | 0.930095493793488 | 9.59607762942571e-05 |
| Rodentibacter | neg\_3689 | 0.830303013324738 | 0.00294022813612127 |
| Palleronia | neg\_3689 | -0.881463050842285 | 0.000746860533753502 |
| Izhakiella | neg\_3689 | 0.881463050842285 | 0.000746860533753502 |
| Succinimonas | neg\_3689 | -0.866666674613953 | 0.0011735379121256 |
| Candidatus\_Arcanobacter | neg\_3689 | 0.806060612201691 | 0.0048620605246823 |
| Litorimonas | neg\_3705 | -0.806060612201691 | 0.0048620605246823 |
| Haliscomenobacter | neg\_3705 | -0.806060612201691 | 0.0048620605246823 |
| Porticoccus | neg\_3705 | -0.854545474052429 | 0.00163680247839526 |
| Succinimonas | neg\_3705 | 0.806060612201691 | 0.0048620605246823 |
| Lmd1virus | neg\_3723 | 0.806643962860107 | 0.00480744414521128 |
| Anaerotignum | neg\_3739 | -0.830303013324738 | 0.00294022813612127 |
| Erythrobacter | neg\_3739 | 0.842424213886261 | 0.00222003275351312 |
| Kriegella | neg\_3739 | 0.830303013324738 | 0.00294022813612127 |
| Candidatus\_Kryptonium | neg\_3739 | 0.842424213886261 | 0.00222003275351312 |
| Parapedobacter | neg\_3739 | 0.854545474052429 | 0.00163680247839526 |
| Pakpunavirus | neg\_3739 | 0.857146799564362 | 0.0015278405327328 |
| Ottowia | neg\_3739 | -0.939393937587738 | 5.48405364009241e-05 |
| Propionicimonas | neg\_3739 | 0.917937397956848 | 0.00017952998751225 |
| Candidatus\_Sulfopaludibacter | neg\_3739 | 0.806060612201691 | 0.0048620605246823 |
| Palleronia | neg\_3739 | -0.869304955005646 | 0.00108698415655106 |
| Sinirhodobacter | neg\_3739 | -0.806060612201691 | 0.0048620605246823 |
| Microscilla | neg\_3739 | 0.915151536464691 | 0.000204472206099204 |
| Melghirimyces | neg\_3739 | 0.842424213886261 | 0.00222003275351312 |
| Marmoricola | neg\_3739 | -0.915151536464691 | 0.000204472206099204 |
| Novimethylophilus | neg\_3739 | 0.890909075737 | 0.000542144516154419 |
| Jiangella | neg\_3739 | 0.878787875175476 | 0.000813862205061078 |
| Melissococcus | neg\_3739 | -0.915151536464691 | 0.000204472206099204 |
| Mailhella | neg\_3739 | -0.915151536464691 | 0.000204472206099204 |
| Fimbriimonas | neg\_3739 | 0.818181812763214 | 0.00381492051076338 |
| Candidatus\_Kryptobacter | neg\_3739 | -0.830303013324738 | 0.00294022813612127 |
| Candidatus\_Sulfopaludibacter | neg\_3759 | 0.840554058551788 | 0.00232177976116144 |
| Candidatus\_Electrothrix | neg\_3759 | 0.828283190727234 | 0.00307477558489877 |
| Lmd1virus | neg\_3762 | -0.800390899181366 | 0.0054162086926719 |
| Mongoliibacter | neg\_3785 | 0.854545474052429 | 0.00163680247839526 |
| Emticicia | neg\_3785 | 0.842424213886261 | 0.00222003275351312 |
| Suttonella | neg\_3785 | -0.842424213886261 | 0.00222003275351312 |
| Candidatus\_Symbiobacter | neg\_3785 | 0.866666674613953 | 0.0011735379121256 |
| Anaerotignum | neg\_3807 | -0.806060612201691 | 0.0048620605246823 |
| Lawsonella | neg\_3807 | 0.875383973121643 | 0.00090532610126548 |
| Palleronia | neg\_3807 | -0.802435338497162 | 0.00521145970824932 |
| Dethiosulfovibrio | neg\_3807 | -0.854545474052429 | 0.00163680247839526 |
| Izhakiella | neg\_3807 | 0.802435338497162 | 0.00521145970824932 |
| Leminorella | neg\_3807 | -0.830303013324738 | 0.00294022813612127 |
| Amantichitinum | neg\_3807 | 0.806060612201691 | 0.0048620605246823 |
| Thermobifida | neg\_3820 | -0.830303013324738 | 0.00294022813612127 |
| Stappia | neg\_3820 | 0.818181812763214 | 0.00381492051076338 |
| Anaerotignum | neg\_3820 | -0.866666674613953 | 0.0011735379121256 |
| Erythrobacter | neg\_3820 | 0.878787875175476 | 0.000813862205061078 |
| Litorimonas | neg\_3820 | 0.854545474052429 | 0.00163680247839526 |
| Lawsonella | neg\_3820 | 0.820672512054443 | 0.00362170350001234 |
| Rodentibacter | neg\_3820 | 0.842424213886261 | 0.00222003275351312 |
| Marivita | neg\_3820 | -0.806060612201691 | 0.0048620605246823 |
| Acetivibrio | neg\_3820 | -0.854545474052429 | 0.00163680247839526 |
| Harryflintia | neg\_3820 | 0.830303013324738 | 0.00294022813612127 |
| Trichormus | neg\_3820 | -0.806060612201691 | 0.0048620605246823 |
| Fimbriimonas | neg\_3820 | 0.806060612201691 | 0.0048620605246823 |
| Izhakiella | neg\_3820 | 0.820672512054443 | 0.00362170350001234 |
| Candidatus\_Sulfotelmatomonas | neg\_3820 | 0.842424213886261 | 0.00222003275351312 |
| Desulfocucumis | neg\_3820 | -0.927272737026215 | 0.000112034447641074 |
| Promicromonospora | neg\_3820 | 0.842424213886261 | 0.00222003275351312 |
| Mudcatvirus | neg\_3820 | 0.854545474052429 | 0.00163680247839526 |
| Caloramator | neg\_3852 | 0.830303013324738 | 0.00294022813612127 |
| Catenovulum | neg\_3852 | -0.903030276298523 | 0.000343612565232743 |
| Kriegella | neg\_3852 | 0.854545474052429 | 0.00163680247839526 |
| Candidatus\_Kryptonium | neg\_3852 | 0.806060612201691 | 0.0048620605246823 |
| Mycolicibacter | neg\_3852 | -0.842424213886261 | 0.00222003275351312 |
| Parapedobacter | neg\_3852 | 0.842424213886261 | 0.00222003275351312 |
| Propionicimonas | neg\_3852 | 0.802435338497162 | 0.00521145970824932 |
| Lawsonella | neg\_3852 | 0.905779242515564 | 0.000307323103084567 |
| Microscilla | neg\_3852 | 0.818181812763214 | 0.00381492051076338 |
| Thermoanaerobacter | neg\_3852 | 0.854545474052429 | 0.00163680247839526 |
| Sideroxydans | neg\_3852 | -0.830303013324738 | 0.00294022813612127 |
| Anoxybacillus | neg\_3852 | 0.806060612201691 | 0.0048620605246823 |
| Variovorax | neg\_3852 | 0.878787875175476 | 0.000813862205061078 |
| Caldicellulosiruptor | neg\_3852 | 0.854545474052429 | 0.00163680247839526 |
| Cvm10virus | neg\_3852 | 0.875427544116974 | 0.000904110124716251 |
| Beggiatoa | neg\_3889 | -0.915151536464691 | 0.000204472206099204 |
| Anaerotignum | neg\_392 | -0.842424213886261 | 0.00222003275351312 |
| Litorimonas | neg\_392 | 0.818181812763214 | 0.00381492051076338 |
| Haliscomenobacter | neg\_392 | 0.806060612201691 | 0.0048620605246823 |
| Zhihengliuella | neg\_392 | -0.842424213886261 | 0.00222003275351312 |
| Lawsonella | neg\_392 | 0.875383973121643 | 0.00090532610126548 |
| Rodentibacter | neg\_392 | 0.830303013324738 | 0.00294022813612127 |
| Harryflintia | neg\_392 | 0.806060612201691 | 0.0048620605246823 |
| Izhakiella | neg\_392 | 0.875383973121643 | 0.00090532610126548 |
| Desulfocucumis | neg\_392 | -0.854545474052429 | 0.00163680247839526 |
| Succinimonas | neg\_392 | -0.854545474052429 | 0.00163680247839526 |
| Anaerotignum | neg\_3940 | -0.854545474052429 | 0.00163680247839526 |
| Litorimonas | neg\_3940 | 0.866666674613953 | 0.0011735379121256 |
| Haliscomenobacter | neg\_3940 | 0.842424213886261 | 0.00222003275351312 |
| Zhihengliuella | neg\_3940 | -0.878787875175476 | 0.000813862205061078 |
| Lawsonella | neg\_3940 | 0.936174511909485 | 6.71888372667517e-05 |
| Rodentibacter | neg\_3940 | 0.854545474052429 | 0.00163680247839526 |
| Palleronia | neg\_3940 | -0.802435338497162 | 0.00521145970824932 |
| Limnobacter | neg\_3940 | 0.820672512054443 | 0.00362170350001234 |
| Trichormus | neg\_3940 | -0.818181812763214 | 0.00381492051076338 |
| Izhakiella | neg\_3940 | 0.875383973121643 | 0.00090532610126548 |
| Succinimonas | neg\_3940 | -0.890909075737 | 0.000542144516154419 |
| Thermobifida | neg\_410 | -0.818181812763214 | 0.00381492051076338 |
| Stappia | neg\_410 | 0.854545474052429 | 0.00163680247839526 |
| Caloramator | neg\_410 | 0.915151536464691 | 0.000204472206099204 |
| Chloroflexus | neg\_410 | 0.854545474052429 | 0.00163680247839526 |
| Defluviimonas | neg\_410 | -0.854545474052429 | 0.00163680247839526 |
| Anaerobacterium | neg\_410 | 0.951515138149261 | 2.27985739738035e-05 |
| Methanosphaerula | neg\_410 | -0.842424213886261 | 0.00222003275351312 |
| Natribacillus | neg\_410 | 0.927272737026215 | 0.000112034447641074 |
| Candidatus\_Halobonum | neg\_410 | 0.863225877285004 | 0.00129381457010247 |
| Thermobifida | neg\_412 | -0.818181812763214 | 0.00381492051076338 |
| Haliscomenobacter | neg\_412 | 0.878787875175476 | 0.000813862205061078 |
| Emticicia | neg\_412 | 0.878787875175476 | 0.000813862205061078 |
| Suttonella | neg\_412 | -0.854545474052429 | 0.00163680247839526 |
| Harryflintia | neg\_412 | 0.818181812763214 | 0.00381492051076338 |
| Candidatus\_Symbiobacter | neg\_412 | 0.830303013324738 | 0.00294022813612127 |
| Succinimonas | neg\_412 | -0.854545474052429 | 0.00163680247839526 |
| Anaerotignum | neg\_420 | -0.806060612201691 | 0.0048620605246823 |
| Zhihengliuella | neg\_420 | -0.890909075737 | 0.000542144516154419 |
| Lawsonella | neg\_420 | 0.83890962600708 | 0.00241398974898654 |
| Rodentibacter | neg\_420 | 0.866666674613953 | 0.0011735379121256 |
| Izhakiella | neg\_420 | 0.875383973121643 | 0.00090532610126548 |
| Succinimonas | neg\_420 | -0.818181812763214 | 0.00381492051076338 |
| Thermobifida | neg\_430 | -0.840554058551788 | 0.00232177976116144 |
| Stappia | neg\_430 | 0.828283190727234 | 0.00307477558489877 |
| Litorimonas | neg\_430 | 0.80374151468277 | 0.00508357521208702 |
| Haliscomenobacter | neg\_430 | 0.865095794200897 | 0.00122739260821847 |
| Zhihengliuella | neg\_430 | -0.816012322902679 | 0.0039891464803441 |
| Lawsonella | neg\_430 | 0.867729306221008 | 0.00113809565686207 |
| Harryflintia | neg\_430 | 0.840554058551788 | 0.00232177976116144 |
| Izhakiella | neg\_430 | 0.818496406078339 | 0.00379011736651003 |
| Desulfocucumis | neg\_430 | -0.816012322902679 | 0.0039891464803441 |
| Succinimonas | neg\_430 | -0.889637529850006 | 0.000566964581027118 |
| Rubrivivax | neg\_483 | -0.890909075737 | 0.000542144516154419 |
| Palleronia | neg\_497 | 0.808514356613159 | 0.00463528246178302 |
| Limnobacter | neg\_497 | -0.851067781448364 | 0.00179112909994994 |
| Izhakiella | neg\_497 | -0.869304955005646 | 0.00108698415655106 |
| Thermobifida | neg\_552 | -0.806060612201691 | 0.0048620605246823 |
| Anaerotignum | neg\_552 | -0.903030276298523 | 0.000343612565232743 |
| Erythrobacter | neg\_552 | 0.903030276298523 | 0.000343612565232743 |
| Litorimonas | neg\_552 | 0.842424213886261 | 0.00222003275351312 |
| Pakpunavirus | neg\_552 | 0.802435338497162 | 0.00521145970824932 |
| Zhihengliuella | neg\_552 | -0.806060612201691 | 0.0048620605246823 |
| Lihuaxuella | neg\_552 | 0.818181812763214 | 0.00381492051076338 |
| Rodentibacter | neg\_552 | 0.878787875175476 | 0.000813862205061078 |
| Candidatus\_Sulfopaludibacter | neg\_552 | 0.818181812763214 | 0.00381492051076338 |
| Marivita | neg\_552 | -0.830303013324738 | 0.00294022813612127 |
| Acetivibrio | neg\_552 | -0.866666674613953 | 0.0011735379121256 |
| Harryflintia | neg\_552 | 0.806060612201691 | 0.0048620605246823 |
| Fimbriimonas | neg\_552 | 0.818181812763214 | 0.00381492051076338 |
| Izhakiella | neg\_552 | 0.857146799564362 | 0.0015278405327328 |
| Candidatus\_Sulfotelmatomonas | neg\_552 | 0.830303013324738 | 0.00294022813612127 |
| Desulfocucumis | neg\_552 | -0.939393937587738 | 5.48405364009241e-05 |
| Promicromonospora | neg\_552 | 0.830303013324738 | 0.00294022813612127 |
| Mudcatvirus | neg\_552 | 0.866666674613953 | 0.0011735379121256 |
| Porticoccus | neg\_552 | 0.806060612201691 | 0.0048620605246823 |
| Brevinema | neg\_560 | -0.830303013324738 | 0.00294022813612127 |
| Mycolicibacter | neg\_560 | 0.842424213886261 | 0.00222003275351312 |
| Candidatus\_Moduliflexus | neg\_560 | -0.915151536464691 | 0.000204472206099204 |
| Marivita | neg\_560 | 0.842424213886261 | 0.00222003275351312 |
| Comamonas | neg\_560 | 0.878787875175476 | 0.000813862205061078 |
| Anoxybacillus | neg\_560 | -0.830303013324738 | 0.00294022813612127 |
| Cvm10virus | neg\_560 | -0.90043979883194 | 0.000380598613888061 |
| Brevinema | neg\_580 | -0.830303013324738 | 0.00294022813612127 |
| Rodentibacter | neg\_580 | -0.806060612201691 | 0.0048620605246823 |
| Candidatus\_Sulfopaludibacter | neg\_580 | -0.806060612201691 | 0.0048620605246823 |
| Dolosicoccus | neg\_580 | 0.854545474052429 | 0.00163680247839526 |
| Candidatus\_Electrothrix | neg\_580 | -0.806060612201691 | 0.0048620605246823 |
| Thermopetrobacter | neg\_580 | -0.806060612201691 | 0.0048620605246823 |
| Thermobifida | neg\_59 | 0.830303013324738 | 0.00294022813612127 |
| Stappia | neg\_59 | -0.830303013324738 | 0.00294022813612127 |
| Anaerotignum | neg\_59 | 0.890909075737 | 0.000542144516154419 |
| Erythrobacter | neg\_59 | -0.854545474052429 | 0.00163680247839526 |
| Brevinema | neg\_59 | -0.818181812763214 | 0.00381492051076338 |
| Pakpunavirus | neg\_59 | -0.802435338497162 | 0.00521145970824932 |
| Ottowia | neg\_59 | 0.854545474052429 | 0.00163680247839526 |
| Zhihengliuella | neg\_59 | 0.818181812763214 | 0.00381492051076338 |
| Lihuaxuella | neg\_59 | -0.866666674613953 | 0.0011735379121256 |
| Rodentibacter | neg\_59 | -0.866666674613953 | 0.0011735379121256 |
| Candidatus\_Sulfopaludibacter | neg\_59 | -0.854545474052429 | 0.00163680247839526 |
| Palleronia | neg\_59 | 0.851067781448364 | 0.00179112909994994 |
| Marivita | neg\_59 | 0.818181812763214 | 0.00381492051076338 |
| Acetivibrio | neg\_59 | 0.890909075737 | 0.000542144516154419 |
| Dolosicoccus | neg\_59 | 0.806060612201691 | 0.0048620605246823 |
| Izhakiella | neg\_59 | -0.863225877285004 | 0.00129381457010247 |
| Thiodictyon | neg\_59 | 0.818181812763214 | 0.00381492051076338 |
| Desulfocucumis | neg\_59 | 0.903030276298523 | 0.000343612565232743 |
| Promicromonospora | neg\_59 | -0.818181812763214 | 0.00381492051076338 |
| Mudcatvirus | neg\_59 | -0.842424213886261 | 0.00222003275351312 |
| Candidatus\_Arcanobacter | neg\_59 | -0.806060612201691 | 0.0048620605246823 |
| Zhihengliuella | neg\_637 | 0.866666674613953 | 0.0011735379121256 |
| Dolosicoccus | neg\_637 | 0.878787875175476 | 0.000813862205061078 |
| Thermopetrobacter | neg\_637 | -0.878787875175476 | 0.000813862205061078 |
| Anaerotignum | neg\_64 | -0.878787875175476 | 0.000813862205061078 |
| Erythrobacter | neg\_64 | 0.878787875175476 | 0.000813862205061078 |
| Kriegella | neg\_64 | 0.915151536464691 | 0.000204472206099204 |
| Candidatus\_Kryptonium | neg\_64 | 0.842424213886261 | 0.00222003275351312 |
| Parapedobacter | neg\_64 | 0.854545474052429 | 0.00163680247839526 |
| Ottowia | neg\_64 | -0.818181812763214 | 0.00381492051076338 |
| Propionicimonas | neg\_64 | 0.887542068958282 | 0.000609666476501403 |
| Lawsonella | neg\_64 | 0.802435338497162 | 0.00521145970824932 |
| Rodentibacter | neg\_64 | 0.915151536464691 | 0.000204472206099204 |
| Palleronia | neg\_64 | -0.930095493793488 | 9.59607762942571e-05 |
| Sinirhodobacter | neg\_64 | -0.830303013324738 | 0.00294022813612127 |
| Microscilla | neg\_64 | 0.951515138149261 | 2.27985739738035e-05 |
| Limnobacter | neg\_64 | 0.83890962600708 | 0.00241398974898654 |
| Melghirimyces | neg\_64 | 0.866666674613953 | 0.0011735379121256 |
| Novimethylophilus | neg\_64 | 0.890909075737 | 0.000542144516154419 |
| Jiangella | neg\_64 | 0.854545474052429 | 0.00163680247839526 |
| Acidaminobacter | neg\_64 | 0.866666674613953 | 0.0011735379121256 |
| Trichormus | neg\_64 | -0.830303013324738 | 0.00294022813612127 |
| Sideroxydans | neg\_64 | -0.842424213886261 | 0.00222003275351312 |
| Lawsonella | neg\_675 | -0.844988703727722 | 0.00208579660703911 |
| Variovorax | neg\_675 | -0.830303013324738 | 0.00294022813612127 |
| Thermobifida | neg\_677 | -0.818181812763214 | 0.00381492051076338 |
| Pakpunavirus | neg\_677 | 0.863225877285004 | 0.00129381457010247 |
| Ottowia | neg\_677 | -0.854545474052429 | 0.00163680247839526 |
| Haliscomenobacter | neg\_677 | 0.818181812763214 | 0.00381492051076338 |
| Lihuaxuella | neg\_677 | 0.903030276298523 | 0.000343612565232743 |
| Candidatus\_Sulfopaludibacter | neg\_677 | 0.830303013324738 | 0.00294022813612127 |
| Fimbriimonas | neg\_677 | 0.818181812763214 | 0.00381492051076338 |
| Thermopetrobacter | neg\_677 | 0.818181812763214 | 0.00381492051076338 |
| Candidatus\_Arcanobacter | neg\_677 | 0.854545474052429 | 0.00163680247839526 |
| Catenovulum | neg\_685 | -0.806060612201691 | 0.0048620605246823 |
| Kriegella | neg\_685 | 0.818181812763214 | 0.00381492051076338 |
| Brevinema | neg\_685 | 0.842424213886261 | 0.00222003275351312 |
| Limnobacter | neg\_685 | 0.826751530170441 | 0.00317971472634282 |
| Thermoanaerobacter | neg\_685 | 0.878787875175476 | 0.000813862205061078 |
| Lmd1virus | neg\_685 | -0.806643962860107 | 0.00480744414521128 |
| Sideroxydans | neg\_685 | -0.806060612201691 | 0.0048620605246823 |
| Litorimonas | neg\_708 | -0.806060612201691 | 0.0048620605246823 |
| Porticoccus | neg\_708 | -0.915151536464691 | 0.000204472206099204 |
| Anaerotignum | neg\_71 | -0.878787875175476 | 0.000813862205061078 |
| Kriegella | neg\_71 | 0.830303013324738 | 0.00294022813612127 |
| Candidatus\_Kryptonium | neg\_71 | 0.878787875175476 | 0.000813862205061078 |
| Zhihengliuella | neg\_71 | -0.830303013324738 | 0.00294022813612127 |
| Lawsonella | neg\_71 | 0.893621146678925 | 0.000491873064334847 |
| Rodentibacter | neg\_71 | 0.866666674613953 | 0.0011735379121256 |
| Palleronia | neg\_71 | -0.948332667350769 | 2.92858403718732e-05 |
| Sinirhodobacter | neg\_71 | -0.866666674613953 | 0.0011735379121256 |
| Microscilla | neg\_71 | 0.830303013324738 | 0.00294022813612127 |
| Limnobacter | neg\_71 | 0.802435338497162 | 0.00521145970824932 |
| Melghirimyces | neg\_71 | 0.854545474052429 | 0.00163680247839526 |
| Izhakiella | neg\_71 | 0.857146799564362 | 0.0015278405327328 |
| Leminorella | neg\_71 | -0.818181812763214 | 0.00381492051076338 |
| Anaerotignum | neg\_726 | -0.842424213886261 | 0.00222003275351312 |
| Litorimonas | neg\_726 | 0.818181812763214 | 0.00381492051076338 |
| Haliscomenobacter | neg\_726 | 0.806060612201691 | 0.0048620605246823 |
| Zhihengliuella | neg\_726 | -0.842424213886261 | 0.00222003275351312 |
| Lawsonella | neg\_726 | 0.875383973121643 | 0.00090532610126548 |
| Rodentibacter | neg\_726 | 0.830303013324738 | 0.00294022813612127 |
| Harryflintia | neg\_726 | 0.806060612201691 | 0.0048620605246823 |
| Izhakiella | neg\_726 | 0.875383973121643 | 0.00090532610126548 |
| Desulfocucumis | neg\_726 | -0.854545474052429 | 0.00163680247839526 |
| Succinimonas | neg\_726 | -0.854545474052429 | 0.00163680247839526 |
| Thermobifida | neg\_727 | -0.852824926376343 | 0.00171189540742711 |
| Litorimonas | neg\_727 | 0.901908397674561 | 0.000359290276283897 |
| Brevinema | neg\_727 | 0.877366662025452 | 0.000851188393096614 |
| Candidatus\_Tokpelaia | neg\_727 | 0.80374151468277 | 0.00508357521208702 |
| Haliscomenobacter | neg\_727 | 0.889637529850006 | 0.000566964581027118 |
| Zhihengliuella | neg\_727 | -0.901908397674561 | 0.000359290276283897 |
| Lawsonella | neg\_727 | 0.843112826347351 | 0.00218339276204738 |
| Rodentibacter | neg\_727 | 0.80374151468277 | 0.00508357521208702 |
| Limnobacter | neg\_727 | 0.85542106628418 | 0.00159951806374248 |
| Lmd1virus | neg\_727 | -0.848254203796387 | 0.00192348015922827 |
| Porticoccus | neg\_727 | 0.816012322902679 | 0.0039891464803441 |
| Succinimonas | neg\_727 | -0.865095794200897 | 0.00122739260821847 |
| Thermobifida | neg\_757 | -0.818181812763214 | 0.00381492051076338 |
| Litorimonas | neg\_757 | 0.866666674613953 | 0.0011735379121256 |
| Brevinema | neg\_757 | 0.818181812763214 | 0.00381492051076338 |
| Haliscomenobacter | neg\_757 | 0.830303013324738 | 0.00294022813612127 |
| Zhihengliuella | neg\_757 | -0.866666674613953 | 0.0011735379121256 |
| Rodentibacter | neg\_757 | 0.806060612201691 | 0.0048620605246823 |
| Marivita | neg\_757 | -0.806060612201691 | 0.0048620605246823 |
| Harryflintia | neg\_757 | 0.830303013324738 | 0.00294022813612127 |
| Desulfocucumis | neg\_757 | -0.830303013324738 | 0.00294022813612127 |
| Porticoccus | neg\_757 | 0.818181812763214 | 0.00381492051076338 |
| Succinimonas | neg\_757 | -0.830303013324738 | 0.00294022813612127 |
| Anaerotignum | neg\_758 | -0.818181812763214 | 0.00381492051076338 |
| Erythrobacter | neg\_758 | 0.878787875175476 | 0.000813862205061078 |
| Kriegella | neg\_758 | 0.890909075737 | 0.000542144516154419 |
| Ottowia | neg\_758 | -0.854545474052429 | 0.00163680247839526 |
| Propionicimonas | neg\_758 | 0.887542068958282 | 0.000609666476501403 |
| Rodentibacter | neg\_758 | 0.854545474052429 | 0.00163680247839526 |
| Palleronia | neg\_758 | -0.911858320236206 | 0.000237143587637378 |
| Sinirhodobacter | neg\_758 | -0.806060612201691 | 0.0048620605246823 |
| Larkinella | neg\_758 | 0.830303013324738 | 0.00294022813612127 |
| Microscilla | neg\_758 | 0.927272737026215 | 0.000112034447641074 |
| Limnobacter | neg\_758 | 0.863225877285004 | 0.00129381457010247 |
| Melghirimyces | neg\_758 | 0.818181812763214 | 0.00381492051076338 |
| Novimethylophilus | neg\_758 | 0.866666674613953 | 0.0011735379121256 |
| Jiangella | neg\_758 | 0.830303013324738 | 0.00294022813612127 |
| Acidaminobacter | neg\_758 | 0.830303013324738 | 0.00294022813612127 |
| Trichormus | neg\_758 | -0.806060612201691 | 0.0048620605246823 |
| Fimbriimonas | neg\_758 | 0.806060612201691 | 0.0048620605246823 |
| Eah2virus | neg\_758 | 0.833002269268036 | 0.00276710271221536 |
| Mycolicibacter | neg\_763 | 0.818181812763214 | 0.00381492051076338 |
| Cvm10virus | neg\_763 | -0.806643962860107 | 0.00480744414521128 |
| Beggiatoa | neg\_763 | -0.878787875175476 | 0.000813862205061078 |
| Catenovulum | neg\_769 | 0.903030276298523 | 0.000343612565232743 |
| Kriegella | neg\_769 | -0.951515138149261 | 2.27985739738035e-05 |
| Parapedobacter | neg\_769 | -0.830303013324738 | 0.00294022813612127 |
| Propionicimonas | neg\_769 | -0.911858320236206 | 0.000237143587637378 |
| Microscilla | neg\_769 | -0.842424213886261 | 0.00222003275351312 |
| Limnobacter | neg\_769 | -0.911858320236206 | 0.000237143587637378 |
| Thermoanaerobacter | neg\_769 | -0.890909075737 | 0.000542144516154419 |
| Jiangella | neg\_769 | -0.818181812763214 | 0.00381492051076338 |
| Negativicoccus | neg\_769 | 0.818181812763214 | 0.00381492051076338 |
| Acidaminobacter | neg\_769 | -0.818181812763214 | 0.00381492051076338 |
| Trichormus | neg\_769 | 0.842424213886261 | 0.00222003275351312 |
| Sideroxydans | neg\_769 | 0.878787875175476 | 0.000813862205061078 |
| Mailhella | neg\_769 | 0.818181812763214 | 0.00381492051076338 |
| Synergistes | neg\_769 | 0.903030276298523 | 0.000343612565232743 |
| Flexilinea | neg\_769 | 0.818181812763214 | 0.00381492051076338 |
| Eah2virus | neg\_769 | -0.846658051013947 | 0.00200163182665913 |
| Mongoliibacter | neg\_771 | -0.915151536464691 | 0.000204472206099204 |
| Candidatus\_Symbiobacter | neg\_771 | -0.842424213886261 | 0.00222003275351312 |
| Thermobifida | neg\_781 | 0.818181812763214 | 0.00381492051076338 |
| Stappia | neg\_781 | -0.818181812763214 | 0.00381492051076338 |
| Anaerotignum | neg\_781 | 0.866666674613953 | 0.0011735379121256 |
| Ottowia | neg\_781 | 0.842424213886261 | 0.00222003275351312 |
| Haliscomenobacter | neg\_781 | -0.806060612201691 | 0.0048620605246823 |
| Lihuaxuella | neg\_781 | -0.939393937587738 | 5.48405364009241e-05 |
| Rodentibacter | neg\_781 | -0.818181812763214 | 0.00381492051076338 |
| Candidatus\_Sulfopaludibacter | neg\_781 | -0.878787875175476 | 0.000813862205061078 |
| Palleronia | neg\_781 | 0.814593434333801 | 0.00410613103846336 |
| Acetivibrio | neg\_781 | 0.806060612201691 | 0.0048620605246823 |
| Melghirimyces | neg\_781 | -0.806060612201691 | 0.0048620605246823 |
| Dolosicoccus | neg\_781 | 0.806060612201691 | 0.0048620605246823 |
| Izhakiella | neg\_781 | -0.899700224399567 | 0.000391673296721073 |
| Thermopetrobacter | neg\_781 | -0.806060612201691 | 0.0048620605246823 |
| Desulfocucumis | neg\_781 | 0.854545474052429 | 0.00163680247839526 |
| Succinimonas | neg\_781 | 0.806060612201691 | 0.0048620605246823 |
| Candidatus\_Arcanobacter | neg\_781 | -0.878787875175476 | 0.000813862205061078 |
| Brevinema | neg\_783 | -0.878787875175476 | 0.000813862205061078 |
| Candidatus\_Tokpelaia | neg\_783 | -0.830303013324738 | 0.00294022813612127 |
| Candidatus\_Sulfopaludibacter | neg\_783 | -0.818181812763214 | 0.00381492051076338 |
| Kordiimonas | neg\_783 | -0.866666674613953 | 0.0011735379121256 |
| Dolosicoccus | neg\_783 | 0.806060612201691 | 0.0048620605246823 |
| Candidatus\_Electrothrix | neg\_783 | -0.915151536464691 | 0.000204472206099204 |
| Rubrivivax | neg\_783 | 0.806060612201691 | 0.0048620605246823 |
| Thermobifida | neg\_794 | -0.866666674613953 | 0.0011735379121256 |
| Litorimonas | neg\_794 | 0.854545474052429 | 0.00163680247839526 |
| Brevinema | neg\_794 | 0.830303013324738 | 0.00294022813612127 |
| Haliscomenobacter | neg\_794 | 0.878787875175476 | 0.000813862205061078 |
| Zhihengliuella | neg\_794 | -0.878787875175476 | 0.000813862205061078 |
| Lmd1virus | neg\_794 | -0.806643962860107 | 0.00480744414521128 |
| Porticoccus | neg\_794 | 0.806060612201691 | 0.0048620605246823 |
| Succinimonas | neg\_794 | -0.830303013324738 | 0.00294022813612127 |
| Stappia | neg\_84 | -0.806060612201691 | 0.0048620605246823 |
| Anaerotignum | neg\_84 | 0.818181812763214 | 0.00381492051076338 |
| Candidatus\_Kryptonium | neg\_84 | -0.818181812763214 | 0.00381492051076338 |
| Haliscomenobacter | neg\_84 | -0.818181812763214 | 0.00381492051076338 |
| Lawsonella | neg\_84 | -0.942253589630127 | 4.53587077142714e-05 |
| Palleronia | neg\_84 | 0.826751530170441 | 0.00317971472634282 |
| Dethiosulfovibrio | neg\_84 | 0.806060612201691 | 0.0048620605246823 |
| Izhakiella | neg\_84 | -0.857146799564362 | 0.0015278405327328 |
| Succinimonas | neg\_84 | 0.866666674613953 | 0.0011735379121256 |
| Amantichitinum | neg\_84 | -0.842424213886261 | 0.00222003275351312 |
| Chloroflexus | neg\_842 | 0.830303013324738 | 0.00294022813612127 |
| Candidatus\_Tokpelaia | neg\_842 | 0.830303013324738 | 0.00294022813612127 |
| Pakpunavirus | neg\_842 | 0.802435338497162 | 0.00521145970824932 |
| Haliscomenobacter | neg\_842 | 0.806060612201691 | 0.0048620605246823 |
| Pararhodospirillum | neg\_842 | 0.818181812763214 | 0.00381492051076338 |
| Halothiobacillus | neg\_842 | -0.842424213886261 | 0.00222003275351312 |
| Parafilimonas | neg\_842 | -0.915151536464691 | 0.000204472206099204 |
| Succinimonas | neg\_842 | -0.806060612201691 | 0.0048620605246823 |
| Candidatus\_Tokpelaia | neg\_846 | -0.808135211467743 | 0.00466981917809761 |
| Zhihengliuella | neg\_846 | 0.808135211467743 | 0.00466981917809761 |
| Rodentibacter | neg\_846 | -0.821065366268158 | 0.00359188206024896 |
| Candidatus\_Sulfopaludibacter | neg\_846 | -0.846925735473633 | 0.0019883679465349 |
| Kordiimonas | neg\_846 | -0.859855890274048 | 0.00142005806532719 |
| Pararhodospirillum | neg\_846 | -0.898646354675293 | 0.000407860267078952 |
| Dolosicoccus | neg\_846 | 0.846925735473633 | 0.0019883679465349 |
| Candidatus\_Electrothrix | neg\_846 | -0.859855890274048 | 0.00142005806532719 |
| Thermopetrobacter | neg\_846 | -0.924506723880768 | 0.000129632545059266 |
| Anaerotignum | neg\_861 | -0.830303013324738 | 0.00294022813612127 |
| Litorimonas | neg\_861 | 0.854545474052429 | 0.00163680247839526 |
| Haliscomenobacter | neg\_861 | 0.818181812763214 | 0.00381492051076338 |
| Zhihengliuella | neg\_861 | -0.806060612201691 | 0.0048620605246823 |
| Lawsonella | neg\_861 | 0.802435338497162 | 0.00521145970824932 |
| Rodentibacter | neg\_861 | 0.806060612201691 | 0.0048620605246823 |
| Larkinella | neg\_861 | 0.830303013324738 | 0.00294022813612127 |
| Harryflintia | neg\_861 | 0.890909075737 | 0.000542144516154419 |
| Izhakiella | neg\_861 | 0.875383973121643 | 0.00090532610126548 |
| Desulfocucumis | neg\_861 | -0.830303013324738 | 0.00294022813612127 |
| Porticoccus | neg\_861 | 0.842424213886261 | 0.00222003275351312 |
| Succinimonas | neg\_861 | -0.866666674613953 | 0.0011735379121256 |
| Anaerotignum | neg\_911 | -0.927272737026215 | 0.000112034447641074 |
| Candidatus\_Kryptonium | neg\_911 | 0.830303013324738 | 0.00294022813612127 |
| Brevinema | neg\_911 | 0.806060612201691 | 0.0048620605246823 |
| Parapedobacter | neg\_911 | 0.818181812763214 | 0.00381492051076338 |
| Haliscomenobacter | neg\_911 | 0.818181812763214 | 0.00381492051076338 |
| Zhihengliuella | neg\_911 | -0.878787875175476 | 0.000813862205061078 |
| Lihuaxuella | neg\_911 | 0.830303013324738 | 0.00294022813612127 |
| Lawsonella | neg\_911 | 0.911858320236206 | 0.000237143587637378 |
| Rodentibacter | neg\_911 | 0.903030276298523 | 0.000343612565232743 |
| Palleronia | neg\_911 | -0.899700224399567 | 0.000391673296721073 |
| Marivita | neg\_911 | -0.806060612201691 | 0.0048620605246823 |
| Microscilla | neg\_911 | 0.806060612201691 | 0.0048620605246823 |
| Melghirimyces | neg\_911 | 0.842424213886261 | 0.00222003275351312 |
| Izhakiella | neg\_911 | 0.936174511909485 | 6.71888372667517e-05 |
| Desulfocucumis | neg\_911 | -0.830303013324738 | 0.00294022813612127 |
| Succinimonas | neg\_911 | -0.866666674613953 | 0.0011735379121256 |
| Candidatus\_Arcanobacter | neg\_911 | 0.830303013324738 | 0.00294022813612127 |
| Erythrobacter | neg\_919 | 0.806060612201691 | 0.0048620605246823 |
| Kriegella | neg\_919 | 0.854545474052429 | 0.00163680247839526 |
| Ottowia | neg\_919 | -0.854545474052429 | 0.00163680247839526 |
| Propionicimonas | neg\_919 | 0.83890962600708 | 0.00241398974898654 |
| Palleronia | neg\_919 | -0.887542068958282 | 0.000609666476501403 |
| Sinirhodobacter | neg\_919 | -0.818181812763214 | 0.00381492051076338 |
| Larkinella | neg\_919 | 0.806060612201691 | 0.0048620605246823 |
| Microscilla | neg\_919 | 0.830303013324738 | 0.00294022813612127 |
| Limnobacter | neg\_919 | 0.899700224399567 | 0.000391673296721073 |
| Marmoricola | neg\_919 | -0.854545474052429 | 0.00163680247839526 |
| Mailhella | neg\_919 | -0.806060612201691 | 0.0048620605246823 |
| Fimbriimonas | neg\_919 | 0.806060612201691 | 0.0048620605246823 |
| Leminorella | neg\_919 | -0.830303013324738 | 0.00294022813612127 |
| Eah2virus | neg\_919 | 0.846658051013947 | 0.00200163182665913 |
| Thermobifida | neg\_94 | 0.818181812763214 | 0.00381492051076338 |
| Stappia | neg\_94 | -0.818181812763214 | 0.00381492051076338 |
| Anaerotignum | neg\_94 | 0.866666674613953 | 0.0011735379121256 |
| Ottowia | neg\_94 | 0.842424213886261 | 0.00222003275351312 |
| Haliscomenobacter | neg\_94 | -0.806060612201691 | 0.0048620605246823 |
| Lihuaxuella | neg\_94 | -0.939393937587738 | 5.48405364009241e-05 |
| Rodentibacter | neg\_94 | -0.818181812763214 | 0.00381492051076338 |
| Candidatus\_Sulfopaludibacter | neg\_94 | -0.878787875175476 | 0.000813862205061078 |
| Palleronia | neg\_94 | 0.814593434333801 | 0.00410613103846336 |
| Acetivibrio | neg\_94 | 0.806060612201691 | 0.0048620605246823 |
| Melghirimyces | neg\_94 | -0.806060612201691 | 0.0048620605246823 |
| Dolosicoccus | neg\_94 | 0.806060612201691 | 0.0048620605246823 |
| Izhakiella | neg\_94 | -0.899700224399567 | 0.000391673296721073 |
| Thermopetrobacter | neg\_94 | -0.806060612201691 | 0.0048620605246823 |
| Desulfocucumis | neg\_94 | 0.854545474052429 | 0.00163680247839526 |
| Succinimonas | neg\_94 | 0.806060612201691 | 0.0048620605246823 |
| Candidatus\_Arcanobacter | neg\_94 | -0.878787875175476 | 0.000813862205061078 |
| Stappia | neg\_985 | 0.842424213886261 | 0.00222003275351312 |
| Caloramator | neg\_985 | 0.915151536464691 | 0.000204472206099204 |
| Anaerotignum | neg\_985 | -0.806060612201691 | 0.0048620605246823 |
| Erythrobacter | neg\_985 | 0.818181812763214 | 0.00381492051076338 |
| Litorimonas | neg\_985 | 0.818181812763214 | 0.00381492051076338 |
| Brevinema | neg\_985 | 0.830303013324738 | 0.00294022813612127 |
| Candidatus\_Tokpelaia | neg\_985 | 0.806060612201691 | 0.0048620605246823 |
| Zhihengliuella | neg\_985 | -0.806060612201691 | 0.0048620605246823 |
| Lawsonella | neg\_985 | 0.881463050842285 | 0.000746860533753502 |
| Rodentibacter | neg\_985 | 0.866666674613953 | 0.0011735379121256 |
| Marivita | neg\_985 | -0.878787875175476 | 0.000813862205061078 |
| Acetivibrio | neg\_985 | -0.818181812763214 | 0.00381492051076338 |
| Trichormus | neg\_985 | -0.818181812763214 | 0.00381492051076338 |
| Candidatus\_Electrothrix | neg\_985 | 0.806060612201691 | 0.0048620605246823 |
| Desulfocucumis | neg\_985 | -0.806060612201691 | 0.0048620605246823 |
| Mudcatvirus | neg\_985 | 0.818181812763214 | 0.00381492051076338 |
| Eah2virus | neg\_985 | 0.805690705776215 | 0.00489692183645341 |
| Thermobifida | neg\_994 | -0.854545474052429 | 0.00163680247839526 |
| Stappia | neg\_994 | 0.842424213886261 | 0.00222003275351312 |
| Brevinema | neg\_994 | 0.818181812763214 | 0.00381492051076338 |
| Chloroflexus | neg\_994 | 0.878787875175476 | 0.000813862205061078 |
| Mycolicibacter | neg\_994 | -0.842424213886261 | 0.00222003275351312 |
| Candidatus\_Tokpelaia | neg\_994 | 0.903030276298523 | 0.000343612565232743 |
| Pakpunavirus | neg\_994 | 0.911858320236206 | 0.000237143587637378 |
| Ottowia | neg\_994 | -0.842424213886261 | 0.00222003275351312 |
| Haliscomenobacter | neg\_994 | 0.830303013324738 | 0.00294022813612127 |
| Candidatus\_Sulfopaludibacter | neg\_994 | 0.878787875175476 | 0.000813862205061078 |
| Kordiimonas | neg\_994 | 0.842424213886261 | 0.00222003275351312 |
| Pararhodospirillum | neg\_994 | 0.866666674613953 | 0.0011735379121256 |
| Comamonas | neg\_994 | -0.866666674613953 | 0.0011735379121256 |
| Candidatus\_Electrothrix | neg\_994 | 0.927272737026215 | 0.000112034447641074 |
| Thermopetrobacter | neg\_994 | 0.842424213886261 | 0.00222003275351312 |
| Rubrivivax | neg\_994 | -0.818181812763214 | 0.00381492051076338 |
| Parafilimonas | neg\_994 | -0.866666674613953 | 0.0011735379121256 |
