# Supplementary material for: Multi-omics analyses reveal that the gut microbiome and its metabolites promote milk fat synthesis in Zhongdian yak cows
Source: PeerJ. 2022 Dec 2;10:e14444. doi: 10.7717/peerj.14444 (PMC9744170; doi:10.7717/peerj.14444)
Supplement: Supplemental Information 13 [file peerj-10-14444-s013.zip › Web_Report/readme.pdf]

BIOMARKER
百迈客生物科技有限公司

结果目录说明

- Web_Report
  - Data_assess 数据评估结果
    - group_info.txt 样本分组信息
    - metabolites_intensity 代谢物定量结果
      - All_metabolite_profiles.xls 所有鉴定代谢物定量文件
      - metabolites_exp.xls 鉴定到代谢物名称的定量文件
      - metabolites_full_table.xls 带有代谢物注释信息的定量文件
    - correlation_analysis 样本相关性结果
      - All_cor.pdf 样本相关性热图
      - All_cor.png 样本相关性热图
      - All_cor.xls 样本相关性文件
    - heatmap 所有样本聚类热图结果
      - All_heatmap.pdf 所有样本聚类热图
      - All_heatmap.png 所有样本聚类热图
      - All_heatmap.xls 所有样本聚类热图作图文件
    - PCA 所有样本主成分分析结果
      - All_pca3D.pdf 所有样本PCA分析三维图
      - All_pca3D.png 所有样本PCA分析三维图
      - All_pca.pdf 所有样本PCA分析二维图
      - All_pca.png 所有样本PCA分析二维图
      - All_pca_loadings.xls 所有样本PCA分析载荷值文件
  - Metabolites_annotation 所有代谢物数据库注释结果
    - KEGG 所有代谢物KEGG数据库注释结果
      - meta_kegg_anno_categroy.xls 所有代谢物KEGG通路统计结果
      - meta_kegg_anno.xls 所有代谢物KEGG注释结果
      - meta_kegg_anno.png 所有代谢物KEGG通路top20柱形图
      - All_kegg_map 所有代谢物通路图注释
        - ko*.html 所有代谢物通路图
        - ko*.png 所有代谢物通路图
    - HMDB 所有代谢物HMDB数据库注释结果
      - meta_hmdb_anno.xls 所有代谢物HMDB数据库注释表格
      - meta_hmdb_anno_categroy.xls 所有代谢物HMDB数据库分类表格
      - meta_hmdb_anno.pdf 所有代谢物HMDB数据库分类top20柱形图
    - Lipidmaps 所有代谢物Lipidmaps数据库注释结果
      - meta_lipidmaps_anno.xls 所有代谢物Lipidmaps数据库注释表格
      - meta_lipidmaps_anno_categroy.xls 所有代谢物Lipidmaps数据库分类结果
      - meta_lipidmaps_anno.png 所有代谢物Lipidmaps数据库分类top20柱形图
  - Diff_analysis 各个分组差异分析结果
    - Diff_KEGG_anno_stat.xls 差异代谢物KEGG注释统计结果
    - *_vs_* 差异分组分析结果
      - *_vs_*.all.xls 未经过筛选的差异分析结果
      - *_vs_*.diff_final.xls 经过筛选的差异分析结果
      - *_vs_*_diff_heatmap.pdf 差异代谢物聚类热图(进行样本聚类)
      - *_vs_*_diff_heatmap.png 差异代谢物聚类热图(进行样本聚类)
      - *_vs_*_diff_heatmap_name.pdf 差异代谢物聚类热图(进行样本聚类且添加代谢物名称)
      - *_vs_*_diff_heatmap_nonclustered_samples.pdf 差异代谢物聚类热图(不进行样本聚类)
      - *_vs_*_diff_heatmap_nonclustered_samples.png 差异代谢物聚类热图(不进行样本聚类)
      - *_vs_*_diff_heatmap_nonclustered_samples_name.pdf 差异代谢物聚类热图(不进行样本聚类且添加代谢物名称)
      - *_vs_*_OPLS_DA.pdf 差异分组OPLS-DA得分图
      - *_vs_*_OPLS_DA_permutation.pdf 差异分组OPLS-DA检验图
      - *_vs_*_OPLS_DA_permutation.png 差异分组OPLS-DA检验图
      - *_vs_*_OPLS_DA.png 差异分组OPLS-DA得分图

```
|       |       |------ *_vs_*_pca3D.pdf 差异分组PCA分析三维图
|       |       |------ *_vs_*_pca3D.png 差异分组PCA分析三维图
|       |       |------ *_vs_*_pca.pdf 差异分组PCA分析二维图
|       |       |------ *_vs_*_pca.png 差异分组PCA分析二维图
|       |       |------ *_vs_*_Top_20_FC_change.pdf 差异分组差异倍数柱图
|       |       |------ *_vs_*_Top_20_FC_change.png 差异分组差异倍数柱图
|       |       |------ *_vs_*.volcano.pdf 差异分组火山图
|       |       |------ *_vs_*.volcano.png 差异分组火山图
|       |       |------ KEGG 差异分组KEGG注释结果
|       |       |       |------ Graph 差异分组KEGG富集结果
|       |       |       |------ kegg_map 差异代谢物通路图注释
|       |       |       |       |------ ko*.html 差异代谢物通路图
|       |       |       |       |------ ko*.png 差异代谢物通路图
|       |------ venn 各分组间的韦恩图
|       |       |------ *_venn.pdf 差异分组间的韦恩图
|       |       |------ *_venn.png 差异分组间的韦恩图
|       |       |------ All_diff_stat.txt 差异代谢物数目统计结果
|       |       |------ Diff_Venn_Intersection.xls 差异代谢物交集统计结果
|       |       |------ Diff_Venn_Union.xls 差异代谢物并集统计结果
|------ readme.pdf 说明文档
```

Copyright © 2009-2021 北京百迈客生物科技有限公司版权所有 京ICP备10042835号

公司地址：北京市顺义区南法信府前街12号顺捷大厦5层

Tel:400-600-3186 Fax:010-57045001 Tel:400-600-3186 E-mail:tech@biomarker.com.cn (mailto:tech@biomarker.com.cn)

微信:biomarker_tech 百迈客生物云平台 (https://www.biocloud.net/external/login/toLogin)

关于我们 (http://www.biomarker.com.cn)
