# Supplementary material for: Multi-omics analyses reveal that the gut microbiome and its metabolites promote milk fat synthesis in Zhongdian yak cows
Source: PeerJ. 2022 Dec 2;10:e14444. doi: 10.7717/peerj.14444 (PMC9744170; doi:10.7717/peerj.14444)
Supplement: Supplemental Information 13 [file peerj-10-14444-s013.zip › Web_Report/Quality_control/QC.html]

BMK211021-AP383-ZX01-0201|西南林业大学10个宏基因组12个代谢组建库测序分析

BMK211021-AP383-ZX01-0201|西南林业大学10个宏基因组12个代谢组建库测序分析

## BMK211021-AP383-ZX01-0201|西南林业大学10个宏基因组12个代谢组建库测序分析

### Abstract

本页面展示项目结果质控（QC）信息。第一部分是所有质控指标列表，第二部分是每个指标的详细说明和结果展示。

项目信息概况：本项目对12 个样品中的2,400 个代谢物进行了分析。

### 1 所有质控指标列表

表达定量指标是指基于表达量结果结合相关性分析检查生物学重复，一般来说生物学重复样本间具有较高的相关性，具体统计结果如下：

表1 表达定量指标

| 指标名称 | 评价标准 | 是否达标 |
| --- | --- | --- |
| 组内相关性最小值 | ≥0.5 | yes |

注：组内相关性最小值：计算不同样品间的表达量的皮尔逊相关系数或斯皮尔曼相关系数，同一个生物学重复组内样品的相关性>=0.7时，认为重复性较好。

差异结果指标是对差异分组中的差异代谢物数目的统计，差异数目的多少将影响后续的研究，具体统计结果如下：

表2 差异结果指标

| 指标名称 | 评价标准 | 是否达标 |
| --- | --- | --- |
| 差异数目最大值 | ≥10 | yes |

注：差异数目最大值：不同差异分组中差异代谢物数量的最大值。

下面列出一些常见的指标异常的情况、原因和处理方法。

常见问题

- 1.组内相关性指标异常可能的原因

  答：1.若数据存在缺失值，可能是缺失值填补的问题，可尝试其他缺失填补方法；2.可能是因为样本本身重复性不好

- 2.差异结果指标异常可能的原因

  答：1.可能是差异分组中组间样本相关性太高；2.可尝试调整差异分析参数

### 2 每个指标的详细说明和结果展示

#### 2.1 过程质控

样品的检测要持续很长时间，尤其是当样本量很大的时候。在检测过程中实时地监控仪器稳定性、信号是否正常就十分重要。及时发现异常，尽早将问题排除，以保证最终采集数据的质量。由图1可以看到，QC样本BPI出峰保留时间和峰面积都重叠很好，说明仪器稳定性很好。

图1 QC样本BPI重叠图

#### 2.2 样本TIC图

根据标准品的保留时间与峰型的信息，对每种物质在不同样本中检测到的质谱峰进行校正，以确保定性定量的准确。各样本的TIC图如下文件：

- tic-total-0
- tic-total-1
- tic-total-2
- tic-total-3
- tic-total-4

图2 样本TIC图

#### 2.3 表达定量指标

表3 样品相关性结果

| SampleID | H1 | H2 | H3 | H4 | H5 | H6 | L2 | L6 | L1 | L4 | L5 | L3 |
| --- | --- | --- | --- | --- | --- | --- | --- | --- | --- | --- | --- | --- |
| H1 | 1 | 0.87 | 0.78 | 0.79 | 0.87 | 0.91 | 0.83 | 0.82 | 0.8 | 0.86 | 0.85 | 0.77 |
| H2 | 0.87 | 1 | 0.83 | 0.84 | 0.85 | 0.89 | 0.82 | 0.83 | 0.85 | 0.88 | 0.87 | 0.78 |
| H3 | 0.78 | 0.83 | 1 | 0.85 | 0.82 | 0.89 | 0.82 | 0.91 | 0.87 | 0.86 | 0.91 | 0.89 |
| H4 | 0.79 | 0.84 | 0.85 | 1 | 0.82 | 0.89 | 0.84 | 0.87 | 0.84 | 0.83 | 0.89 | 0.82 |
| H5 | 0.87 | 0.85 | 0.82 | 0.82 | 1 | 0.92 | 0.87 | 0.86 | 0.86 | 0.87 | 0.89 | 0.8 |
| H6 | 0.91 | 0.89 | 0.89 | 0.89 | 0.92 | 1 | 0.88 | 0.91 | 0.88 | 0.91 | 0.94 | 0.86 |
| L2 | 0.83 | 0.82 | 0.82 | 0.84 | 0.87 | 0.88 | 1 | 0.92 | 0.86 | 0.84 | 0.91 | 0.8 |
| L6 | 0.82 | 0.83 | 0.91 | 0.87 | 0.86 | 0.91 | 0.92 | 1 | 0.89 | 0.86 | 0.97 | 0.91 |
| L1 | 0.8 | 0.85 | 0.87 | 0.84 | 0.86 | 0.88 | 0.86 | 0.89 | 1 | 0.83 | 0.91 | 0.84 |
| L4 | 0.86 | 0.88 | 0.86 | 0.83 | 0.87 | 0.91 | 0.84 | 0.86 | 0.83 | 1 | 0.91 | 0.81 |
| L5 | 0.85 | 0.87 | 0.91 | 0.89 | 0.89 | 0.94 | 0.91 | 0.97 | 0.91 | 0.91 | 1 | 0.9 |
| L3 | 0.77 | 0.78 | 0.89 | 0.82 | 0.8 | 0.86 | 0.8 | 0.91 | 0.84 | 0.81 | 0.9 | 1 |

样品相关性结果的完整表格如下：

All\_cor.xls

图3 样品相关性图

#### 2.4 差异结果指标

表4 各分组差异结果

| group name | All diff | down-regulated | up-regulated |
| --- | --- | --- | --- |
| H\_vs\_L | 106 | 49 | 57 |

各分组差异结果的完整表格如下：

diff\_result.detail.xls

- Abstract
- 1 所有质控指标列表
- 2 每个指标的详细说明和结果展示 
  - 2.1 过程质控
  - 2.2 样本TIC图
  - 2.3 表达定量指标
  - 2.4 差异结果指标

Copyright © 2009-2021 北京百迈客生物科技有限公司版权所有 京ICP备10042835号

公司地址：北京市顺义区南法信府前街12号顺捷大厦5层

- Tel:400-600-3186
- Fax:010-57045001
- E-mail:tech@biomarker.com.cn
- 微信:biomarker\_tech
- 百迈客生物云平台
- 关于我们
