# Supplementary material for: Multi-omics analyses reveal that the gut microbiome and its metabolites promote milk fat synthesis in Zhongdian yak cows
Source: PeerJ. 2022 Dec 2;10:e14444. doi: 10.7717/peerj.14444 (PMC9744170; doi:10.7717/peerj.14444)
Supplement: Supplemental Information 13 [file peerj-10-14444-s013.zip › Web_Report/Quality_control/qc_data/tic-total.pdf]

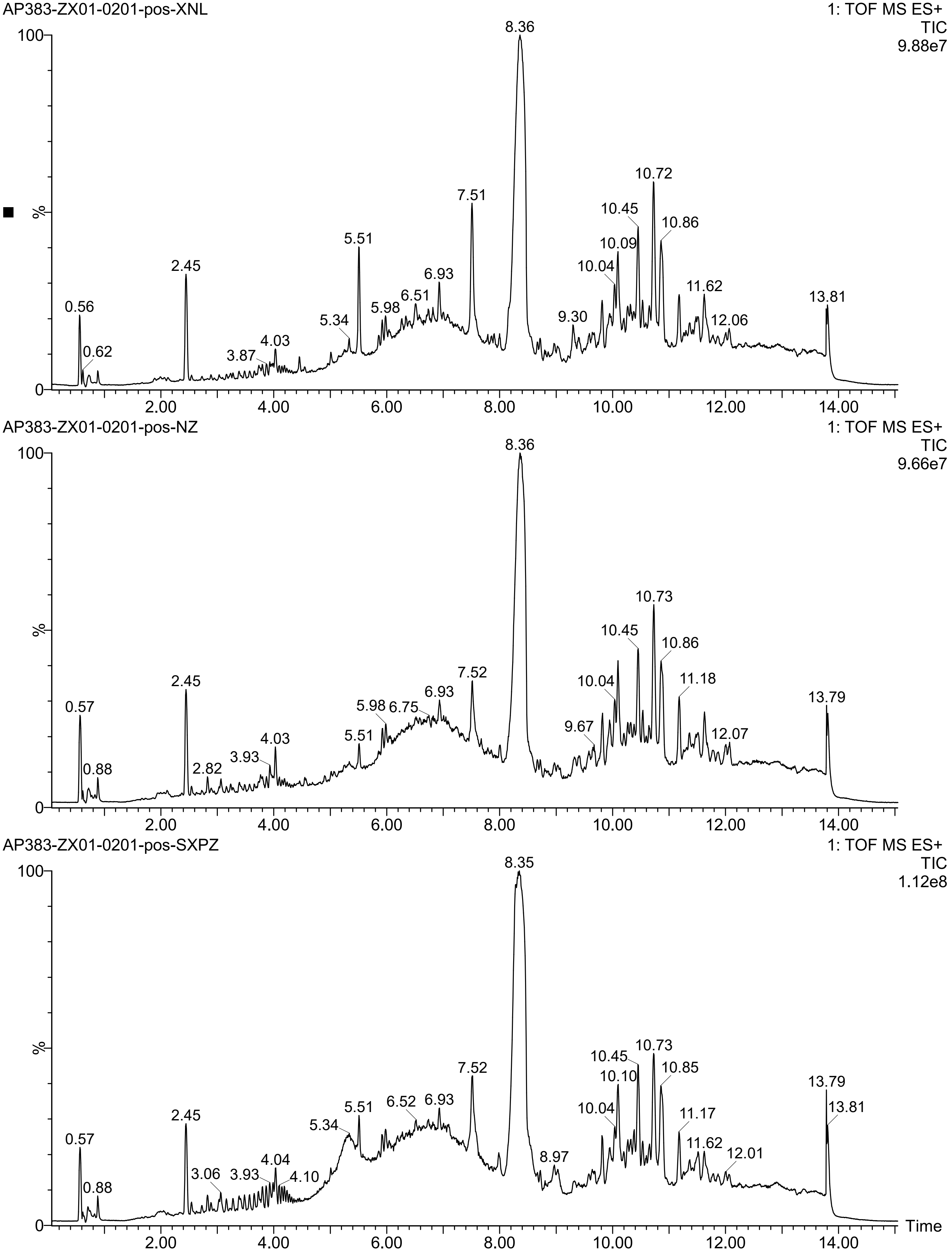
AP383-ZX01-0201-pos-XNL
1: TOF MS ES+
TIC
9.88e7
8.36
7.51
10.72
10.45
10.86
10.09
5.51
10.04
2.45
6.93
0.56
11.62
13.81
5.98
6.51
9.30
5.34
12.06
0.62
4.03
3.87
AP383-ZX01-0201-pos-NZ
1: TOF MS ES+
TIC
9.66e7
8.36
10.73
10.45
10.86
11.18
2.45
7.52
10.04
6.93
0.57
5.98
6.75
13.79
9.67
12.07
4.03
5.51
3.93
0.88
2.82
AP383-ZX01-0201-pos-SXPZ
1: TOF MS ES+
TIC
1.12e8
8.35
10.45
10.73
10.10
10.85
7.52
13.79
6.52
6.93
5.51
13.81
2.45
10.04
11.17
5.34
0.57
11.62
8.97
12.01
4.04
3.06
3.93
4.10
0.88
100
%
0
2.00
4.00
6.00
8.00
10.00
12.00
14.00
Time

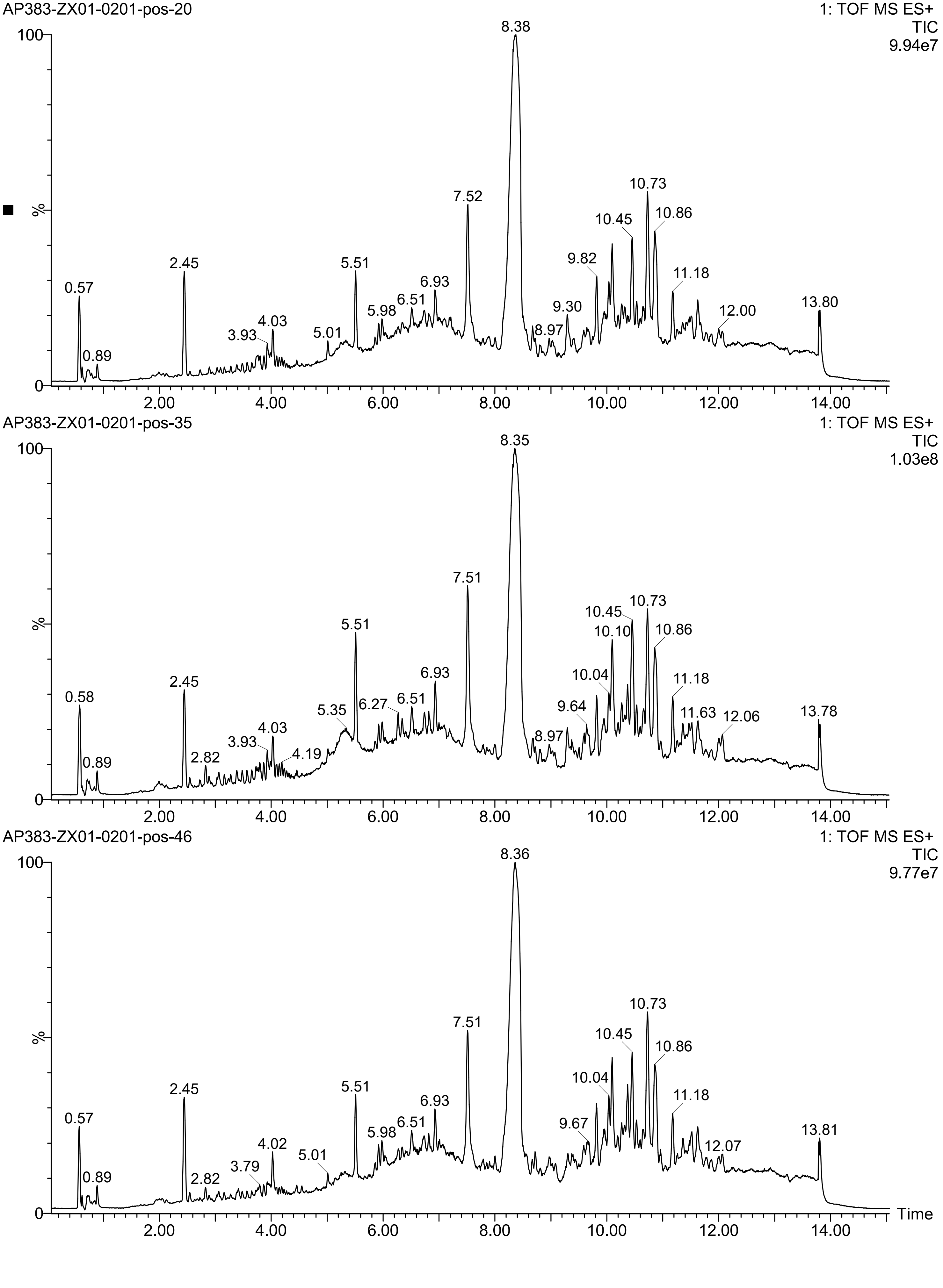

AP383-ZX01-0201-pos-20
1: TOF MS ES+
TIC
9.94e7
100
%
0
8.38
7.52
10.73
10.45
10.86
2.45
5.51
9.82
0.57
6.93
11.18
6.51
5.98
9.30
12.00
13.80
4.03
3.93
5.01
8.97
0.89
2.00
4.00
6.00
8.00
10.00
12.00
14.00
AP383-ZX01-0201-pos-35
1: TOF MS ES+
TIC
1.03e8
8.35
7.51
10.73
10.45
10.10
10.86
5.51
6.93
2.45
10.04
11.18
0.58
6.27
6.51
9.64
5.35
11.63
12.06
13.78
4.03
3.93
8.97
4.19
2.82
0.89
AP383-ZX01-0201-pos-46
1: TOF MS ES+
TIC
9.77e7
8.36
10.73
7.51
10.45
10.86
10.04
2.45
5.51
6.93
11.18
0.57
5.98
6.51
9.67
13.81
4.02
5.01
12.07
3.79
2.82
0.89
Time

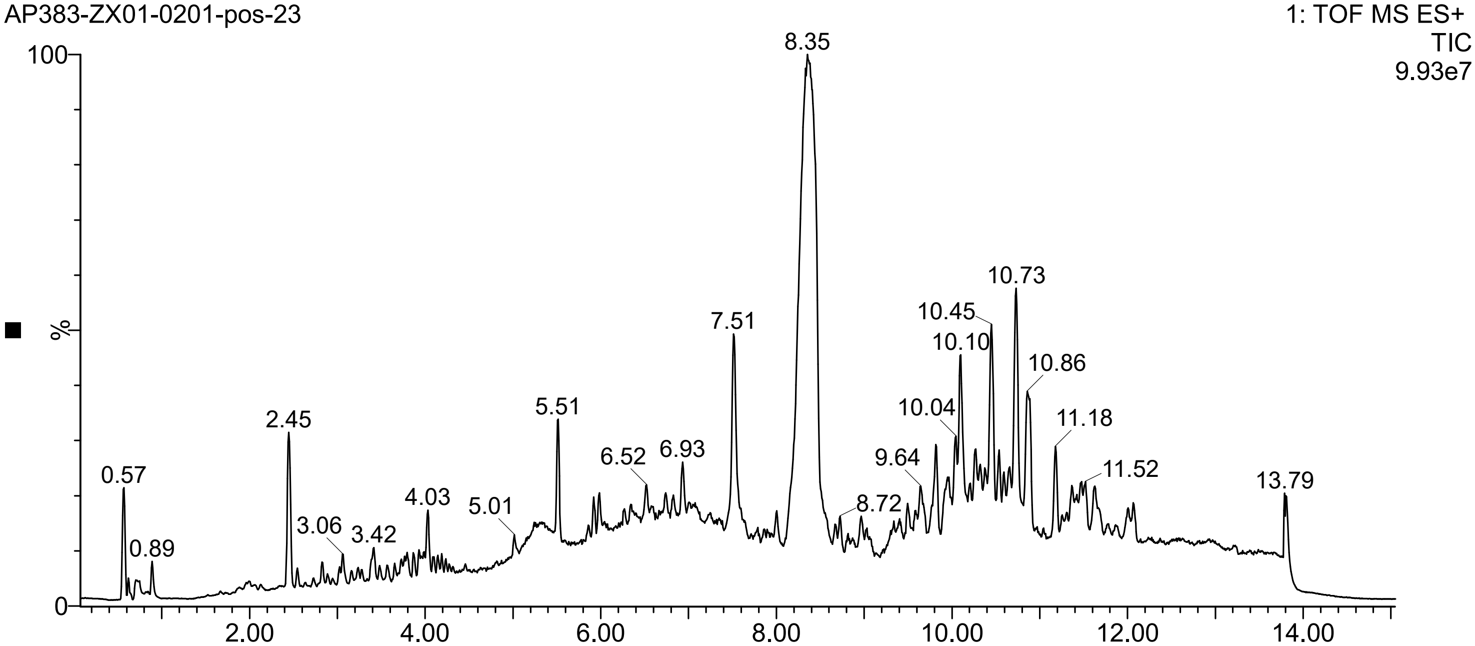
AP383-ZX01-0201-pos-23
1: TOF MS ES+
TIC
9.93e7
8.35
7.51
10.73
10.45
10.10
10.86
10.04
11.18
11.52
9.64
8.72
13.79
6.52
6.93
5.51
5.01
4.03
3.06
3.42
2.45
0.57
0.89
100
%
0
2.00
4.00
6.00
8.00
10.00
12.00
14.00

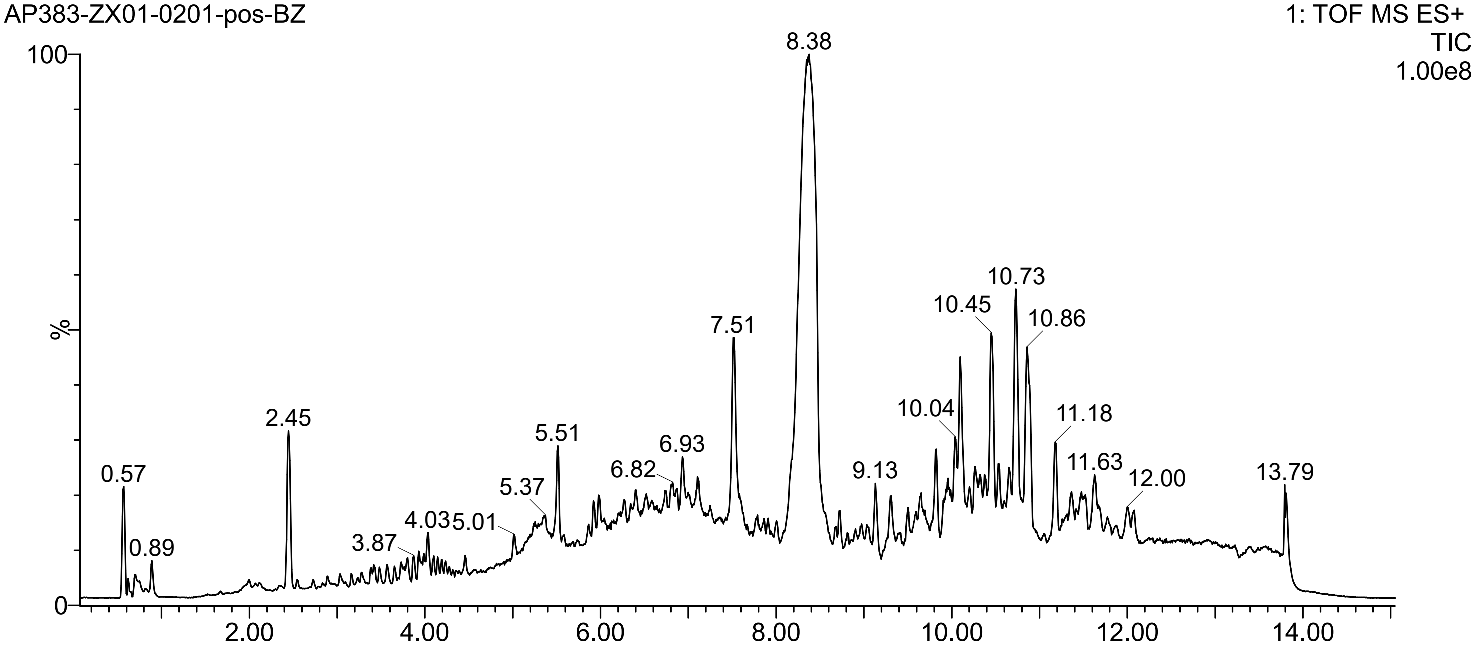
AP383-ZX01-0201-pos-BZ
1: TOF MS ES+
TIC
1.00e8
8.38
7.51
10.73
10.45
10.86
10.04
11.18
11.63
12.00
9.13
13.79
6.82
6.93
5.51
5.37
4.035.01
3.87
2.45
0.57
0.89
100
%
0
2.00
4.00
6.00
8.00
10.00
12.00
14.00

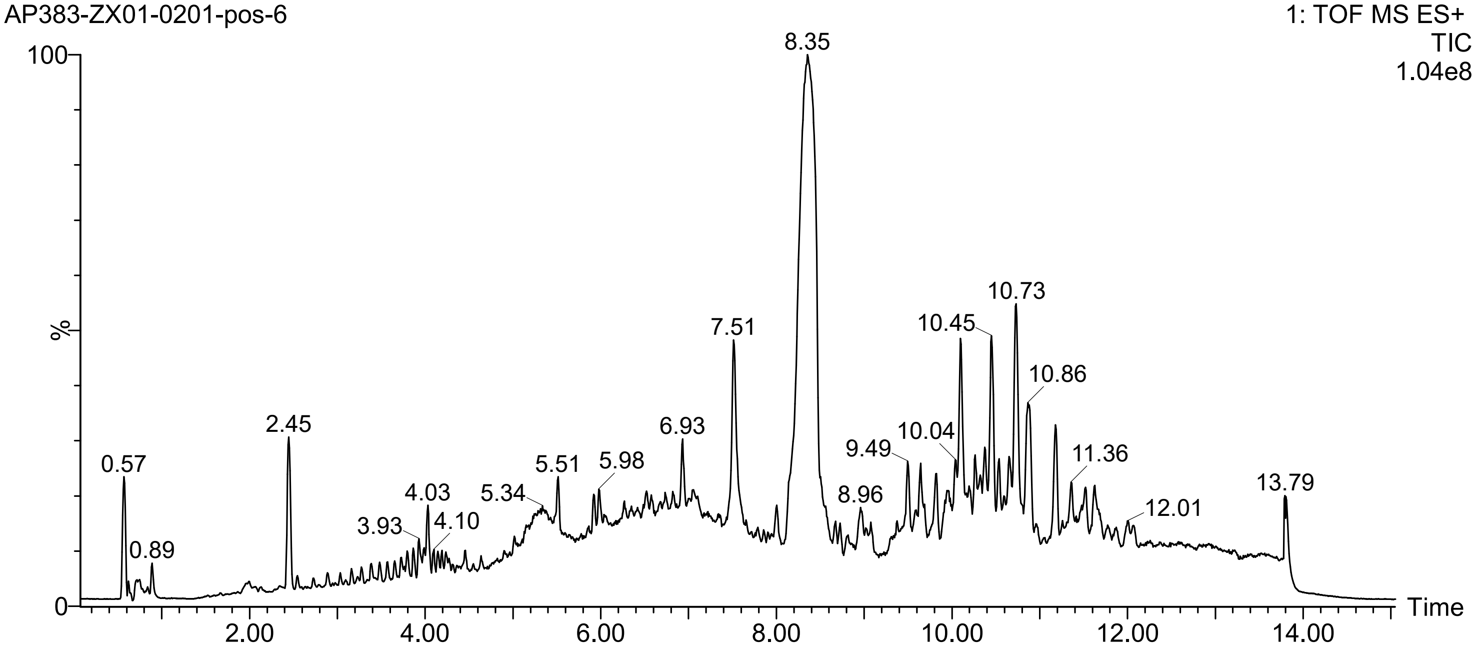
AP383-ZX01-0201-pos-6
1: TOF MS ES+
TIC
1.04e8
8.35
7.51
10.73
10.45
10.86
10.04
9.49
11.36
12.01
8.96
13.79
6.93
5.98
5.51
5.34
4.03
4.10
3.93
2.45
0.57
0.89
100
%
0
2.00
4.00
6.00
8.00
10.00
12.00
14.00
Time

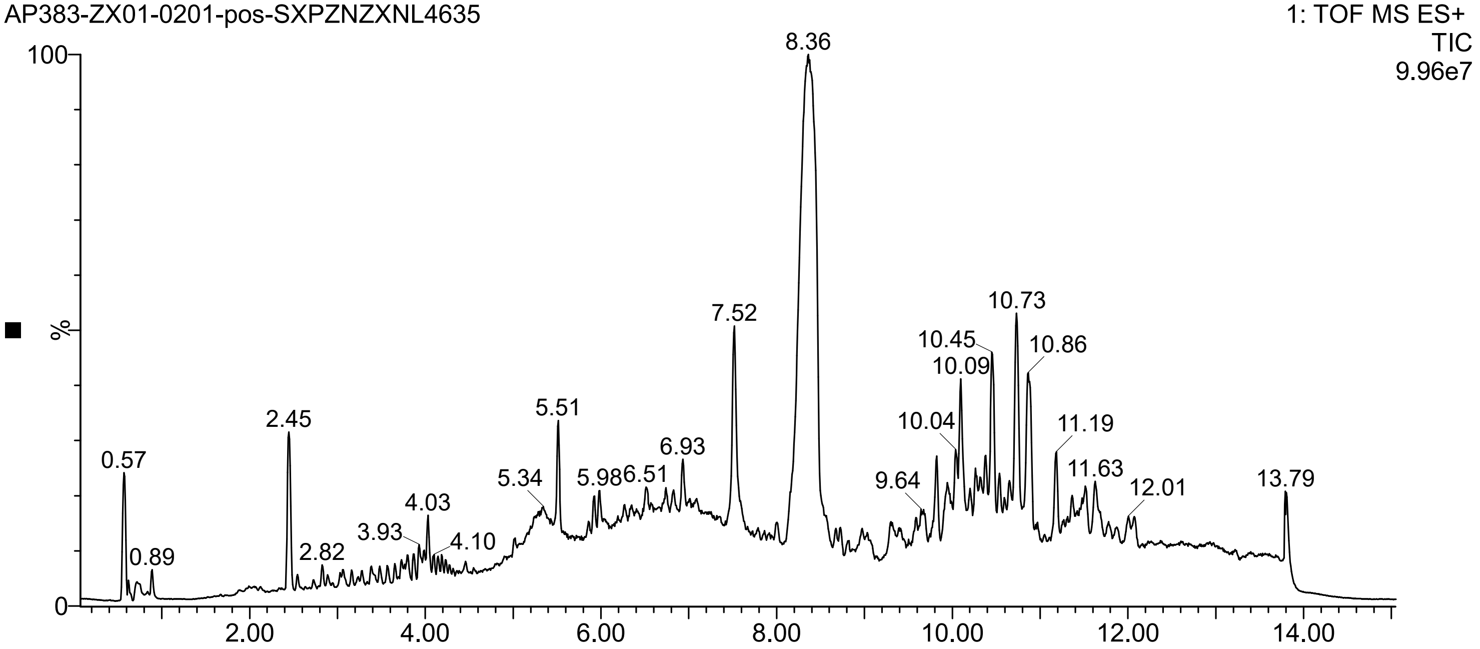
AP383-ZX01-0201-pos-SXPZNZXNL4635
1: TOF MS ES+
TIC
9.96e7
100
%
0
8.36
7.52
10.73
10.45
10.86
10.09
10.04
11.19
11.63
12.01
13.79
9.64
6.93
5.98
6.51
5.51
5.34
4.03
3.93
4.10
2.45
2.82
0.57
0.89
2.00
4.00
6.00
8.00
10.00
12.00
14.00

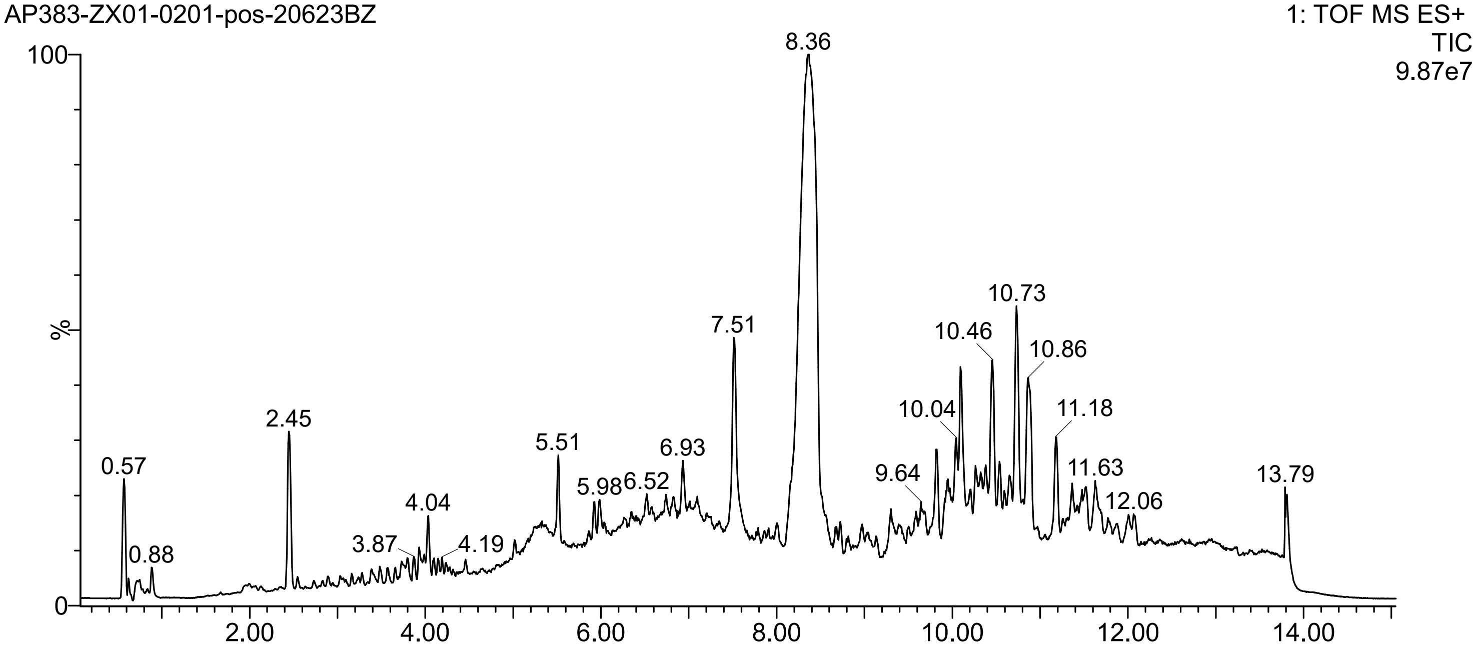
AP383-ZX01-0201-pos-20623BZ
1: TOF MS ES+
TIC
9.87e7
100
%
0
8.36
7.51
10.73
10.46
10.86
10.04
11.18
11.63
12.06
13.79
9.64
6.93
5.98
6.52
5.51
4.04
3.87
4.19
2.45
0.57
0.88
2.00
4.00
6.00
8.00
10.00
12.00
14.00

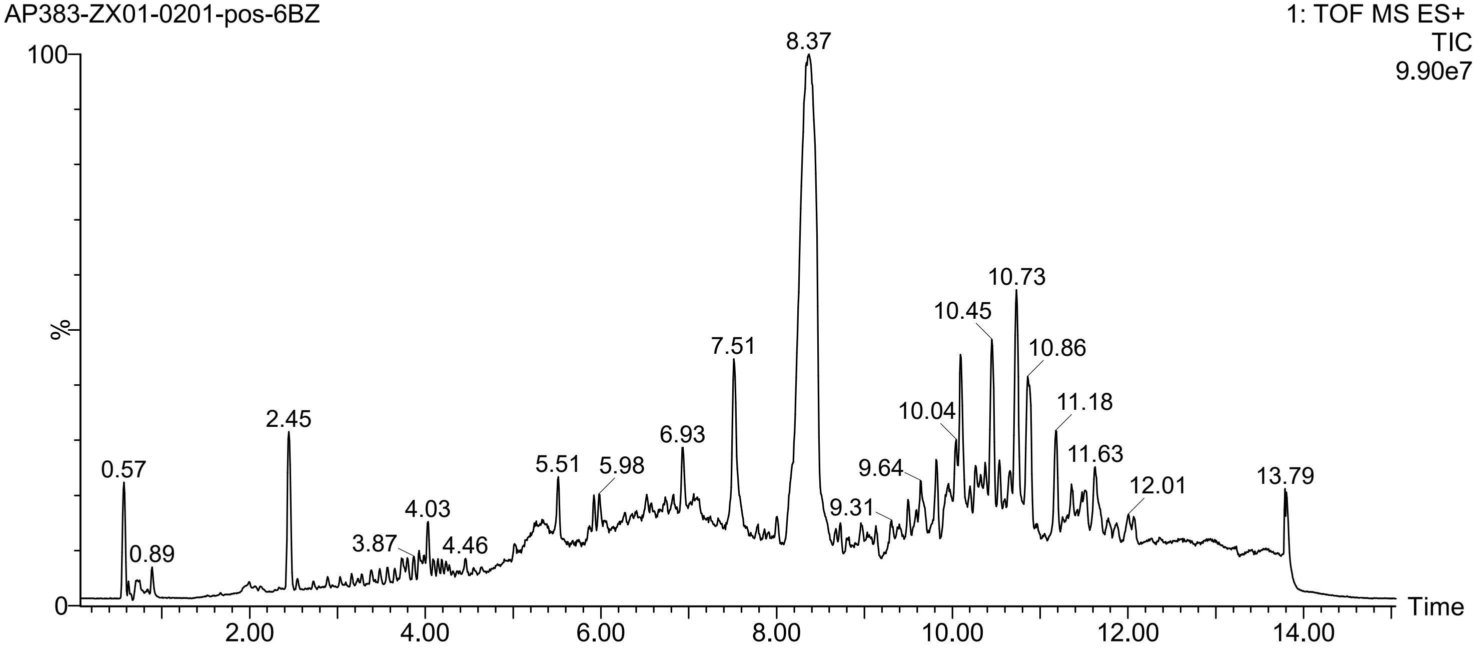
AP383-ZX01-0201-pos-6BZ
1: TOF MS ES+
TIC
9.90e7
100
%
0
8.37
10.73
10.45
10.86
7.51
11.18
10.04
11.63
12.01
13.79
9.64
9.31
6.93
5.51
5.98
4.03
3.87
4.46
2.45
0.57
0.89
Time
2.00
4.00
6.00
8.00
10.00
12.00
14.00

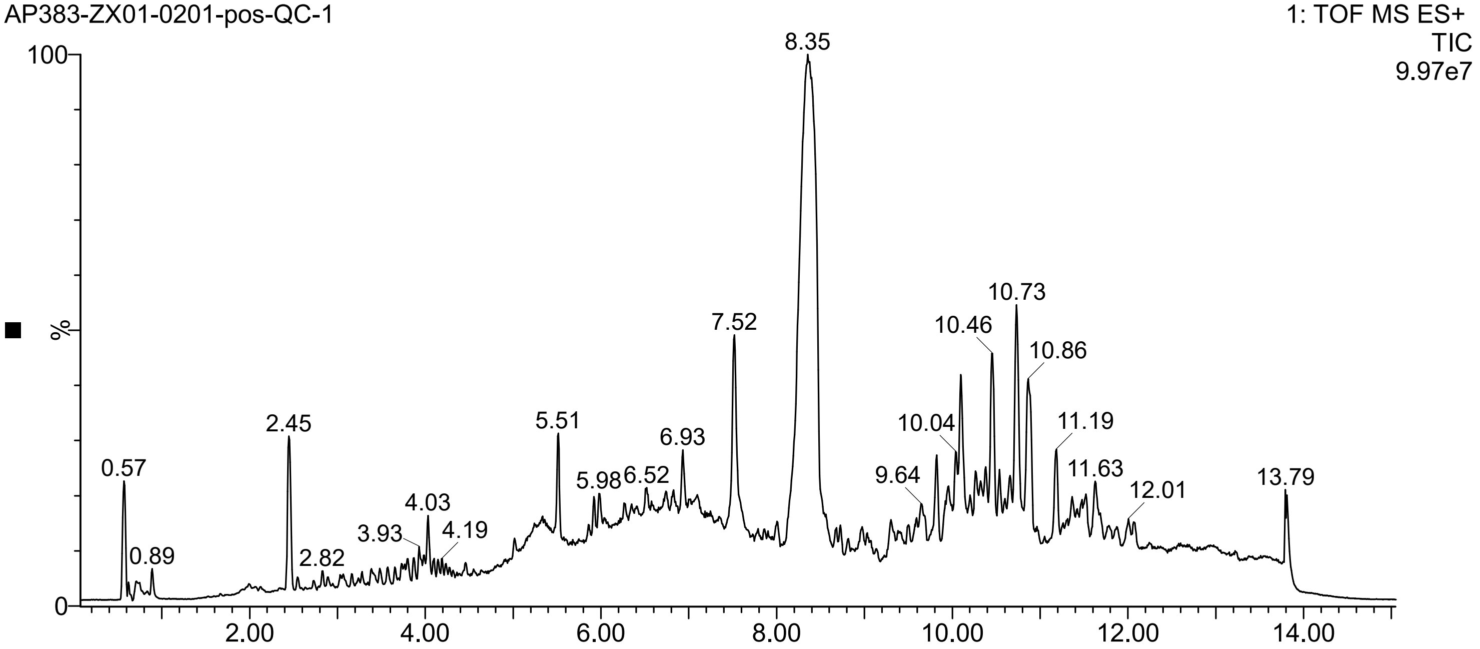
AP383-ZX01-0201-pos-QC-1
1: TOF MS ES+
TIC
9.97e7
100
%
0
8.35
7.52
10.73
10.46
10.86
10.04
11.19
2.45
5.51
6.93
0.57
5.98
6.52
9.64
11.63
12.01
13.79
4.03
3.93
4.19
0.89
2.82
2.00
4.00
6.00
8.00
10.00
12.00
14.00

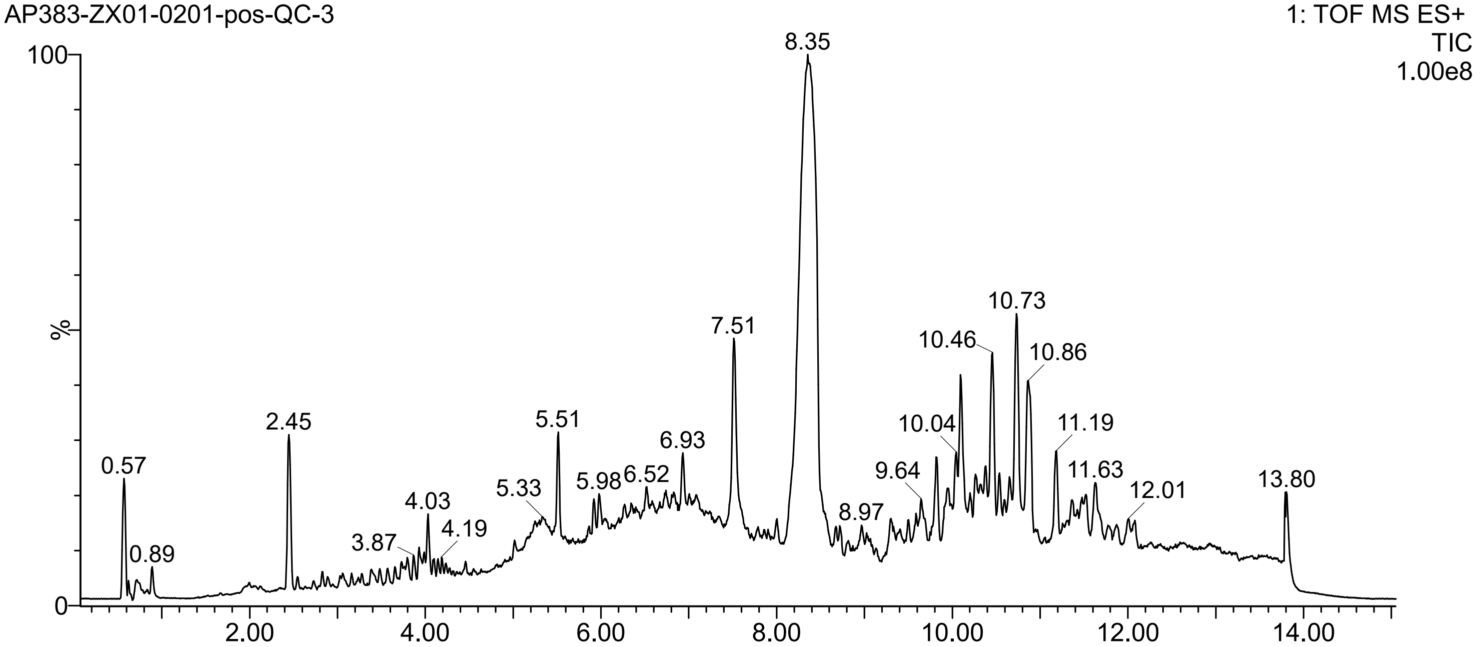
AP383-ZX01-0201-pos-QC-3
1: TOF MS ES+
TIC
1.00e8
100
%
0
8.35
7.51
10.73
10.46
10.86
10.04
11.19
2.45
5.51
6.93
0.57
5.33
5.98
6.52
9.64
8.97
11.63
12.01
13.80
4.03
3.87
4.19
0.89
2.00
4.00
6.00
8.00
10.00
12.00
14.00

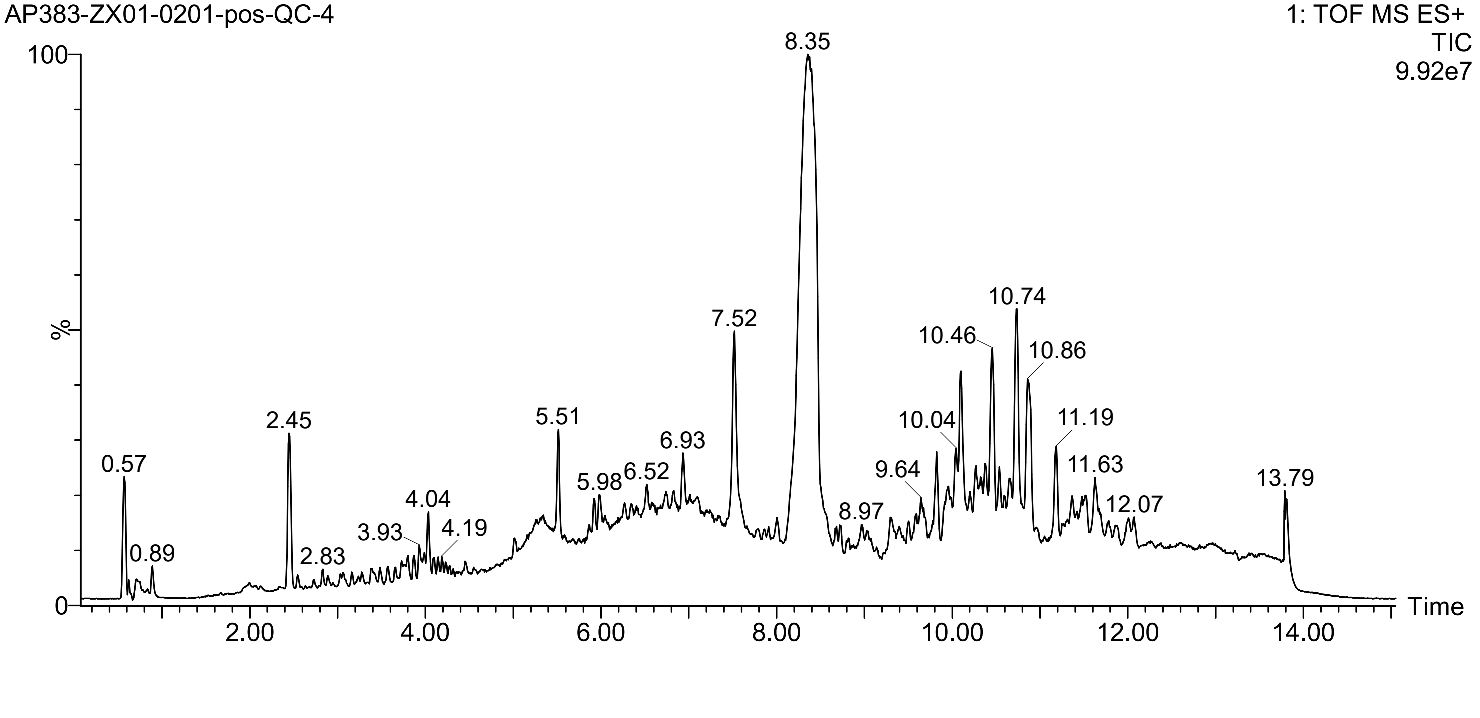
AP383-ZX01-0201-pos-QC-4
1: TOF MS ES+
TIC
9.92e7
100
%
0
8.35
7.52
10.74
10.46
10.86
10.04
11.19
2.45
5.51
6.93
0.57
5.98
6.52
9.64
8.97
11.63
12.07
13.79
4.04
3.93
4.19
0.89
2.83
Time
2.00
4.00
6.00
8.00
10.00
12.00
14.00
