# Supplementary material for: Multi-omics analyses reveal that the gut microbiome and its metabolites promote milk fat synthesis in Zhongdian yak cows
Source: PeerJ. 2022 Dec 2;10:e14444. doi: 10.7717/peerj.14444 (PMC9744170; doi:10.7717/peerj.14444)
Supplement: Supplemental Information 13 [file peerj-10-14444-s013.zip › Web_Report/Quality_control/HTML_qc/All_cor.xls.html.cloud]

All\_cor.xls


## All\_cor.xls

| SampleID | H1 | H2 | H3 | H4 | H5 | H6 | L2 | L6 | L1 | L4 | L5 | L3 |
| --- | --- | --- | --- | --- | --- | --- | --- | --- | --- | --- | --- | --- |
| H1 | 1 | 0.865290535550878 | 0.781374907091865 | 0.785834145065881 | 0.869413019924784 | 0.911079380601281 | 0.828568683087045 | 0.822203718233743 | 0.80225156630741 | 0.857911384653624 | 0.848464438823915 | 0.768902275725177 |
| H2 | 0.865290535550878 | 1 | 0.829589557714205 | 0.83684226443238 | 0.852485887677063 | 0.894301375574025 | 0.821713784491348 | 0.828733004911613 | 0.850564580539212 | 0.87580721958771 | 0.865514194029591 | 0.778022280277591 |
| H3 | 0.781374907091865 | 0.829589557714205 | 1 | 0.850474005457387 | 0.817344468155385 | 0.894116733929285 | 0.820323619803731 | 0.907773900100813 | 0.87252311768844 | 0.858298382364411 | 0.913107509812553 | 0.894006640632846 |
| H4 | 0.785834145065881 | 0.83684226443238 | 0.850474005457387 | 1 | 0.82398623904645 | 0.889451313204254 | 0.835072474244982 | 0.867027144524929 | 0.843679572630069 | 0.826579594503713 | 0.885712901501776 | 0.816634148323873 |
| H5 | 0.869413019924784 | 0.852485887677063 | 0.817344468155385 | 0.82398623904645 | 1 | 0.917460690040942 | 0.868919202585806 | 0.864346846315106 | 0.855919909212813 | 0.872194128088791 | 0.888699095647381 | 0.801304795859627 |
| H6 | 0.911079380601281 | 0.894301375574025 | 0.894116733929285 | 0.889451313204254 | 0.917460690040942 | 1 | 0.875095162988298 | 0.910728932945653 | 0.882940179434087 | 0.913053555088694 | 0.939089082094084 | 0.856804057606365 |
| L2 | 0.828568683087045 | 0.821713784491348 | 0.820323619803731 | 0.835072474244982 | 0.868919202585806 | 0.875095162988298 | 1 | 0.921300197546377 | 0.863901053849779 | 0.843733831172232 | 0.912934575982577 | 0.796202203056524 |
| L6 | 0.822203718233743 | 0.828733004911613 | 0.907773900100813 | 0.867027144524929 | 0.864346846315106 | 0.910728932945653 | 0.921300197546377 | 1 | 0.886372340713268 | 0.857415822981174 | 0.96736606134076 | 0.911358398070997 |
| L1 | 0.80225156630741 | 0.850564580539212 | 0.87252311768844 | 0.843679572630069 | 0.855919909212813 | 0.882940179434087 | 0.863901053849779 | 0.886372340713268 | 1 | 0.831234581070535 | 0.910997922407981 | 0.835859030865826 |
| L4 | 0.857911384653624 | 0.87580721958771 | 0.858298382364411 | 0.826579594503713 | 0.872194128088791 | 0.913053555088694 | 0.843733831172232 | 0.857415822981174 | 0.831234581070535 | 1 | 0.906343444828126 | 0.811287576641922 |
| L5 | 0.848464438823915 | 0.865514194029591 | 0.913107509812553 | 0.885712901501776 | 0.888699095647381 | 0.939089082094084 | 0.912934575982577 | 0.96736606134076 | 0.910997922407981 | 0.906343444828126 | 1 | 0.904760909692788 |
| L3 | 0.768902275725177 | 0.778022280277591 | 0.894006640632846 | 0.816634148323873 | 0.801304795859627 | 0.856804057606365 | 0.796202203056524 | 0.911358398070997 | 0.835859030865826 | 0.811287576641922 | 0.904760909692788 | 1 |
