# Supplementary material for: Multi-omics analyses reveal that the gut microbiome and its metabolites promote milk fat synthesis in Zhongdian yak cows
Source: PeerJ. 2022 Dec 2;10:e14444. doi: 10.7717/peerj.14444 (PMC9744170; doi:10.7717/peerj.14444)
Supplement: Supplemental Information 13 [file peerj-10-14444-s013.zip › Web_Report/Quality_control/HTML_qc/diff_result.detail.xls.html]

diff\_result.detail.xls


## diff\_result.detail.xls

| group name | All diff | down-regulated | up-regulated |
| --- | --- | --- | --- |
| H\_vs\_L | 106 | 49 | 57 |
