# Supplementary figures and images for: Multi-omics analyses reveal that the gut microbiome and its metabolites promote milk fat synthesis in Zhongdian yak cows (part 2 of 2)
Source: PeerJ. 2022 Dec 2;10:e14444. doi: 10.7717/peerj.14444 (PMC9744170; doi:10.7717/peerj.14444)

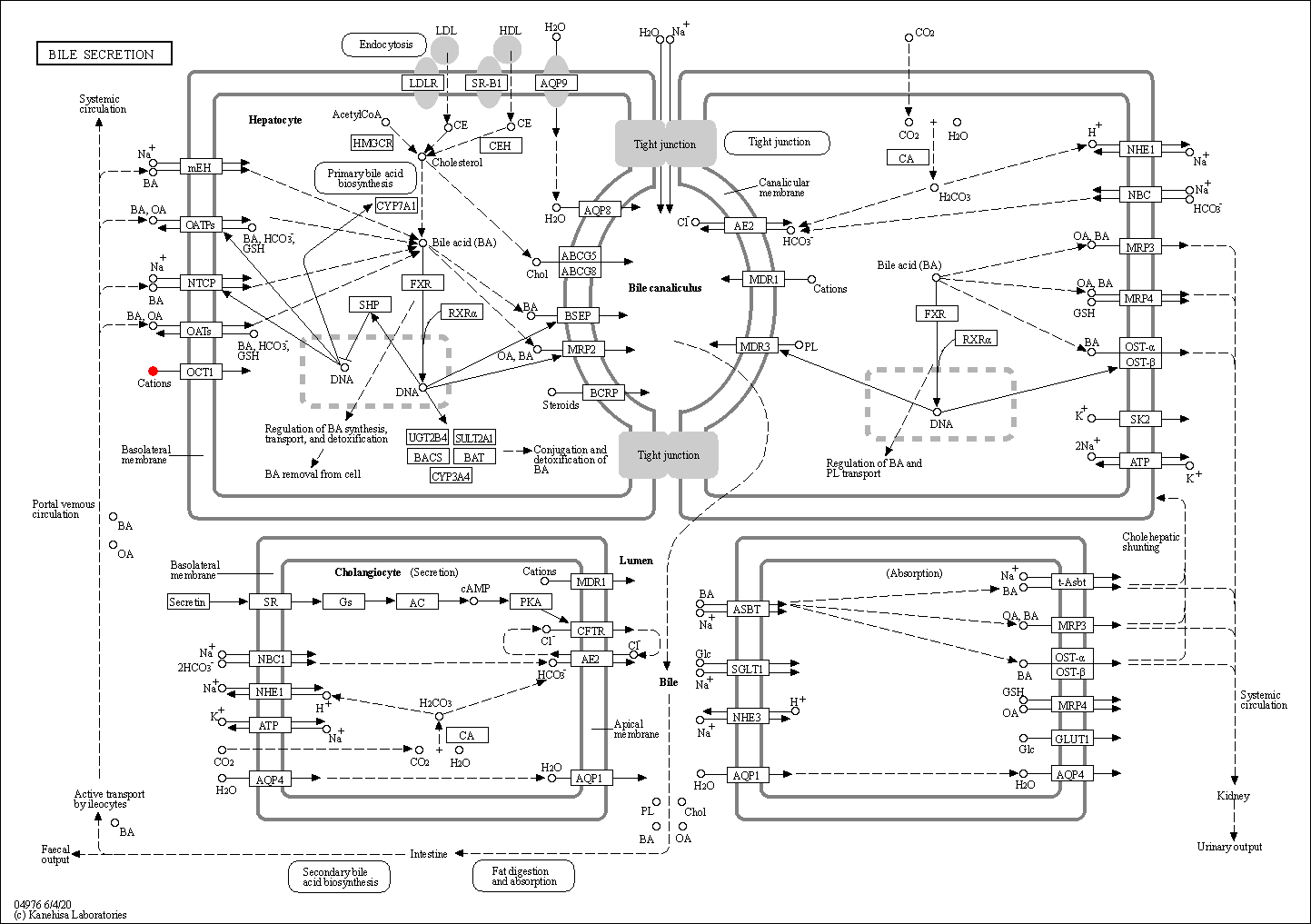

Supplement: Supplemental Information 13 [file peerj-10-14444-s013.zip › Web_Report/Diff_analysis/H_vs_L/KEGG/kegg_map/ko04976.png]

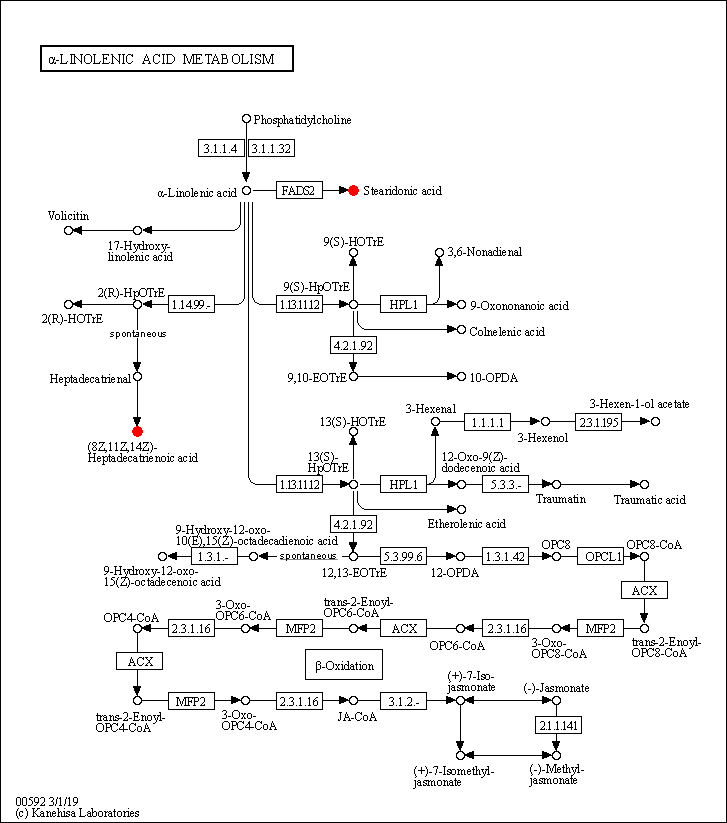

Supplement: Supplemental Information 13 [file peerj-10-14444-s013.zip › Web_Report/Diff_analysis/H_vs_L/KEGG/kegg_map/ko00592.png]

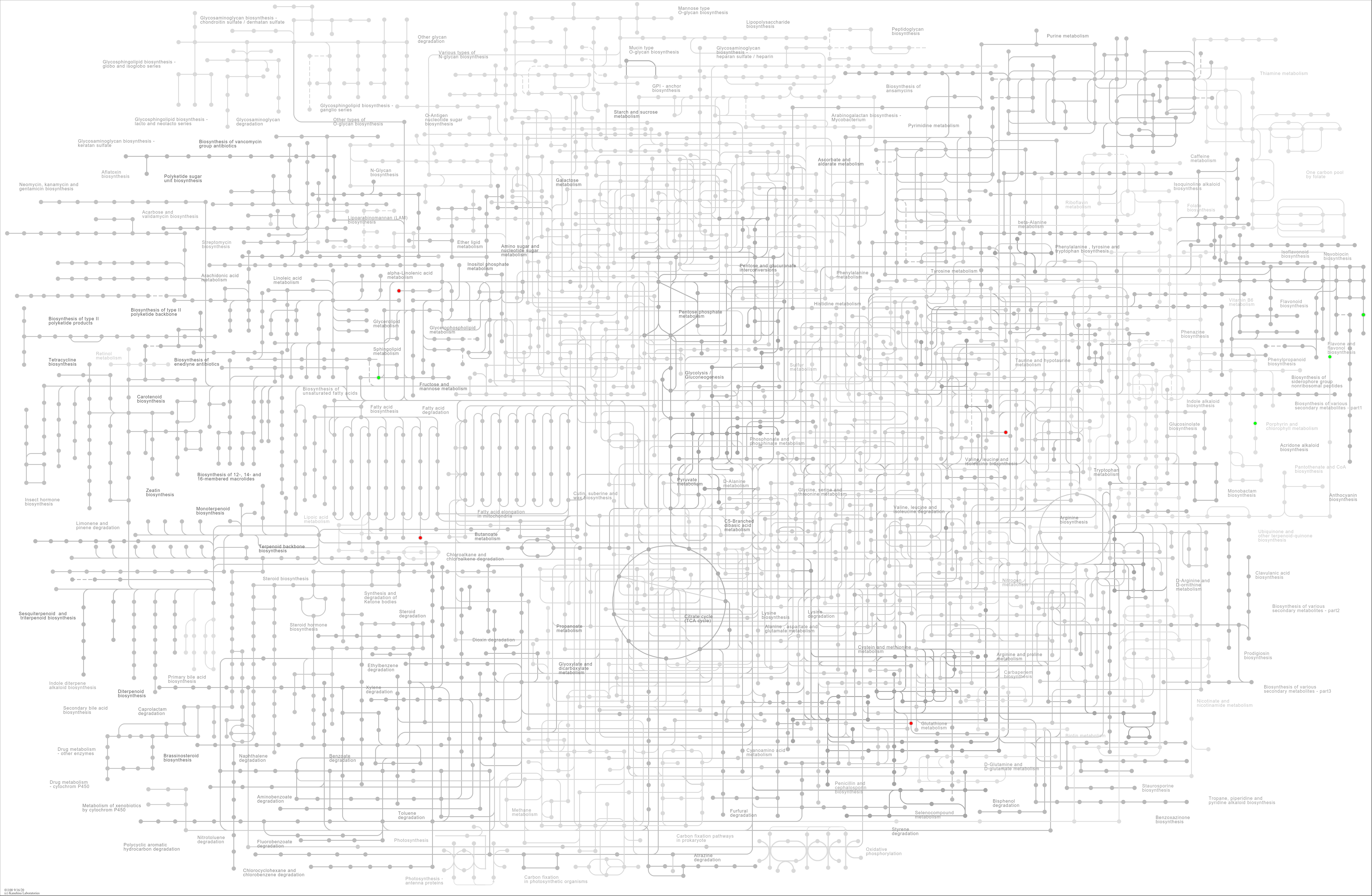

Supplement: Supplemental Information 13 [file peerj-10-14444-s013.zip › Web_Report/Diff_analysis/H_vs_L/KEGG/kegg_map/ko01100.png]

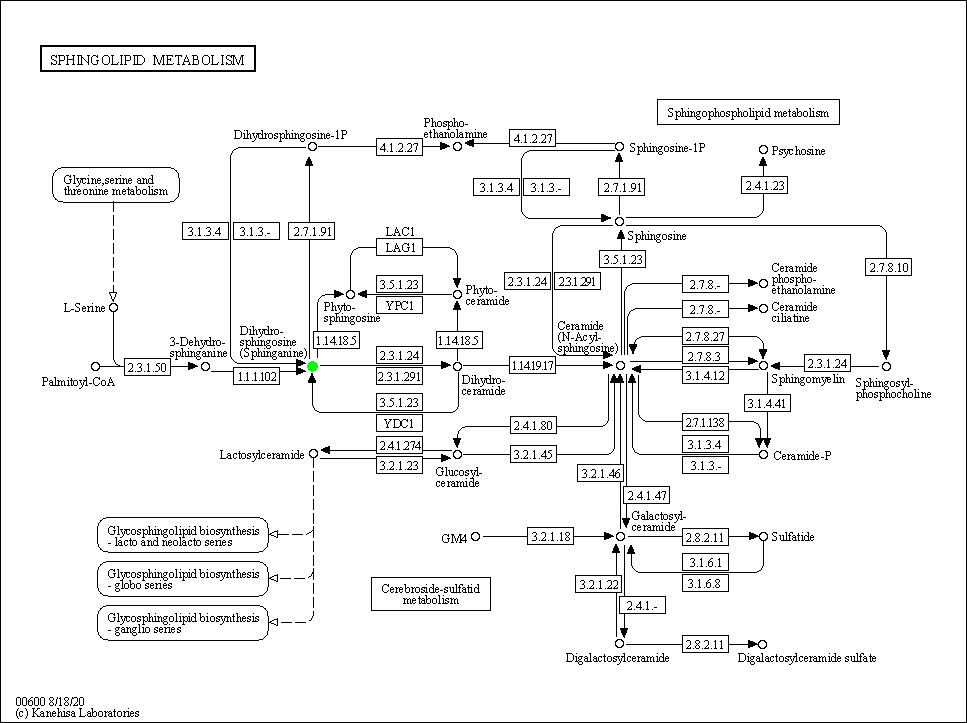

Supplement: Supplemental Information 13 [file peerj-10-14444-s013.zip › Web_Report/Diff_analysis/H_vs_L/KEGG/kegg_map/ko00600.png]

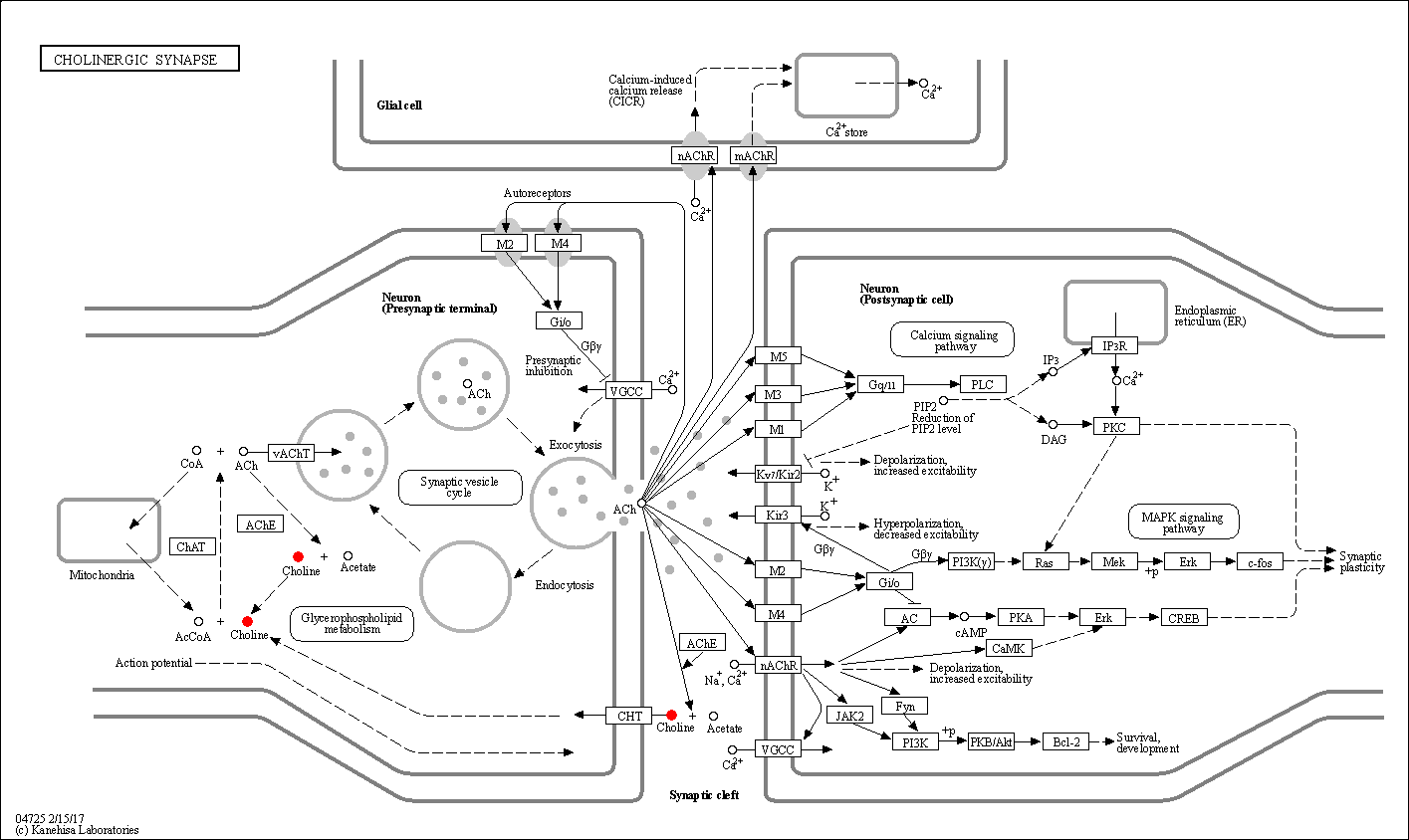

Supplement: Supplemental Information 13 [file peerj-10-14444-s013.zip › Web_Report/Diff_analysis/H_vs_L/KEGG/kegg_map/ko04725.png]

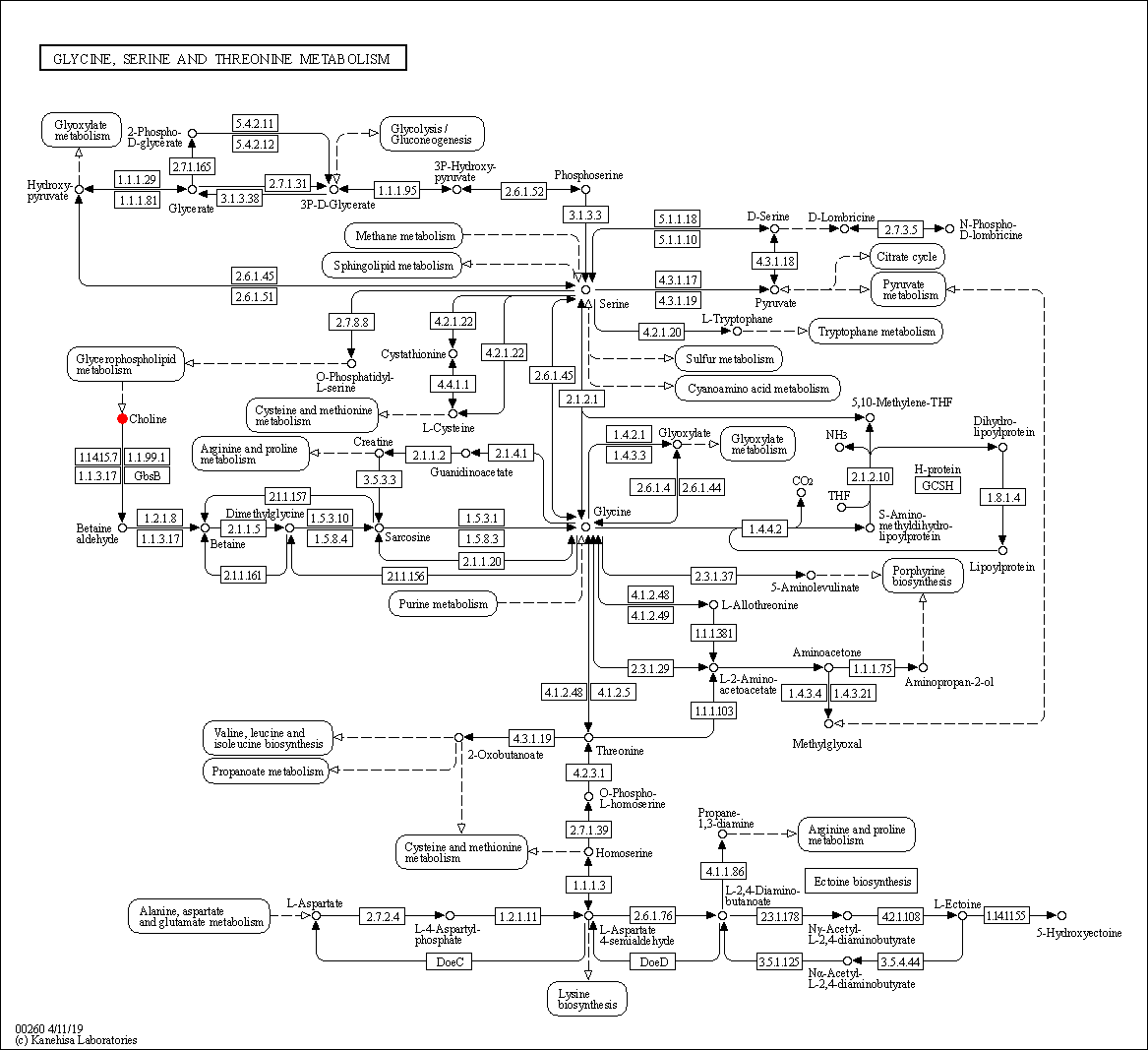

Supplement: Supplemental Information 13 [file peerj-10-14444-s013.zip › Web_Report/Diff_analysis/H_vs_L/KEGG/kegg_map/ko00260.png]

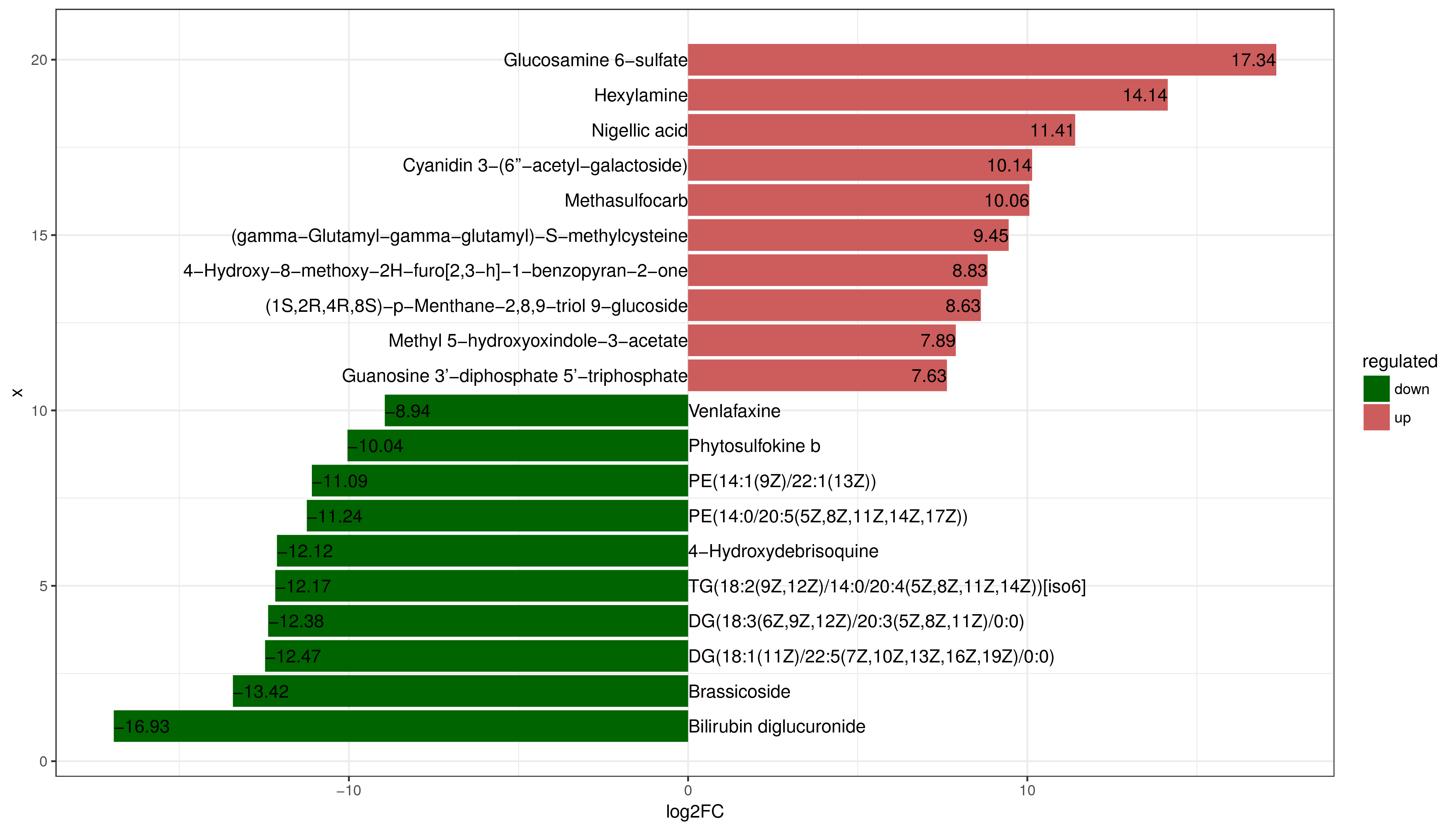

Supplement: Supplemental Information 13 [file peerj-10-14444-s013.zip › Web_Report/Diff_analysis/H_vs_L/H_vs_L_Top_20_FC_change.png]

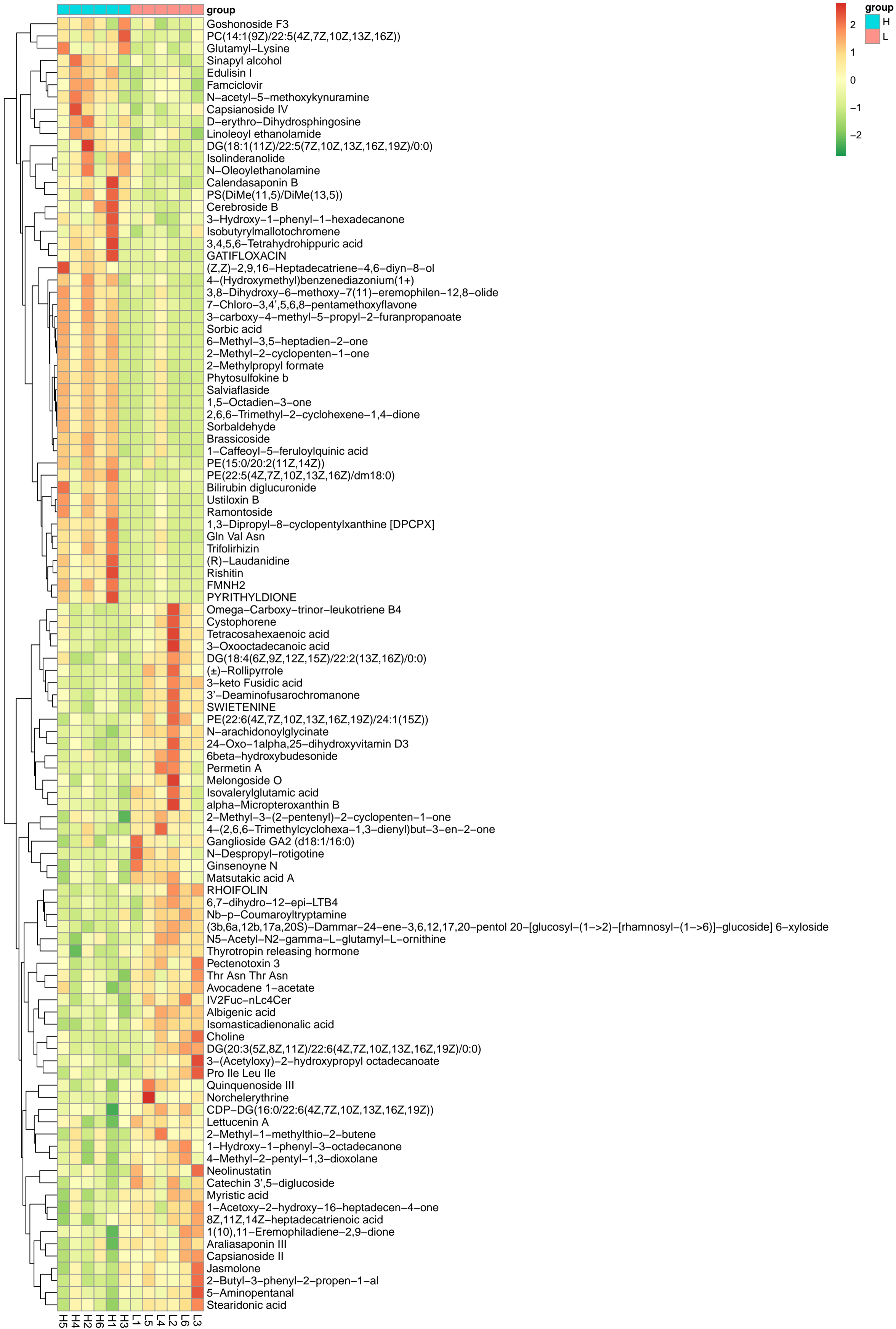

Supplement: Supplemental Information 13 [file peerj-10-14444-s013.zip › Web_Report/Diff_analysis/H_vs_L/H_vs_L_diff_heatmap_nonclustered_samples_name.pdf]

# Scores (OPLS-DA)

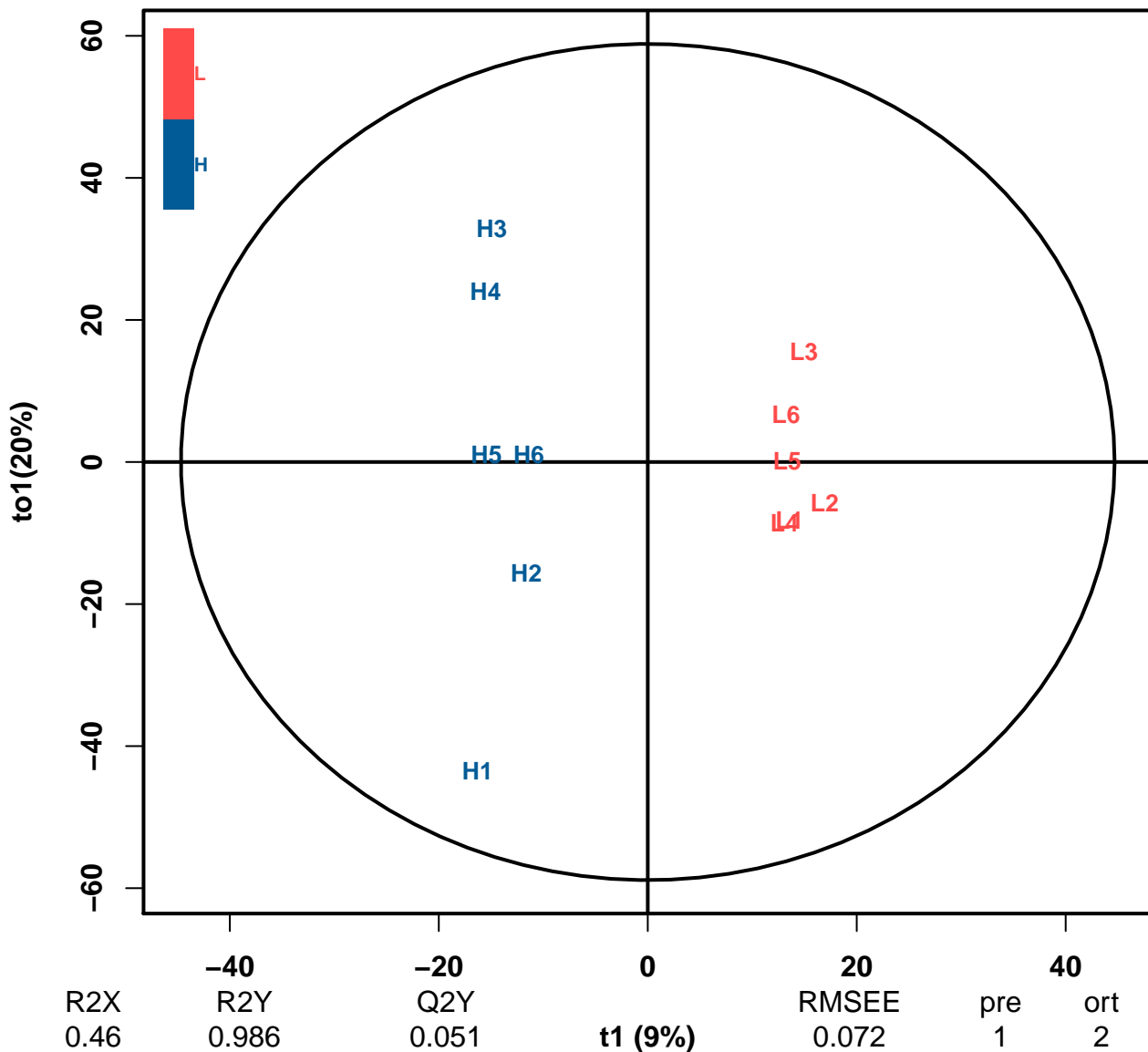

Supplement: Supplemental Information 13 [file peerj-10-14444-s013.zip › Web_Report/Diff_analysis/H_vs_L/H_vs_L_OPLS_DA.pdf]

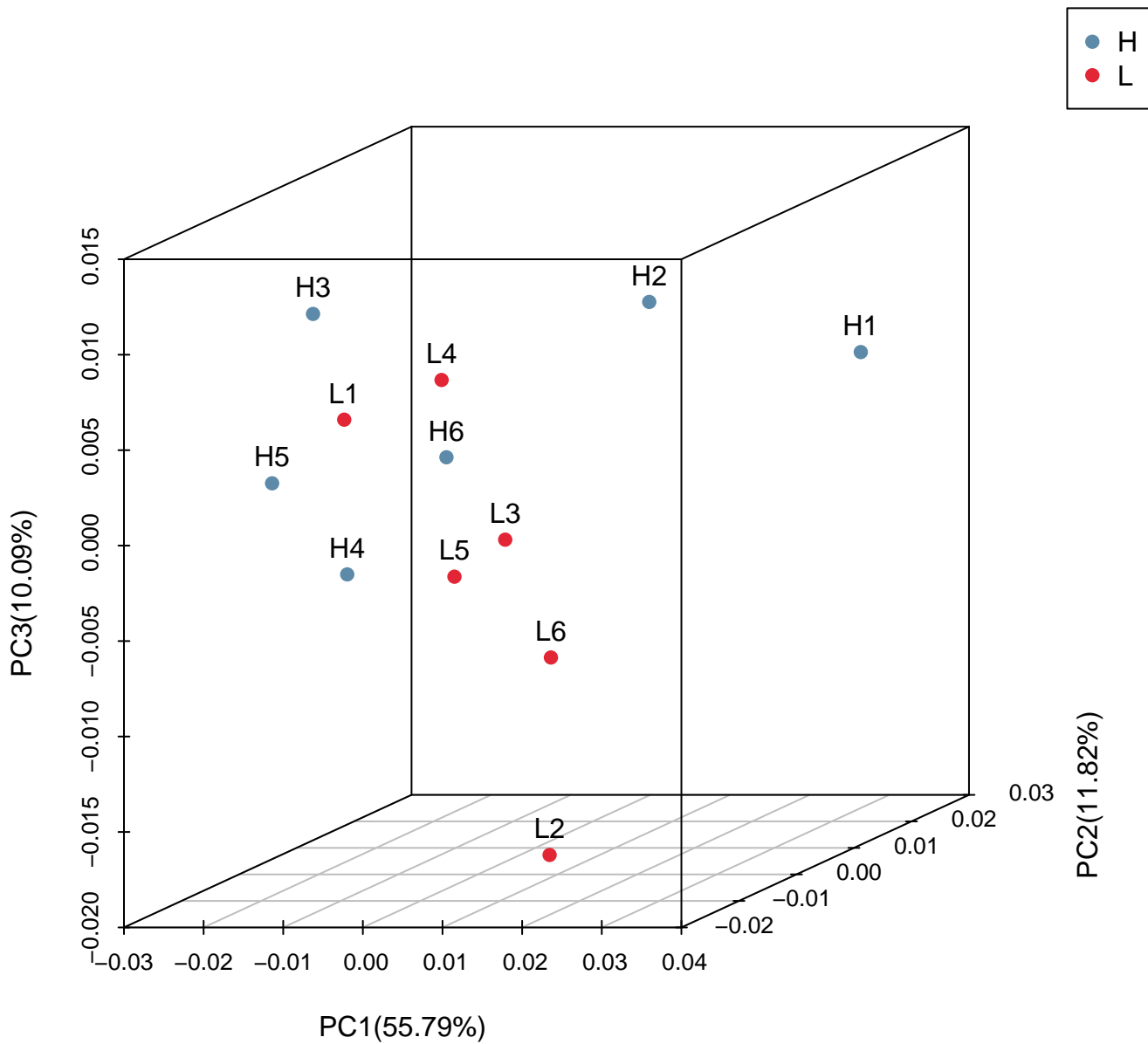

Supplement: Supplemental Information 13 [file peerj-10-14444-s013.zip › Web_Report/Diff_analysis/H_vs_L/H_vs_L_pca3D.pdf]

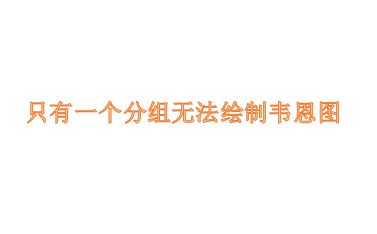

Supplement: Supplemental Information 13 [file peerj-10-14444-s013.zip › Web_Report/Diff_analysis/venn/only_one_group_venn.png]

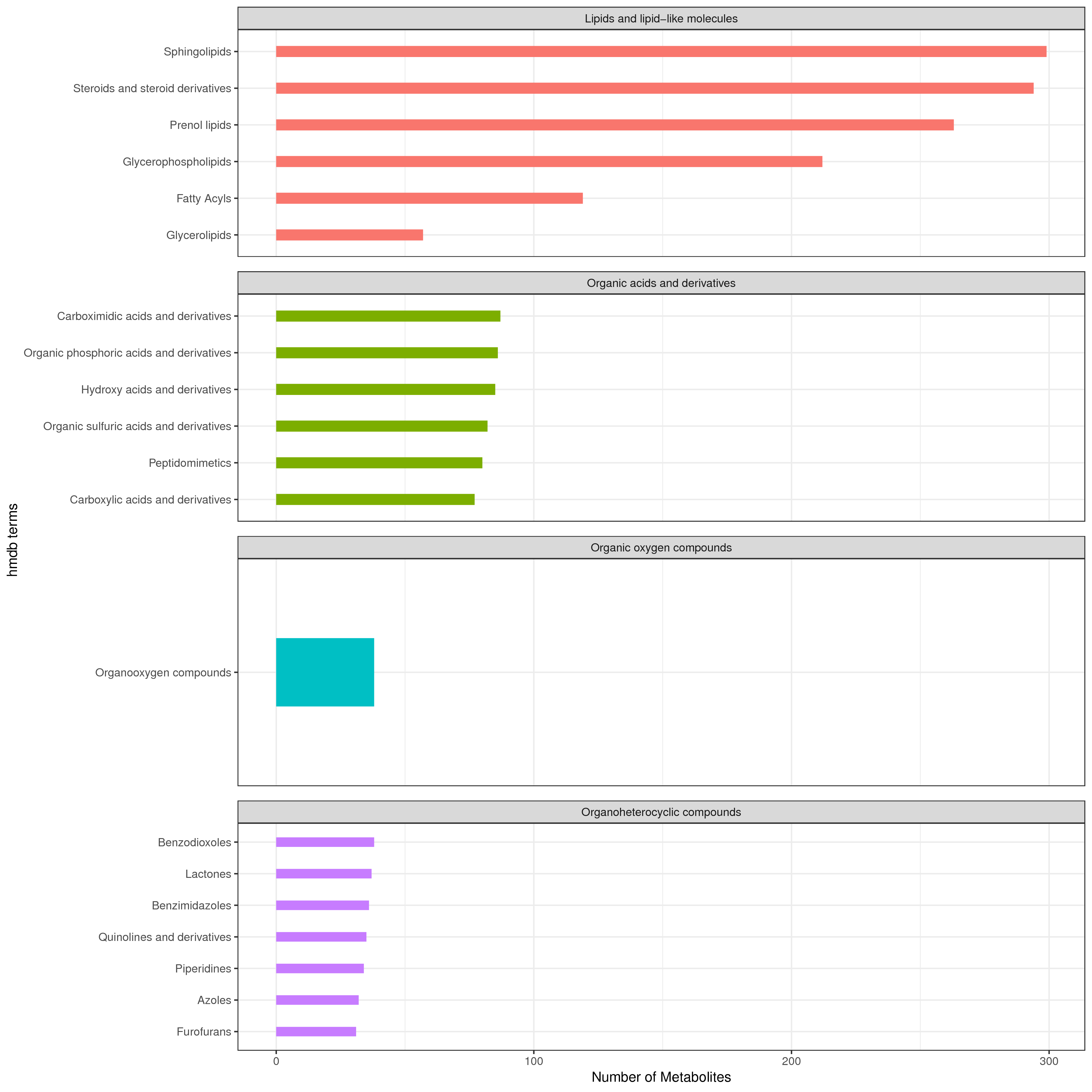

Supplement: Supplemental Information 13 [file peerj-10-14444-s013.zip › Web_Report/Metabolites_annotation/HMDB/meta_hmdb_anno.png]

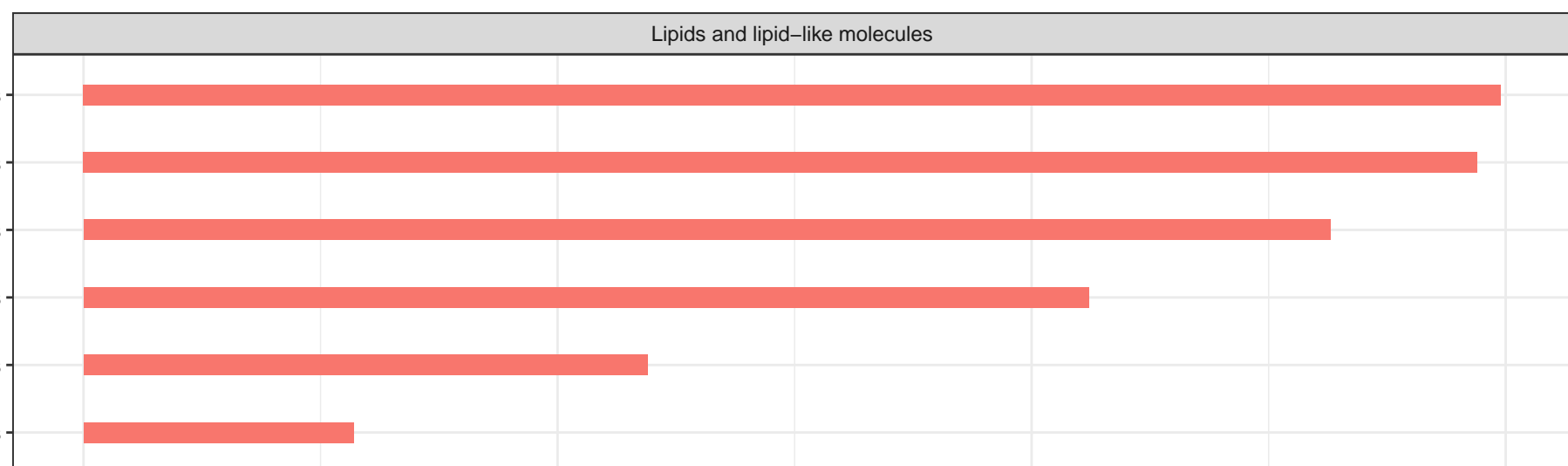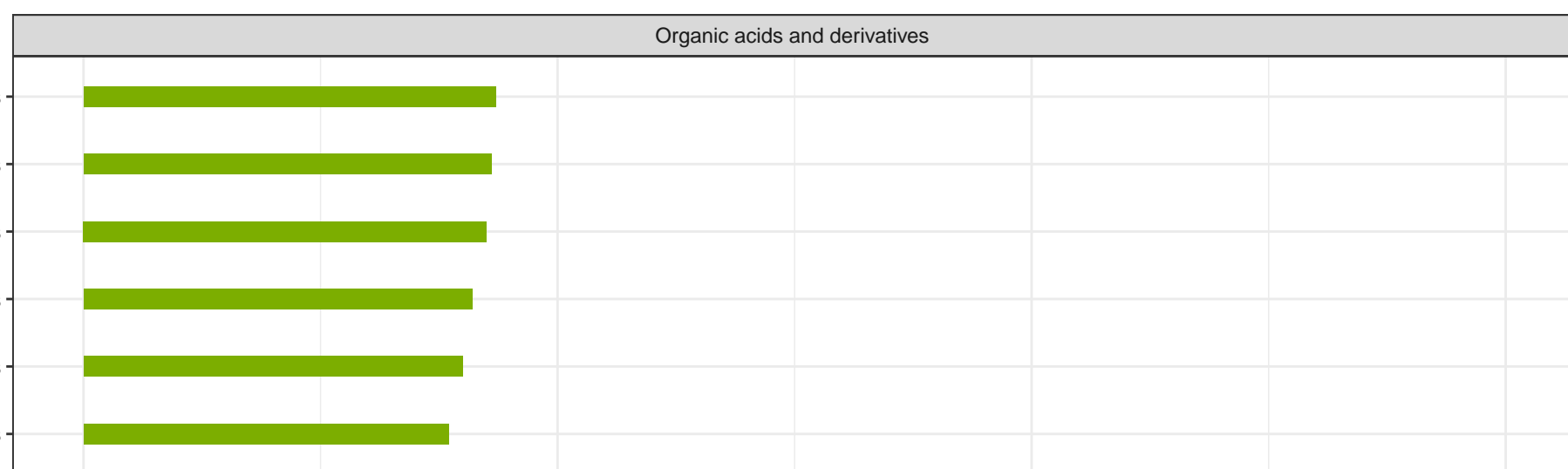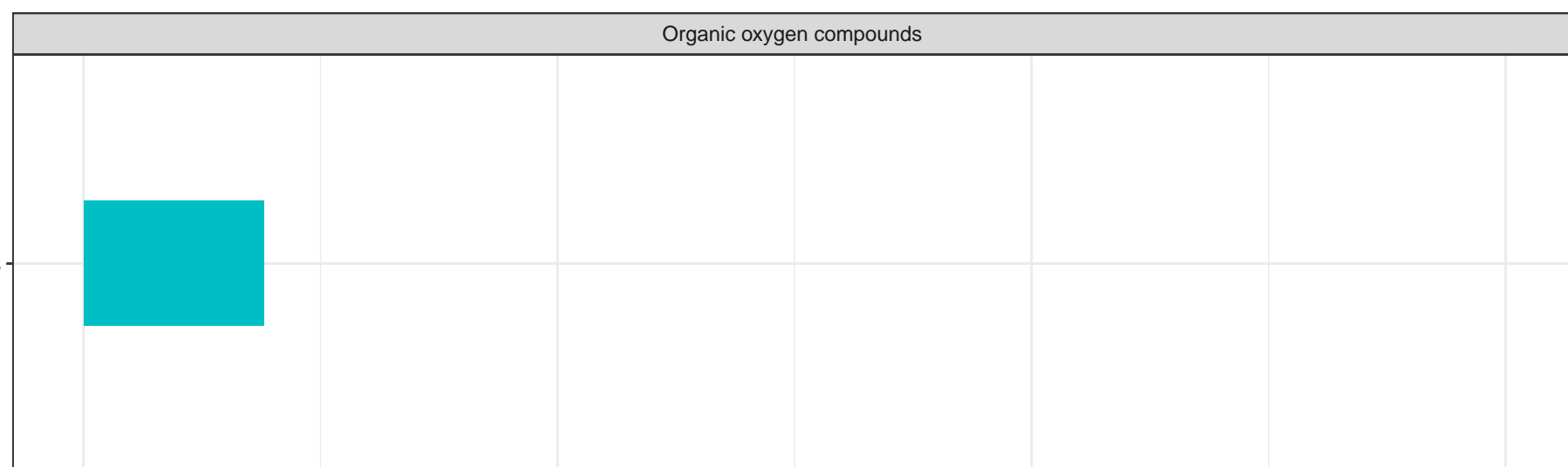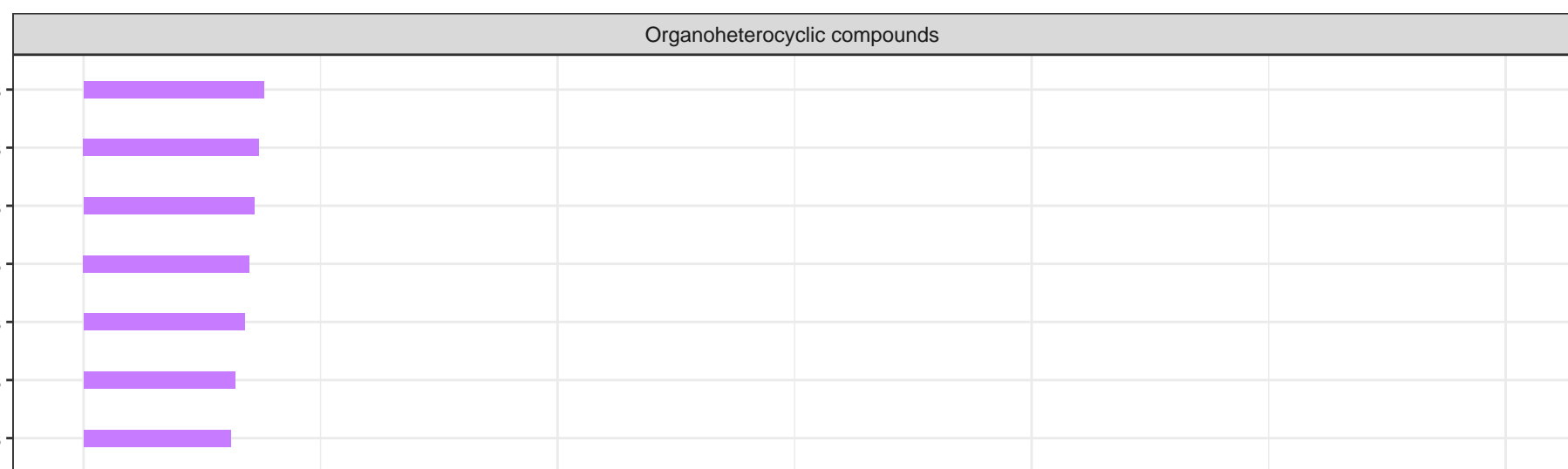

0 100 200 300

Number of Metabolites

Supplement: Supplemental Information 13 [file peerj-10-14444-s013.zip › Web_Report/Metabolites_annotation/HMDB/meta_hmdb_anno.pdf]

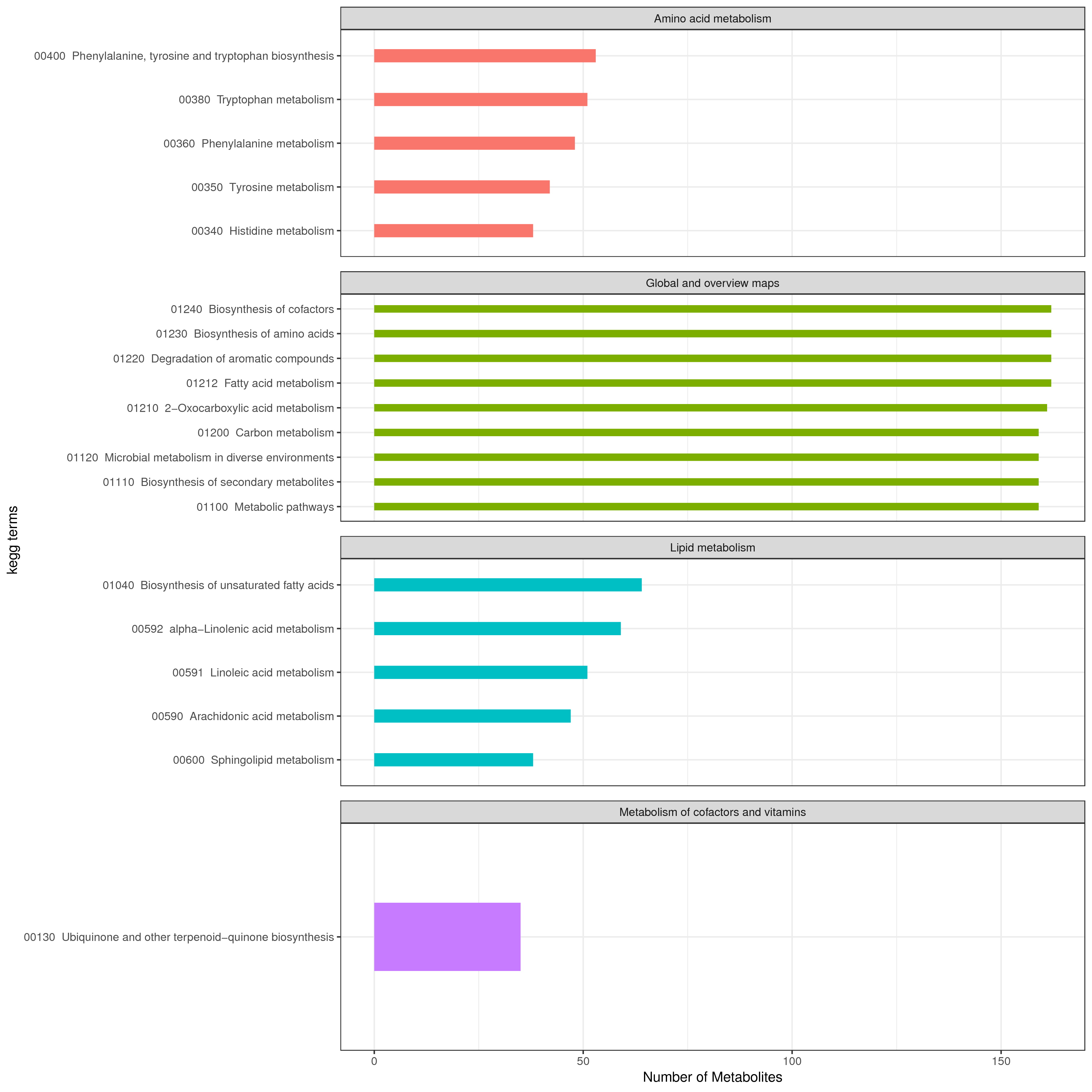

Supplement: Supplemental Information 13 [file peerj-10-14444-s013.zip › Web_Report/Metabolites_annotation/KEGG/meta_kegg_anno.png]

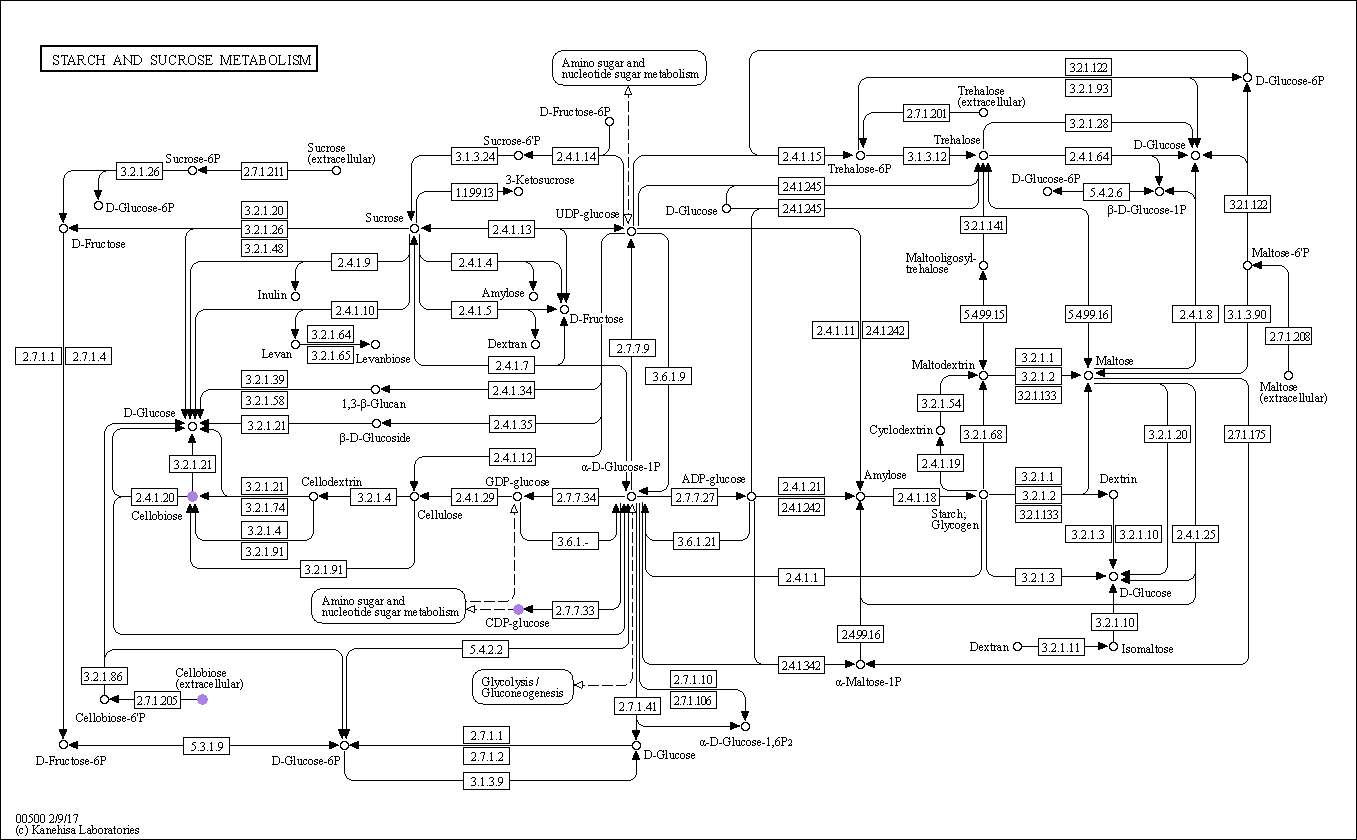

Supplement: Supplemental Information 13 [file peerj-10-14444-s013.zip › Web_Report/Metabolites_annotation/KEGG/All_kegg_map/ko00500.png]

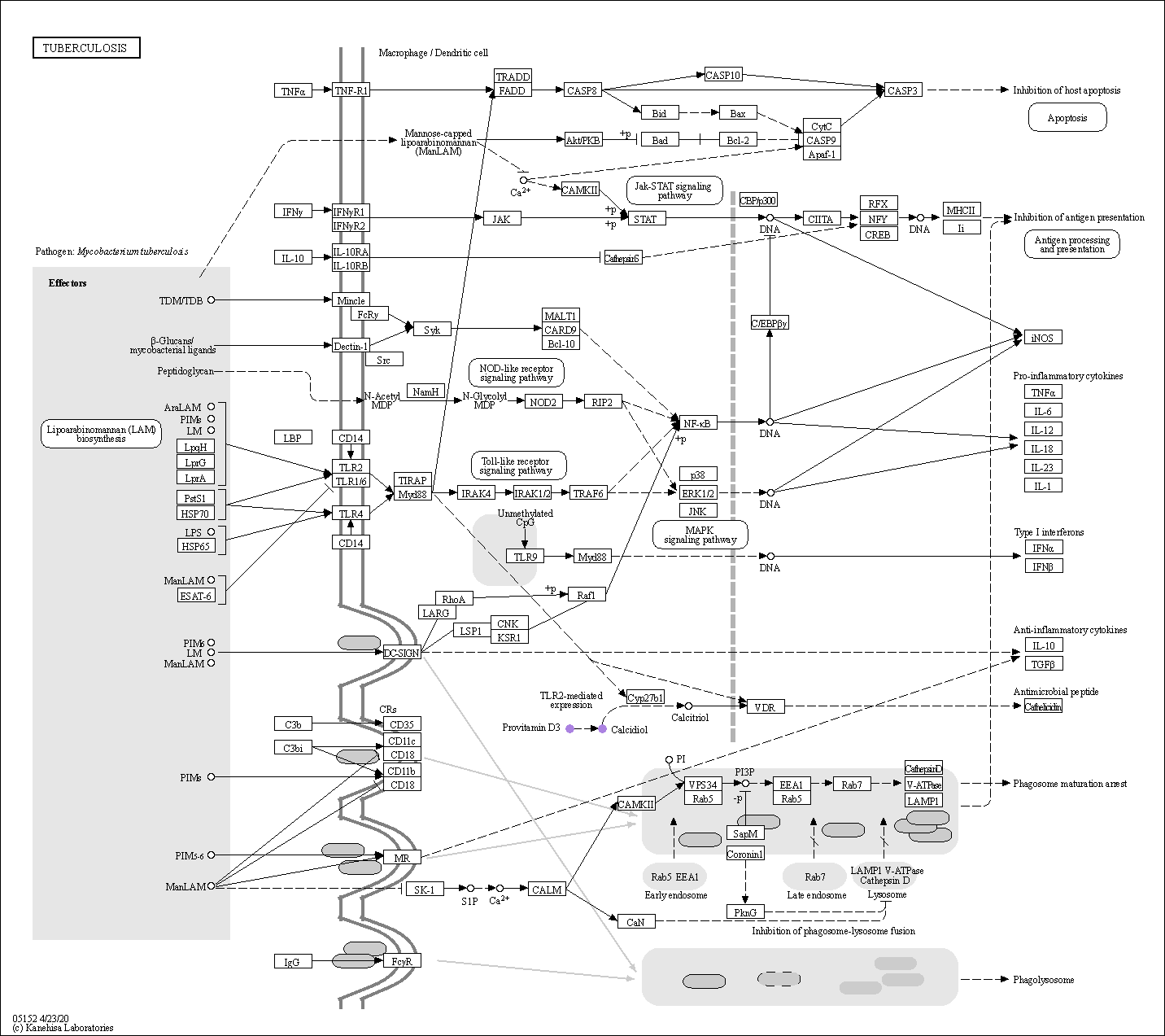

Supplement: Supplemental Information 13 [file peerj-10-14444-s013.zip › Web_Report/Metabolites_annotation/KEGG/All_kegg_map/ko05152.png]

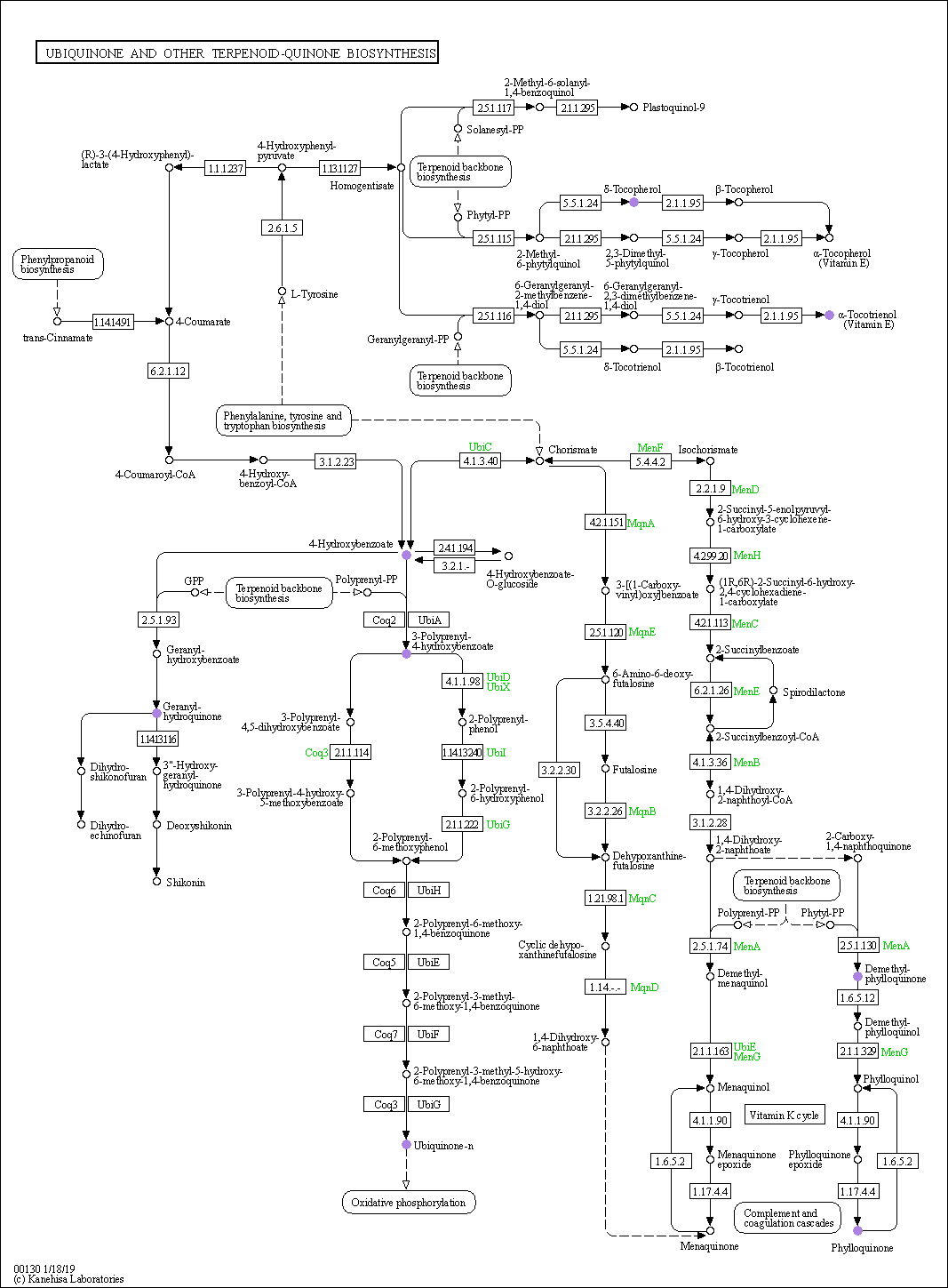

Supplement: Supplemental Information 13 [file peerj-10-14444-s013.zip › Web_Report/Metabolites_annotation/KEGG/All_kegg_map/ko00130.png]

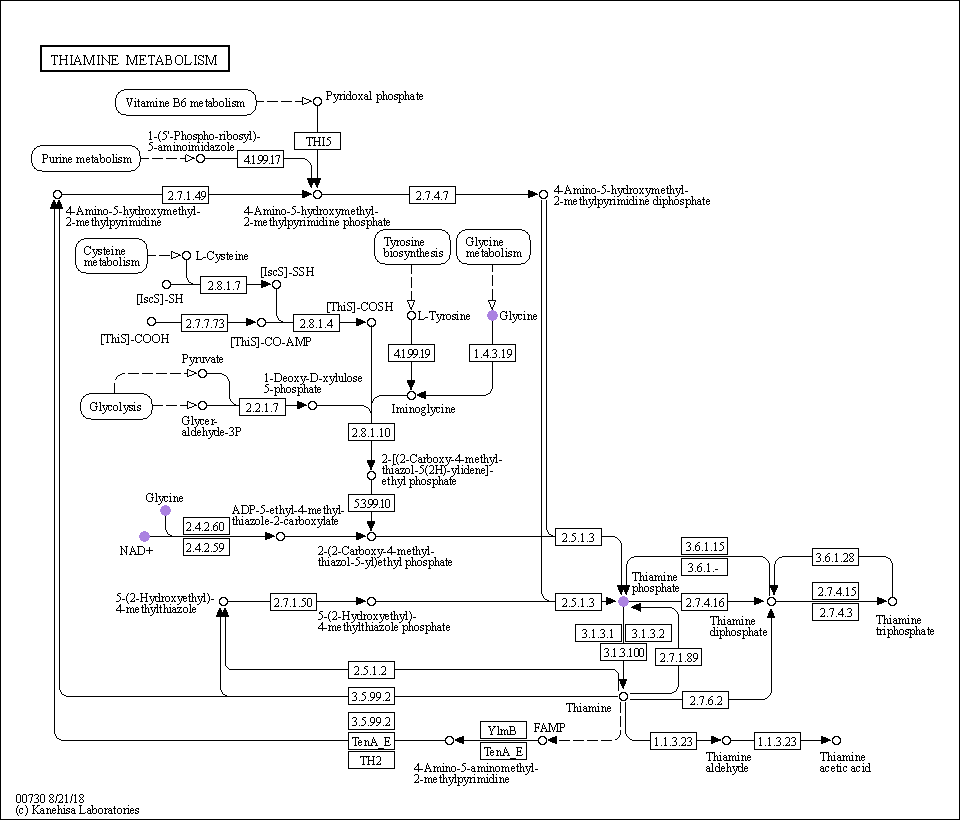

Supplement: Supplemental Information 13 [file peerj-10-14444-s013.zip › Web_Report/Metabolites_annotation/KEGG/All_kegg_map/ko00730.png]

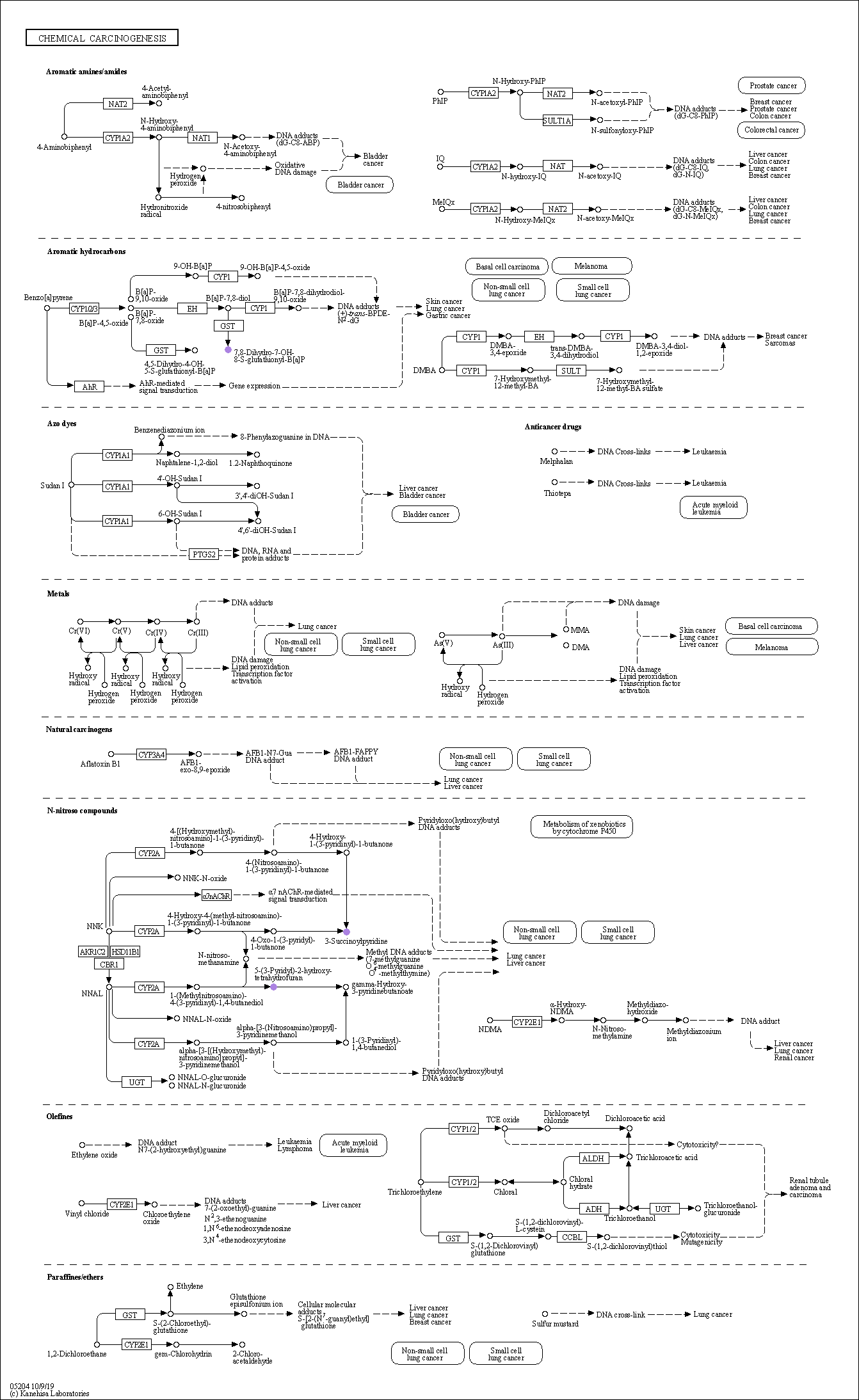

Supplement: Supplemental Information 13 [file peerj-10-14444-s013.zip › Web_Report/Metabolites_annotation/KEGG/All_kegg_map/ko05204.png]

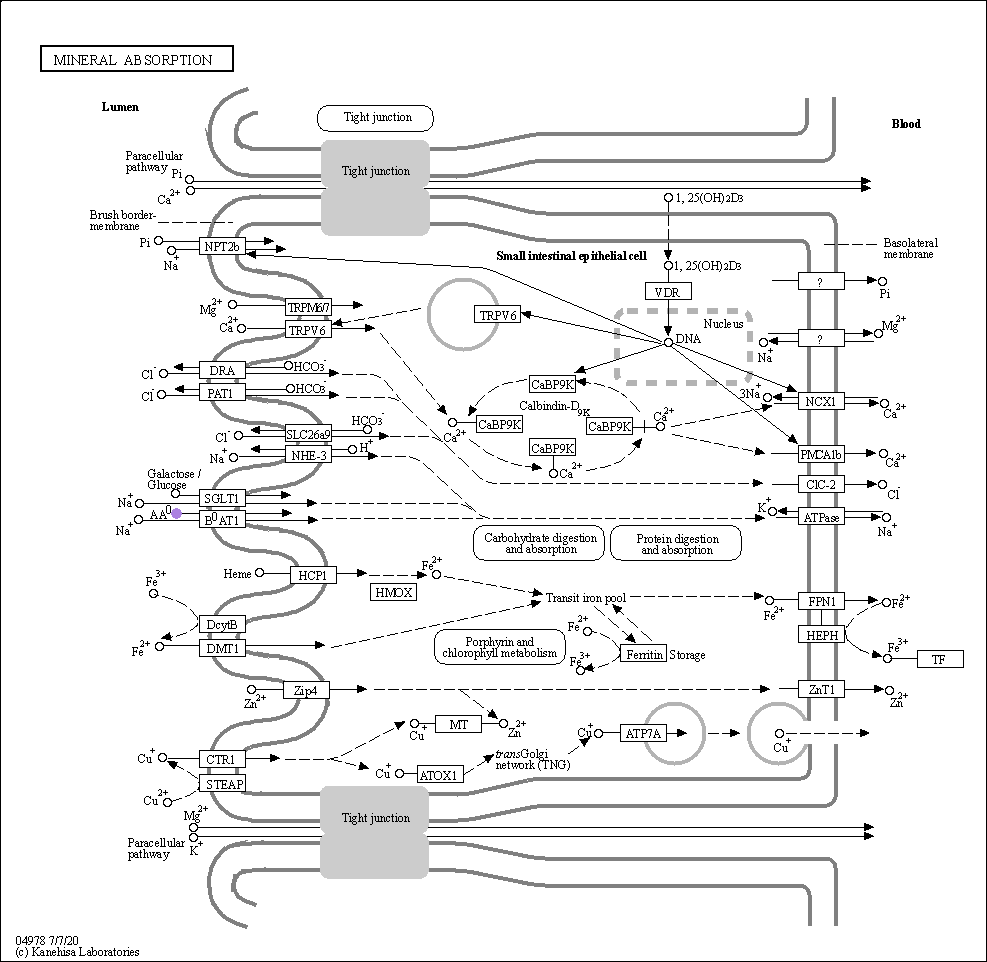

Supplement: Supplemental Information 13 [file peerj-10-14444-s013.zip › Web_Report/Metabolites_annotation/KEGG/All_kegg_map/ko04978.png]

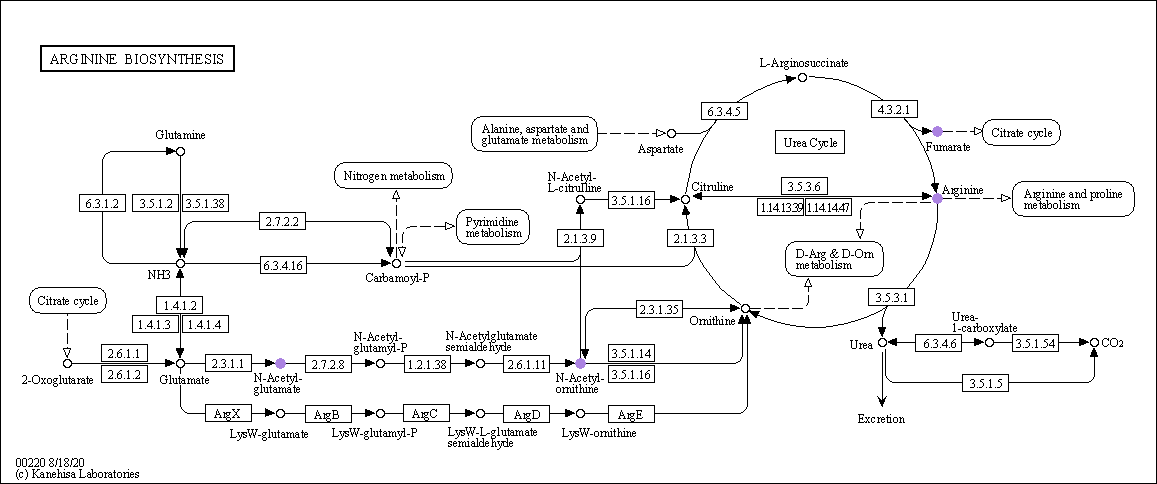

Supplement: Supplemental Information 13 [file peerj-10-14444-s013.zip › Web_Report/Metabolites_annotation/KEGG/All_kegg_map/ko00220.png]

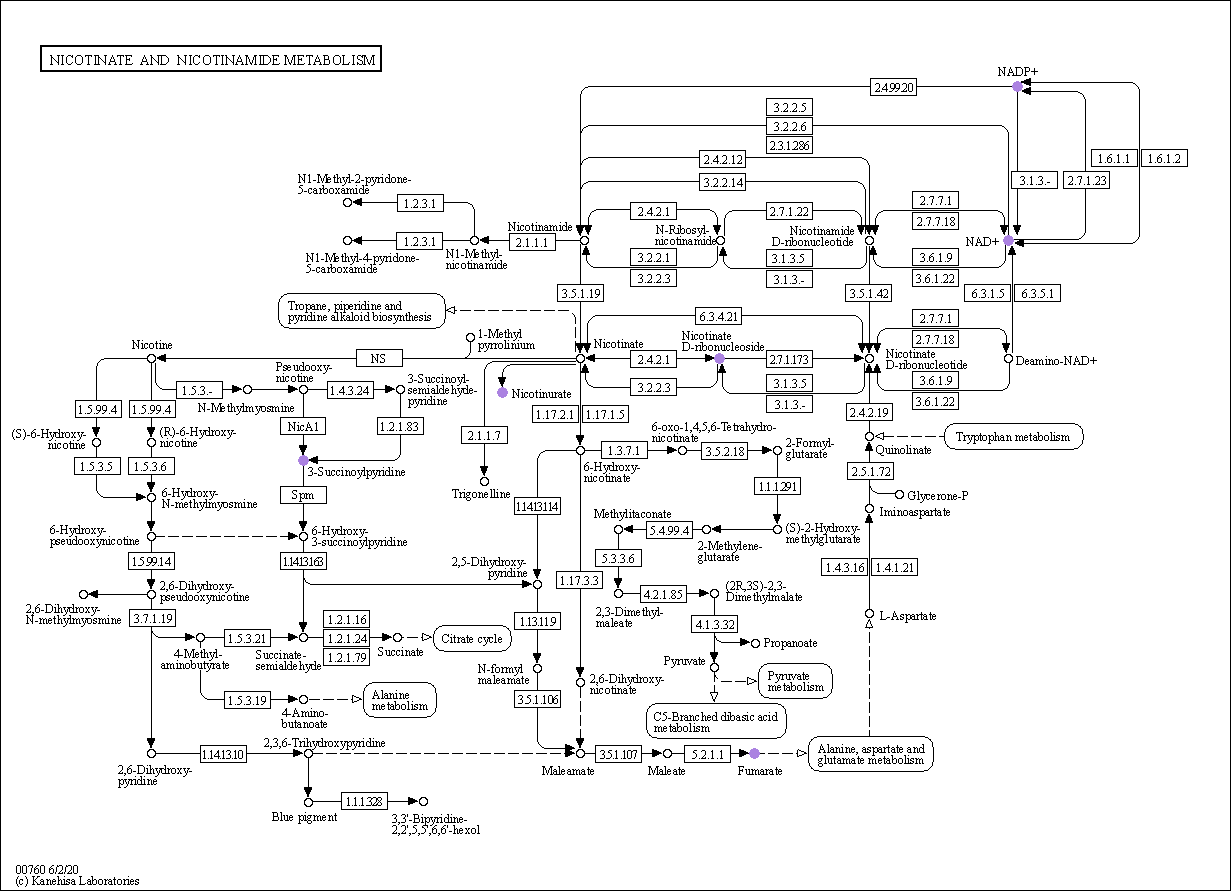

Supplement: Supplemental Information 13 [file peerj-10-14444-s013.zip › Web_Report/Metabolites_annotation/KEGG/All_kegg_map/ko00760.png]

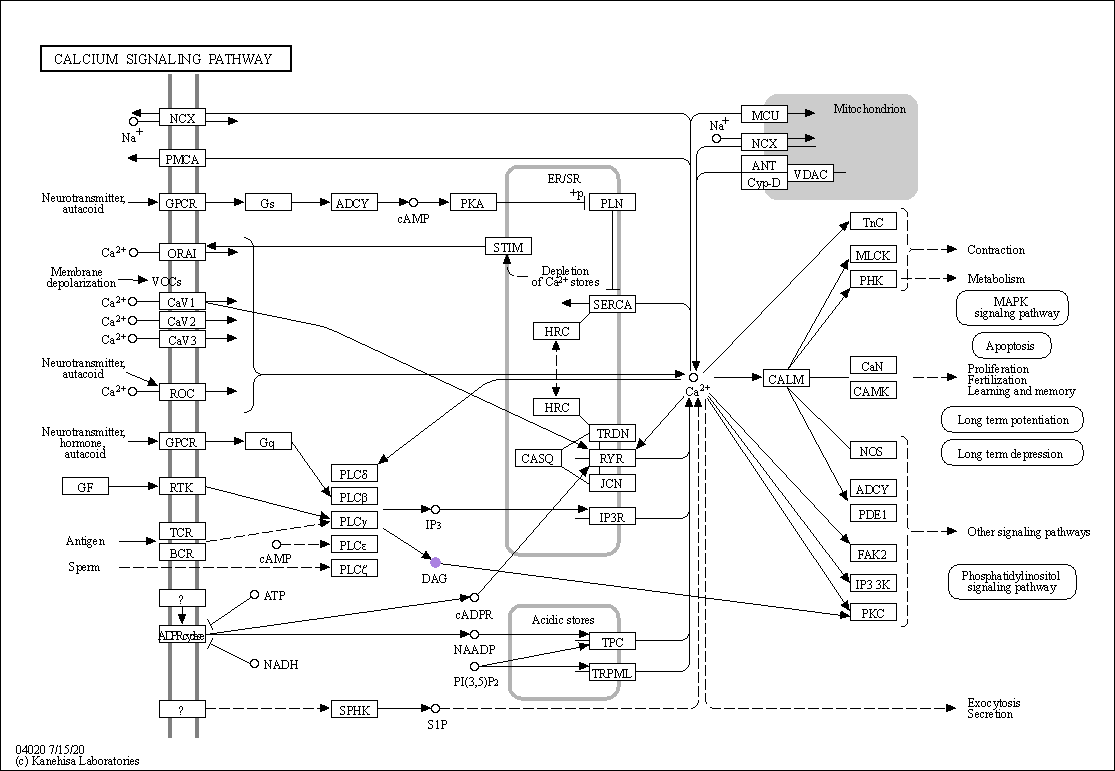

Supplement: Supplemental Information 13 [file peerj-10-14444-s013.zip › Web_Report/Metabolites_annotation/KEGG/All_kegg_map/ko04020.png]

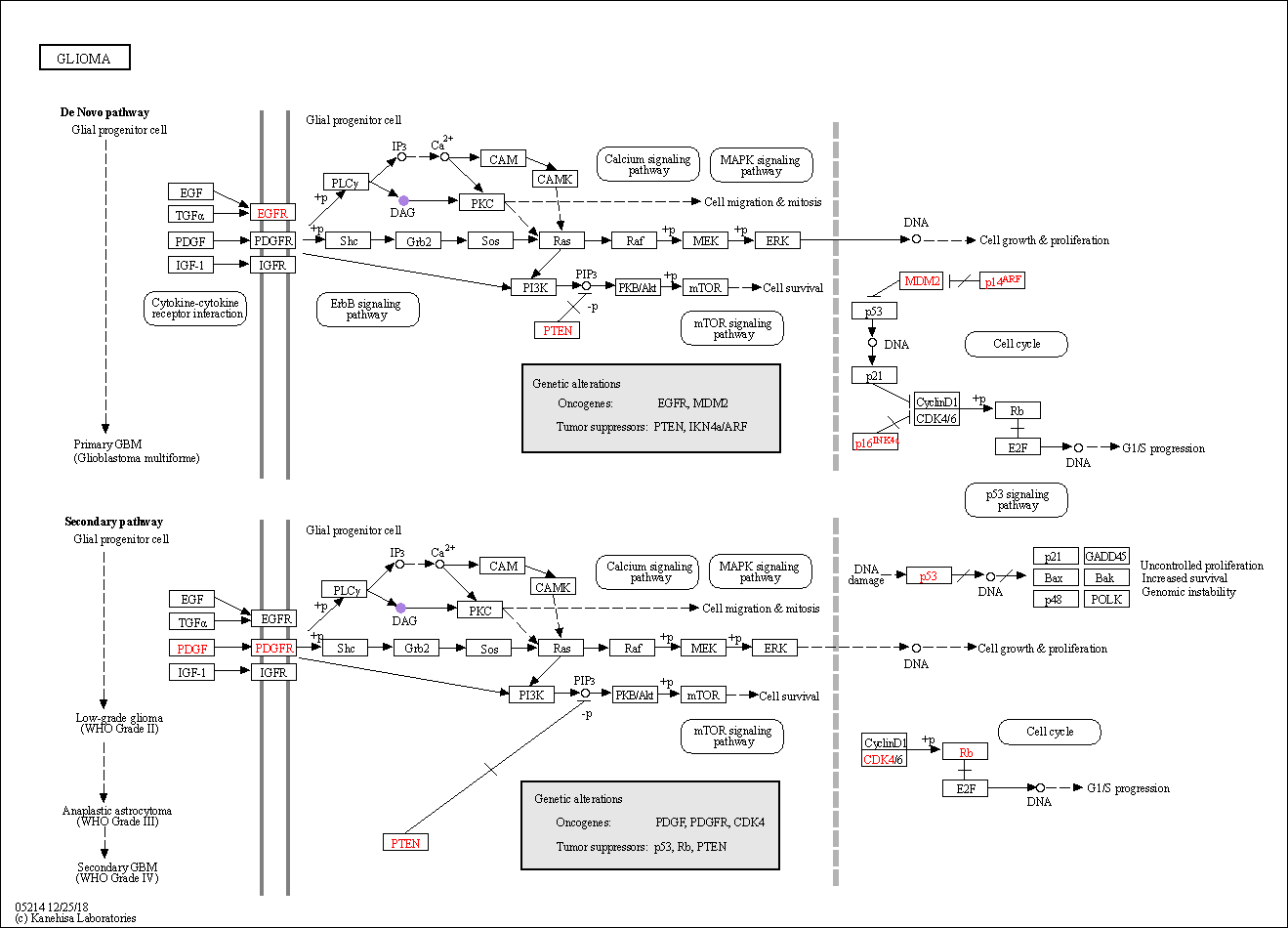

Supplement: Supplemental Information 13 [file peerj-10-14444-s013.zip › Web_Report/Metabolites_annotation/KEGG/All_kegg_map/ko05214.png]

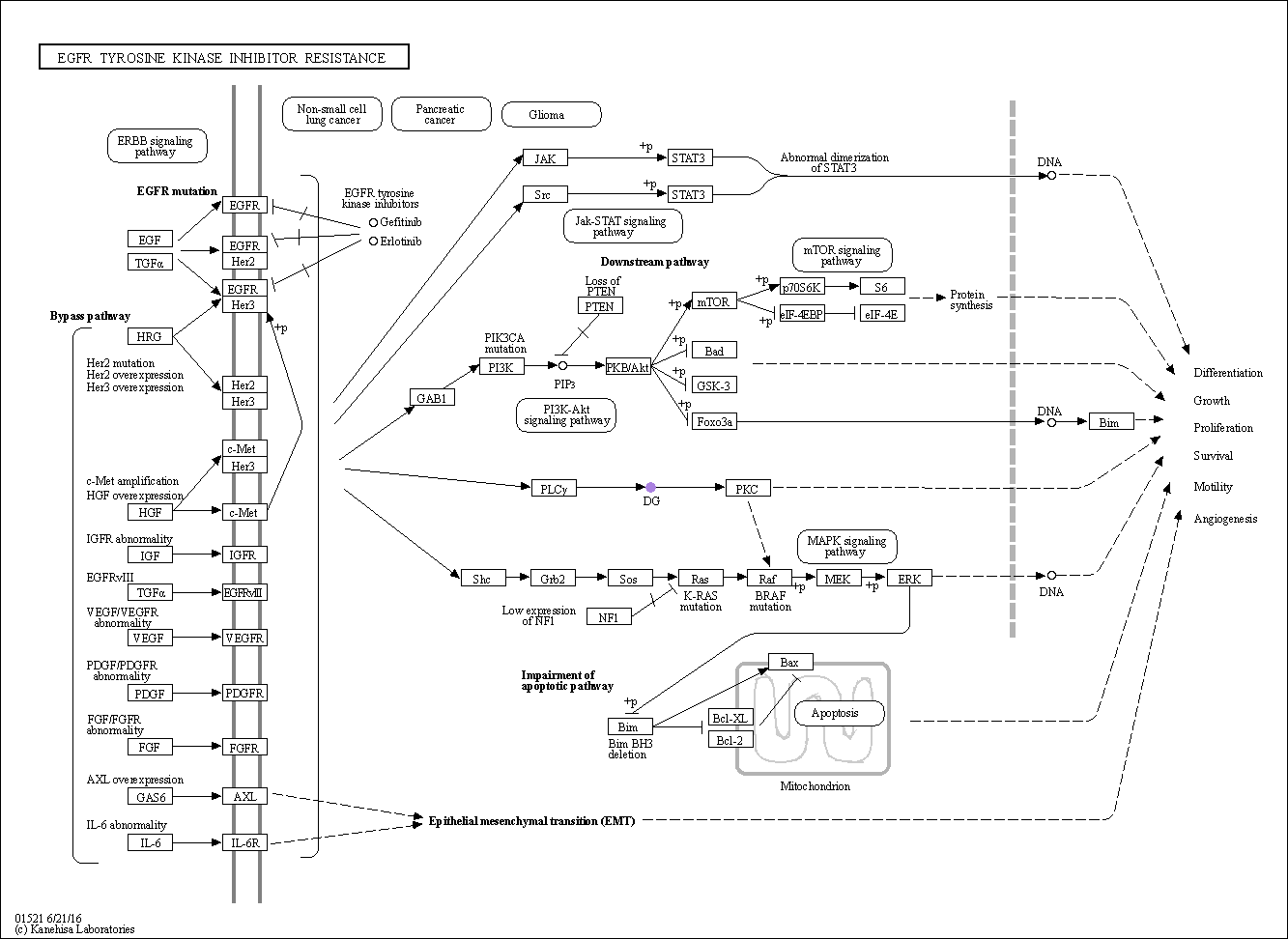

Supplement: Supplemental Information 13 [file peerj-10-14444-s013.zip › Web_Report/Metabolites_annotation/KEGG/All_kegg_map/ko01521.png]

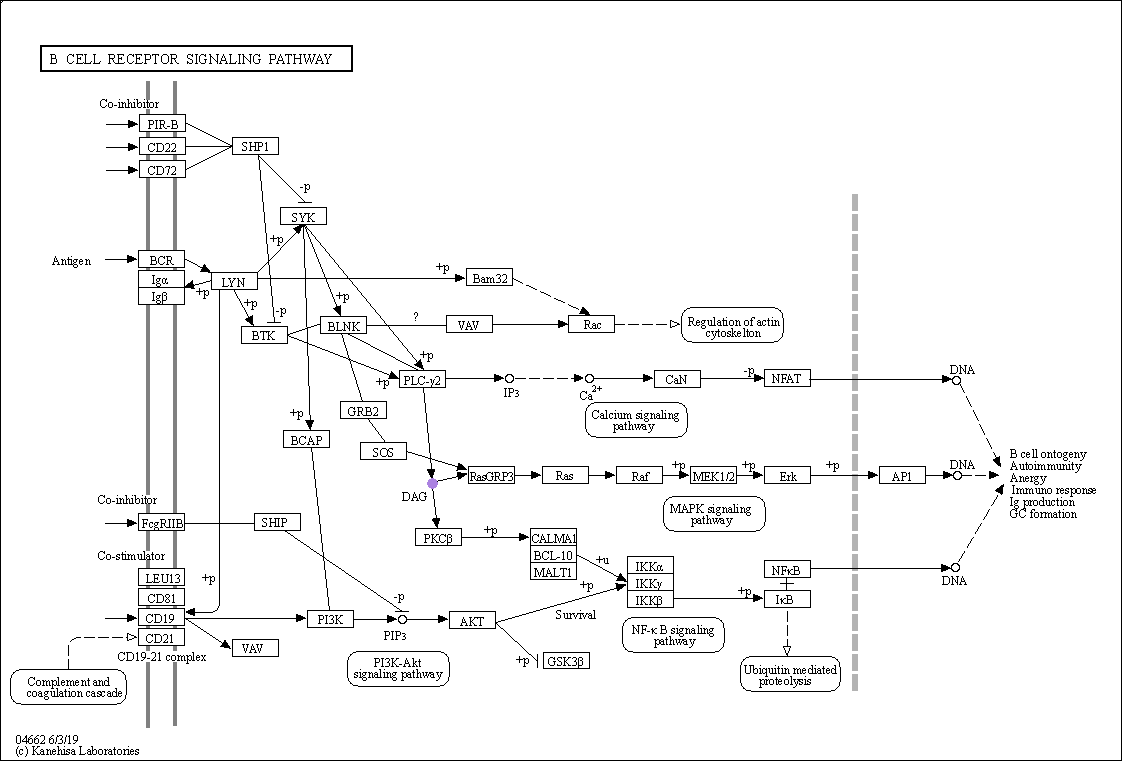

Supplement: Supplemental Information 13 [file peerj-10-14444-s013.zip › Web_Report/Metabolites_annotation/KEGG/All_kegg_map/ko04662.png]

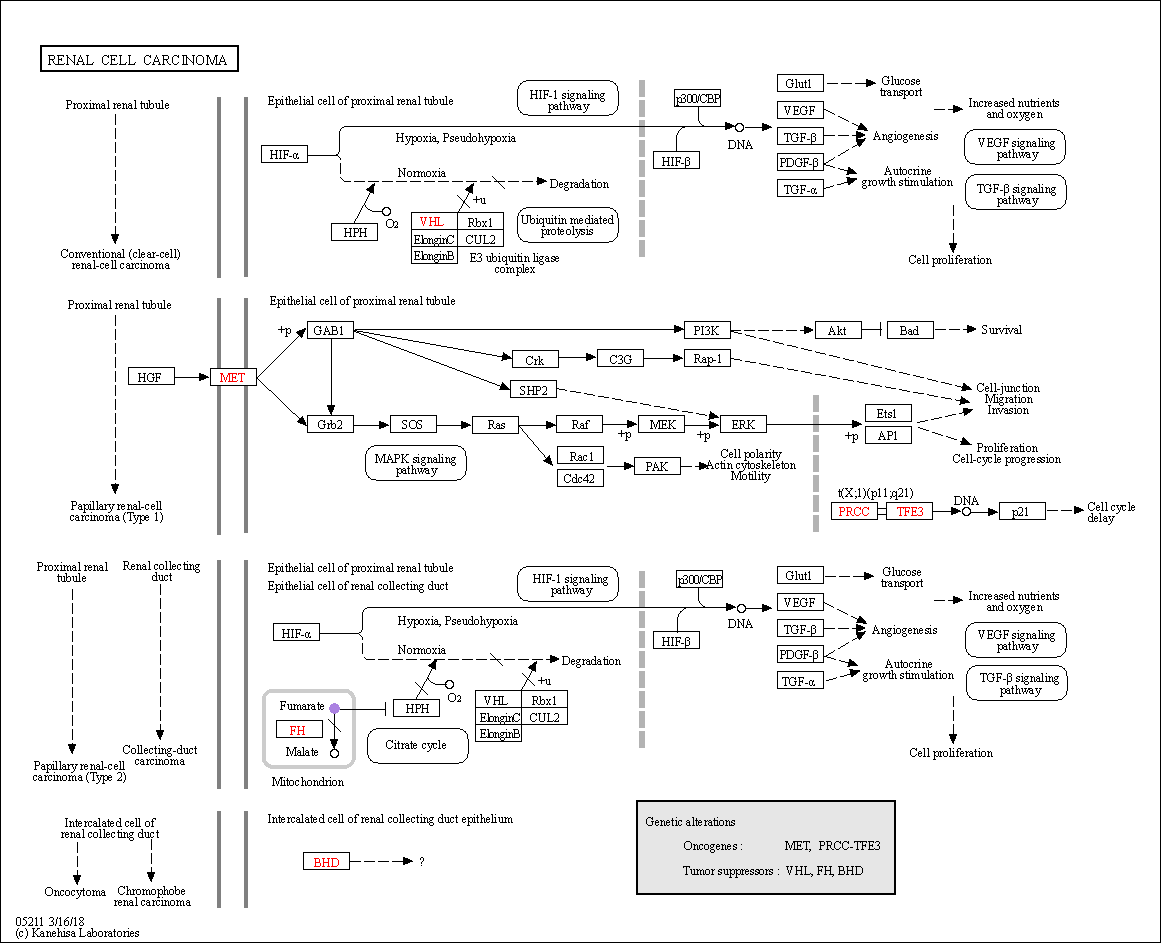

Supplement: Supplemental Information 13 [file peerj-10-14444-s013.zip › Web_Report/Metabolites_annotation/KEGG/All_kegg_map/ko05211.png]

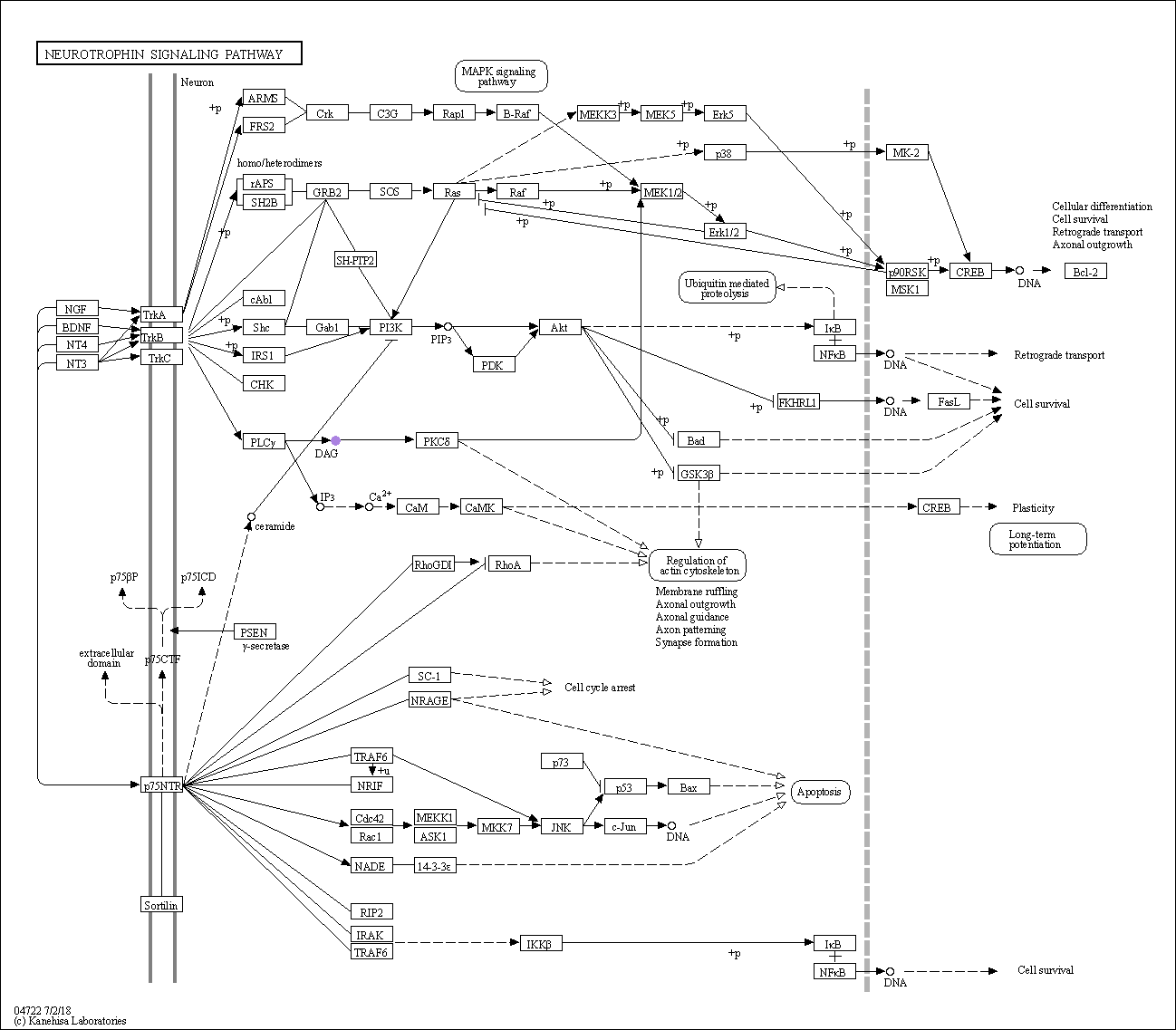

Supplement: Supplemental Information 13 [file peerj-10-14444-s013.zip › Web_Report/Metabolites_annotation/KEGG/All_kegg_map/ko04722.png]

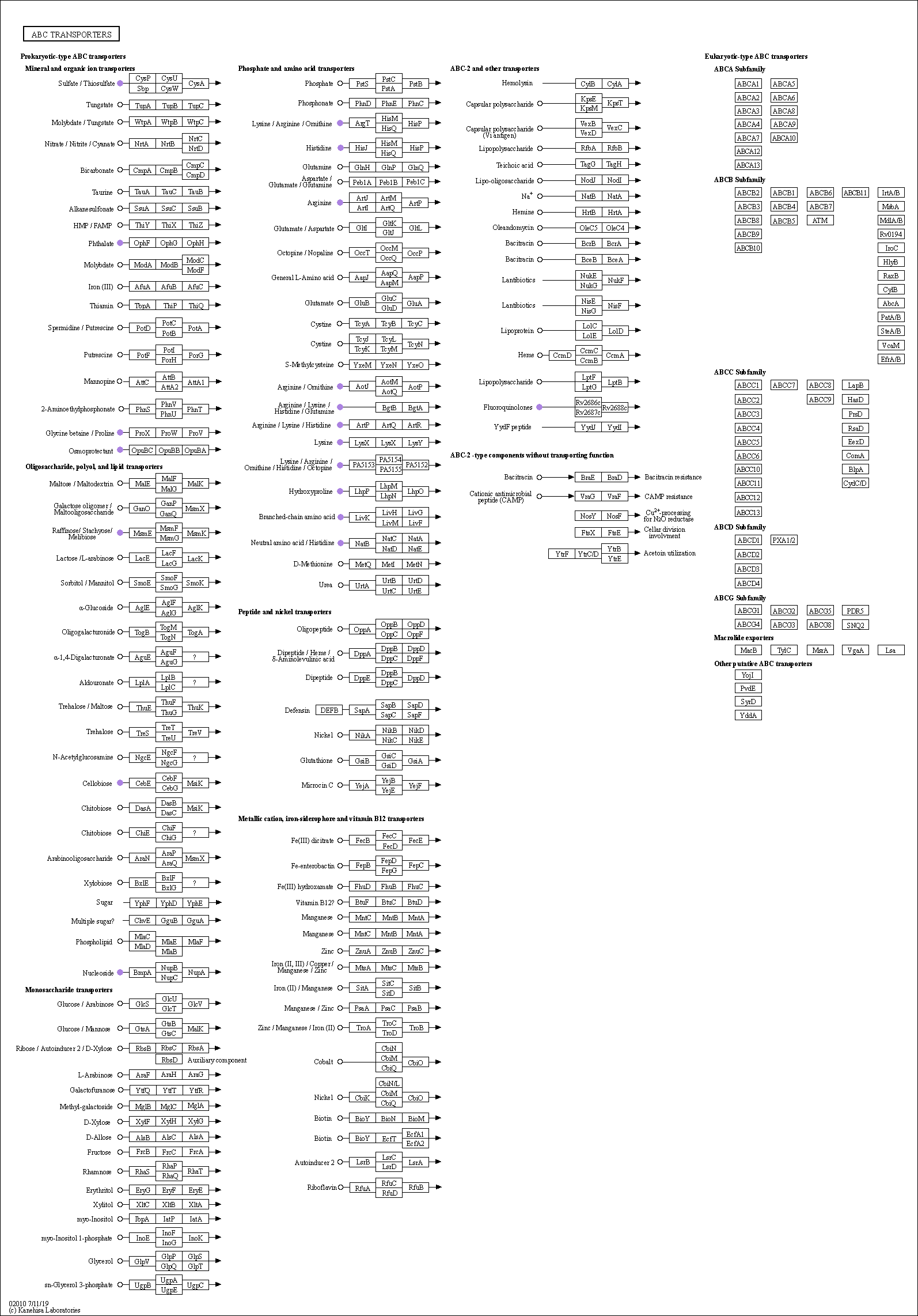

Supplement: Supplemental Information 13 [file peerj-10-14444-s013.zip › Web_Report/Metabolites_annotation/KEGG/All_kegg_map/ko02010.png]

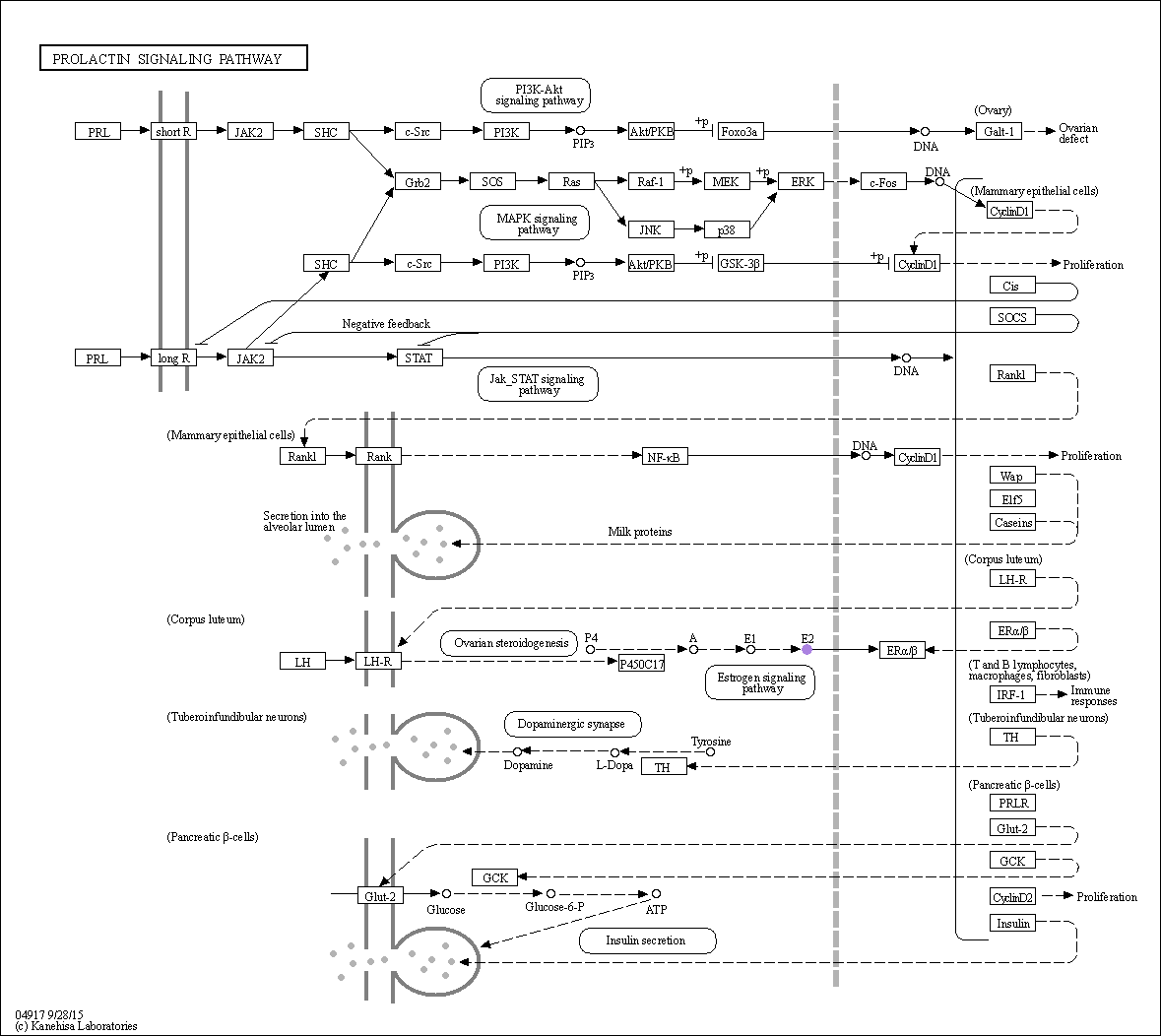

Supplement: Supplemental Information 13 [file peerj-10-14444-s013.zip › Web_Report/Metabolites_annotation/KEGG/All_kegg_map/ko04917.png]

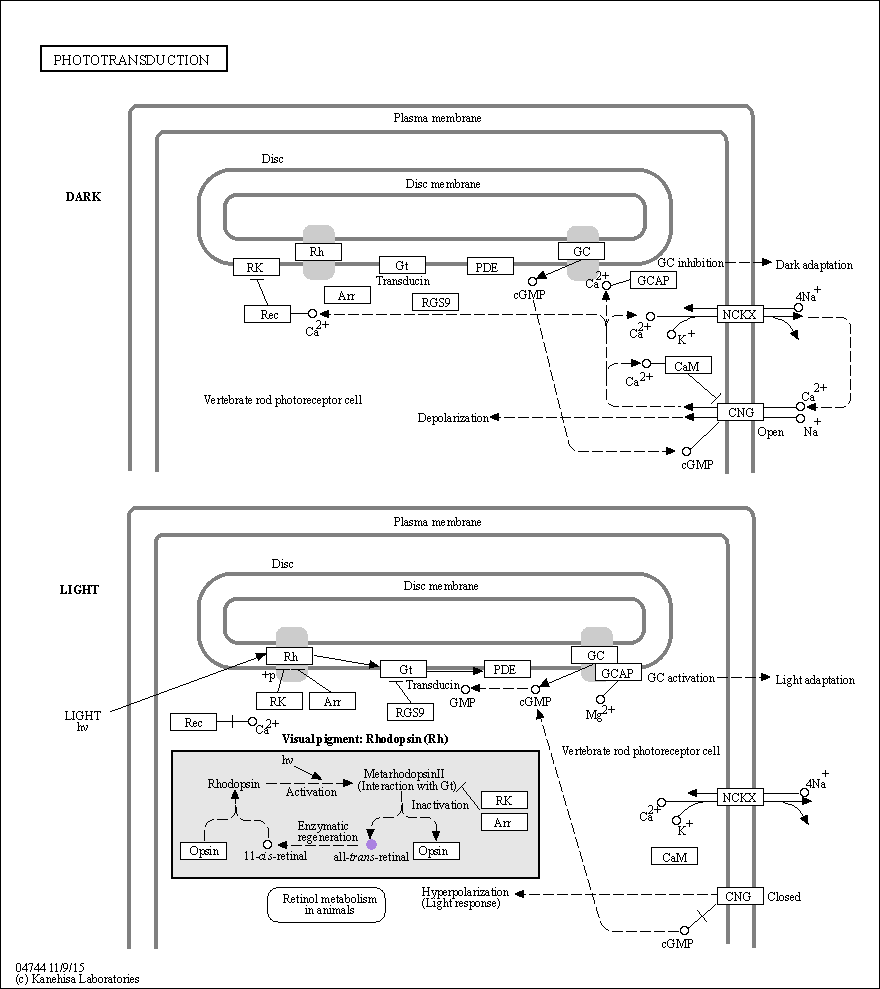

Supplement: Supplemental Information 13 [file peerj-10-14444-s013.zip › Web_Report/Metabolites_annotation/KEGG/All_kegg_map/ko04744.png]

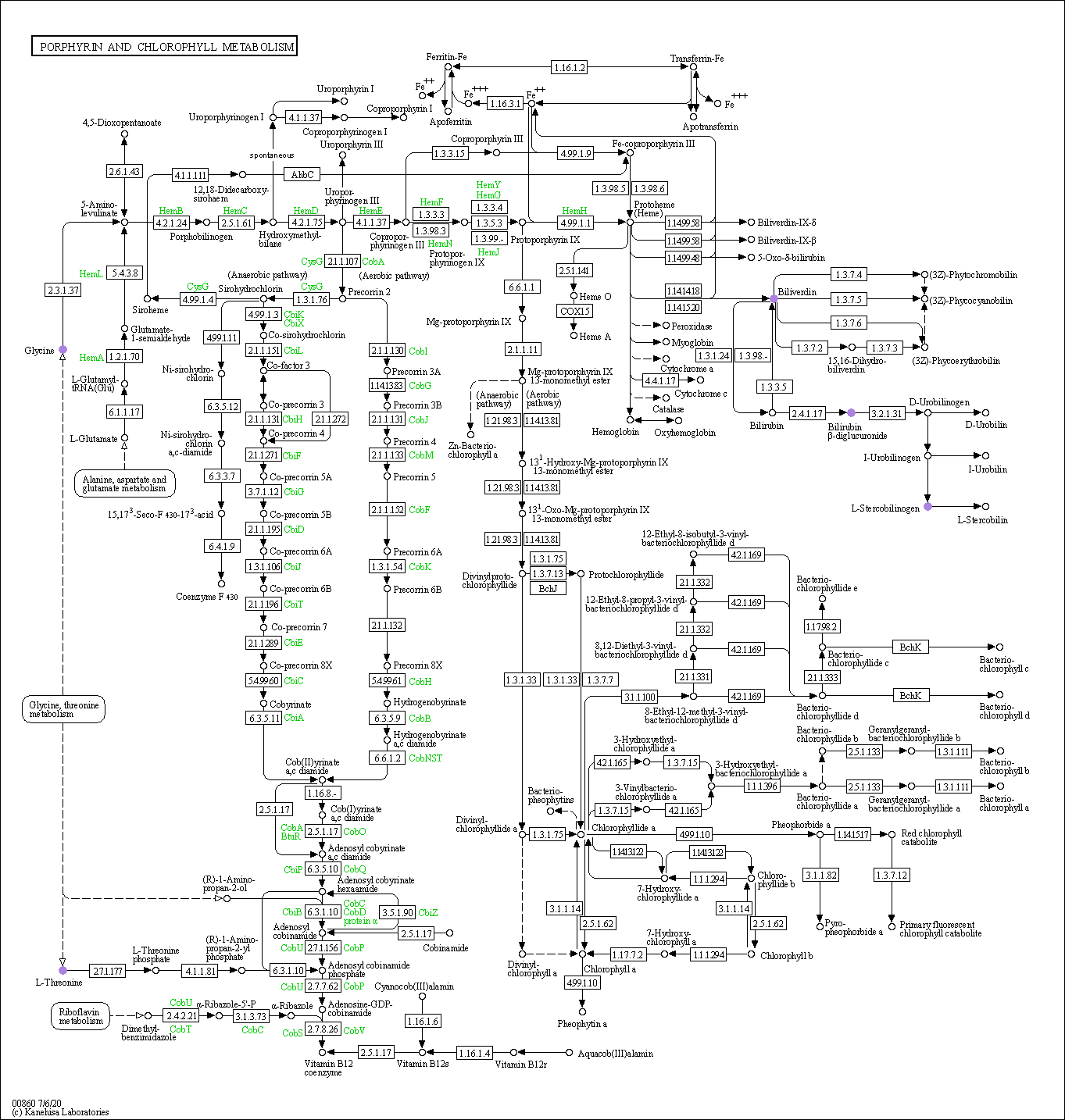

Supplement: Supplemental Information 13 [file peerj-10-14444-s013.zip › Web_Report/Metabolites_annotation/KEGG/All_kegg_map/ko00860.png]

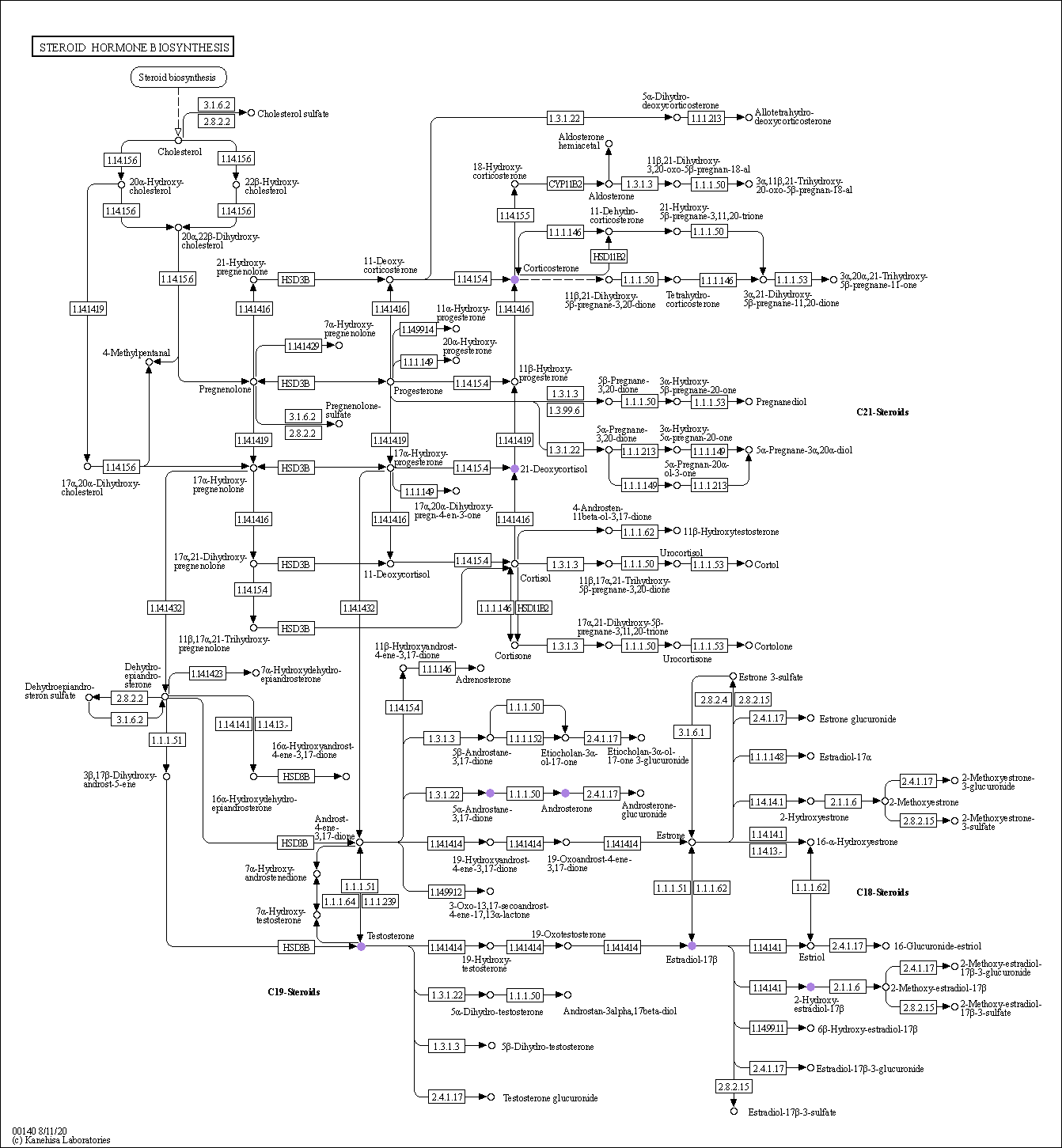

Supplement: Supplemental Information 13 [file peerj-10-14444-s013.zip › Web_Report/Metabolites_annotation/KEGG/All_kegg_map/ko00140.png]
