# Supplementary material for: Multi-omics analyses reveal that the gut microbiome and its metabolites promote milk fat synthesis in Zhongdian yak cows
Source: PeerJ. 2022 Dec 2;10:e14444. doi: 10.7717/peerj.14444 (PMC9744170; doi:10.7717/peerj.14444)
Supplement: Supplemental Information 10 [file peerj-10-14444-s010.zip › network.neededData.zip!/network.pdf]

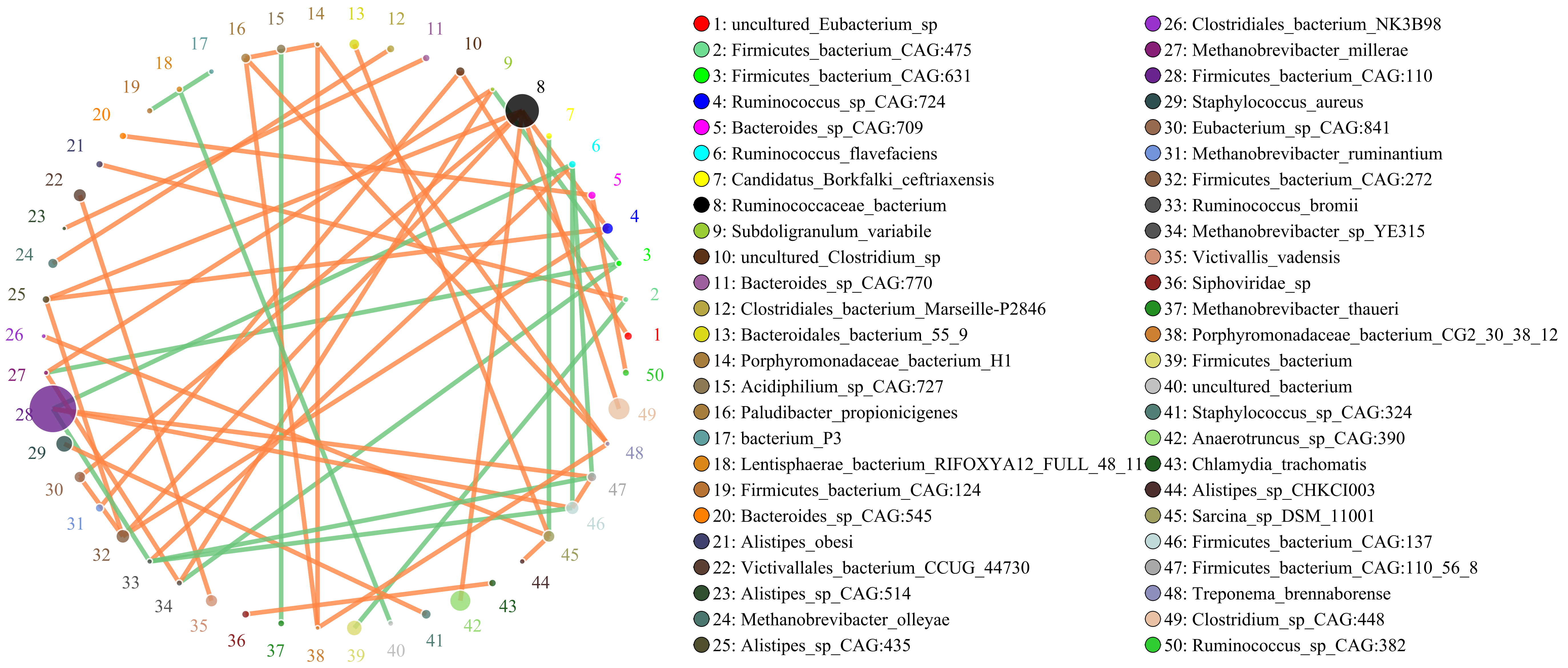

1: uncultured_Eubacterium_sp
2: Firmicutes_bacterium_CAG:475
3: Firmicutes_bacterium_CAG:631
4: Ruminococcus_sp_CAG:724
5: Bacteroides_sp_CAG:709
6: Ruminococcus_flavefaciens
7: Candidatus_Borkfalki_ceftriaxensis
8: Ruminococcaceae_bacterium
9: Subdoligranulum_variabile
10: uncultured_Clostridium_sp
11: Bacteroides_sp_CAG:770
12: Clostridiales_bacterium_Marseille-P2846
13: Bacteroidales_bacterium_55_9
14: Porphyromonadaceae_bacterium_H1
15: Acidiphilium_sp_CAG:727
16: Paludibacter_propionicigenes
17: bacterium_P3
18: Lentisphaerae_bacterium_RIFOXYA12_FULL_48_11
19: Firmicutes_bacterium_CAG:124
20: Bacteroides_sp_CAG:545
21: Alistipes_obesi
22: Victivallales_bacterium_CCUG_44730
23: Alistipes_sp_CAG:514
24: Methanobrevibacter_olleyae
25: Alistipes_sp_CAG:435
26: Clostridiales_bacterium_NK3B98
27: Methanobrevibacter_millerae
28: Firmicutes_bacterium_CAG:110
29: Staphylococcus_aureus
30: Eubacterium_sp_CAG:841
31: Methanobrevibacter_ruminantium
32: Firmicutes_bacterium_CAG:272
33: Ruminococcus_bromii
34: Methanobrevibacter_sp_YE315
35: Victivallis_vadensis
36: Siphoviridae_sp
37: Methanobrevibacter_thaueri
38: Porphyromonadaceae_bacterium_CG2_30_38_12
39: Firmicutes_bacterium
40: uncultured_bacterium
41: Staphylococcus_sp_CAG:324
42: Anaerotruncus_sp_CAG:390
43: Chlamydia_trachomatis
44: Alistipes_sp_CHKCI003
45: Sarcina_sp_DSM_11001
46: Firmicutes_bacterium_CAG:137
47: Firmicutes_bacterium_CAG:110_56_8
48: Treponema_brennaborense
49: Clostridium_sp_CAG:448
50: Ruminococcus_sp_CAG:382
