# Supplementary material for: Multi-omics analyses reveal that the gut microbiome and its metabolites promote milk fat synthesis in Zhongdian yak cows
Source: PeerJ. 2022 Dec 2;10:e14444. doi: 10.7717/peerj.14444 (PMC9744170; doi:10.7717/peerj.14444)
Supplement: Supplemental Information 11 [file peerj-10-14444-s011.zip › network.neededData.zip!/network.pdf]

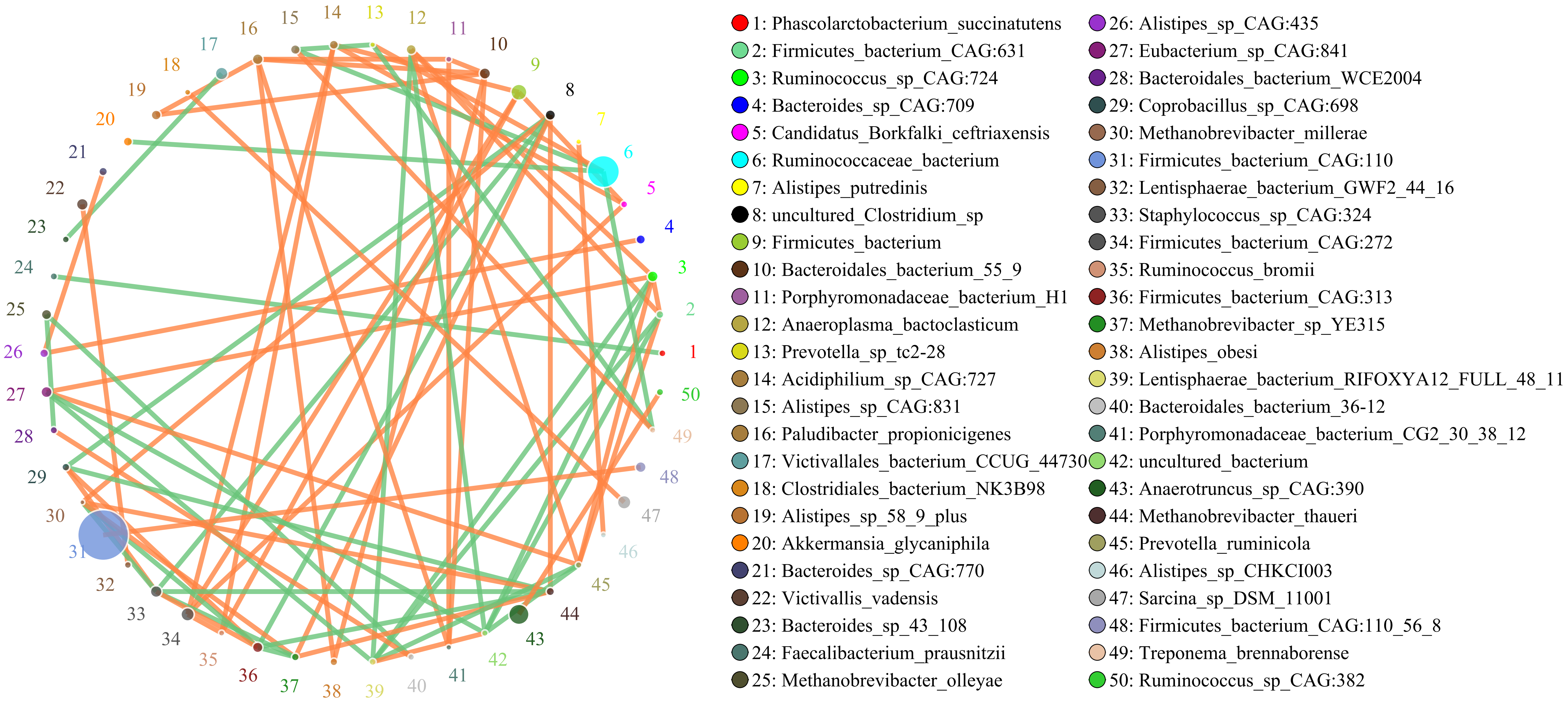
1: Phascolarctobacterium_succinatutens
2: Firmicutes_bacterium_CAG:631
3: Ruminococcus_sp_CAG:724
4: Bacteroides_sp_CAG:709
5: Candidatus_Borkfalki_ceftriaxensis
6: Ruminococcaceae_bacterium
7: Alistipes_putredinis
8: uncultured_Clostridium_sp
9: Firmicutes_bacterium
10: Bacteroidales_bacterium_55_9
11: Porphyromonadaceae_bacterium_H1
12: Anaeroplasma_bactoclasticum
13: Prevotella_sp_tc2-28
14: Acidiphilium_sp_CAG:727
15: Alistipes_sp_CAG:831
16: Paludibacter_propionicigenes
17: Victivallales_bacterium_CCUG_44730
18: Clostridiales_bacterium_NK3B98
19: Alistipes_sp_58_9_plus
20: Akkermansia_glycaniphila
21: Bacteroides_sp_CAG:770
22: Victivallis_vadensis
23: Bacteroides_sp_43_108
24: Faecalibacterium_prausnitzii
25: Methanobrevibacter_olleyae
26: Alistipes_sp_CAG:435
27: Eubacterium_sp_CAG:841
28: Bacteroidales_bacterium_WCE2004
29: Coprobacillus_sp_CAG:698
30: Methanobrevibacter_millerae
31: Firmicutes_bacterium_CAG:110
32: Lentisphaerae_bacterium_GWF2_44_16
33: Staphylococcus_sp_CAG:324
34: Firmicutes_bacterium_CAG:272
35: Ruminococcus_bromii
36: Firmicutes_bacterium_CAG:313
37: Methanobrevibacter_sp_YE315
38: Alistipes_obesi
39: Lentisphaerae_bacterium_RIFOXYA12_FULL_48_11
40: Bacteroidales_bacterium_36-12
41: Porphyromonadaceae_bacterium_CG2_30_38_12
42: uncultured_bacterium
43: Anaerotruncus_sp_CAG:390
44: Methanobrevibacter_thaueri
45: Prevotella_ruminicola
46: Alistipes_sp_CHKCI003
47: Sarcina_sp_DSM_11001
48: Firmicutes_bacterium_CAG:110_56_8
49: Treponema_brennaborense
50: Ruminococcus_sp_CAG:382
